# Antioxidant, anti-inflammatory, cytotoxicity and larvicidal activities of *Ziziphus oenopolia* leaves: *in vitro* and *in silico* studies

**DOI:** 10.1371/journal.pone.0358577

**Published:** 2026-10-30

**Authors:** Shunmuga Vadivu Ramalingam, Vasthi Gnanarani Soloman, Senthil Bakthavatchalam, Karnan Ramachandran, Ramachandran Vinayagam, Zhi-Hong Wen, Chandramohan Govindasamy, Khalid M. Almutairi, Yi-Hao Lo

**Affiliations:** 1 Department of Biochemistry, SRM Dental College, Bharathi Salai, Ramapuram, Chennai, Tamil Nadu, India; 2 Department of Chemistry, Faculty of Engineering and Technology, SRM Institute of Science and Technology, Bharathi Salai, Ramapuram, Chennai, Tamil Nadu, India; 3 Department of Zoology, Annai Vailankanni Arts and Science College, (Affiliated to Bharathidasan University), Thanjavur, Tamil Nadu, India; 4 Department of Biotechnology, Institute of Biotechnology, College of Life and Applied Sciences, Yeungnam University, Gyeongsan, Republic of Korea; 5 Department of Marine Biotechnology and Resources, National Sun Yat-Sen University, Kaohsiung, Taiwan; 6 National Museum of Marine Biology and Aquarium, Pingtung, Taiwan; 7 Department of Community Health Sciences, College of Applied Medical Sciences, King Saud University, Riyadh, Saudi Arabia; 8 Department of Family Medicine, Zuoying Armed Forces General Hospital, Kaohsiung, Taiwan; 9 Department of Nursing, Meiho University, Pingtung, Taiwan; Guru Nanak College, INDIA

## Abstract

The present study investigated the phytochemical composition, *in vitro* and *in silico* biological activities, and insecticidal properties of *Ziziphus oenopolia* (L) Mill. leaf extracts. The LC-MS profile of *Ziziphus oenopolia* leaves extract, which identified major phytochemical classes of fatty acids, flavonoids, and phenolic acids. The GC-MS profile, including n-hexadecanoic acid, phytol, and 9,12,15-octadecatrienoic acid, which were predicted by PASS and ADMET profiling to exhibit strong antioxidant, anti-inflammatory, cytotoxic, and larvicidal activities. The hydroalcoholic extract demonstrated the highest biological efficacy across multiple assays. *In vitro* antioxidant evaluation revealed strong DPPH and ABTS radical scavenging activities with IC₅₀ values of 41.04 and 43.54 µg/mL, respectively. The extract also showed significant protein anti-denaturation effects against bovine serum albumin and egg albumin (IC₅₀ = 315.53 and 311.60 µg/mL), significant cytotoxicity (EC₅₀ = 173.92 µg/mL), and strong larvicidal activity (LC₅₀ = 126.09 µg/mL for *Aedes aegypti* and 136.01 µg/mL for *Tribolium castaneum*), all exhibiting dose-dependent inhibition. *In silico* molecular docking further supported these findings, revealing strong binding affinities of the phytoconstituents toward human peroxiredoxin 5 as an antioxidant target, COX-2 (PDB: 1PXX) as an anti-inflammatory target, EGFR (PDB: 2EB3) as an anticancer target, and juvenile hormone binding protein (PDB: 5V13) as a larvicidal target. Overall, *Z. oenopolia* may contribute to the development of natural antioxidants and anti-inflammatory agents for mitigating oxidative stress-related and chronic inflammatory diseases.

## Introduction

Cellular metabolism continuously generates reactive oxygen species (ROS), which are typically regulated by endogenous antioxidant defense systems to maintain homeostasis. However, excessive ROS production is induced by external factors such as lifestyle, environmental toxins, radiation, unhealthy dietary patterns, and immune responses, which can disrupt this balance, leading to oxidative stress [1]. Persistent oxidative stress results in structural and functional damage to biomolecules, including lipids, proteins, and nucleic acids, thereby accelerating aging and triggering chronic inflammatory responses [2]. Inflammation is a protective immune-mediated process that facilitates tissue repair and defense against harmful stimuli. It is broadly classified as acute and chronic forms. Chronic inflammation is strongly associated with metabolic disorders, cardiovascular disorders, neurodegenerative conditions, and cancer [3]. Notably, oxidative stress and inflammation are interdependent processes that amplify each other, contributing collectively to the onset and progression of various acute and chronic pathologies [4]. Therefore, identifying bioactive agents capable of modulating oxidative stress and inflammatory pathways remains a critical area of biomedical research.

Medicinal plants have served as a primary source of therapeutic agents for the management of diverse diseases, including chronic inflammatory conditions. Traditional knowledge systems have provided a valuable framework for the identification of bioactive phytoconstituents with pharmacological relevance [5]. Numerous plants-derived compound, such as flavonoids, phenolics, alkaloids, and terpenoids, have demonstrated significant antioxidant and anti-inflammatory activities by modulating key signaling pathways involved in oxidative stress and immune responses. Many of these compounds exhibit notable anti-arthritic, anti-inflammatory, and antioxidant properties. The increasing interest in phytochemical screening is driven by the potential of natural products to serve as safer alternatives to synthetic drugs, owing to their relatively low toxicity and minimal side effects [6]. Consequently, systematic evaluation of plant extracts with ethnomedicinal impact is essential for the discovery of novel therapeutic candidates.

*Ziziphus* genus (Rhamnaceae) comprises approximately 135 species known for their rich bioactive compounds, including saponins, tannins, flavonoids, and phenolics. Traditionally, Ziziphus species have been used to treat various ailments, particularly chronic inflammatory diseases, with increasing scientific validation for their ethnomedicinal applications [7]. *Ziziphus oenoplia* (L.) Mill., a thorny shrub from the Rhamnaceae family, grows in forests and thickets across the Indian subcontinent, southern China, Southeast Asia, and northern Australia [8]. Its leaves, stems, roots, and fruits have various traditional uses, including treating wounds (leaves), sore throats, dysentery, and uterine inflammation (stem bark), and aiding wound healing (root bark decoction). The roots are also used for helminthiasis and hyperacidity, while the edible fruits treat stomachaches, coryza, and serve as an aphrodisiac, tonic, and for fevers. Previous phytochemical investigations, including GC-MS analysis, have identified several bioactive compounds in *Z. oenoplia* leaf extracts, exhibiting antimicrobial, antioxidant, anti-inflammatory, analgesic, hypocholesterolemic, and cytotoxic effects [8,9]. From the literature, the pharmacological properties of *Z. Oenoplia*, as a comprehensive study integrating its antioxidant, anti-inflammatory, cytotoxic, and larvicidal activities, were limited within a single experimental framework. Furthermore, the molecular mechanism underlying these bioactivities, particularly interaction with specific protein targets, is not well elucidated. Existing studies predominantly focus on isolation and biological activities without establishing a mechanistic correlation between phytochemical composition and functional efficacy.

The present study was designed to systematically evaluate the antioxidant, anti-inflammatory, cytotoxic, and larvicidal activities of *Z. oenoplia* leaf extract using *in vitro* assays. Additionally, an *in-silico* approach was employed to investigate the interaction of identified phytoconstituents with relevant biological targets, thereby providing mechanistic insights into their pharmacological potential. This integrated approach aims to bridge a gap between traditional knowledge and modern therapeutic validation, supporting the development of plant-based bioactive agents. However, the anti-inflammatory potential of Ziziphus species remains insufficiently explored.

## Materials and methods

### Chemicals required

The chemicals required for the synthesis and biological activity included Gallic acid (SRL Pvt. Ltd.; CAS no: 5995-86-8), Quercetin (Sigma; CAS no: 117-39-5), Linalool (Sigma-Aldrich; CAS no: 78-70-6), 2,2-diphenyl-1-picrylhydrazyl (DPPH) (Alfa Aesar; CAS no: 1898-66-4), 2,2′-azino-bis(3-ethylbenzothiazoline-6-sulfonic acid) (ABTS) (Alfa Aesar; CAS no: 30931-67-0) and ethanol (Sigma Aldrich: CAS no: 64-17-5). All experiments were conducted using deionized water. All chemicals were of analytical grade, procured from the respective suppliers, and used without further purification.

### Collection, extraction, and phytochemical screening of *Ziziphus oenopolia (L.) Mill.* Leaves

The leaves of *Ziziphus oenopolia (L.) Mill. Leaves* were collected from the Thanjavur district in January 2023, and a voucher specimen (Voucher ID: R.K.001; S1 Fig) was deposited at St. Joseph's College, Trichy, Tamil Nadu, India. The leaves (Fig 1A) were air-dried at room temperature for one week and ground into a fine powder. The powder was extracted using aqueous, ethanol, and hydroalcoholic solvents for a qualitative phytochemical screening method (Fig 1B) [10]. The total phenolic content was quantitatively estimated, and the results were reported as mg of gallic acid equivalents (GAEs) per gram in plant materials using the Folin–Ciocalteu assay. The total flavonoid content is expressed as mg of quercetin equivalents (QEs) per gram of plant materials, using aluminium chloride colorimetric assay [11]. The qualitative analysis of terpenoids is expressed as mg of linalool equivalents per gram of plant materials by the methods [12].

**Fig 1:**
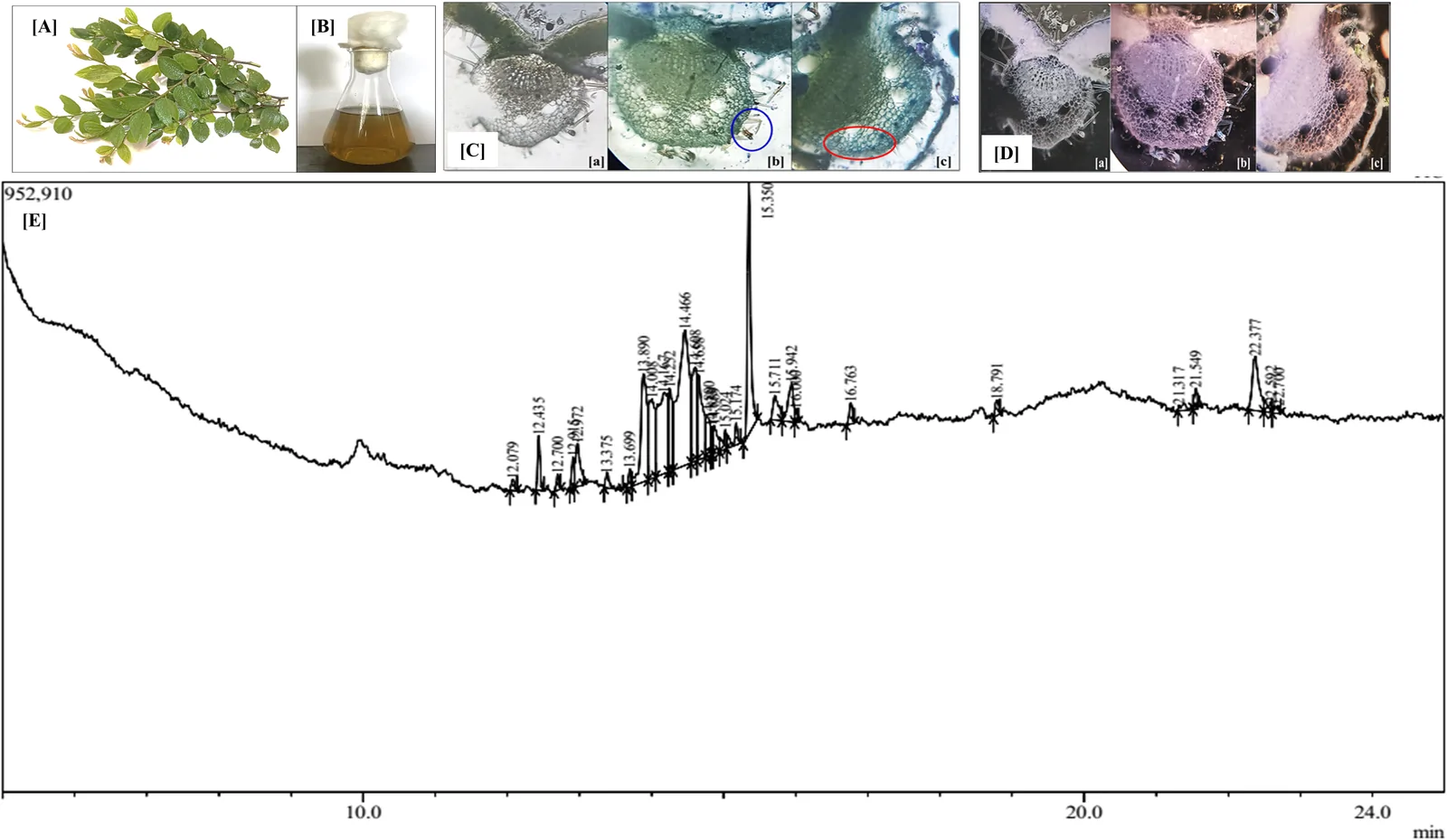
Identification of polyphenols from the leaf of *Z. oenopolia* by GC-MS. (A) *Z. oenopolia* Leaves; (B) Hydro-ethanolic extract. Identification of polyphenols from the leaf of *Z. oenopolia*, using cross-section and Histochemical techniques (C) Without computation; (D) With computation using Imagej tool; a: Without toluidine blue and b-c: With toluidine blue) reveals the presence of polyphenol with green, red, and blue colors. (E) GC-MS chromatogram of *Z. oenopolia* leaves hydroalcoholic extract.

### GC-MS profiling of the leaves extract

GC-MS analysis of the hydroalcoholic extract was performed using JEOL-GC MATE II (Shimadzu GC 2010 Plus Tokyo, Japan). The relative abundance of chemical compounds in the extract was determined as a percentage based on the chromatogram peak areas. Bioactive compounds were detected by comparing GC retention times and spectral data with computer software databases, including Replib and Mainlab standards [13].

### LC-MS profiling of leaves extract

The Liquid chromatography-mass spectroscopy (LC-MS) was performed using a Thermo Scientific Dionex, UltiMate 3000 UHPLC system, which is coupled with an Exactive Plus mass spectrometer (Thermo Fisher Scientific, Germany), and is available at Central University, Thiruvarur, Tamil Nadu, India. A linear trap quadrupole (LTQ) Orbitrap mass spectrometer was used to carry out liquid chromatography mass spectrometry. The instrument was coupled to an Agilent 1200 HPLC system, and it is connected with a photodiode array (PDA) detector. The leaves extract was filtered (0.22 μm), and it was appropriately diluted before injection; an aliquot was introduced into the auto samples. Chromatographic separation was achieved using reverse phase Luna C18 column (60x3mm, 3 μm particle size). A mobile phase consists of water (+0.1% formic acid) A and methanol (+0.1% formic acid) B, at a flow rate of 360 μL/min. The gradient is configured to be a linear gradient from 96% A to 100% B over 14 mins, followed by 100% B for 4 mins, then a return to the initial concentration of 96% A in 0.6 mins, and then it is allowed to equilibrate for 4.6 mins. The column oven temperature was maintained at 30°C, and the injection volume was around 6 μL. The spectrometry analysis was performed in positive electrospray ionisation mode with a nominal mass resolving power of 60000 at 400m/z, spray voltage of 6kV, and a scan rate of 1 Hz. The spectrometer was operated with a capillary temperature of (300 ºC), a tube lens of 100 V Collision-induced dissociation (CID) was performed using argon as the collision gas, where applicable. The identified compounds were tentatively confirmed by comparing their mass spectra, retention times, and fragmentation patterns with published literature and available spectral databases [14,15].

### ADMET and PASS prediction

Analyzing ADME studies is essential to determining the pharmacodynamic properties of the drug. Utilizing the web-based SWISSADME tool and toxicity assessment, the toxicity of metabolites was analyzed and predicted using the online tool ProTox-III [16]. The phytochemicals in *Z. oenopolia* leaves (ZiOL) were identified, and their antioxidant and anti-inflammatory properties were predicted using the online tool PASS (Prediction of Activity Spectra for Substances) as described [17,18].

### *In vitro* DPPH free radical scavenging assay

The free radical scavenging ability of ZiOL was assessed using a method [19] against the 2,2-diphenyl-1-picrylhydrazyl (DPPH) radical. The procedure involved mixing 3 mL of 0.1 mM DPPH solution in methanol with 1 mL of ZiOL extract solution (10–80 µg/mL) and ascorbic acid (20–100 µg/mL). After incubating for 30 minutes at room temperature in the dark, the absorbance of the reaction mixture was measured at 517 nm. The free radical scavenging activity of ZiOL was calculated using the following formula (1).

### *In vitro* ABTS free radical scavenging assay

The ABTS scavenging capacity was assessed [20]. A radical cation was generated by mixing 2.45 mM potassium persulfate with 7 mM ABTS stock solution and incubating the mixture in the dark at room temperature for 10–16 hours. After dilution, the ABTS solution was adjusted to an absorbance of 0.70 ± 0.02 at 734 nm. 3.0 mL portion of this solution was mixed with 1 mL of ZiOL at varying concentrations (10–80 µg/mL), and the absorbance (Spectrophotometer, SYSTRONICS, Ahmedabad, India) was recorded at 734 nm following a 6-minute incubation. Ascorbic acid (20–100 µg/mL) served as the positive control. The scavenging activity was determined using the following formula (1).


$$\begin{array}{c} \%\text{of inhibitions}={\text{A}}_{c}-{\text{A}}_{t}/{\text{A}}_{c}\times \text{100.} \end{array}$$ (1)

where “A_c_” (control) was the absorbance of the ABTS blank solution, and “At” was the final absorbance of the tested sample.

## *In vitro* anti-inflammatory effect of *Z. oenopolia* hydroalcoholic extract against protein denaturation

### *In vitro* egg albumin denaturation assay

*In vitro* anti-inflammatory activity was evaluated using the egg albumin denaturation method [21] with ZiOL hydroalcoholic extract concentrations ranging from 100 to 500 µg/mL. The reaction mixture (5 mL) contained 0.2 mL of egg albumin, 2.8 mL of phosphate-buffered saline (PBS, pH 6.4), and 2 mL of varying concentrations of ZiOL. A similar volume of double-distilled water was used as a control. The mixtures were incubated at 37 °C for 15 mins and then heated at 70 °C for 5 mins. After cooling, absorbance was measured at 660 nm, and viscosity was determined using an Ostwald viscometer. Diclofenac sodium (100–500 µg/mL) served as the reference drug. The percentage inhibition of protein denaturation was calculated using formula (2).


$$\begin{array}{c} \%\text{inhibition}=\text{100}\times ({\text{V}}_{t}/{\text{V}}_{c}-\text{1}) \end{array}$$ (2)

Where, V_t_ = absorbance of test sample, V_c_ = absorbance of control.

### *In vitro* bovine serum albumin (BSA) denaturation assay

The anti-inflammatory activity of ZiOL hydroalcoholic extract was assessed using the bovine serum albumin (BSA) protein anti-denaturation assay [22]. A 2 mL aliquot of ZiOL extract (100–500 µg/mL) was mixed with 1.5 mL of 1% BSA solution in 0.5 mM Tris buffer (pH 6.5) and incubated at 37 ºC for 30 minutes, followed by heating at 70 ºC for 20 minutes. After cooling, absorbance was measured at 660 nm. Diclofenac sodium was used as the reference standard. The procedure was repeated in triplicate for statistical analysis, and the percentage inhibition of protein denaturation was calculated using formula (3).


$$\begin{array}{c} \%\text{Inhibition of BSA denaturation}=({\text{A}}_{c}-{\text{A}}_{s})/{\text{A}}_{c}\text{x 100} \end{array}$$ (3)

Where, A_c_= Control absorbance, and A_s_=Sample absorbance.

### *In vitro* Cytotoxicity (anti-proliferative) activity of *Z. oenopolia* hydroalcoholic extract

The antiproliferative potential of the *Ziziphus oenopolia* leaves (ZiOL) hydroalcoholic extract was evaluated using a yeast cell model following the method [23]. Seeded broth was prepared by inoculating 100 mL of sterilized nutrient broth with 5 g of commercial yeast and incubating it at 37 °C for 24 h. The culture was then diluted with sterile distilled water to achieve approximately 2.54 × 10⁴ cells/mL [24]. Different concentrations of the ZiOL extract (50–250 µg/mL) were each mixed with 0.5 mL of yeast inoculum and 2.5 mL of potato dextrose broth (PDB) in test tubes. Controls contained only yeast inoculum and PDB. After incubation at 37 °C for 24 h, cell suspensions were stained with 0.1% methylene blue and examined microscopically. Viable cells appeared transparent, while non-viable cells stained blue. Cell counts from 16 hemocytometer chambers were averaged, and cell viability (%) was calculated using the formula:


$$\begin{array}{c} \text{Viable cells}/\text{mL }=\text{ average no of viable cells in one square dilution factor }\times {\text{10}}^{\text{4}} \end{array}$$

$$\begin{array}{c} \text{Percentage of cell viability }=\text{ Total viable cells }/\text{ Total cells }\times \text{ 100} \end{array}$$

### Larvicidal activity of *Z. oenopolia* hydroalcoholic extract

#### Mosquito *Aedes aegypti.*

*A. aegypti* larvae were reared in deionized water enriched with glucose and yeast powder under controlled laboratory conditions (27 °C, 75 ± 5% relative humidity, 14 h photoperiod) following standard rearing protocols with minor modifications [25]. The larvicidal potential of the ZiOL hydroalcoholic extract was assessed against fourth instar *A. aegypti* larvae using the World Health Organization [26] bioassay guidelines. Test solutions were prepared at five concentrations (50, 100, 150, 200, and 250 µg/mL) in 100 mL of deionized water within 200 mL glass beakers. Twenty larvae were introduced into each beaker, while control groups containing only deionized water were maintained under identical conditions. All assays were performed in triplicate, and larval mortality was recorded after 24 h of exposure.

#### Storage pest *T. castaneum.*

The fumigant toxicity of the ZiOL hydroalcoholic extract was evaluated against fifth-instar larvae of *Tribolium castaneum*, following the method described [27]. In each 90 mm Petri dish, 1 g of wheat flour was evenly spread and seeded with 20 larvae from laboratory cultures. The inner surface of each dish was coated with ZiOL extract at varying concentrations (50–250 µg/mL), then sealed with parafilm to prevent vapor loss. The plates were maintained in darkness for 24 hours, after which larval mortality was recorded to assess fumigant toxicity.

### Molecular docking

Phytochemical ligands such as 9,12,15-Octadecatrienoic acid, n-Hexadecanoic acid and Phytol were retrieved from the PubChem database, and the molecular targets, human peroxiredoxin 5 (PDB ID: 1HD2) for antioxidant, COX-2 (PDB ID: 1PXX) for anti-inflammatory, EGFR (PDB: 2EB3) for anti-cancer, and juvenile hormone-binding protein (PDB: 5V13) for larvicidal activity, were sourced from the PDB. The ligands were converted into PDB format using OpenBabel software, and water molecules and extraneous ligands were removed from the target protein structure. The prepared protein structure was saved in PDB format using PyMOL software. Molecular docking was performed using PyRx 0.8, utilizing the Autodock Vina program for grid dimension setup as described [28]. Post-docking results were visualized using PyMOL, BIOVIA Discovery Studio Visualizer, and UCSF Chimera 1.14.

### Statistical analysis

All experiments were triplicated (n = 3). Cytotoxicity and Larvicidal data were performed by one-way ANOVA with Tukey's HSD post-hoc test. IC_50_, EC_50,_ and LC_50_ were calculated by regression equations and correlation coefficient (r), using IBM SPSS Version 20.0 software. Significance level 0.05.

## Results and discussion

### Phytochemical profile of *Z. oenopolia* leaves

*Z. oenopolia* leaves extract contained polyphenols, tannins, flavonoids, coumarins, saponins, terpenoids, triterpenoids, steroids, and glycosides, with the highest concentration found in the hydroalcoholic extract (70%), compared to the ethanol and aqueous extracts (Table 1). Fig 1C-1D confirms the presence of phenolic compounds in the leaf cross-section, stained with toluidine blue [26–30]. Phenolic compounds were identified in the plant tissues, stained blue and green by toluidine blue [31]. The significant amounts of total phenols (176.38 ± 4.92 mg gallic acid equivalents per gram), flavonoids (53.26 ± 3.45 mg quercetin equivalents per gram), and terpenoid content (37.45 ± 2.97 mg linalool equivalents per gram) were reported, which significantly contribute to biological activity.

**Table 1:** Qualitative analysis of *Z. oenopolia* leaves extract.

Phytochemicals	Aqueous extract	Ethanolic extract	Hydroalcoholic extract
Polyphenols	++	++	++
Tannins	+	++	++
Flavonoids	++	++	++
Coumarins	++	+	++
Saponins	++	+	++
Alkaloids	–	+	+
Terpenoids	++	++	++
Triterpenoids	++	++	++
Steroids	+	++	++
Glycosides	+	++	++

(+: Present; -: Absent and ++: High concentration).

GC–MS profiling of the hydroalcoholic extract revealed 15 phytochemical constituents (Table 2 and Fig 1E; S2 Data), including several bioactive compounds. Notable constituents identified were Neophytadiene, known for its anthelmintic activity [32]; n-Hexadecanoic acid, exhibiting antioxidant, hypocholesterolemic, nematicidal, pesticidal, lubricating, and antiandrogenic properties [33]; Docosane, with reported antimicrobial potential [31]; Phytol, possessing antimicrobial, anticancer, cancer-preventive, and anti-inflammatory effects [33]; 9-Octadecenal, associated with antimicrobial activity [34]; and 9,12,15-Octadecatrienoic acid, noted for its anti-inflammatory, insect-repellent, hypocholesterolemic, cancer-preventive, nematicidal, hepatoprotective, and antihistaminic properties [33]. *Z. oenopolia* is abundant in bioactive compounds and possesses notable therapeutic potential [35].

**Table 2:** GC-MS analysis of *Z. oenopolia* leaves hydroalcoholic extract.

Peak #	R. Time	Area %	Height %	M. Weight (g/mol)	M. Formula	Phytochemicals
1	12.079	0.47	0.77	–	–	Unidentified
2	12.435	1.79	3.78	278	C_20_H_38_	Neophytadiene
3	12.700	0.59	1.14	296	C_20_H_40_O	3,7,11,15-Tetramethyl-2-hexadecen-1-ol
4	12.915	1.07	2.12	–	–	Unidentified
5	12.972	2.53	2.97	278	C_16_H_22_O_4_	1,2-Benzenedicarboxylic acid, bis(2-methylpropyl) ester
6	13.375	0.79	0.99	312	C_19_H_36_O_3_	Oxiraneundecanoic acid, 3-pentyl-, methyl ester, trans-
7	13.699	0.60	1.26	–	–	Unidentified
8	13.890	8.89	7.41	256	C_16_H_32_O_2_	n-Hexadecanoic acid
9	14.008	6.50	5.49	–	–	Unidentified
10	14.167	10.61	5.56	198	C_14_H_30_	Heptane, 2,2,3,3,5,6,6-heptamethyl
11	14.252	4.06	5.73	–	–	Unidentified
12	14.466	19.61	9.31	380	C_27_H_56_	2-methylhexacosane
13	14.608	6.02	6.46	310	C_22_H_46_	Docosane
14	14.658	5.68	5.82	256	C_16_H_32_O_2_	Isovaleric acid, undecyl ester
15	14.800	2.16	2.56	–	–	Unidentified
16	14.833	0.51	1.94	170	C_10_H_18_O_2_	2(3H)-Furanone, 5-hexyldihydro
17	14.869	1.46	1.94	356	C_22_H_44_O_3_	Eicosanoic acid, 2-hydroxyethyl ester
18	15.024	1.02	1.36	–	–	Unidentified
19	15.174	1.22	1.51	–	–	Unidentified
20	15.350	10.65	17.34	296	C_20_H_40_O	Phytol
21	15.711	1.81	1.68	266	C_18_H_34_O	9-Octadecenal
22	15.942	2.71	2.68	278	C_18_H_30_O_2_	9,12,15-Octadecatrienoic acid
23	16.000	0.40	0.95	156	C_6_H_4_O_5_	Aconitic anhydride
24	16.763	0.87	1.45	–	–	Unidentified
25	18.791	0.72	1.06	–	–	Unidentified
26	21.317	0.53	0.41	–	–	Unidentified
27	21.549	0.83	1.40	–	–	Unidentified
28	22.377	4.67	3.74	–	–	Unidentified
29	22.592	0.73	0.62	–	–	Unidentified
30	22.700	0.51	0.53	–	–	Unidentified

The LC–MS profiling of the *Z. oenopolia* leaves extract revealed the presence of diverse secondary metabolites, such as fatty acids, flavonoids, and phenolic derivatives (Table 3 and and Fig 2A). A total of eight compounds were tentatively annotated based on their accurate mass (m/z) values, retention behavior, and compared with previously reported literature. The chromatographic separation was performed on a reverse-phase C18 column, which enables the detection of the bioactive compounds across a wide range of polarity. Notably, the peaks observed at retention times between 24–29 mins were attributed to phenolic acids such as caffeoylquinic acid (m/z 353) and rosmarinic acid (m/z 359), which are widely reported in the plant metabolite studies.

**Table 3:** LC–MS-based tentative identification of phytochemicals in *Z. oenopolia* leaves extract.

Rt (min)	Observed m/z	Proposed Ion	Molecular Formula	Compound Name	Class	Key Remarks	Reference
6.19	171.14	[M+Na]+	C_8_H_16_O_2_	Caprylic acid	Fatty acid	m/z suggests possible Na⁺ adduct; requires confirmation	[36]
20.64	616.35	[M+H]+	C_27_H_36_O_16_	Luteolin C-hexoside O-hexoside	Flavonoid glycoside	Typical mass for a diglycosylated luteolin derivative	[37]
21.55	149.01	[M–H]^–^ / [M + H]+	C_4_H_6_O_6_	Tartaric acid	Organic acid	Retention time is unusually high for polarity; verify peak purity	[38]
22.45	297.18	[M+H]+	C_17_H_14_O_5_	Dimethoxy apigenin	Flavonoid	Consistent with methoxylated apigenin derivatives	[39]
22.95	327.00	[M–H]^–^	C_18_H_32_O_5_	Trihydroxy-octadecadienoic acid	Oxidized fatty acid	Likely oxidized linoleic acid derivative; needs MS/MS	[40]
23.56	317.17	[M+H]+	C_16_H_12_O_7_	Monomethoxy-tetrahydroxy flavone	Flavonoid	Fits flavone backbone; positional isomer possible	[38]
24.55	359.24	[M–H]^–^ / [M + H]+	C_18_H_16_O_8_	Rosmarinic acid	Phenolic acid	Well-known plant metabolite; RT consistent	[41]
29.25	353.26	[M–H]^–^	C_16_H_18_O_9_	Caffeoylquinic acid	Phenolic acid	Diagnostic compound; typical plant phenolic	[42]

**Fig 2:**
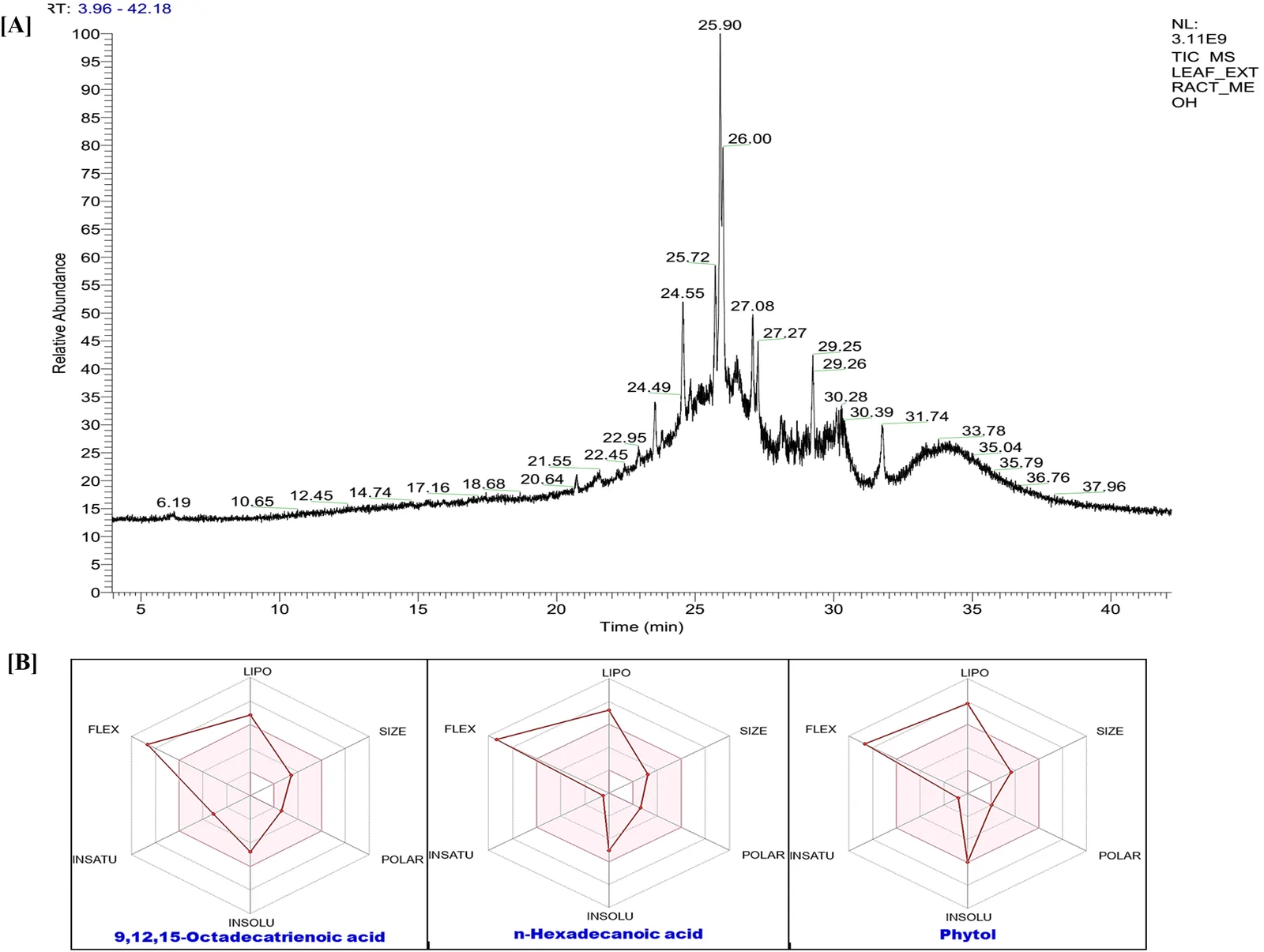
*Z. oenopolia* leaves extract and Bioavailability. (A) LC-MS fingerprint of *Z. oenopolia* leaves extract. (B) The Bioavailability Radar for the relevant metabolites of *Z. oenopolia* leaves hydroalcoholic extract *in vitro* and *in silico* antioxidant activity of *Z. oenopolia* leaves.

Flavonoid-related compounds such as luteolin C-hexoside (m/z 616), dimethoxy apigenin (m/Z 297), and monomethoxy-tetrahydroxy flavone (m/z 317) were also identified within the expected mass range for flavone derivatives and glycosylated flavonoids. However, structural confirmation of such metabolites typically relies on the fragmentation pathways, like the loss of sugar moieties or the formation of aglycon ions, which is not examined in the present analysis.

Organic acids and fatty acid derivatives such as tartaric acid (m/z 149) and trihydroxy-octadecadienoic acid (m/z 327) further support the presence of primary and oxidized lipid metabolites. Nevertheless, the retention time of certain highly polar compounds, including tartaric acid, appears relatively delayed under the described chromatographic conditions, which may indicate co-elution effects or the presence of structurally related isomers. It is important that the compounds are annotated in this study by matching observed m/z values with the reported literature. In addition, the ionization mode and possible adduct formation were not explicitly considered, which may influence the observed m/z values for certain compounds.

### ADMET and PASS profile of *Z. oenopolia* leaves

The identification of active molecules and the responsible use of standard medications are guided by *in silico* profiling [43]. SwissADME and ProTox-III were employed to determine the ADMET characteristics of the selected phytoconstituents from *Ziziphus oenopolia* leaves (Table 4). The selected phytochemicals 9,12,15-Octadecatrienoic acid, n-Hexadecanoic acid, and Phytol complied with Lipinski’s rule of five, indicating favorable drug-likeness and suitability for oral administration. Toxicity prediction revealed these compounds to be non-hepatotoxic, non-neurotoxic, non-nephrotoxic, and devoid of respiratory or cardiotoxic effects, with LD₅₀ values ranging from 900 to 10,000 mg/kg. The absence of organ-specific toxicity suggests that the phytoconstituents of *Z. oenopolia* leaf hydroalcoholic extract exhibit a safe profile, supporting their potential applicability in therapeutic formulations for human disease management. Fig 2B, which is a visual representation, frequently in the form of a radar chart, that shows the expected ADMET, illustrates the bioavailability radar for the relevant metabolites. The majority of the phytocompounds’ ADMET characteristics fell within the suggested ranges, making them viable options for drug development [44]. PASS prediction analysis revealed the anti-inflammatory, antioxidant, cytotoxicity, and insecticidal potential of the phytoconstituents in *Z. oenopolia* leaf extracts, which was further validated by *in vitro* studies (Table 5). PASS was employed to assess the antioxidant (0.475 to 0.222 range of Pa), anti-inflammatory (0.804 to 0.458 range of Pa), cytostatic (0.487 to 0.262 range of Pa), and insecticidal (0.405 to 0.214 range of Pa) properties of the phytochemicals, and the findings were utilized in multiple analyses. Jamkhande et al. identified compounds with higher Pa values than Pi [18]. The *in silico* PASS predictions aligned with the *in vitro* results [45].

**Table 4:** ADMET analysis of *Z.s oenopolia* leaves bioactive compounds.

Parameters	9,12,15-Octadecatrienoic acid	n-Hexadecanoic acid	Phytol
**Physicochemical Properties**			
Formula	C_18_H_30_O_2_	C_16_H_32_O_2_	C_20_H_40_O
Molecular weight (g/mol)	278.43	256.42	296.53
Num. heavy atoms	20	18	21
Num. arom. heavy atoms	0	0	0
Fraction Csp3	0.61	0.94	0.90
Num. rotatable bonds	13	14	13
Num. H-bond acceptors	2	2	1
Num. H-bond donors	1	1	1
Molar Refractivity	88.99	80.80	98.94
TPSA (Å²)	37.30	37.30	20.23
**Lipophilicity**			
Log *P*_o/w_ (iLOGP)	3.36	3.85	4.85
Log *P*_o/w_ (XLOGP3)	6.46	7.17	8.19
Log *P*_o/w_ (WLOGP)	5.66	5.55	6.36
Log *P*_o/w_ (MLOGP)	4.38	4.19	5.25
Log *P*_o/w_ (SILICOS-IT)	5.59	5.25	6.57
Consensus Log *P*_o/w_	5.09	5.20	6.25
**Water Solubility**			
Log *S* (ESOL)	−4.78	−5.02	−5.98
Solubility	4.64e-03 mg/ml	2.43e-03 mg/ml	3.10e-04 mg/ml
Class	Moderately soluble	Moderately soluble	Moderately soluble
Log *S* (Ali)	−7.04	−7.77	−8.47
Solubility	2.55e-05 mg/ml	4.31e-06 mg/ml	9.94e-07 mg/ml
Class	Poorly soluble	Poorly soluble	Poorly soluble
Log *S* (SILICOS-IT)	−3.96	−5.31	−5.51
Solubility	3.08e-02 mg/ml	1.25e-03 mg/ml	9.06e-04 mg/ml
Class	Soluble	Moderately soluble	Moderately soluble
**Pharmacokinetics**			
GI absorption	High	High	Low
BBB permeant	Yes	Yes	No
P-gp substrate	No	No	Yes
CYP1A2 inhibitor	Yes	Yes	No
CYP2C19 inhibitor	No	No	No
CYP2C9 inhibitor	Yes	Yes	Yes
CYP2D6 inhibitor	No	No	No
CYP3A4 inhibitor	No	No	No
Log *K*_p_ (skin permeation)	−3.41 cm/s	−2.77 cm/s	−2.29 cm/s
**Druglikeness**			
Lipinski	Yes; 1 violation: MLOGP>4.15	Yes; 1 violation: MLOGP>4.15	Yes; 1 violation: MLOGP>4.15
Ghose	No; 1 violation: WLOGP>5.6	Yes	No; 1 violation: WLOGP>5.6
Veber	No; 1 violation: Rotors>10	No; 1 violation: Rotors>10	No; 1 violation: Rotors>10
Egan	Yes	Yes	No; 1 violation: WLOGP>5.88
Muegge	No; 1 violation: XLOGP3 > 5	No; 1 violation: XLOGP3 > 5	No; 2 violations: XLOGP3 > 5, Heteroatoms<2
Bioavailability Score	0.85	0.85	0.55
**Medicinal Chemistry**			
PAINS	0 alert	0 alert	0 alert
Brenk	1 alert: isolated_alkene	0 alert	1 alert: isolated_alkene
Leadlikeness	No; 2 violations: Rotors>7, XLOGP3 > 3.5	No; 2 violations: Rotors>7, XLOGP3 > 3.5	No; 2 violations: Rotors>7, XLOGP3 > 3.5
Synthetic accessibility	3.03	2.31	4.30
**Toxicity (Prediction/ Probability)**			
**LD50 (mg/kg)**	10000	900	5000
**Hepatotoxicity**	Inactive/0.54	Inactive/0.52	Inactive/0.79
**Neurotoxicity**	Inactive/0.92	Inactive/0.92	Inactive/0.78
**Nephrotoxicity**	Inactive/0.50	Inactive/0.53	Inactive/0.74
**Respiratory toxicity**	Inactive/0.72	Inactive/0.85	Inactive/0.81
**Cardiotoxicity**	Inactive/0.98	Inactive/0.99	Inactive/0.84

**Table 5:** PASS prediction of *Z. oenopolia* leaves bioactive compounds.

Phytochemicals	Antioxidant		Anti-inflammatory		Insecticide		Cytostatic	
	Pa	Pi	Pa	Pi	Pa	Pi	Pa	Pi
**9,12,15-Octadecatrienoic acid**	0.364	0.015	0.804	0.006	0.214	0.033	0.262	0.087
**n-Hexadecanoic acid**	0.222	0.045	0.515	0.052	0.293	0.018	0.408	0.041
**Phytol**	0.475	0.008	0.458	0.070	0.405	0.006	0.487	0.027

Antioxidants are crucial in mitigating the harmful effects of free radicals on metabolism. Common methods to assess antioxidant capacity include the DPPH and ABTS assays [46,47]. The antioxidant potential of plant extracts in reducing DPPH has been explored [48]. The hydroalcoholic extract of *Z. oenopolia* leaves demonstrated significant dose-dependent inhibition in both DPPH and ABTS assays, with IC_50_ values of 41.04 and 43.54 µg/ml, respectively, compared to 18.08 and 21.48 µg/ml for ascorbic acid (R^2^ = 0.92 to 0.96) (Fig 3A-3B). The extract's potent antioxidant activity is attributed to its phenolic compounds, which facilitate radical scavenging by reducing DPPH and ABTS radicals, leading to a color change from purple to yellow and blue to colorless, respectively [47,49]. The presence of hydrogen-donating antioxidants like flavonoids and phenols further enhances the scavenging activity [47].

**Fig 3:**
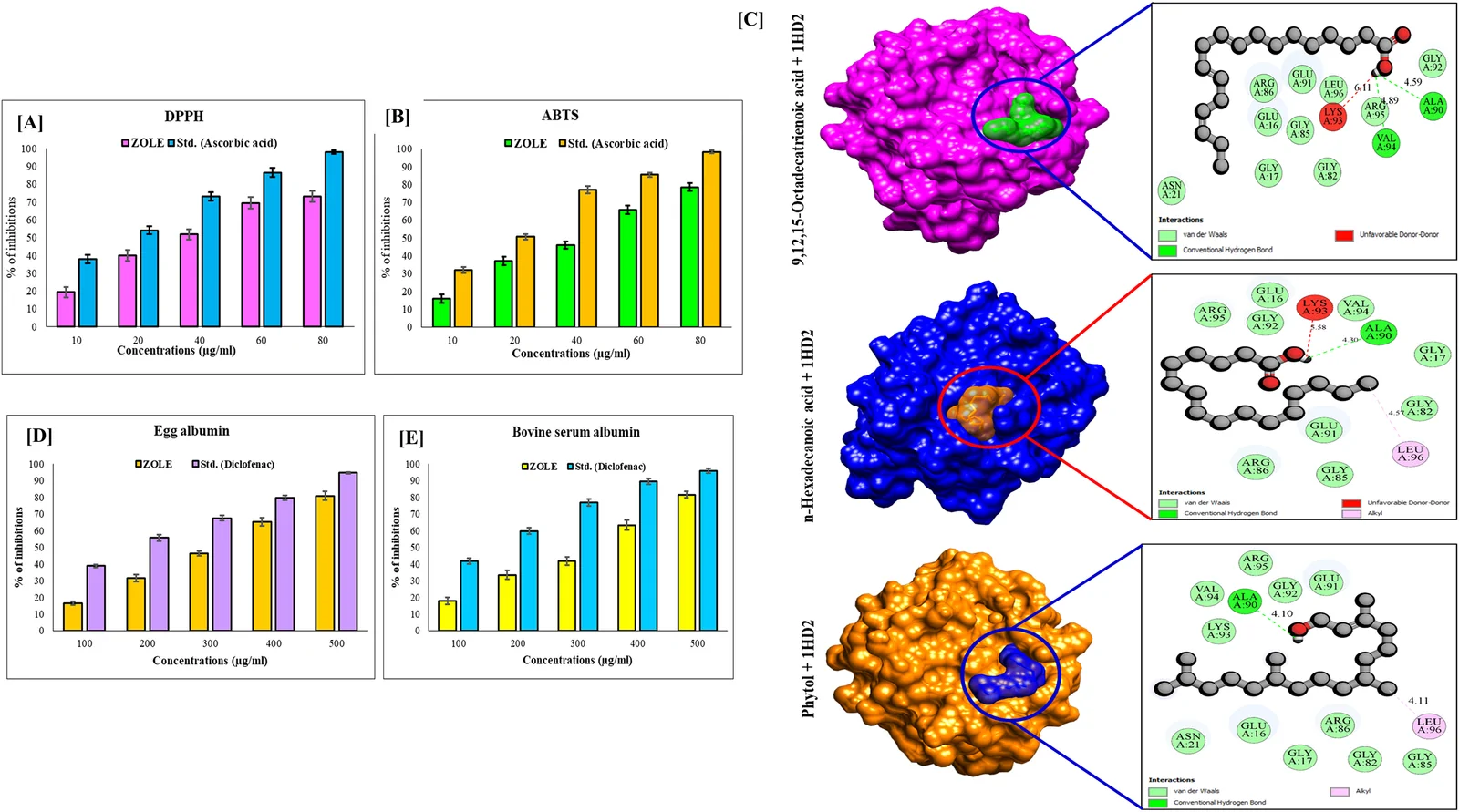
*In vitro* anti-oxidant activity of *Z. oenopolia* leaves hydroalcoholic extract. (A) DPPH; (B) ABTS inhibitory activity); (C) Molecular docking of 9,12,15-Octadecatrienoic acid, n-Hexadecanoic acid, and Phytol against the antioxidant target of human peroxidase enzyme (PDB ID: 1HD2); (D-E) *In vitro* anti-inflammatory activity of *Z. oenopolia* leaves hydroalcoholic extract; (D) Egg albumin; (E) BSA protein anti-denaturation assay.

Peroxiredoxin 5 (PDB: 1HD2) is an antioxidant enzyme that facilitates peroxide reduction and modulates redox signaling, positioning it as a promising target for innovative antioxidant research [50]. This enzyme plays a crucial role in cellular responses to oxidative stress [51,52]. *In silico* molecular docking studies were conducted to explore the interactions between selected phytochemicals from *Z. oenopolia* leaves and Human Peroxiredoxin 5 (PDB ID: 1HD2) [53]. The docking results revealed that the phytochemicals exhibit strong binding affinities, supporting their role as natural antioxidants (Table 6). The identified phytochemicals interact with critical binding sites (e.g., Gly 92, Val 94, Arg 95, Leu 96, Glu 16, Gly 17, Gly 82, Gly 85, Glu 91, Arg 86, and Ala 90), suggesting a similar mode of action on antioxidant molecular targets (Fig 3C). These findings align with the *in vitro* antioxidant activity, providing evidence that *Z. oenopolia* phytochemicals could reduce oxidative stress-related diseases [54,55].

**Table 6:** *In silico* antioxidant activity of *Z. oenopolia* leaf phytochemicals on the human peroxidase enzyme (PDB ID: 1HD2).

Phytochemicals	Binding Affinity (Kcal/mol)	Molecular anti-oxidant target of human peroxidase enzyme (PDB ID: 1HD2) binding to the Amino acid residue
9,12,15-Octadecatrienoic acid	−4.90	Asn 21, Gly 17, Gly 82, Gly 85, Glu 16, Arg 86, Glu 91, Leu 96, Lys 93, Arg 95, Val 94, Ala 90, Gly 92.
n-Hexadecanoic acid	−4.40	Arg 95, Gly 92, Glu 16, Lys 93, Val 94, Ala 90, Gly 17, Gly 82, Leu 96, Gly 85, Glu 91, Arg 86.
Phytol	−4.60	Asn 21, Glu 16, Gly 17, Arg 86, Gly 82, Gly 85, Leu 96, Glu 91, Gly 92, Arg 95, Val 94, Ala 90, Lys 93.

### *In vitro* anti-inflammatory activity of *Z. oenopolia* leaves hydroalcoholic extract

Rheumatoid arthritis (RA) is an autoimmune inflammatory disorder. Medicinal herbs offer a potential treatment option for this condition [10]. Inflammation is linked to pain, which can lead to protein denaturation [56]. Plant extracts with significant inhibition of protein denaturation may have anti-inflammatory properties [57].

*In vitro* studies evaluated the anti-inflammatory effects *of Z. oenopolia* leaves hydroalcoholic extract compared to the reference drug diclofenac sodium. The study measured the inhibition of egg albumin and bovine serum albumin denaturation. Both diclofenac and the *Z. oenopolia* extract demonstrated dose-dependent inhibition of protein denaturation. The half-maximal inhibitory concentration (IC_50_) values for diclofenac were 172.67 µg/ml for egg albumin and 135.09 µg/ml for bovine serum albumin. The IC_50_ values for the *Z. oenopolia* extract were 311.60 µg/ml and 315.53 µg/ml, respectively. The results showed a significant dose-dependent relationship (R^2^ = 0.96 to 0.99) between the extract concentration and protein denaturation inhibition (Fig 3D-3E).

Ranaweera et al. reported an IC_50_ value of 179.20 µg/ml for diclofenac sodium *in vitro* egg albumin denaturation [58], similar to the present study. Aqueous, methanol, dichloromethane (DCM), and hexane leaf extracts of *J. zeylanica* exhibited IC_50_ values of 1297, 26.14, 568.1, and 2.190 µg/ml, respectively [58]. Diclofenac sodium demonstrated maximum inhibition of BSA denaturation (90–100%) at a concentration of 500 µg/mL, likely contributing to its potent anti-inflammatory effects [59].

The anti-inflammatory properties of hydro-methanolic extracts from *Cassia auriculata, Passiflora edulis, Sesbania grandiflora, Olax zeylanica, Gymnema lactiferum,* and *Centella asiatica* leaves can be attributed to their bioactive compounds [60]. Furthermore, Artemisia scoparia crude extract exhibited significant inhibition of thermally-induced protein denaturation [61]. Similarly, *Z. oenopolia* enhanced protein denaturation inhibition using egg albumin and bovine serum albumin, potentially reducing chronic diseases. This effect may be attributed to the phytochemicals present in *Z. oenopolia*, as confirmed by *in silico* studies.

Cyclooxygenase-2 (COX-2), a key enzyme in inflammation, is a common therapeutic target. COX-2 inhibition is central to the action of many pain and fever medications. Rana et al. reported that numerous phytochemicals possess anti-inflammatory properties through COX-2 inhibition [62]. Molecular docking studies suggest that phytochemicals from *Ziziphus oenopolia* leaves exhibit strong potential for COX-2 inhibition (Table 7 and Fig 4A). Malladi et al. established that effective COX-2 inhibitors are anti-inflammatory compounds that block the COX-2 enzyme (PDB: 1PXX) [63]. *In silico* molecular docking studies predicted potential anti-inflammatory activity for the identified bioactive compounds [64]. The anti-inflammatory and antioxidant properties of *Z. oenopolia* leaf extracts and their constituent phytochemicals provide scientific support for its traditional medicinal uses [65]. Bioactive plant components possess health-promoting qualities [66]. Overall, the findings from *Z. oenopolia* leave demonstrate potential for the development of novel medications to treat oxidative stress-related diseases.

**Table 7:** In silico anti-inflammatory activity of *Z. oenopolia* leaves phytochemicals on COX-2.

Phytochemicals	Binding Affinity (Kcal/mol)	Molecular anti-inflammatory target of COX-2 (PDB ID: 1PXX) binding Amino acid residue
9,12,15-Octadecatrienoic acid	−5.70	Cys 159, Ala 156, Cys 36, Gly 135, Arg 44, Cys 41, Pro 153, Gln 461, Leu 152, Gly 45, Glu 46, Tyr 130, Cys 47, Asn 39, Cys 37, Val 155.
n-Hexadecanoic acid	−4.70	Val 291, Phe 210, Val 447, Tyr 385, Thr 206, His 386, His 388, His 207, Lys 211, His 214, Asn 382, Tyr 148, Thr 212.
Phytol	−6.20	Asn 39, Pro 154, Cys 36, Cys 47, Glu 46, Pro 153, Gln 461, Gly 45, Tyr 130, Leu 152, Arg 469, Arg 44, Glu 465, Lys 468, Gln 42, Asn 43, Ala 156, Gly 135, Val 155, Cys 37.

**Fig 4:**
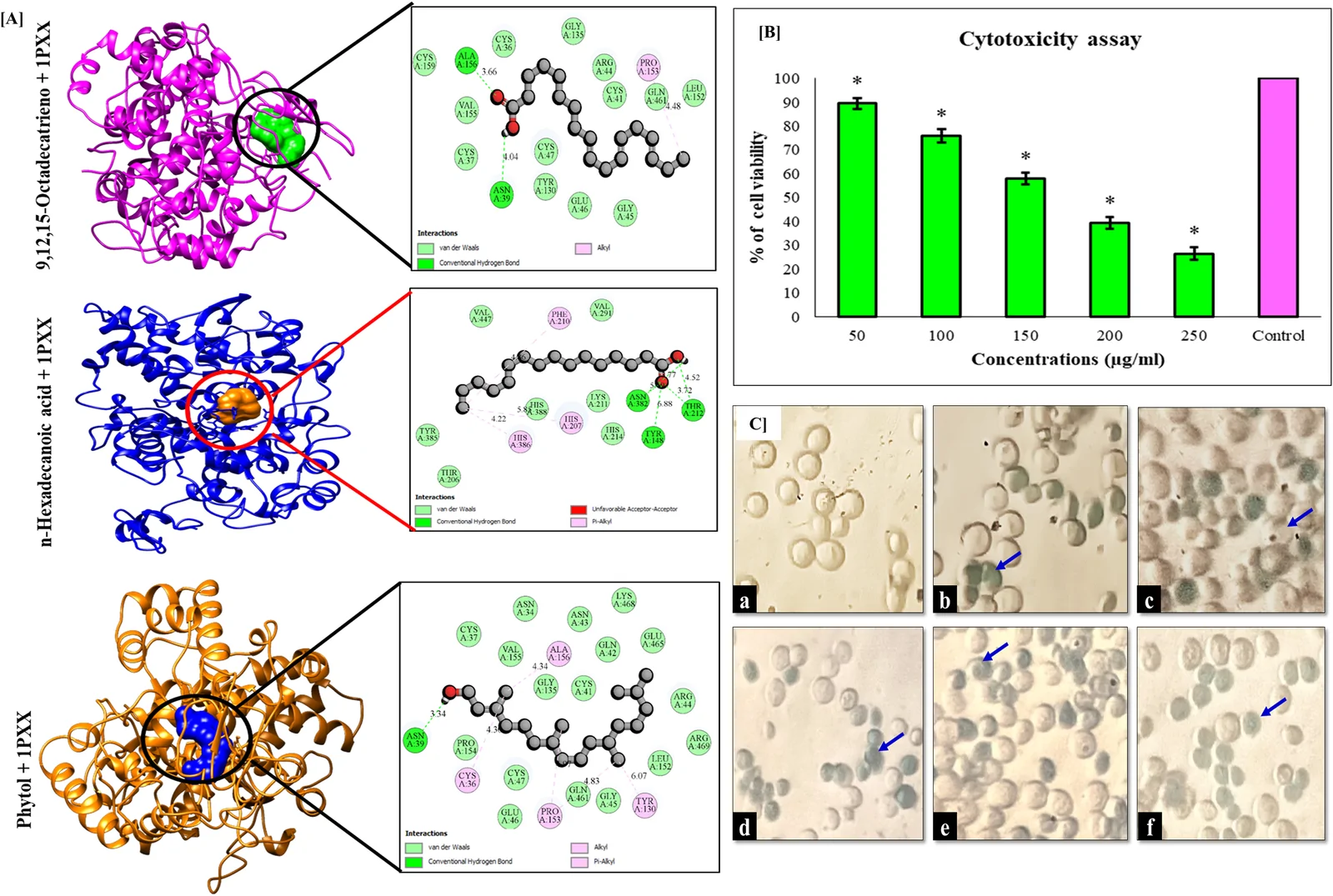
Molecular docking and cytotoxicity. (A) Molecular docking of 9,12,15-Octadecatrienoic acid, n-Hexadecanoic acid, and Phytol against the anti-inflammatory target of COX-2 (PDB ID: 1PXX). (B) Cytotoxicity activity of *Z. oenopolia* leaves hydroalcoholic extract. (C) Morphological presentation of cytotoxicity, blue arrow marks dead cell; a: control; b to f: various concentrations of *Z. oenopolia* leaves hydroalcoholic extract (50–250 µg/mL); blue colour cell death cell; without blue colour cell live cell; blue arrow indicates death cell. *statistically significant from control (*P* < 0.05).

### Cytotoxicity activity of *Z. oenopolia* leaves hydroalcoholic extract

Cytotoxic agents inhibit key cellular pathways and genes essential for cancer cell proliferation [67]. Plants serve as a vital reservoir of novel bioactive molecules and continue to drive innovation in cancer research, offering safer, eco-friendly, cost-effective, and less toxic alternatives to conventional chemotherapeutics [68]. In the present study, the cytotoxic potential of *Ziziphus oenopolia* leaf hydroethanolic extract was investigated using both *in vitro* and *in silico* approaches. The *Saccharomyces cerevisiae* yeast model, a well-established system for cytotoxicity assessment, demonstrated a clear dose-dependent reduction in cell viability upon treatment with the extract (R^2^ = 0.9), with an EC₅₀ value of 173.92 µg/mL. Maximum cytotoxicity was observed at 250 µg/mL, where cell viability decreased to 26.41 ± 2.62%, compared with the control (100 ± 0.00%) (*p* < 0.05; Fig 4B). Microscopic examination further revealed distinct morphological alterations, with methylene blue-stained cells indicating loss of viability (Fig 4C).

*In silico* molecular docking supported the *in vitro* results, showing that major phytochemicals 9,12,15-octadecatrienoic acid, n-hexadecanoic acid, and phytol exhibited inhibitory interactions with the epidermal growth factor receptor (EGFR) (PDB ID: 2EB3) (Table 8). Among these, 9,12,15-octadecatrienoic acid showed the strongest binding affinity (–5.70 kcal/mol), followed by phytol (–5.50 kcal/mol) and n-hexadecanoic acid (–4.80 kcal/mol). The 2D and 3D molecular docking visualizations (Fig 5A) revealed stable ligand–receptor interactions, suggesting effective blockade of EGFR signaling.

**Table 8:** *In silico* cytotoxicity activity of *Z.oenopolia* leaves phytochemicals on EGFR (PDB: 2EB3).

Phytochemicals	Binding Affinity (Kcal/mol)	Molecular cytotoxicity target of EGFR (PDB: 2EB3) binding Amino acid residue.
9,12,15-Octadecatrienoic acid	−5.70	Leu 844, Gly 796, Leu 792, Met 793, Thr 790, Leu 788, Ile 789, Lys 745, Ile 744, Ala 743, Val 726, Leu 718, Gly 719.
n-Hexadecanoic acid	−4.80	Lys 745, Met 766, Leu 792, Leu 718, Thr 854, Asp 855, Leu 844, Arg 841, Asn 842, Gly 796, Val 726, Met 793, Ala 743, Thr 790, Leu 788, Ile 744.
Phytol	−5.50	Gly 719, Leu 792, Leu 718, Ala 743, Met 793, Thr 790, Ile 789, Leu 788, Ile 744, Lys 745, Met 766, Cys 797, Val 726, Gly 796, Thr 854, Leu 844.

**Fig 5:**
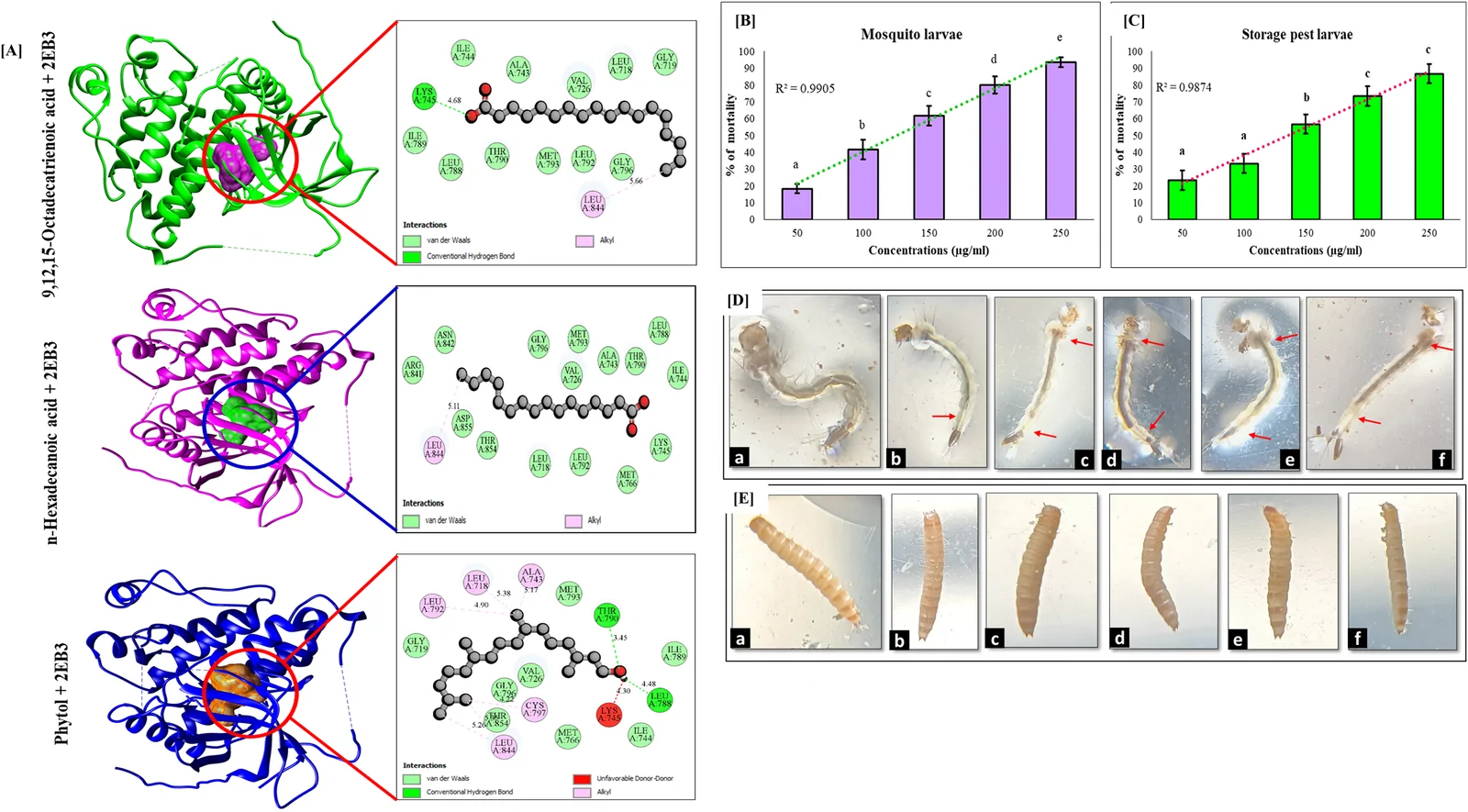
Molecular docking and *the effect of Z. oenopolia leaves hydroalcoholic extract* on insect larvae toxicity. (A) Molecular docking of 9,12,15-Octadecatrienoic acid, n-Hexadecanoic acid, and Phytol on EGFR (PDB: 2EB3). (B-C) Larvicidal activity of *Z. oenopolia* leaves hydroalcoholic extract (B: *Aedes aegypti* larvae toxicity; C: *Tribolium castaneum larvae toxicity)*. (D-E) Morphological observation of *Z. oenopolia* leaves hydroalcoholic extract effect on insect larvae toxicity (D: *Aedes aegypti* larvae alteration; E: *Tribolium castaneum larvae* alteration; a: Control; b to f: various concentrations of *Z. oenopolia* leaves hydroalcoholic extract (50–250 µg/mL); red colour mark indicates larvae outer layer damage and intestine alterations*).*

EGFR is a critical regulator of cell growth, proliferation, and differentiation [69], and its inhibitors (EGFRIs) represent a cornerstone of targeted cancer therapies [70]. The observed inhibitory potential of *Z. oenopolia* phytoconstituents against EGFR aligns with previously reported cytotoxic plant-derived compounds such as those from *Artemisia monosperma*, *Ochradenus baccatus*, *Pulicaria glutinosa*, *Calendula arvensis*, and *Solanum dasyphyllum* [71,72]. Henceforth, these findings suggest that the *Z. oenopolia* leaf extract possesses promising cytotoxic and anticancer potential, primarily attributed to its bioactive constituents phytol and 9,12,15-octadecatrienoic acid, which may serve as safe and effective candidates for future anticancer drug development.

### Larvicidal activity of *Z. oenopolia* leaves hydroalcoholic extract

The hydroalcoholic extract of *Z. oenopolia* leaves demonstrated potent larvicidal activity against two model organisms, the mosquito *Aedes aegypti* and the storage pest *Tribolium castaneum* (Figs 5B and 5C). The extract exhibited a dose-dependent toxic response (R² = 0.9), with *A. aegypti* larvae showing higher susceptibility (LC₅₀ = 126.09 µg/mL) compared to *T. castaneum* larvae (LC₅₀ = 136.01 µg/mL). *Z. oenopolia* leaves’ hydroalcoholic extract, between the concentrations of 50 and 250 µg/mL, was statistically significant (p < 0.05) in the *A. aegypti* larvicidal assay. *T. castaneum* larvicidal assay homogeneous subsets are three categories, such as the 50–100 µg/mL concentration of *Z. oenopolia*, which are non-significant (p > 0.05) compared to the 150–250 µg/mL concentration of *Z. oenopolia*. The 200–250 µg/mL concentration of *Z. oenopolia* is non-significant (p > 0.05), while R² = 0.9, using one-way ANOVA with Tukey's HSD post-hoc test*.* Morphological analysis (Fig 5D-5E) revealed significant larval deformities and mortality following treatment, confirming the extract’s toxic effect on insect physiology.

*In silico* docking further substantiated the larvicidal efficacy of *Z. oenopolia* phytochemicals by targeting the juvenile hormone-binding protein (JHBP; PDB ID: 5V13), a key regulator of insect metamorphosis. Among the major constituents, 9,12,15-octadecatrienoic acid showed the strongest binding affinity (–8.40 kcal/mol), followed by n-hexadecanoic acid (–7.60 kcal/mol) and phytol (–6.30 kcal/mol). The 2D and 3D molecular interaction profiles (Table 9; Fig 6) revealed stable hydrogen bonding and hydrophobic interactions within the JHBP active site, supporting the inhibitory potential of these compounds.

**Table 9:** *In silico* cytotoxicity activity of *Z. oenopolia* leaves phytochemicals on EGFR (PDB: 2EB3).

Phytochemicals	Binding Affinity (Kcal/mol)	Molecular cytotoxicity target of EGFR (PDB: 2EB3) binding, Amino acid residue.
9,12,15-Octadecatrienoic acid	−5.70	Leu 844, Gly 796, Leu 792, Met 793, Thr 790, Leu 788, Ile 789, Lys 745, Ile 744, Ala 743, Val 726, Leu 718, Gly 719.
n-Hexadecanoic acid	−4.80	Lys 745, Met 766, Leu 792, Leu 718, Thr 854, Asp 855, Leu 844, Arg 841, Asn 842, Gly 796, Val 726, Met 793, Ala 743, Thr 790, Leu 788, Ile 744.
Phytol	−5.50	Gly 719, Leu 792, Leu 718, Ala 743, Met 793, Thr 790, Ile 789, Leu 788, Ile 744, Lys 745, Met 766, Cys 797, Val 726, Gly 796, Thr 854, Leu 844.

**Fig 6:**
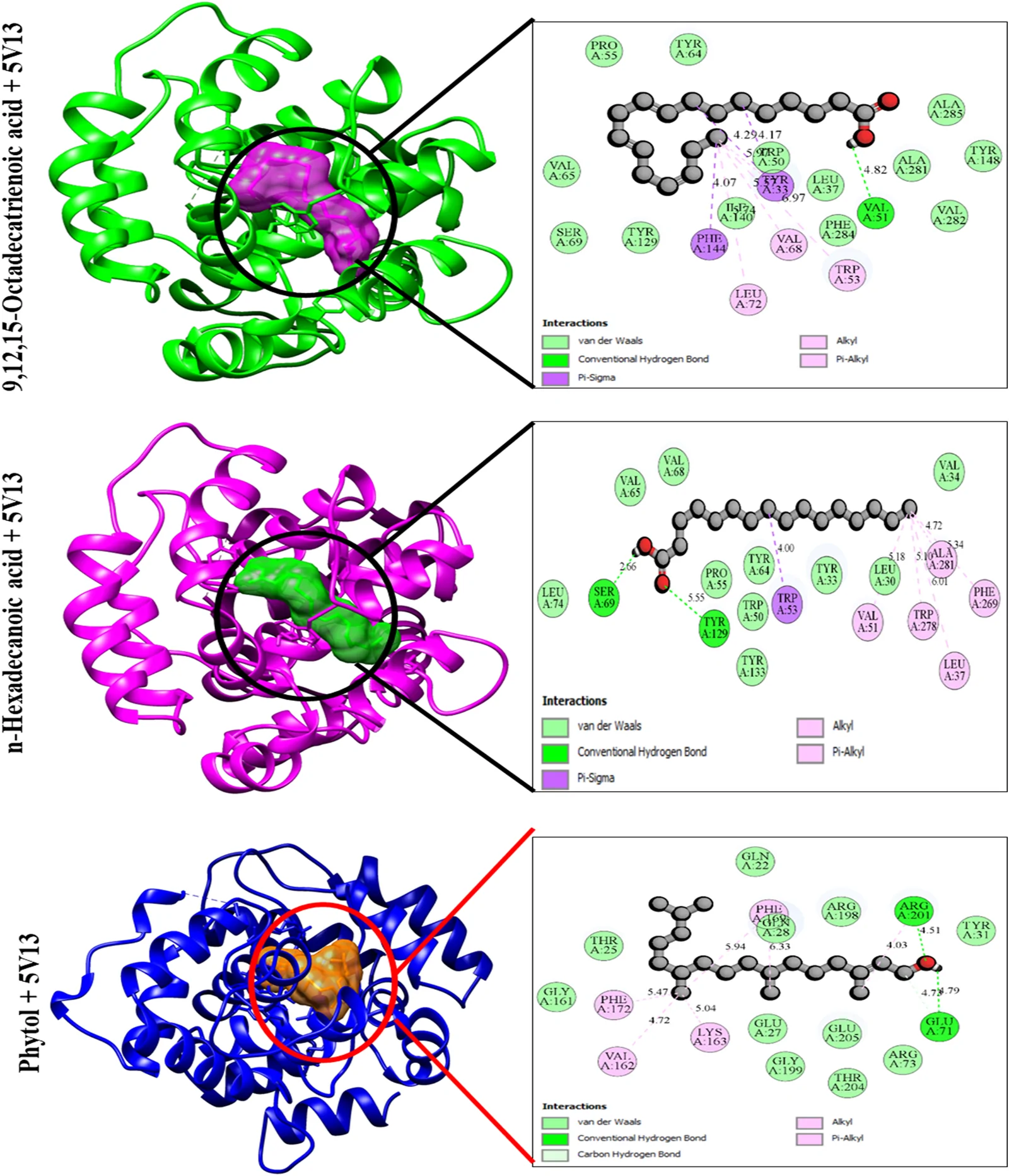
*In silico* larvicidal activity of 9,12,15-Octadecatrienoic acid, n-Hexadecanoic acid, and Phytol on juvenile hormone-binding protein (PDB: 5V13).

Plant-derived larvicides are increasingly recognized as eco-friendly and sustainable alternatives to chemical insecticides [73,74]. Several botanical extracts have shown comparable activity, including *Tridax procumbens* (LC₅₀ = 219 µg/mL), *Nerium oleander* (LC₅₀ = 158.92 µg/mL), and *Agave angustifolia* (LC₅₀ = 259.18 µg/mL) [75–77]. Moreover, phytoconstituents such as alkaloids have been reported to inhibit the mosquito juvenile hormone-binding protein (mJHBP), suggesting their potential as natural insect growth regulators [78,79].

The present findings indicate that *Z. oenopolia* leaf extract exerts significant larvicidal activity both experimentally and computationally. The presence of key bioactive compounds, particularly 9,12,15-octadecatrienoic acid, n-hexadecanoic acid, and phytol, appears to interfere with insect hormonal regulation, thereby disrupting larval development and metamorphosis. These results highlight *Z. oenopolia* as a promising source of plant-based bio-insecticidal agents for environmentally safe vector and pest control.

## Conclusions

*Z.oenopolia* leaves exhibited a rich phytochemical profile in the hydroalcoholic extract compared to ethanol and aqueous extracts. The significant amounts of total phenols, flavonoids, and terpenoids were quantified with the LC-MS profile of *Z.oenopolia* leaves extract, which identified major phytochemical classes of fatty acids, flavonoids, and phenolic acids. Four bioactive compounds were identified in the hydroalcoholic extract: n-Hexadecanoic acid, Phytol, and 9,12,15-Octadecatrienoic acid. *In silico* PASS and ADMET predictions of *Z. oenopolia* leaf phytoconstituents indicated potential antioxidant, anti-inflammatory, cytotoxicity, and insecticidal activities, aligning with *in vitro* findings. The hydroalcoholic extract demonstrated significant antioxidant and anti-inflammatory properties through *in vitro* assays, including DPPH and ABTS radical scavenging, and inhibition of egg albumin and bovine serum albumin denaturation in a dose-dependent manner. The hydroalcoholic extract of *Z. oenopolia* leaves exhibited multifaceted bioactivity, significantly inhibiting *Saccharomyces cerevisiae* cell proliferation and demonstrating potent larvicidal effects against *Aedes aegypti* and *Tribolium castaneum* through interference with larval metamorphosis. Computational analyses further substantiated these results, revealing strong molecular interactions of the extract’s phytoconstituents with key biological targets, including human peroxiredoxin 5, COX-2, EGFR, and juvenile hormone binding protein. Collectively, these findings highlight the antioxidant, anti-inflammatory, cytotoxic, and larvicidal efficacy of *Z. oenopolia*, suggesting its potential to mitigate oxidative stress, prevent protein denaturation, inhibit fungal proliferation, and control mosquito larvae. The integrated in vitro and in silico outcomes position *Z. oenopolia* as a promising natural source of bioactive compounds with therapeutic relevance and vector control potential, meriting further exploration for biomedical and biocontrol applications.

## Supporting information

S1 Fig Plant authentication. (PDF)

S2 Data GC-MS Raw data for figures. (PDF)
