# Supplementary material for: Antioxidant, anti-inflammatory, cytotoxicity and larvicidal activities of Ziziphus oenopolia leaves: in vitro and in silico studies
Source: PLoS One. 2026 Oct 30;21(10):e0358577. doi: 10.1371/journal.pone.0358577 (PMC13722189; doi:10.1371/journal.pone.0358577)
Supplement: Supplementary file 1 [file pone.0358577.s001.pdf]

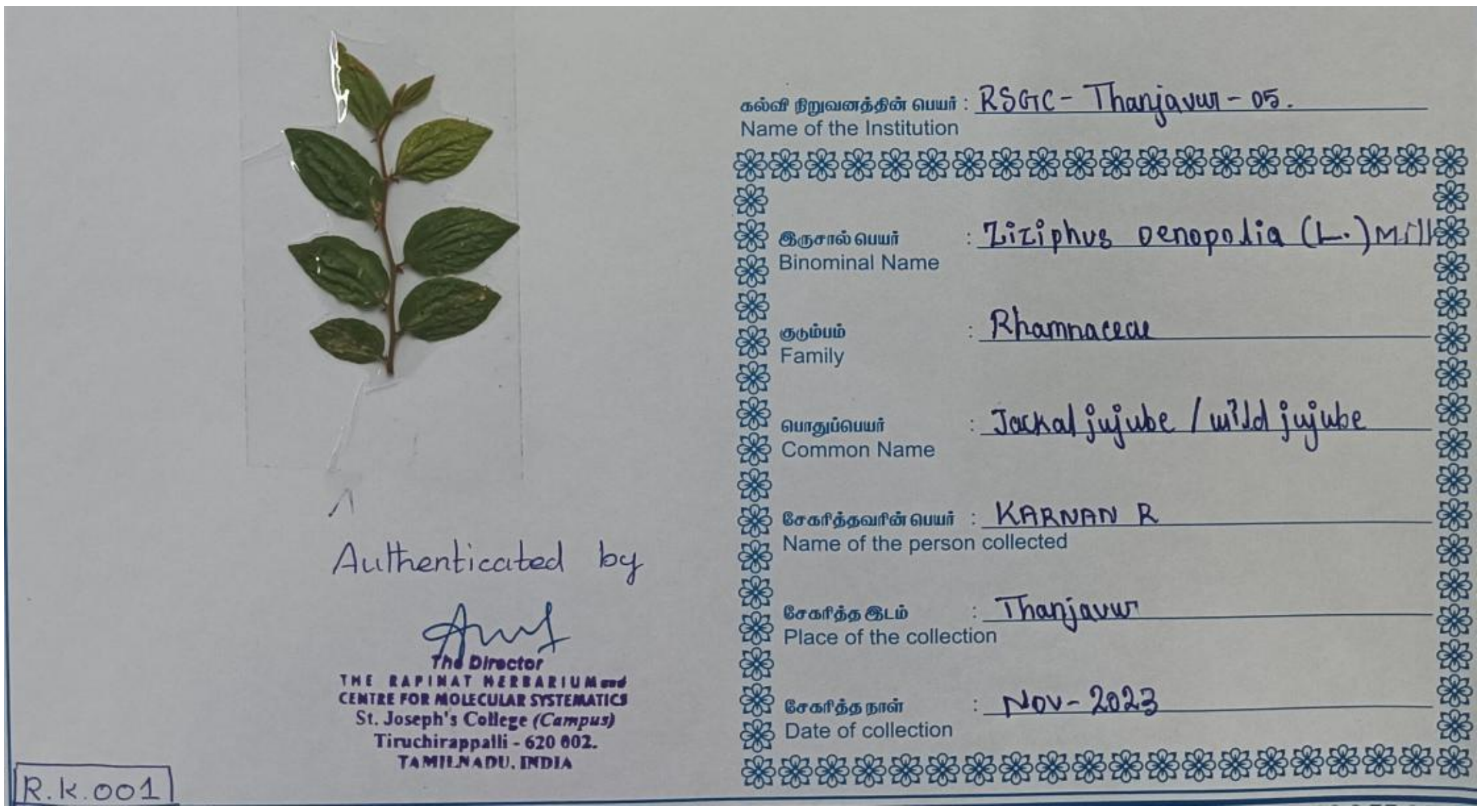

S1 Figure. Plant authentication
