# Supplementary material for: Antioxidant, anti-inflammatory, cytotoxicity and larvicidal activities of Ziziphus oenopolia leaves: in vitro and in silico studies
Source: PLoS One. 2026 Oct 30;21(10):e0358577. doi: 10.1371/journal.pone.0358577 (PMC13722189; doi:10.1371/journal.pone.0358577)
Supplement: Supplementary file 2 [file pone.0358577.s002.pdf]

Sample Information

Analyzed by	: Admin
Analyzed	: 23-09-2025 14:29:58
Sample Type	: Unknown
Sample Name	: K-ZI - 23.09.2025
Sample ID	: K-ZI - 23.09.2025
Injection Volume	: 2.00
Data File	: D:\K-ZI - 23.09.2025 .QGD
Method File	: D:\GCMS\Methode\23.09.2025.qgm
Tuning File	: D:\GCMS\Tuning\Tuning 14-05-25.qgt

Chromatogram K-ZI - 23.09.2025 D:\K-ZI - 23.09.2025 .QGD

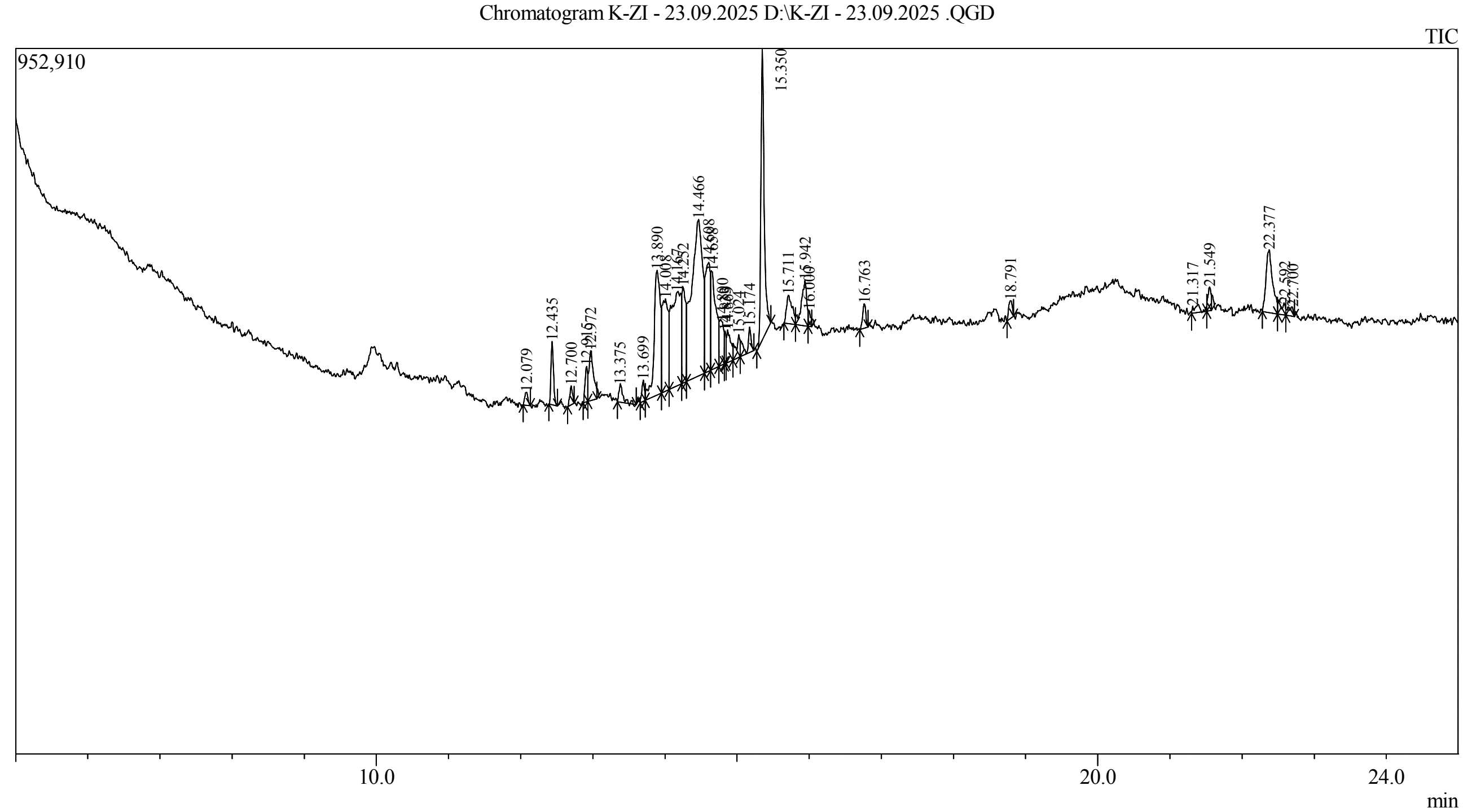

Peak Report TIC

Peak#	R.Time	Area	Area%	Height	Height%	A/H	Name
1	12.079	54082	0.47	17449	0.77	3.10	2-(2',4',4',6',6',8',8'-Heptamethyltetrasiloxan-2'
2	12.435	207189	1.79	85756	3.78	2.42	NEOPHYTADIENE
3	12.700	68795	0.59	25869	1.14	2.66	3,7,11,15-Tetramethyl-2-hexadecen-1-ol
4	12.915	123830	1.07	48120	2.12	2.57	2-(2',4',4',6',6',8',8'-Heptamethyltetrasiloxan-2'
5	12.972	293593	2.53	67388	2.97	4.36	1,2-Benzenedicarboxylic acid, bis(2-methylpro
6	13.375	91247	0.79	22476	0.99	4.06	Oxiraneundecanoic acid, 3-pentyl-, methyl este
7	13.699	69463	0.60	28669	1.26	2.42	Silane, [[4-[1,2-bis[(trimethylsilyl)oxy]ethyl]-
8	13.890	1031292	8.89	168073	7.41	6.14	n-Hexadecanoic acid
9	14.008	754320	6.50	124466	5.49	6.06	Di-n-octyl phthalate
10	14.167	1231525	10.61	126203	5.56	9.76	Heptane, 2,2,3,3,5,6,6-heptamethyl-
11	14.252	470778	4.06	130023	5.73	3.62	d-Glucitol, 4-O-decyl-
12	14.466	2275754	19.61	211274	9.31	10.77	2-methylhexacosane
13	14.608	698758	6.02	146636	6.46	4.77	Docosane (CAS) n-Docosane
14	14.658	658929	5.68	131968	5.82	4.99	TETRAPENTACONTAN, 1,54-DIBROMO-
15	14.800	250765	2.16	58045	2.56	4.32	Octanoic acid, 2-methylphenyl ester
16	14.833	58636	0.51	44082	1.94	1.33	2(3H)-Furanone, 5-hexyldihydro-
17	14.869	169085	1.46	44120	1.94	3.83	Eicosanoic acid, 2-hydroxyethyl ester
18	15.024	118752	1.02	30765	1.36	3.86	3-Pyridinecarboxaldehyde, O-acetyloxime, (E)
19	15.174	141346	1.22	34324	1.51	4.12	Benzoic acid, 2,4-bis[(trimethylsilyl)oxy]-, trir
20	15.350	1235896	10.65	393495	17.34	3.14	Phytol
21	15.711	209687	1.81	38057	1.68	5.51	9-Octadecenal, (Z)-
22	15.942	314602	2.71	60900	2.68	5.17	9,12,15-Octadecatrienal
23	16.000	46364	0.40	21571	0.95	2.15	2,2-dimethyl-4-phenylaminobutanone
24	16.763	101202	0.87	32968	1.45	3.07	1,1,1,5,7,7,7-Heptamethyl-3,3-bis(trimethylsil
25	18.791	83851	0.72	24068	1.06	3.48	Octasiloxane, 1,1,3,3,5,5,7,7,9,9,11,11,13,13,
26	21.317	61870	0.53	9325	0.41	6.63	Bidrin
27	21.549	95763	0.83	31716	1.40	3.02	SILIKONFETT SE30 (GREVELS)

Peak#	R.Time	Area	Area%	Height	Height%	A/H	Name
28	22.377	541310	4.67	84966	3.74	6.37	Di-n-octyl phthalate
29	22.592	84950	0.73	14165	0.62	6.00	1,3-Butadiene, 1,1-bis(methylthio)-
30	22.700	59169	0.51	11919	0.53	4.96	ETHYL 1-HEXYL-4-HYDROXY-2(1H)-OX(
		11602803	100.00	2268856	100.00		

Library

<< Target >>
Line#:1 R.Time:12.075(Scan#:850) MassPeaks:35
RawMode:Averaged 12.067-12.083(849-851) BasePeak:73.10(6833)
BG Mode:Calc. from Peak Group 1 - Event 1 Scan

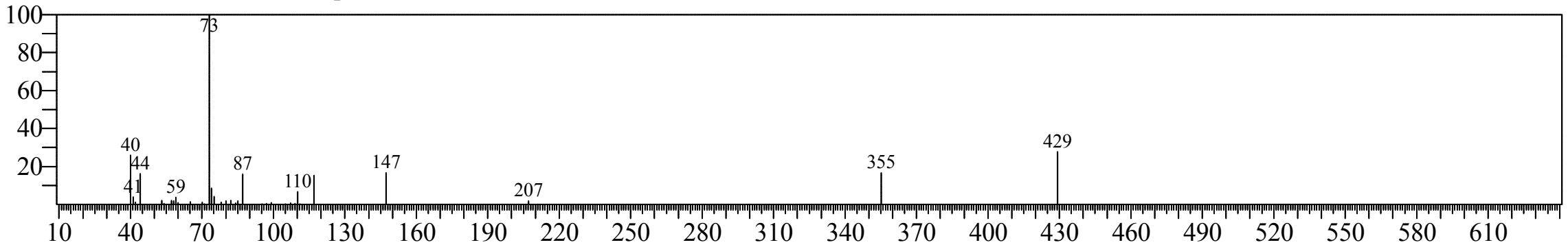

Hit#:1 Entry:161854 Library:NIST05.LIB
SI:69 Formula:C16H48O10Si9 CAS:145344-72-5 MolWeight:652 RetIndex:1716
CompName:2-(2',4',4',6',6',8',8'-Heptamethyltetrasiloxan-2'-yloxy)-2,4,4,6,6,8,8,10,10-nonamethylcyclopentasiloxane $$ 2-[(2,4,4,6,6,8,8-Heptamethyl-1,3,5

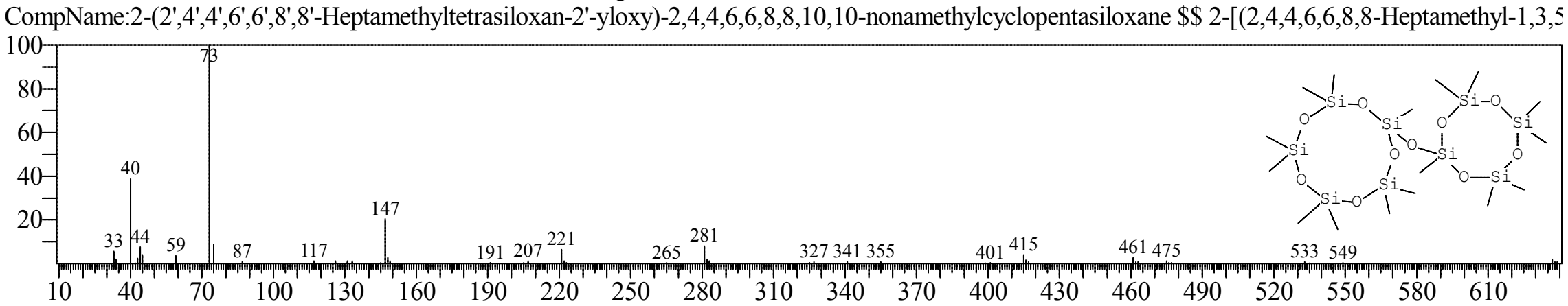

Hit#:2 Entry:306966 Library:WILEY7.LIB
SI:69 Formula:C20 H42 O4 SI4 CAS:56114-62-6 MolWeight:458 RetIndex:0
CompName:Silane, [[4-[1,2-bis[(trimethylsilyl)oxy]ethyl]-1,2-phenylene]bis(oxy)]bis[trimethyl- (CAS) 3,4-DIHYDROXYPHENYLGLYCOL-TETRATMS

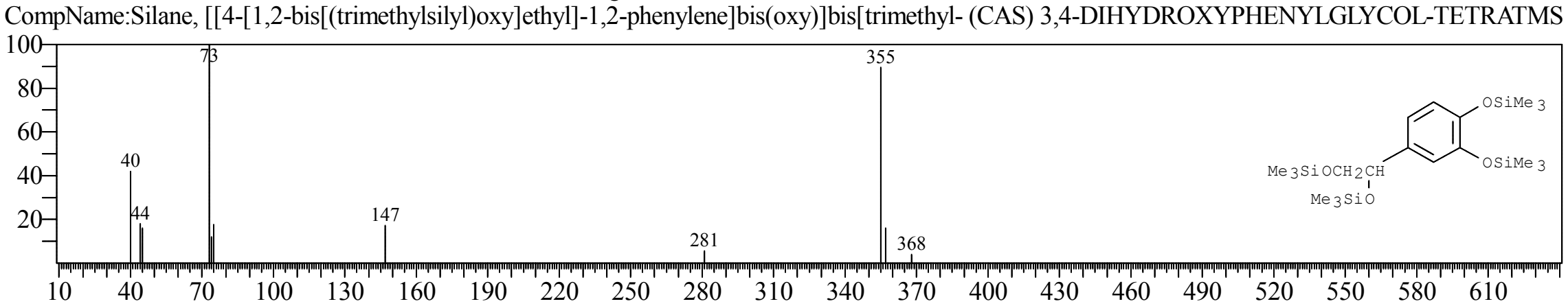

Hit#:3 Entry:32238 Library:NIST05.LIB
SI:66 Formula:C11H24Si CAS:252991-06-3 MolWeight:184 RetIndex:1015
CompName:Silane, trimethyl-7-octenyl- $$ Trimethyl(7-octenyl)silane # $$

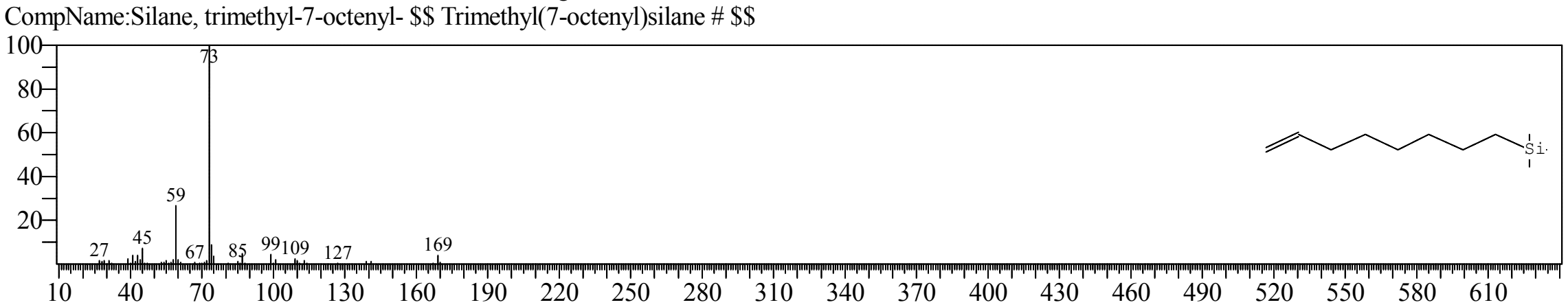

Hit#:4 Entry:76222 Library:WILEY7.LIB
SI:66 Formula:C11 H24 SI CAS:0-00-0 MolWeight:184 RetIndex:0
CompName:SILANE, TRIMETHYL-7-OCTENYL- $$

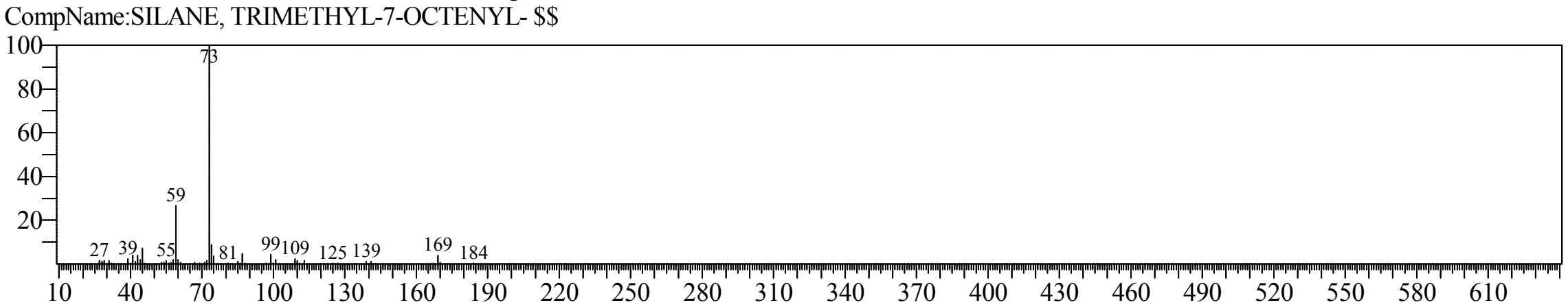

<< Target >>
Line#:1   R.Time:12.075(Scan#:850) MassPeaks:35
RawMode:Averaged 12.067-12.083(849-851) BasePeak:73.10(6833)
BG Mode:Calc. from Peak Group 1 - Event 1 Scan

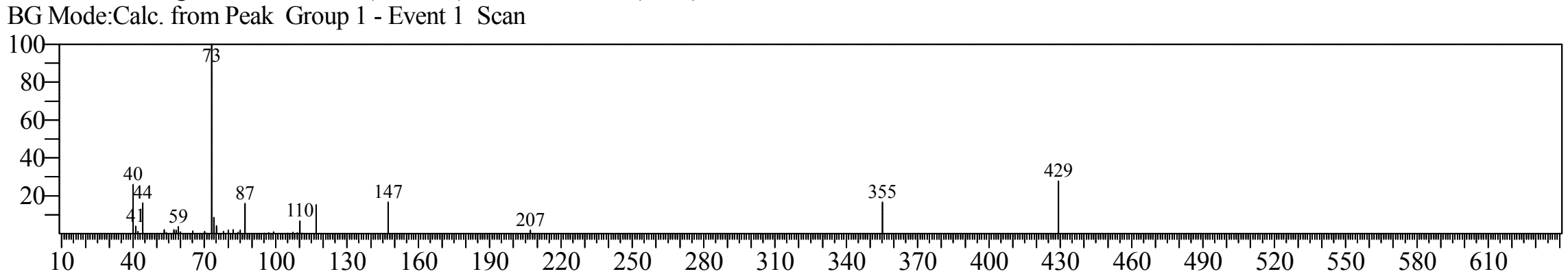

Hit#:5 Entry:8329 Library:NIST05.LIB
SI:66 Formula:C7H16O2 CAS:74421-00-4 MolWeight:132 RetIndex:720
CompName:Butane, 2,3-dimethoxy-2-methyl- $$ 2,3-Dimethoxy-2-methylbutane # $$

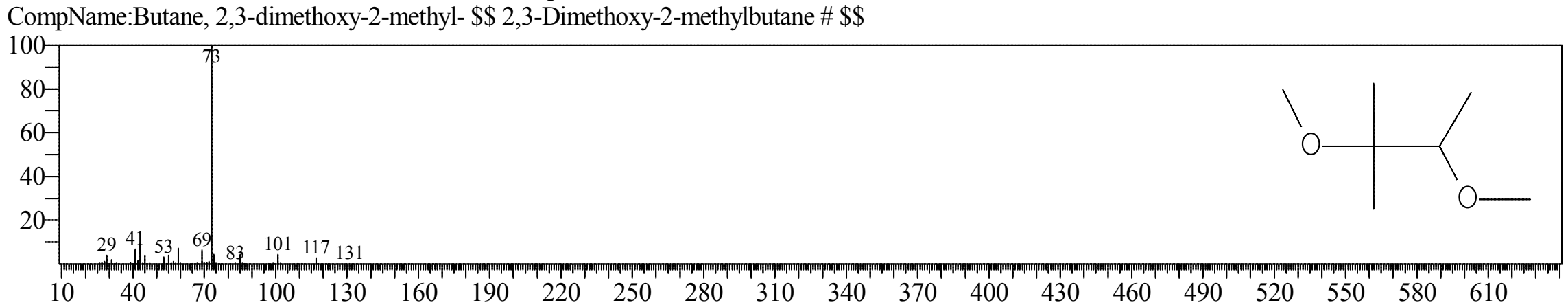

Hit#:6 Entry:7605 Library:WILEY7.LIB
SI:65 Formula:C5 H14 SI CAS:3439-38-1 MolWeight:102 RetIndex:0
CompName:Silane, ethyltrimethyl- (CAS) Ethyltrimethylsilane $$ Trimethylethylsilane $$

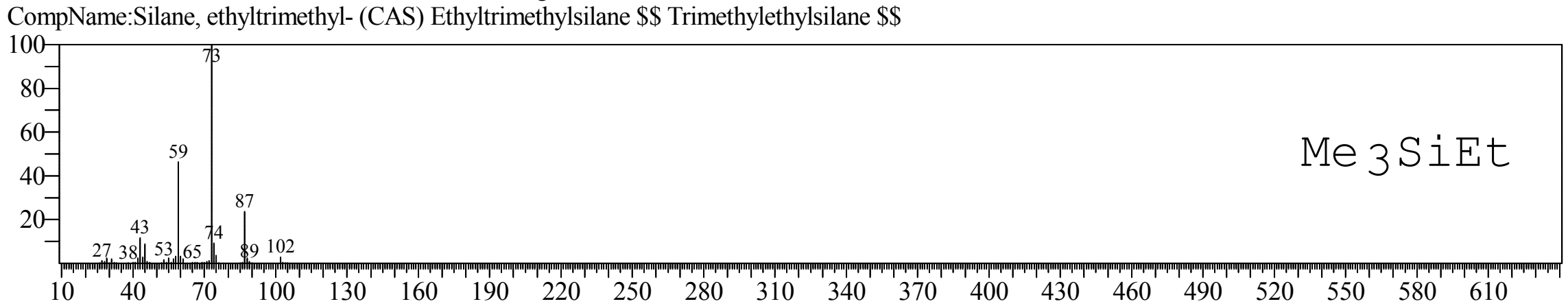

Hit#:7 Entry:27089 Library:NIST11.lib
SI:65 Formula:C10H20O2 CAS:4359-57-3 MolWeight:172 RetIndex:1235
CompName:1,3-Dioxolane, 2-heptyl- $$ 2-Heptyl-1,3-dioxolane # $$

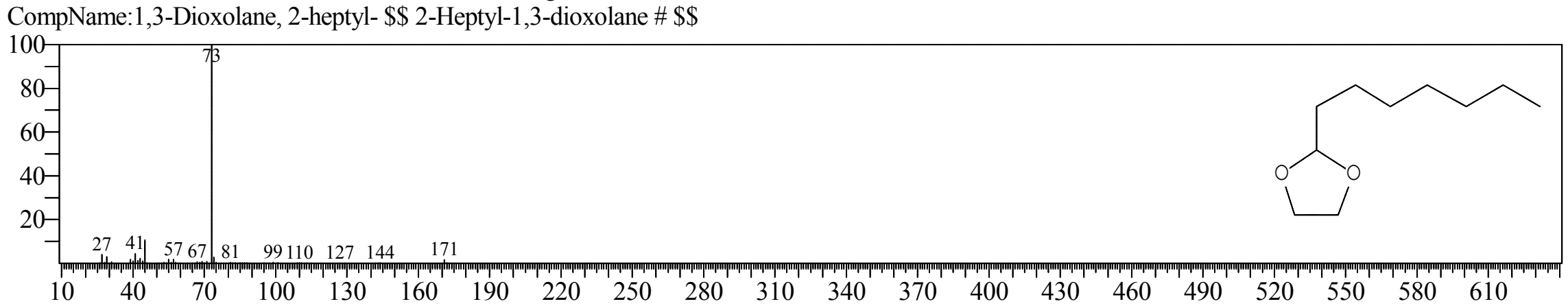

Hit#:8 Entry:313972 Library:WILEY7.LIB
SI:65 Formula:C24 H50 O4 SI3 CAS:129745-62-6 MolWeight:486 RetIndex:0
CompName:(-)-(4R,5S,6R)-4,5,6-Tris-{[(tert-butyl)dimethylsilyl]oxy}cyclohex-2-en-1-one $$

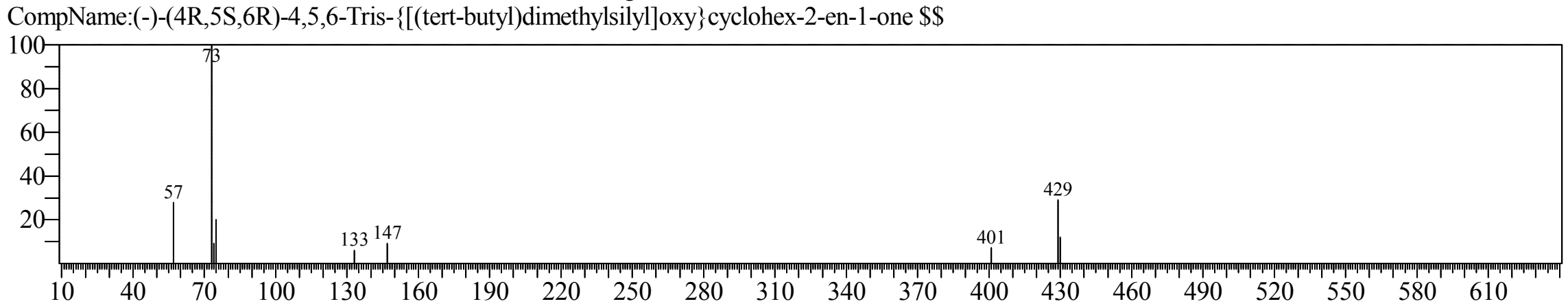

Hit#:9 Entry:48310 Library:WILEY7.LIB
SI:65 Formula:C9 H21 N O CAS:0-00-0 MolWeight:159 RetIndex:0
CompName:N,N-DIMETHYL-2-BUTOXYISOPROPYLAMINE $$

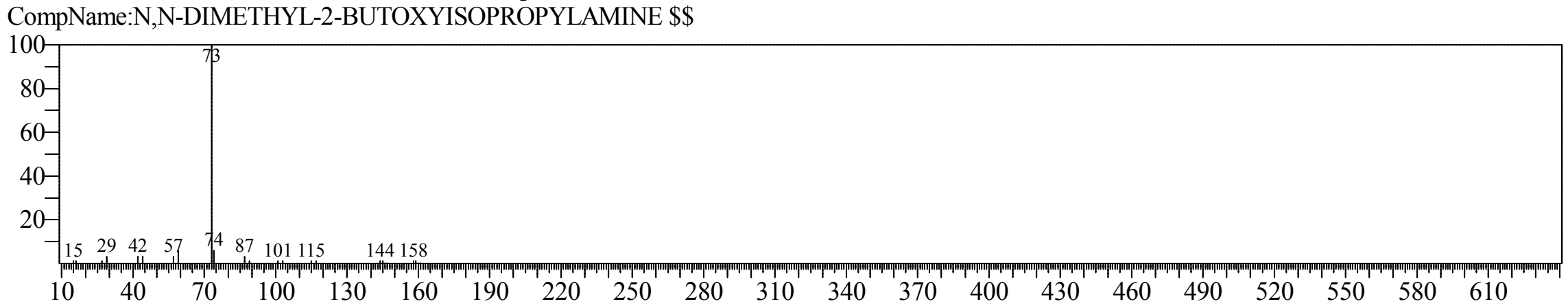

<< Target >>
Line#:1   R.Time:12.075(Scan#:850) MassPeaks:35
RawMode:Averaged 12.067-12.083(849-851) BasePeak:73.10(6833)
BG Mode:Calc. from Peak Group 1 - Event 1 Scan

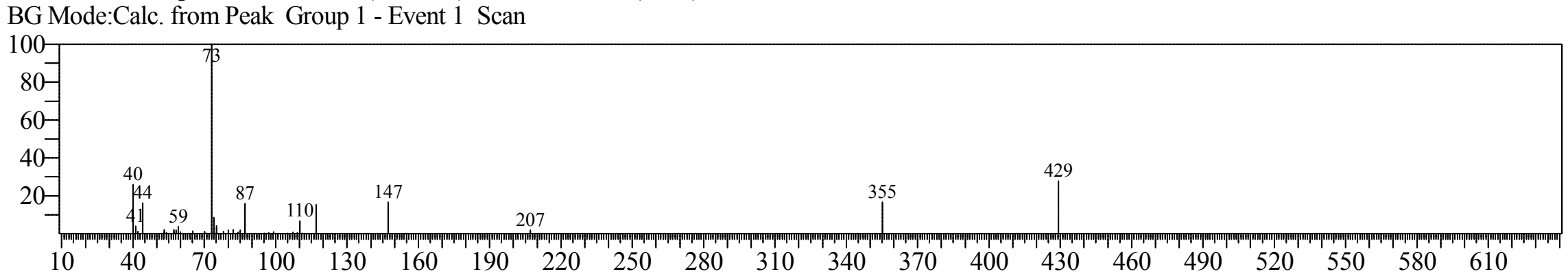

Hit#:10 Entry:12194 Library:NIST11.lib
SI:65 Formula:C8H18Si CAS:53264-56-5 MolWeight:142 RetIndex:670
CompName:Silane, trimethyl-3-penten-2-yl-, trans $$ Trimethyl[(2E)-1-methyl-2-butenyl]silane # $$

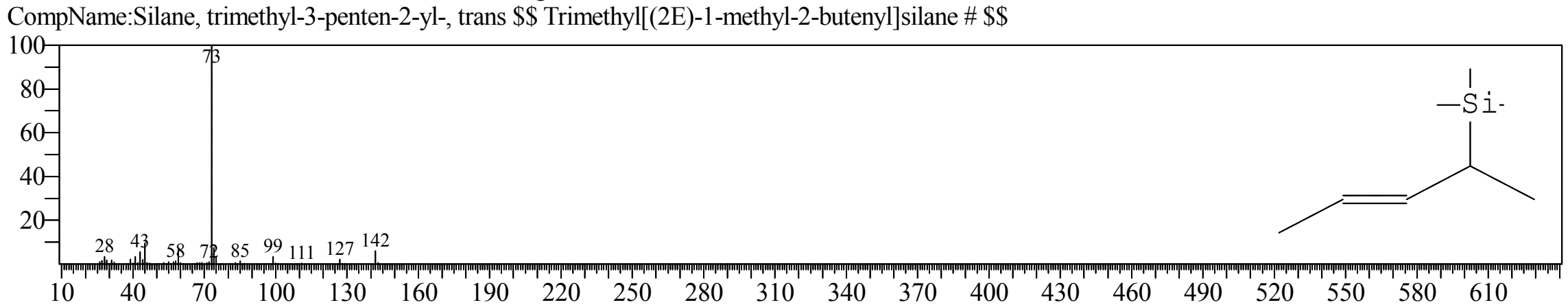

Hit#:11 Entry:31091 Library:WILEY7.LIB
SI:65 Formula:C8 H18 SI CAS:0-00-0 MolWeight:142 RetIndex:0
CompName:SILANE, TRIMETHYL-3-PENTEN-2-YL-, TRANS $$

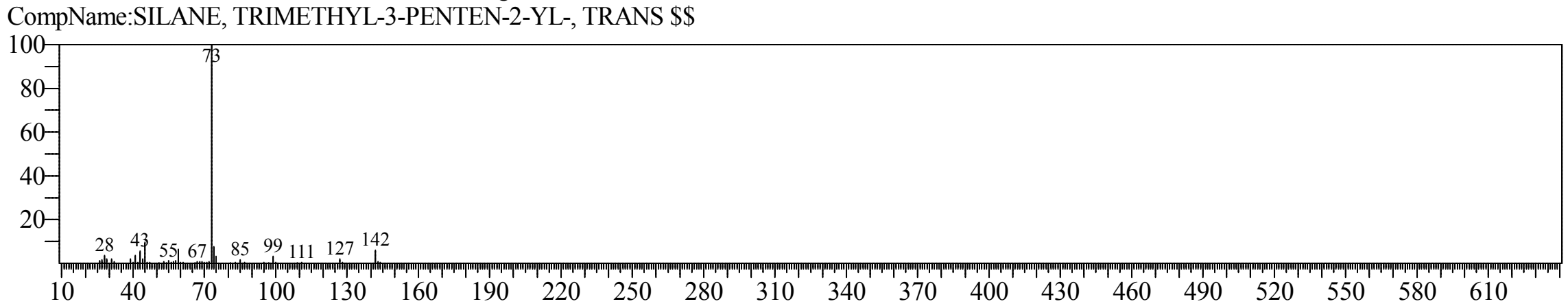

Hit#:12 Entry:53342 Library:NIST11.lib
SI:65 Formula:C11H22O2Si CAS:0-00-0 MolWeight:214 RetIndex:1200
CompName:3-Octenoic acid, trimethylsilyl ester $$ Trimethylsilyl (3E)-3-octenoate # $$

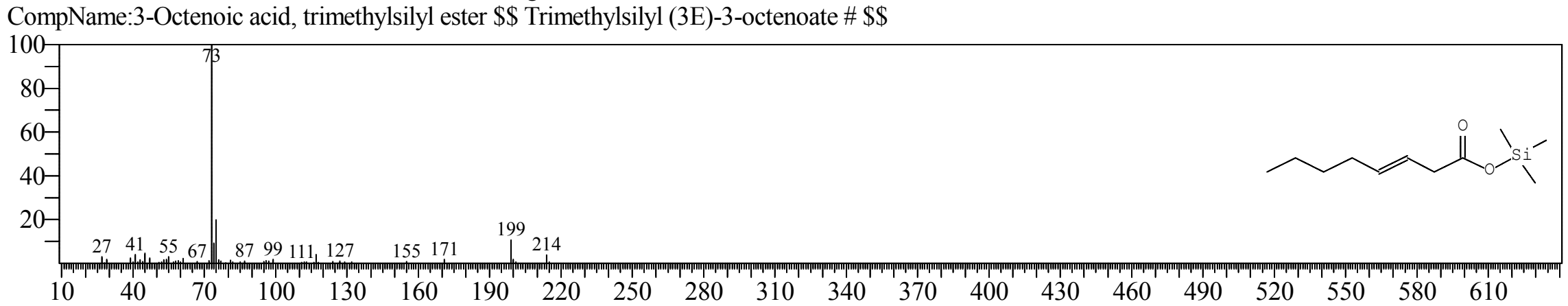

Hit#:13 Entry:49335 Library:NIST05.LIB
SI:65 Formula:C11H22O2Si CAS:0-00-0 MolWeight:214 RetIndex:1200
CompName:3-Octenoic acid, trimethylsilyl ester

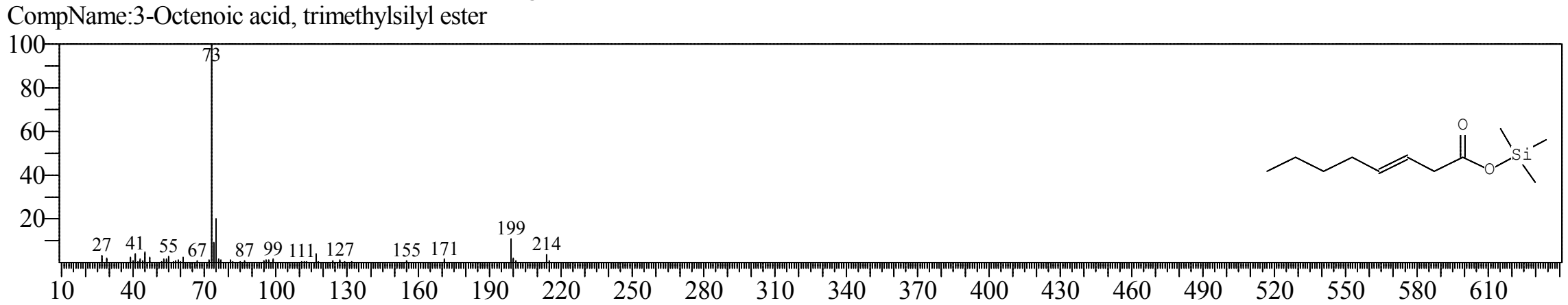

Hit#:14 Entry:112587 Library:WILEY7.LIB
SI:64 Formula:C11 H22 O2 SI CAS:0-00-0 MolWeight:214 RetIndex:0
CompName:3-OCTENEOIC ACID 1TMS $$

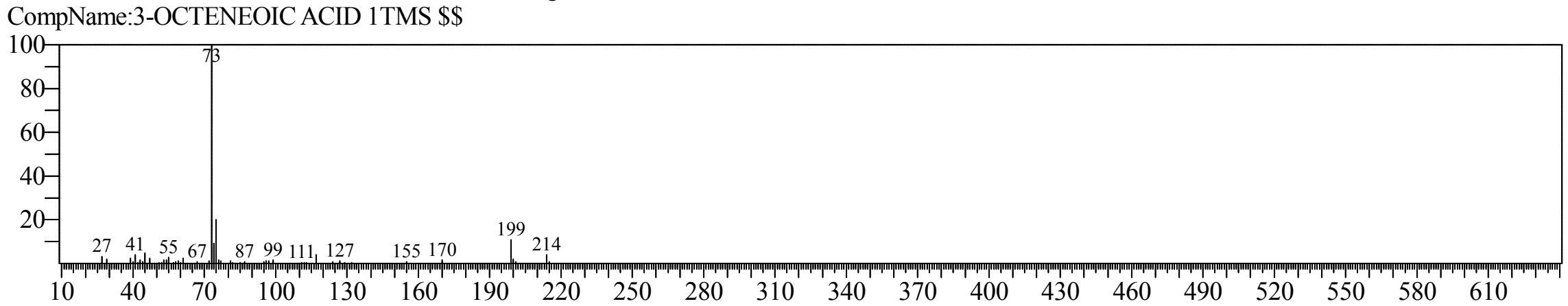

<< Target >>
Line#:1   R.Time:12.075(Scan#:850) MassPeaks:35
RawMode:Averaged 12.067-12.083(849-851) BasePeak:73.10(6833)
BG Mode:Calc. from Peak Group 1 - Event 1 Scan

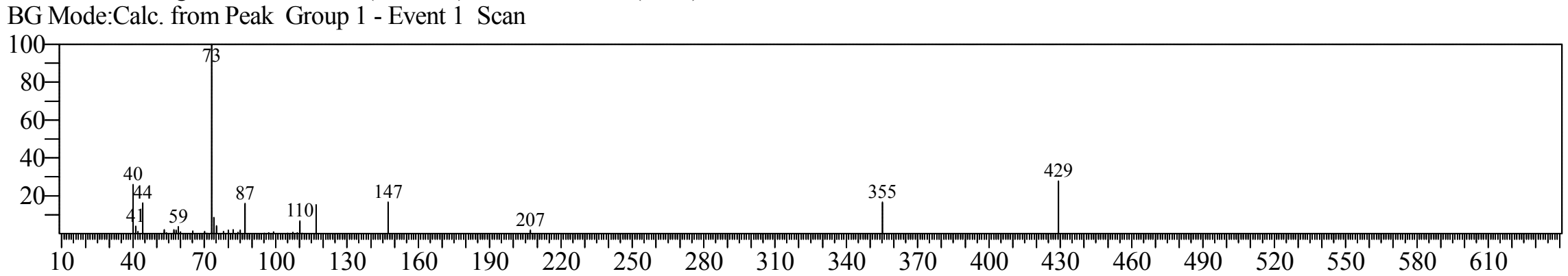

Hit#:15 Entry:21128 Library:NIST11.lib
SI:64 Formula:C8H18O3 CAS:0-00-0 MolWeight:162 RetIndex:960
CompName:1-(2-Methoxyethoxy)-2-methyl-2-propanol, methyl ether

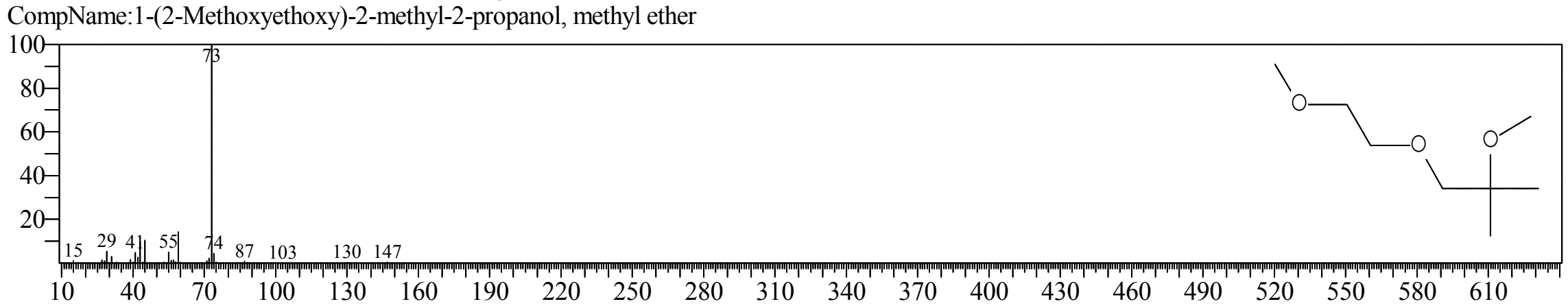

Hit#:16 Entry:45888 Library:WILEY7.LIB
SI:64 Formula:C8 H8 D4 O3 CAS:60378-35-0 MolWeight:156 RetIndex:0
CompName:3,3,6,6-TERTADEUTERIO-1,5-DIOXA-2-CIS-DECALONE $$ Pyrano[3,2-b]pyran-2(3H)-one-3,3,6-d3, hexahydro-6-d-, cis- (CAS)

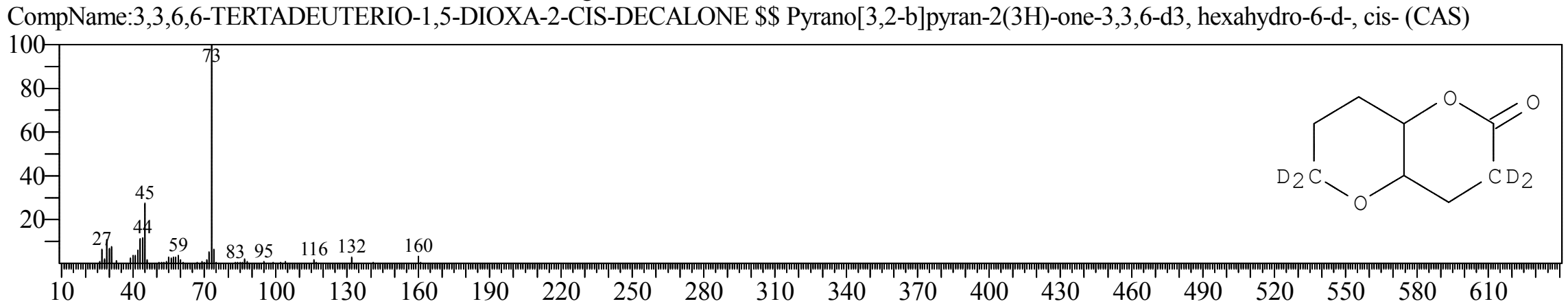

Hit#:17 Entry:102985 Library:NIST05.LIB
SI:64 Formula:C13H28O4Si2 CAS:90744-45-9 MolWeight:304 RetIndex:1468
CompName:Malonic acid, bis(2-trimethylsilylethyl ester $$ Bis[2-(trimethylsilyl)ethyl] malonate # $$

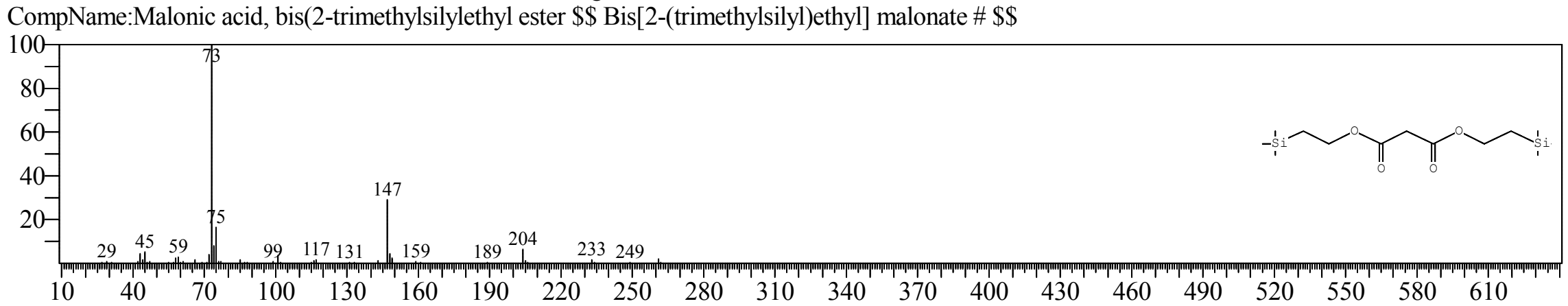

Hit#:18 Entry:7265 Library:NIST11.lib
SI:64 Formula:C7H16Si CAS:10111-13-4 MolWeight:128 RetIndex:612
CompName:Silane, trimethyl(1-methyl-1-propenyl)-, (E)- $$ Trimethyl[(1E)-1-methyl-1-propenyl]silane # $$

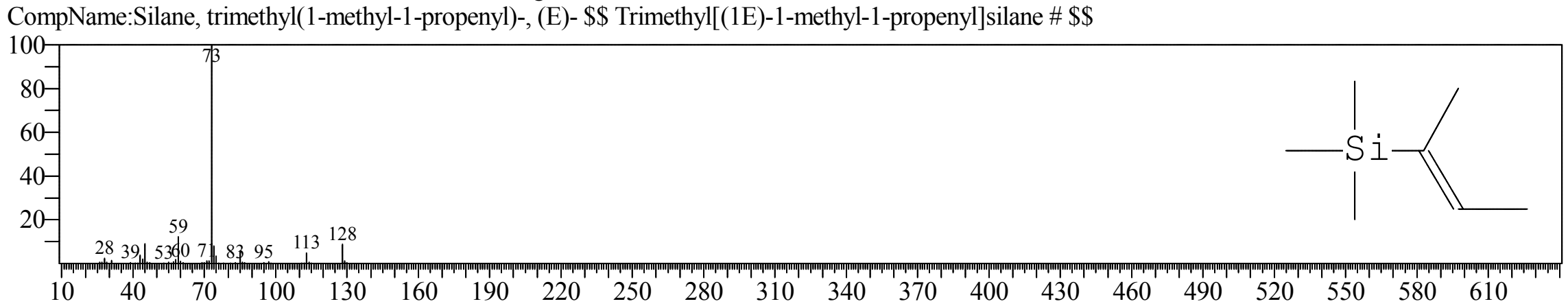

Hit#:19 Entry:310785 Library:WILEY7.LIB
SI:63 Formula:C20 H40 O5 SI4 CAS:37148-65-5 MolWeight:472 RetIndex:0
CompName:Benzeneacetic acid, .alpha.,3,4-tris[(trimethylsilyl)oxy]-, trimethylsilyl ester (CAS) TETRAKISTRIMETHYLSILYL 3,4-DIHYDROXYMAND

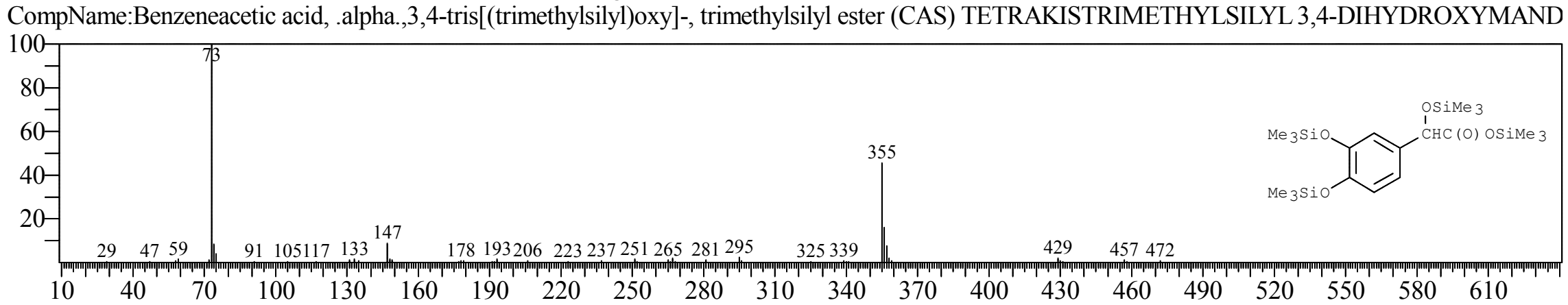

<< Target >>
Line#:1   R.Time:12.075(Scan#:850) MassPeaks:35
RawMode:Averaged 12.067-12.083(849-851) BasePeak:73.10(6833)
BG Mode:Calc. from Peak Group 1 - Event 1 Scan

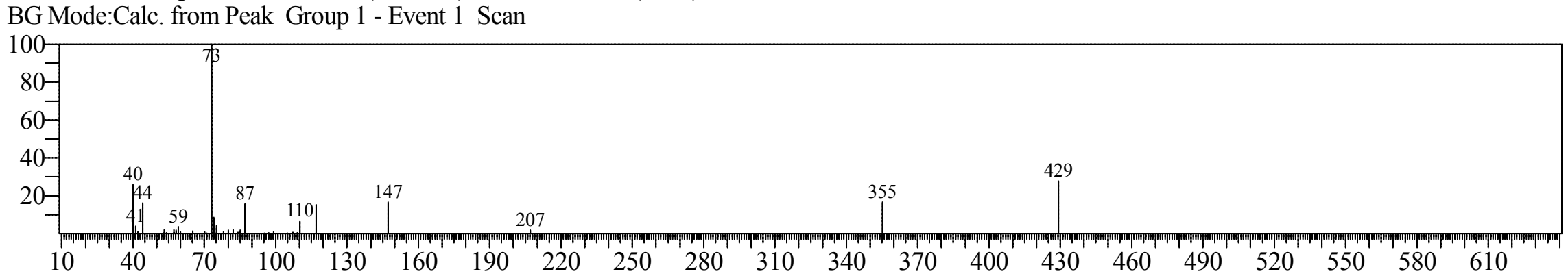

Hit#:20 Entry:92718 Library:NIST05.LIB
SI:63 Formula:C14H34Si3 CAS:136935-44-9 MolWeight:286 RetIndex:1087
CompName:1,3,5-Tris(trimethylsilyl)pent-1-ene $$ [(1Z)-3,5-Bis(trimethylsilyl)-1-pentenyl](trimethyl)silane # $$

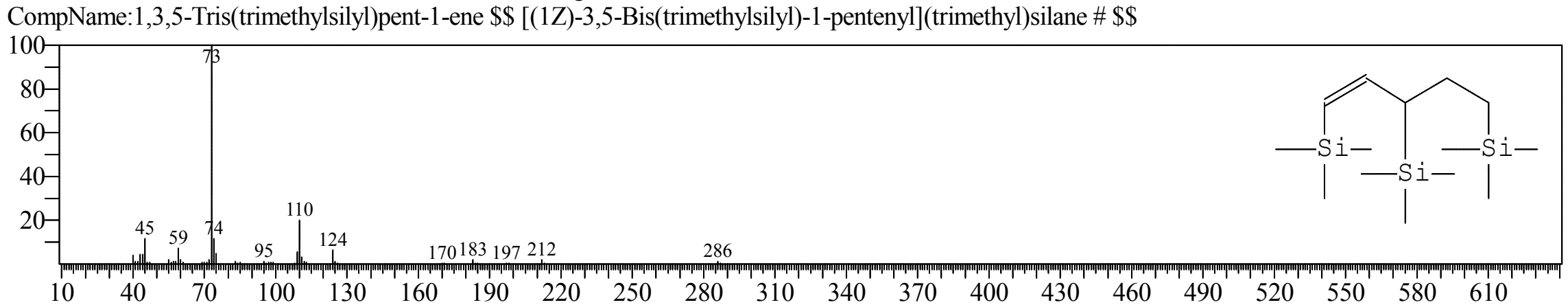

<< Target >>
Line#:2   R.Time:12.433(Scan#:893) MassPeaks:48
RawMode:Averaged 12.425-12.442(892-894) BasePeak:68.10(6749)
BG Mode:Calc. from Peak Group 1 - Event 1 Scan

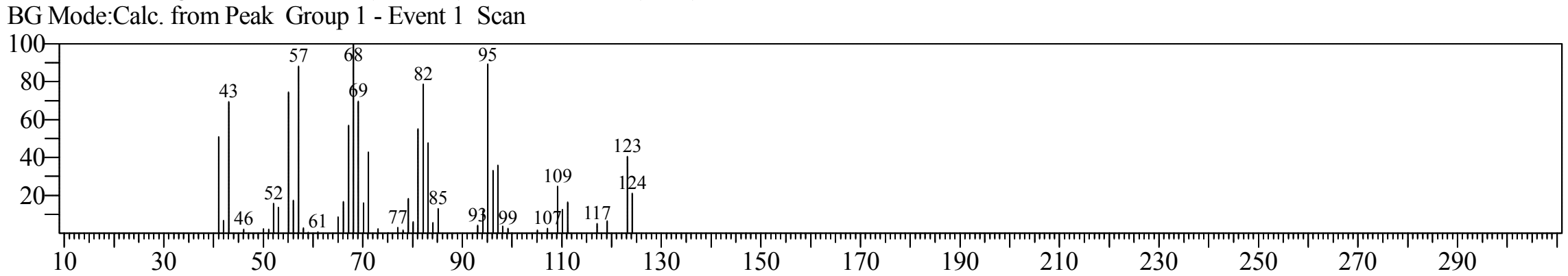

Hit#:1 Entry:189040 Library:WILEY7.LIB
SI:91 Formula:C20 H38 CAS:0-00-0 MolWeight:278 RetIndex:0
CompName:NEOPHYTADIENE $$ 2,6,10-TRIMETHYL,14-ETHYLENE-14-PENTADECNE $$

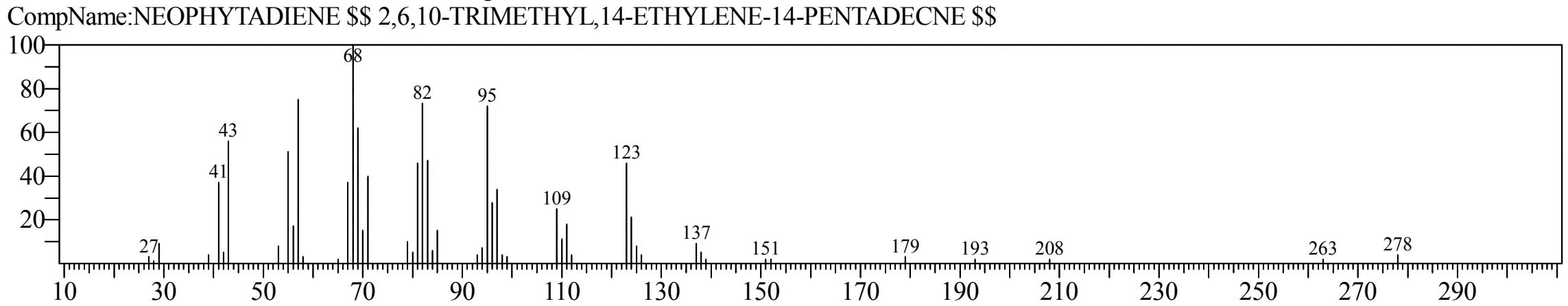

Hit#:2 Entry:207900 Library:WILEY7.LIB
SI:89 Formula:C20 H40 O CAS:150-86-7 MolWeight:296 RetIndex:0
CompName:2-Hexadecen-1-ol, 3,7,11,15-tetramethyl-, [R-[R*,R*-(E)]]- (CAS) Phytol $$ trans-Phytol $$ (E)-(7R,11R)-3,7,11,15-tetramethyl-2-hexadecen-

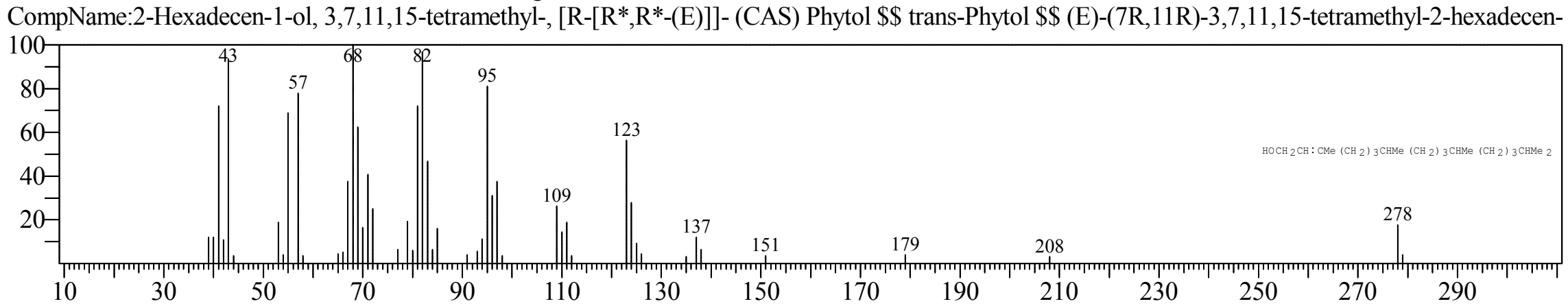

Hit#:3 Entry:98880 Library:NIST05.LIB
SI:88 Formula:C20H40O CAS:102608-53-7 MolWeight:296 RetIndex:2045
CompName:3,7,11,15-Tetramethyl-2-hexadecen-1-ol $$ (2E)-3,7,11,15-Tetramethyl-2-hexadecen-1-ol # $$

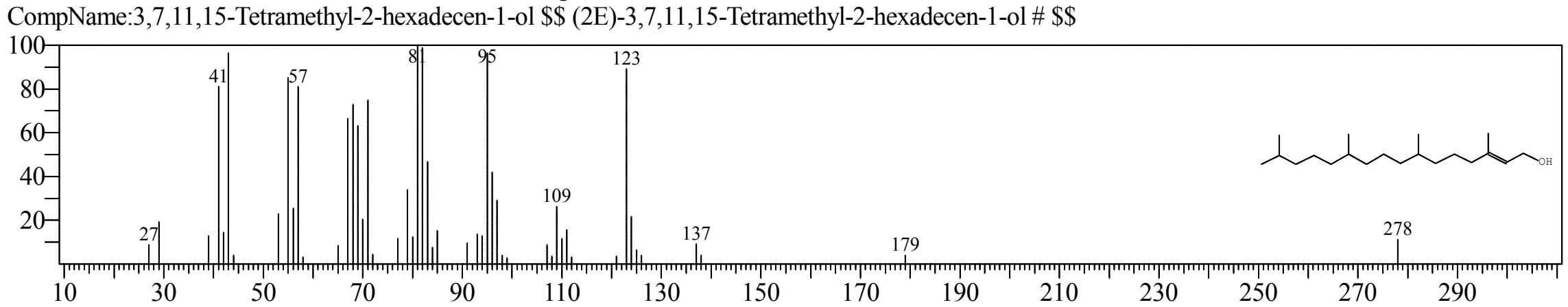

Hit#:4 Entry:189041 Library:WILEY7.LIB
SI:87 Formula:C20 H38 CAS:0-00-0 MolWeight:278 RetIndex:0
CompName:NEOPHYTADIENE $$ 2,6,10-TRIMETHYL,14-ETHYLENE-14-PENTADECNE $$

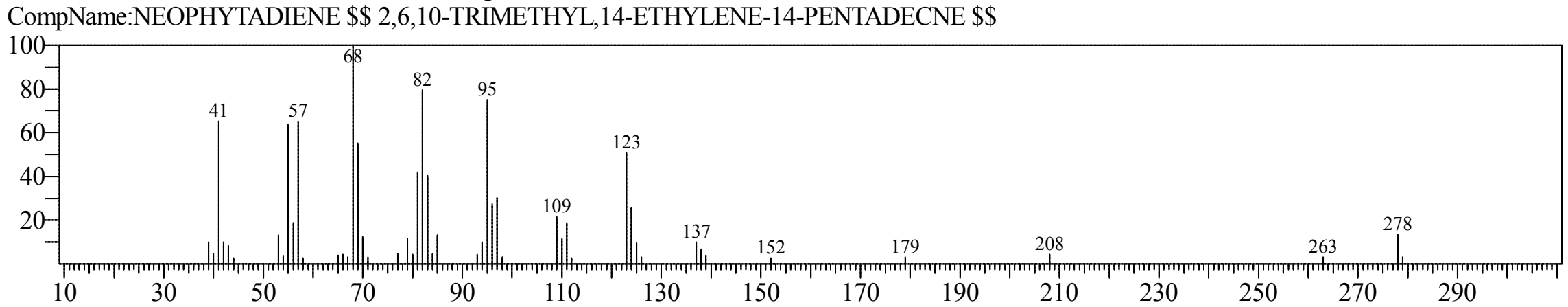

Hit#:5 Entry:147507 Library:NIST11.lib
SI:87 Formula:C22H42O2 CAS:0-00-0 MolWeight:338 RetIndex:2168
CompName:Phytol, acetate

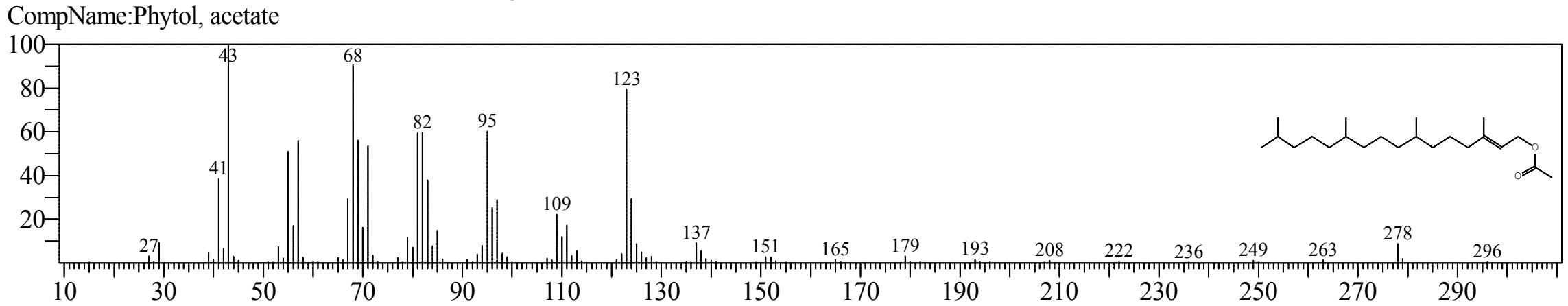

<< Target >>
Line#:2 R.Time:12.433(Scan#:893) MassPeaks:48
RawMode:Averaged 12.425-12.442(892-894) BasePeak:68.10(6749)
BG Mode:Calc. from Peak Group 1 - Event 1 Scan

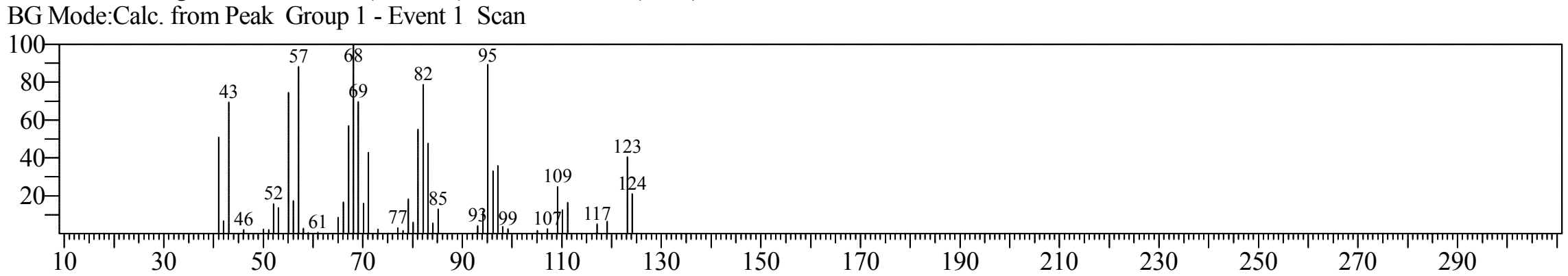

Hit#:6 Entry:20938 Library:NIST05s.LIB
SI:86 Formula:C18H34 CAS:629-89-0 MolWeight:250 RetIndex:1808
CompName:1-Octadecyne

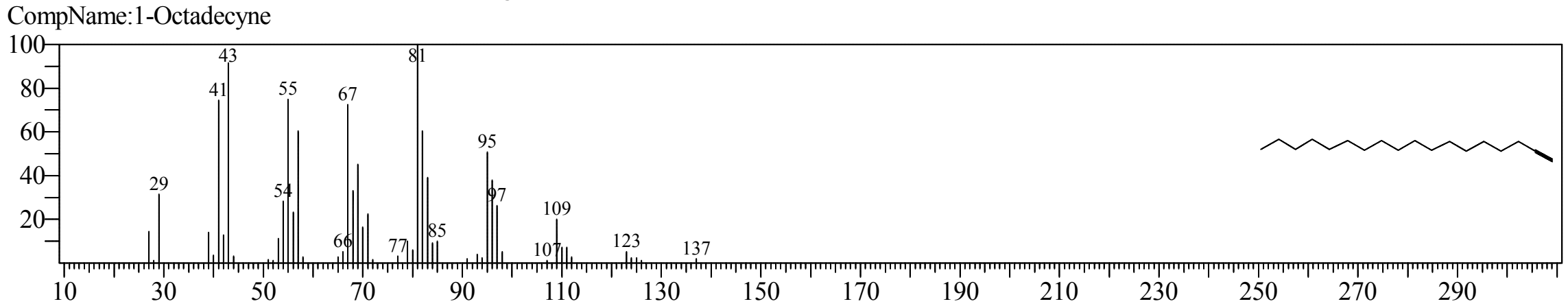

Hit#:7 Entry:88250 Library:NIST05.LIB
SI:86 Formula:C20H38 CAS:71899-38-2 MolWeight:278 RetIndex:2027
CompName:9-Eicosyne $$ 9-Icosyne # $$

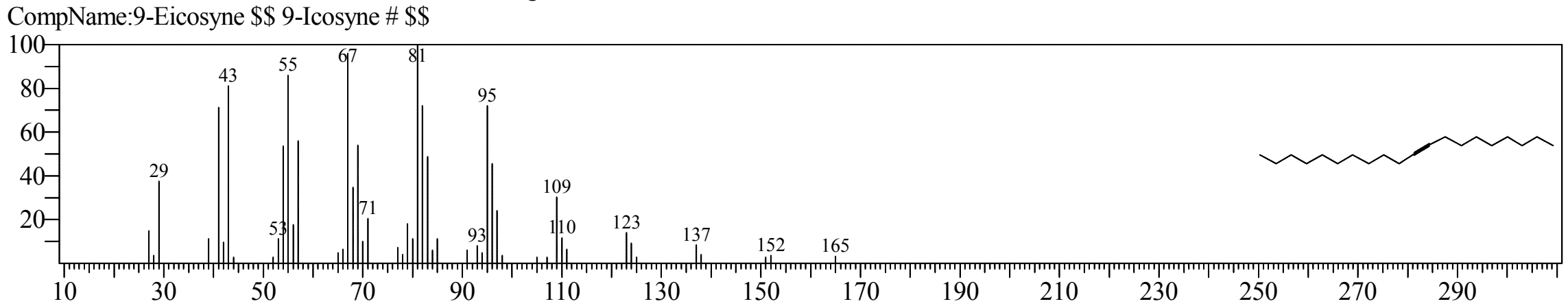

Hit#:8 Entry:20218 Library:NIST05s.LIB
SI:86 Formula:C16H32O CAS:7320-37-8 MolWeight:240 RetIndex:1702
CompName:Oxirane, tetradecyl- $$ Hexadecane, 1,2-epoxy- $$ Hexadecylene oxide $$ 1,2-Epoxyhexadecane $$ 1,2-Hexadecane oxide $$ 1,2-Hexadecene

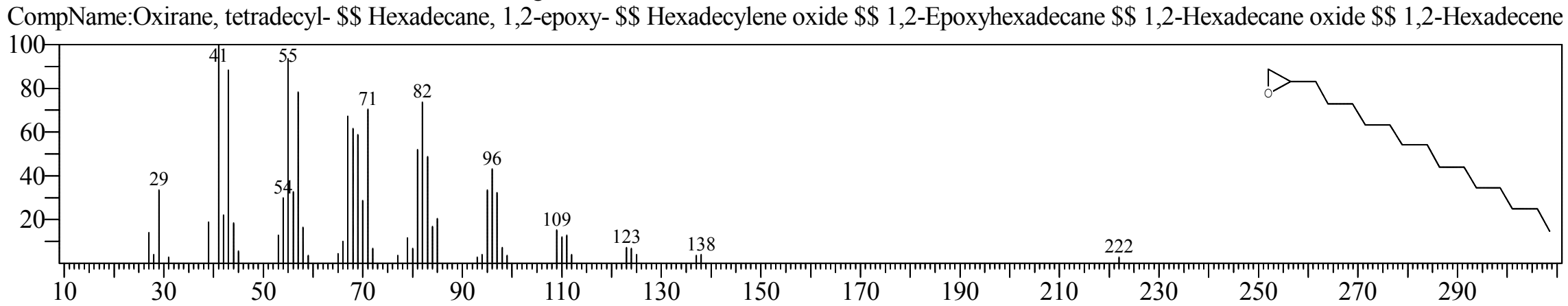

Hit#:9 Entry:57144 Library:NIST05.LIB
SI:86 Formula:C15H30O CAS:2765-11-9 MolWeight:226 RetIndex:1701
CompName:Pentadecanal-

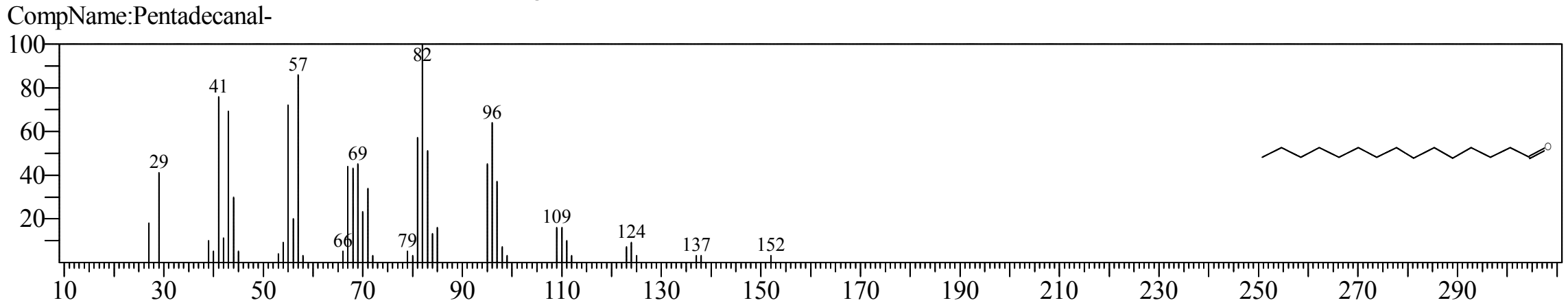

Hit#:10 Entry:55804 Library:WILEY7.LIB
SI:86 Formula:C12 H22 CAS:0-00-0 MolWeight:166 RetIndex:0
CompName:Cyclopentene, 3-heptyl- $$

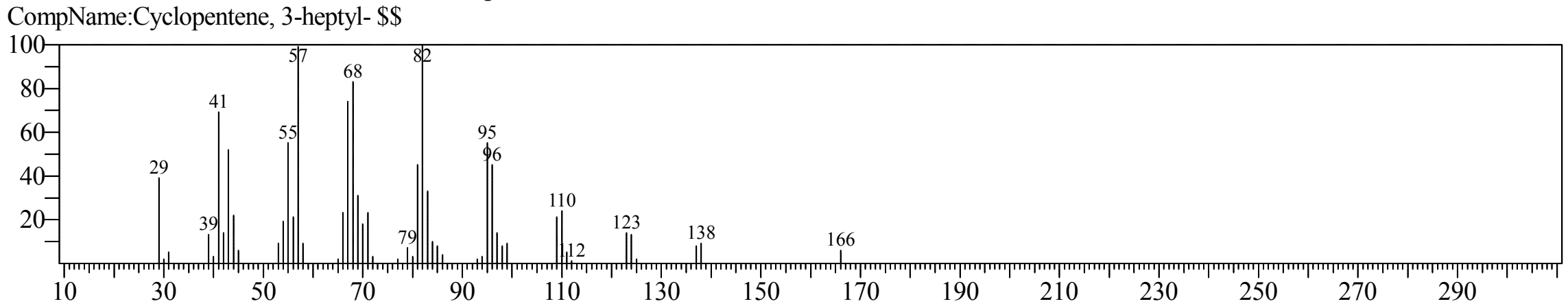

<< Target >>
Line#:2 R.Time:12.433(Scan#:893) MassPeaks:48
RawMode:Averaged 12.425-12.442(892-894) BasePeak:68.10(6749)
BG Mode:Calc. from Peak Group 1 - Event 1 Scan

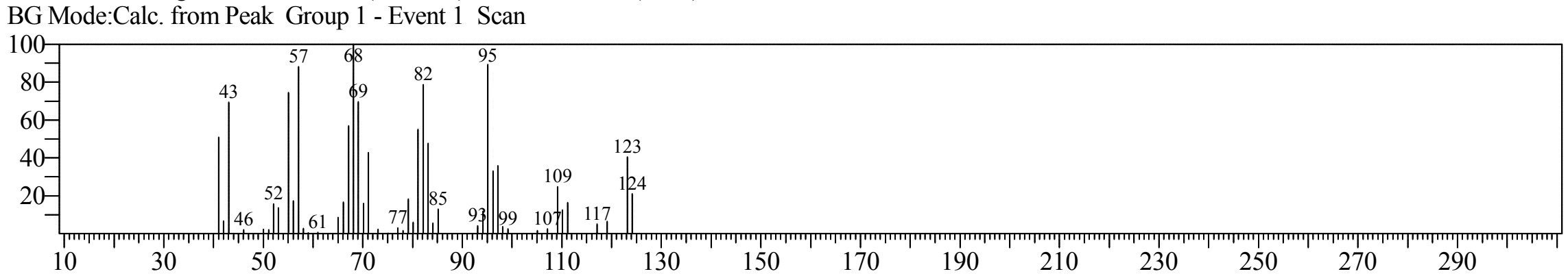

Hit#:11 Entry:73796 Library:NIST05.LIB
SI:85 Formula:C17H34O CAS:30689-78-2 MolWeight:254 RetIndex:1898
CompName:(R)-(-)-(Z)-14-Methyl-8-hexadecen-1-ol $$ (8Z)-14-Methyl-8-hexadecen-1-ol # $$

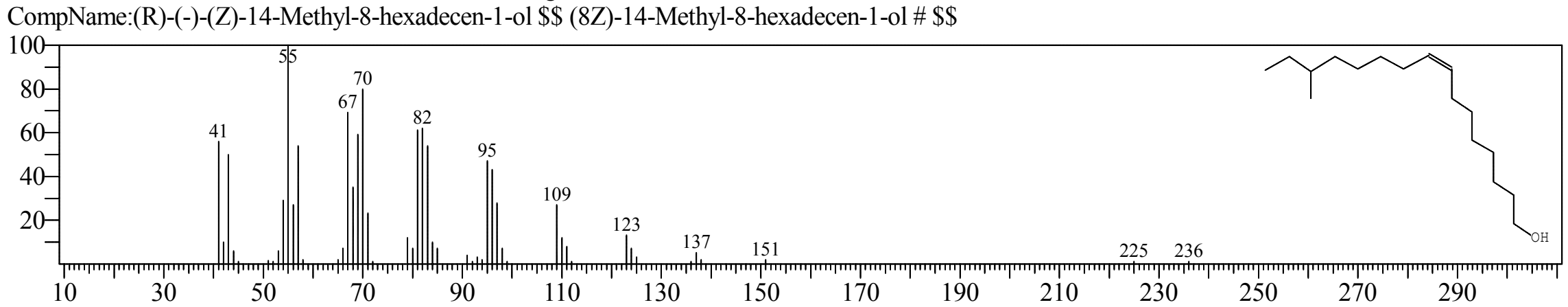

Hit#:12 Entry:91119 Library:WILEY7.LIB
SI:85 Formula:C12 H20 O2 CAS:25905-14-0 MolWeight:196 RetIndex:0
CompName:lavandulyl acetate $$

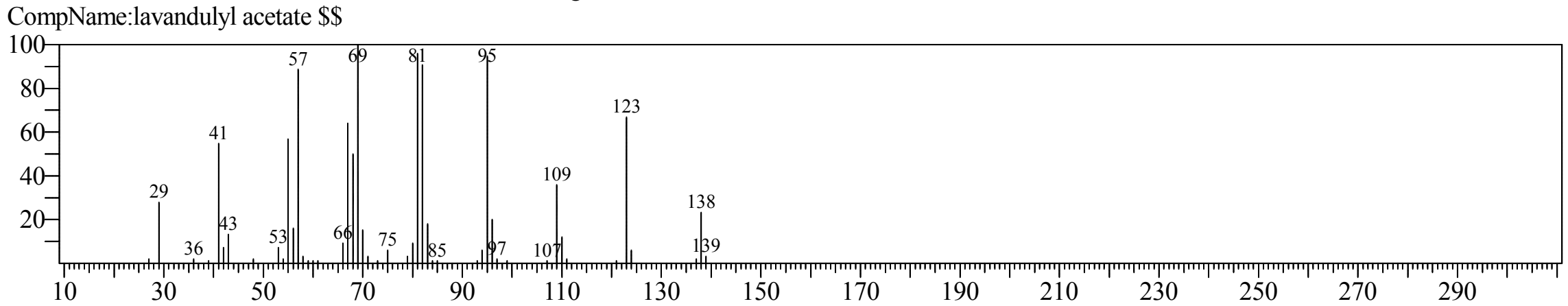

Hit#:13 Entry:124924 Library:NIST11.lib
SI:85 Formula:C20H36O2 CAS:0-00-0 MolWeight:308 RetIndex:2193
CompName:Z,Z-2,15-Octadecedien-1-ol acetate $$ (2Z,15Z)-2,15-Octadecadienyl acetate # $$

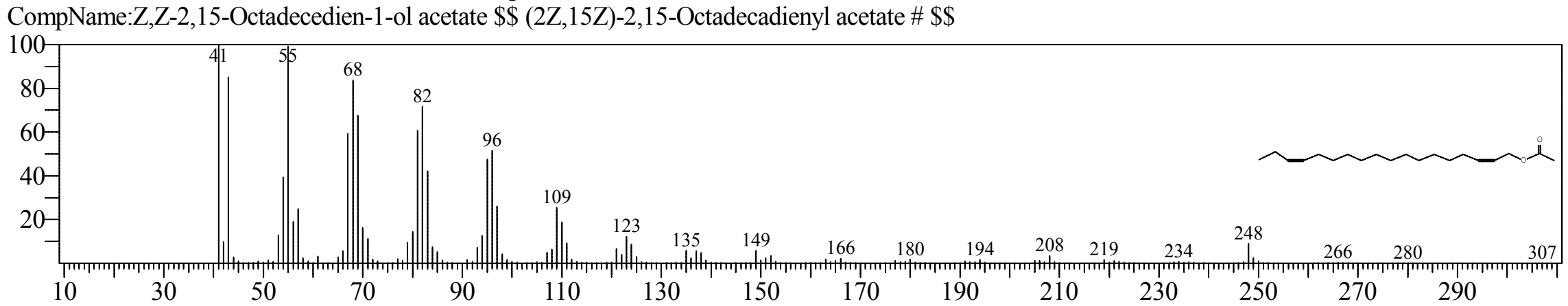

Hit#:14 Entry:22088 Library:NIST05s.LIB
SI:85 Formula:C18H36O CAS:7390-81-0 MolWeight:268 RetIndex:1901
CompName:Oxirane, hexadecyl- $$ 1,2-Epoxyoctadecane $$ 2-Hexadecyloxirane # $$

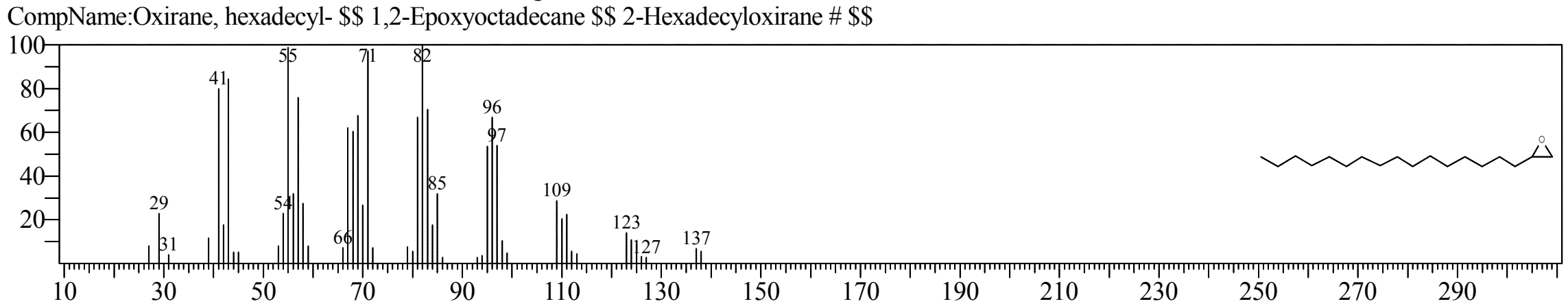

Hit#:15 Entry:105748 Library:NIST05.LIB
SI:85 Formula:C20H36O2 CAS:0-00-0 MolWeight:308 RetIndex:2193
CompName:Z,Z-2,15-Octadecedien-1-ol acetate

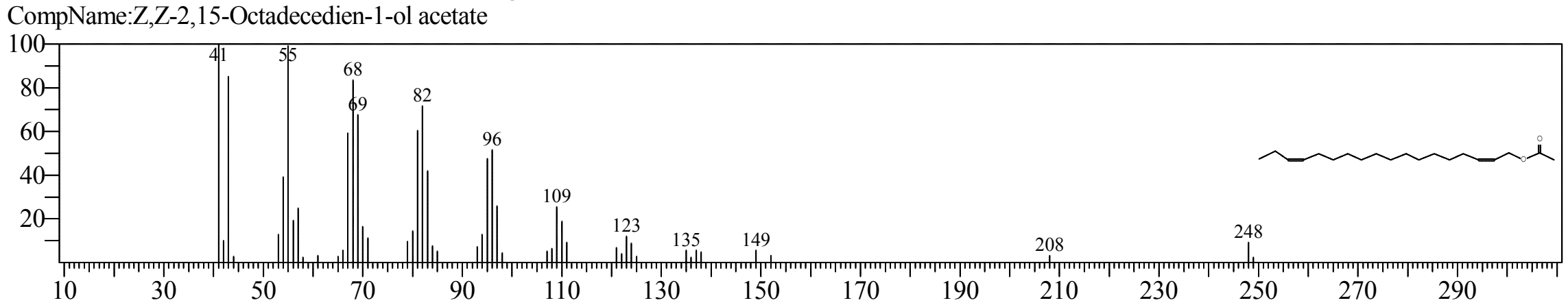

<< Target >>
Line#:2   R.Time:12.433(Scan#:893) MassPeaks:48
RawMode:Averaged 12.425-12.442(892-894) BasePeak:68.10(6749)
BG Mode:Calc. from Peak Group 1 - Event 1 Scan

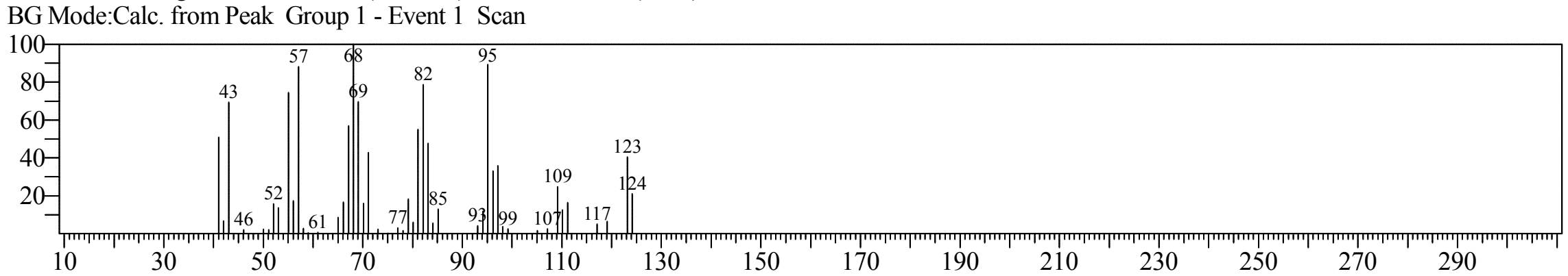

Hit#:16 Entry:72472 Library:NIST11.lib
SI:85 Formula:C16H32O CAS:629-80-1 MolWeight:240 RetIndex:1800
CompName:Hexadecanal $$ Palmitaldehyde $$ 1-Hexadecanal $$ n-Hexadecanal $$

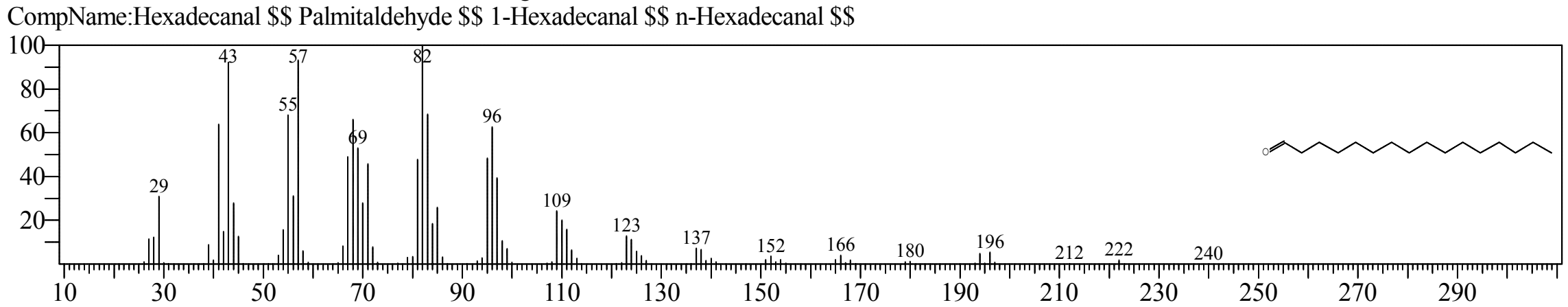

Hit#:17 Entry:82262 Library:NIST05.LIB
SI:85 Formula:C18H36O CAS:143-28-2 MolWeight:268 RetIndex:2061
CompName:Oleyl Alcohol $$ 9-Octadecen-1-ol, (Z)- $$ cis-9-Octadecen-1-ol $$ cis-9-Octadecenyl Alcohol $$ Adol 320 $$ Adol 85 $$ Atalco O $$ Cachal

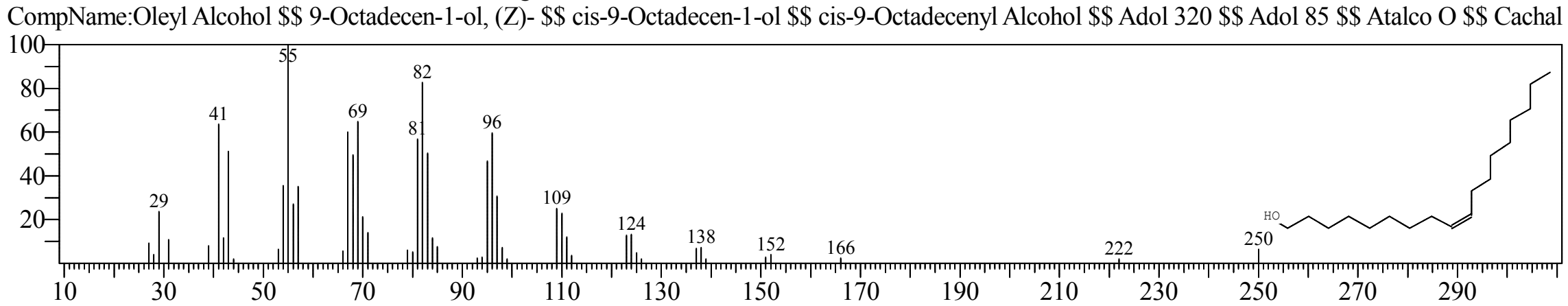

Hit#:18 Entry:48539 Library:NIST05.LIB
SI:85 Formula:C14H28O CAS:3234-28-4 MolWeight:212 RetIndex:1503
CompName:Oxirane, dodecyl- $$ 1,2-Epoxytetradecane $$ 2-Dodecyloxirane # $$

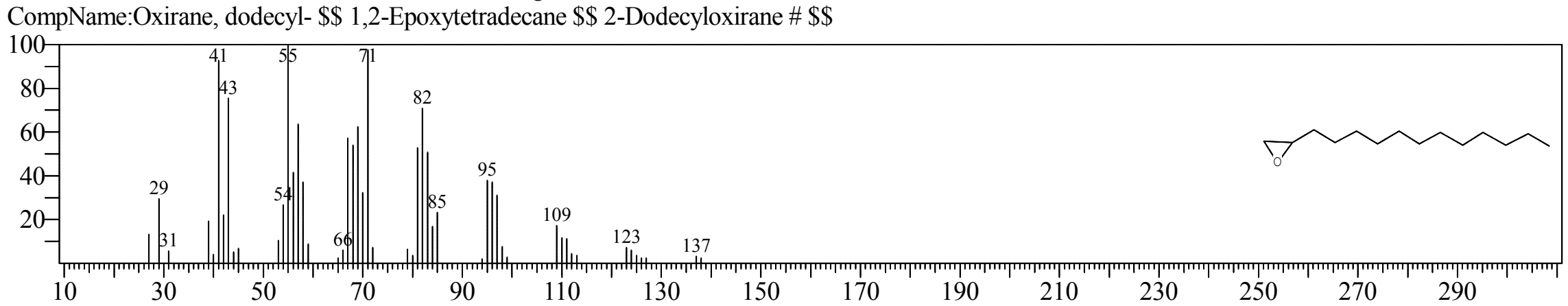

Hit#:19 Entry:79761 Library:NIST05.LIB
SI:85 Formula:C19H36 CAS:35354-38-2 MolWeight:264 RetIndex:1863
CompName:7-Octadecyne, 2-methyl- $$ 2-Methyl-7-octadecyne $$

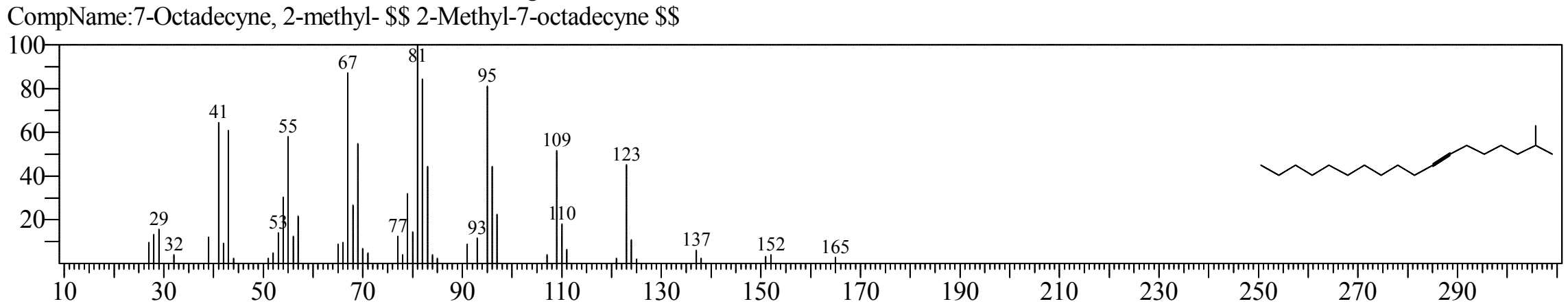

Hit#:20 Entry:82265 Library:NIST05.LIB
SI:85 Formula:C18H36O CAS:0-00-0 MolWeight:268 RetIndex:2061
CompName:Z-2-Octadecen-1-ol

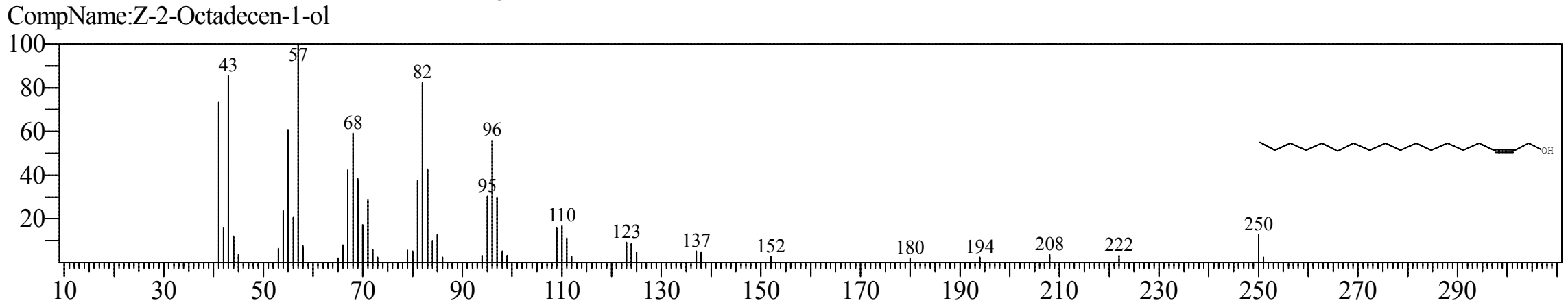

<< Target >>
Line#:3   R.Time:12.700(Scan#:925) MassPeaks:44
RawMode:Averaged 12.692-12.708(924-926) BasePeak:57.10(1792)
BG Mode:Calc. from Peak Group 1 - Event 1 Scan

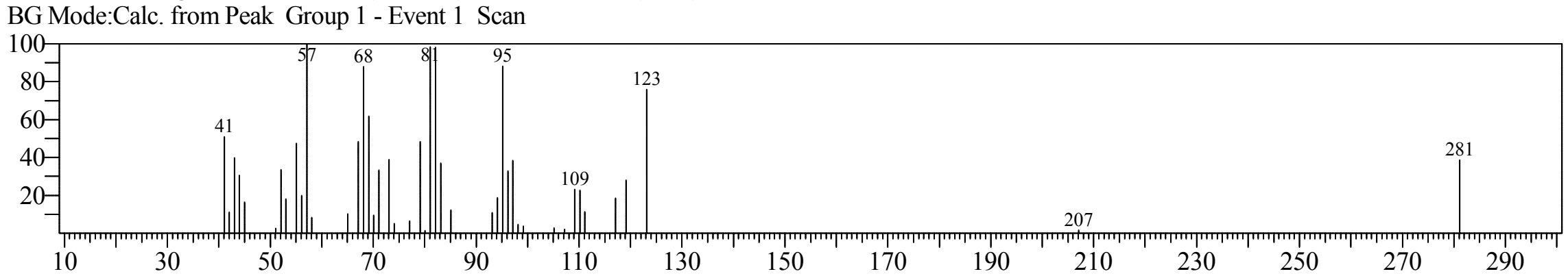

Hit#:1 Entry:98880 Library:NIST05.LIB
SI:83 Formula:C20H40O CAS:102608-53-7 MolWeight:296 RetIndex:2045
CompName:3,7,11,15-Tetramethyl-2-hexadecen-1-ol $$ (2E)-3,7,11,15-Tetramethyl-2-hexadecen-1-ol # $$

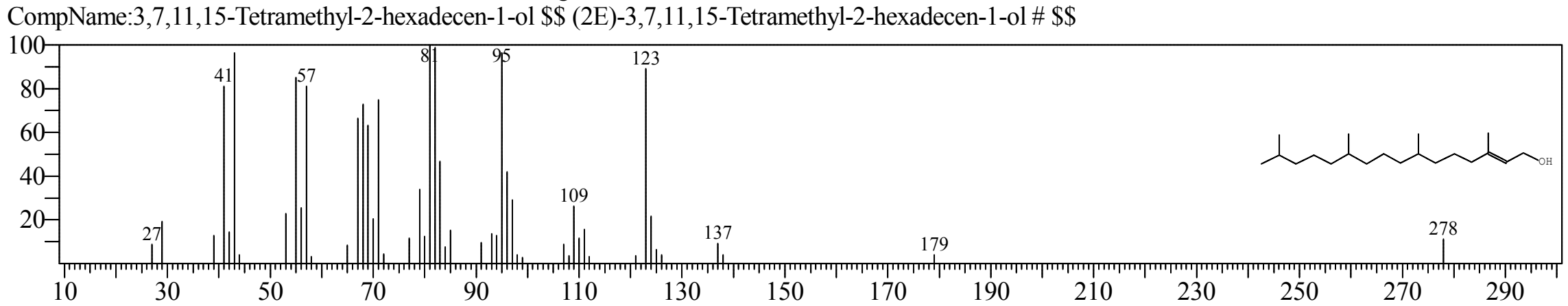

Hit#:2 Entry:207900 Library:WILEY7.LIB
SI:81 Formula:C20 H40 O CAS:150-86-7 MolWeight:296 RetIndex:0
CompName:2-Hexadecen-1-ol, 3,7,11,15-tetramethyl-, [R-[R*,R*-(E)]]- (CAS) Phytol $$ trans-Phytol $$ (E)-(7R,11R)-3,7,11,15-tetramethyl-2-hexadecen-

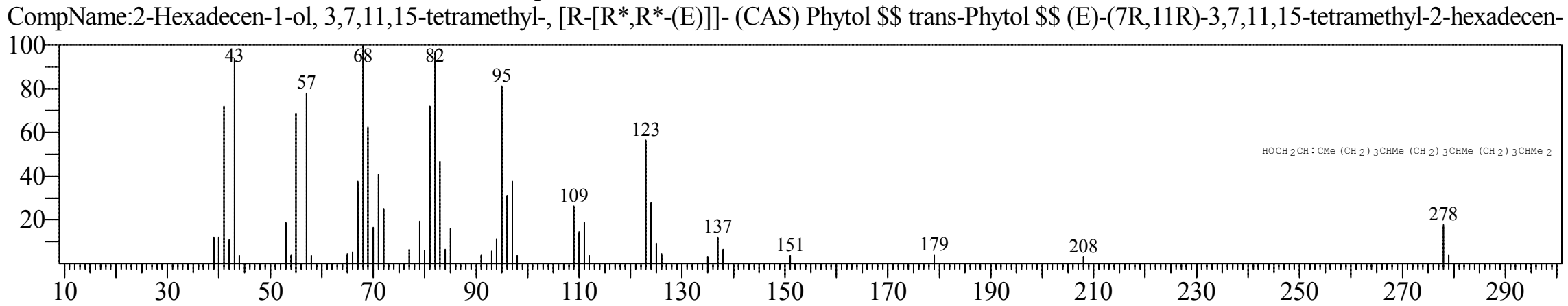

Hit#:3 Entry:189040 Library:WILEY7.LIB
SI:80 Formula:C20 H38 CAS:0-00-0 MolWeight:278 RetIndex:0
CompName:NEOPHYTADIENE $$ 2,6,10-TRIMETHYL,14-ETHYLENE-14-PENTADECNE $$

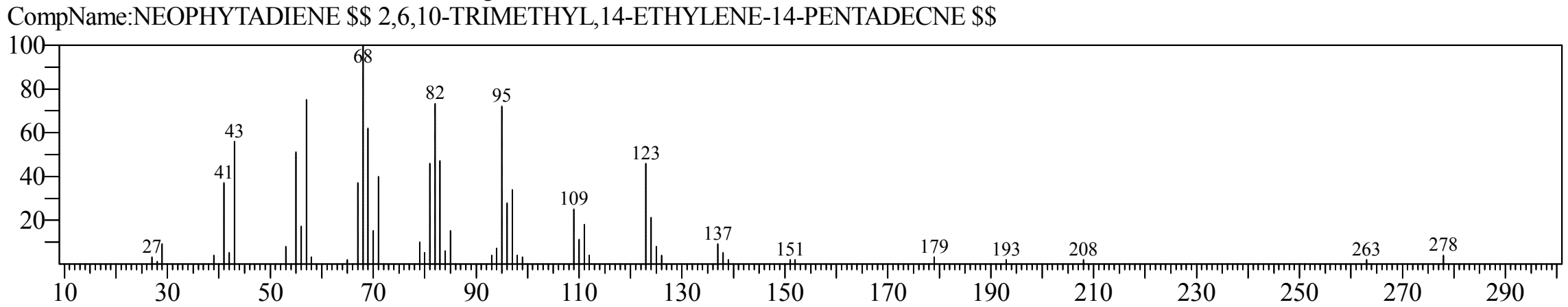

Hit#:4 Entry:55804 Library:WILEY7.LIB
SI:80 Formula:C12 H22 CAS:0-00-0 MolWeight:166 RetIndex:0
CompName:Cyclopentene, 3-heptyl- $$

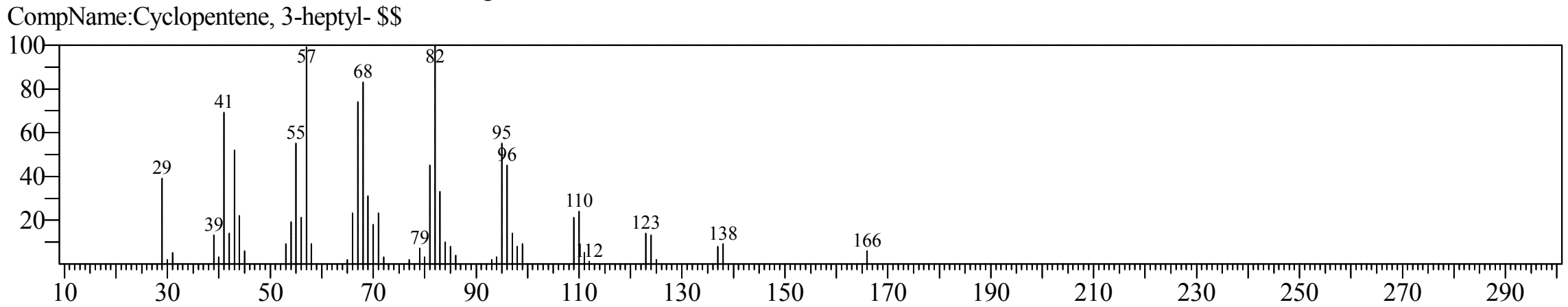

Hit#:5 Entry:14110 Library:NIST05s.LIB
SI:80 Formula:C12H24O CAS:1724-39-6 MolWeight:184 RetIndex:1627
CompName:Cyclododecanol

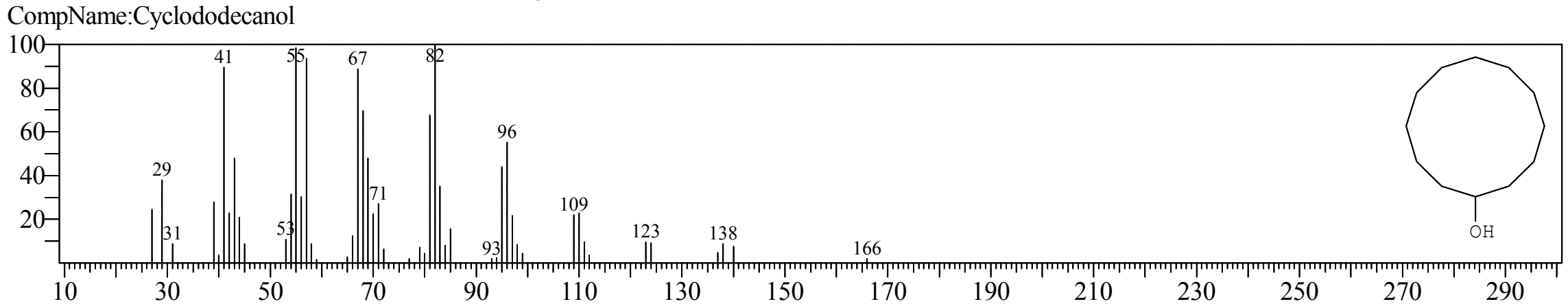

<< Target >>
Line#:3 R.Time:12.700(Scan#:925) MassPeaks:44
RawMode:Averaged 12.692-12.708(924-926) BasePeak:57.10(1792)
BG Mode:Calc. from Peak Group 1 - Event 1 Scan

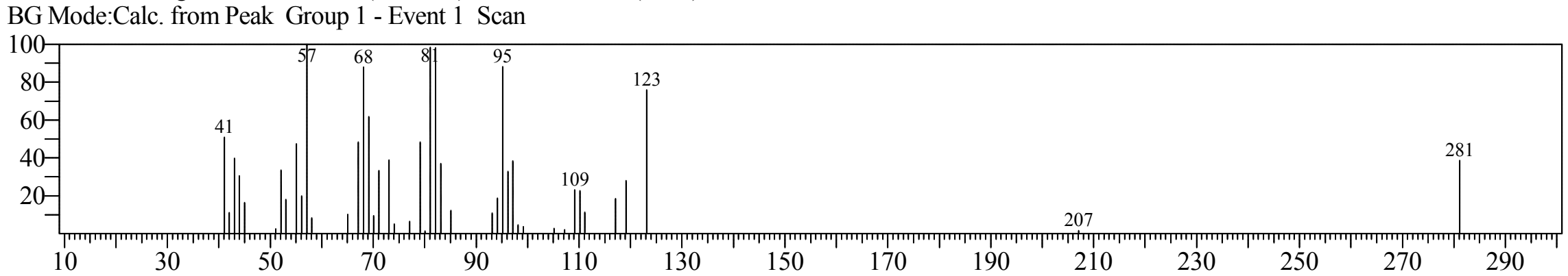

Hit#:6 Entry:91119 Library:WILEY7.LIB
SI:80 Formula:C12 H20 O2 CAS:25905-14-0 MolWeight:196 RetIndex:0
CompName:lavandulyl acetate $$

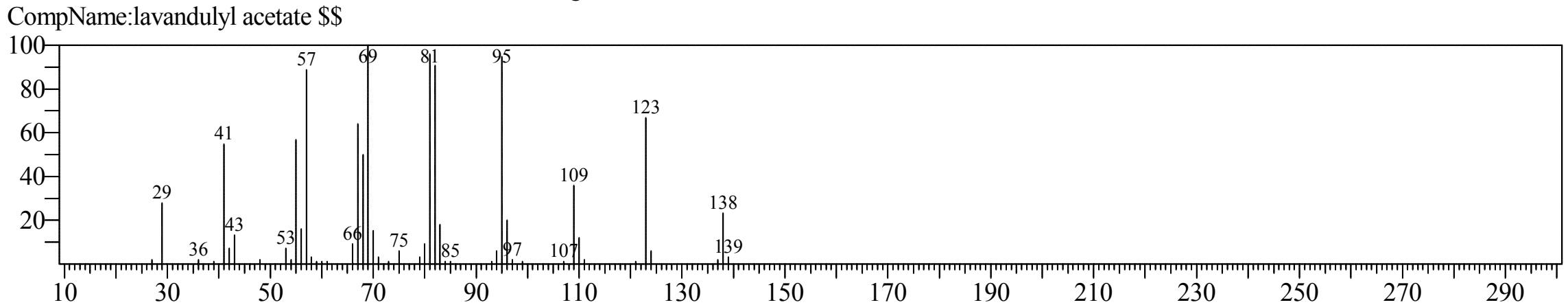

Hit#:7 Entry:57144 Library:NIST05.LIB
SI:79 Formula:C15H30O CAS:2765-11-9 MolWeight:226 RetIndex:1701
CompName:Pentadecanal-

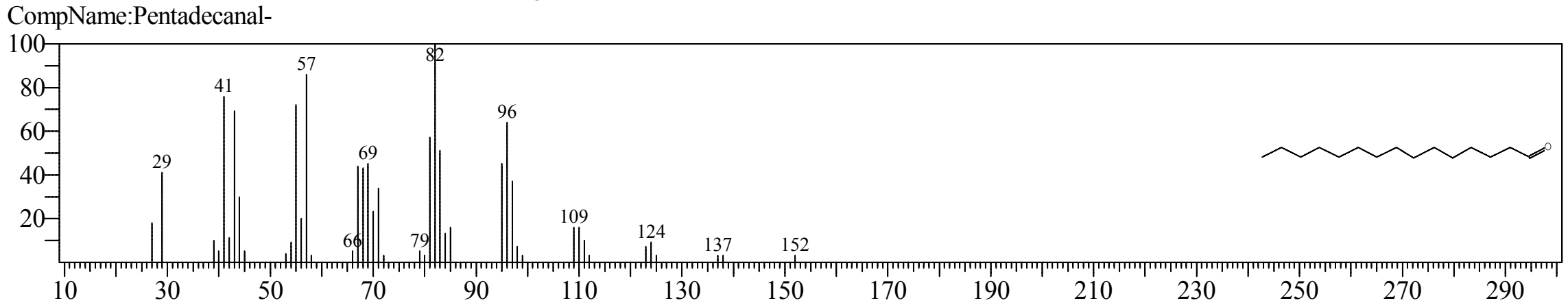

Hit#:8 Entry:95043 Library:NIST11.lib
SI:79 Formula:C16H30O3 CAS:0-00-0 MolWeight:270 RetIndex:1845
CompName:Carbonic acid, butyl undec-10-enyl ester

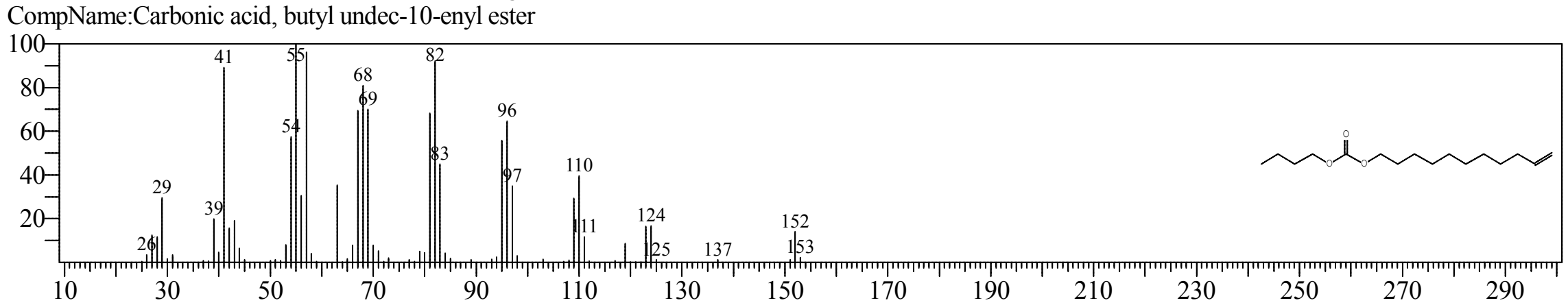

Hit#:9 Entry:83365 Library:NIST05.LIB
SI:79 Formula:C16H30O3 CAS:0-00-0 MolWeight:270 RetIndex:1845
CompName:Carbonic acid, butyl undec-10-enyl ester

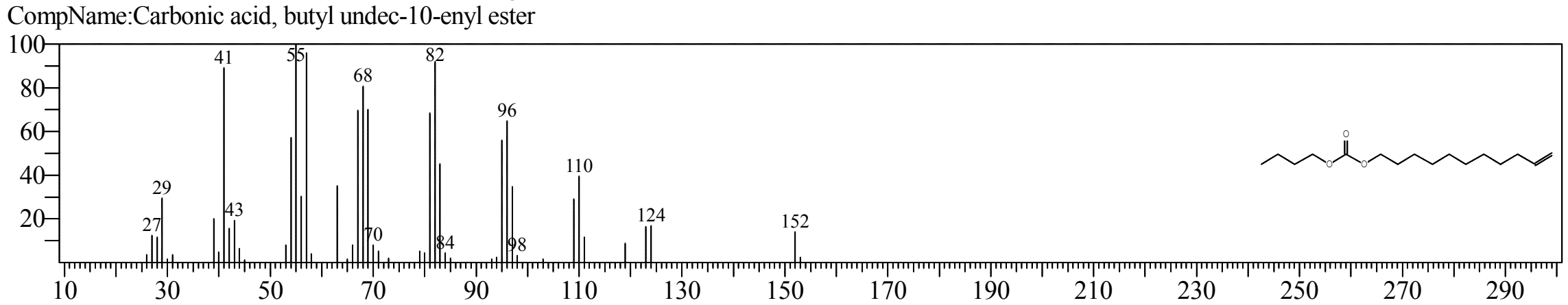

Hit#:10 Entry:145670 Library:WILEY7.LIB
SI:79 Formula:C15 H28 O2 CAS:0-00-0 MolWeight:240 RetIndex:0
CompName:CITRONELLYL VALERATE $$

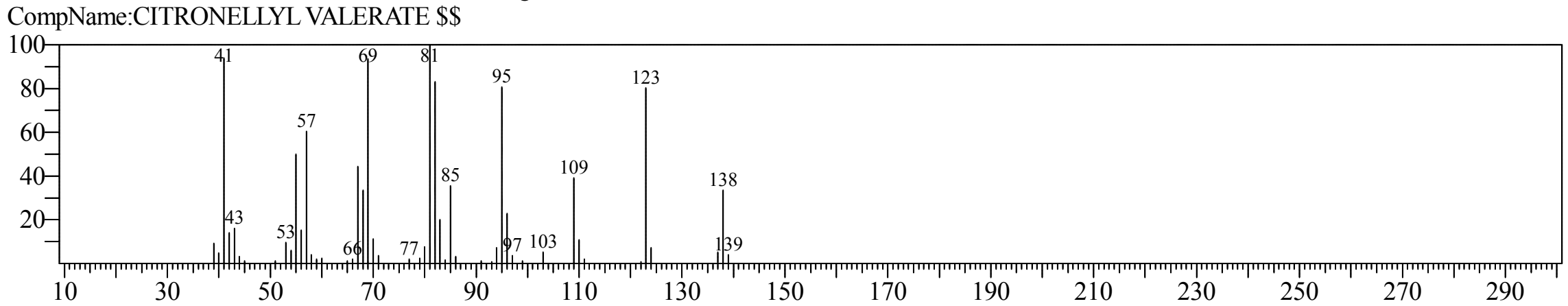

<< Target >>
Line#:3 R.Time:12.700(Scan#:925) MassPeaks:44
RawMode:Averaged 12.692-12.708(924-926) BasePeak:57.10(1792)
BG Mode:Calc. from Peak Group 1 - Event 1 Scan

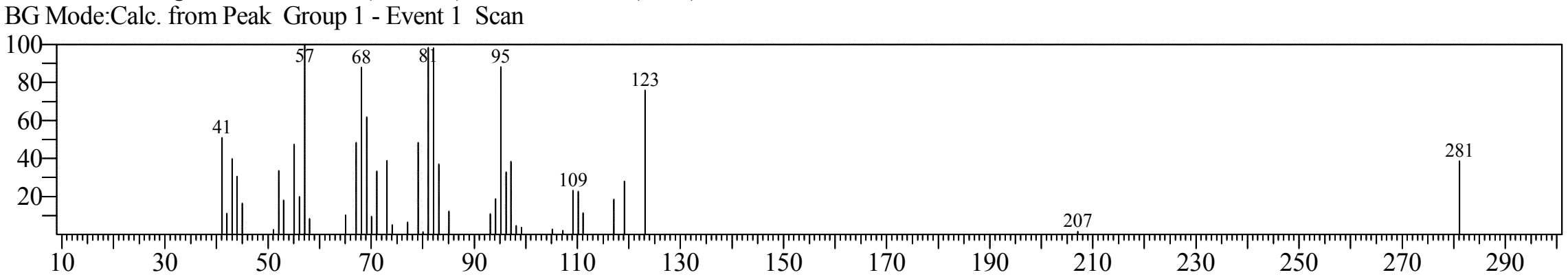

Hit#:11 Entry:72472 Library:NIST11.lib
SI:79 Formula:C16H32O CAS:629-80-1 MolWeight:240 RetIndex:1800
CompName:Hexadecanal $$ Palmitaldehyde $$ 1-Hexadecanal $$ n-Hexadecanal $$

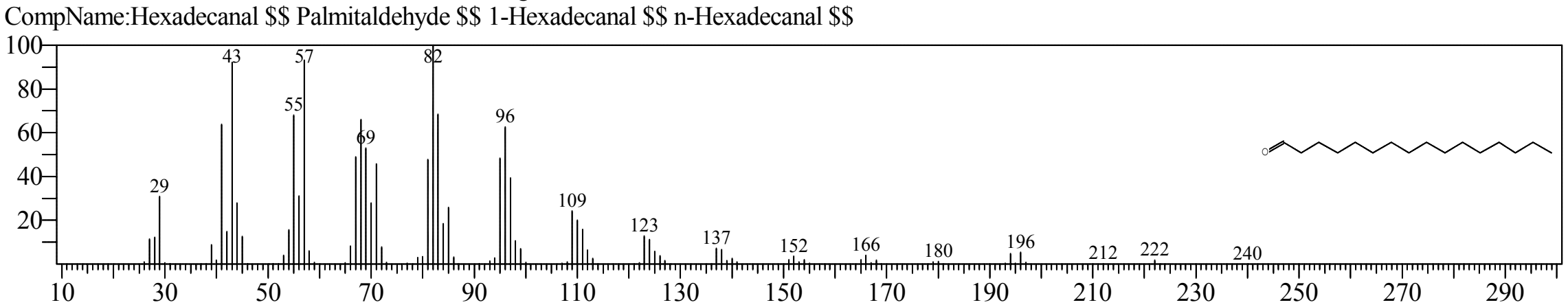

Hit#:12 Entry:79761 Library:NIST05.LIB
SI:79 Formula:C19H36 CAS:35354-38-2 MolWeight:264 RetIndex:1863
CompName:7-Octadecyne, 2-methyl- $$ 2-Methyl-7-octadecyne $$

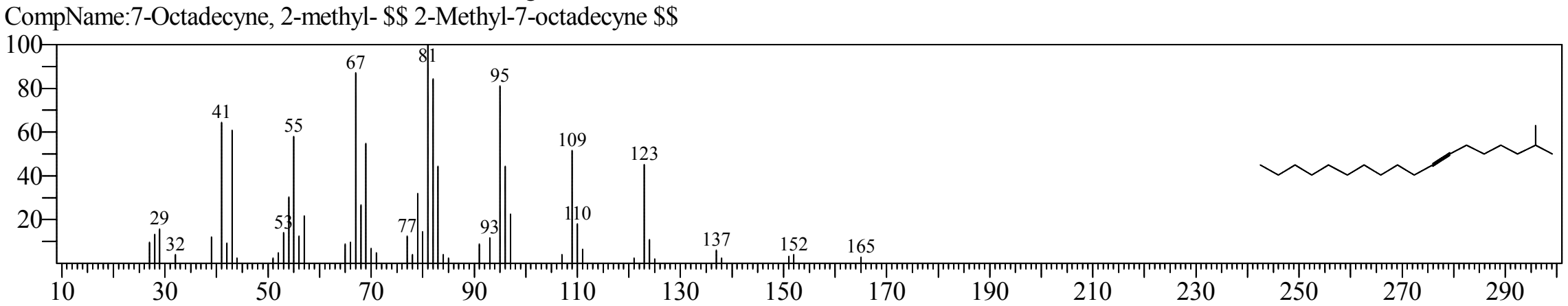

Hit#:13 Entry:147507 Library:NIST11.lib
SI:79 Formula:C22H42O2 CAS:0-00-0 MolWeight:338 RetIndex:2168
CompName:Phytol, acetate

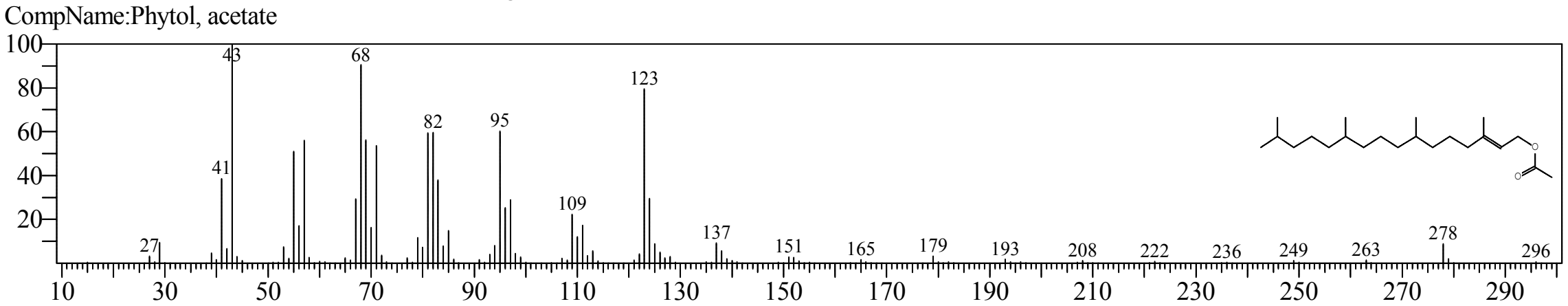

Hit#:14 Entry:10353 Library:NIST11s.lib
SI:79 Formula:C10H20O CAS:2216-51-5 MolWeight:156 RetIndex:1164
CompName:Levomenthol $$ Cyclohexanol, 5-methyl-2-(1-methylethyl)-, [1R-(1.alpha.,2.beta.,5.alpha.)]- $$ L-(-)-Menthol $$ Menthol, (1R,3R,4S)-(-)- $$

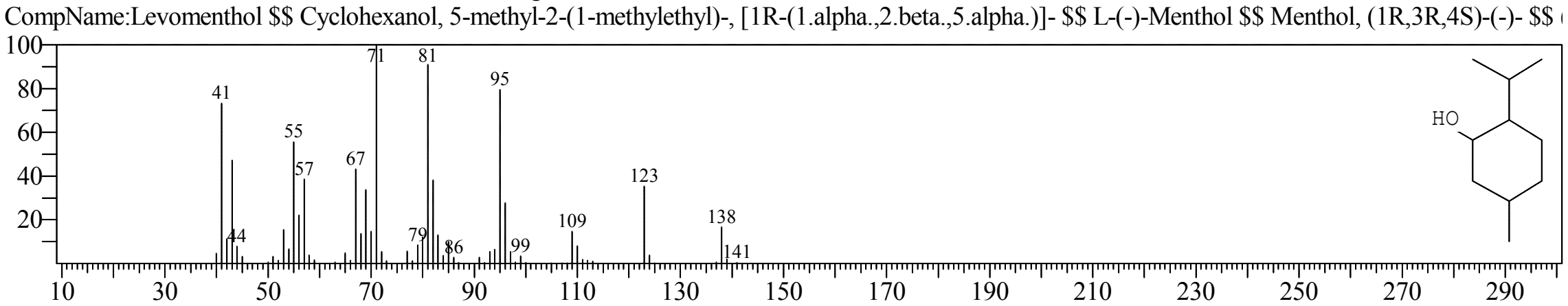

Hit#:15 Entry:20938 Library:NIST05s.LIB
SI:79 Formula:C18H34 CAS:629-89-0 MolWeight:250 RetIndex:1808
CompName:1-Octadecyne

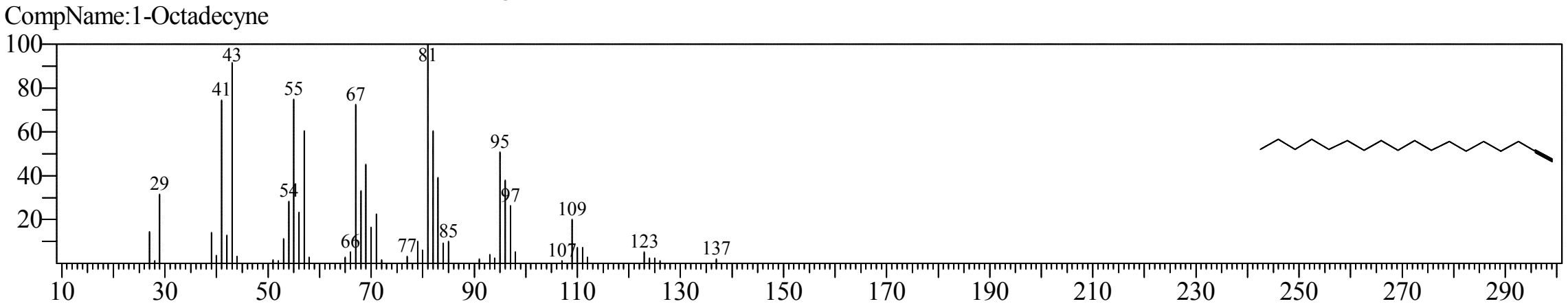

<< Target >>
Line#:3   R.Time:12.700(Scan#:925)   MassPeaks:44
RawMode:Averaged 12.692-12.708(924-926) BasePeak:57.10(1792)
BG Mode:Calc. from Peak Group 1 - Event 1 Scan

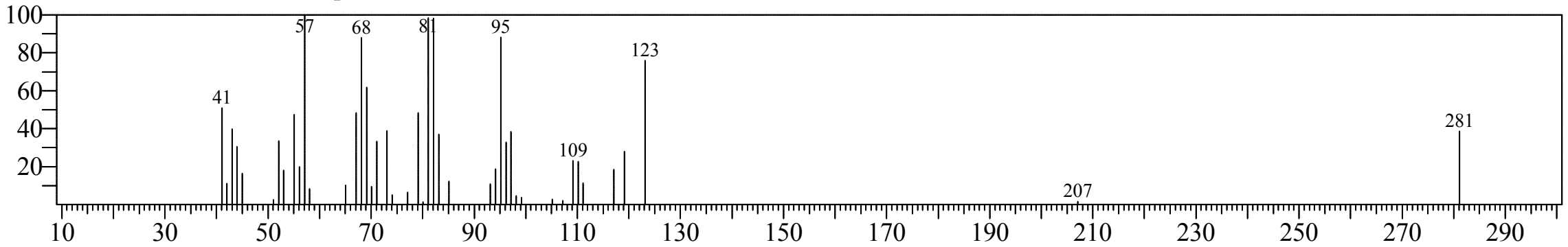

Hit#:16 Entry:88250 Library:NIST05.LIB
SI:78 Formula:C20H38 CAS:71899-38-2 MolWeight:278 RetIndex:2027
CompName:9-Eicosyne $$ 9-Icosyne # $$

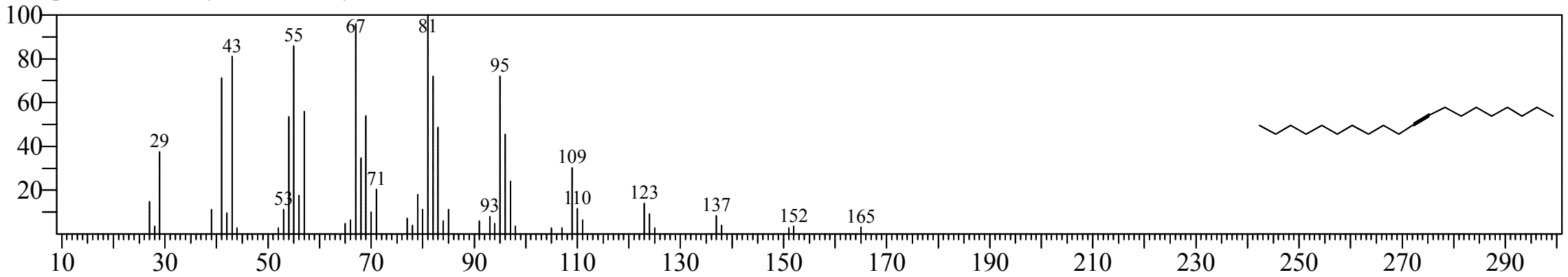

Hit#:17 Entry:17632 Library:NIST05s.LIB
SI:78 Formula:C13H24O2 CAS:141-14-0 MolWeight:212 RetIndex:1402
CompName:6-Octen-1-ol, 3,7-dimethyl-, propanoate $$ 6-Octen-1-ol, 3,7-dimethyl-, propionate $$ Citronellyl propionate $$ Citronellyl n-proprionate $$ 3,

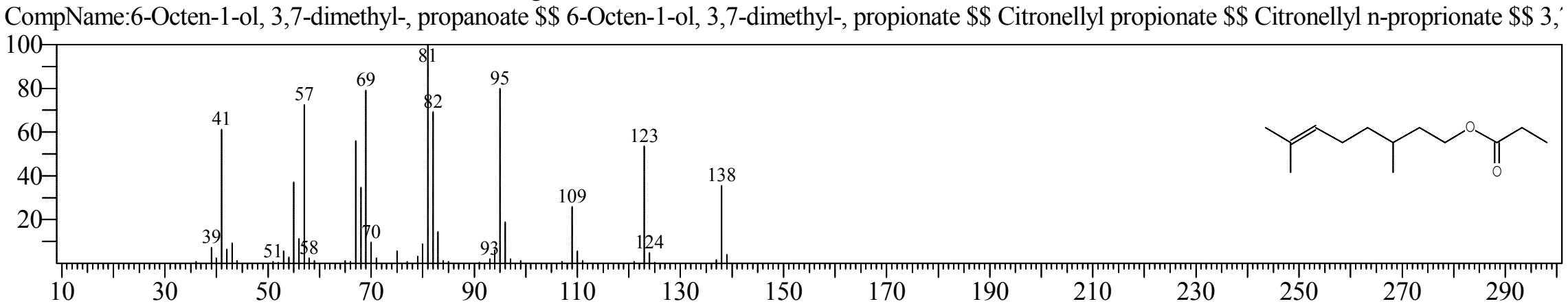

Hit#:18 Entry:93510 Library:WILEY7.LIB
SI:78 Formula:C12 H22 O2 CAS:150-84-5 MolWeight:198 RetIndex:0
CompName:Citronellyl acetate $$ 6-Octen-1-ol, 3,7-dimethyl-, acetate (CAS) Citronellol acetate $$ Natural rhodinol, acetylated $$ 3,7-Dimethyl-6-octenyl a

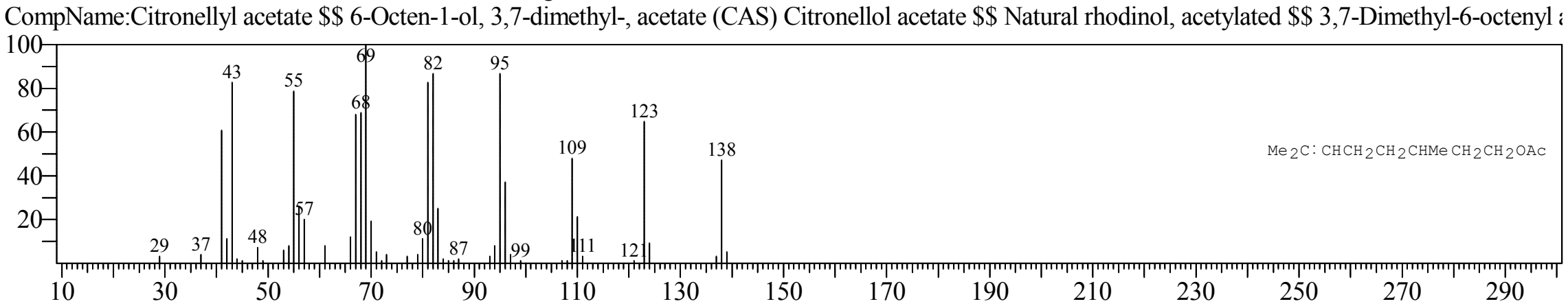

Hit#:19 Entry:189041 Library:WILEY7.LIB
SI:78 Formula:C20 H38 CAS:0-00-0 MolWeight:278 RetIndex:0
CompName:NEOPHYTADIENE $$ 2,6,10-TRIMETHYL,14-ETHYLENE-14-PENTADECNE $$

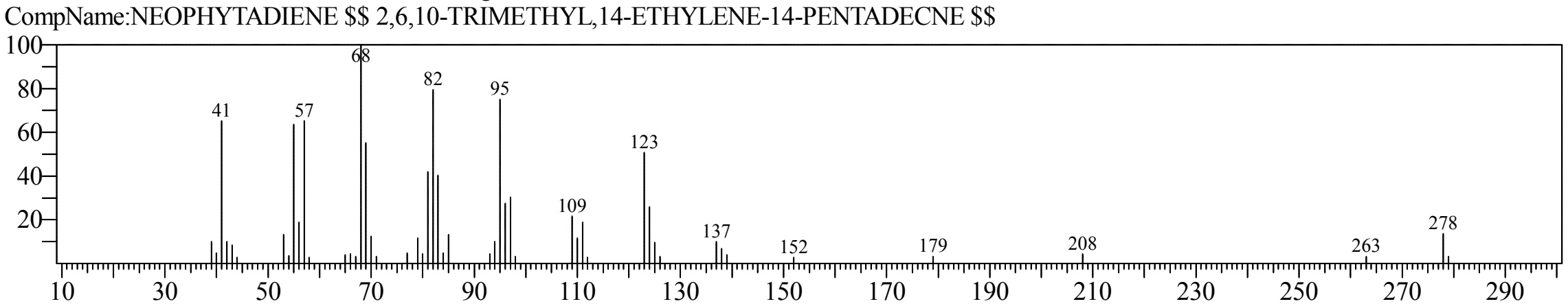

Hit#:20 Entry:82265 Library:NIST05.LIB
SI:78 Formula:C18H36O CAS:0-00-0 MolWeight:268 RetIndex:2061
CompName:Z-2-Octadecen-1-ol

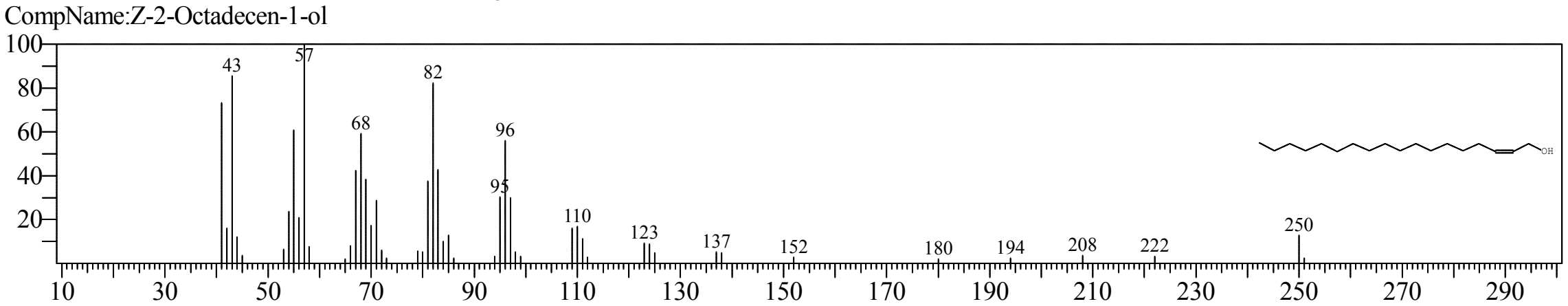

<< Target >>
Line#:4 R.Time:12.917(Scan#:951) MassPeaks:42
RawMode:Averaged 12.908-12.925(950-952) BasePeak:82.10(1962)
BG Mode:Calc. from Peak Group 1 - Event 1 Scan

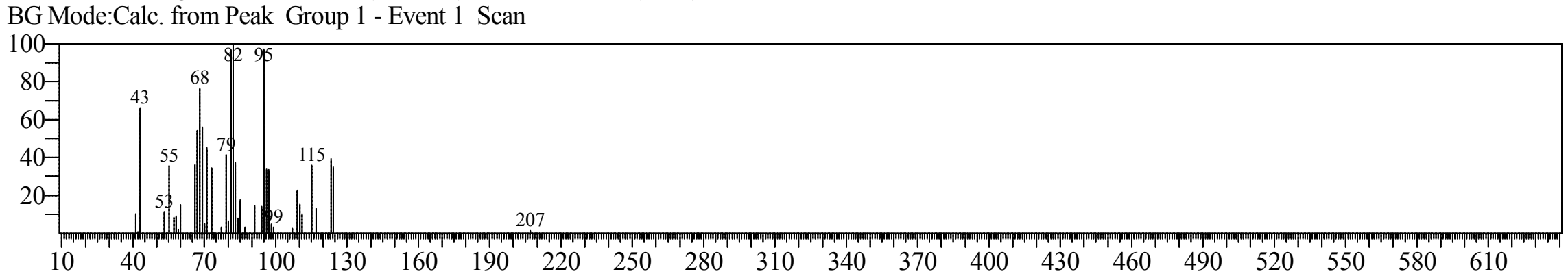

Hit#:1 Entry:161854 Library:NIST05.LIB
SI:69 Formula:C16H48O10Si9 CAS:145344-72-5 MolWeight:652 RetIndex:1716
CompName:2-(2',4',4',6',6',8',8'-Heptamethyltetrasiloxan-2'-yloxy)-2,4,4,6,6,8,8,10,10-nonamethylcyclopentasiloxane $$ 2-[(2,4,4,6,6,8,8-Heptamethyl-1,3,5

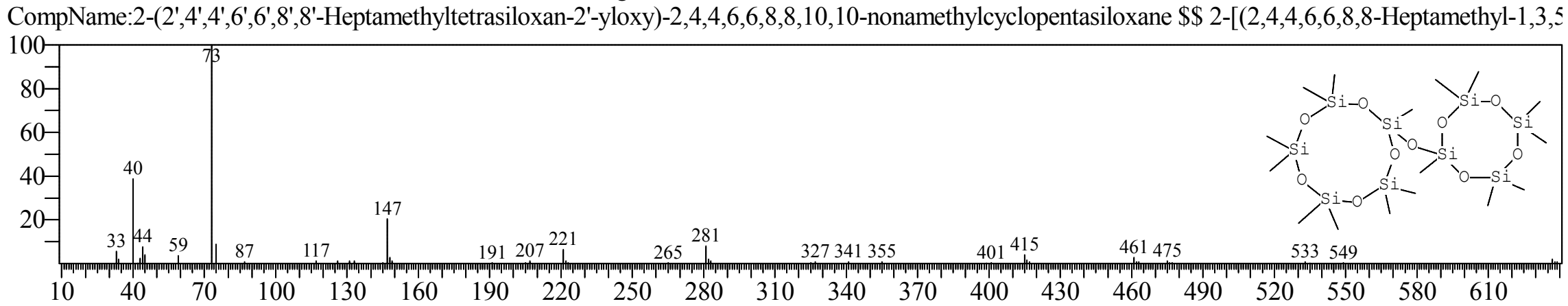

Hit#:2 Entry:306966 Library:WILEY7.LIB
SI:69 Formula:C20 H42 O4 SI4 CAS:56114-62-6 MolWeight:458 RetIndex:0
CompName:Silane, [[4-[1,2-bis[(trimethylsilyl)oxy]ethyl]-1,2-phenylene]bis(oxy)]bis[trimethyl- (CAS) 3,4-DIHYDROXYPHENYLGLYCOL-TETRATMS

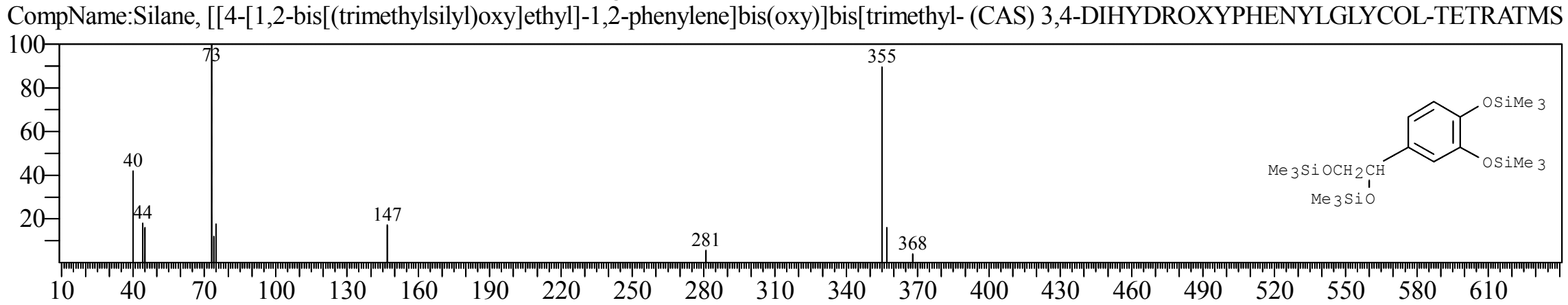

Hit#:3 Entry:32238 Library:NIST05.LIB
SI:66 Formula:C11H24Si CAS:252991-06-3 MolWeight:184 RetIndex:1015
CompName:Silane, trimethyl-7-octenyl- $$ Trimethyl(7-octenyl)silane # $$

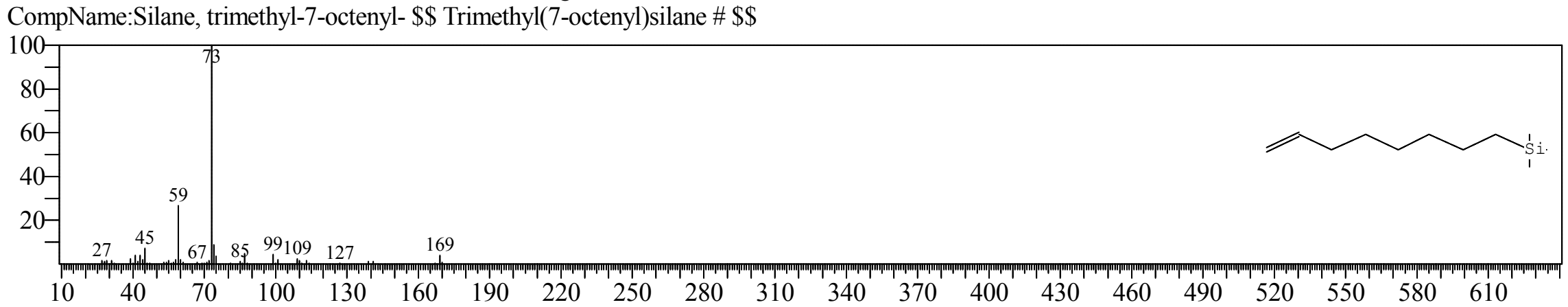

Hit#:4 Entry:76222 Library:WILEY7.LIB
SI:66 Formula:C11 H24 SI CAS:0-00-0 MolWeight:184 RetIndex:0
CompName:SILANE, TRIMETHYL-7-OCTENYL- $$

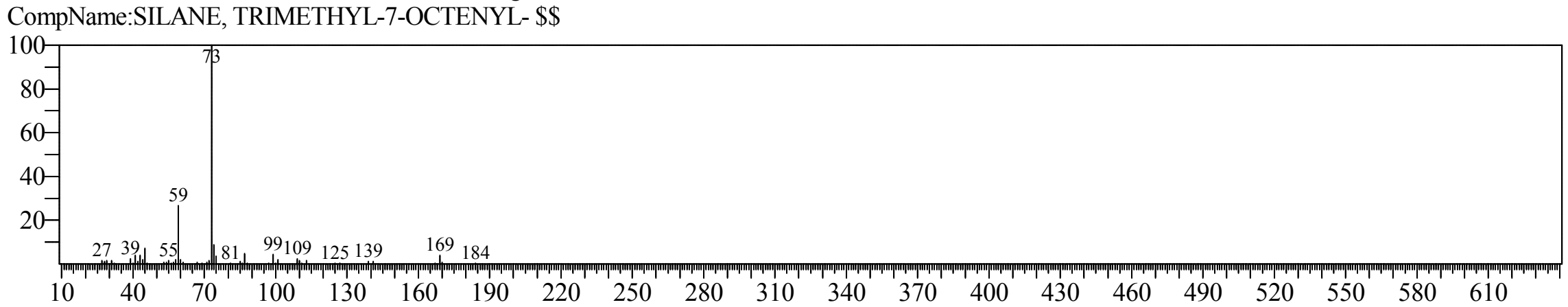

Hit#:5 Entry:8329 Library:NIST05.LIB
SI:66 Formula:C7H16O2 CAS:74421-00-4 MolWeight:132 RetIndex:720
CompName:Butane, 2,3-dimethoxy-2-methyl- $$ 2,3-Dimethoxy-2-methylbutane # $$

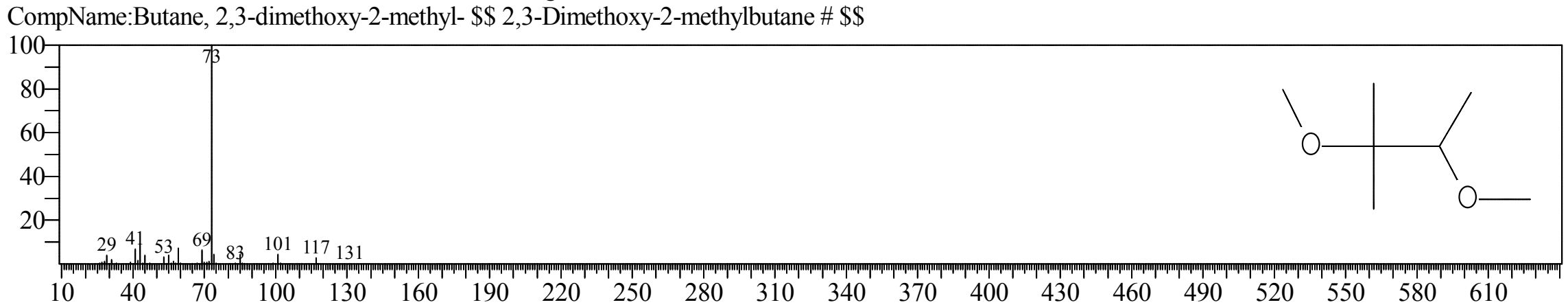

<< Target >>
Line#:4 R.Time:12.917(Scan#:951) MassPeaks:42
RawMode:Averaged 12.908-12.925(950-952) BasePeak:82.10(1962)
BG Mode:Calc. from Peak Group 1 - Event 1 Scan

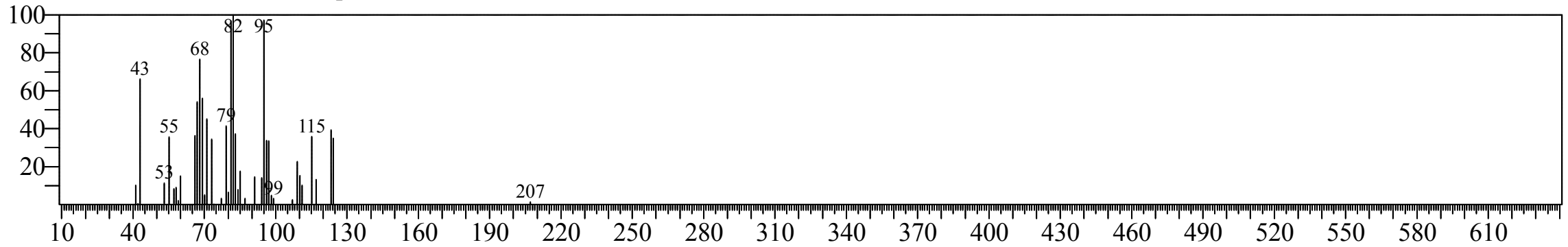

Hit#:6 Entry:7605 Library:WILEY7.LIB
SI:65 Formula:C5 H14 SI CAS:3439-38-1 MolWeight:102 RetIndex:0
CompName:Silane, ethyltrimethyl- (CAS) Ethyltrimethylsilane $$ Trimethylethylsilane $$

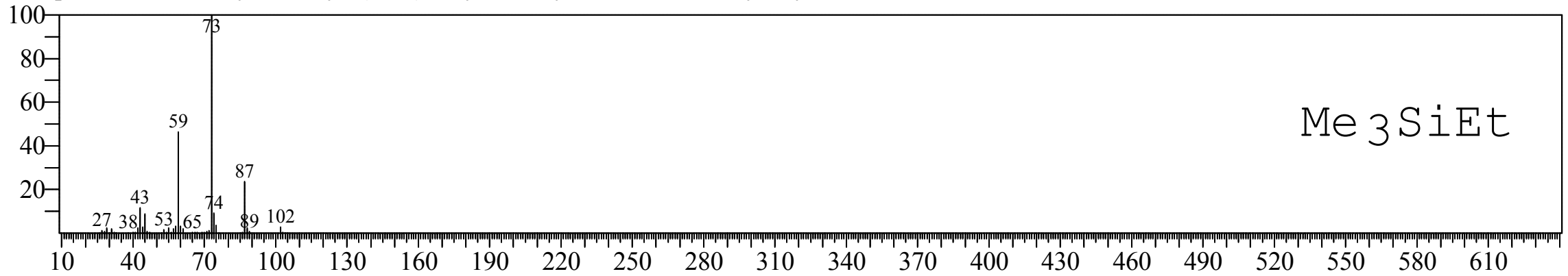

Hit#:7 Entry:27089 Library:NIST11.lib
SI:65 Formula:C10H20O2 CAS:4359-57-3 MolWeight:172 RetIndex:1235
CompName:1,3-Dioxolane, 2-heptyl- $$ 2-Heptyl-1,3-dioxolane # $$

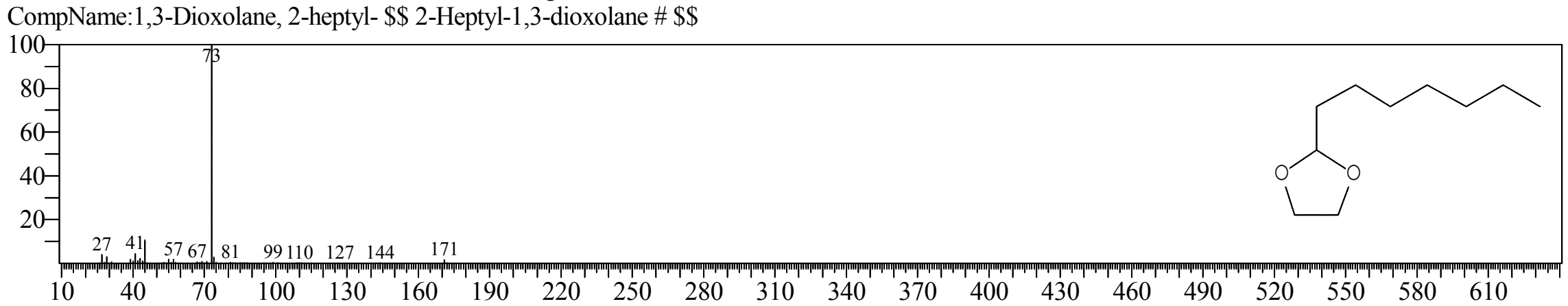

Hit#:8 Entry:313972 Library:WILEY7.LIB
SI:65 Formula:C24 H50 O4 SI3 CAS:129745-62-6 MolWeight:486 RetIndex:0
CompName:(-)-(4R,5S,6R)-4,5,6-Tris-{[(tert-butyl)dimethylsilyl]oxy}cyclohex-2-en-1-one $$

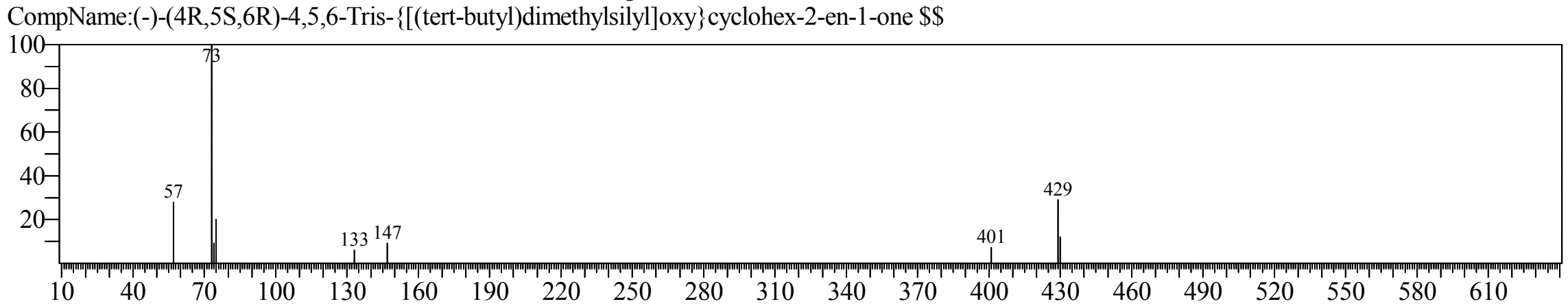

Hit#:9 Entry:48310 Library:WILEY7.LIB
SI:65 Formula:C9 H21 N O CAS:0-00-0 MolWeight:159 RetIndex:0
CompName:N,N-DIMETHYL-2-BUTOXYISOPROPYLAMINE $$

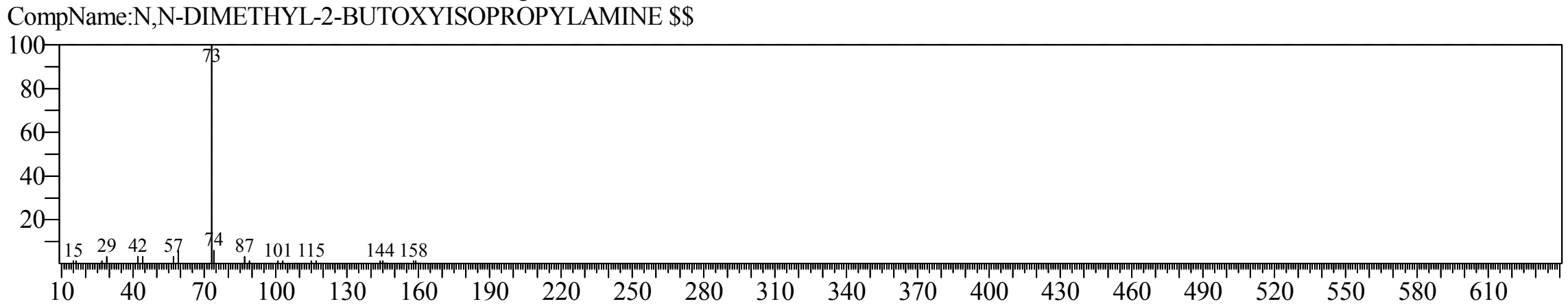

Hit#:10 Entry:12194 Library:NIST11.lib
SI:65 Formula:C8H18Si CAS:53264-56-5 MolWeight:142 RetIndex:670
CompName:Silane, trimethyl-3-penten-2-yl-, trans $$ Trimethyl[(2E)-1-methyl-2-butenyl]silane # $$

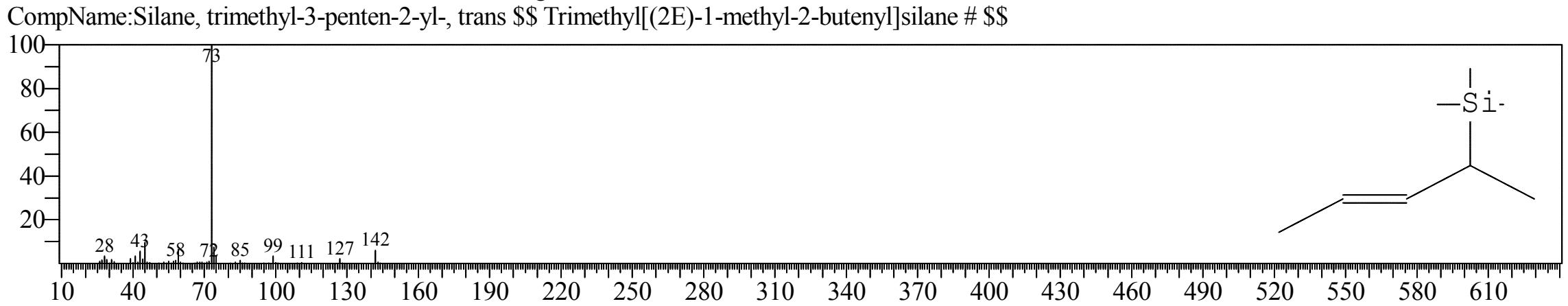

<< Target >>
Line#:4   R.Time:12.917(Scan#:951) MassPeaks:42
RawMode:Averaged 12.908-12.925(950-952) BasePeak:82.10(1962)
BG Mode:Calc. from Peak Group 1 - Event 1 Scan

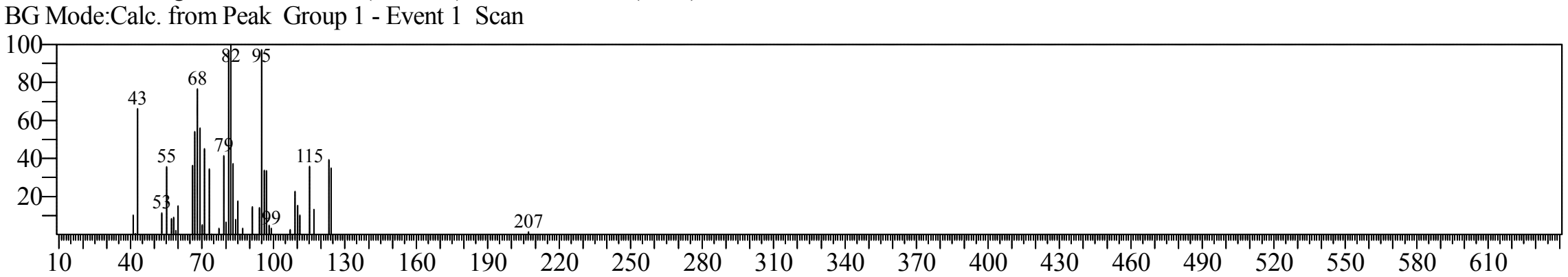

Hit#:11 Entry:31091 Library:WILEY7.LIB
SI:65 Formula:C8 H18 SI CAS:0-00-0 MolWeight:142 RetIndex:0
CompName:SILANE, TRIMETHYL-3-PENTEN-2-YL-, TRANS $$

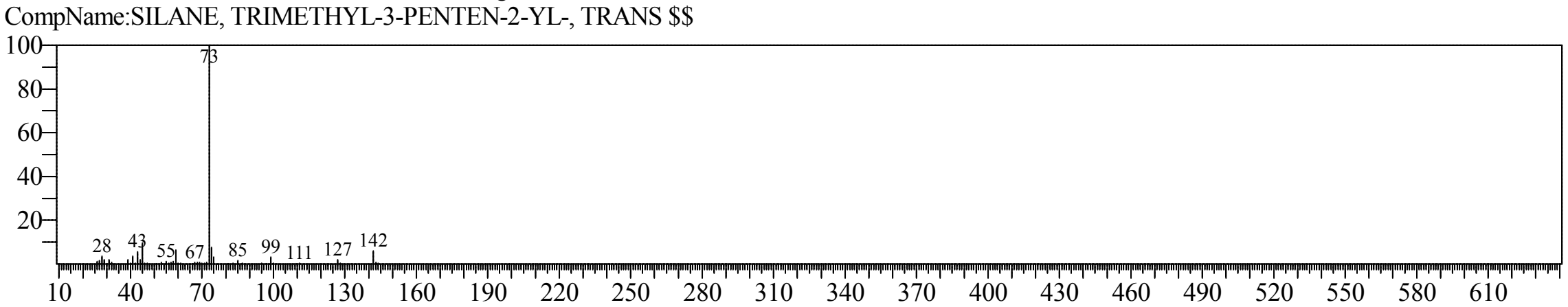

Hit#:12 Entry:53342 Library:NIST11.lib
SI:65 Formula:C11H22O2Si CAS:0-00-0 MolWeight:214 RetIndex:1200
CompName:3-Octenoic acid, trimethylsilyl ester $$ Trimethylsilyl (3E)-3-octenoate # $$

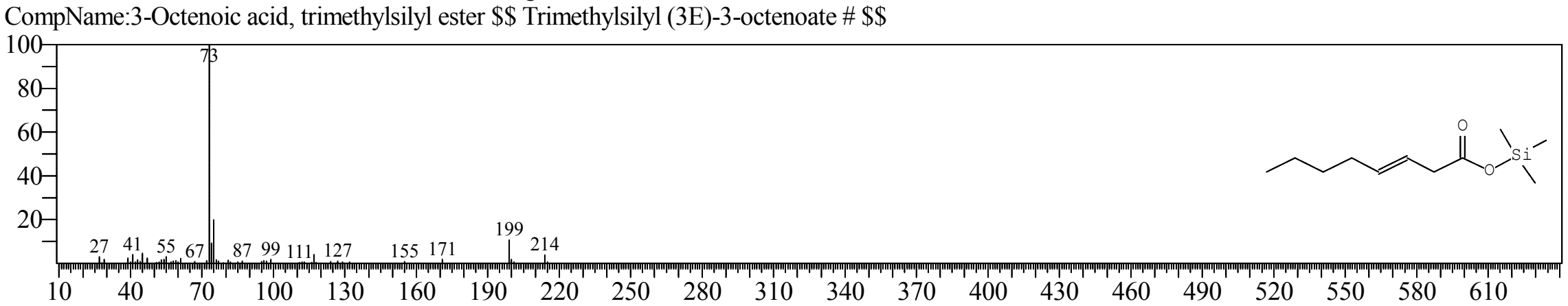

Hit#:13 Entry:49335 Library:NIST05.LIB
SI:65 Formula:C11H22O2Si CAS:0-00-0 MolWeight:214 RetIndex:1200
CompName:3-Octenoic acid, trimethylsilyl ester

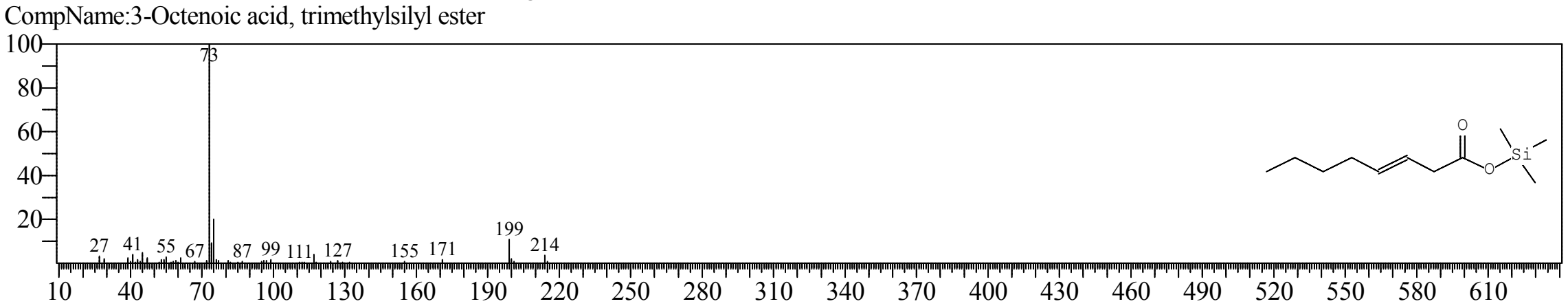

Hit#:14 Entry:112587 Library:WILEY7.LIB
SI:64 Formula:C11 H22 O2 SI CAS:0-00-0 MolWeight:214 RetIndex:0
CompName:3-OCTENEOIC ACID 1TMS $$

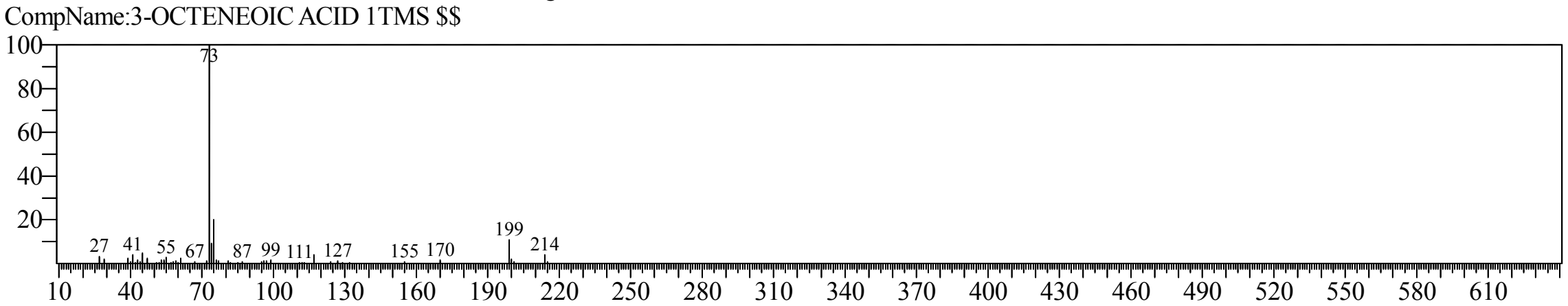

Hit#:15 Entry:21128 Library:NIST11.lib
SI:64 Formula:C8H18O3 CAS:0-00-0 MolWeight:162 RetIndex:960
CompName:1-(2-Methoxyethoxy)-2-methyl-2-propanol, methyl ether

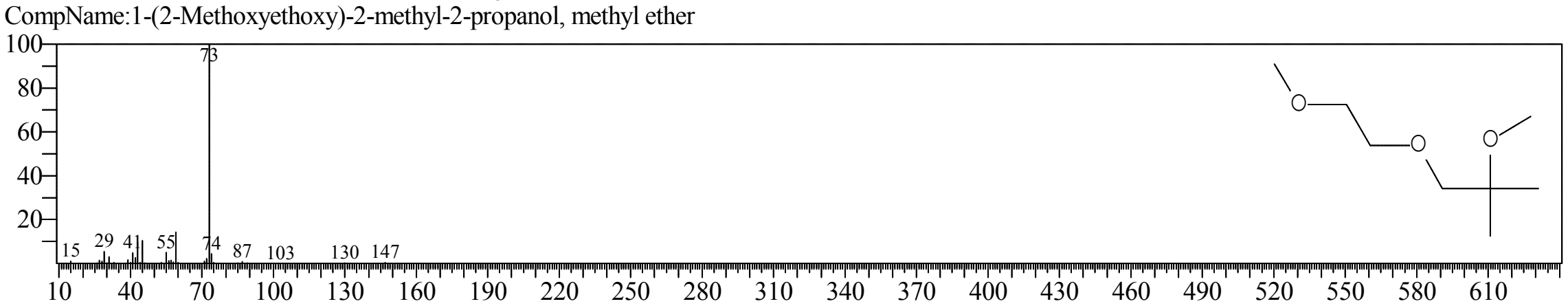

<< Target >>
Line#:4 R.Time:12.917(Scan#:951) MassPeaks:42
RawMode:Averaged 12.908-12.925(950-952) BasePeak:82.10(1962)
BG Mode:Calc. from Peak Group 1 - Event 1 Scan

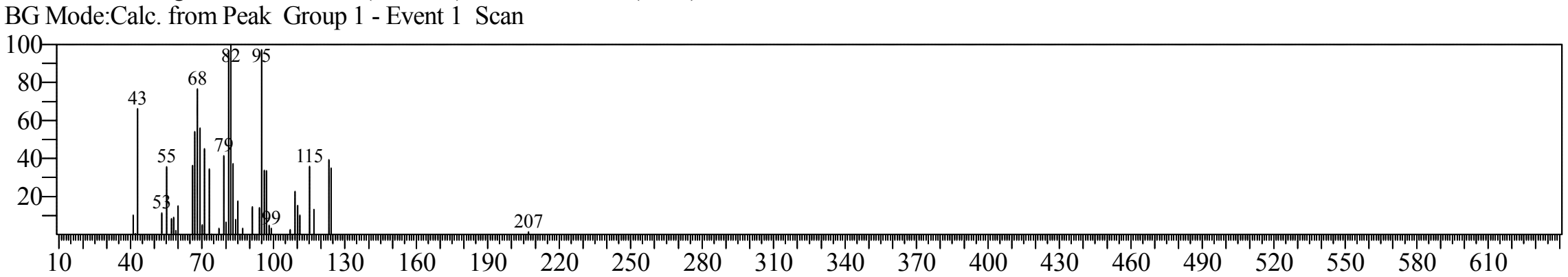

Hit#:16 Entry:45888 Library:WILEY7.LIB
SI:64 Formula:C8 H8 D4 O3 CAS:60378-35-0 MolWeight:156 RetIndex:0
CompName:3,3,6,6-TERTADEUTERIO-1,5-DIOXA-2-CIS-DECALONE $$ Pyrano[3,2-b]pyran-2(3H)-one-3,3,6-d3, hexahydro-6-d-, cis- (CAS)

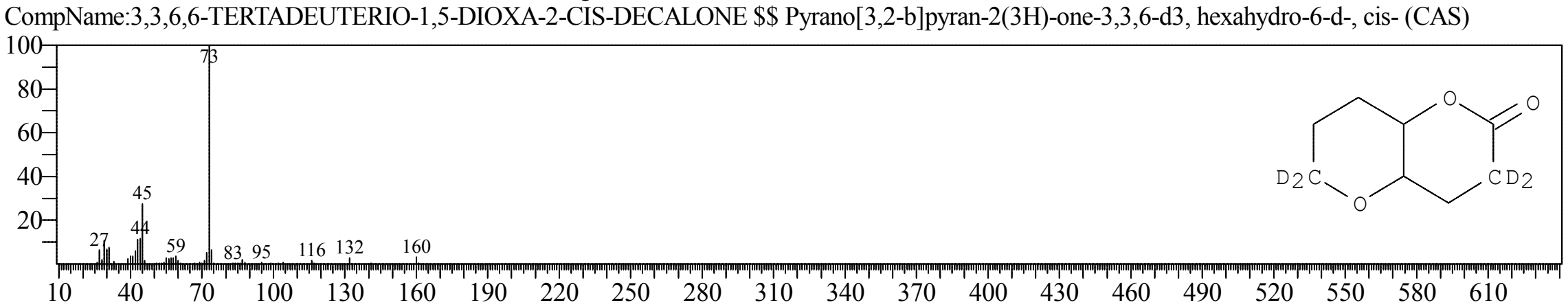

Hit#:17 Entry:102985 Library:NIST05.LIB
SI:64 Formula:C13H28O4Si2 CAS:90744-45-9 MolWeight:304 RetIndex:1468
CompName:Malonic acid, bis(2-trimethylsilylethyl ester $$ Bis[2-(trimethylsilyl)ethyl] malonate # $$

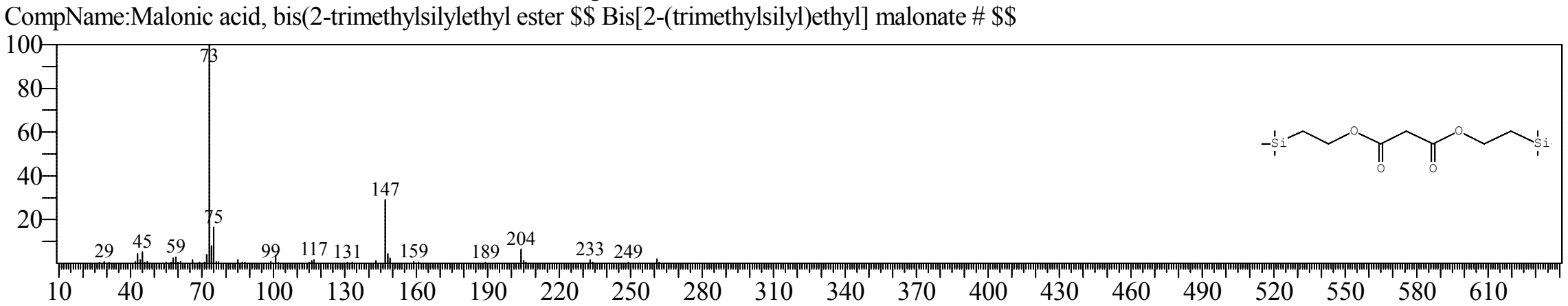

Hit#:18 Entry:7265 Library:NIST11.lib
SI:64 Formula:C7H16Si CAS:10111-13-4 MolWeight:128 RetIndex:612
CompName:Silane, trimethyl(1-methyl-1-propenyl)-, (E)- $$ Trimethyl[(1E)-1-methyl-1-propenyl]silane # $$

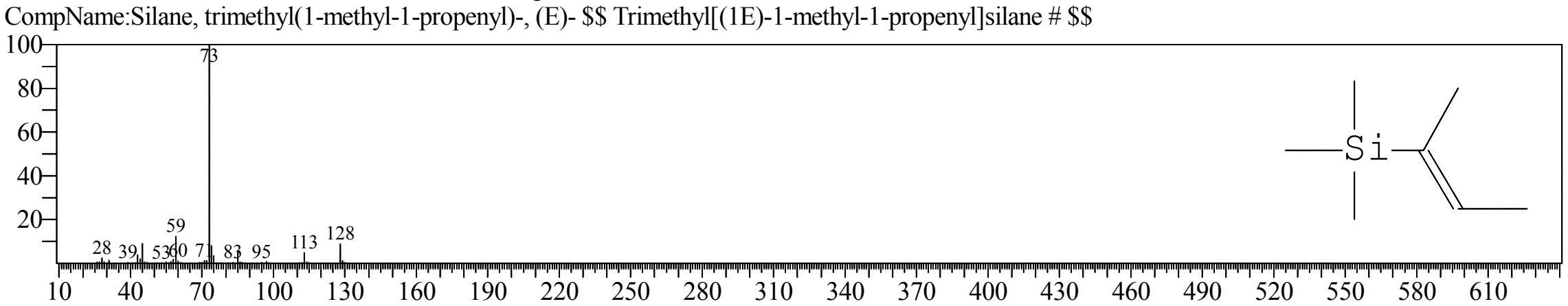

Hit#:19 Entry:310785 Library:WILEY7.LIB
SI:63 Formula:C20 H40 O5 SI4 CAS:37148-65-5 MolWeight:472 RetIndex:0
CompName:Benzeneacetic acid, .alpha.,3,4-tris[(trimethylsilyl)oxy]-, trimethylsilyl ester (CAS) TETRAKISTRIMETHYLSILYL 3,4-DIHYDROXYMAND

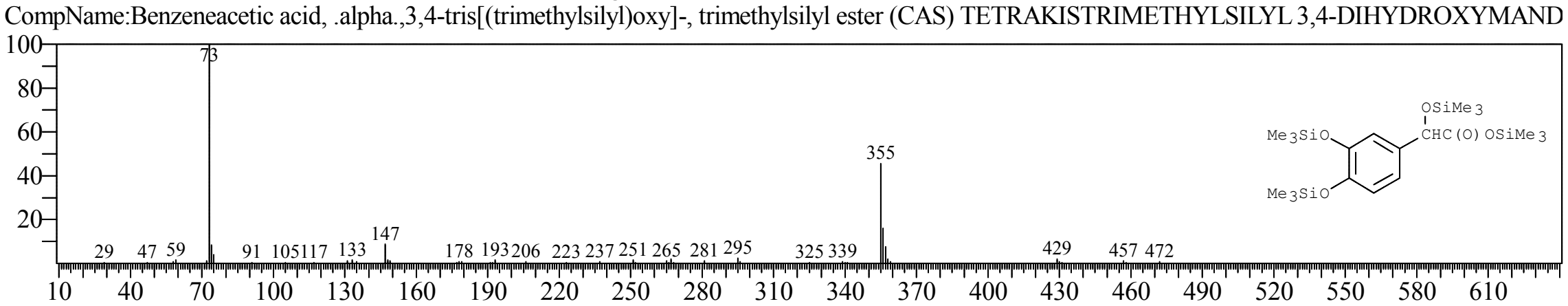

Hit#:20 Entry:92718 Library:NIST05.LIB
SI:63 Formula:C14H34Si3 CAS:136935-44-9 MolWeight:286 RetIndex:1087
CompName:1,3,5-Tris(trimethylsilyl)pent-1-ene $$ [(1Z)-3,5-Bis(trimethylsilyl)-1-pentenyl](trimethyl)silane # $$

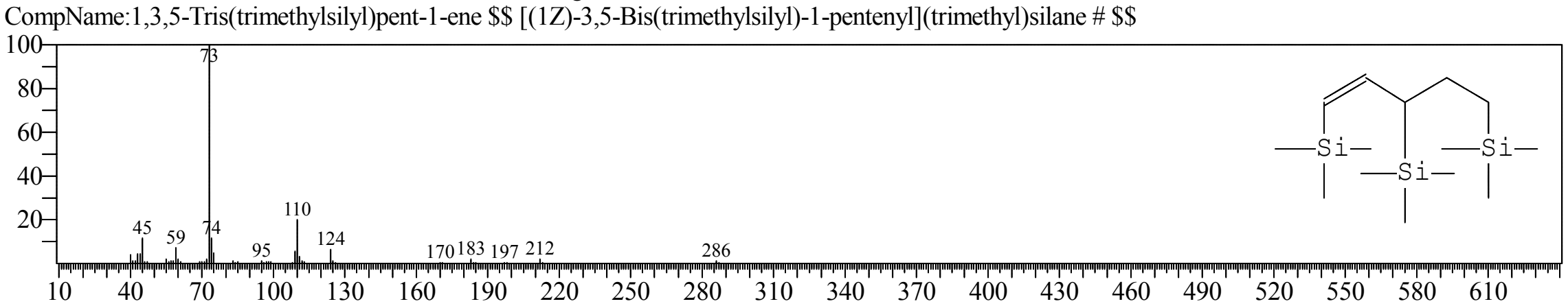

<< Target >>
Line#:5   R.Time:12.975(Scan#:958) MassPeaks:45
RawMode:Averaged 12.967-12.983(957-959) BasePeak:149.10(10464)
BG Mode:Calc. from Peak Group 1 - Event 1 Scan

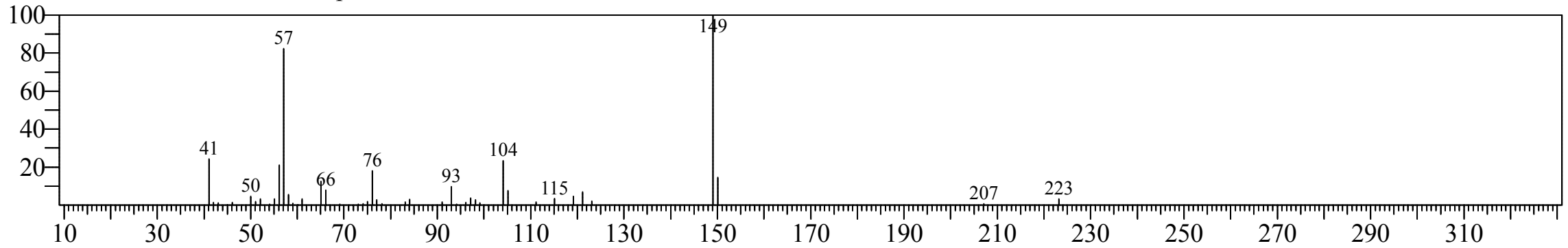

Hit#:1 Entry:24790 Library:NIST11s.lib
SI:85 Formula:C16H22O4 CAS:84-69-5 MolWeight:278 RetIndex:1908
CompName:1,2-Benzenedicarboxylic acid, bis(2-methylpropyl) ester $$ Phthalic acid, diisobutyl ester $$ Diisobutyl phthalate $$ Hexaplas M/1B $$ Isobuty

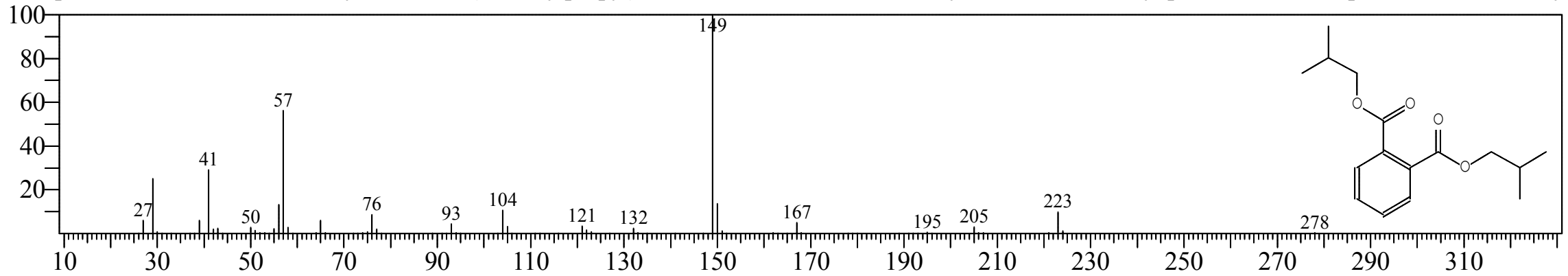

Hit#:2 Entry:188238 Library:WILEY7.LIB
SI:84 Formula:C16 H22 O4 CAS:0-00-0 MolWeight:278 RetIndex:0
CompName:diisobutyl benzene-1,2-dicarboxylate $$

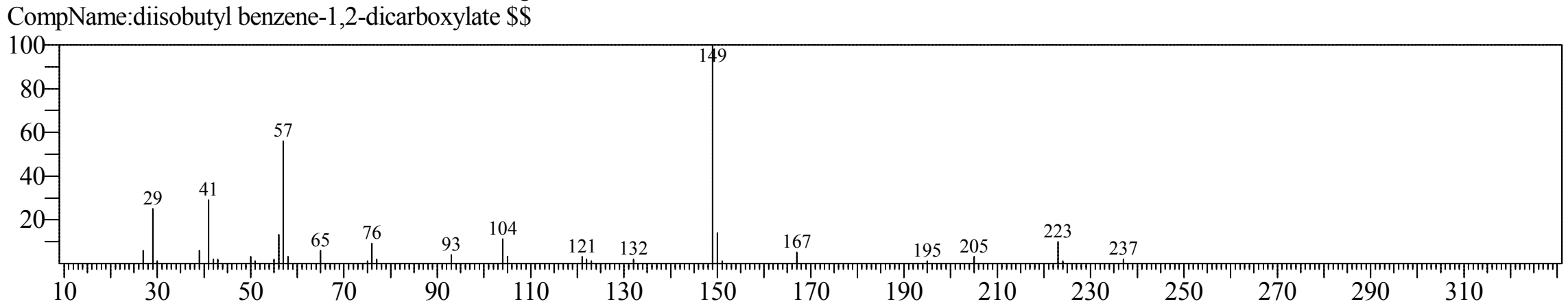

Hit#:3 Entry:188937 Library:WILEY7.LIB
SI:80 Formula:C16 H22 O4 CAS:84-74-2 MolWeight:278 RetIndex:0
CompName:1,2-Benzenedicarboxylic acid, dibutyl ester (CAS) Butyl phthalate $$ DI-N-BUTYLPHTHALATE $$ PX 104 $$ DIBUTYLPHTALATE $$ DI

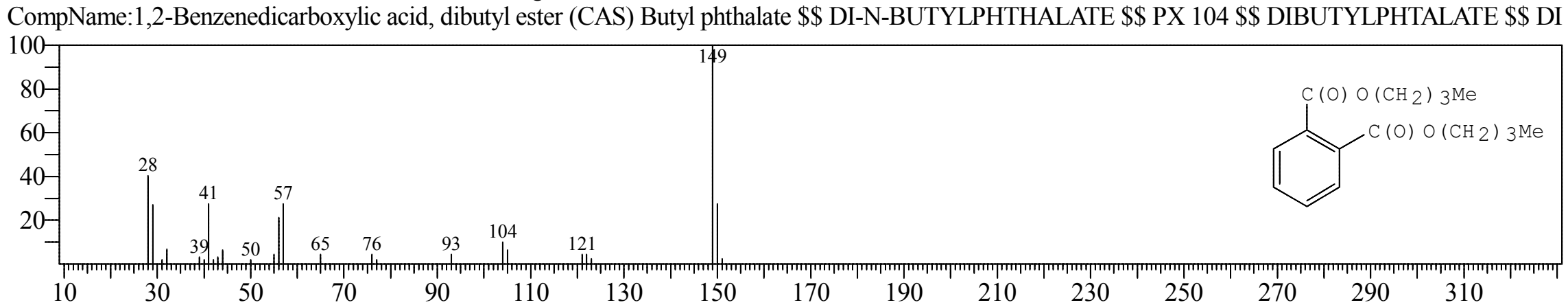

Hit#:4 Entry:123698 Library:WILEY7.LIB
SI:80 Formula:C12 H14 O4 CAS:131-70-4 MolWeight:222 RetIndex:0
CompName:1,2-Benzenedicarboxylic acid, monobutyl ester (CAS) Monobutyl phthalate $$ Butyl hydrogen phthalate $$ Phthalic acid, monobutyl ester $$ M

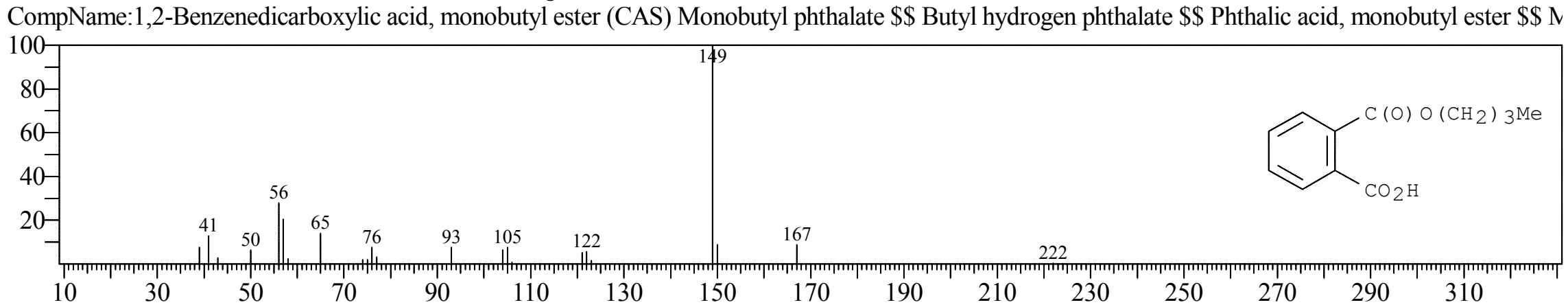

Hit#:5 Entry:58908 Library:NIST11.lib
SI:78 Formula:C12H14O4 CAS:33693-84-4 MolWeight:222 RetIndex:1744
CompName:t-Butyl hydrogen phthalate $$ tert-Butyl hydrogen phthalate $$ 2-(tert-Butoxycarbonyl)benzoic acid # $$

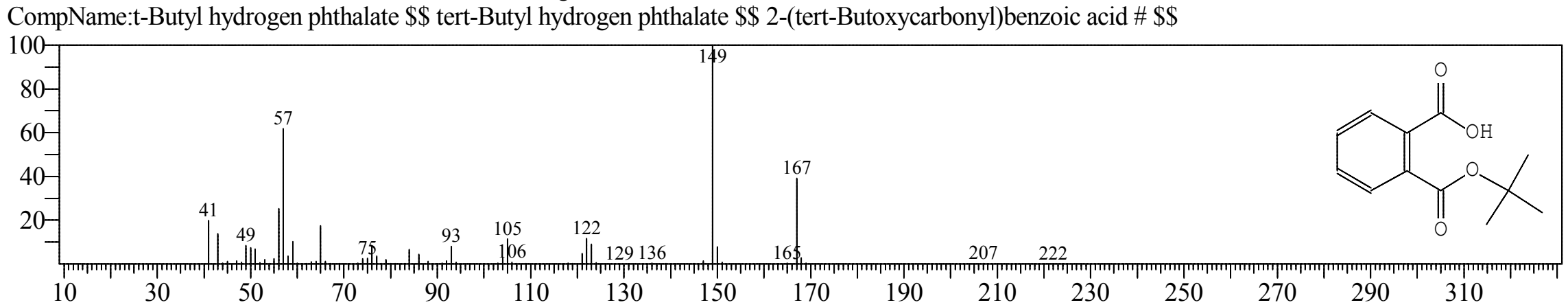

<< Target >>
Line#:5   R.Time:12.975(Scan#:958) MassPeaks:45
RawMode:Averaged 12.967-12.983(957-959) BasePeak:149.10(10464)
BG Mode:Calc. from Peak Group 1 - Event 1 Scan

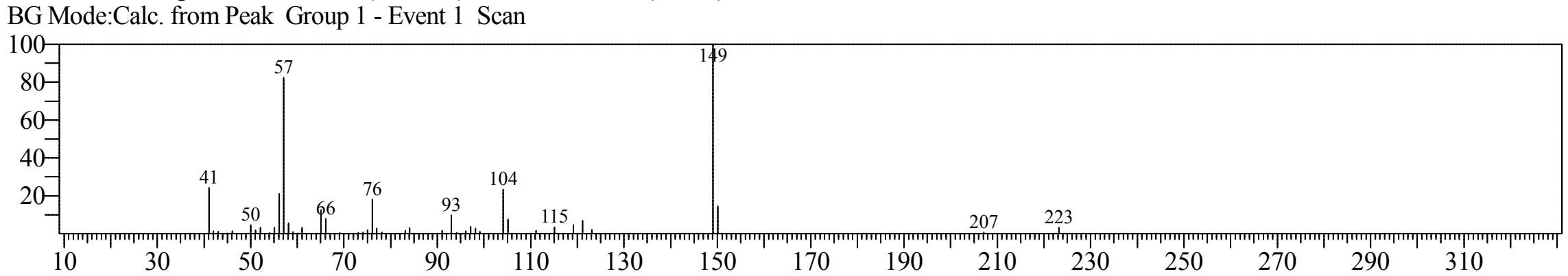

Hit#:6 Entry:79501 Library:NIST11.lib
SI:77 Formula:C14H18O4 CAS:0-00-0 MolWeight:250 RetIndex:2027
CompName:2-(Hexyloxycarbonyl)benzoic acid $$ Phthalic acid, monohexyl ester $$ Hexyl hydrogen phthalate $$ Monohexyl phthalate $$

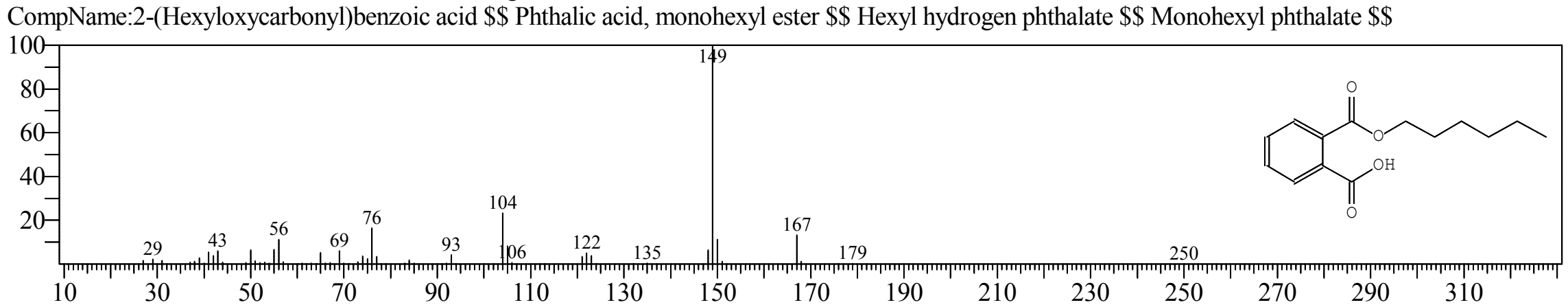

Hit#:7 Entry:134150 Library:NIST11.lib
SI:77 Formula:C19H28O4 CAS:0-00-0 MolWeight:320 RetIndex:2122
CompName:Phthalic acid, 4,4-dimethylpent-2-yl isobutyl ester

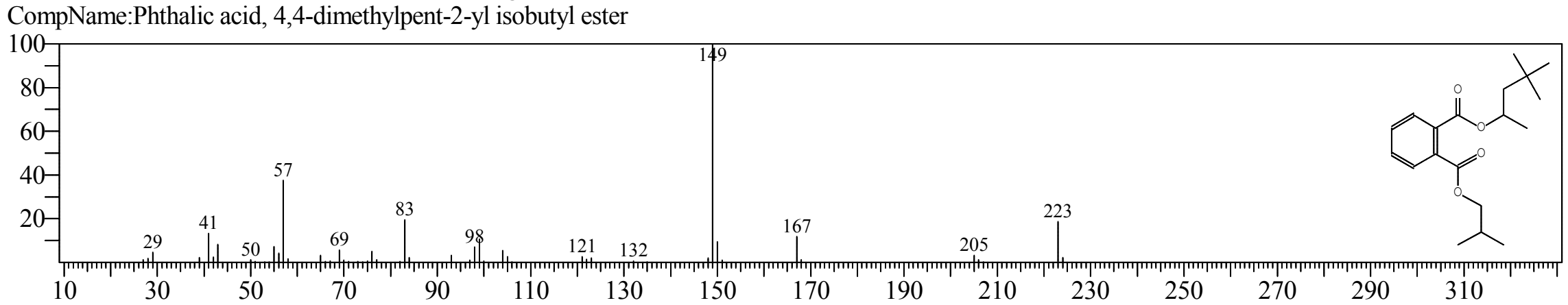

Hit#:8 Entry:25070 Library:NIST05s.LIB
SI:77 Formula:C20H30O4 CAS:85-69-8 MolWeight:334 RetIndex:2370
CompName:1,2-Benzenedicarboxylic acid, butyl 2-ethylhexyl ester $$ Phthalic acid, butyl 2-ethylhexyl ester $$ Butyl 2-ethylhexyl phthalate $$ 1-Butyl 2-(2

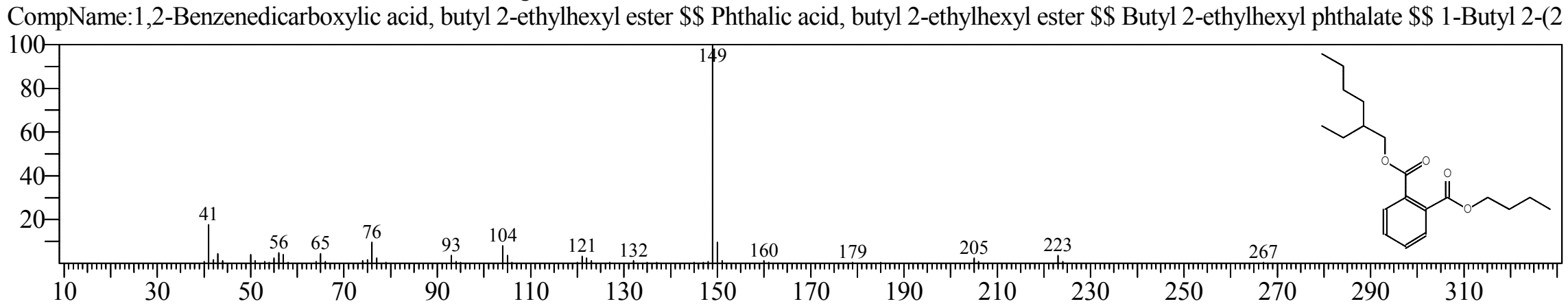

Hit#:9 Entry:58910 Library:NIST11.lib
SI:77 Formula:C12H14O4 CAS:0-00-0 MolWeight:222 RetIndex:1764
CompName:2-(Isobutoxycarbonyl)benzoic acid $$ Component at scan 1370 (14.624 min) [Model = +104u] in C:\\DATA\\11-15-09\\LMO_137_1.D\\DATA

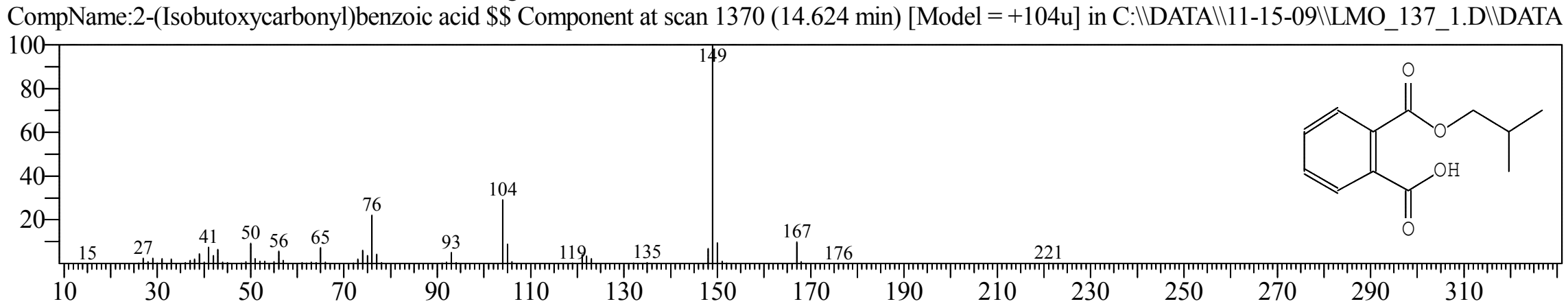

Hit#:10 Entry:144738 Library:NIST11.lib
SI:76 Formula:C20H30O4 CAS:0-00-0 MolWeight:334 RetIndex:2370
CompName:Phthalic acid, butyl oct-3-yl ester

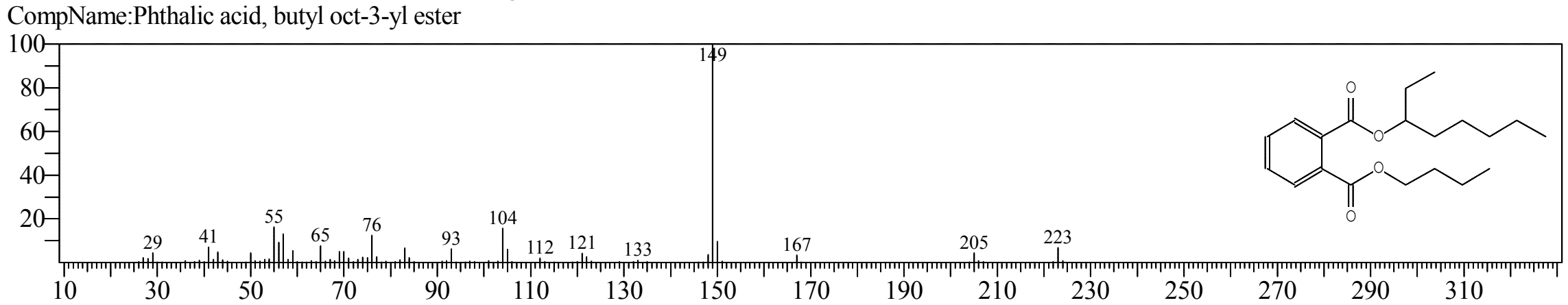

<< Target >>
Line#:5 R.Time:12.975(Scan#:958) MassPeaks:45
RawMode:Averaged 12.967-12.983(957-959) BasePeak:149.10(10464)
BG Mode:Calc. from Peak Group 1 - Event 1 Scan

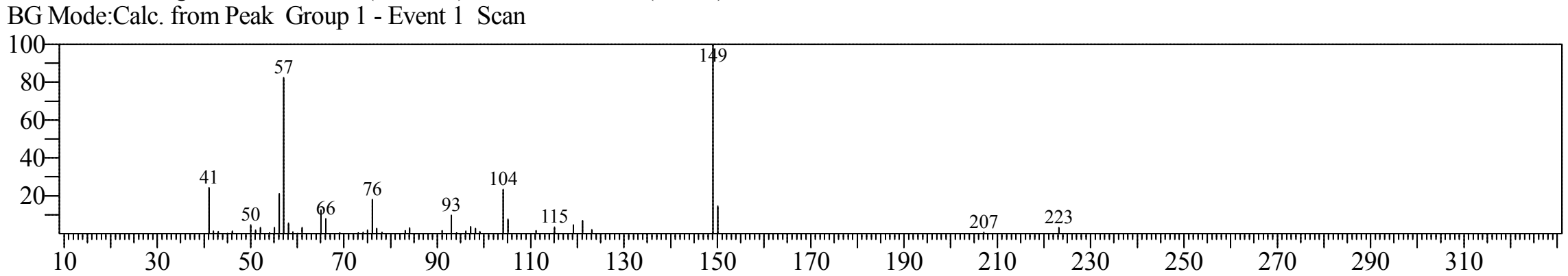

Hit#:11 Entry:25069 Library:NIST05s.LIB
SI:76 Formula:C20H30O4 CAS:84-78-6 MolWeight:334 RetIndex:2434
CompName:1,2-Benzenedicarboxylic acid, butyl octyl ester $$ Phthalic acid, butyl octyl ester $$ Butyl octyl phthalate $$ Octyl butyl phthalate $$ Plasticizer

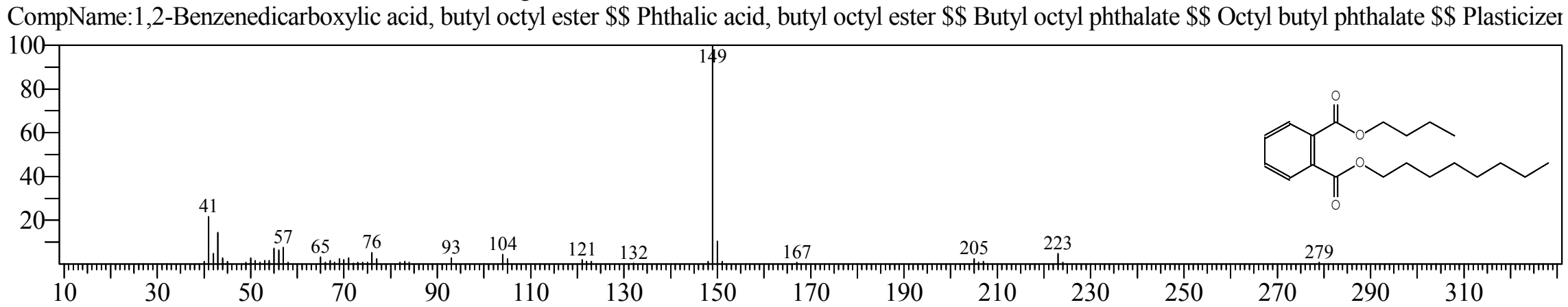

Hit#:12 Entry:232354 Library:WILEY7.LIB
SI:76 Formula:C18 H26 O5 CAS:33374-28-6 MolWeight:322 RetIndex:0
CompName:1,2-Benzenedicarboxylic acid, 2-butoxyethyl butyl ester (CAS) BUTYL BUTOXYETHYL PHTHALATE $$ BFBG $$ BUTYL PHTHALYL B

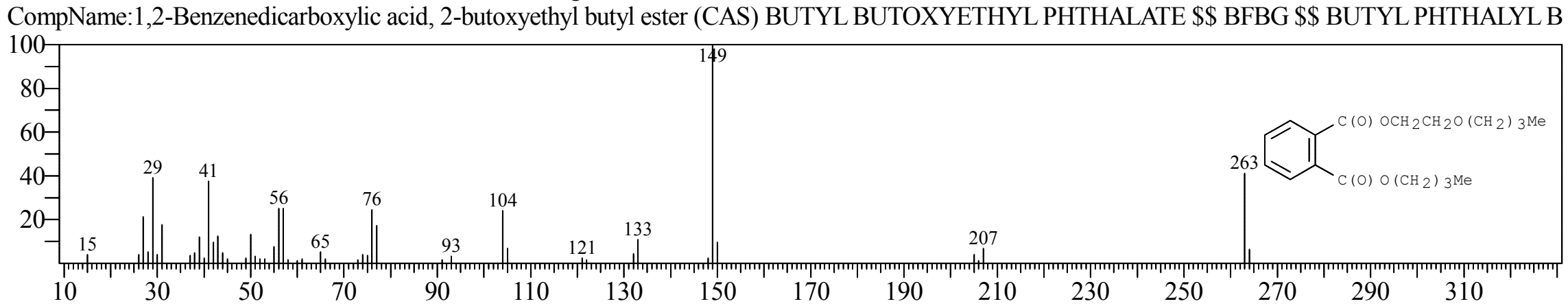

Hit#:13 Entry:25120 Library:NIST05s.LIB
SI:76 Formula:C18H24O6 CAS:85-70-1 MolWeight:336 RetIndex:2403
CompName:Phthalic acid, butyl ester, ester with butyl glycolate $$ Butyl carbobutoxymethyl phthalate $$ Butyl glycolyl butyl phthalate $$ Butyl phthalate b

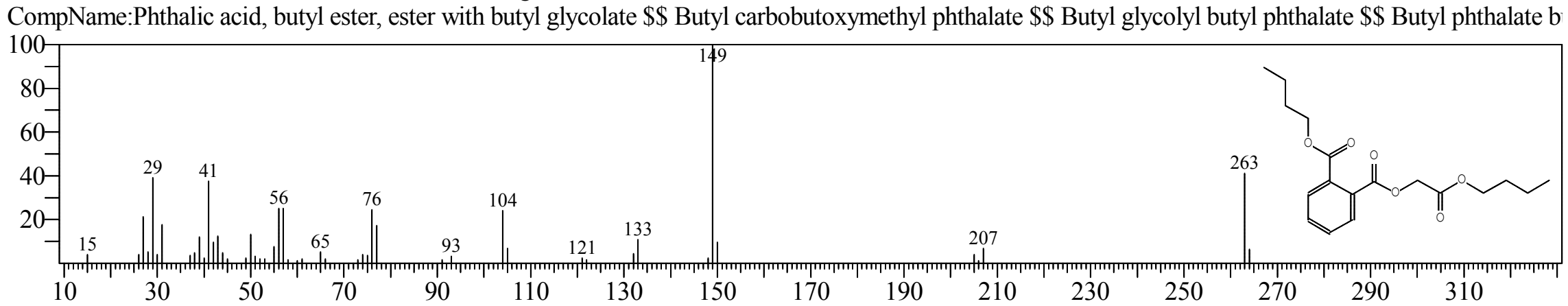

Hit#:14 Entry:71067 Library:NIST05.LIB
SI:76 Formula:C14H18O4 CAS:131-16-8 MolWeight:250 RetIndex:1838
CompName:1,2-Benzenedicarboxylic acid, dipropyl ester $$ Phthalic acid, dipropyl ester $$ Di-n-propyl phthalate $$ Dipropyl phthalate $$ Phthalic acid di-

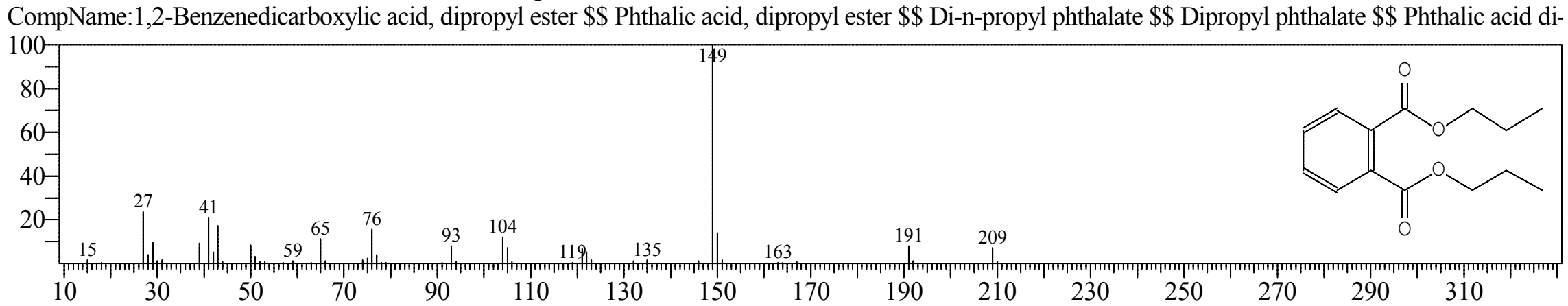

Hit#:15 Entry:57345 Library:NIST11.lib
SI:75 Formula:C12H12O4 CAS:0-00-0 MolWeight:220 RetIndex:1818
CompName:2-((But-3-enyloxy)carbonyl)benzoic acid $$ Phthalic acid, monobut-3-enyl ester $$ But-3-enyl hydrogen phthalate $$ Monobut-3-enyl phthalate

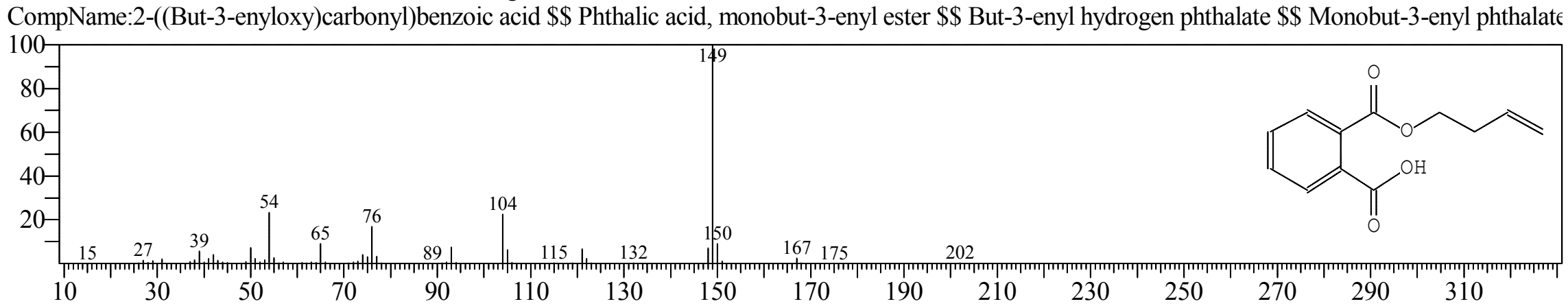

<< Target >>
Line#:5   R.Time:12.975(Scan#:958) MassPeaks:45
RawMode:Averaged 12.967-12.983(957-959) BasePeak:149.10(10464)
BG Mode:Calc. from Peak Group 1 - Event 1 Scan

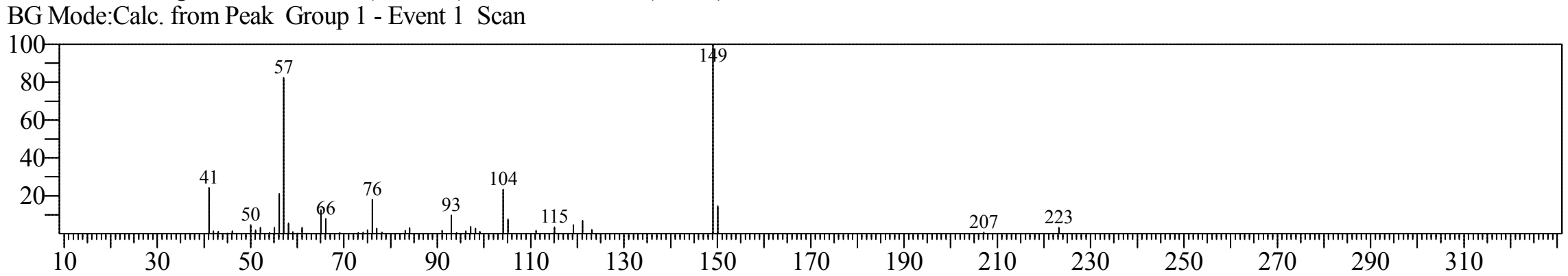

Hit#:16 Entry:146007 Library:NIST11.lib
SI:75 Formula:C19H28O5 CAS:0-00-0 MolWeight:336 RetIndex:2198
CompName:Phthalic acid, 1-tert-butoxyprop-2-yl isobutyl ester

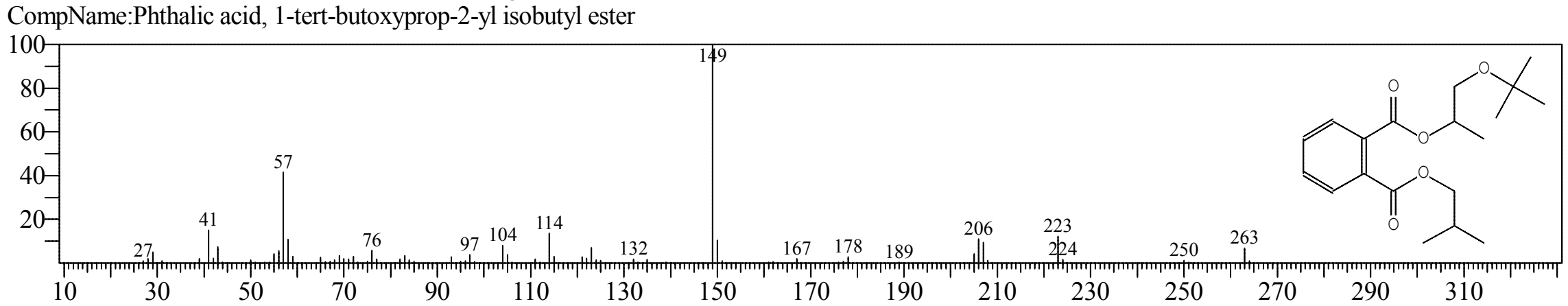

Hit#:17 Entry:134149 Library:NIST11.lib
SI:75 Formula:C19H28O4 CAS:0-00-0 MolWeight:320 RetIndex:2186
CompName:Phthalic acid, 4,4-dimethylpent-2-yl butyl ester

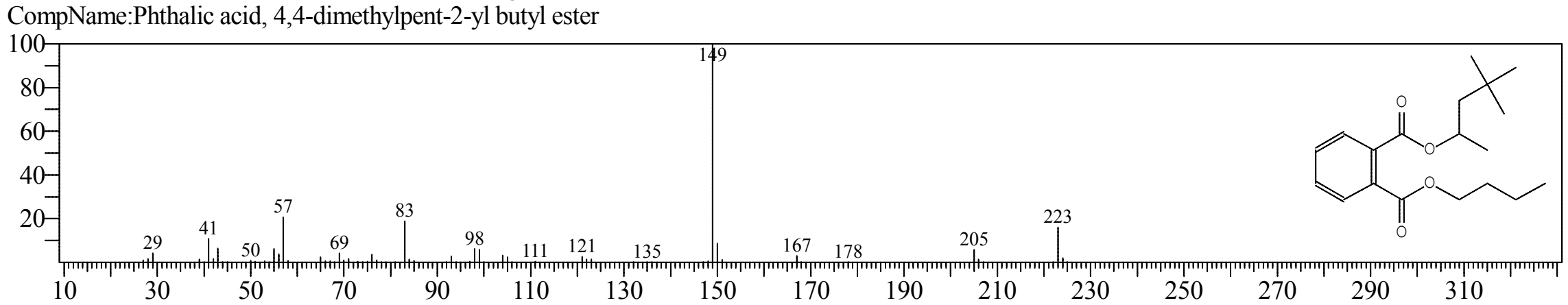

Hit#:18 Entry:137785 Library:NIST05.LIB
SI:75 Formula:C23H36O4 CAS:0-00-0 MolWeight:376 RetIndex:2732
CompName:Phthalic acid, butyl undecyl ester

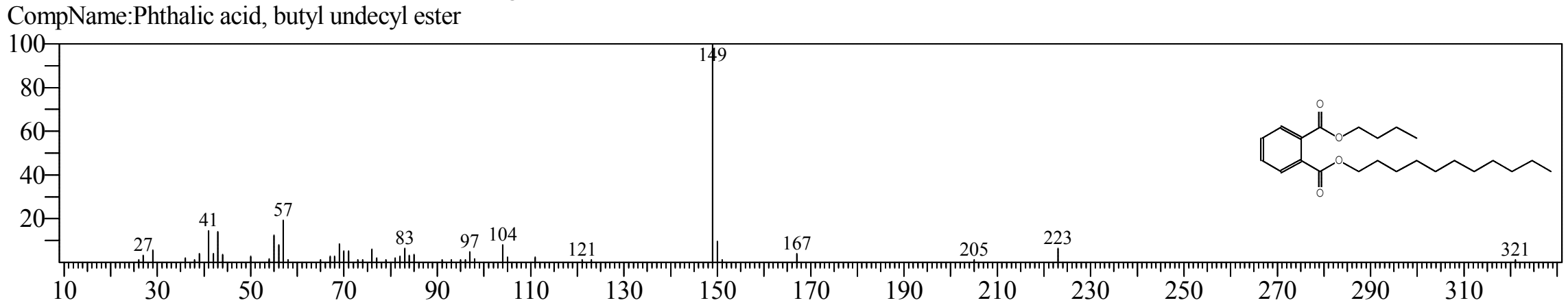

Hit#:19 Entry:171438 Library:NIST11.lib
SI:75 Formula:C23H36O4 CAS:0-00-0 MolWeight:376 RetIndex:2668
CompName:Phthalic acid, heptyl oct-3-yl ester

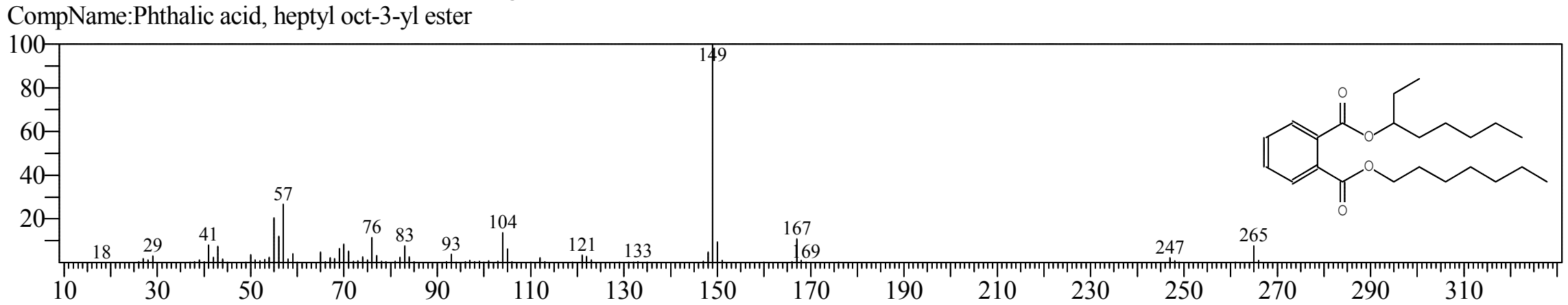

Hit#:20 Entry:171436 Library:NIST11.lib
SI:75 Formula:C23H36O4 CAS:0-00-0 MolWeight:376 RetIndex:2732
CompName:Phthalic acid, butyl undecyl ester

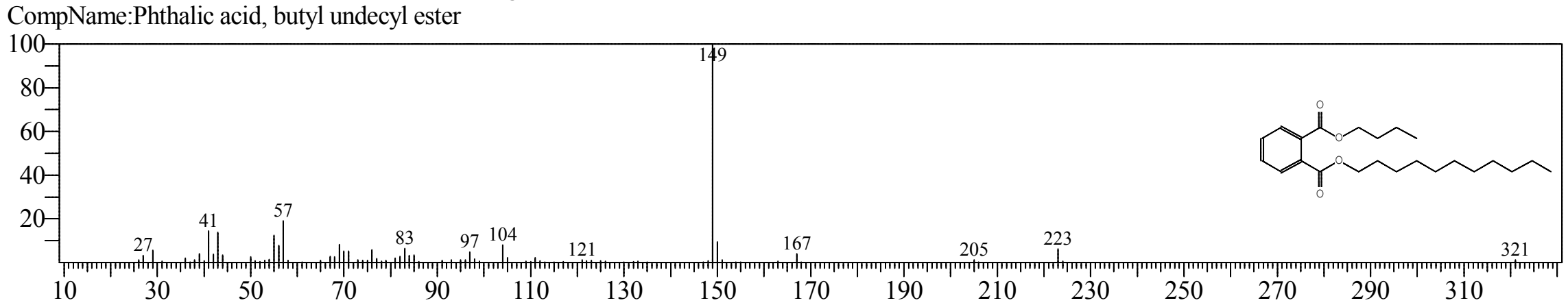

<< Target >>
Line#:6 R.Time:13.375(Scan#:1006) MassPeaks:50
RawMode:Averaged 13.367-13.383(1005-1007) BasePeak:74.05(1737)
BG Mode:Calc. from Peak Group 1 - Event 1 Scan

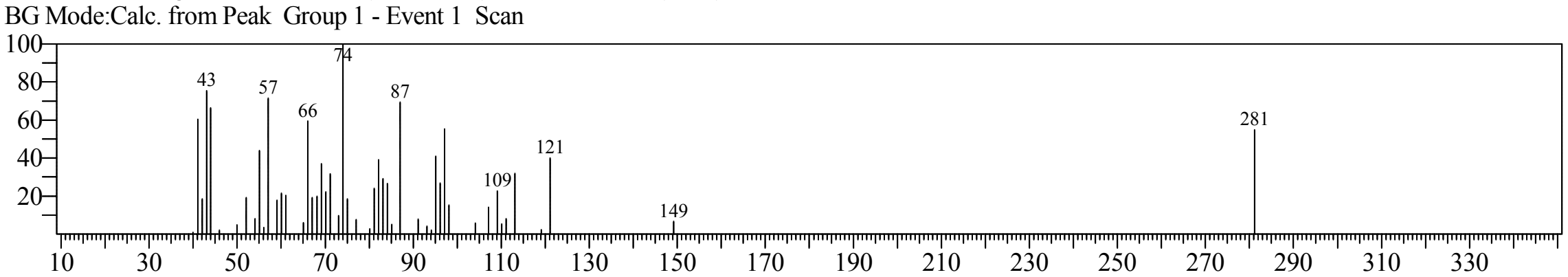

Hit#:1 Entry:107959 Library:NIST05.LIB
SI:73 Formula:C19H36O3 CAS:38520-31-9 MolWeight:312 RetIndex:2129
CompName:Oxiraneundecanoic acid, 3-pentyl-, methyl ester, trans- $$ Methyl 11-(3-pentyl-2-oxiranyl)undecanoate # $$

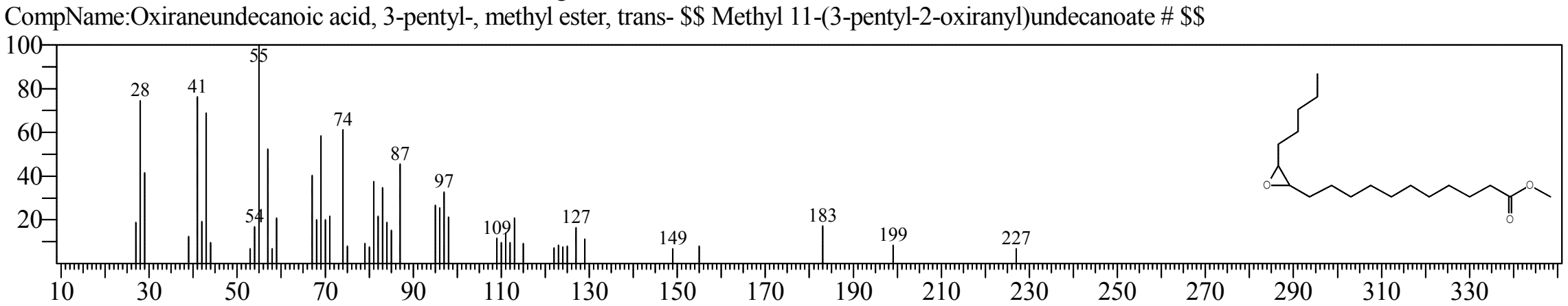

Hit#:2 Entry:109077 Library:NIST05.LIB
SI:73 Formula:C19H38O3 CAS:2420-36-2 MolWeight:314 RetIndex:2239
CompName:Octadecanoic acid, 3-hydroxy-, methyl ester $$ Methyl 3-hydroxyoctadecanoate $$

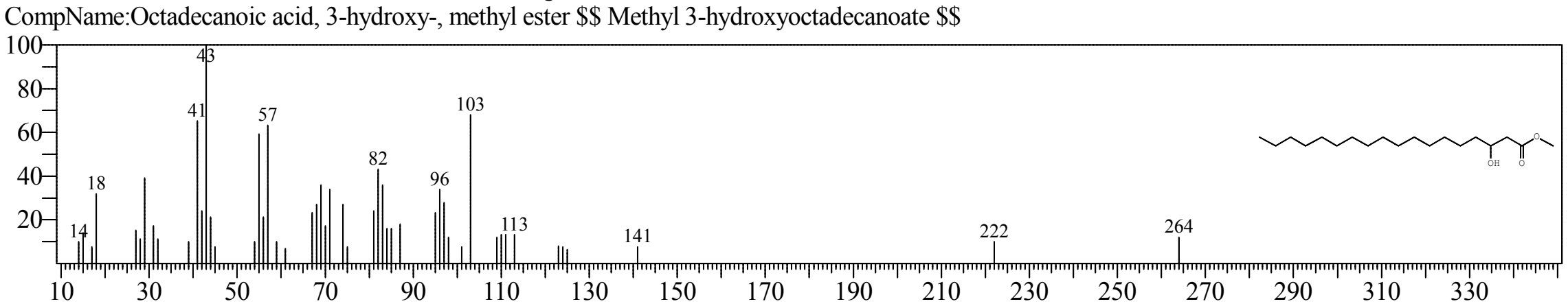

Hit#:3 Entry:64005 Library:NIST05.LIB
SI:73 Formula:C15H26O2 CAS:56909-03-6 MolWeight:238 RetIndex:1677
CompName:13-Tetradecynoic acid, methyl ester $$ Methyl 13-tetradecynoate # $$

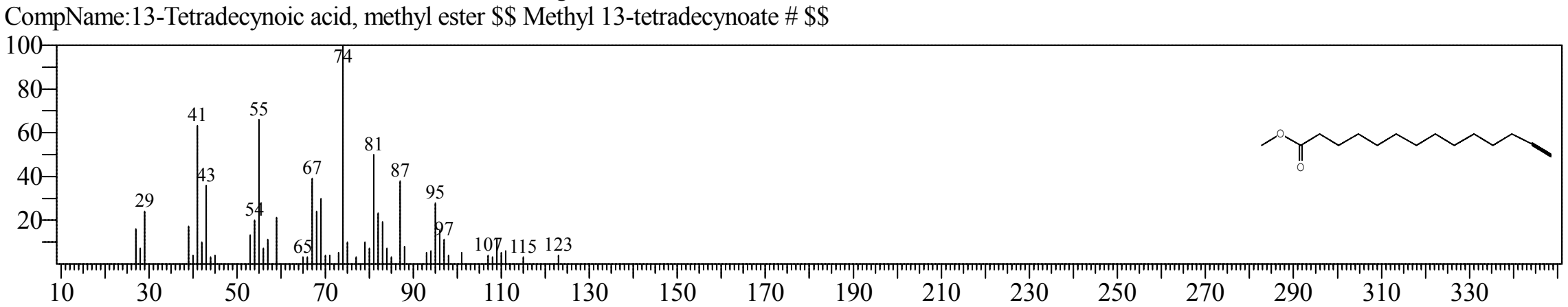

Hit#:4 Entry:234093 Library:WILEY7.LIB
SI:73 Formula:C21 H40 O2 CAS:27070-40-2 MolWeight:324 RetIndex:0
CompName:Eicosenoic acid, methyl ester (CAS) METHYL EICOSENOATE $$

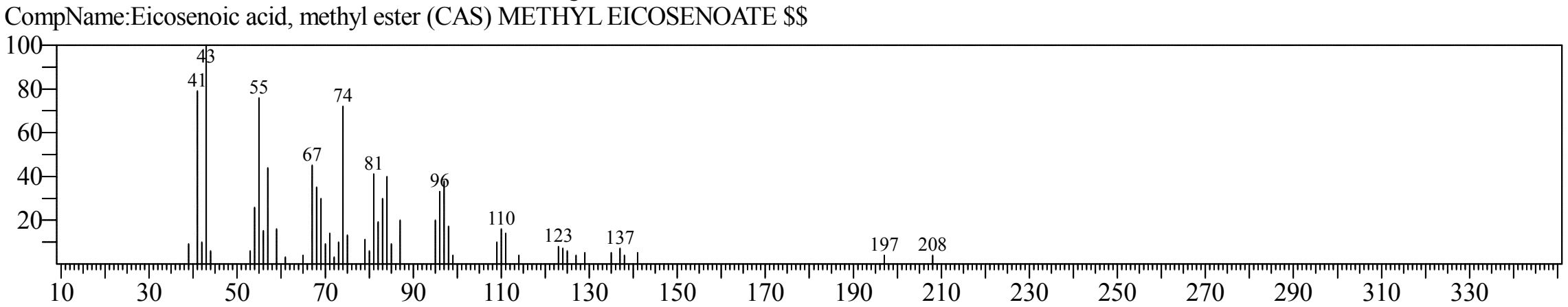

Hit#:5 Entry:25263 Library:NIST05s.LIB
SI:72 Formula:C22H44O2 CAS:6064-90-0 MolWeight:340 RetIndex:2375
CompName:Heneicosanoic acid, methyl ester $$ Methyl heneicosanoate $$ Methyl henelcosanoate $$ Methyl henicosanoate # $$

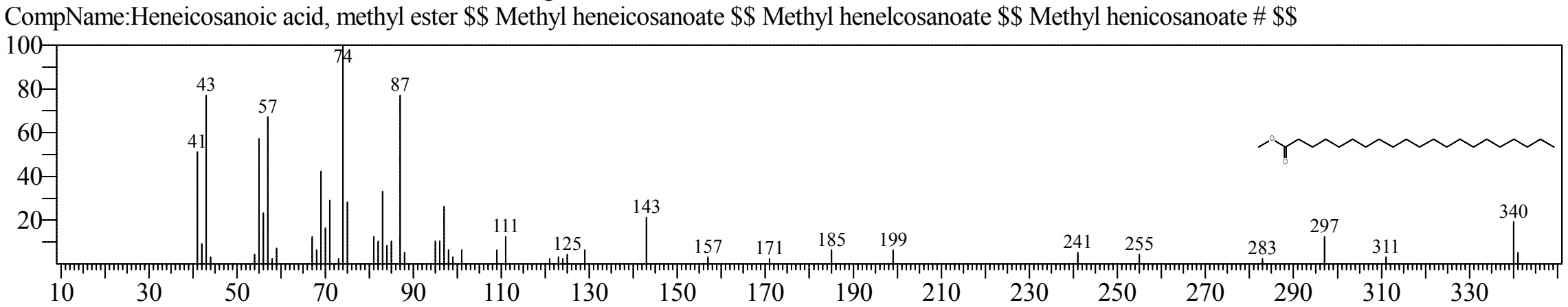

<< Target >>
Line#:6 R.Time:13.375(Scan#:1006) MassPeaks:50
RawMode:Averaged 13.367-13.383(1005-1007) BasePeak:74.05(1737)
BG Mode:Calc. from Peak Group 1 - Event 1 Scan

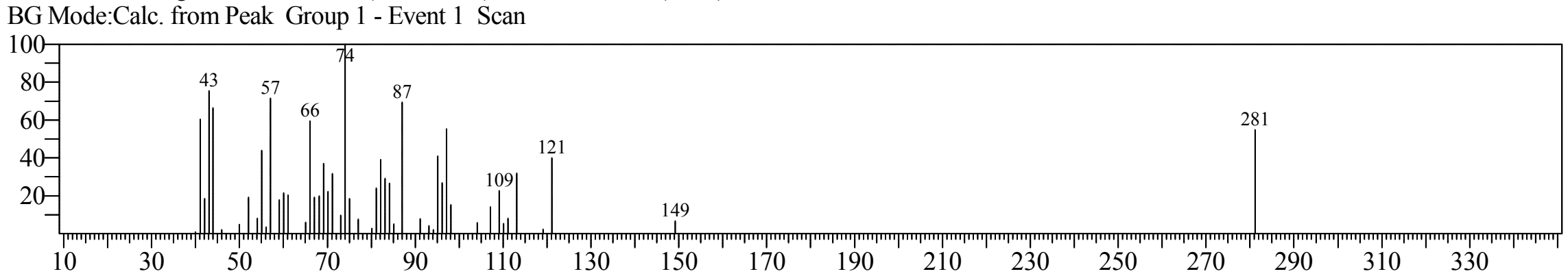

Hit#:6 Entry:113954 Library:NIST11.lib
SI:72 Formula:C19H34O2 CAS:0-00-0 MolWeight:294 RetIndex:2075
CompName:17-Octadecynoic acid, methyl ester

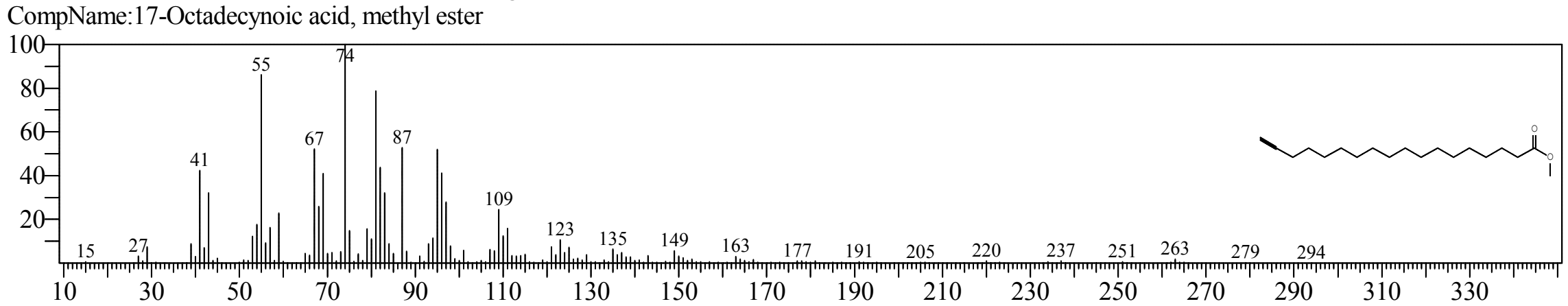

Hit#:7 Entry:207873 Library:WILEY7.LIB
SI:72 Formula:C19 H36 O2 CAS:1937-62-8 MolWeight:296 RetIndex:0
CompName:9-Octadecenoic acid, methyl ester, (E)- (CAS) Methyl elaidate $$ METHYL-TRANS 9-OCTADECENOATE $$ Elaidic acid methyl ester $$ El

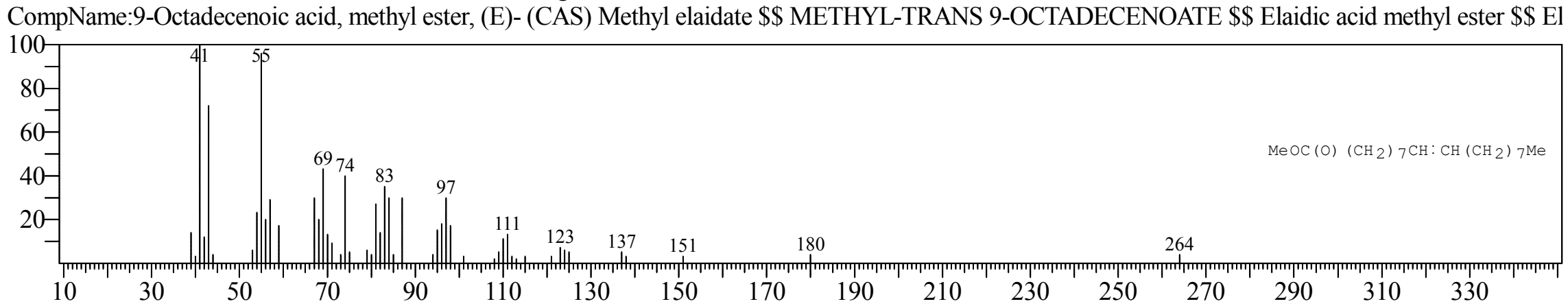

Hit#:8 Entry:337808 Library:WILEY7.LIB
SI:72 Formula:C70 H140 O2 CAS:40710-36-9 MolWeight:1013 RetIndex:0
CompName:Nonahexacontanoic acid, methyl ester (CAS) METHYL N-NONAHEXACONTANOATE $$ Methyl nonahexacontanoate $$

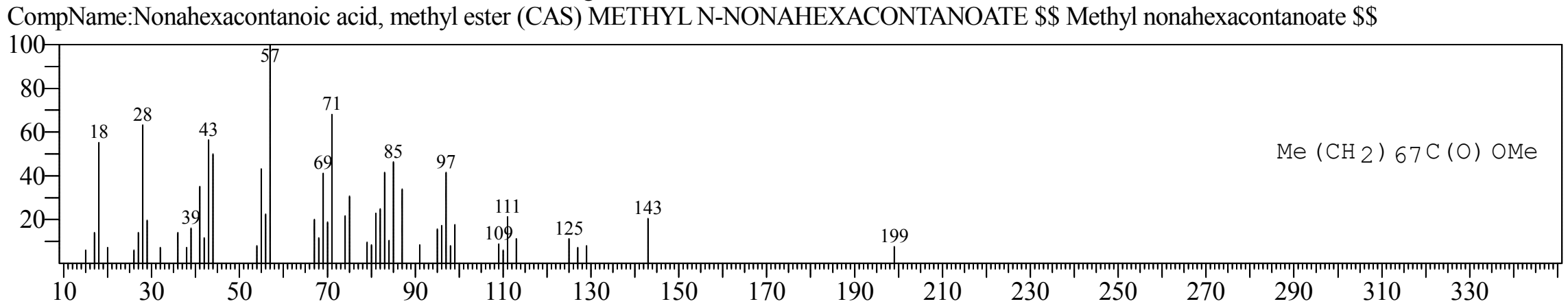

Hit#:9 Entry:112483 Library:NIST05.LIB
SI:72 Formula:C21H36O2 CAS:17364-32-8 MolWeight:320 RetIndex:2300
CompName:8,11,14-Eicosatrienoic acid, methyl ester $$ Methyl (8E,11E,14E)-8,11,14-icosatrienoate # $$

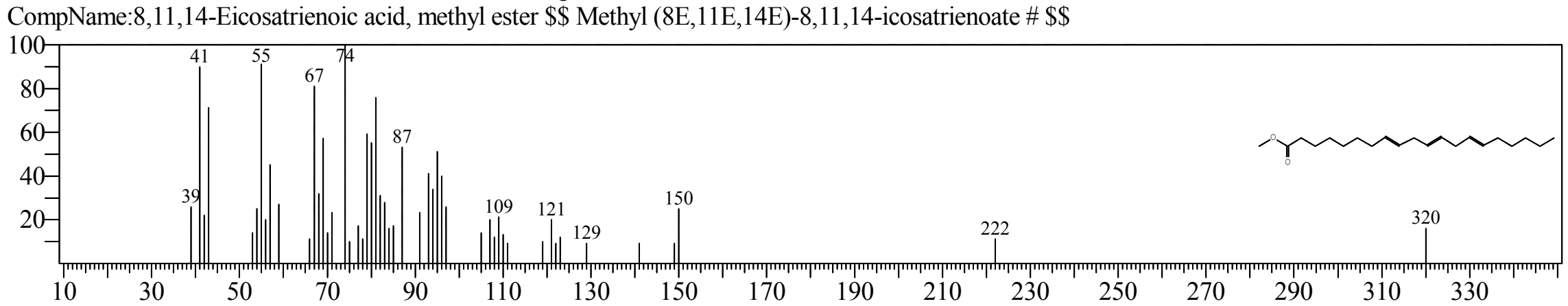

Hit#:10 Entry:111365 Library:WILEY7.LIB
SI:72 Formula:C14 H28 O CAS:124-25-4 MolWeight:212 RetIndex:0
CompName:Tetradecanal (CAS) Myristaldehyde $$ Myristylaldehyde $$ Tetradecylaldehyde $$ n-Tetradecanal $$ Aldehyde C-14 $$ Aldehyde C-14, myris

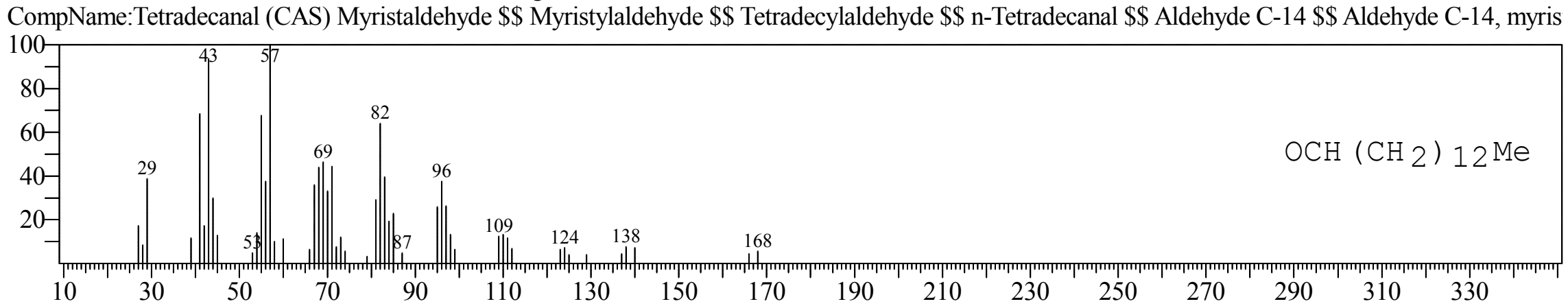

<< Target >>
Line#:6 R.Time:13.375(Scan#:1006) MassPeaks:50
RawMode:Averaged 13.367-13.383(1005-1007) BasePeak:74.05(1737)
BG Mode:Calc. from Peak Group 1 - Event 1 Scan

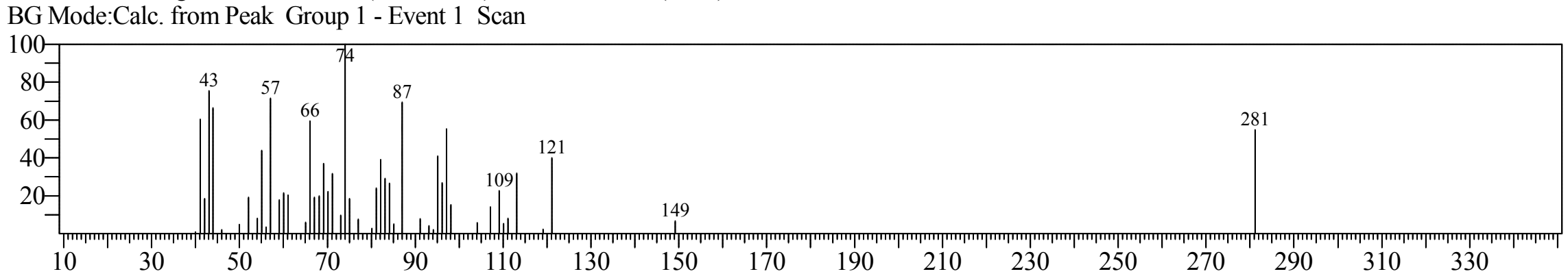

Hit#:11 Entry:26165 Library:NIST05s.LIB
SI:72 Formula:C25H48O2 CAS:2733-88-2 MolWeight:380 RetIndex:2682
CompName:15-Tetracosenoic acid, methyl ester, (Z)- $$ Methyl nervonate $$ Methyl (15Z)-15-tetracosenoate # $$

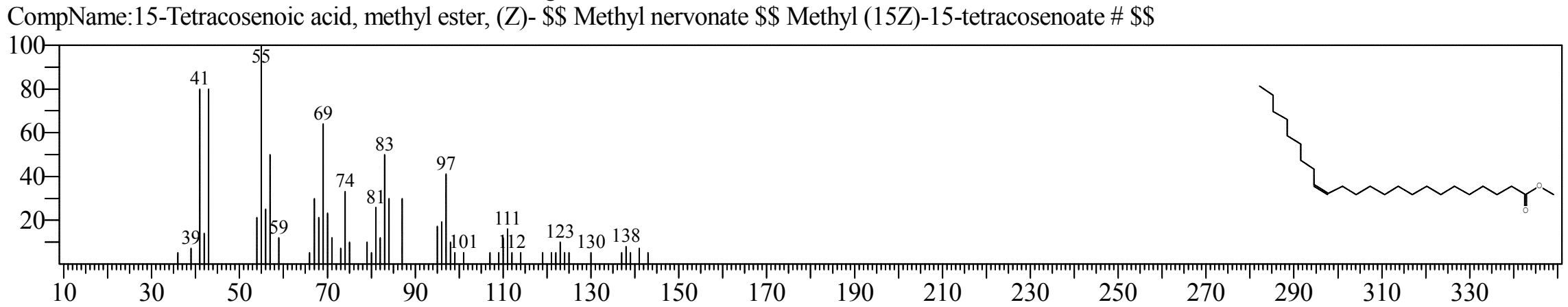

Hit#:12 Entry:82142 Library:NIST05.LIB
SI:72 Formula:C17H32O2 CAS:2825-81-2 MolWeight:268 RetIndex:1886
CompName:2-Hexadecenoic acid, methyl ester, (E)- $$ Methyl (2E)-2-hexadecenoate # $$

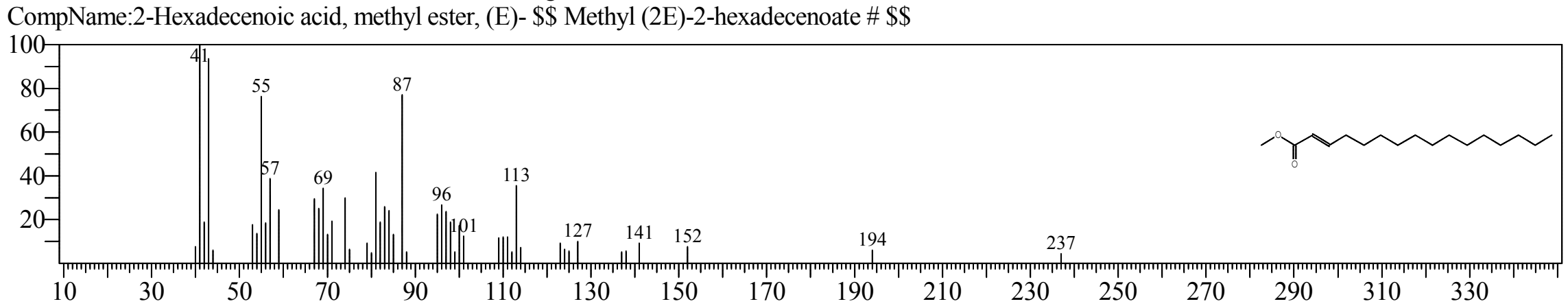

Hit#:13 Entry:137112 Library:NIST05.LIB
SI:72 Formula:C25H42O2 CAS:56051-53-7 MolWeight:374 RetIndex:2528
CompName:Cyclopropanebutanoic acid, 2-[[2-[[2-[(2-pentylcyclopropyl)methyl]cyclopropyl]methyl]cyclopropyl]methyl]-, methyl ester $$ Methyl 4-(2-([2-

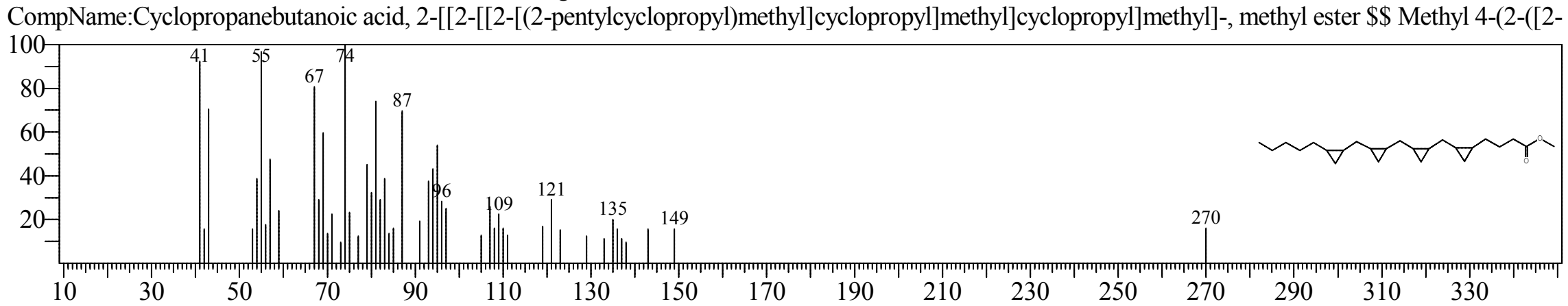

Hit#:14 Entry:20363 Library:NIST05s.LIB
SI:71 Formula:C15H30O2 CAS:1002-84-2 MolWeight:242 RetIndex:1869
CompName:Pentadecanoic acid $$ Pentadecylic acid $$ n-Pentadecanoic acid $$ n-Pentadecylic acid $$

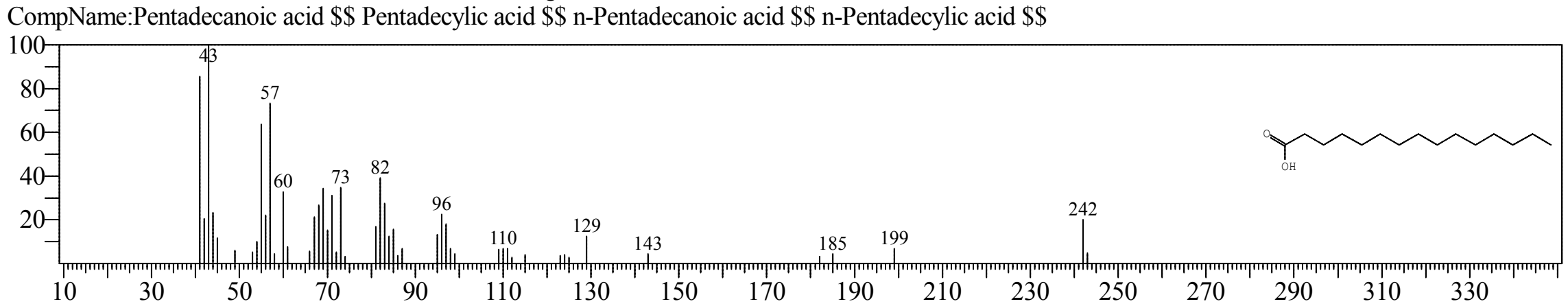

Hit#:15 Entry:60579 Library:WILEY7.LIB
SI:71 Formula:C10 H16 D2 O2 CAS:20731-26-4 MolWeight:170 RetIndex:0
CompName:METHYL-4,5-DIDEUTERIO-4 NONENOATE $$ 4-Nonenoic-4,5-d2 acid, methyl ester (CAS)

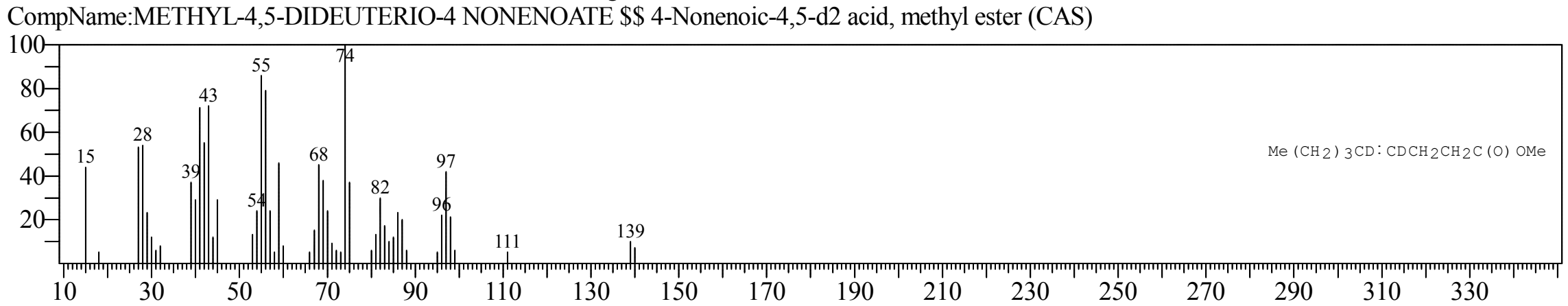

<< Target >>
Line#:6 R.Time:13.375(Scan#:1006) MassPeaks:50
RawMode:Averaged 13.367-13.383(1005-1007) BasePeak:74.05(1737)
BG Mode:Calc. from Peak Group 1 - Event 1 Scan

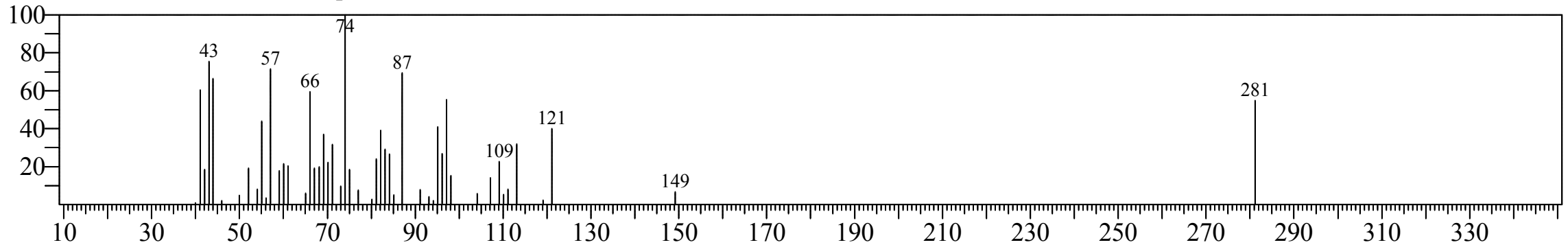

Hit#:16 Entry:107960 Library:NIST05.LIB
SI:71 Formula:C19H36O3 CAS:38520-30-8 MolWeight:312 RetIndex:2129
CompName:Oxiraneundecanoic acid, 3-pentyl-, methyl ester, cis- $$ Methyl 11-(3-pentyl-2-oxiranyl)undecanoate # $$

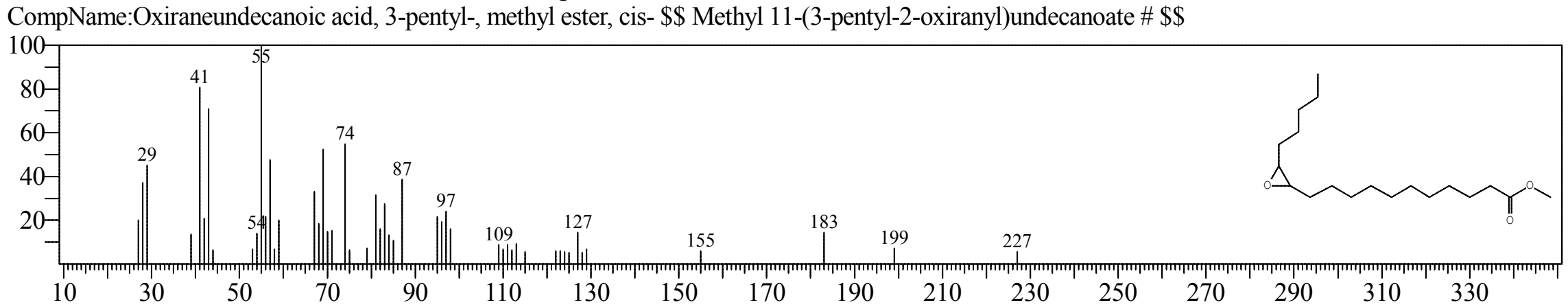

Hit#:17 Entry:82070 Library:NIST05.LIB
SI:71 Formula:C16H28O3 CAS:0-00-0 MolWeight:268 RetIndex:1877
CompName:Z-(13,14-Epoxy)tetradec-11-en-1-ol acetate

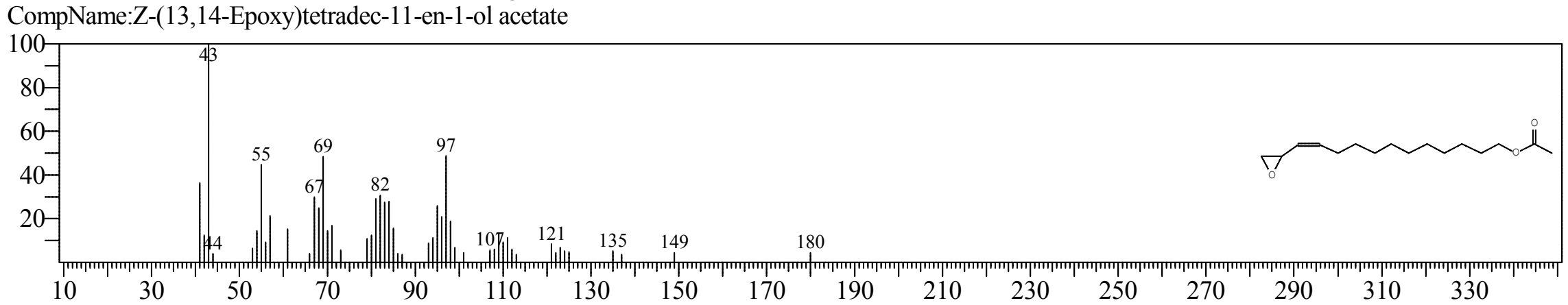

Hit#:18 Entry:93374 Library:NIST11.lib
SI:71 Formula:C16H28O3 CAS:0-00-0 MolWeight:268 RetIndex:1877
CompName:Z-(13,14-Epoxy)tetradec-11-en-1-ol acetate $$ (11Z)-12-(2-Oxiranyl)-11-dodecenyl acetate # $$

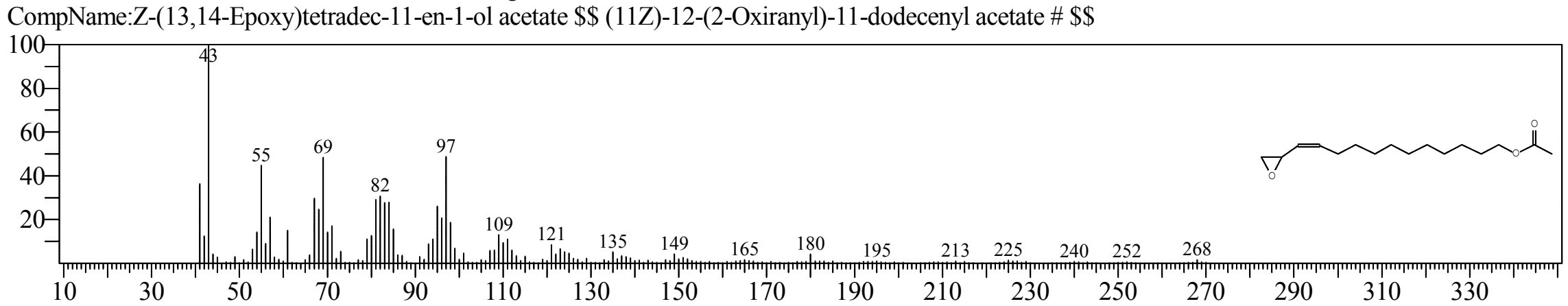

Hit#:19 Entry:95351 Library:NIST05.LIB
SI:71 Formula:C19H30O2 CAS:56846-98-1 MolWeight:290 RetIndex:2112
CompName:13,16-Octadecadiynoic acid, methyl ester $$ Methyl 13,16-octadecadiynoate # $$

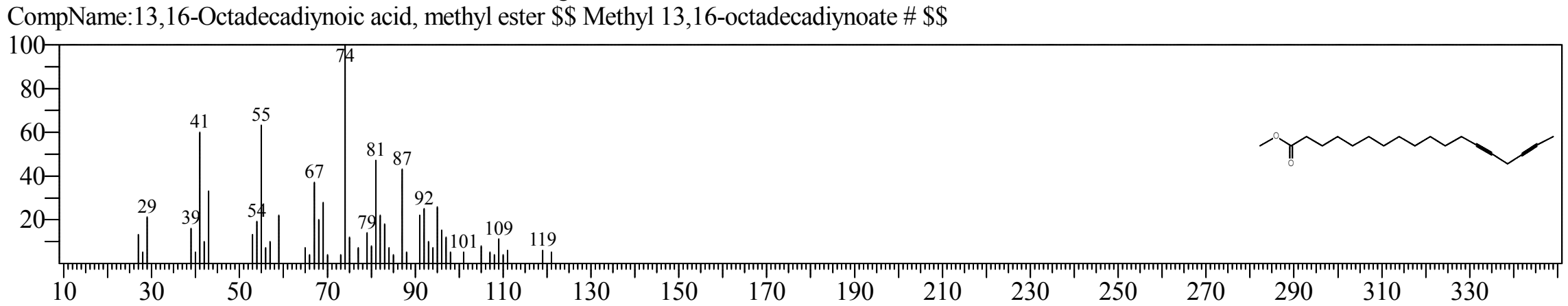

Hit#:20 Entry:93487 Library:NIST11.lib
SI:71 Formula:C17H32O2 CAS:0-00-0 MolWeight:268 RetIndex:1822
CompName:10-Methyl-E-11-tridecen-1-ol propionate $$ (11E)-10-Methyl-11-tridecenyl propionate # $$

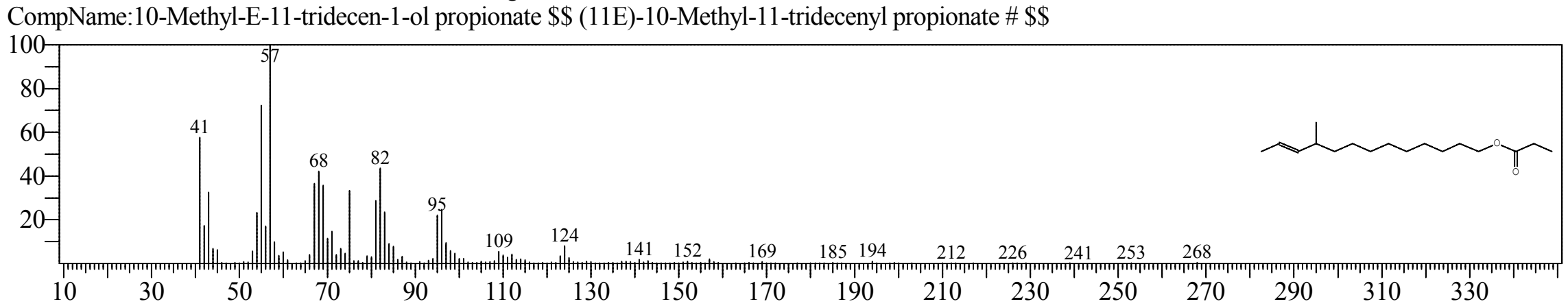

<< Target >>
Line#:7 R.Time:13.700(Scan#:1045) MassPeaks:47
RawMode:Averaged 13.692-13.708(1044-1046) BasePeak:73.05(6485)
BG Mode:Calc. from Peak Group 1 - Event 1 Scan

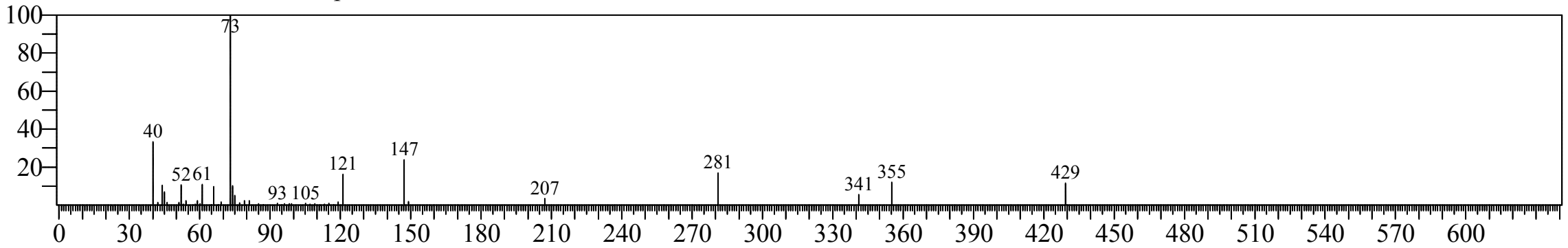

Hit#:1 Entry:306966 Library:WILEY7.LIB
SI:72 Formula:C20 H42 O4 SI4 CAS:56114-62-6 MolWeight:458 RetIndex:0
CompName:Silane, [[4-[1,2-bis[(trimethylsilyl)oxy]ethyl]-1,2-phenylene]bis(oxy)]bis[trimethyl- (CAS) 3,4-DIHYDROXYPHENYLGLYCOL-TETRATMS

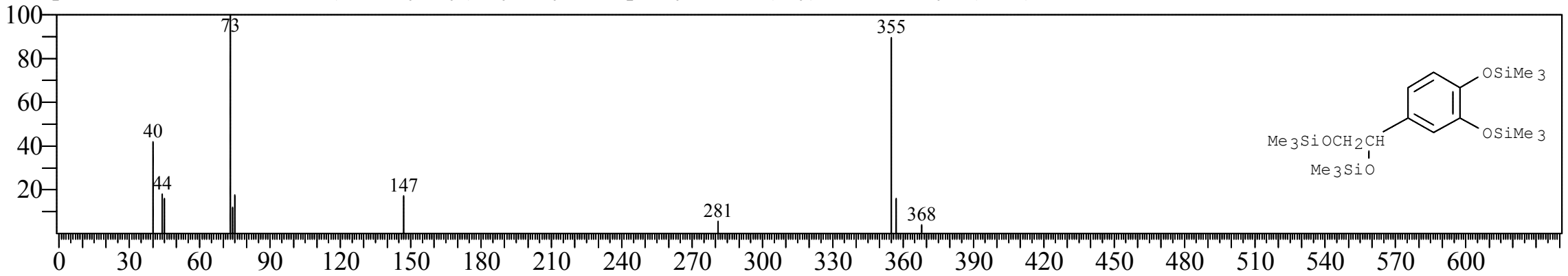

Hit#:2 Entry:161854 Library:NIST05.LIB
SI:71 Formula:C16H48O10Si9 CAS:145344-72-5 MolWeight:652 RetIndex:1716
CompName:2-(2',4',4',6',6',8',8'-Heptamethyltetrasiloxan-2'-yloxy)-2,4,4,6,6,8,8,10,10-nonamethylcyclopentasiloxane $$ 2-[(2,4,4,6,6,8,8-Heptamethyl-1,3,5

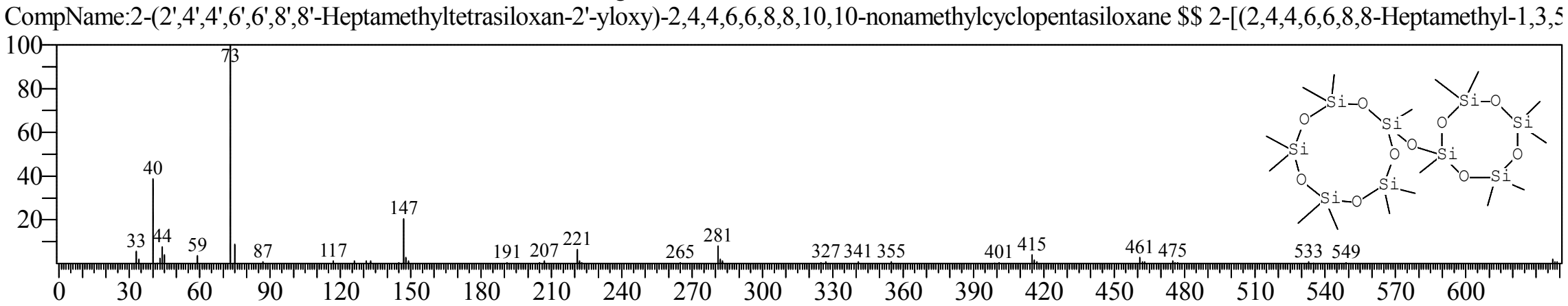

Hit#:3 Entry:138359 Library:WILEY7.LIB
SI:70 Formula:C8 H18 O4 SI2 CAS:18294-04-7 MolWeight:234 RetIndex:0
CompName:Ethanedioic acid, bis(trimethylsilyl) ester (CAS) OXALIC ACID-DITMS $$ BIS(TRIMETHYLSILYL) OXALATE $$ BISTRIMETHYLSILYI

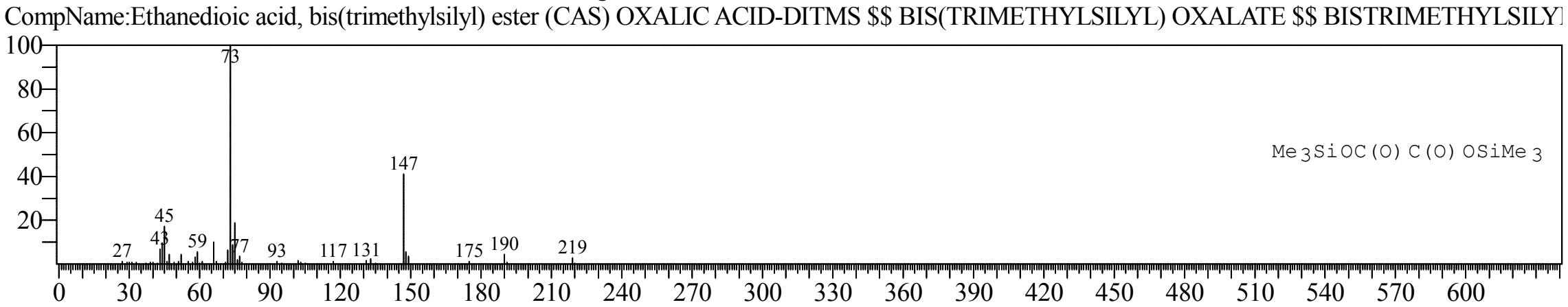

Hit#:4 Entry:26522 Library:NIST05s.LIB
SI:65 Formula:C18H37NO3Si3 CAS:10538-85-9 MolWeight:399 RetIndex:2007
CompName:Phenethylamine, N-methyl-.beta.,3,4-tris(trimethylsiloxy)- $$ 2-(3,4-Bis[(trimethylsilyl)oxy]phenyl)-N-methyl-2-[(trimethylsilyl)oxy]ethanamine

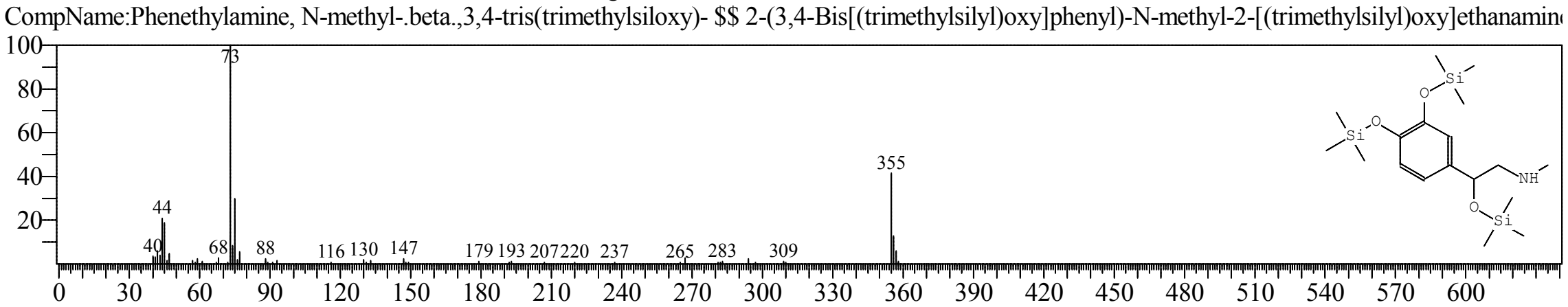

Hit#:5 Entry:156094 Library:WILEY7.LIB
SI:65 Formula:C10 H26 O3 SI2 CAS:0-00-0 MolWeight:250 RetIndex:0
CompName:DIETHYLENE GLYCOL, DI-TMS $$

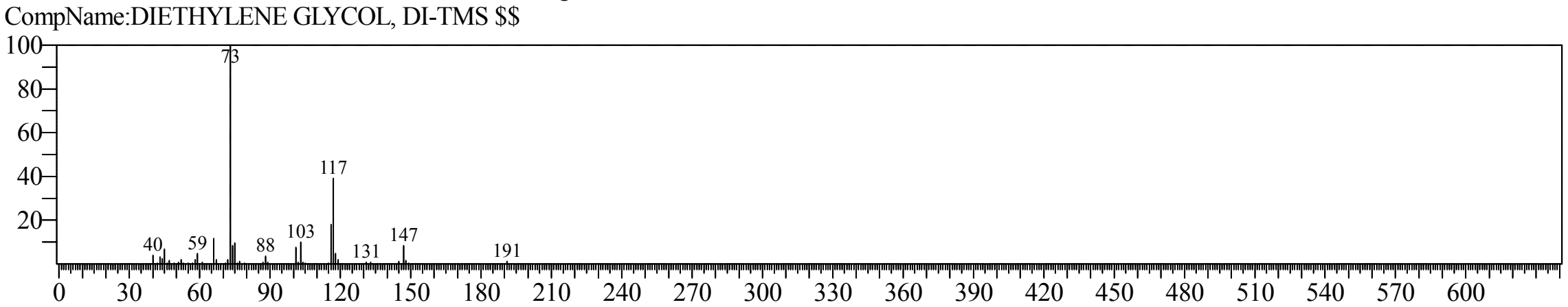

<< Target >>
Line#:7 R.Time:13.700(Scan#:1045) MassPeaks:47
RawMode:Averaged 13.692-13.708(1044-1046) BasePeak:73.05(6485)
BG Mode:Calc. from Peak Group 1 - Event 1 Scan

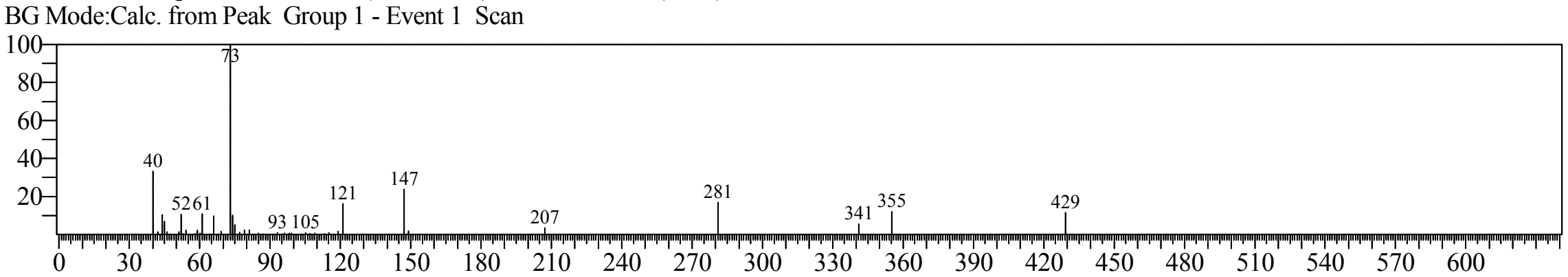

Hit#:6 Entry:162181 Library:WILEY7.LIB
SI:64 Formula:C12 H22 O2 SI2 CAS:5075-52-5 MolWeight:254 RetIndex:0
CompName:Silane, [1,2-phenylenebis(oxy)]bis[trimethyl- (CAS) BISTRIMETHYLSILYL CATECHOL $$ CATECHOL TRIMETHYLSILYL DERIVATIV

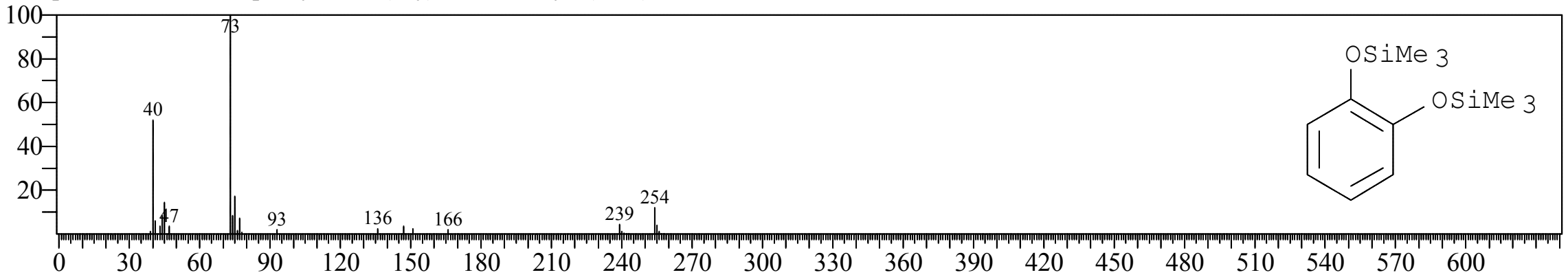

Hit#:7 Entry:102985 Library:NIST05.LIB
SI:64 Formula:C13H28O4Si2 CAS:90744-45-9 MolWeight:304 RetIndex:1468
CompName:Malonic acid, bis(2-trimethylsilylethyl ester $$ Bis[2-(trimethylsilyl)ethyl] malonate # $$

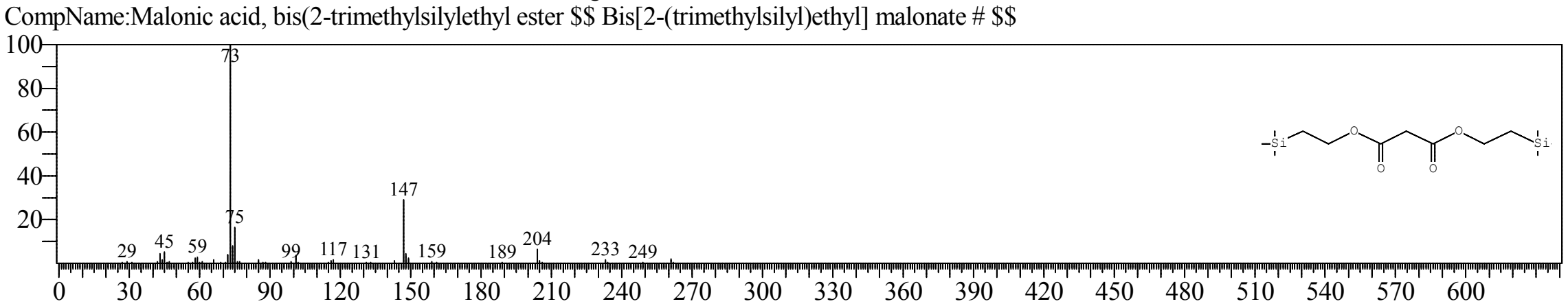

Hit#:8 Entry:72302 Library:NIST05.LIB
SI:64 Formula:C14H28Si2 CAS:0-00-0 MolWeight:252 RetIndex:1193
CompName:1,5-Heptadiene, 1,5-bis(trimethylsilyl-3-methylene-, 1E,5Z-

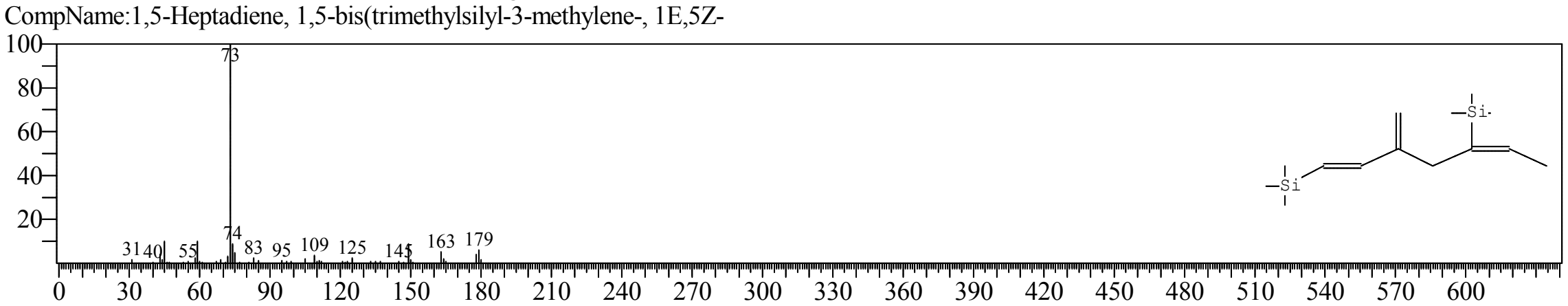

Hit#:9 Entry:81081 Library:WILEY7.LIB
SI:64 Formula:C8 H16 O3 SI CAS:74367-70-7 MolWeight:188 RetIndex:0
CompName:Butanoic acid, 3-methyl-2-oxo-, trimethylsilyl ester (CAS)

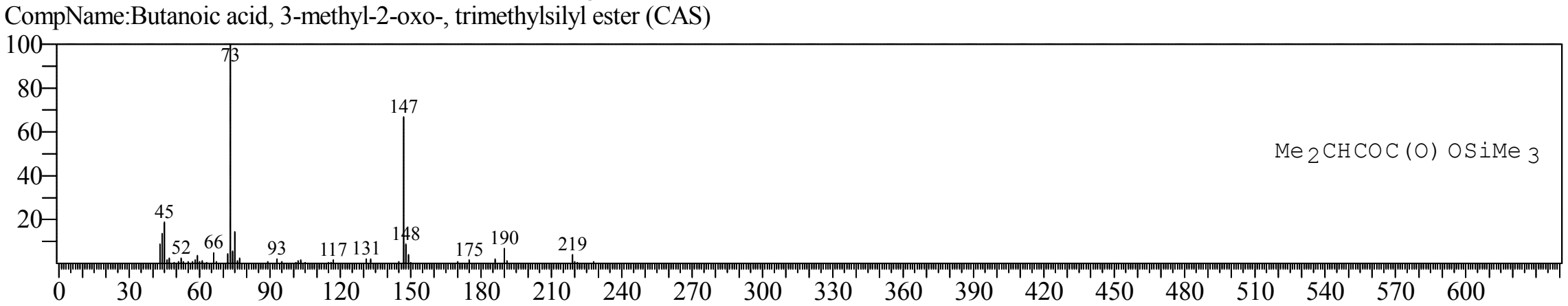

Hit#:10 Entry:18350 Library:NIST05s.LIB
SI:64 Formula:C8H20O3Si2 CAS:33581-77-0 MolWeight:220 RetIndex:880
CompName:Acetic acid, [(trimethylsilyl)oxy]-, trimethylsilyl ester $$ Acetic acid, (trimethylsiloxy)-, trimethylsilyl ester $$ Trimethylsilyl (trimethylsilyloxy)a

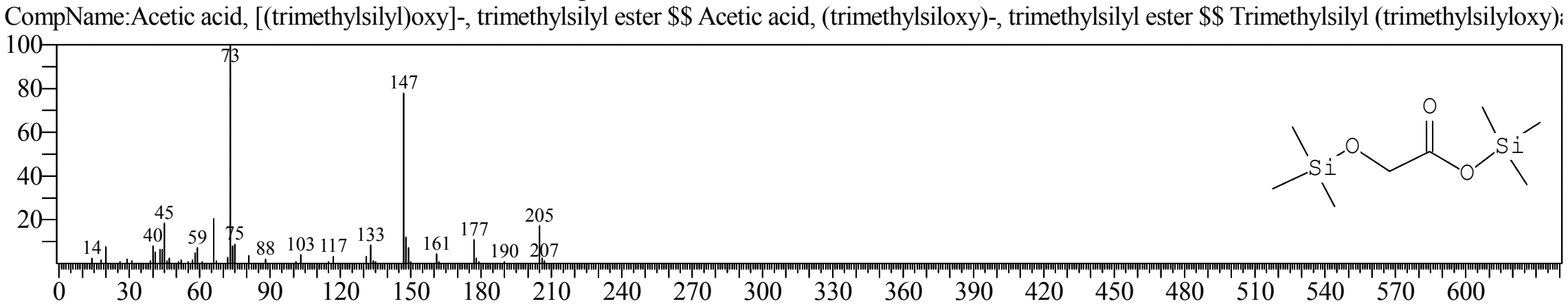

<< Target >>
Line#:7 R.Time:13.700(Scan#:1045) MassPeaks:47
RawMode:Averaged 13.692-13.708(1044-1046) BasePeak:73.05(6485)
BG Mode:Calc. from Peak Group 1 - Event 1 Scan

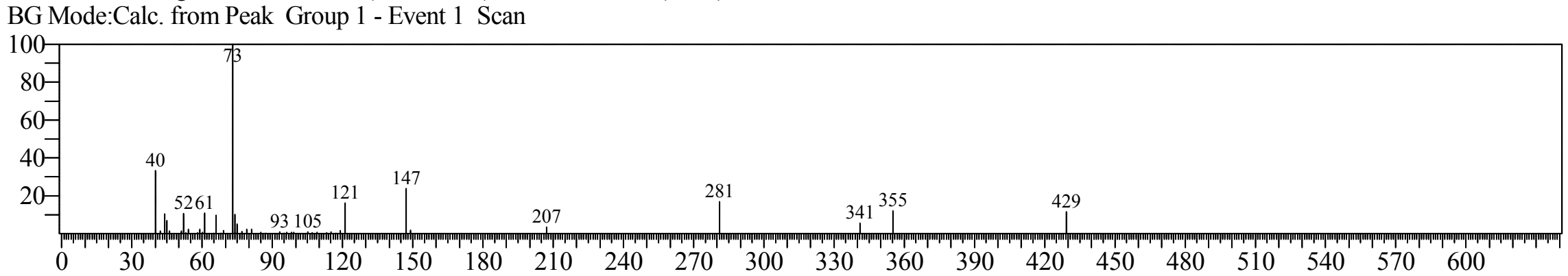

Hit#:11 Entry:38786 Library:NIST05.LIB
SI:64 Formula:C11H16O3 CAS:54710-16-6 MolWeight:196 RetIndex:1516
CompName:4-[1,3]Dioxolan-2-yl-3,4-dimethyl-cyclohex-2-enone $$ 4-(1,3-Dioxolan-2-yl)-3,4-dimethyl-2-cyclohexen-1-one # $$

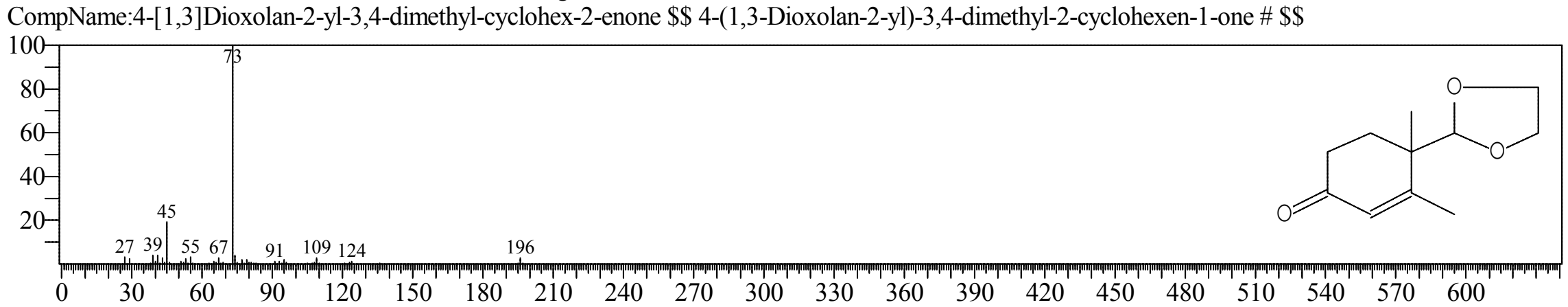

Hit#:12 Entry:4065 Library:WILEY7.LIB
SI:64 Formula:C4 H12 SI CAS:75-76-3 MolWeight:88 RetIndex:0
CompName:Silane, tetramethyl- (CAS) Tetramethylsilane $$ (CH3)4Si $$ Silicon, tetramethyl- $$ Tetramethylsilicane $$ UN 2749 $$ Si(CH3)4 $$ Tetram

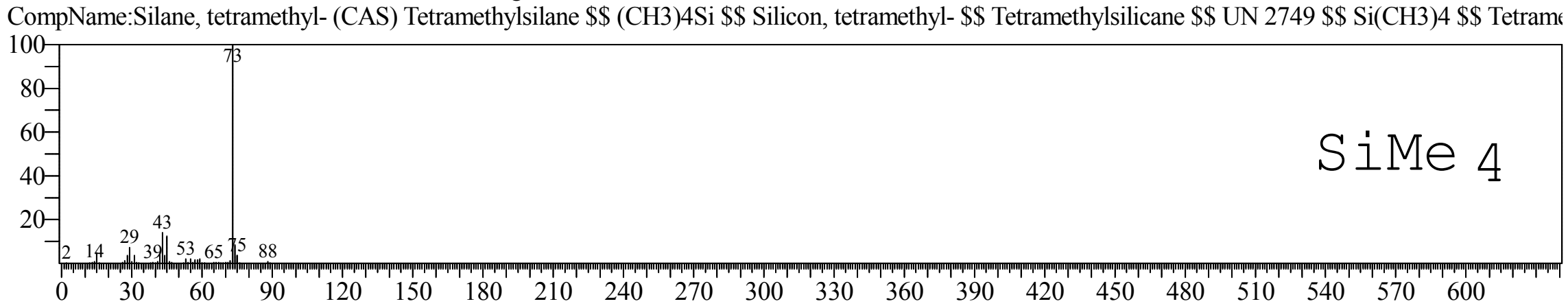

Hit#:13 Entry:159206 Library:WILEY7.LIB
SI:64 Formula:C14 H28 SI2 CAS:0-00-0 MolWeight:252 RetIndex:0
CompName:SPIRO[2.4]HEPT-5-EN, 5-TRIMETHYLSILYLMETHYL-1-TRI METHYLSILYL- $$

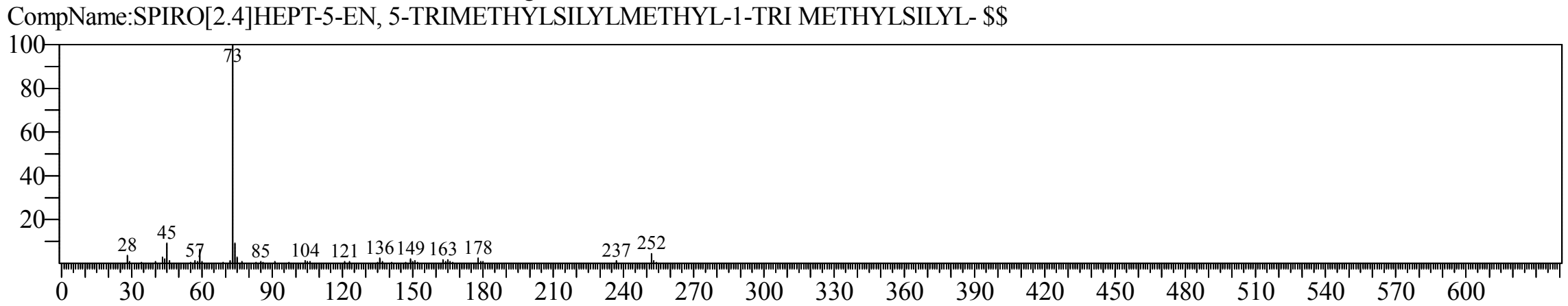

Hit#:14 Entry:72303 Library:NIST05.LIB
SI:64 Formula:C14H28Si2 CAS:0-00-0 MolWeight:252 RetIndex:1204
CompName:Spiro[2.4]hept-5-ene, 5-trimethylsilylmethyl-1-trimethylsilyl-

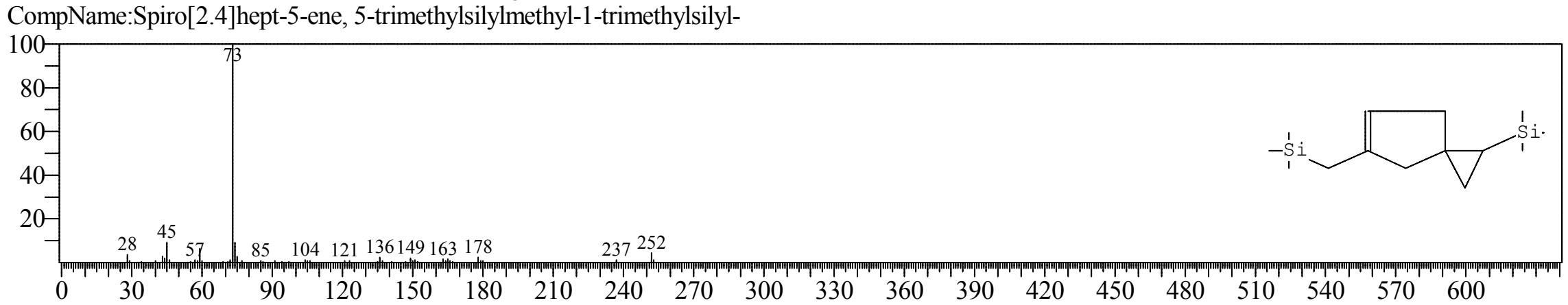

Hit#:15 Entry:81031 Library:NIST11.lib
SI:64 Formula:C14H28Si2 CAS:0-00-0 MolWeight:252 RetIndex:1204
CompName:Spiro[2.4]hept-5-ene, 5-trimethylsilylmethyl-1-trimethylsilyl- $$ Trimethyl([1-(trimethylsilyl)spiro[2.4]hept-5-en-5-yl]methyl)silane # $$

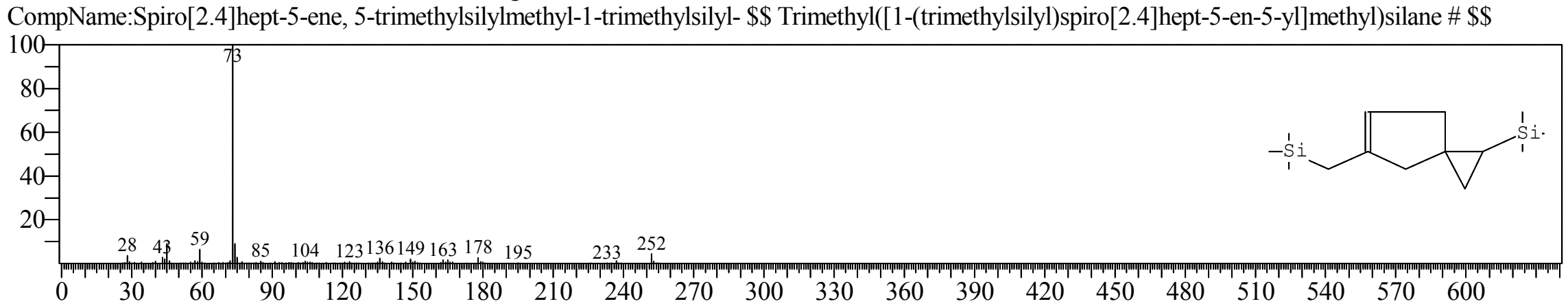

<< Target >>
Line#:7   R.Time:13.700(Scan#:1045) MassPeaks:47
RawMode:Averaged 13.692-13.708(1044-1046) BasePeak:73.05(6485)
BG Mode:Calc. from Peak Group 1 - Event 1 Scan

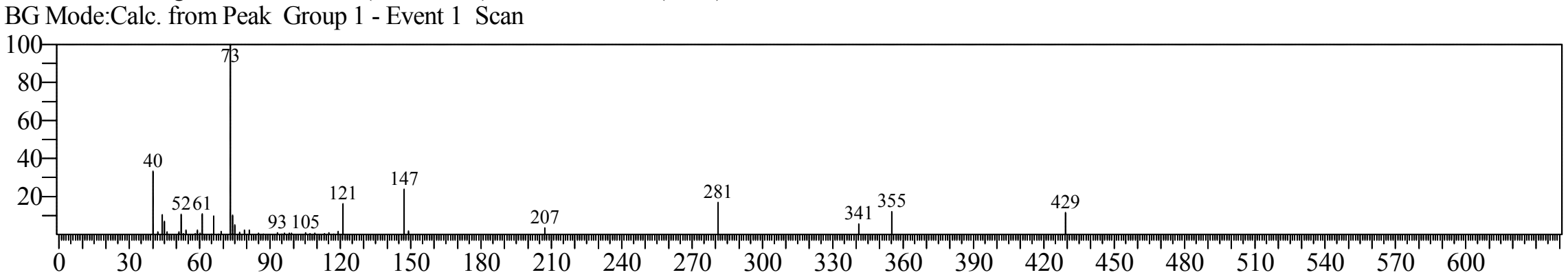

Hit#:16 Entry:89966 Library:WILEY7.LIB
SI:64 Formula:C11 H16 O3 CAS:0-00-0 MolWeight:196 RetIndex:0
CompName:4-[1,3]DIOXOLAN-2-YL-3,4-DIMETHYL-CYCLOHEX-2-ENONE $$

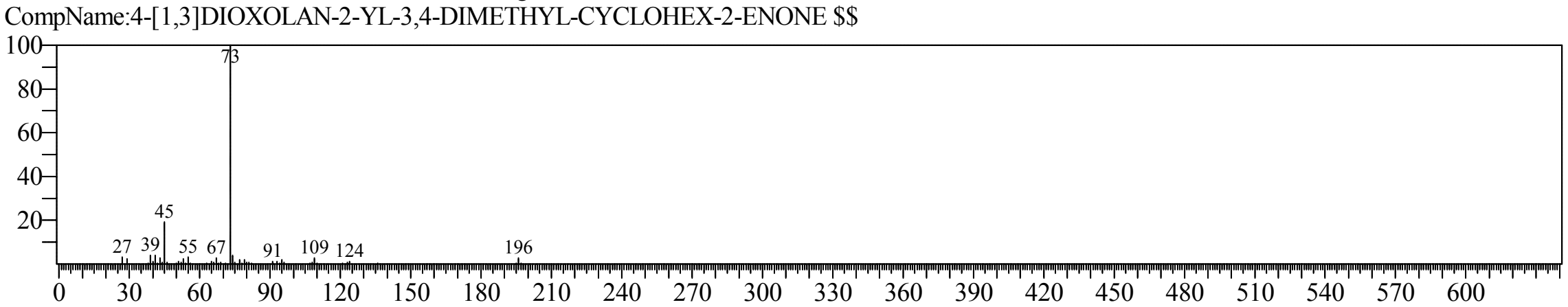

Hit#:17 Entry:93792 Library:NIST05.LIB
SI:63 Formula:C13H28O3Si2 CAS:0-00-0 MolWeight:288 RetIndex:1339
CompName:2-Ethyl-3-ketovalerate, bis(trimethylsilyl)

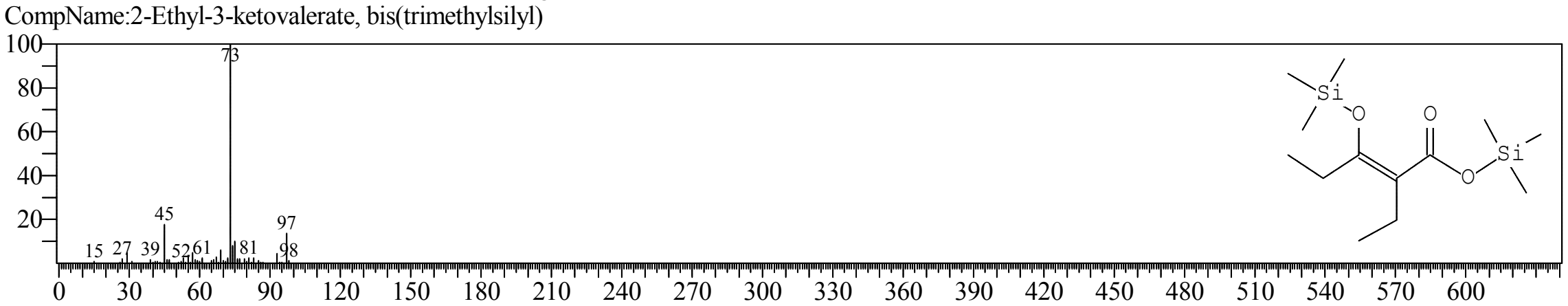

Hit#:18 Entry:90046 Library:WILEY7.LIB
SI:63 Formula:C11 H20 O SI CAS:0-00-0 MolWeight:196 RetIndex:0
CompName:CYCLOPENTENE, 3-METHYL-3-(TRIMETHYLSILYL)ACETYL- $$

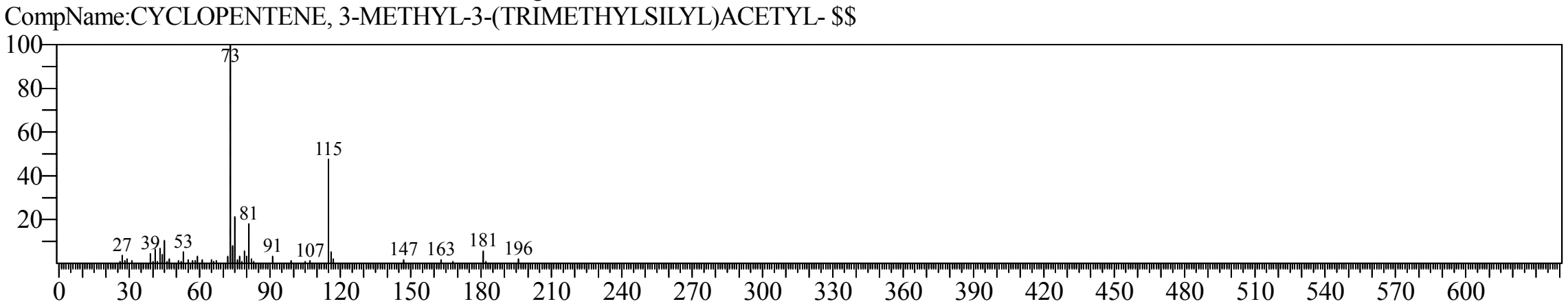

Hit#:19 Entry:38841 Library:NIST05.LIB
SI:63 Formula:C11H20OSi CAS:69561-98-4 MolWeight:196 RetIndex:1160
CompName:Cyclopentene, 3-methyl-3-(trimethylsilyl)acetyl- $$ 1-(1-Methyl-2-cyclopenten-1-yl)-2-(trimethylsilyl)ethanone # $$

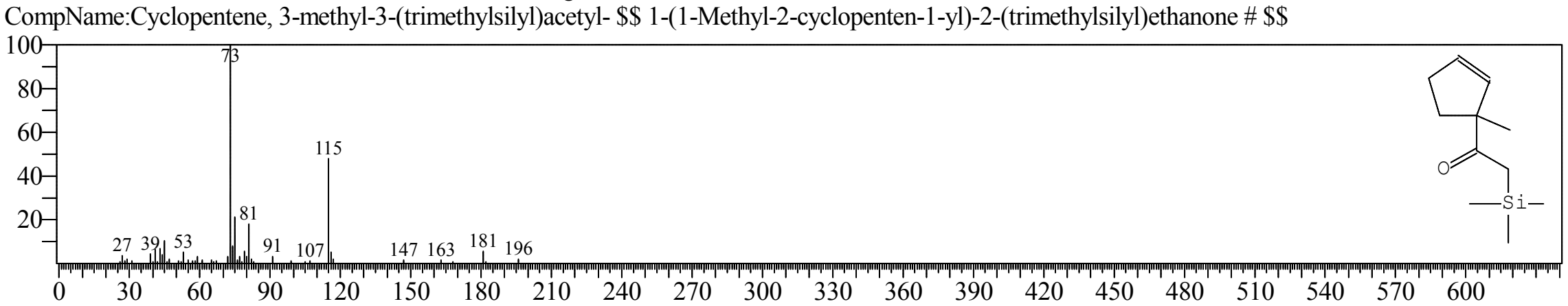

Hit#:20 Entry:98393 Library:NIST05.LIB
SI:63 Formula:C14H24O3Si2 CAS:27750-55-6 MolWeight:296 RetIndex:1566
CompName:Benzeneacetic acid, 3-[(trimethylsilyl)oxy]-, trimethylsilyl ester $$ Acetic acid, [m-(trimethylsiloxy)phenyl]-, trimethylsilyl ester $$ m-Hydroxyp

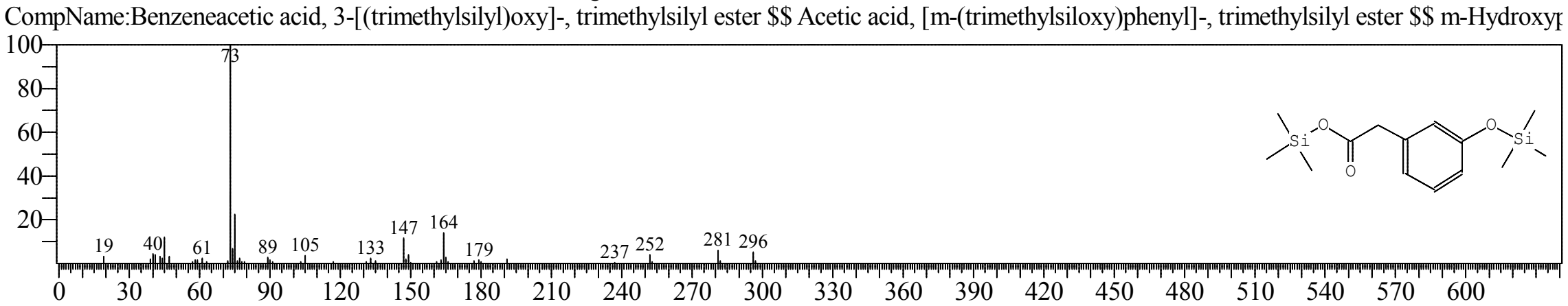

<< Target >>
Line#:8 R.Time:13.892(Scan#:1068) MassPeaks:66
RawMode:Averaged 13.883-13.900(1067-1069) BasePeak:60.05(7252)
BG Mode:Calc. from Peak Group 1 - Event 1 Scan

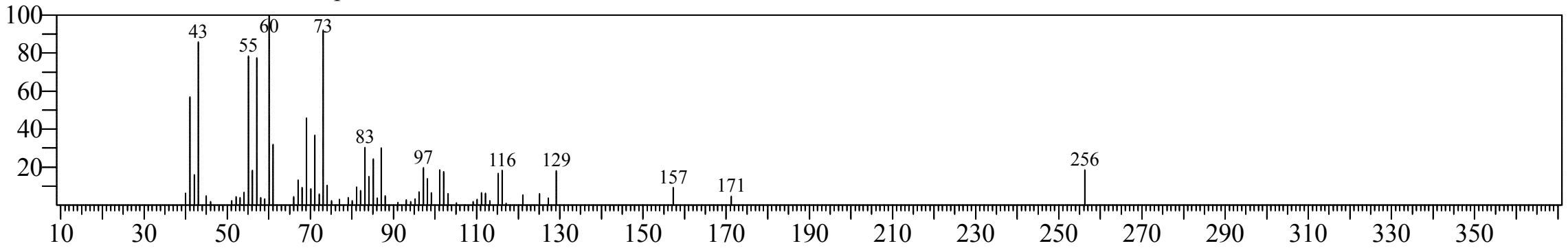

Hit#:1 Entry:23313 Library:NIST11s.lib
SI:91 Formula:C16H32O2 CAS:57-10-3 MolWeight:256 RetIndex:1968
CompName:n-Hexadecanoic acid $$ Hexadecanoic acid $$ n-Hexadecoic acid $$ Palmitic acid $$ Pentadecanecarboxylic acid $$ 1-Pentadecanecarboxylic

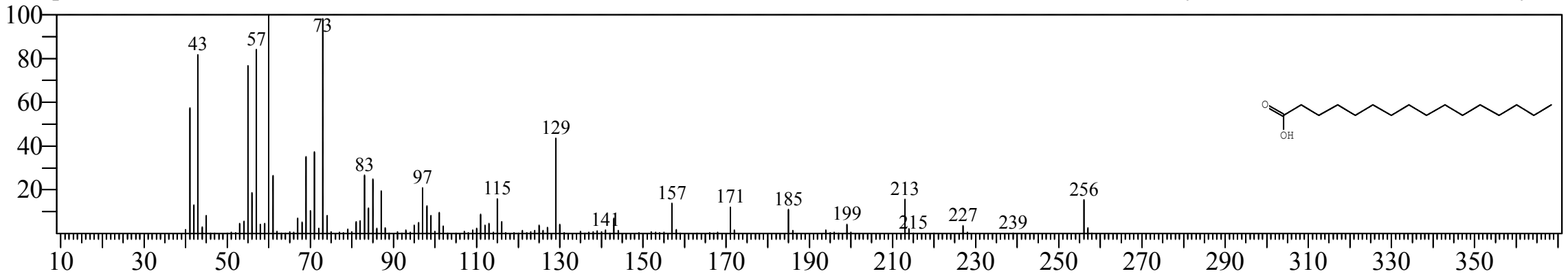

Hit#:2 Entry:193348 Library:WILEY7.LIB
SI:88 Formula:C18 H34 O2 CAS:112-80-1 MolWeight:282 RetIndex:0
CompName:9-Octadecenoic acid (Z)- (CAS) Oleic acid $$ Red oil $$ Oelsauere $$ Oleine 7503 $$ Pamolyn 100 $$ Emersol 211 $$ Vopcolene 27 $$ cis-O

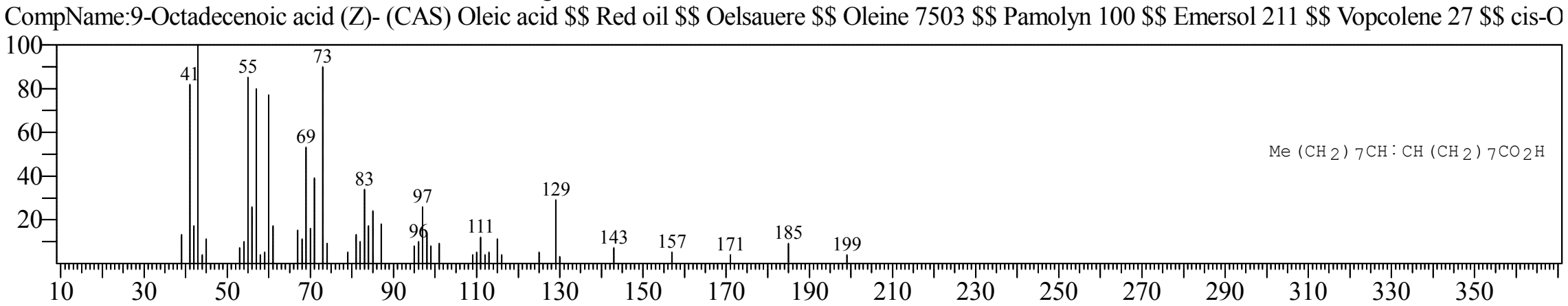

Hit#:3 Entry:53599 Library:NIST11.lib
SI:88 Formula:C13H26O2 CAS:638-53-9 MolWeight:214 RetIndex:1670
CompName:Tridecanoic acid $$ n-Tridecanoic acid $$ n-Tridecoic acid $$ Tridecylic acid $$

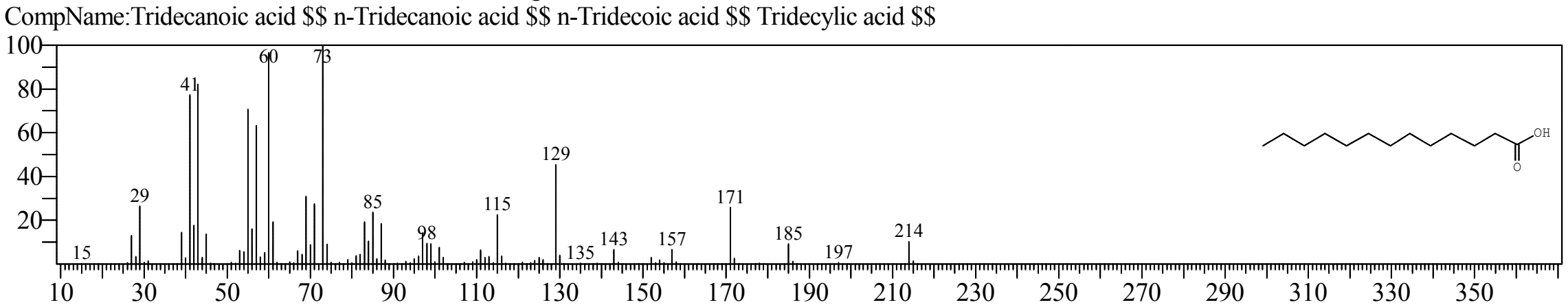

Hit#:4 Entry:195573 Library:WILEY7.LIB
SI:88 Formula:C18 H36 O2 CAS:57-11-4 MolWeight:284 RetIndex:0
CompName:Octadecanoic acid (CAS) Stearic acid $$ n-Octadecanoic acid $$ PD 185 $$ NAA 173 $$ Vanicol $$ Kam 3000 $$ Kam 1000 $$ Kam 2000 $$

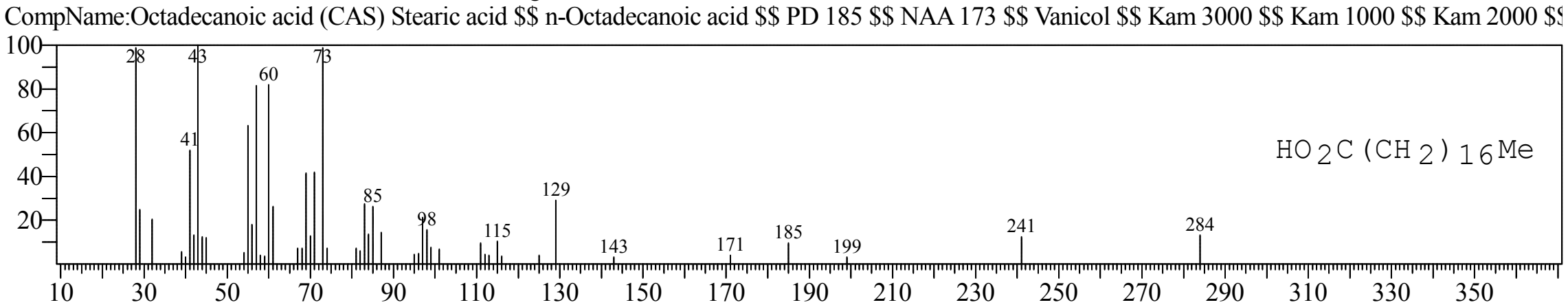

Hit#:5 Entry:73851 Library:NIST11.lib
SI:88 Formula:C15H30O2 CAS:1002-84-2 MolWeight:242 RetIndex:1869
CompName:Pentadecanoic acid $$ Pentadecylic acid $$ n-Pentadecanoic acid $$ n-Pentadecylic acid $$

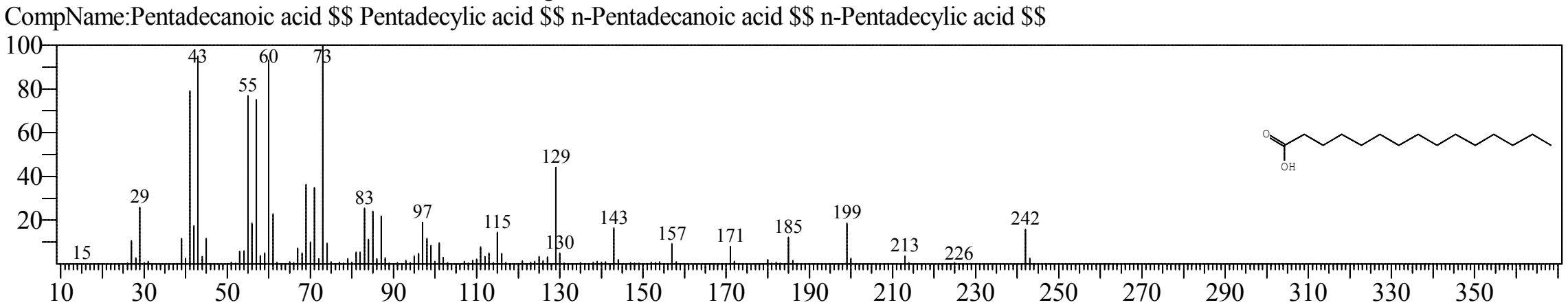

<< Target >>
Line#:8   R.Time:13.892(Scan#:1068) MassPeaks:66
RawMode:Averaged 13.883-13.900(1067-1069) BasePeak:60.05(7252)
BG Mode:Calc. from Peak Group 1 - Event 1 Scan

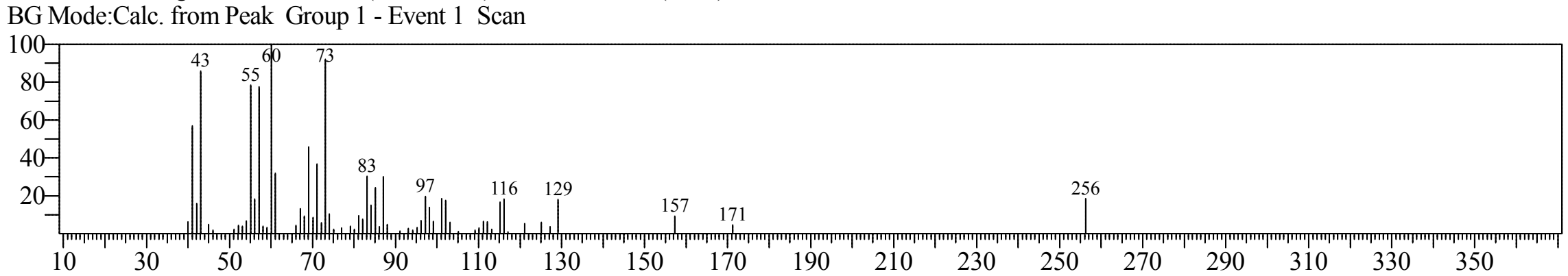

Hit#:6 Entry:19250 Library:NIST05s.LIB
SI:87 Formula:C14H28O2 CAS:544-63-8 MolWeight:228 RetIndex:1769
CompName:Tetradecanoic acid $$ Myristic acid $$ n-Tetradecanoic acid $$ n-Tetradecoic acid $$ Neo-Fat 14 $$ Univol U 316S $$ 1-Tridecanecarboxylic a

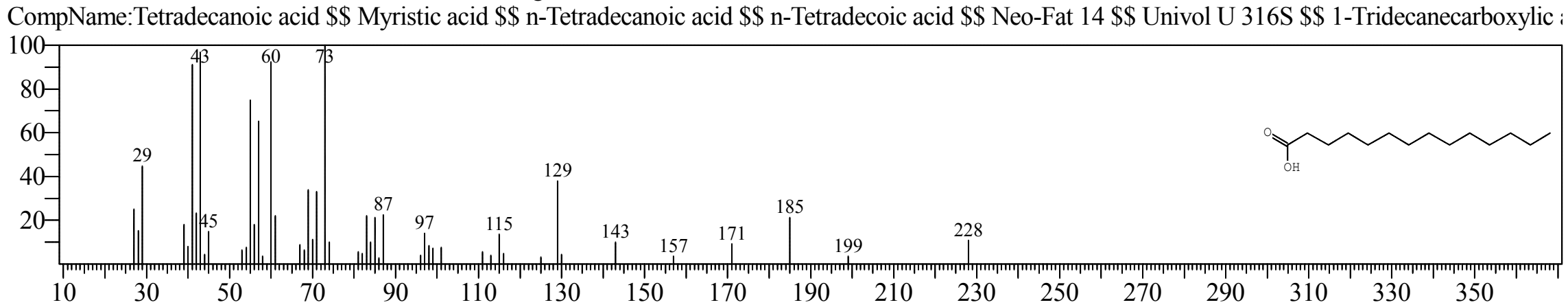

Hit#:7 Entry:16239 Library:NIST05s.LIB
SI:87 Formula:C12H24O2 CAS:143-07-7 MolWeight:200 RetIndex:1570
CompName:Dodecanoic acid $$ n-Dodecanoic acid $$ Neo-fat 12 $$ Aliphat no. 4 $$ Abl $$ Dodecylic acid $$ Lauric acid $$ Laurostearic acid $$ Neo-fat

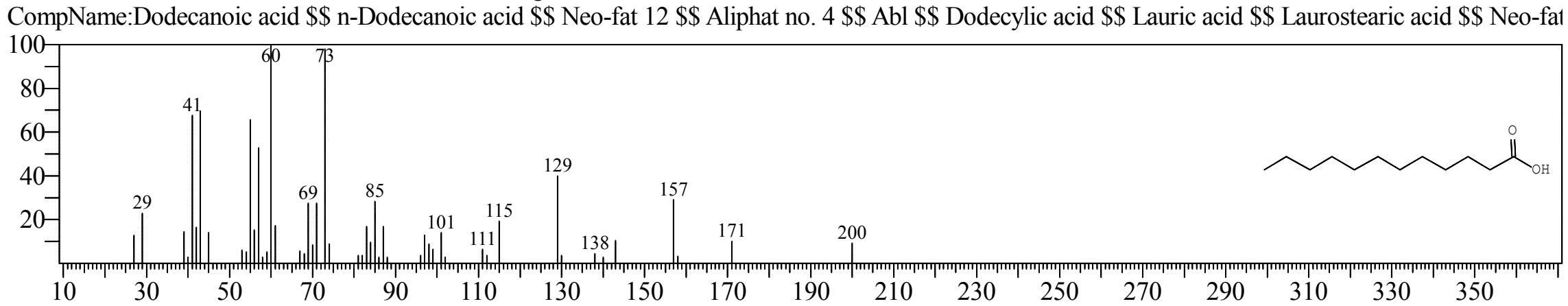

Hit#:8 Entry:108054 Library:NIST05.LIB
SI:87 Formula:C20H40O2 CAS:506-30-9 MolWeight:312 RetIndex:2366
CompName:Eicosanoic acid $$ Arachic acid $$ Arachidic acid $$ Icosanoic acid $$ n-Eicosanoic acid $$ Arachidic acid (synthetic) $$

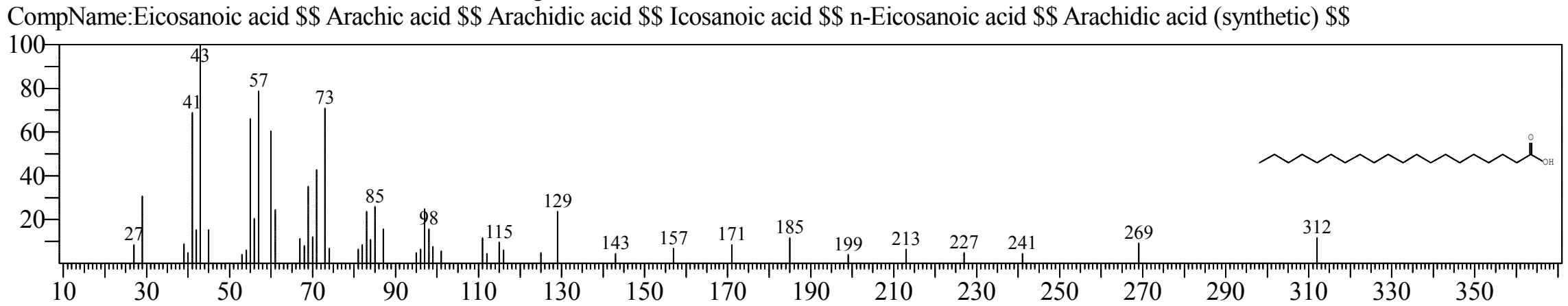

Hit#:9 Entry:209831 Library:WILEY7.LIB
SI:86 Formula:C19 H38 O2 CAS:646-30-0 MolWeight:298 RetIndex:0
CompName:Nonadecanoic acid (CAS) n-Nonadecanoic acid $$

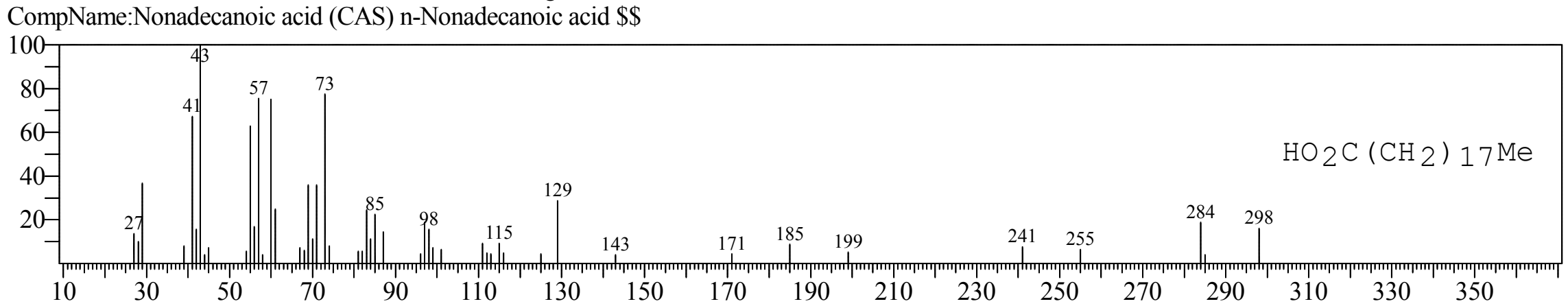

Hit#:10 Entry:247102 Library:WILEY7.LIB
SI:86 Formula:C22 H44 O2 CAS:112-85-6 MolWeight:340 RetIndex:0
CompName:Docosanoic acid (CAS) Behenic acid $$ Glycon B-70 $$ Hydrofol 2022-55 $$ Hydrofol Acid 560 $$ n-Docosanoic acid $$ 1-Docosanoic acid

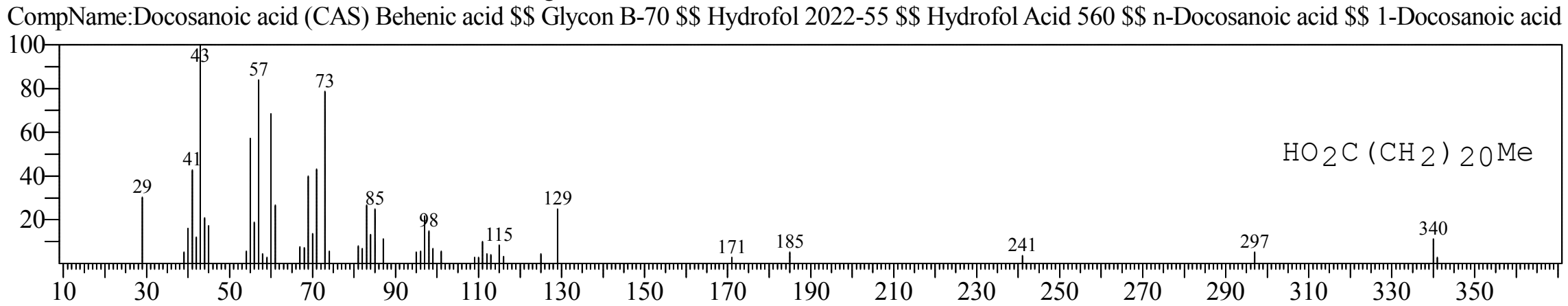

<< Target >>
Line#:8 R.Time:13.892(Scan#:1068) MassPeaks:66
RawMode:Averaged 13.883-13.900(1067-1069) BasePeak:60.05(7252)
BG Mode:Calc. from Peak Group 1 - Event 1 Scan

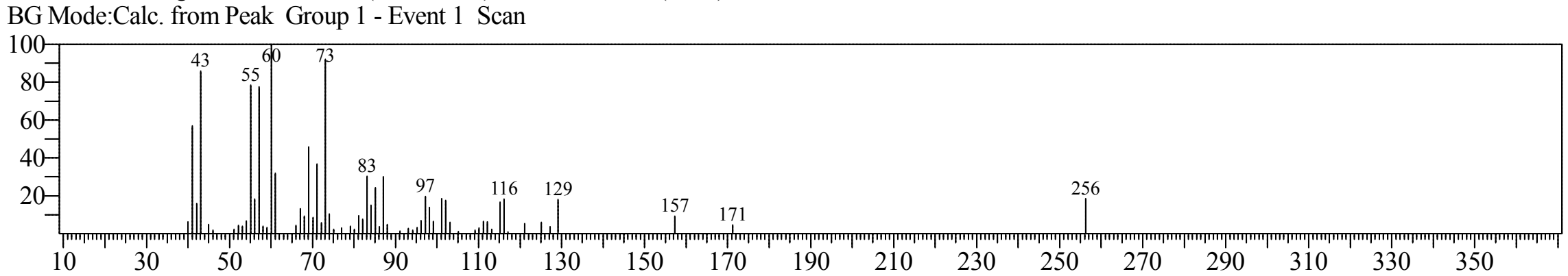

Hit#:11 Entry:326223 Library:WILEY7.LIB
SI:86 Formula:C35 H68 O5 CAS:502-52-3 MolWeight:569 RetIndex:0
CompName:Hexadecanoic acid, 2-hydroxy-1,3-propanediyl ester (CAS) GLYCEROL 1,3-DIHEXADECANOATE $$ 1,3-Dipalmitin $$ Palmitin, 1,3-di- $$

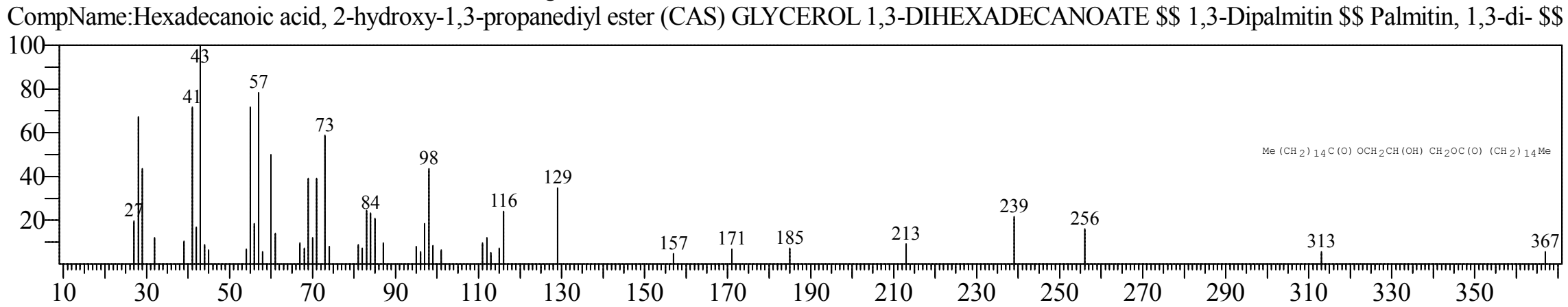

Hit#:12 Entry:63216 Library:WILEY7.LIB
SI:86 Formula:C10 H20 O2 CAS:334-48-5 MolWeight:172 RetIndex:0
CompName:Decanoic acid (CAS) Capric acid $$ Decoic acid $$ Decylic acid $$ Caprynic acid $$ Caprinic acid $$ n-Decoic acid $$ n-Capric acid $$ n-De

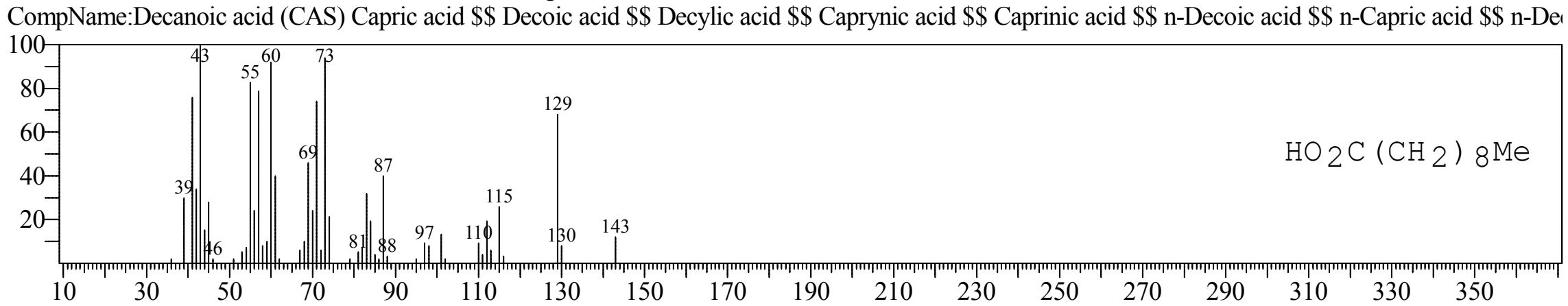

Hit#:13 Entry:198305 Library:WILEY7.LIB
SI:85 Formula:C16 H33 N O3 CAS:120-40-1 MolWeight:287 RetIndex:0
CompName:Dodecanamide, N,N-bis(2-hydroxyethyl)- $$ Bis(2-hydroxyethyl)lauramide $$ LDA $$ LDE $$ Clindrol Superamide 100L $$ Clindrol 200L $

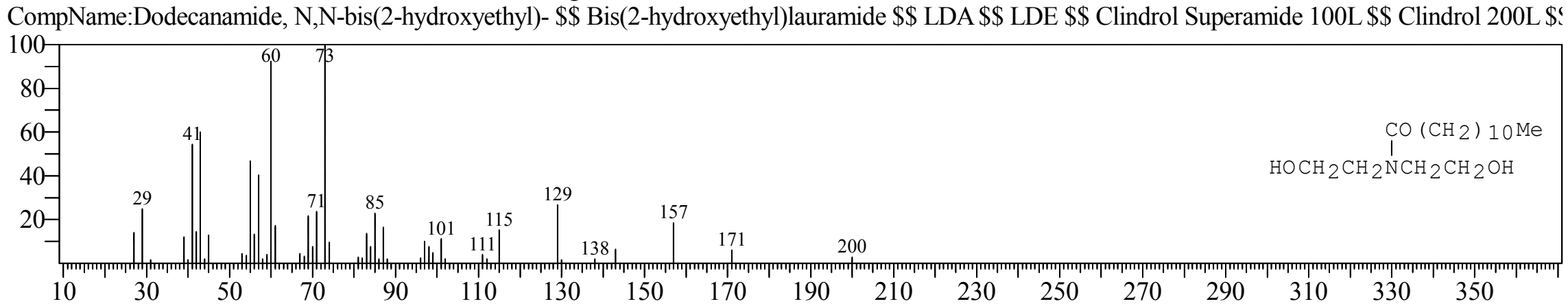

Hit#:14 Entry:235482 Library:WILEY7.LIB
SI:85 Formula:C21 H42 O2 CAS:2363-71-5 MolWeight:326 RetIndex:0
CompName:Heneicosanoic acid (CAS) n-Heneicosanoic acid $$

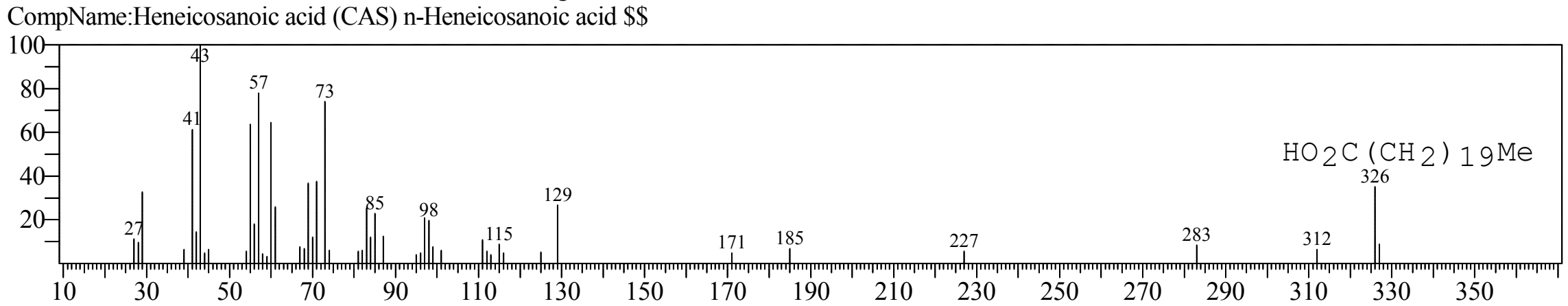

Hit#:15 Entry:177663 Library:WILEY7.LIB
SI:85 Formula:C17 H32 O2 CAS:26265-99-6 MolWeight:268 RetIndex:0
CompName:Heptadecenoic acid (CAS) Heptadecamonoenoic acid $$

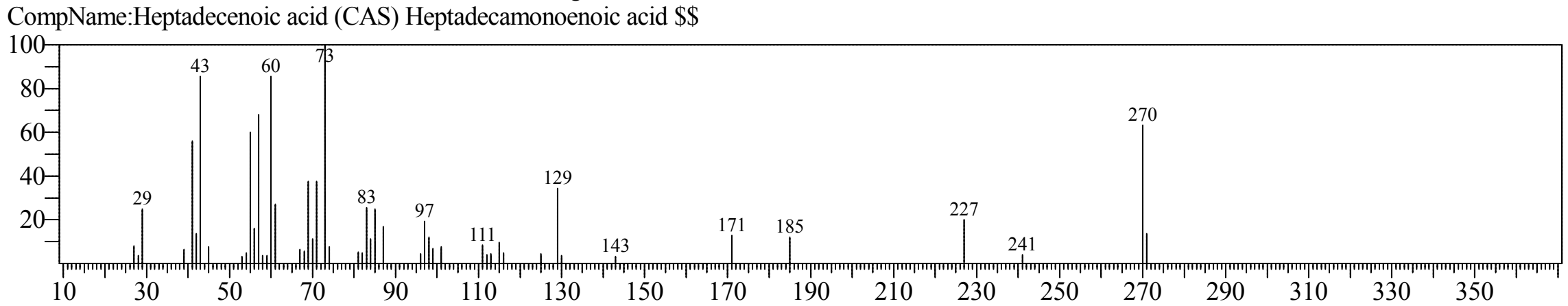

<< Target >>
Line#:8   R.Time:13.892(Scan#:1068) MassPeaks:66
RawMode:Averaged 13.883-13.900(1067-1069) BasePeak:60.05(7252)
BG Mode:Calc. from Peak Group 1 - Event 1 Scan

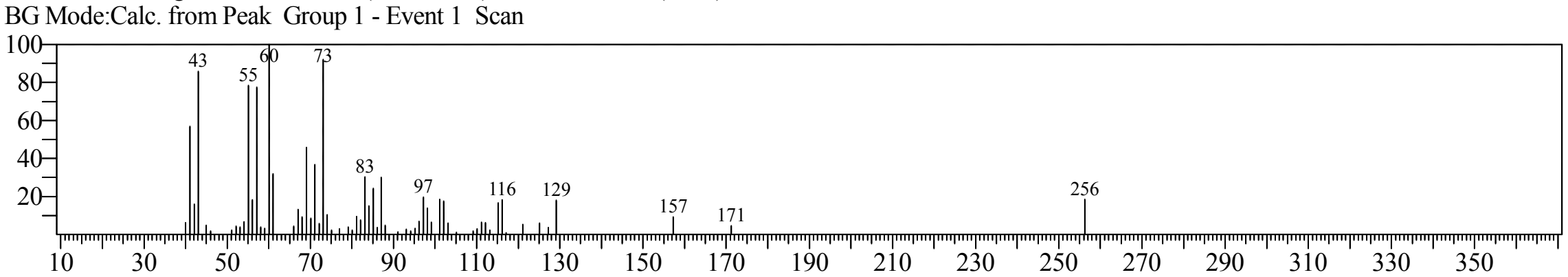

Hit#:16 Entry:22216 Library:NIST05s.LIB
SI:85 Formula:C17H34O2 CAS:506-12-7 MolWeight:270 RetIndex:2067
CompName:Heptadecanoic acid $$ n-Heptadecanoic acid $$ n-Heptadecoic acid $$ n-Heptadecylic acid $$ Margaric acid $$ Margarinic acid $$ Normal-he

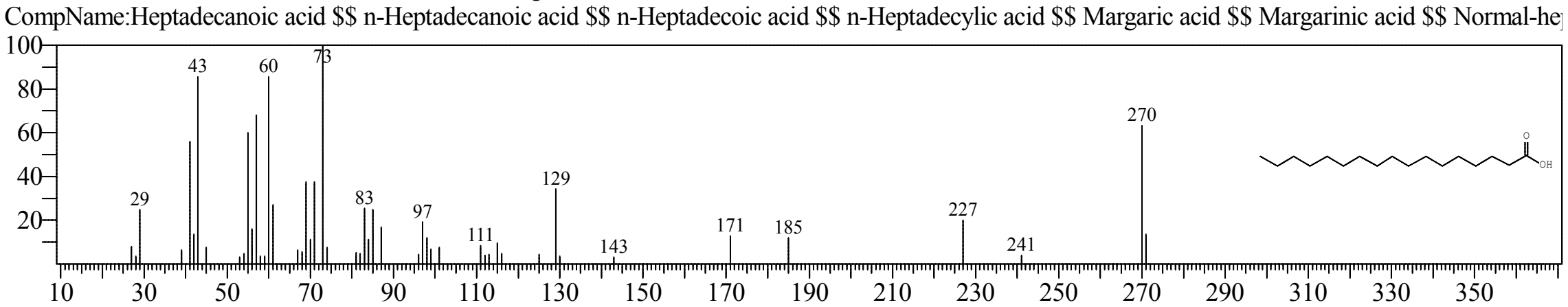

Hit#:17 Entry:217709 Library:WILEY7.LIB
SI:84 Formula:C12 H24 AG O2 CAS:18268-45-6 MolWeight:307 RetIndex:0
CompName:Dodecanoic acid, silver(1+) salt (CAS) Silver laurate $$ Lauric acid, silver(1+) salt $$

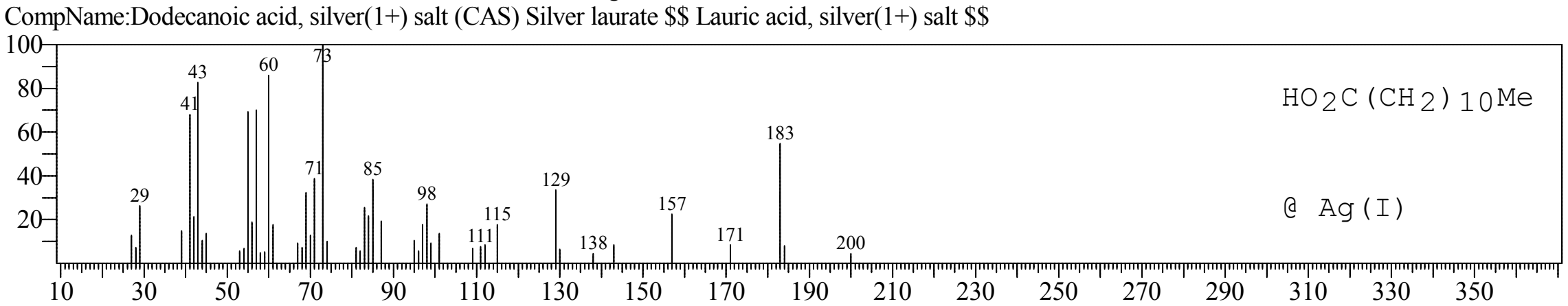

Hit#:18 Entry:79101 Library:WILEY7.LIB
SI:84 Formula:C11 H22 O2 CAS:112-37-8 MolWeight:186 RetIndex:0
CompName:Undecanoic acid (CAS) Undecylic acid $$ n-Undecoic acid $$ Hendecanoic acid $$ n-Undecylic acid $$ n-Undecanoic acid $$ 1-Decanecarbo

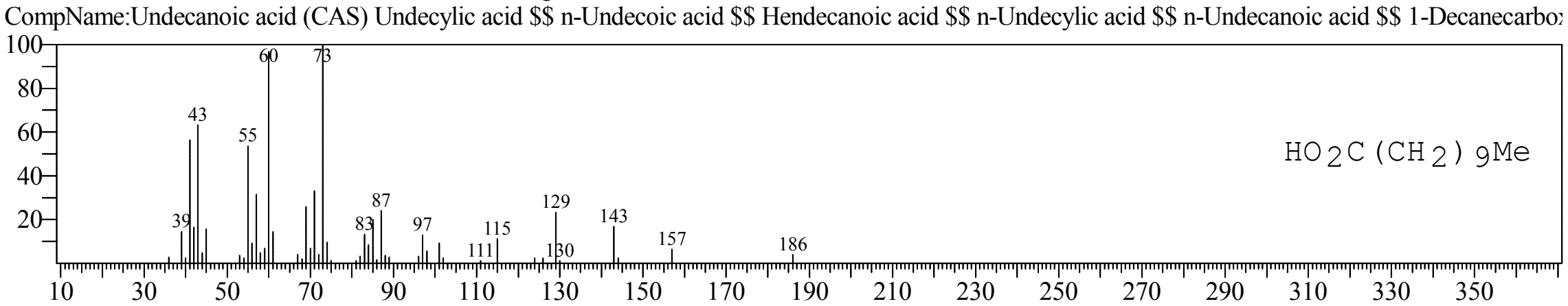

Hit#:19 Entry:161860 Library:NIST05.LIB
SI:84 Formula:C38H68O8 CAS:28474-90-0 MolWeight:652 RetIndex:4765
CompName:l-(+)-Ascorbic acid 2,6-dihexadecanoate

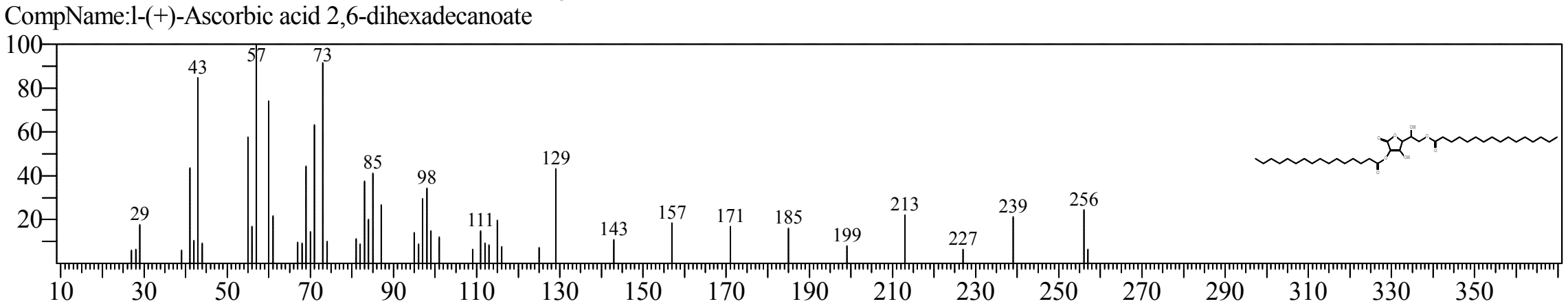

Hit#:20 Entry:134715 Library:NIST05.LIB
SI:84 Formula:C24H48O2 CAS:557-59-5 MolWeight:368 RetIndex:2763
CompName:Tetracosanoic acid $$ Lignoceric acid $$

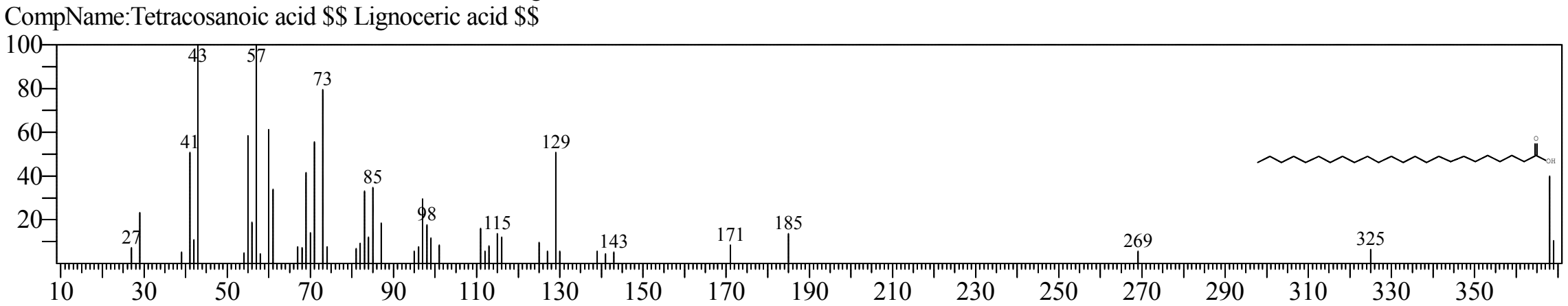

<< Target >>
Line#:9 R.Time:14.008(Scan#:1082) MassPeaks:44
RawMode:Averaged 14.000-14.017(1081-1083) BasePeak:149.10(3375)
BG Mode:Calc. from Peak Group 1 - Event 1 Scan

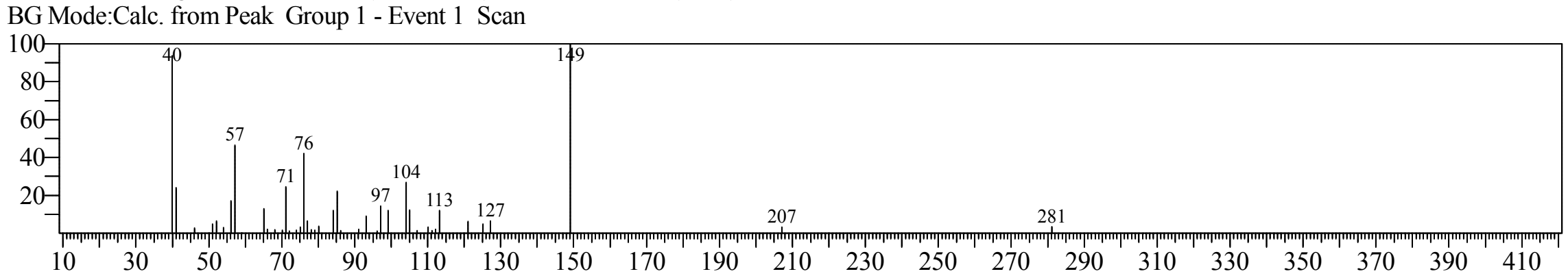

Hit#:1 Entry:26375 Library:NIST05s.LIB
SI:64 Formula:C24H38O4 CAS:117-84-0 MolWeight:390 RetIndex:2832
CompName:Di-n-octyl phthalate $$ 1,2-Benzenedicarboxylic acid, dioctyl ester $$ Phthalic acid, dioctyl ester $$ n-Octyl phthalate $$ Dinopol NOP $$ Dio

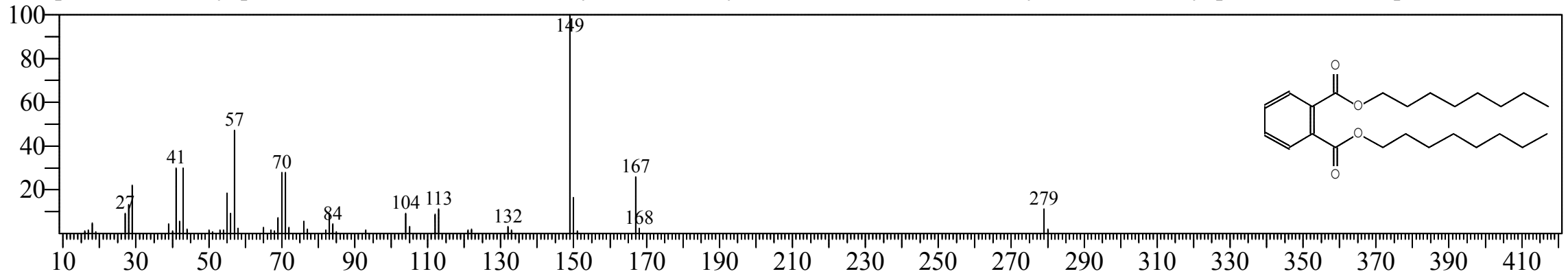

Hit#:2 Entry:29520 Library:NIST11s.lib
SI:63 Formula:C24H38O4 CAS:117-81-7 MolWeight:390 RetIndex:2704
CompName:Bis(2-ethylhexyl) phthalate $$ Phthalic acid, bis(2-ethylhexyl) ester $$ Bis(2-ethylhexyl) 1,2-benzenedicarboxylate $$ Bisoflex 81 $$ Compoun

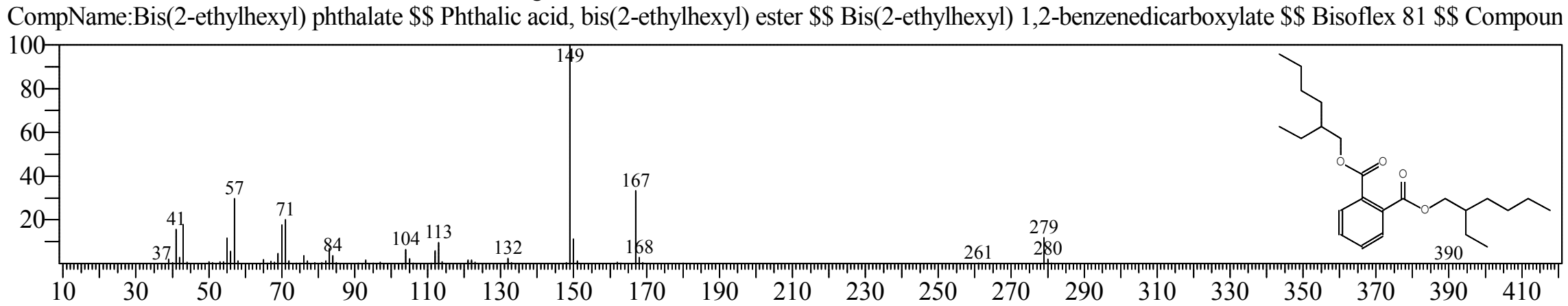

Hit#:3 Entry:26380 Library:NIST05s.LIB
SI:63 Formula:C24H38O4 CAS:27554-26-3 MolWeight:390 RetIndex:2704
CompName:1,2-Benzenedicarboxylic acid, diisooctyl ester $$ Diisooctyl phthalate $$ Hexaplas M/O $$ Isooctyl phthalate $$ Corflex 880 $$ DIOP $$ Flex

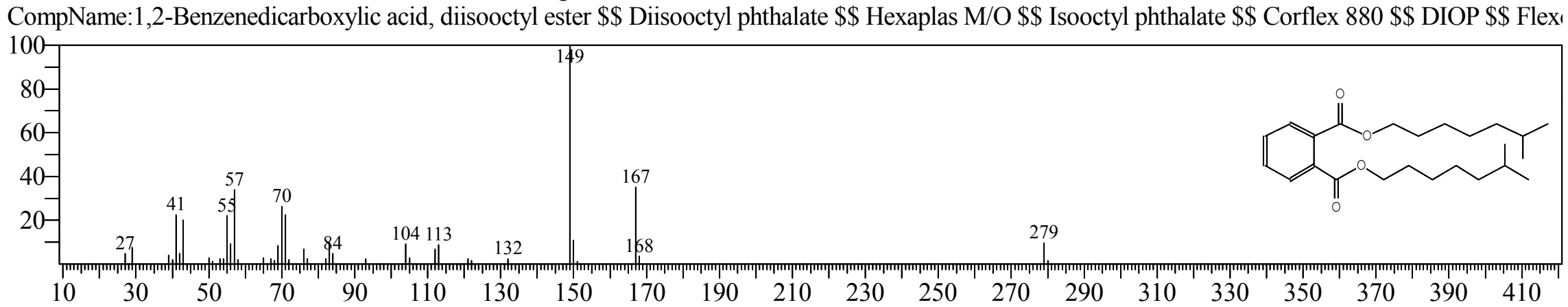

Hit#:4 Entry:29523 Library:NIST11s.lib
SI:63 Formula:C24H38O4 CAS:131-20-4 MolWeight:390 RetIndex:2704
CompName:Diisooctyl phthalate $$ Bis(6-methylheptyl) phthalate $$ 1,2-Benzenedicarboxylic acid, bis(6-methylheptyl) ester $$ Phthalic acid, bis(6-methyl

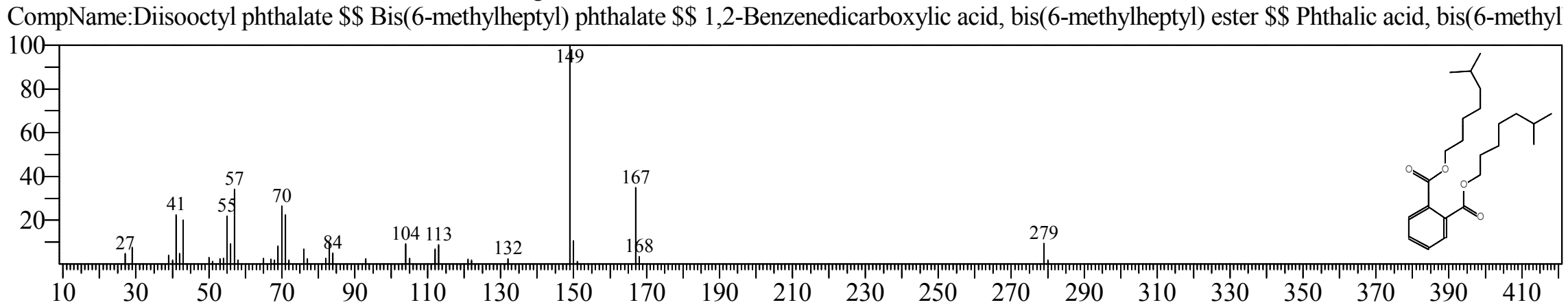

Hit#:5 Entry:79501 Library:NIST11.lib
SI:63 Formula:C14H18O4 CAS:0-00-0 MolWeight:250 RetIndex:2027
CompName:2-(Hexyloxycarbonyl)benzoic acid $$ Phthalic acid, monohexyl ester $$ Hexyl hydrogen phthalate $$ Monohexyl phthalate $$

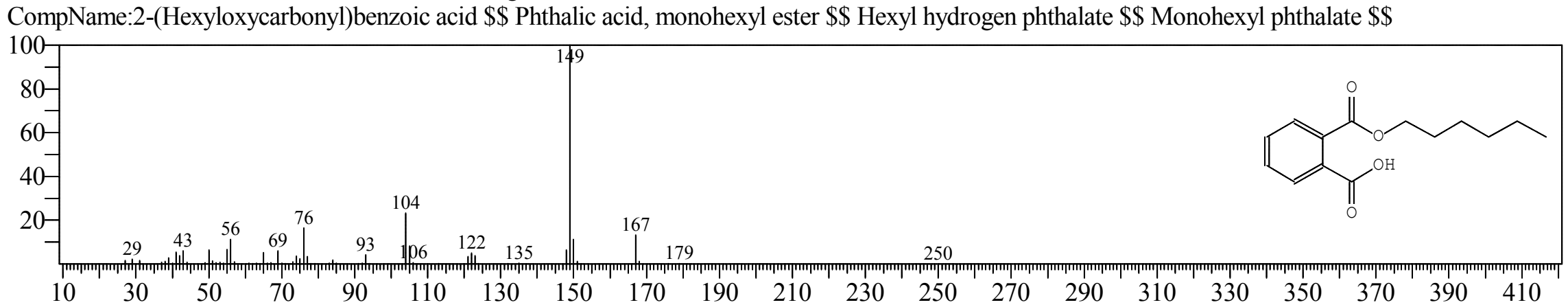

<< Target >>
Line#:9   R.Time:14.008(Scan#:1082) MassPeaks:44
RawMode:Averaged 14.000-14.017(1081-1083) BasePeak:149.10(3375)
BG Mode:Calc. from Peak Group 1 - Event 1 Scan

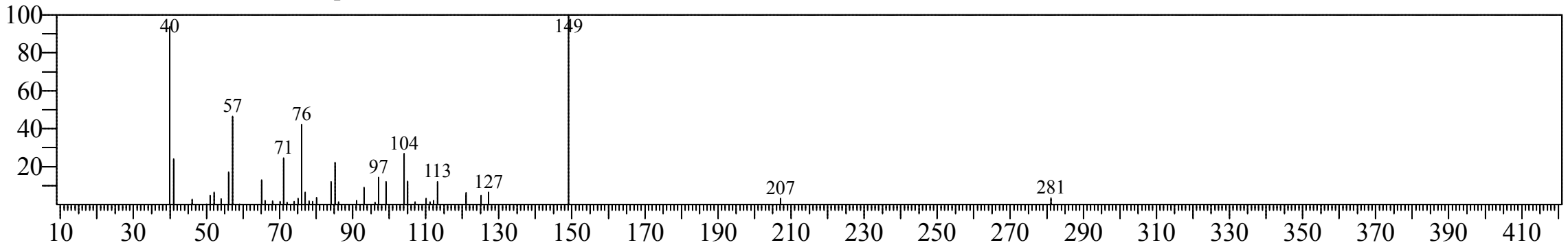

Hit#:6 Entry:58910 Library:NIST11.lib
SI:63 Formula:C12H14O4 CAS:0-00-0 MolWeight:222 RetIndex:1764
CompName:2-(Isobutoxycarbonyl)benzoic acid $$ Component at scan 1370 (14.624 min) [Model = +104u] in C:\\DATA\\11-15-09\\LMO_137_1.D\\DATA

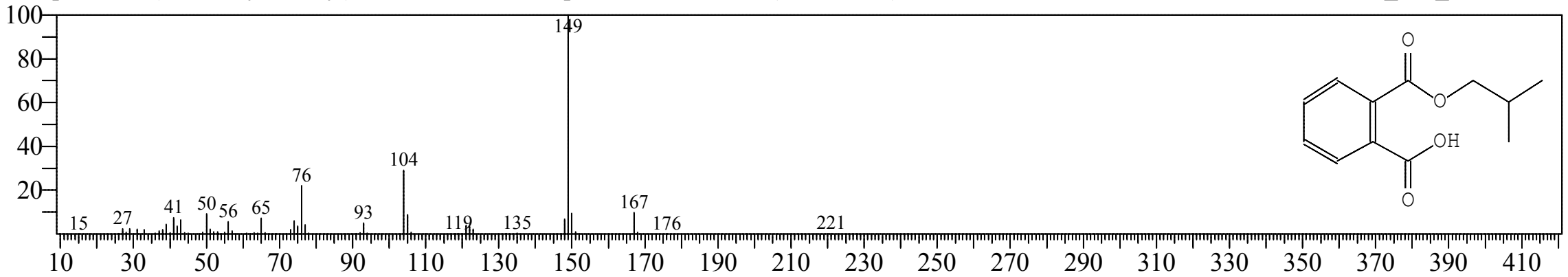

Hit#:7 Entry:232354 Library:WILEY7.LIB
SI:62 Formula:C18 H26 O5 CAS:33374-28-6 MolWeight:322 RetIndex:0
CompName:1,2-Benzenedicarboxylic acid, 2-butoxyethyl butyl ester (CAS) BUTYL BUTOXYETHYL PHTHALATE $$ BFBG $$ BUTYL PHTHALYL B

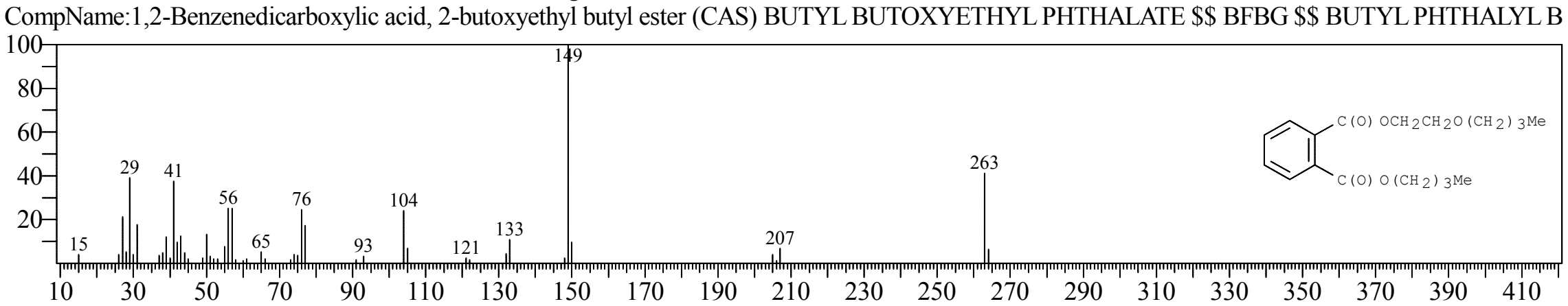

Hit#:8 Entry:25120 Library:NIST05s.LIB
SI:62 Formula:C18H24O6 CAS:85-70-1 MolWeight:336 RetIndex:2403
CompName:Phthalic acid, butyl ester, ester with butyl glycolate $$ Butyl carbobutoxymethyl phthalate $$ Butyl glycolyl butyl phthalate $$ Butyl phthalate b

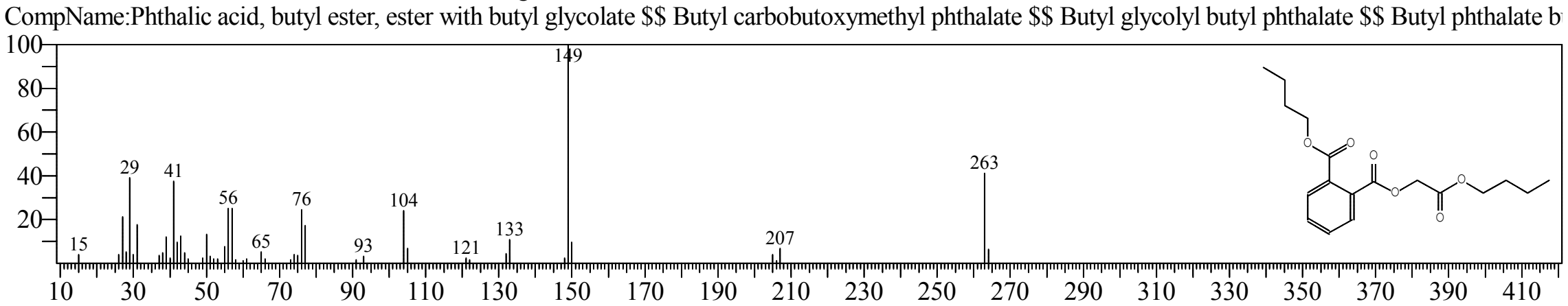

Hit#:9 Entry:123706 Library:WILEY7.LIB
SI:62 Formula:C12 H14 O4 CAS:84-66-2 MolWeight:222 RetIndex:0
CompName:1,2-Benzenedicarboxylic acid, diethyl ester (CAS) Ethyl phthalate $$ Diethyl phthalate $$ Anozol $$ Phthalol $$ Solvanol $$ Neantine $$ Placi

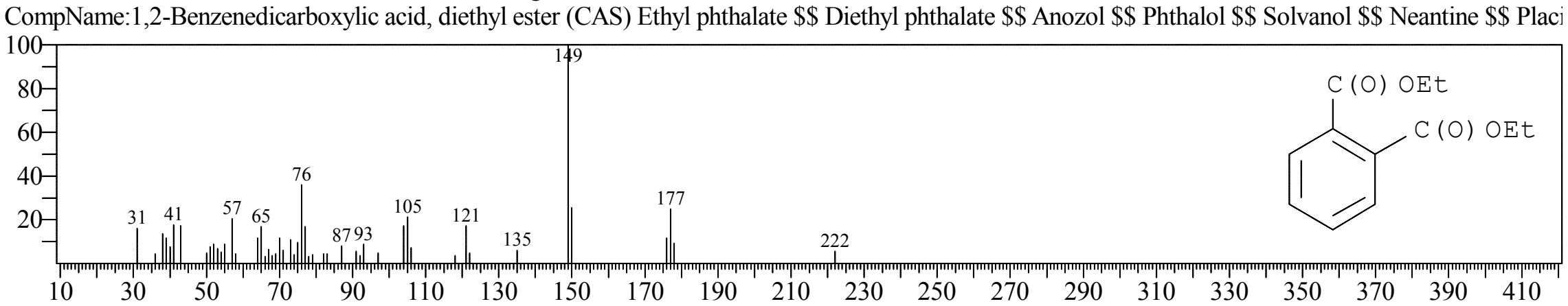

Hit#:10 Entry:158302 Library:NIST11.lib
SI:62 Formula:C22H26O4 CAS:0-00-0 MolWeight:354 RetIndex:2646
CompName:Phthalic acid, 2-ethylhexyl phenyl ester

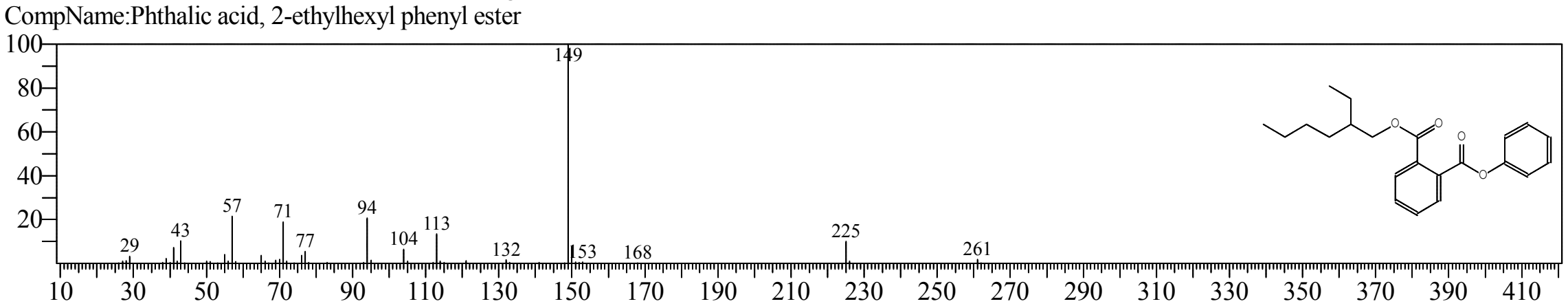

<< Target >>
Line#:9 R.Time:14.008(Scan#:1082) MassPeaks:44
RawMode:Averaged 14.000-14.017(1081-1083) BasePeak:149.10(3375)
BG Mode:Calc. from Peak Group 1 - Event 1 Scan

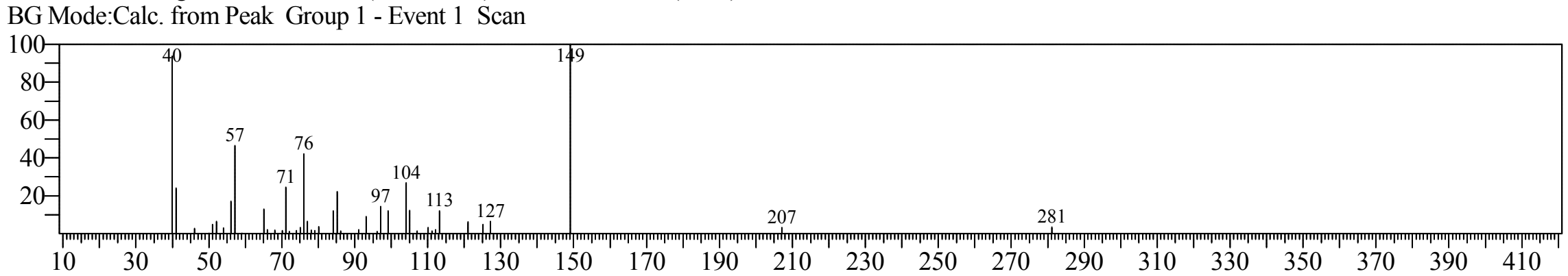

Hit#:11 Entry:188932 Library:WILEY7.LIB
SI:62 Formula:C16 H22 O4 CAS:84-74-2 MolWeight:278 RetIndex:0
CompName:1,2-Benzenedicarboxylic acid, dibutyl ester (CAS) Butyl phthalate $$ DI-N-BUTYLPHTHALATE $$ PX 104 $$ DIBUTYLPHTALATE $$ DI

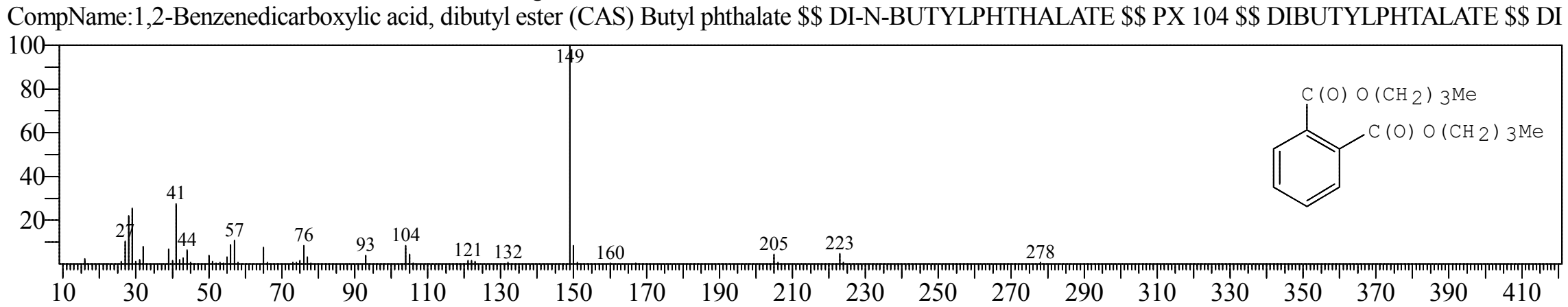

Hit#:12 Entry:129005 Library:NIST05.LIB
SI:62 Formula:C22H26O4 CAS:0-00-0 MolWeight:354 RetIndex:2646
CompName:Phthalic acid, 2-ethylhexyl phenyl ester

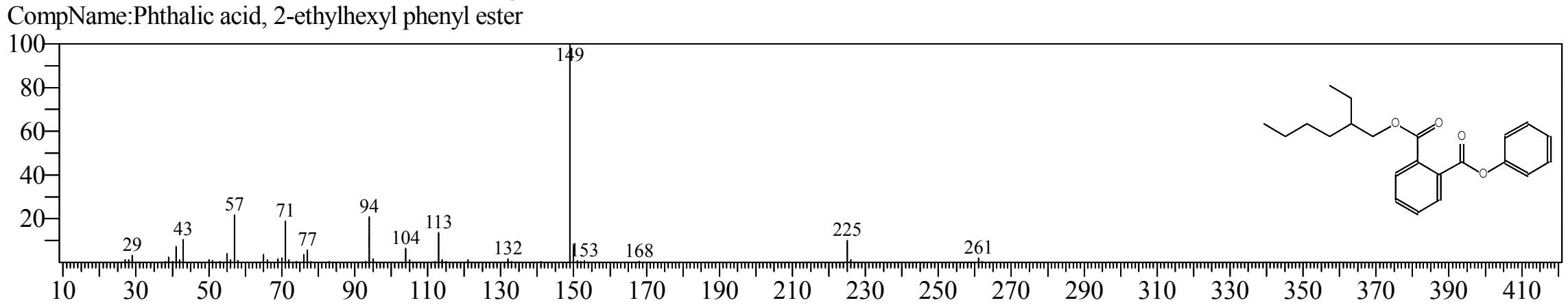

Hit#:13 Entry:188948 Library:WILEY7.LIB
SI:62 Formula:C16 H22 O4 CAS:84-69-5 MolWeight:278 RetIndex:0
CompName:1,2-Benzenedicarboxylic acid, bis(2-methylpropyl) ester (CAS) Isobutyl phthalate $$ ISOBUTYL O-PHTHALATE $$ Di-isobutyl phthalate $$

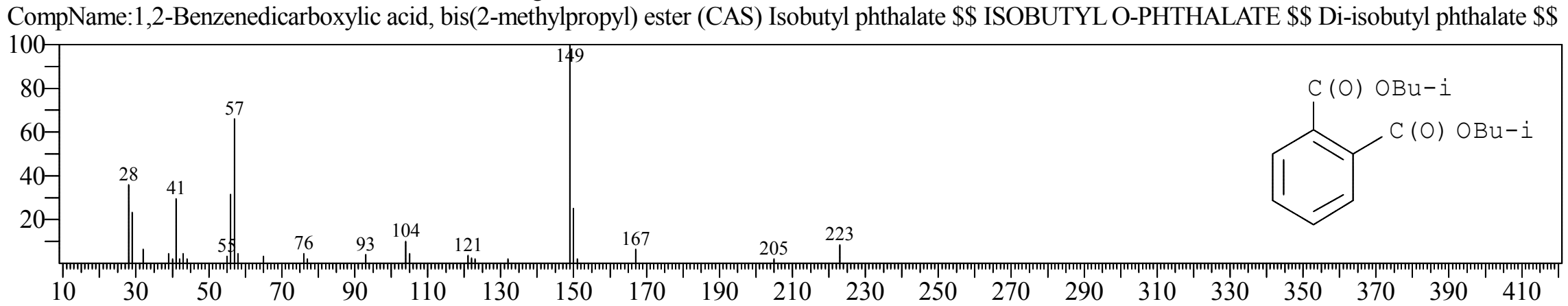

Hit#:14 Entry:96348 Library:NIST05.LIB
SI:62 Formula:C17H24O4 CAS:0-00-0 MolWeight:292 RetIndex:2136
CompName:Phthalic acid, hexyl propyl ester

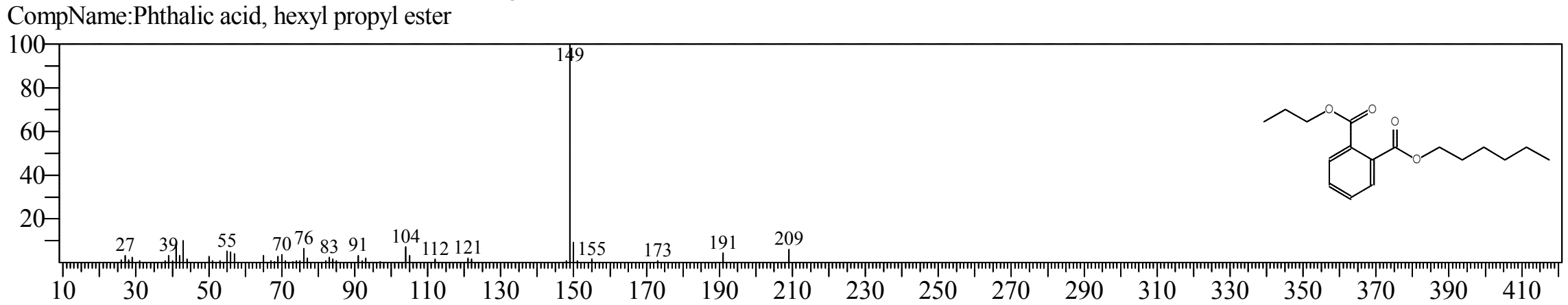

Hit#:15 Entry:112194 Library:NIST11.lib
SI:62 Formula:C17H24O4 CAS:0-00-0 MolWeight:292 RetIndex:2136
CompName:Phthalic acid, hexyl propyl ester

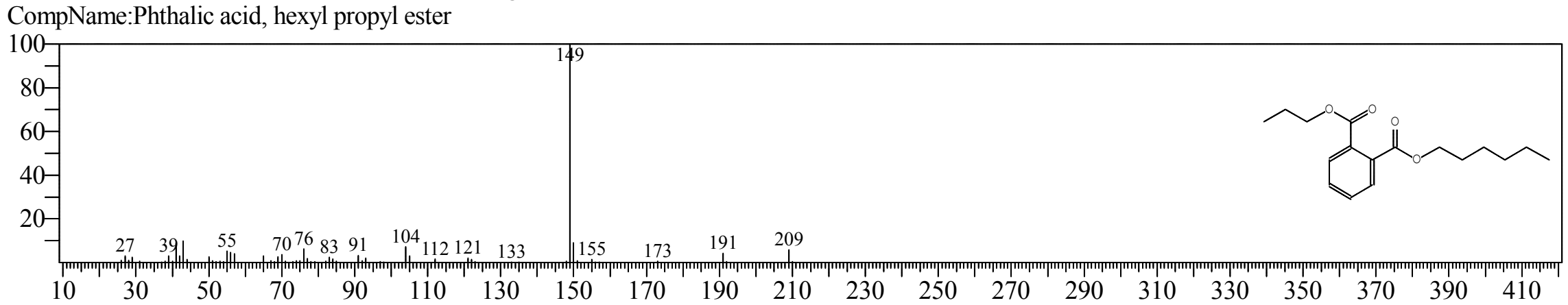

<< Target >>
Line#:9   R.Time:14.008(Scan#:1082) MassPeaks:44
RawMode:Averaged 14.000-14.017(1081-1083) BasePeak:149.10(3375)
BG Mode:Calc. from Peak Group 1 - Event 1 Scan

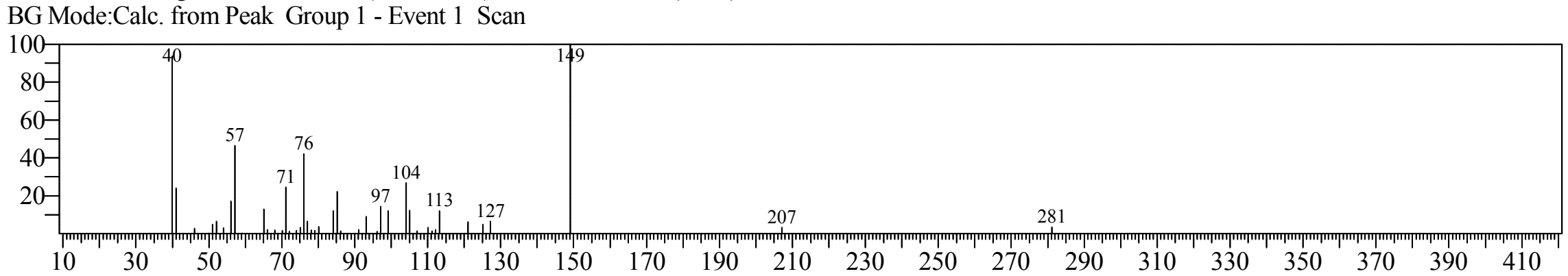

Hit#:16 Entry:201331 Library:NIST11.lib
SI:62 Formula:C30H50O4 CAS:0-00-0 MolWeight:474 RetIndex:3364
CompName:Phthalic acid, isobutyl octadecyl ester

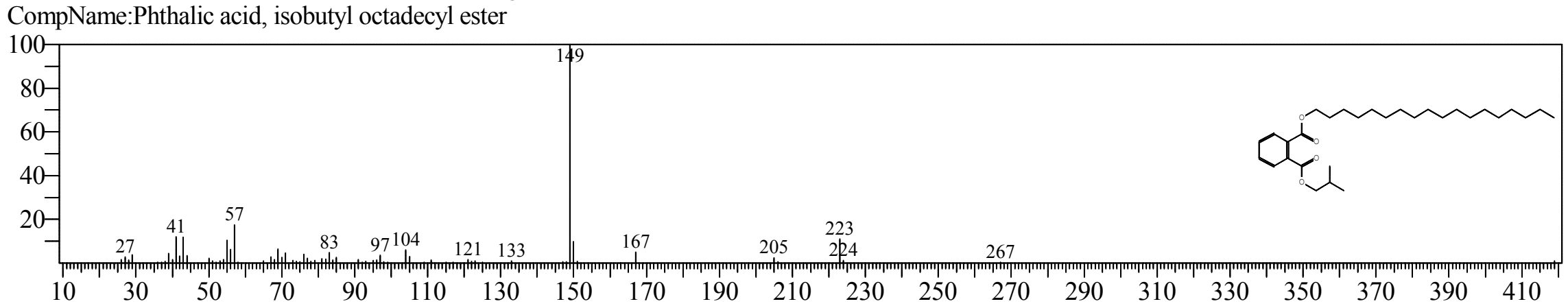

Hit#:17 Entry:155320 Library:NIST05.LIB
SI:62 Formula:C30H50O4 CAS:0-00-0 MolWeight:474 RetIndex:3364
CompName:Phthalic acid, isobutyl octadecyl ester

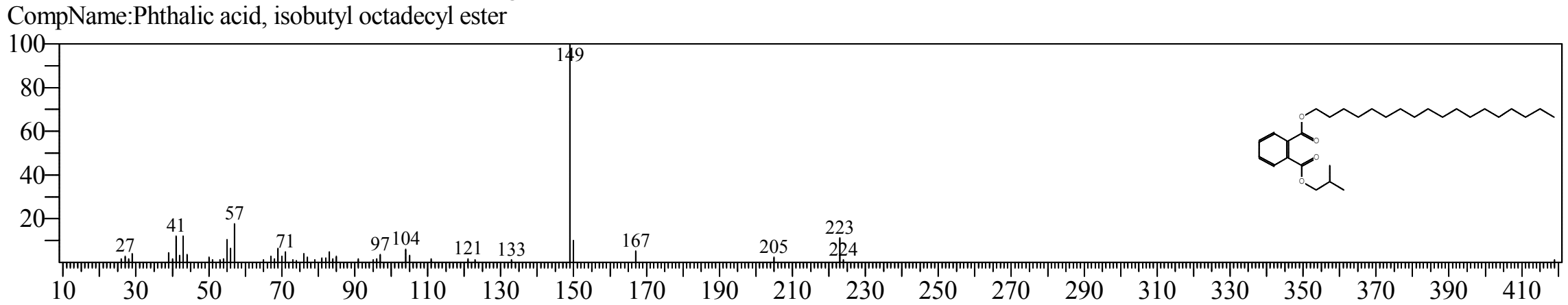

Hit#:18 Entry:204958 Library:NIST11.lib
SI:62 Formula:C32H54O4 CAS:0-00-0 MolWeight:502 RetIndex:3499
CompName:Phthalic acid, 5-methylhex-2-yl heptadecyl ester

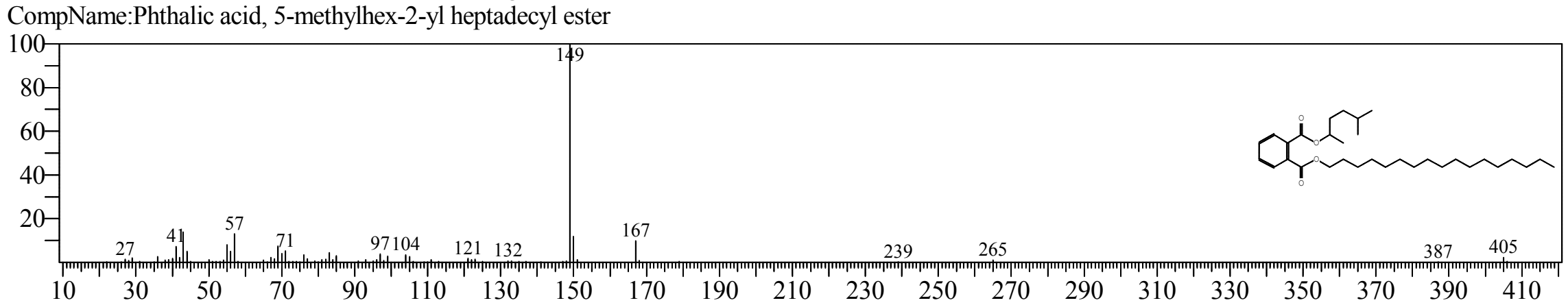

Hit#:19 Entry:188719 Library:NIST11.lib
SI:62 Formula:C26H42O4 CAS:0-00-0 MolWeight:418 RetIndex:2967
CompName:Phthalic acid, decyl 2-ethylhexyl ester

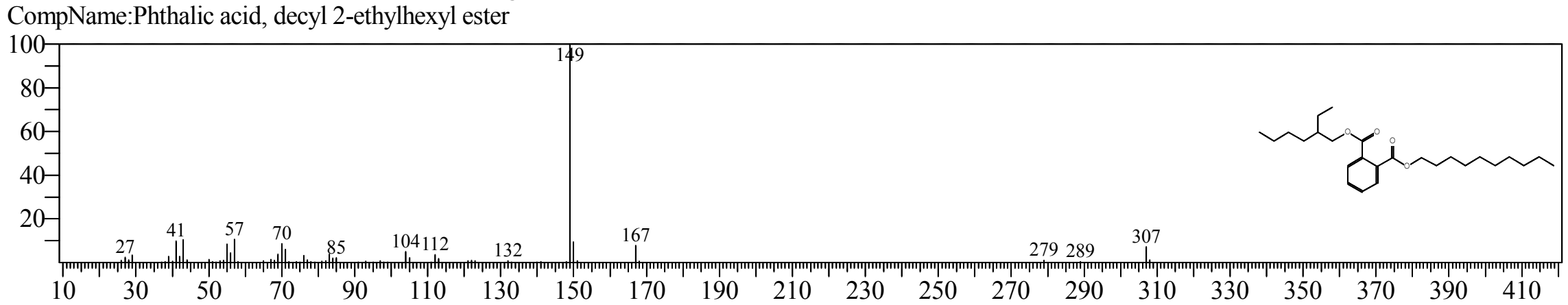

Hit#:20 Entry:148418 Library:NIST05.LIB
SI:62 Formula:C26H42O4 CAS:0-00-0 MolWeight:418 RetIndex:2967
CompName:Phthalic acid, decyl 2-ethylhexyl ester

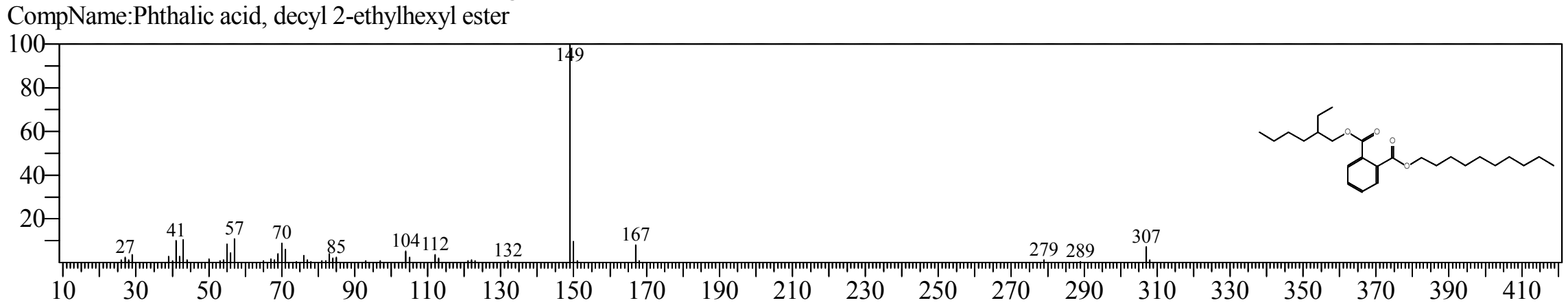

<< Target >>
Line#:10 R.Time:14.167(Scan#:1101) MassPeaks:37
RawMode:Averaged 14.158-14.175(1100-1102) BasePeak:40.00(4561)
BG Mode:Calc. from Peak Group 1 - Event 1 Scan

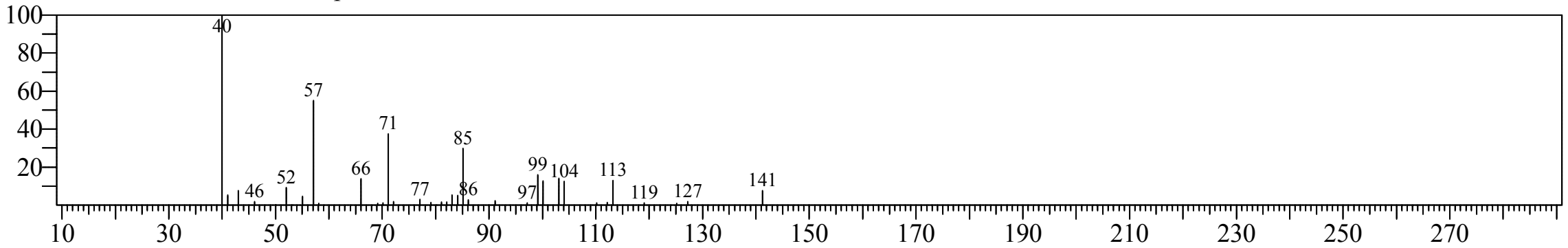

Hit#:1 Entry:17327 Library:NIST11s.lib
SI:70 Formula:C14H30 CAS:7225-67-4 MolWeight:198 RetIndex:1095
CompName:Heptane, 2,2,3,3,5,6,6-heptamethyl- $$ 2,2,3,3,5,6,6-Heptamethylheptane $$

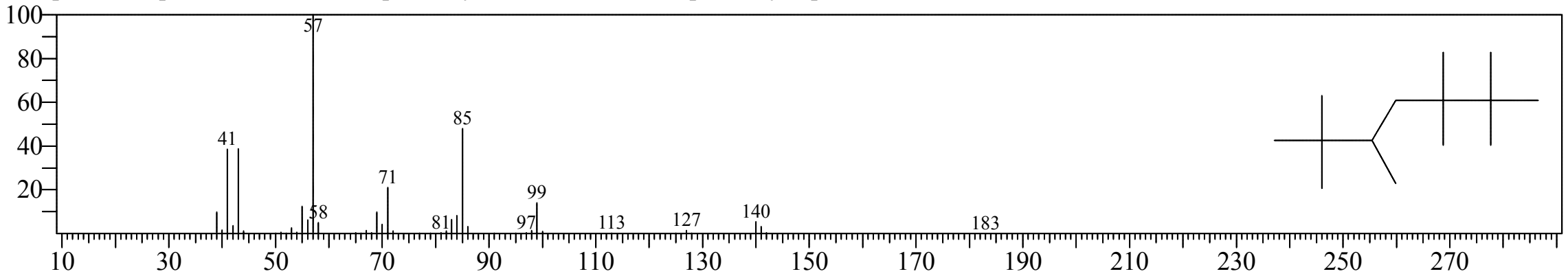

Hit#:2 Entry:178178 Library:WILEY7.LIB
SI:69 Formula:C19 H40 CAS:629-92-5 MolWeight:268 RetIndex:0
CompName:Nonadecane (CAS) n-Nonadecane $$

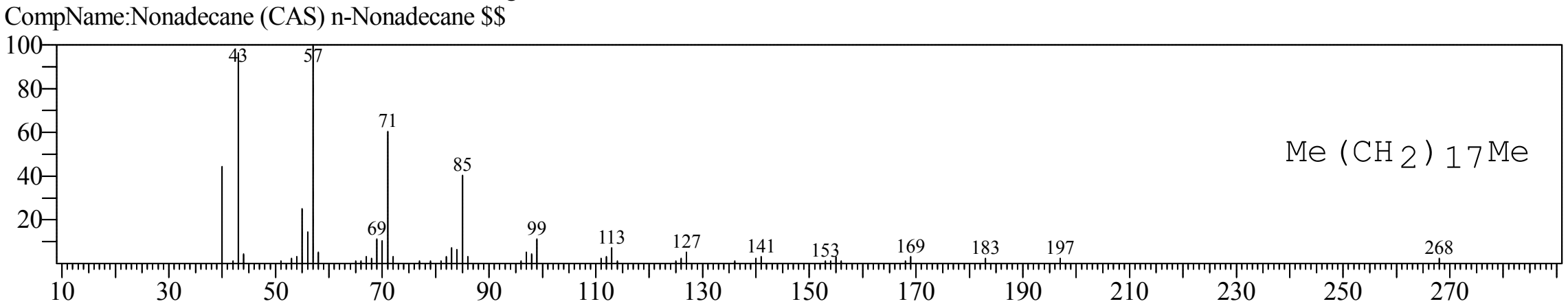

Hit#:3 Entry:24987 Library:NIST05.LIB
SI:69 Formula:C12H26 CAS:17312-55-9 MolWeight:170 RetIndex:1086
CompName:Decane, 3,8-dimethyl- $$ 3,8-Dimethyldecane $$

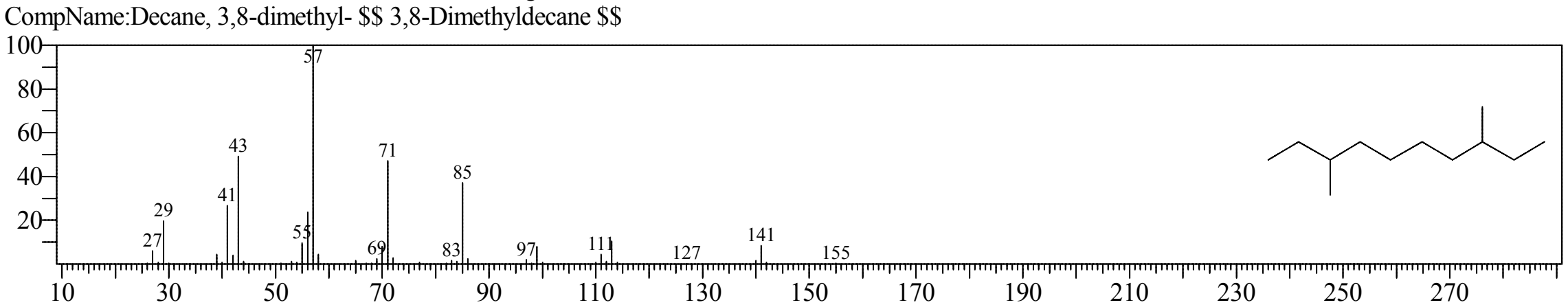

Hit#:4 Entry:62333 Library:NIST11.lib
SI:68 Formula:C14H31BO CAS:0-00-0 MolWeight:226 RetIndex:0
CompName:Borane, diethyl(decyloxy)- $$ Decyl diethylborinate # $$

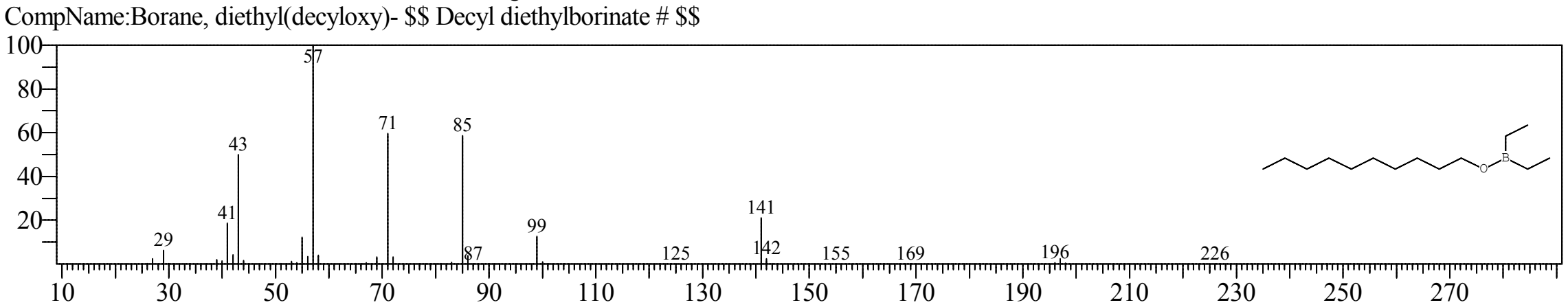

Hit#:5 Entry:128619 Library:WILEY7.LIB
SI:68 Formula:C14 H31 B O CAS:0-00-0 MolWeight:226 RetIndex:0
CompName:DIETHYL(DECYLOXY)-BORANE $$

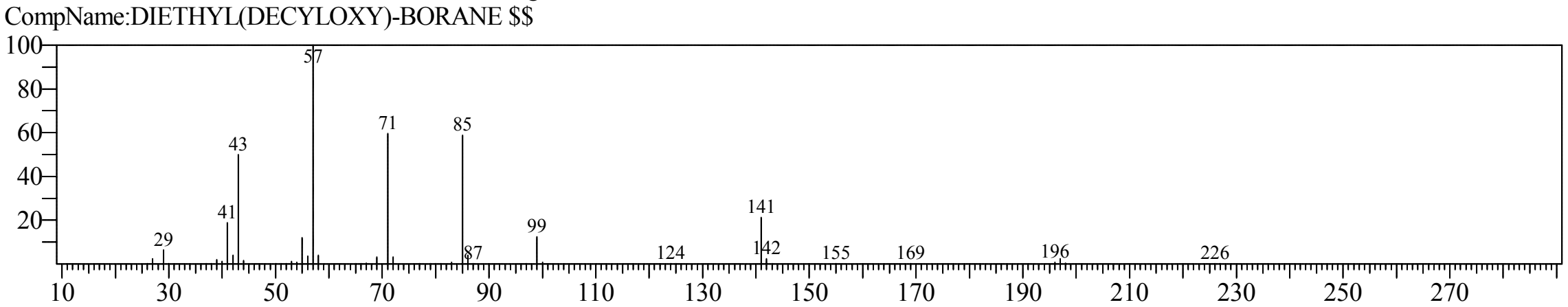

<< Target >>
Line#:10 R.Time:14.167(Scan#:1101) MassPeaks:37
RawMode:Averaged 14.158-14.175(1100-1102) BasePeak:40.00(4561)
BG Mode:Calc. from Peak Group 1 - Event 1 Scan

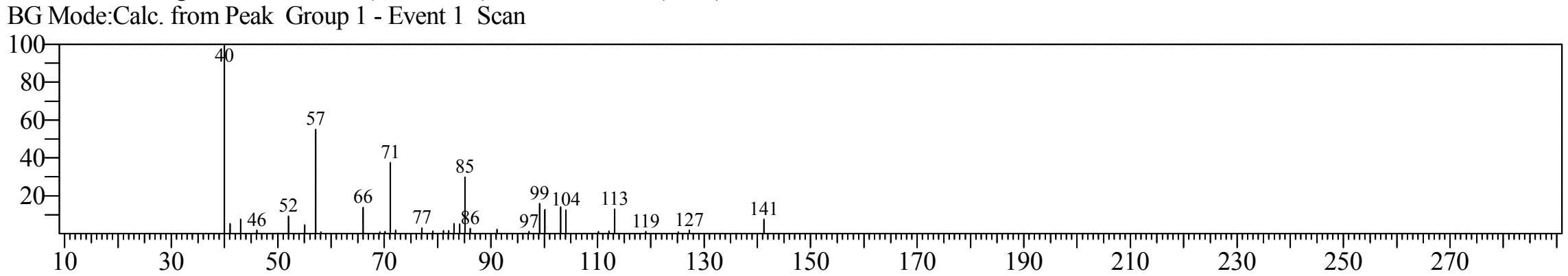

Hit#:6 Entry:20754 Library:NIST11s.lib
SI:68 Formula:C16H34 CAS:4390-04-9 MolWeight:226 RetIndex:1294
CompName:Nonane, 2,2,4,4,6,8,8-heptamethyl- $$ 2,2,4,4,6,8,8-Heptamethylnonane $$ HMN $$ Permethyl 101A $$

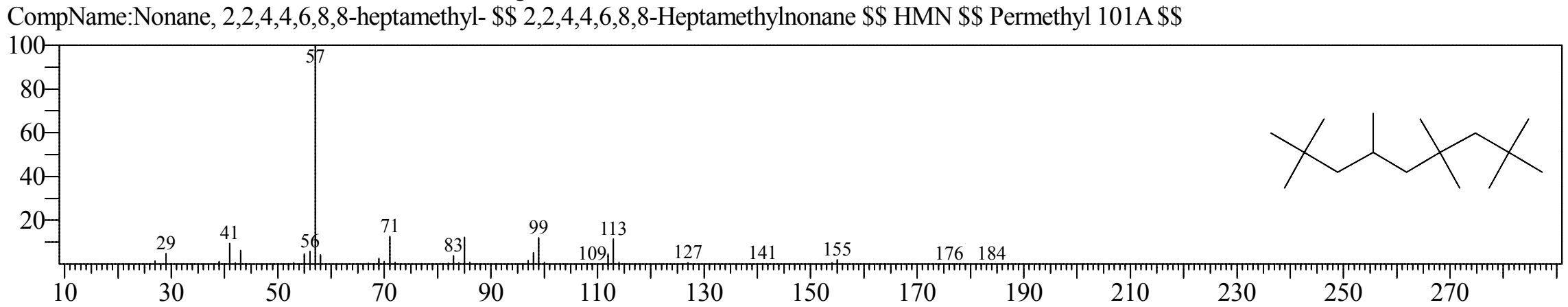

Hit#:7 Entry:57047 Library:NIST05.LIB
SI:68 Formula:C14H31BO CAS:0-00-0 MolWeight:226 RetIndex:0
CompName:Borane, diethyl(decyloxy)-

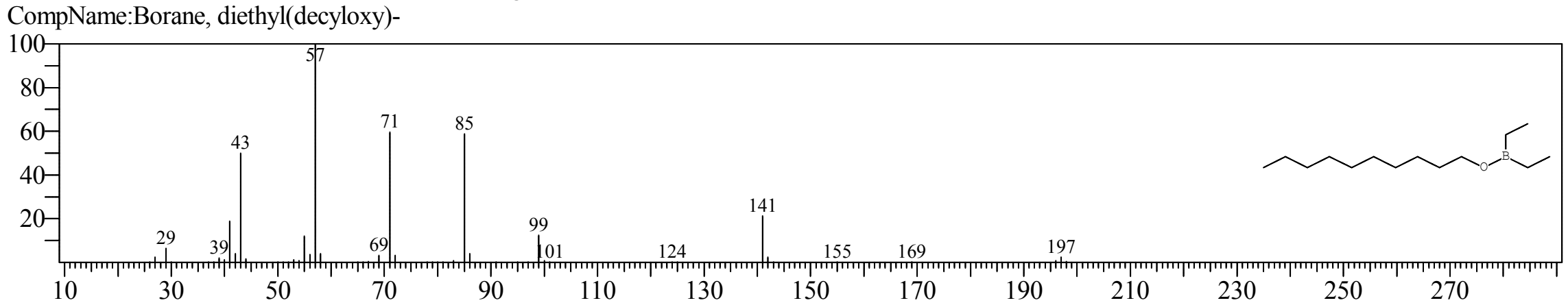

Hit#:8 Entry:32394 Library:NIST05.LIB
SI:68 Formula:C13H28 CAS:62238-11-3 MolWeight:184 RetIndex:1121
CompName:Decane, 2,3,5-trimethyl- $$ 2,3,5-Trimethyldecane # $$

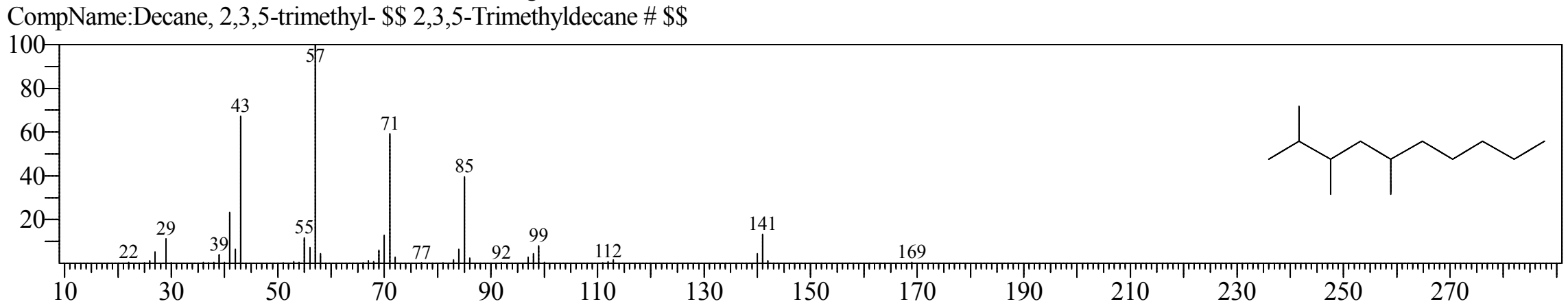

Hit#:9 Entry:77108 Library:WILEY7.LIB
SI:68 Formula:C13 H28 CAS:17301-33-6 MolWeight:184 RetIndex:0
CompName:Undecane, 4,8-dimethyl- (CAS)

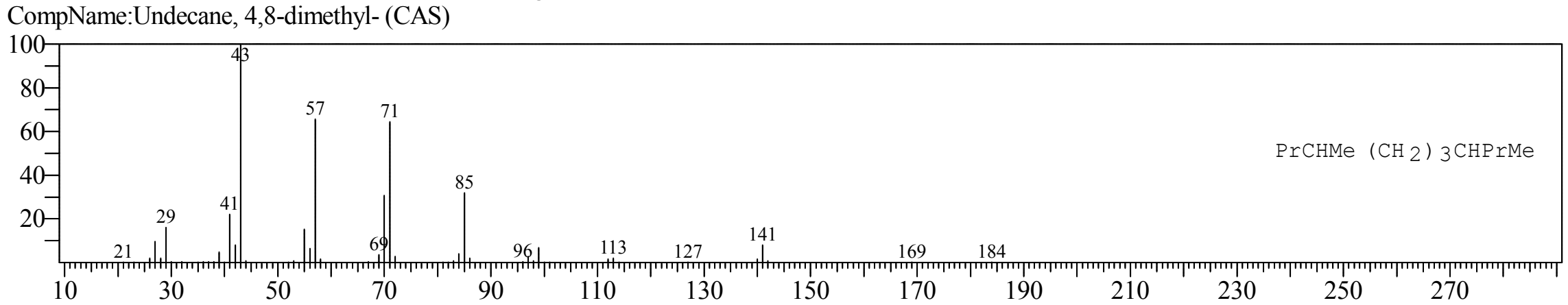

Hit#:10 Entry:40273 Library:NIST05.LIB
SI:68 Formula:C14H30 CAS:107770-99-0 MolWeight:198 RetIndex:1285
CompName:3,5-Dimethyldodecane

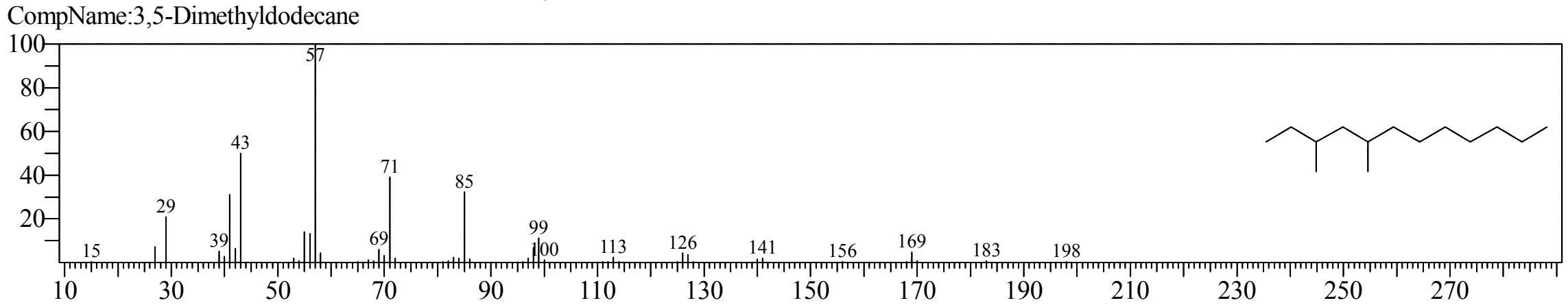

<< Target >>
Line#:10   R.Time:14.167(Scan#:1101) MassPeaks:37
RawMode:Averaged 14.158-14.175(1100-1102) BasePeak:40.00(4561)
BG Mode:Calc. from Peak Group 1 - Event 1 Scan

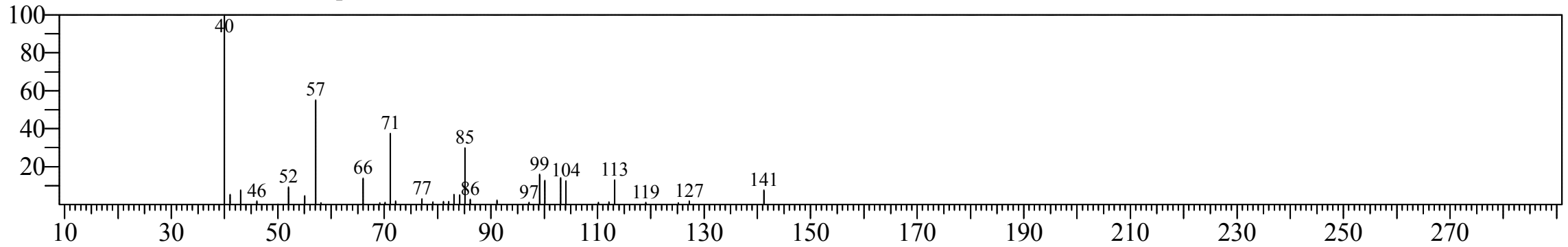

Hit#:11 Entry:103707 Library:NIST11.lib
SI:67 Formula:C11H23I CAS:4282-44-4 MolWeight:282 RetIndex:1529
CompName:1-Iodoundecane $$ Undecane, 1-iodo- $$

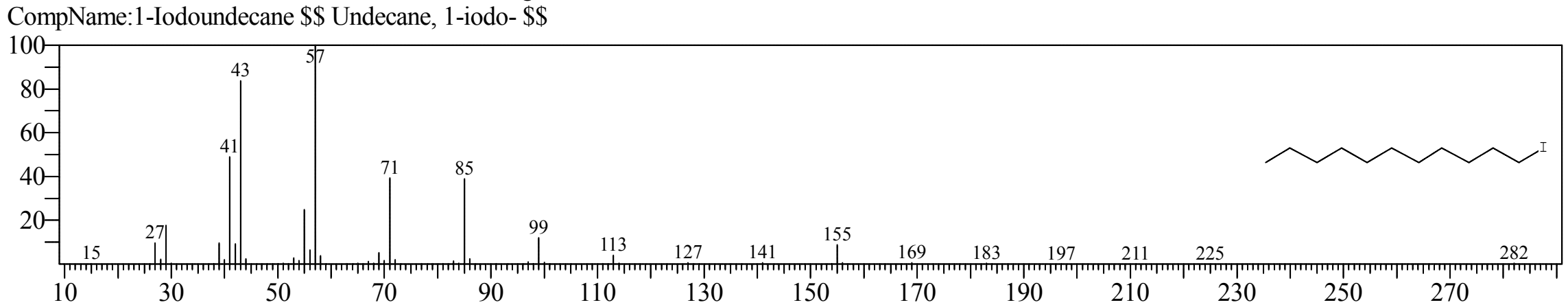

Hit#:12 Entry:23542 Library:NIST05s.LIB
SI:67 Formula:C12H25I CAS:4292-19-7 MolWeight:296 RetIndex:1628
CompName:Dodecane, 1-iodo- $$ n-Dodecyl iodide $$ Dodecyl iodide $$ Lauryl iodide $$ 1-Iodododecane $$

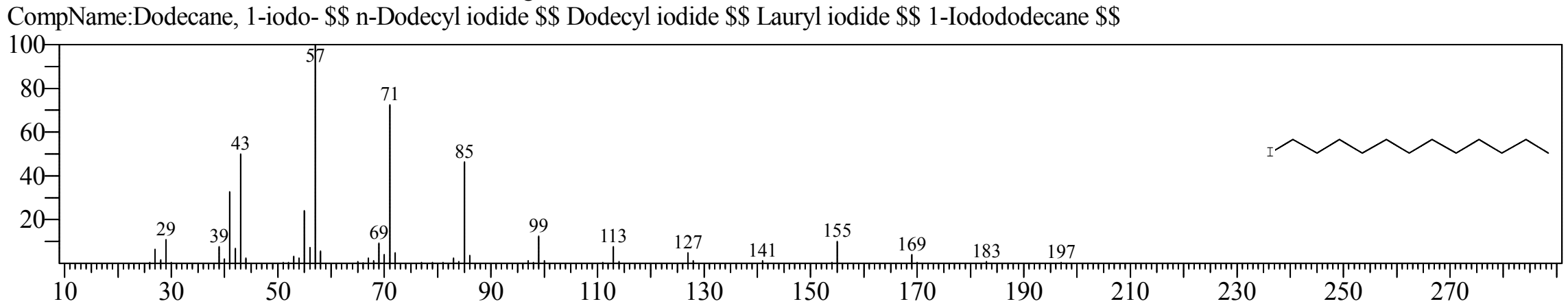

Hit#:13 Entry:12009 Library:NIST05s.LIB
SI:67 Formula:C12H26 CAS:1002-43-3 MolWeight:170 RetIndex:1150
CompName:Undecane, 3-methyl- $$ 3-Methylundecane $$

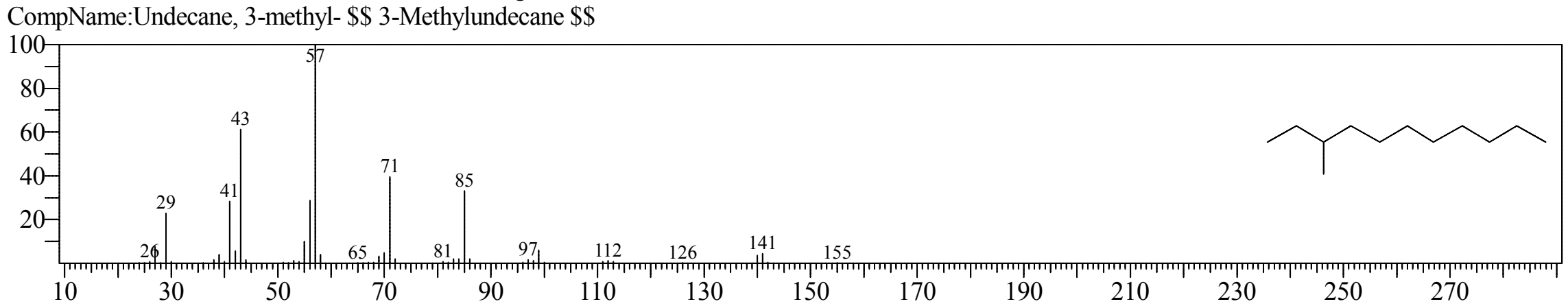

Hit#:14 Entry:18439 Library:NIST11.lib
SI:67 Formula:C9H16O2 CAS:0-00-0 MolWeight:156 RetIndex:1021
CompName:2,2-Dimethylpropanoic acid, cyclobutyl ester $$ Cyclobutyl pivalate # $$

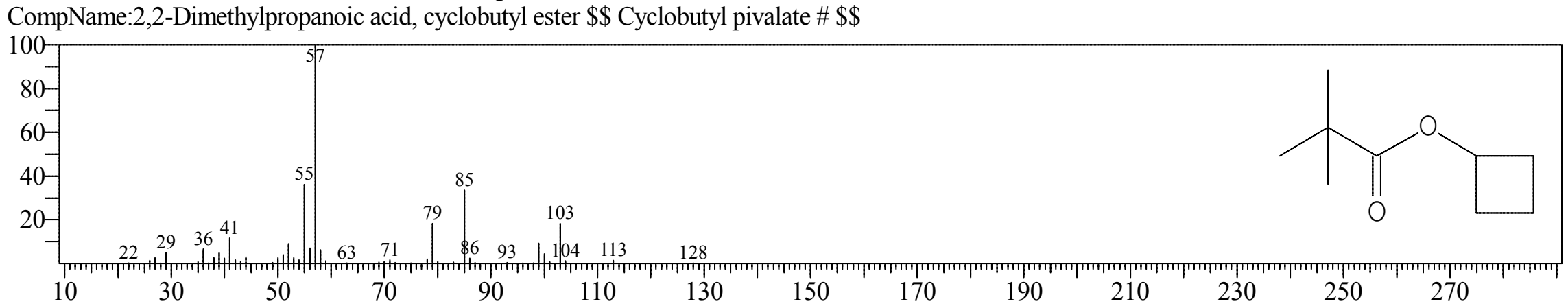

Hit#:15 Entry:17952 Library:NIST05.LIB
SI:67 Formula:C10H20O CAS:0-00-0 MolWeight:156 RetIndex:959
CompName:3-Isopropyl-5-methylhexan-2-one

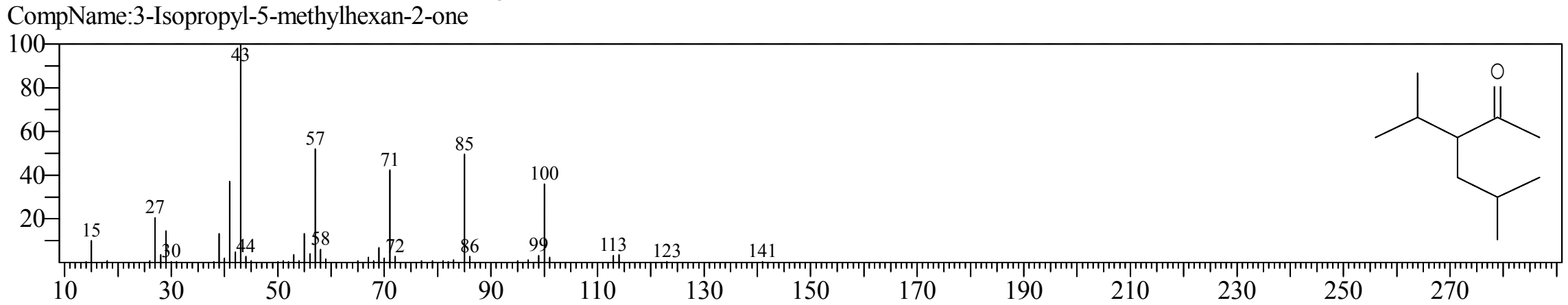

<< Target >>
Line#:10   R.Time:14.167(Scan#:1101) MassPeaks:37
RawMode:Averaged 14.158-14.175(1100-1102) BasePeak:40.00(4561)
BG Mode:Calc. from Peak Group 1 - Event 1 Scan

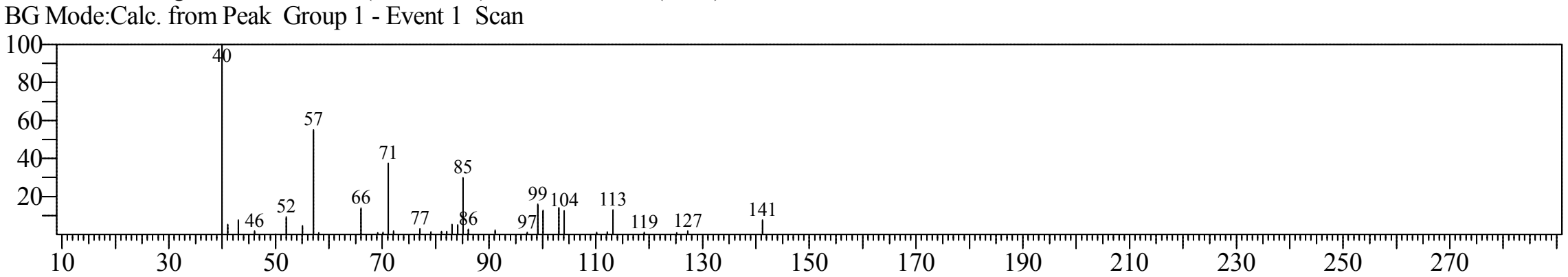

Hit#:16 Entry:17810 Library:NIST05.LIB
SI:67 Formula:C9H16O2 CAS:0-00-0 MolWeight:156 RetIndex:1021
CompName:2,2-Dimethylpropanoic acid, cyclobutyl ester

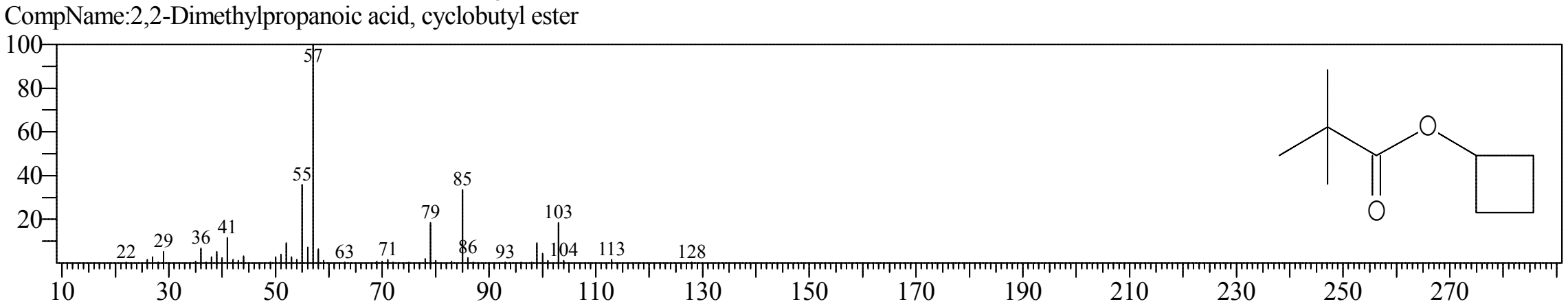

Hit#:17 Entry:18584 Library:NIST11.lib
SI:67 Formula:C10H20O CAS:0-00-0 MolWeight:156 RetIndex:959
CompName:3-Isopropyl-5-methylhexan-2-one $$ 3-Isopropyl-5-methyl-2-hexanone # $$

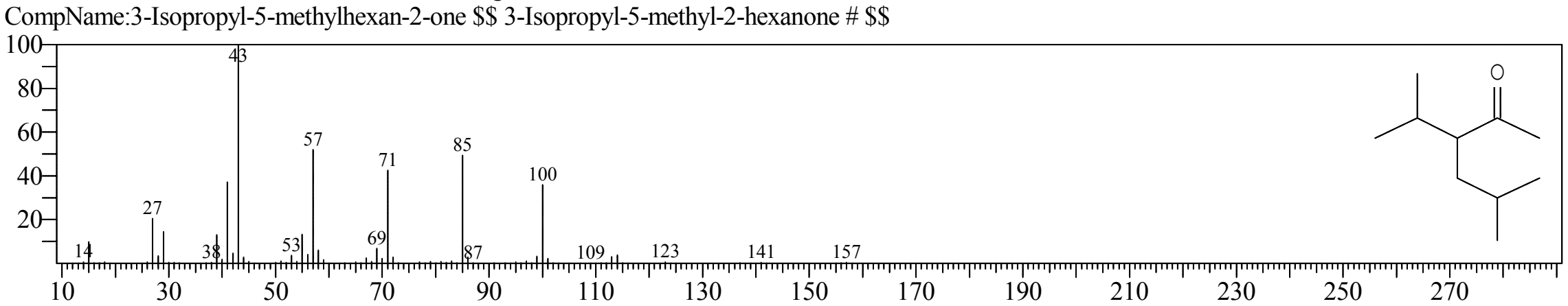

Hit#:18 Entry:45568 Library:WILEY7.LIB
SI:67 Formula:C10 H20 O CAS:0-00-0 MolWeight:156 RetIndex:0
CompName:3-I-PROPYL-5-METHYL-HEXAN-2-ON $$

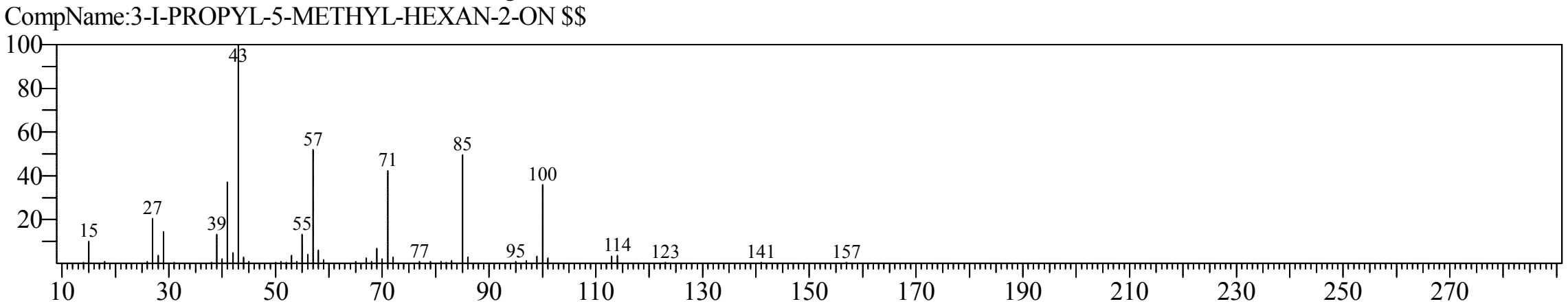

Hit#:19 Entry:24986 Library:NIST05.LIB
SI:67 Formula:C12H26 CAS:17312-53-7 MolWeight:170 RetIndex:1086
CompName:Decane, 3,6-dimethyl- $$ 3,6-Dimethyldecane $$

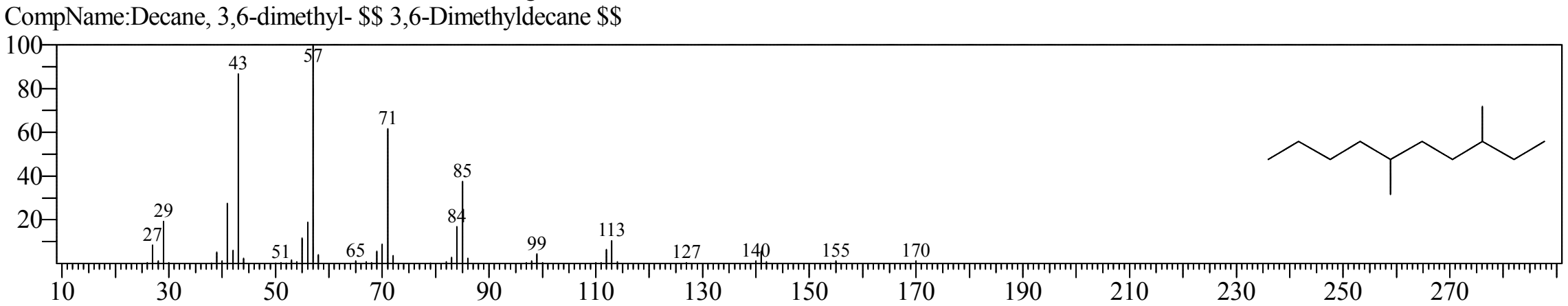

Hit#:20 Entry:76406 Library:WILEY7.LIB
SI:67 Formula:C13 H28 CAS:17301-24-5 MolWeight:184 RetIndex:0
CompName:Undecane, 2,7-dimethyl- (CAS)

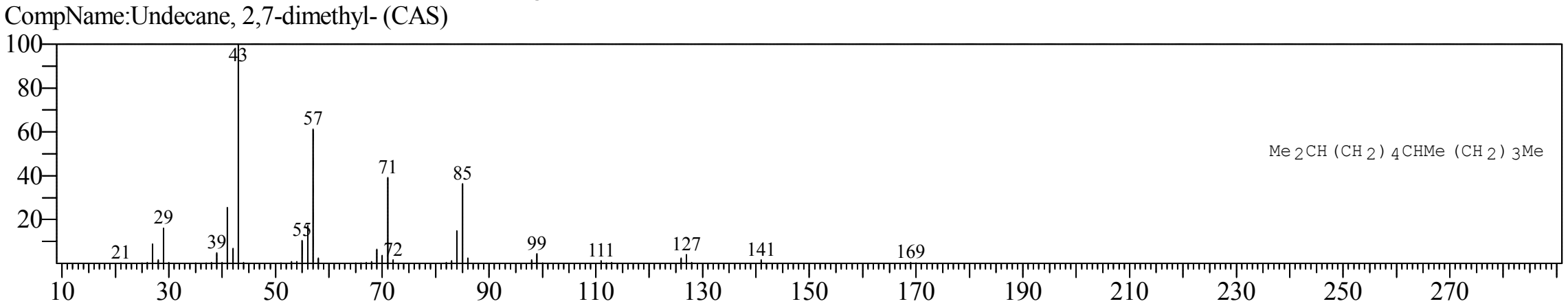

<< Target >>
Line#:11 R.Time:14.250(Scan#:1111) MassPeaks:51
RawMode:Averaged 14.242-14.258(1110-1112) BasePeak:39.95(1595)
BG Mode:Calc. from Peak Group 1 - Event 1 Scan

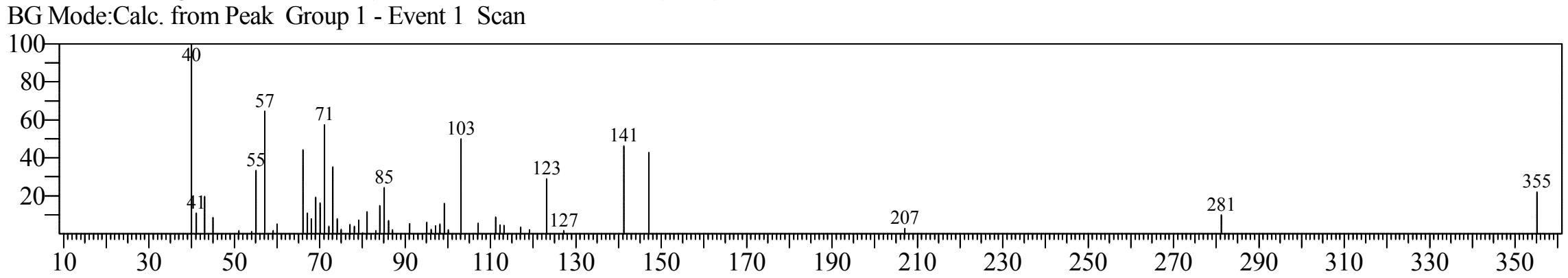

Hit#:1 Entry:113186 Library:NIST05.LIB
SI:65 Formula:C16H34O6 CAS:132591-07-2 MolWeight:322 RetIndex:2596
CompName:d-Glucitol, 4-O-decyl- $$ 4-O-Decyl-d-glucitol # $$

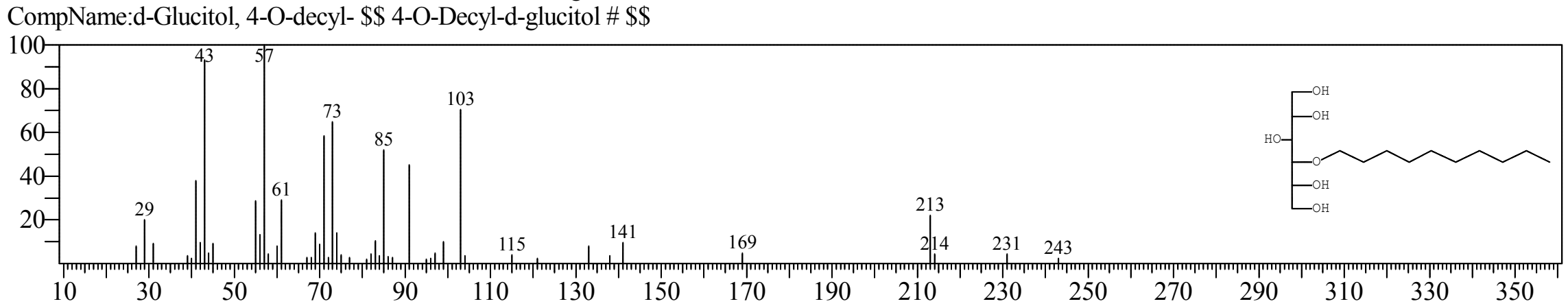

Hit#:2 Entry:170104 Library:WILEY7.LIB
SI:64 Formula:C13 H26 O5 CAS:0-00-0 MolWeight:262 RetIndex:0
CompName:D-MANNOTRIDEC-6-EN-1,2,3,4,5-PENTAOL $$

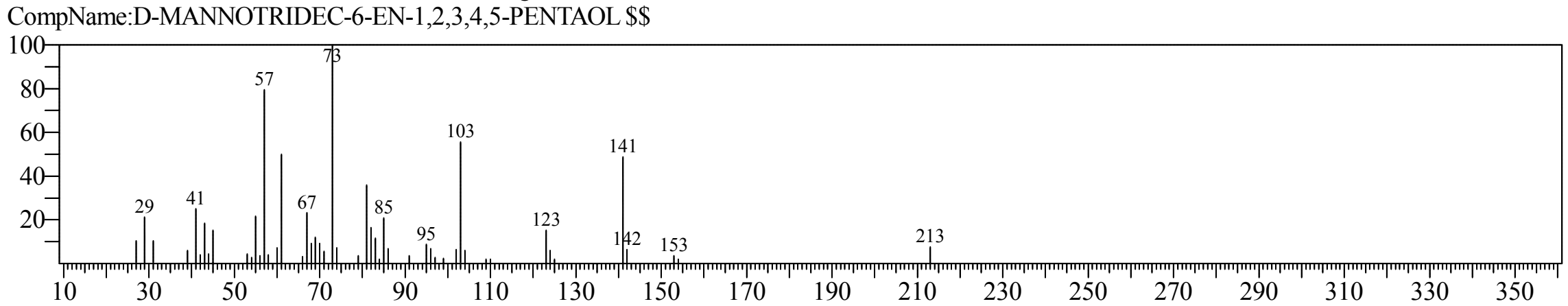

Hit#:3 Entry:88565 Library:NIST11.lib
SI:64 Formula:C13H26O5 CAS:0-00-0 MolWeight:262 RetIndex:2213
CompName:D-Mannotridec-6-ene-1,2,3,4,5-pentaol $$ (6E)-6-Tridecene-1,2,3,4,5-pentol # $$

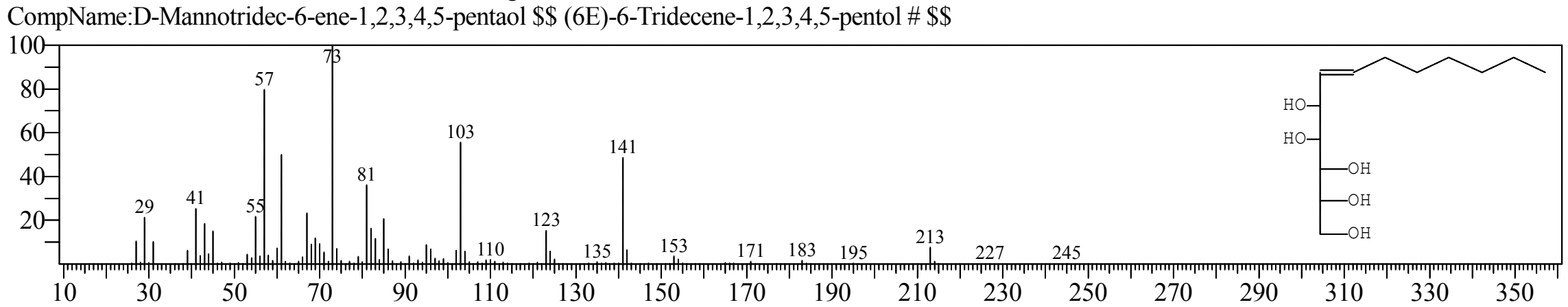

Hit#:4 Entry:78204 Library:NIST05.LIB
SI:64 Formula:C13H26O5 CAS:0-00-0 MolWeight:262 RetIndex:2213
CompName:D-Mannotridec-6-ene-1,2,3,4,5-pentaol

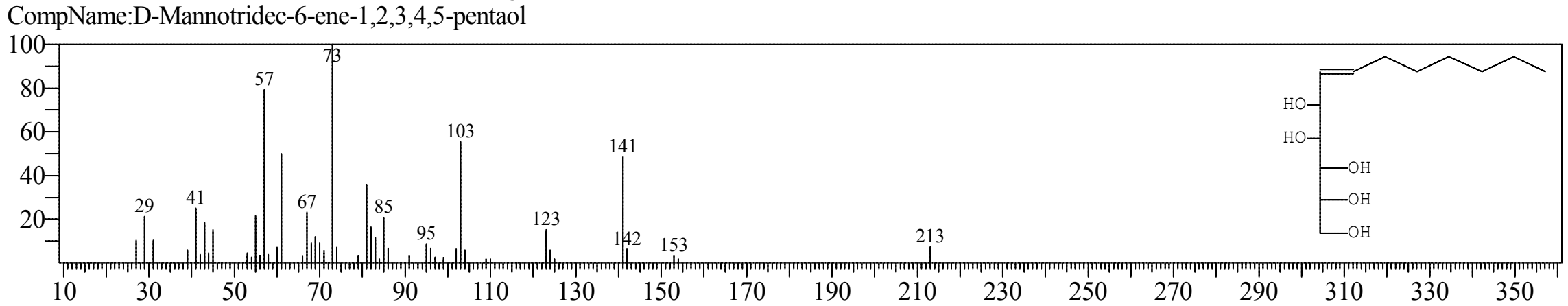

Hit#:5 Entry:107453 Library:NIST11.lib
SI:64 Formula:C16H30O4 CAS:0-00-0 MolWeight:286 RetIndex:1754
CompName:Oxalic acid, 6-ethyloct-3-yl isobutyl ester

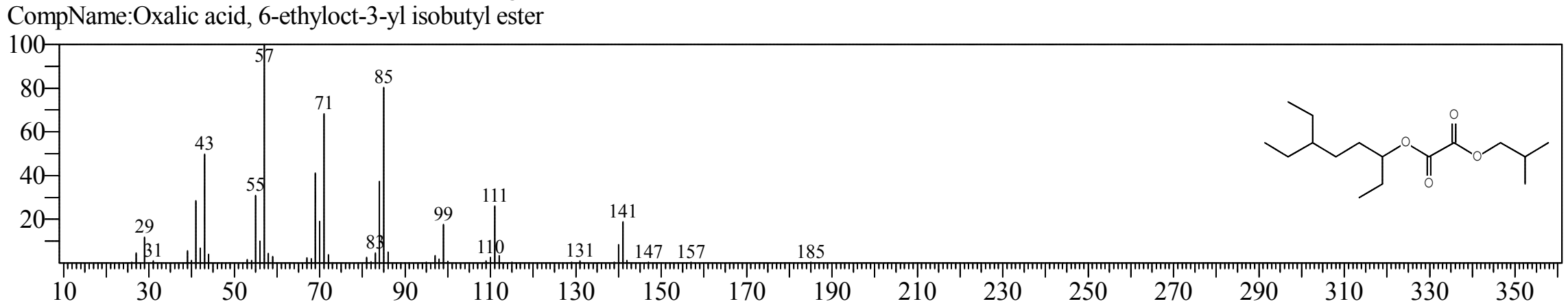

<< Target >>
Line#:11 R.Time:14.250(Scan#:1111) MassPeaks:51
RawMode:Averaged 14.242-14.258(1110-1112) BasePeak:39.95(1595)
BG Mode:Calc. from Peak Group 1 - Event 1 Scan

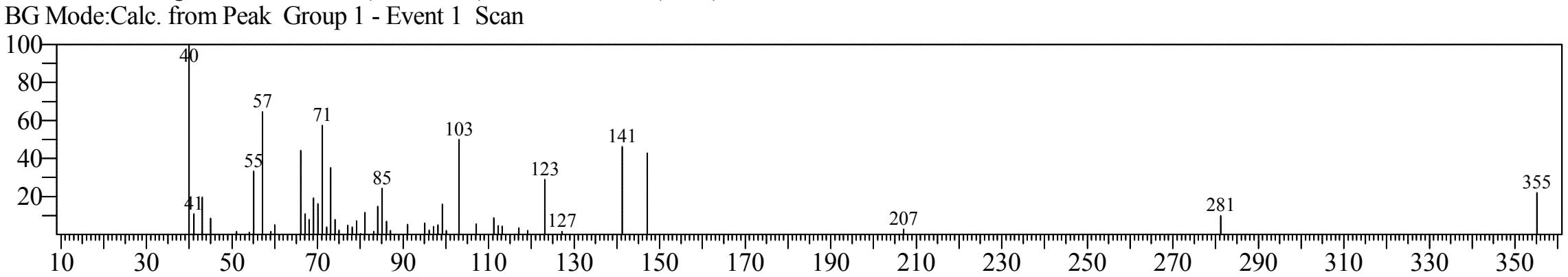

Hit#:6 Entry:92893 Library:NIST05.LIB
SI:64 Formula:C16H30O4 CAS:0-00-0 MolWeight:286 RetIndex:1754
CompName:Oxalic acid, 6-ethyloct-3-yl isobutyl ester

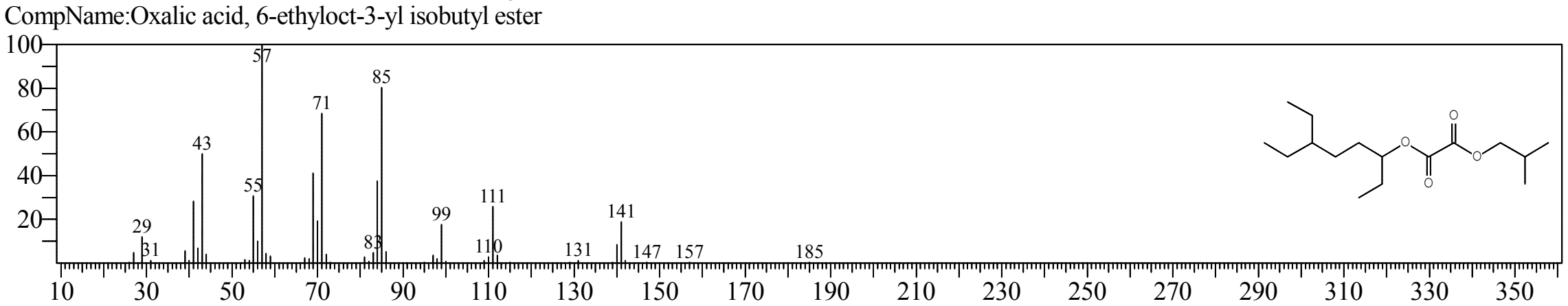

Hit#:7 Entry:65030 Library:NIST11.lib
SI:63 Formula:C13H26O3 CAS:0-00-0 MolWeight:230 RetIndex:1428
CompName:Methoxyacetic acid, 6-ethyl-3-octyl ester $$ 1,4-Diethylhexyl methoxyacetate # $$

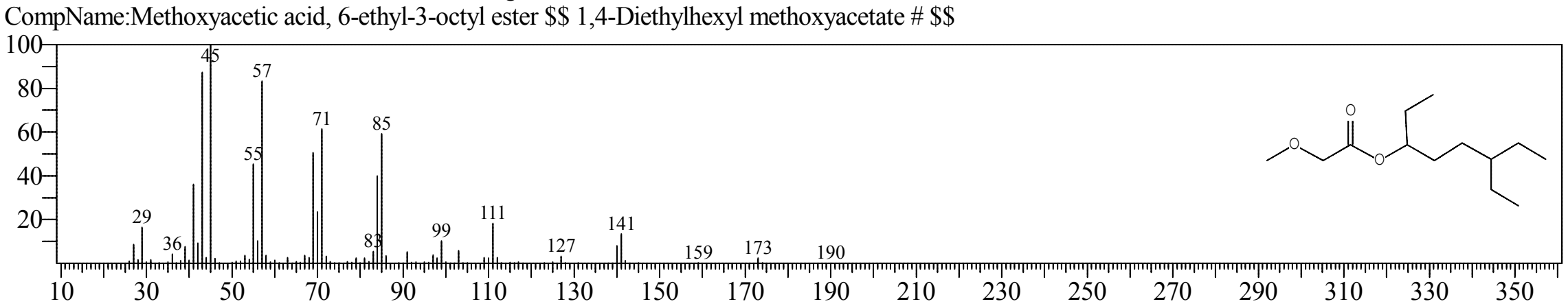

Hit#:8 Entry:59289 Library:NIST05.LIB
SI:63 Formula:C13H26O3 CAS:0-00-0 MolWeight:230 RetIndex:1428
CompName:Methoxyacetic acid, 6-ethyl-3-octyl ester

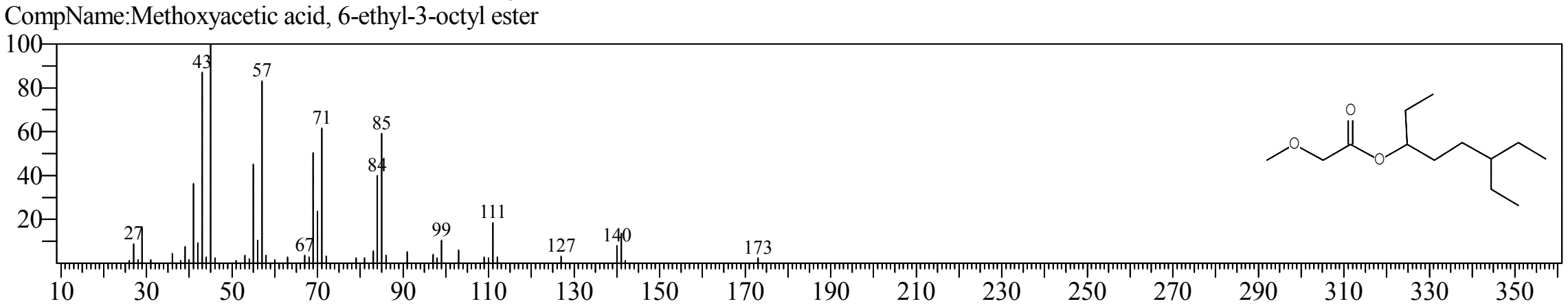

Hit#:9 Entry:268066 Library:WILEY7.LIB
SI:63 Formula:C23 H50 O SI CAS:78695-20-2 MolWeight:370 RetIndex:0
CompName:3,7,11,15-TETRAMETHYLHEXADECAN-1-OL TRIMETHYLSILYL ETHER $$ Silane, trimethyl[(3,7,11,15-tetramethylhexadecyl)oxy]- (C

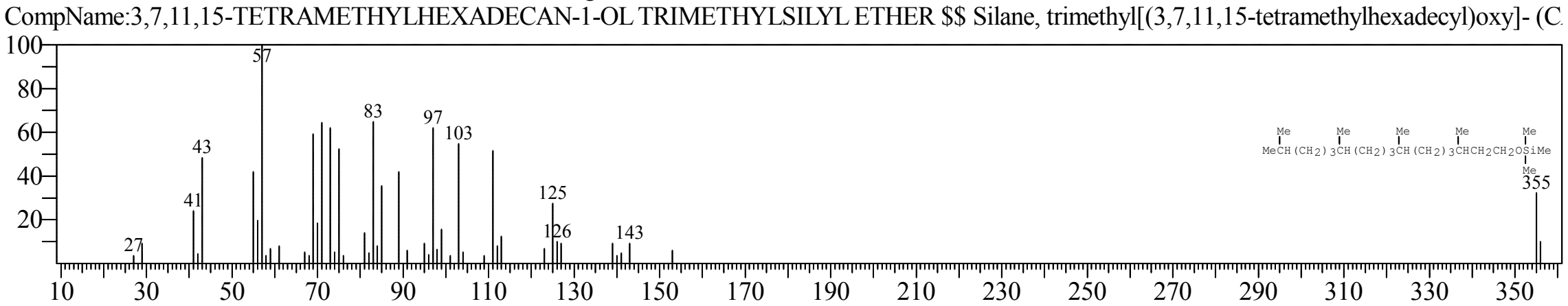

Hit#:10 Entry:90581 Library:WILEY7.LIB
SI:63 Formula:C13 H24 O CAS:0-00-0 MolWeight:196 RetIndex:0
CompName:

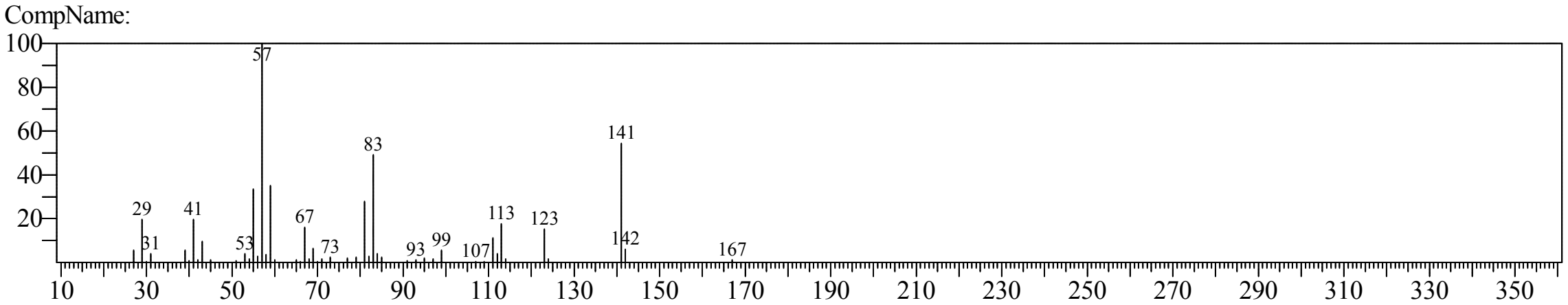

<< Target >>
Line#:11 R.Time:14.250(Scan#:1111) MassPeaks:51
RawMode:Averaged 14.242-14.258(1110-1112) BasePeak:39.95(1595)
BG Mode:Calc. from Peak Group 1 - Event 1 Scan

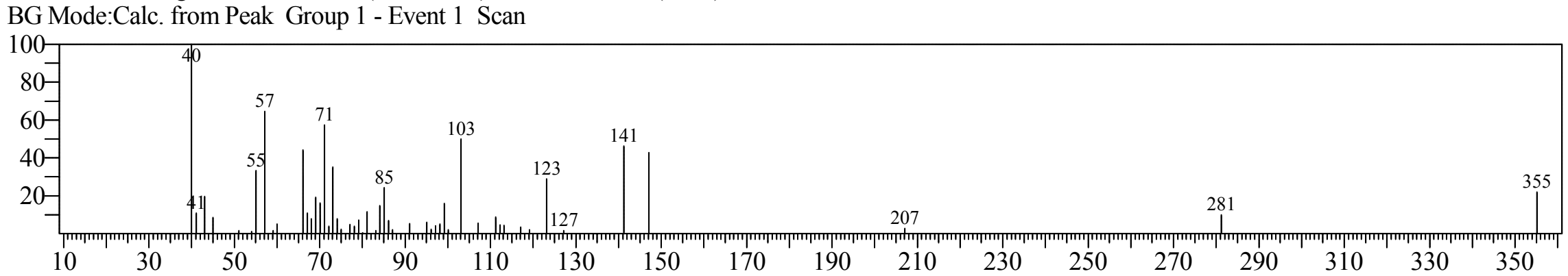

Hit#:11 Entry:41589 Library:NIST11.lib
SI:63 Formula:C13H24O CAS:344308-85-6 MolWeight:196 RetIndex:1395
CompName:1-Hexen-4-ol, 4-cyclohexyl-3-methyl- $$ 3-Cyclohexyl-4-methyl-5-hexen-3-ol # $$

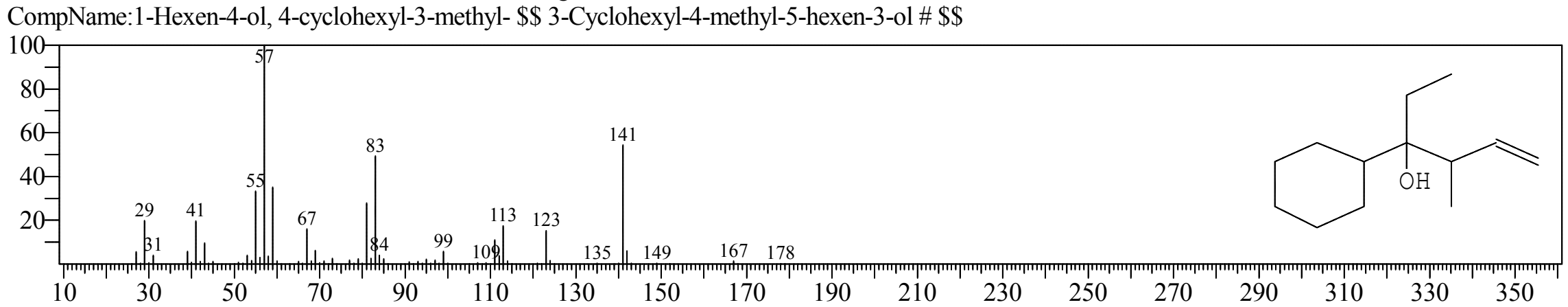

Hit#:12 Entry:107454 Library:NIST11.lib
SI:63 Formula:C16H30O4 CAS:0-00-0 MolWeight:286 RetIndex:1818
CompName:Oxalic acid, butyl 6-ethyloct-3-yl ester

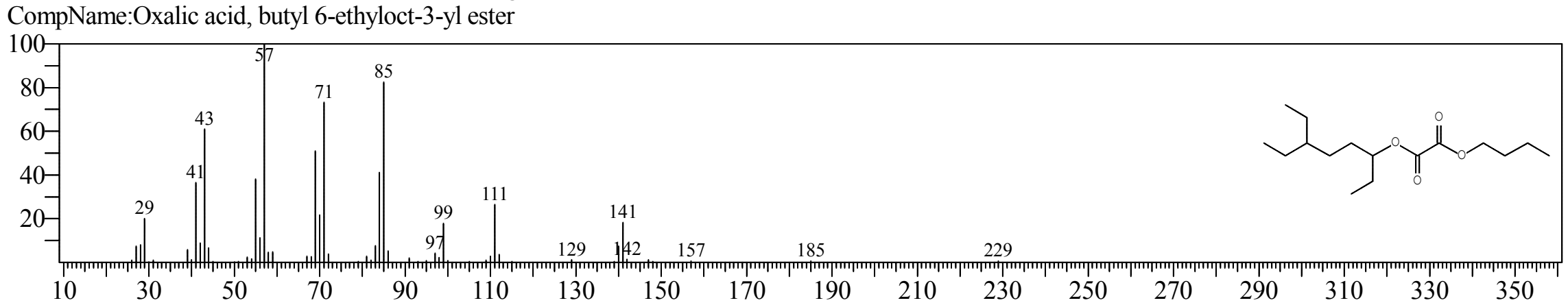

Hit#:13 Entry:92894 Library:NIST05.LIB
SI:63 Formula:C16H30O4 CAS:0-00-0 MolWeight:286 RetIndex:1818
CompName:Oxalic acid, butyl 6-ethyloct-3-yl ester

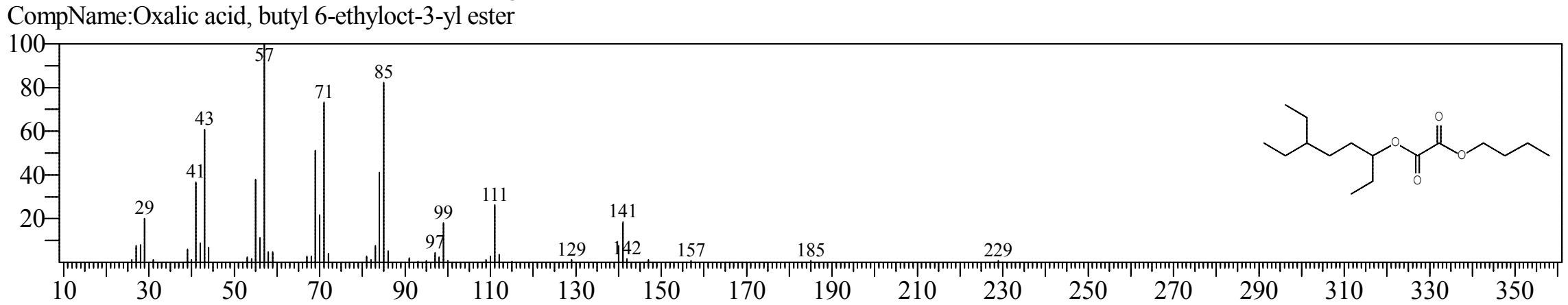

Hit#:14 Entry:15972 Library:NIST05s.LIB
SI:63 Formula:C12H22O2 CAS:7493-72-3 MolWeight:198 RetIndex:1371
CompName:Allyl nonanoate $$ Allyl pelargonate $$ Nonanoic acid, 2-propenyl ester $$ Nonanoic acid, allyl ester $$ Allyl n-nonanoate $$

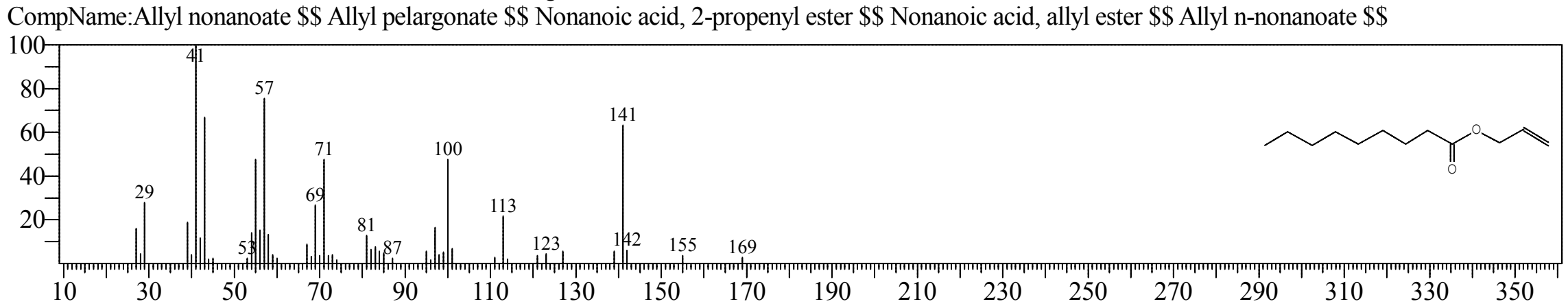

Hit#:15 Entry:26011 Library:NIST05.LIB
SI:63 Formula:C11H24O CAS:54461-00-6 MolWeight:172 RetIndex:1273
CompName:1-Hexanol, 2-ethyl-2-propyl- $$ 2-Ethyl-2-propyl-1-hexanol $$

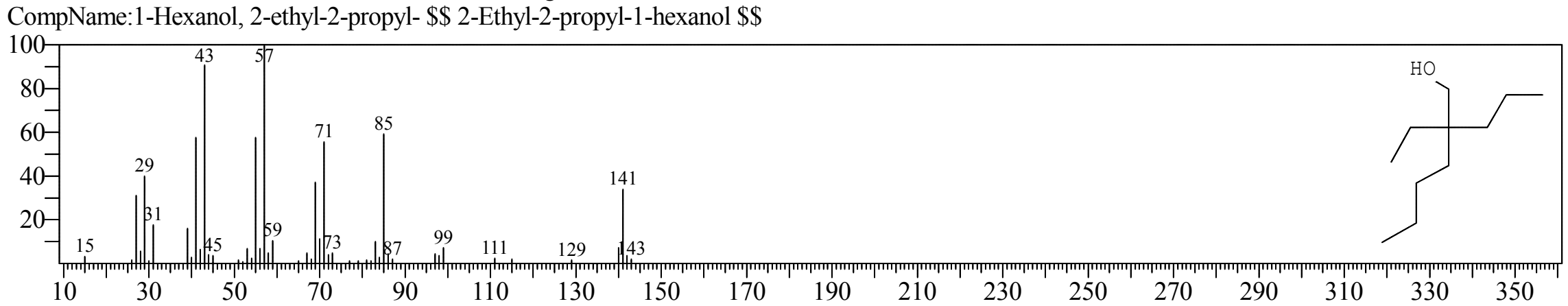

<< Target >>
Line#:11 R.Time:14.250(Scan#:1111) MassPeaks:51
RawMode:Averaged 14.242-14.258(1110-1112) BasePeak:39.95(1595)
BG Mode:Calc. from Peak Group 1 - Event 1 Scan

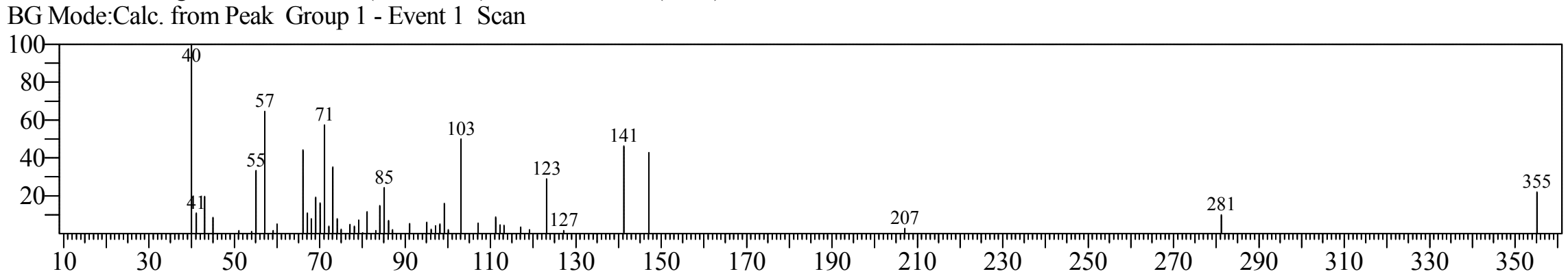

Hit#:16 Entry:145947 Library:WILEY7.LIB
SI:62 Formula:C17 H36 CAS:14905-56-7 MolWeight:240 RetIndex:0
CompName:Tetradecane, 2,6,10-trimethyl- (CAS) 2,6,10-Trimethyltetradecane $$

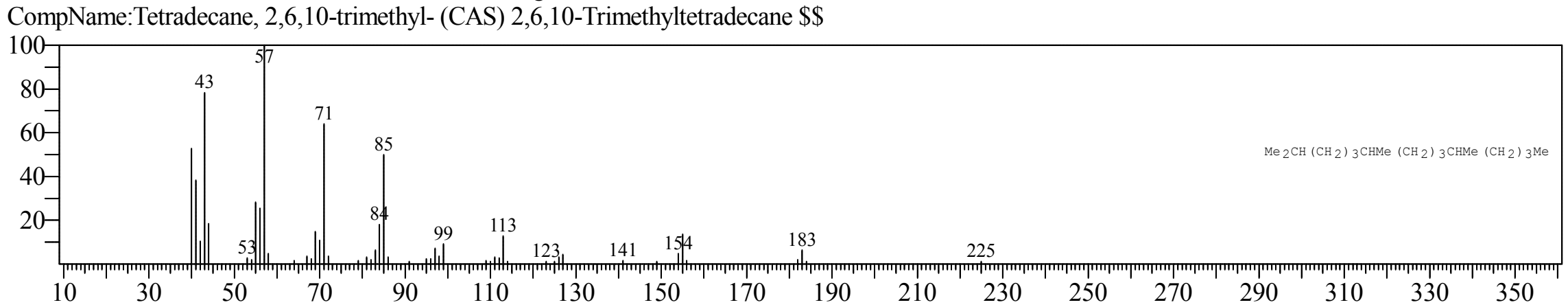

Hit#:17 Entry:24272 Library:NIST05s.LIB
SI:62 Formula:C19H36O3 CAS:2380-18-9 MolWeight:312 RetIndex:2213
CompName:Octadecanoic acid, 2-oxo-, methyl ester $$ Methyl 2-oxooctadecanoate # $$

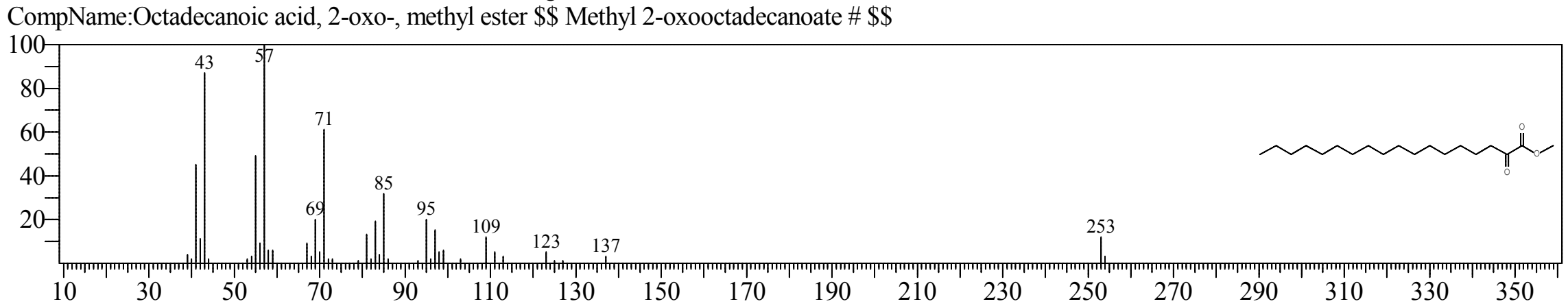

Hit#:18 Entry:178178 Library:WILEY7.LIB
SI:62 Formula:C19 H40 CAS:629-92-5 MolWeight:268 RetIndex:0
CompName:Nonadecane (CAS) n-Nonadecane $$

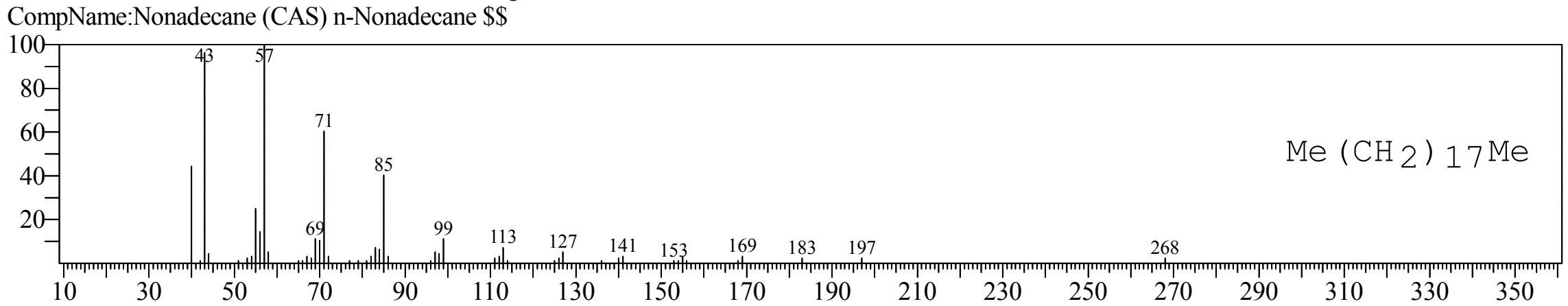

Hit#:19 Entry:101153 Library:NIST05.LIB
SI:62 Formula:C18H36O3 CAS:0-00-0 MolWeight:300 RetIndex:1990
CompName:Methoxyacetic acid, 3-pentadecyl ester

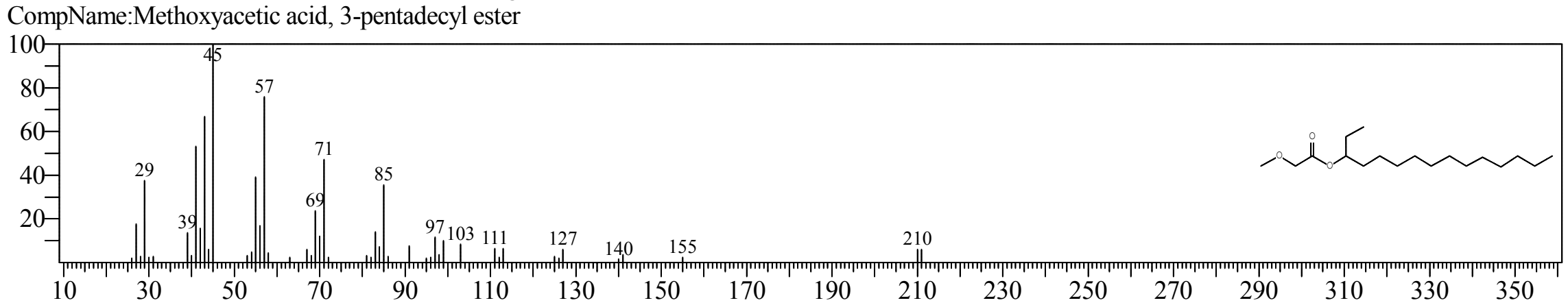

Hit#:20 Entry:62440 Library:NIST11.lib
SI:62 Formula:C15H30O CAS:18787-66-1 MolWeight:226 RetIndex:1648
CompName:3-Pentadecanone

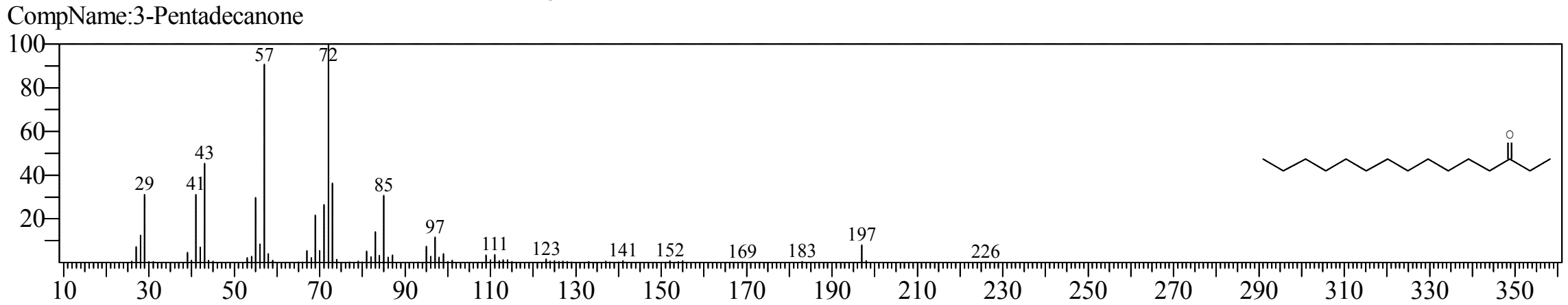

<< Target >>
Line#:12   R.Time:14.467(Scan#:1137) MassPeaks:58
RawMode:Averaged 14.458-14.475(1136-1138) BasePeak:57.10(15653)
BG Mode:Calc. from Peak Group 1 - Event 1 Scan

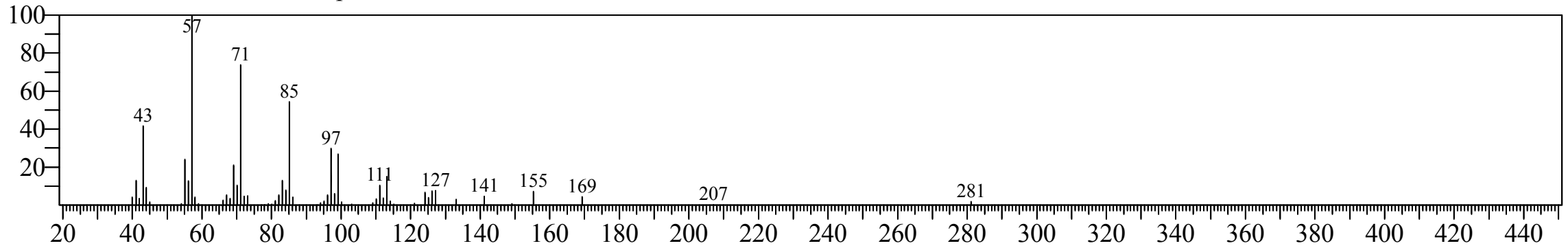

Hit#:1 Entry:173393 Library:NIST11.lib
SI:91 Formula:C27H56 CAS:0-00-0 MolWeight:380 RetIndex:2641
CompName:2-methylhexacosane

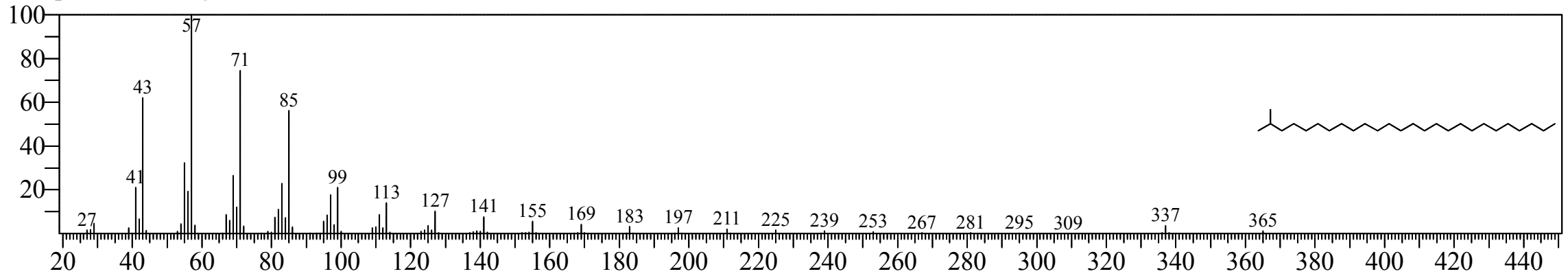

Hit#:2 Entry:185354 Library:NIST11.lib
SI:90 Formula:C29H60 CAS:0-00-0 MolWeight:408 RetIndex:2840
CompName:2-methyloctacosane

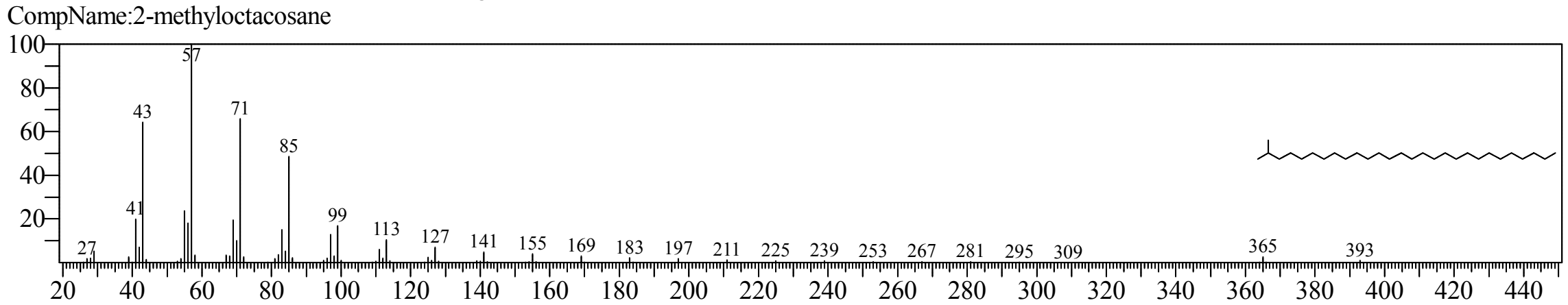

Hit#:3 Entry:27513 Library:NIST05s.LIB
SI:90 Formula:C44H90 CAS:7098-22-8 MolWeight:618 RetIndex:4395
CompName:Tetratetracontane $$ n-Tetratetracontane $$

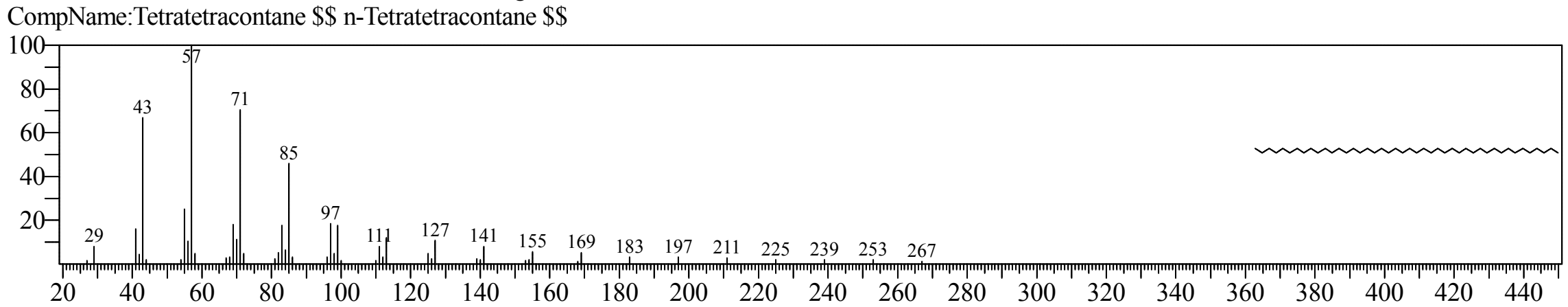

Hit#:4 Entry:27277 Library:NIST05s.LIB
SI:90 Formula:C35H72 CAS:630-07-9 MolWeight:492 RetIndex:3500
CompName:Pentatriacontane $$ n-Pentatriacontane $$

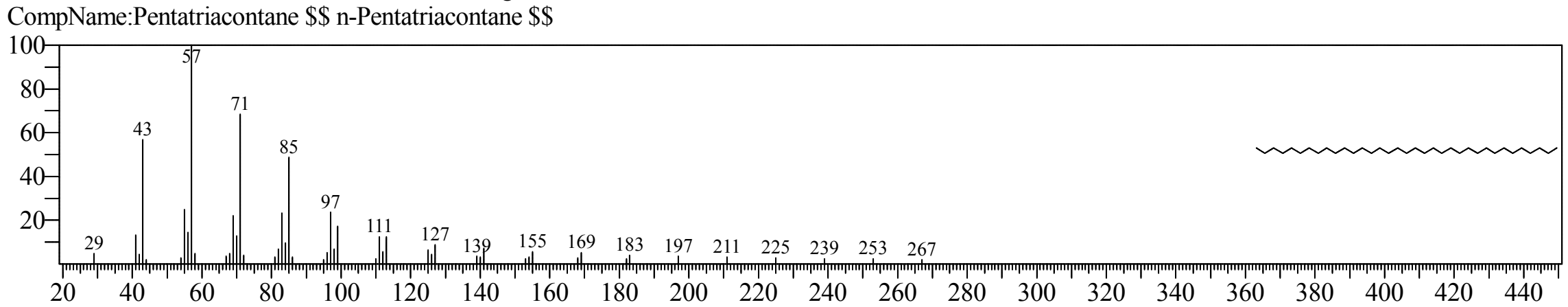

Hit#:5 Entry:27217 Library:NIST05s.LIB
SI:90 Formula:C34H70 CAS:14167-59-0 MolWeight:478 RetIndex:3401
CompName:Tetratriacontane $$ n-Tetratriacontane $$

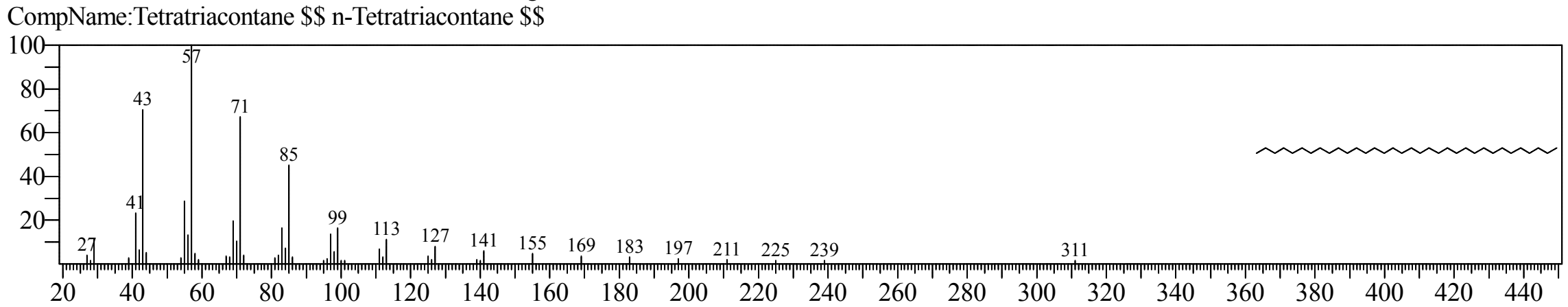

<< Target >>
Line#:12 R.Time:14.467(Scan#:1137) MassPeaks:58
RawMode:Averaged 14.458-14.475(1136-1138) BasePeak:57.10(15653)
BG Mode:Calc. from Peak Group 1 - Event 1 Scan

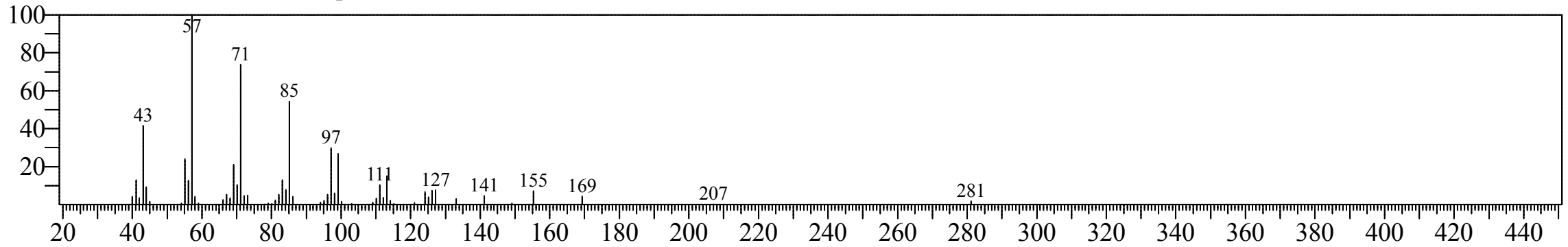

Hit#:6 Entry:161062 Library:NIST05.LIB
SI:89 Formula:C43H88 CAS:7098-21-7 MolWeight:604 RetIndex:4295
CompName:Tritetracontane

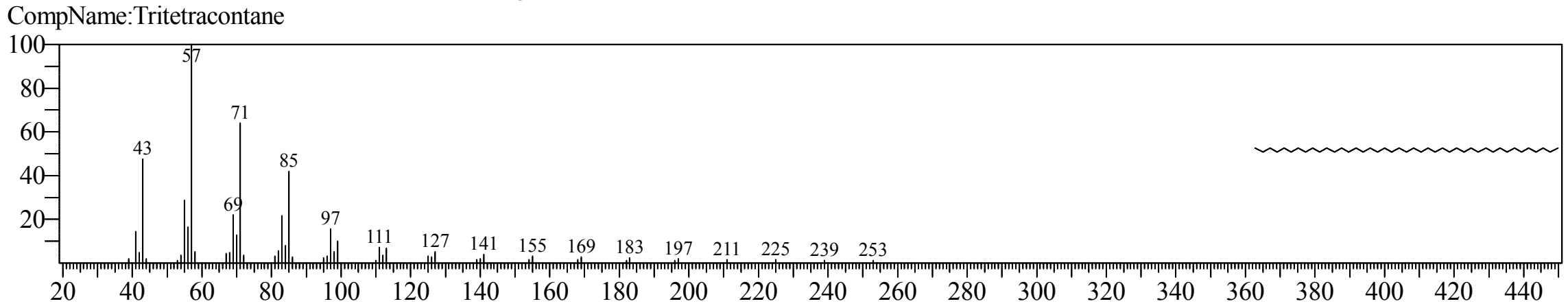

Hit#:7 Entry:23590 Library:NIST05s.LIB
SI:89 Formula:C21H44 CAS:629-94-7 MolWeight:296 RetIndex:2109
CompName:Heneicosane $$ n-Heneicosane $$ Henicosane # $$

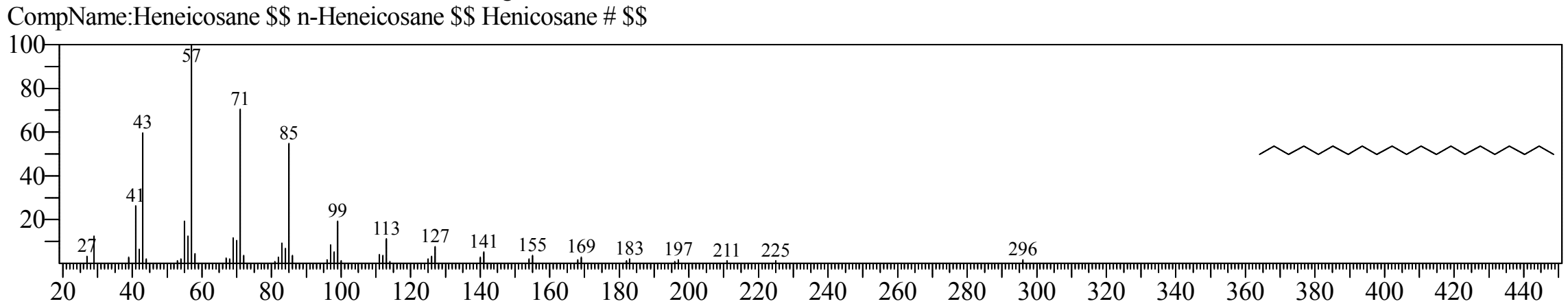

Hit#:8 Entry:26650 Library:NIST05s.LIB
SI:89 Formula:C29H60 CAS:630-03-5 MolWeight:408 RetIndex:2904
CompName:Nonacosane $$ n-Nonacosane $$

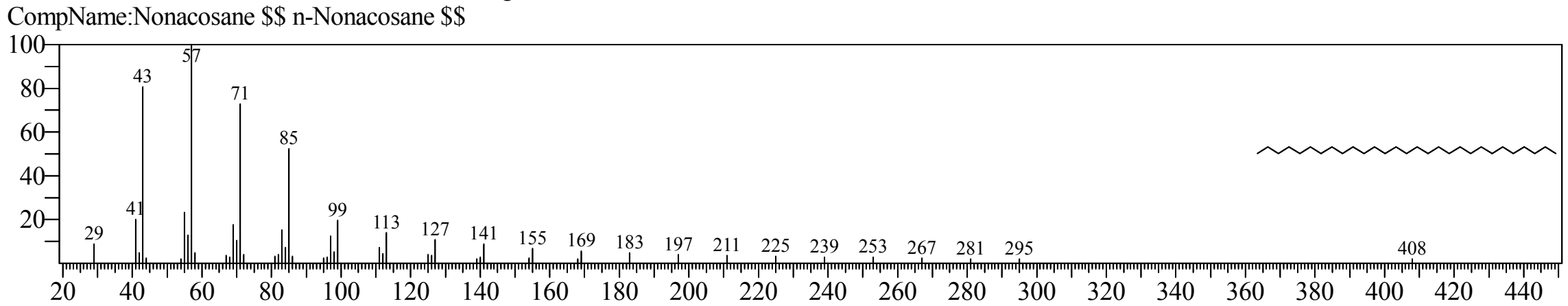

Hit#:9 Entry:22890 Library:NIST05s.LIB
SI:89 Formula:C20H42 CAS:112-95-8 MolWeight:282 RetIndex:2009
CompName:Eicosane $$ n-Eicosane $$ Icosane # $$

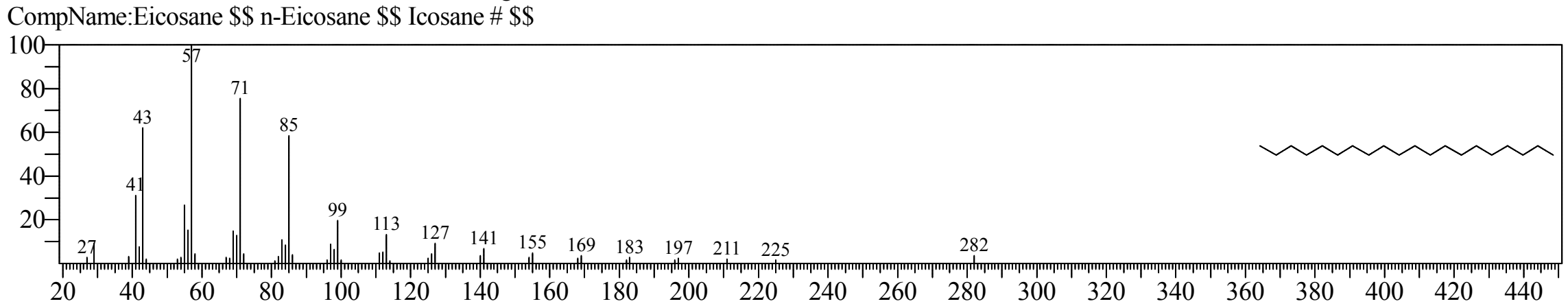

Hit#:10 Entry:178179 Library:WILEY7.LIB
SI:89 Formula:C19 H40 CAS:629-92-5 MolWeight:268 RetIndex:0
CompName:Nonadecane (CAS) n-Nonadecane $$

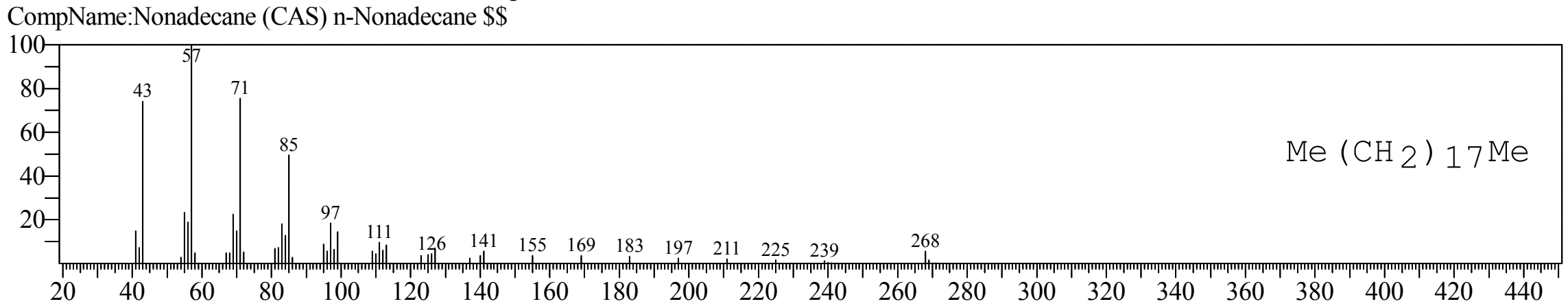

<< Target >>
Line#:12 R.Time:14.467(Scan#:1137) MassPeaks:58
RawMode:Averaged 14.458-14.475(1136-1138) BasePeak:57.10(15653)
BG Mode:Calc. from Peak Group 1 - Event 1 Scan

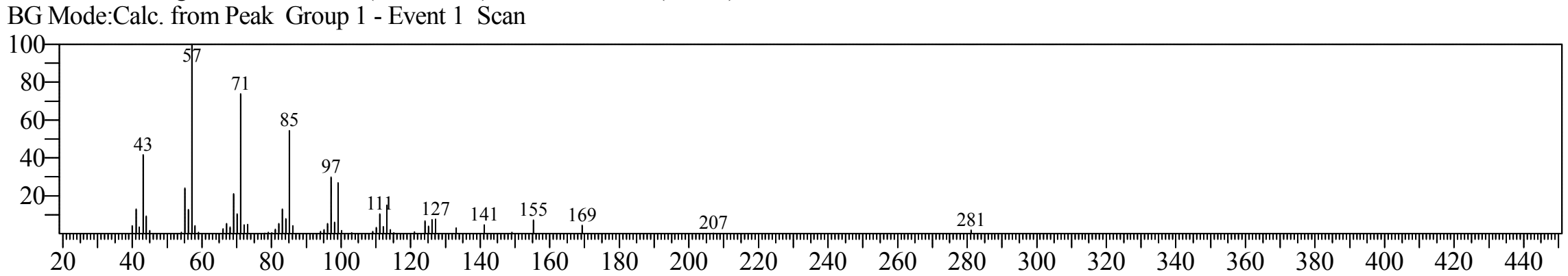

Hit#:11 Entry:27442 Library:NIST05s.LIB
SI:89 Formula:C40H82 CAS:4181-95-7 MolWeight:562 RetIndex:3997
CompName:Tetracontane $$ n-Tetracontane $$

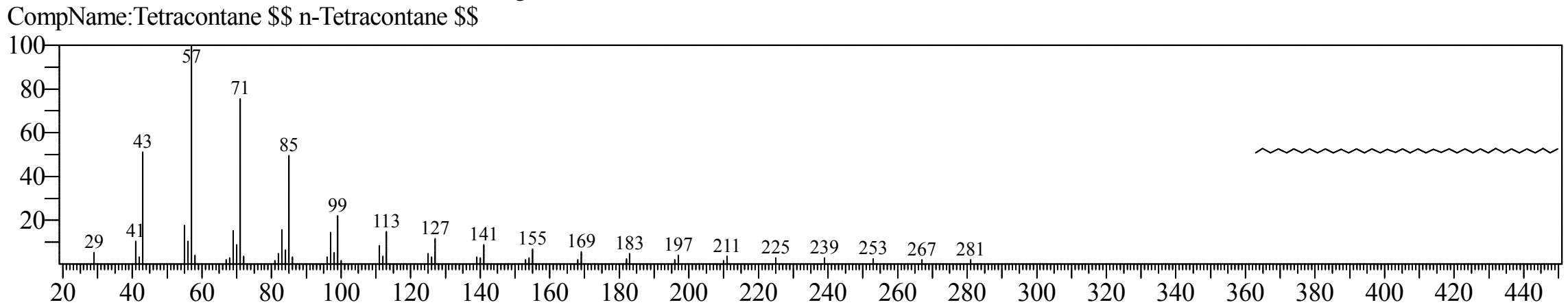

Hit#:12 Entry:27048 Library:NIST05s.LIB
SI:89 Formula:C32H66 CAS:544-85-4 MolWeight:450 RetIndex:3202
CompName:Dotriacontane $$ n-Dotriacontane $$ Bicetyl $$

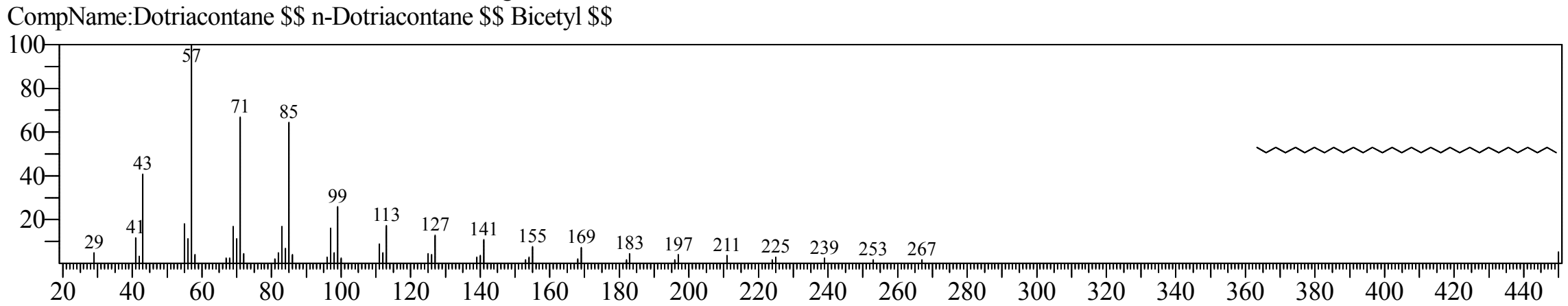

Hit#:13 Entry:28938 Library:NIST11s.lib
SI:89 Formula:C26H54 CAS:630-01-3 MolWeight:366 RetIndex:2606
CompName:Hexacosane $$ n-Hexacosane $$

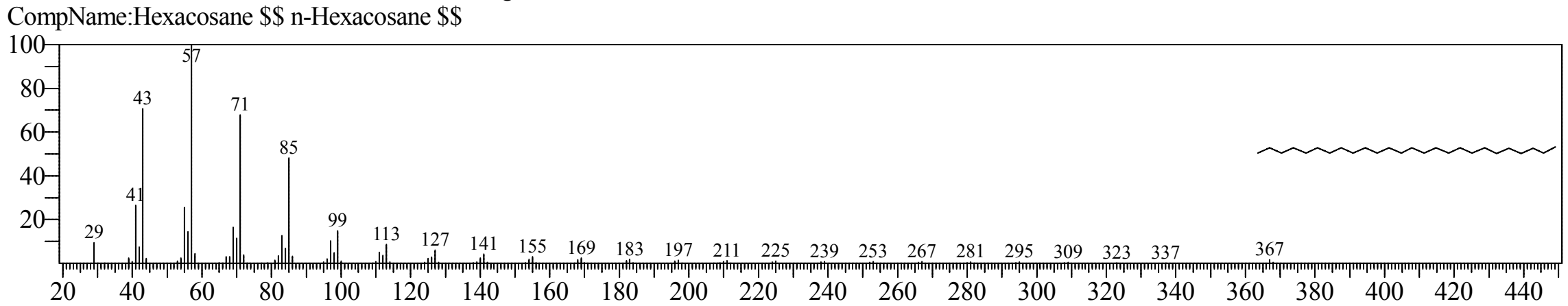

Hit#:14 Entry:90687 Library:NIST05.LIB
SI:88 Formula:C20H42 CAS:56862-62-5 MolWeight:282 RetIndex:1945
CompName:10-Methylnonadecane

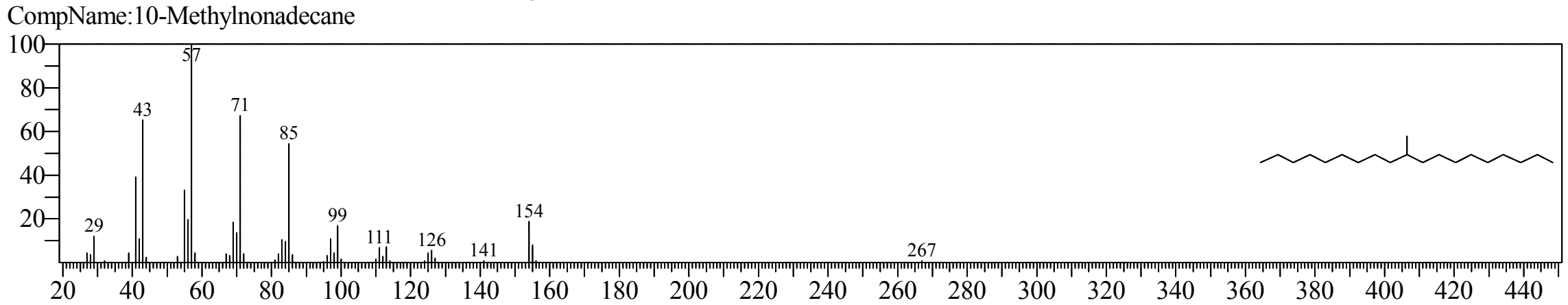

Hit#:15 Entry:98902 Library:NIST05.LIB
SI:88 Formula:C21H44 CAS:13475-75-7 MolWeight:296 RetIndex:2045
CompName:Pentadecane, 8-hexyl- $$ 8-n-Hexylpentadecane $$ 8-Hexylpentadecane # $$

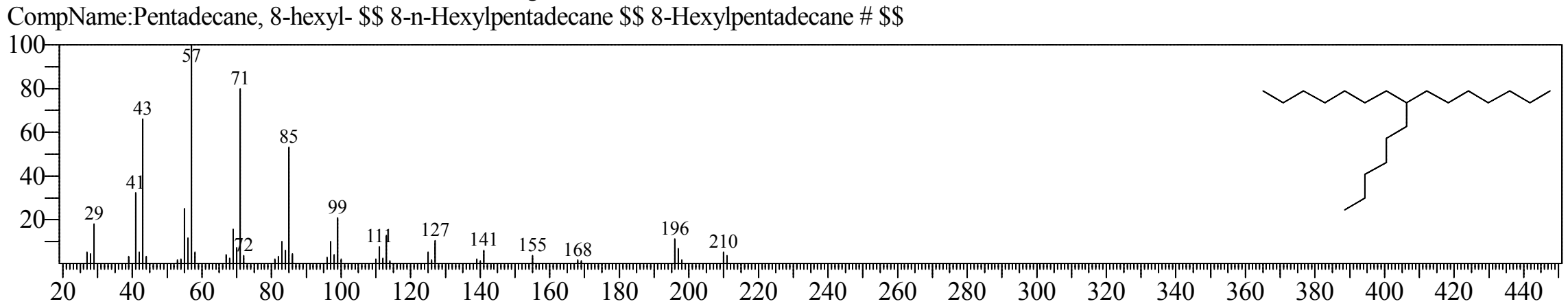

<< Target >>
Line#:12   R.Time:14.467(Scan#:1137) MassPeaks:58
RawMode:Averaged 14.458-14.475(1136-1138) BasePeak:57.10(15653)
BG Mode:Calc. from Peak Group 1 - Event 1 Scan

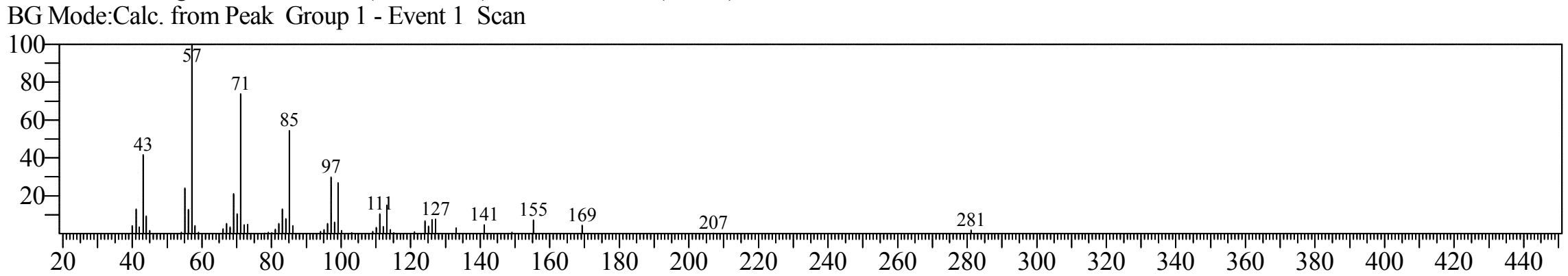

Hit#:16 Entry:255948 Library:WILEY7.LIB
SI:88 Formula:C25 H52 CAS:629-99-2 MolWeight:352 RetIndex:0
CompName:Pentacosane (CAS) n-Pentacosane $$

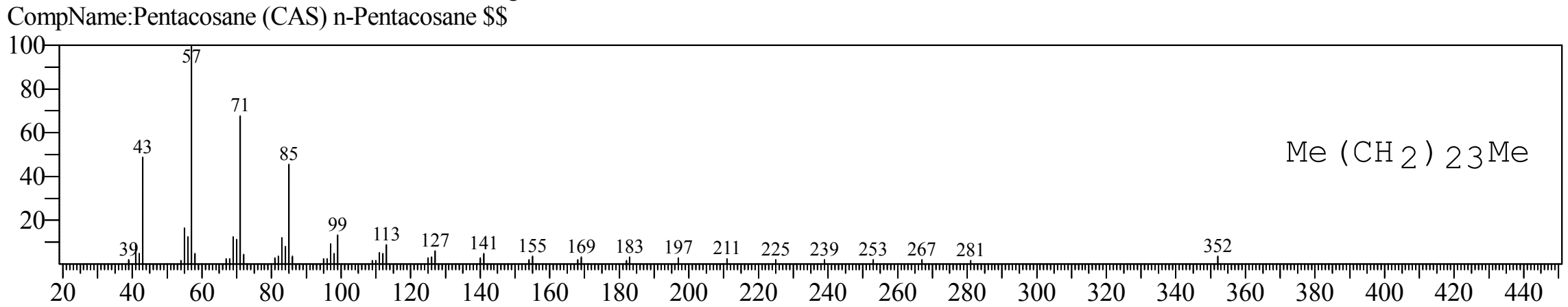

Hit#:17 Entry:221426 Library:WILEY7.LIB
SI:88 Formula:C22 H46 CAS:629-97-0 MolWeight:310 RetIndex:0
CompName:Docosane (CAS) n-Docosane $$ C22H46 STANDARD $$ Normal-docosane $$

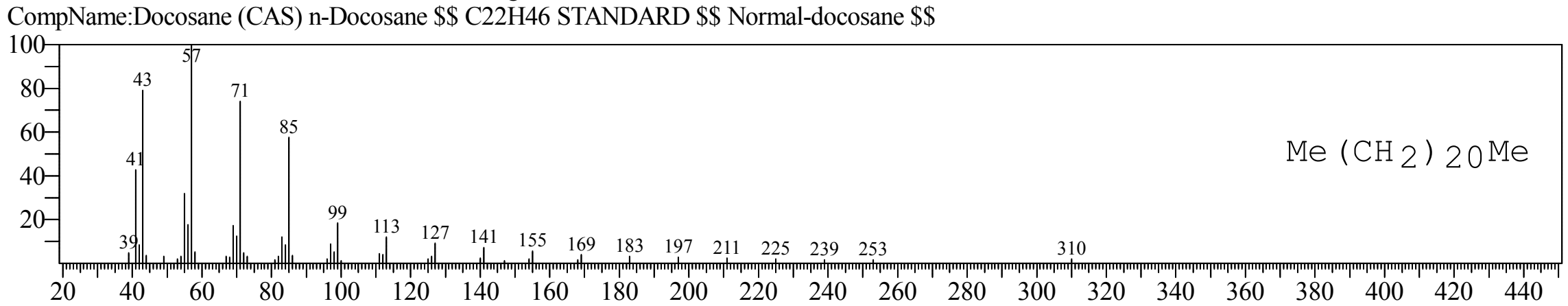

Hit#:18 Entry:98901 Library:NIST05.LIB
SI:88 Formula:C21H44 CAS:54833-48-6 MolWeight:296 RetIndex:1852
CompName:Heptadecane, 2,6,10,15-tetramethyl- $$ 2,6,10,15-Tetramethylheptadecane # $$

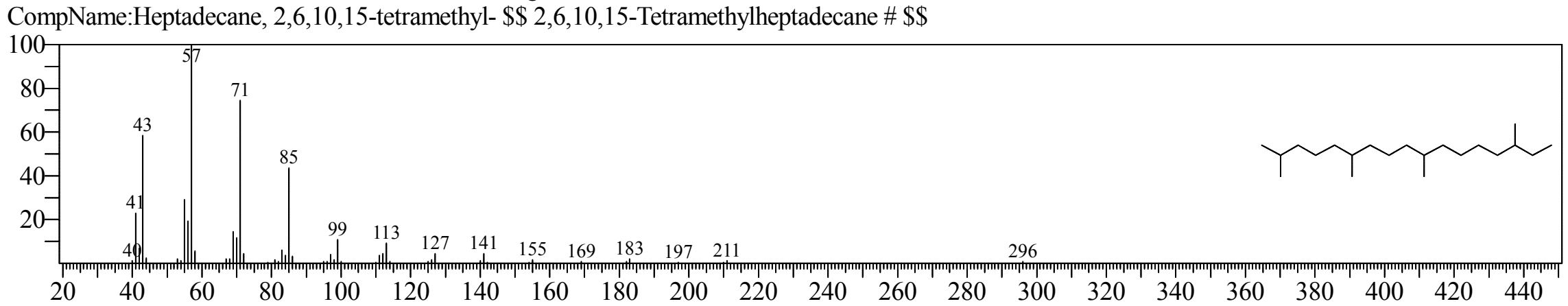

Hit#:19 Entry:26455 Library:NIST05s.LIB
SI:88 Formula:C28H58 CAS:630-02-4 MolWeight:394 RetIndex:2804
CompName:Octacosane $$ n-Octacosane $$

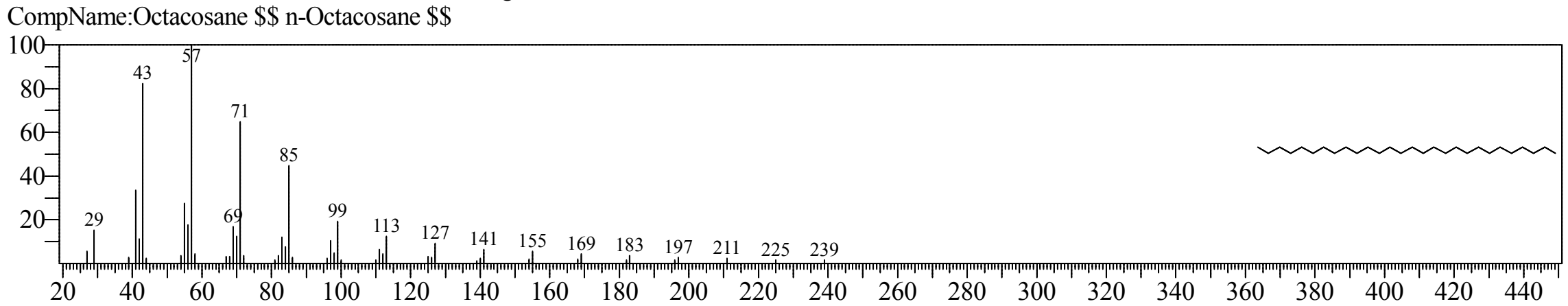

Hit#:20 Entry:157632 Library:NIST05.LIB
SI:88 Formula:C36H74 CAS:630-06-8 MolWeight:506 RetIndex:3600
CompName:Hexatriacontane $$ n-Hexatriacontane $$

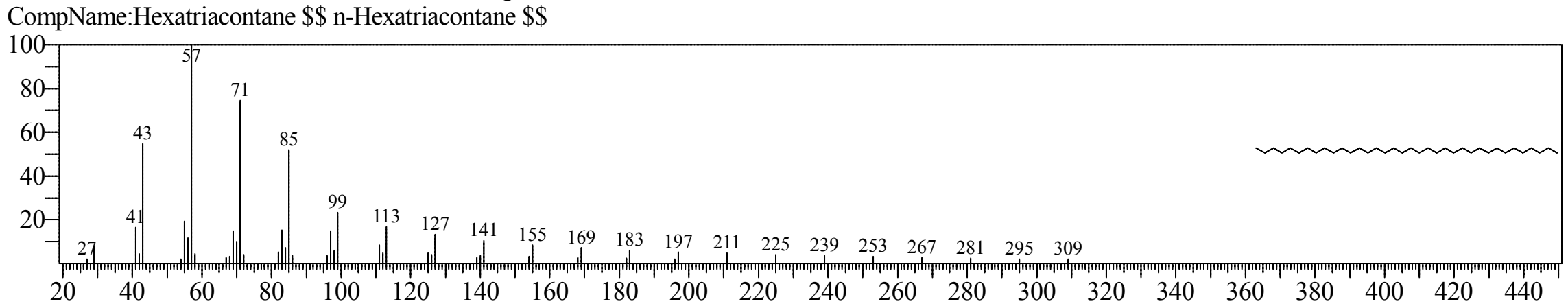

<< Target >>
Line#:13   R.Time:14.608(Scan#:1154) MassPeaks:50
RawMode:Averaged 14.600-14.617(1153-1155) BasePeak:57.10(2417)
BG Mode:Calc. from Peak Group 1 - Event 1 Scan

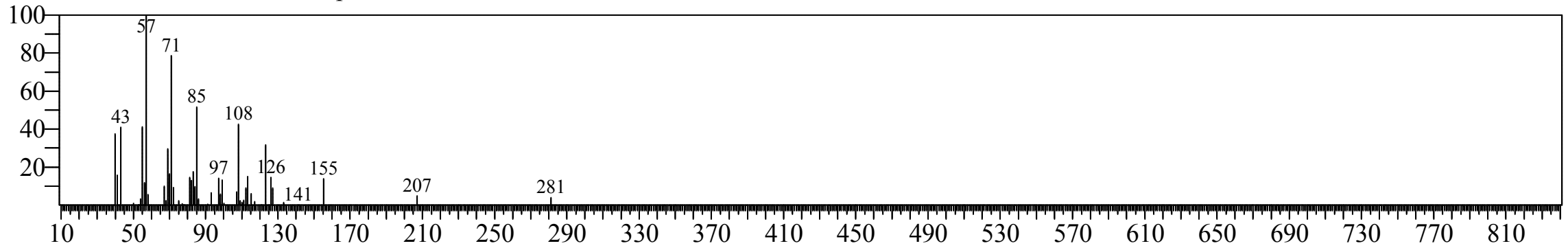

Hit#:1 Entry:221425 Library:WILEY7.LIB
SI:82 Formula:C22 H46 CAS:629-97-0 MolWeight:310 RetIndex:0
CompName:Docosane (CAS) n-Docosane $$ C22H46 STANDARD $$ Normal-docosane $$

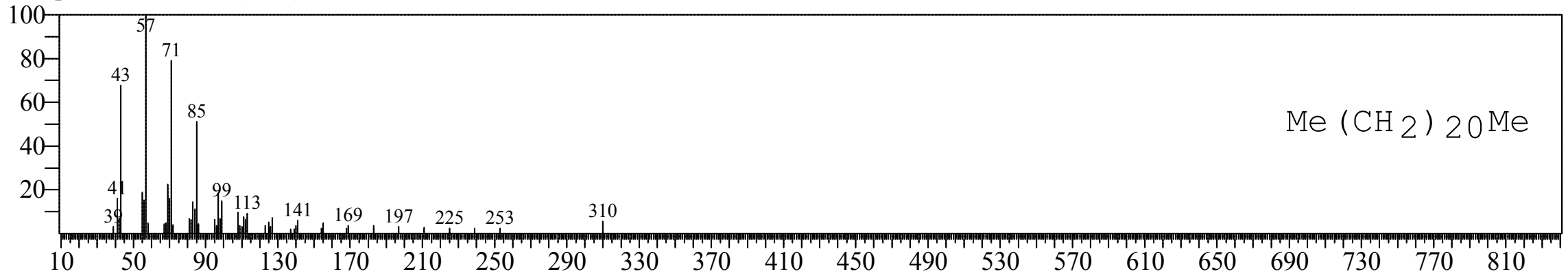

Hit#:2 Entry:157091 Library:NIST11.lib
SI:82 Formula:C25H52 CAS:0-00-0 MolWeight:352 RetIndex:2442
CompName:2-methyltetracosane

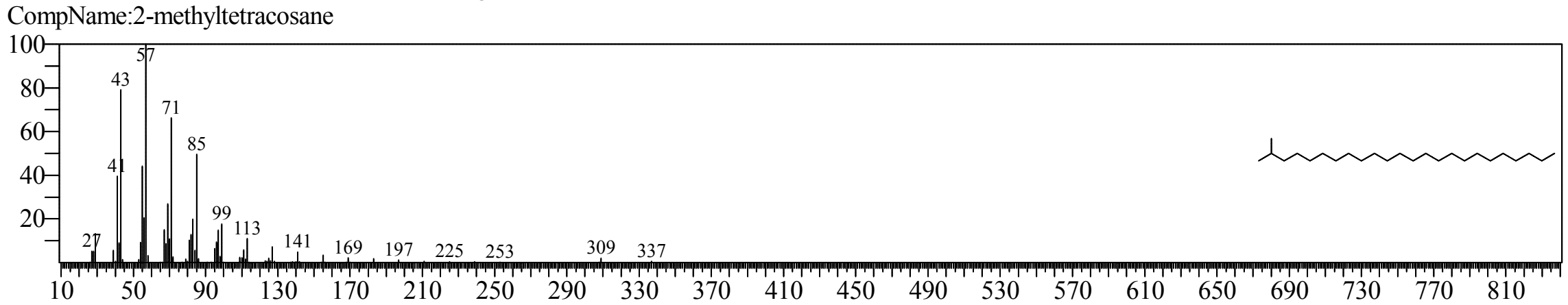

Hit#:3 Entry:178179 Library:WILEY7.LIB
SI:81 Formula:C19 H40 CAS:629-92-5 MolWeight:268 RetIndex:0
CompName:Nonadecane (CAS) n-Nonadecane $$

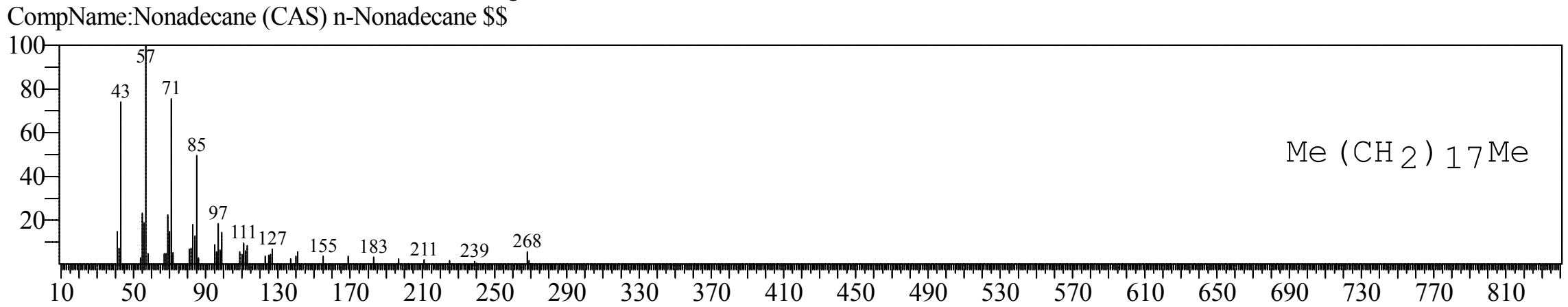

Hit#:4 Entry:145947 Library:WILEY7.LIB
SI:80 Formula:C17 H36 CAS:14905-56-7 MolWeight:240 RetIndex:0
CompName:Tetradecane, 2,6,10-trimethyl- (CAS) 2,6,10-Trimethyltetradecane $$

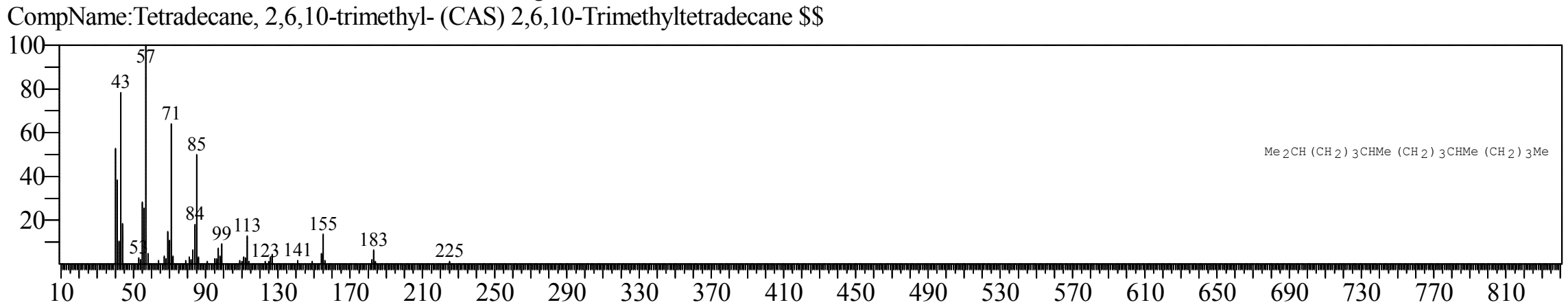

Hit#:5 Entry:173393 Library:NIST11.lib
SI:80 Formula:C27H56 CAS:0-00-0 MolWeight:380 RetIndex:2641
CompName:2-methylhexacosane

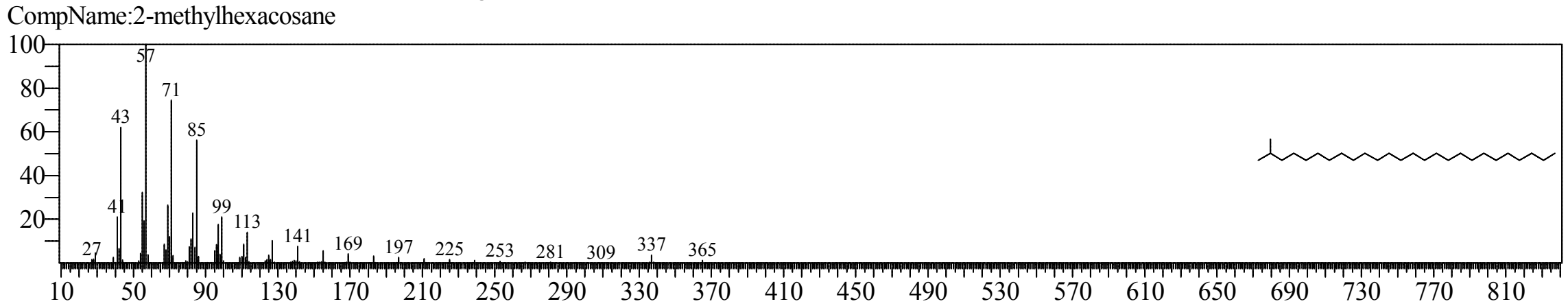

<< Target >>
Line#:13 R.Time:14.608(Scan#:1154) MassPeaks:50
RawMode:Averaged 14.600-14.617(1153-1155) BasePeak:57.10(2417)
BG Mode:Calc. from Peak Group 1 - Event 1 Scan

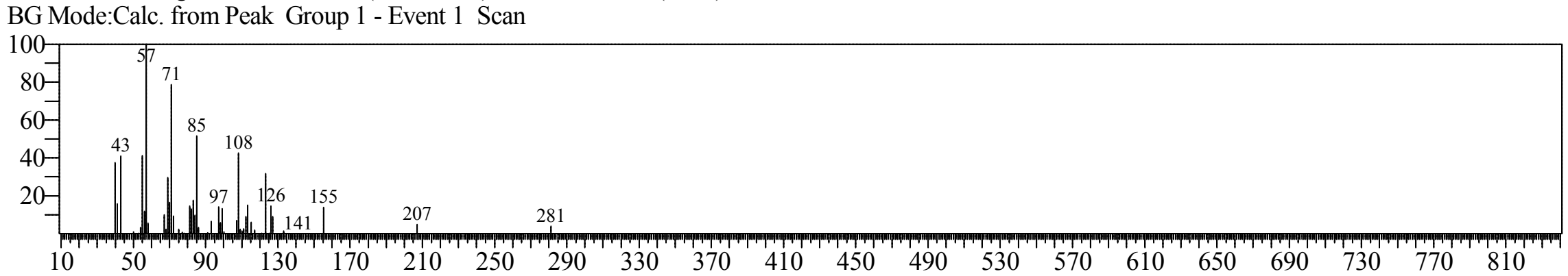

Hit#:6 Entry:212800 Library:NIST11.lib
SI:80 Formula:C54H108Br2 CAS:0-00-0 MolWeight:914 RetIndex:5981
CompName:Tetrapentacontane, 1,54-dibromo- $$ 1,54-Dibromotetrapentacontane # $$

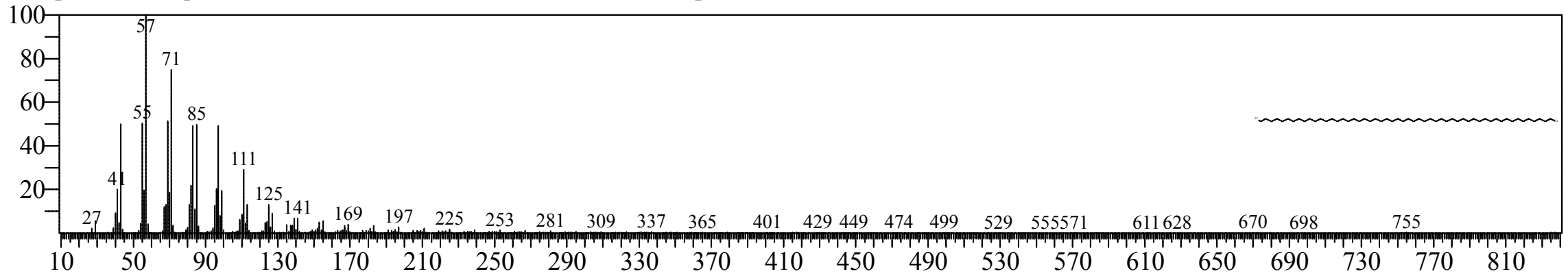

Hit#:7 Entry:163080 Library:NIST05.LIB
SI:80 Formula:C54H108Br2 CAS:0-00-0 MolWeight:914 RetIndex:5981
CompName:Tetrapentacontane, 1,54-dibromo-

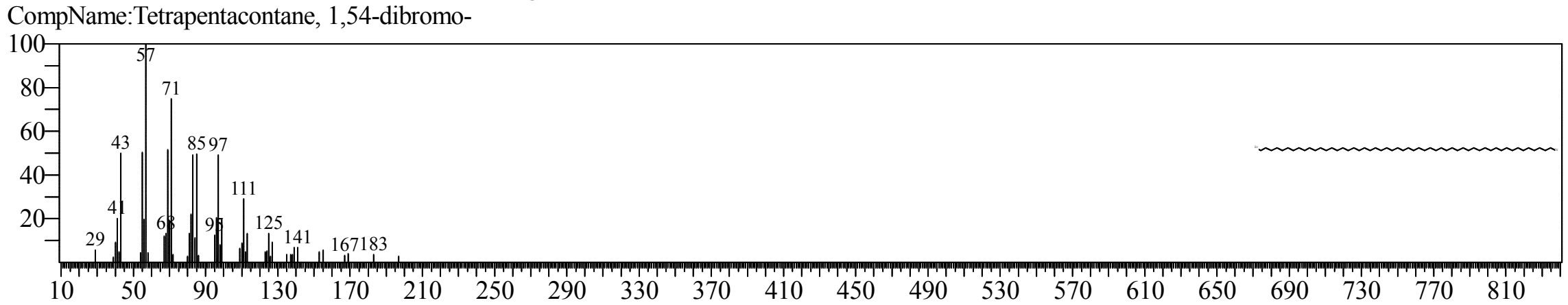

Hit#:8 Entry:337461 Library:WILEY7.LIB
SI:80 Formula:C54 H108 BR2 CAS:0-00-0 MolWeight:915 RetIndex:0
CompName:TETRAPENTACONTAN, 1,54-DIBROMO- $$

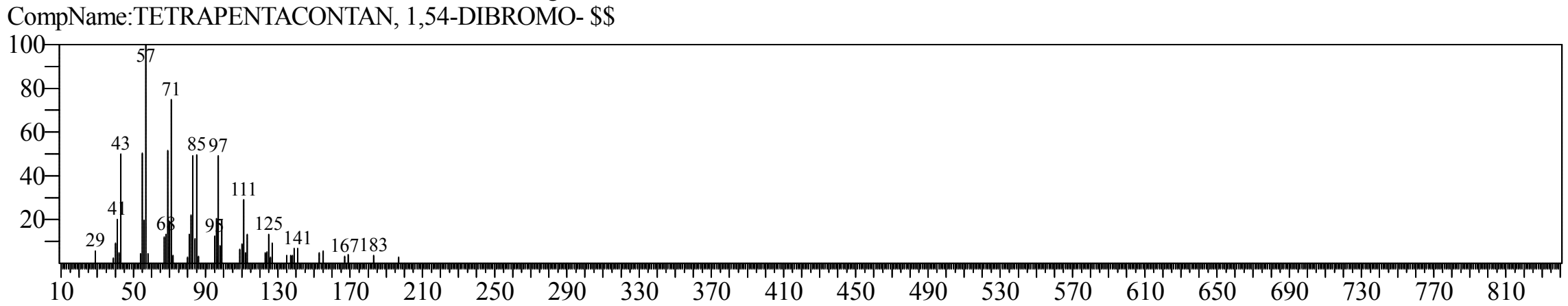

Hit#:9 Entry:99570 Library:NIST11.lib
SI:80 Formula:C14H29Br CAS:74036-95-6 MolWeight:276 RetIndex:1645
CompName:2-Bromotetradecane

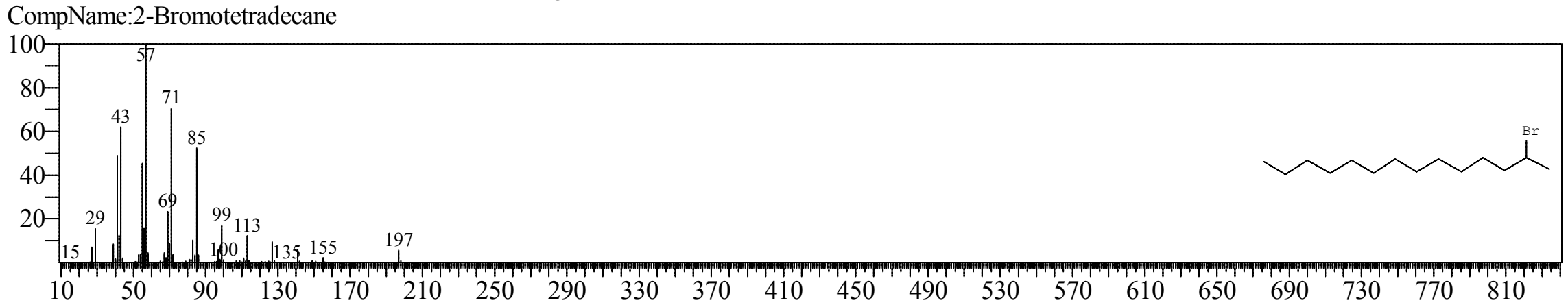

Hit#:10 Entry:98901 Library:NIST05.LIB
SI:79 Formula:C21H44 CAS:54833-48-6 MolWeight:296 RetIndex:1852
CompName:Heptadecane, 2,6,10,15-tetramethyl- $$ 2,6,10,15-Tetramethylheptadecane # $$

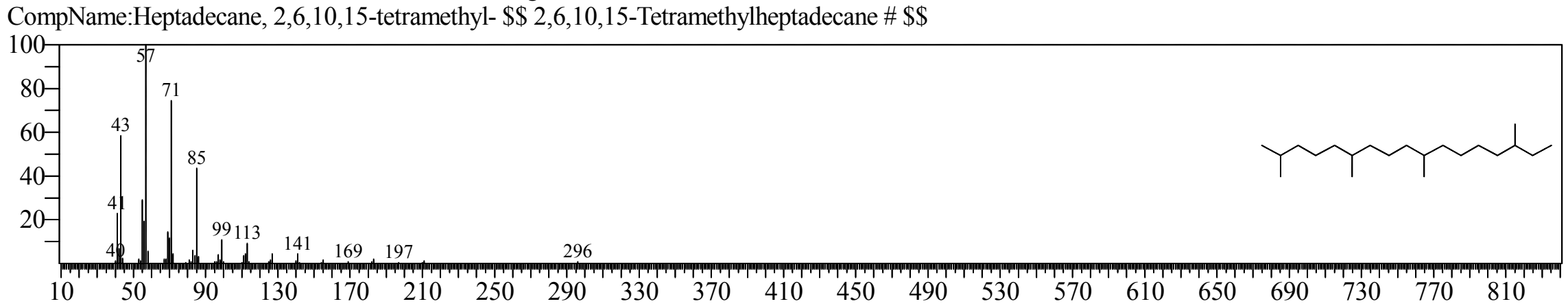

<< Target >>
Line#:13 R.Time:14.608(Scan#:1154) MassPeaks:50
RawMode:Averaged 14.600-14.617(1153-1155) BasePeak:57.10(2417)
BG Mode:Calc. from Peak Group 1 - Event 1 Scan

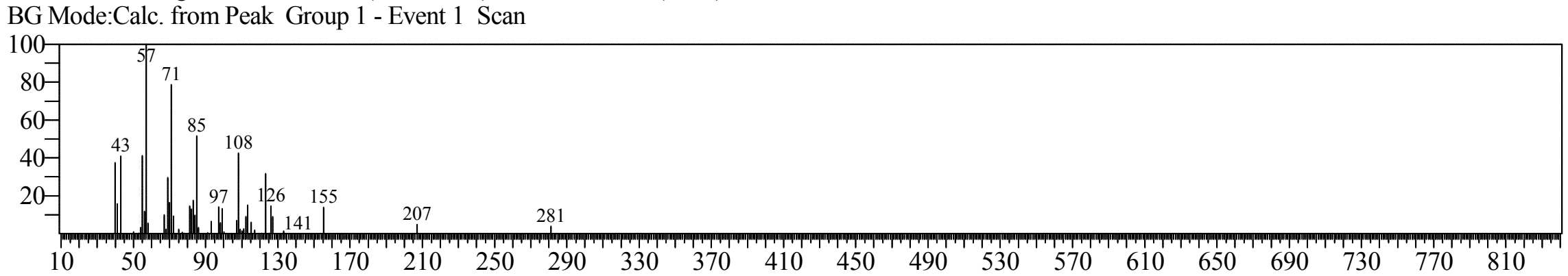

Hit#:11 Entry:304720 Library:WILEY7.LIB
SI:79 Formula:C32 H66 CAS:544-85-4 MolWeight:451 RetIndex:0
CompName:Dotriacontane (CAS) n-Dotriacontane $$ Bicetyl $$ Tris(trimethylsilyl)ether, methyl ester of ethyl anthranilate azo pigment(.alpha.z) of bilivubi

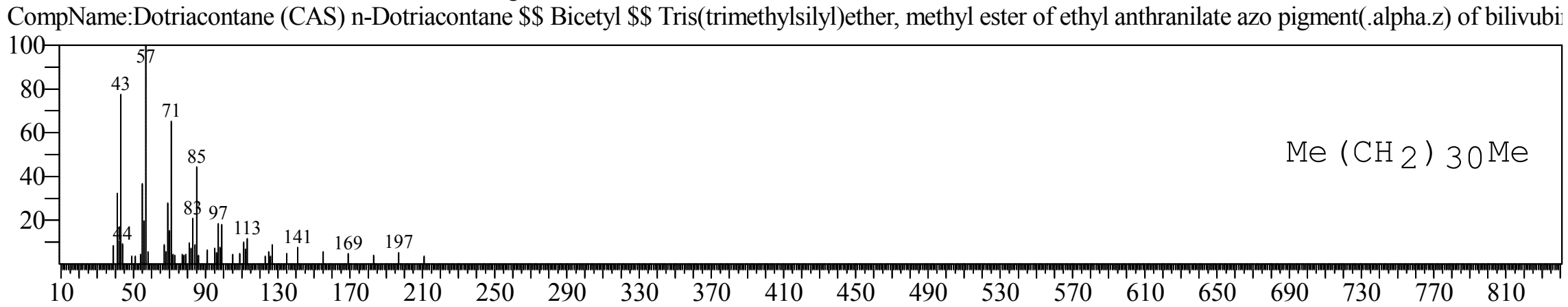

Hit#:12 Entry:234111 Library:WILEY7.LIB
SI:79 Formula:C23 H48 CAS:638-67-5 MolWeight:324 RetIndex:0
CompName:Tricosane (CAS) n-Tricosane $$

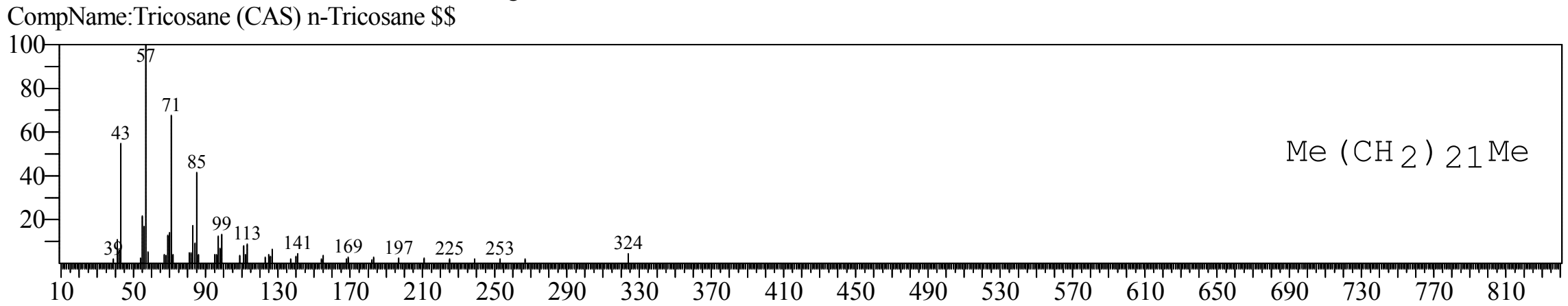

Hit#:13 Entry:157214 Library:NIST05.LIB
SI:79 Formula:C30H61Br CAS:4209-22-7 MolWeight:500 RetIndex:3299
CompName:Triacontane, 1-bromo- $$ 1-Bromotriacontane # $$

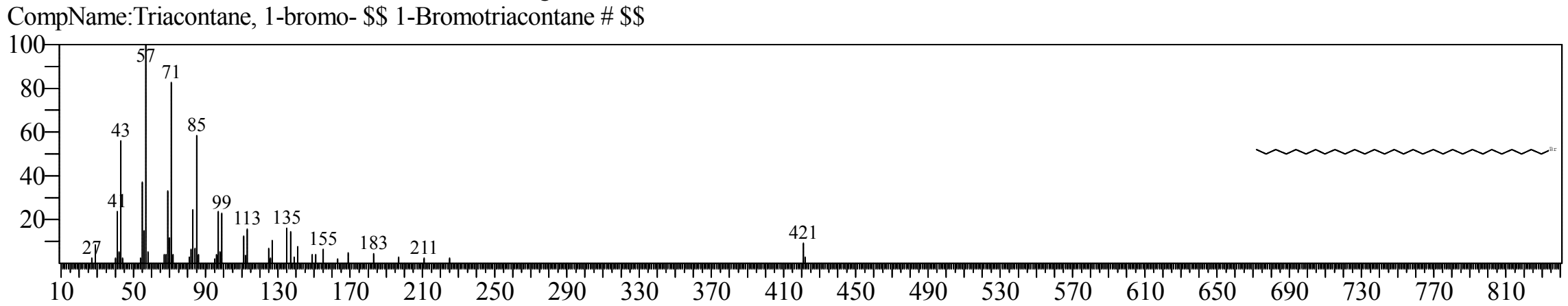

Hit#:14 Entry:162282 Library:NIST05.LIB
SI:79 Formula:C38H76Br2 CAS:102436-01-1 MolWeight:690 RetIndex:4391
CompName:Octatriacontane, 1,38-dibromo- $$ 1,38-Dibromooctatriacontane # $$

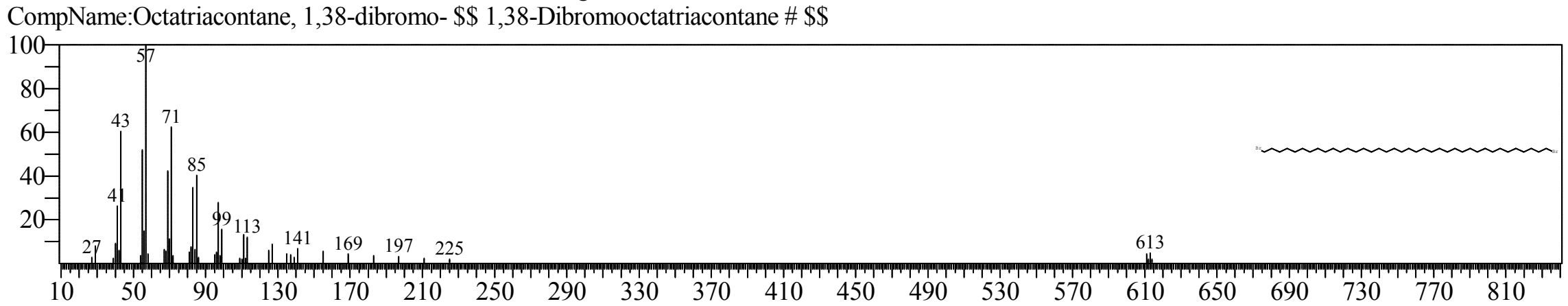

Hit#:15 Entry:161062 Library:NIST05.LIB
SI:79 Formula:C43H88 CAS:7098-21-7 MolWeight:604 RetIndex:4295
CompName:Tritetracontane

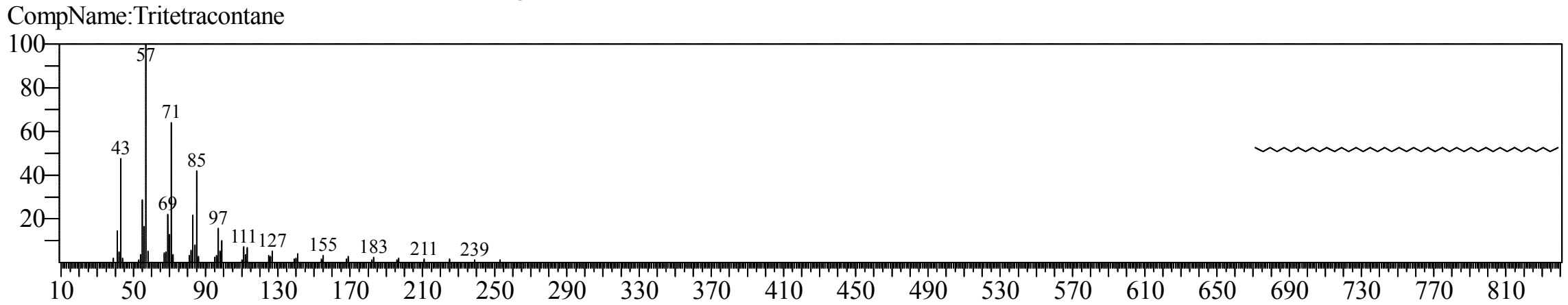

<< Target >>
Line#:13   R.Time:14.608(Scan#:1154) MassPeaks:50
RawMode:Averaged 14.600-14.617(1153-1155) BasePeak:57.10(2417)
BG Mode:Calc. from Peak Group 1 - Event 1 Scan

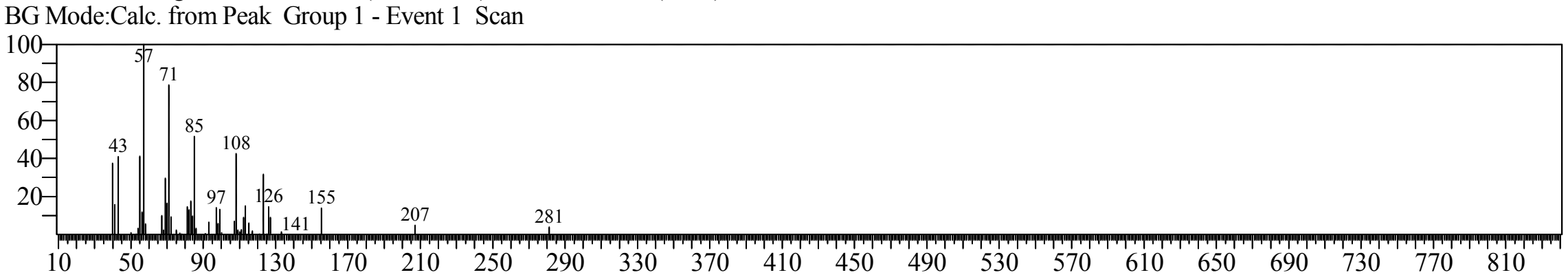

Hit#:16 Entry:147654 Library:NIST05s.LIB
SI:79 Formula:C27H55Cl CAS:62016-79-9 MolWeight:414 RetIndex:2930
CompName:Heptacosane, 1-chloro- $$ 1-Chloroheptacosane # $$

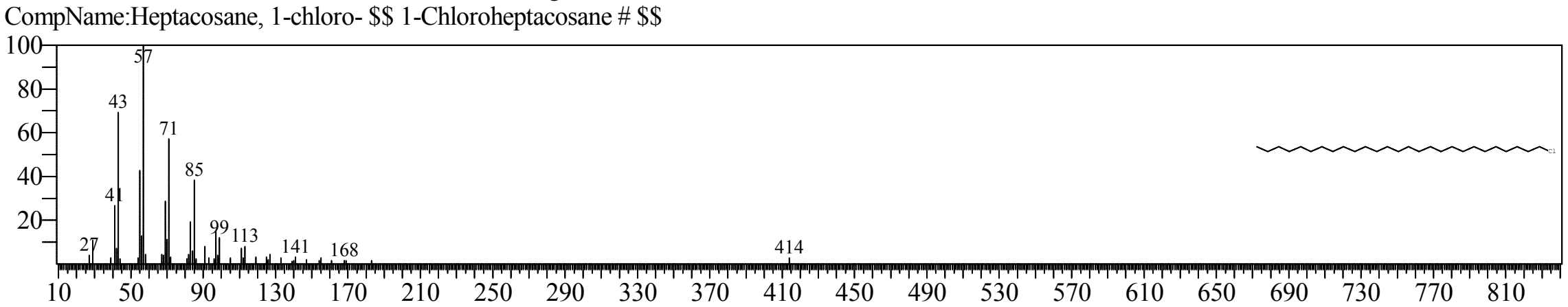

Hit#:17 Entry:27587 Library:NIST05s.LIB
SI:79 Formula:C50H102 CAS:55256-09-2 MolWeight:702 RetIndex:4863
CompName:Triacontane, 11,20-didecyl- $$ 11,20-Di-n-decyltriacontane $$ 11,20-Didecyltriacontane # $$

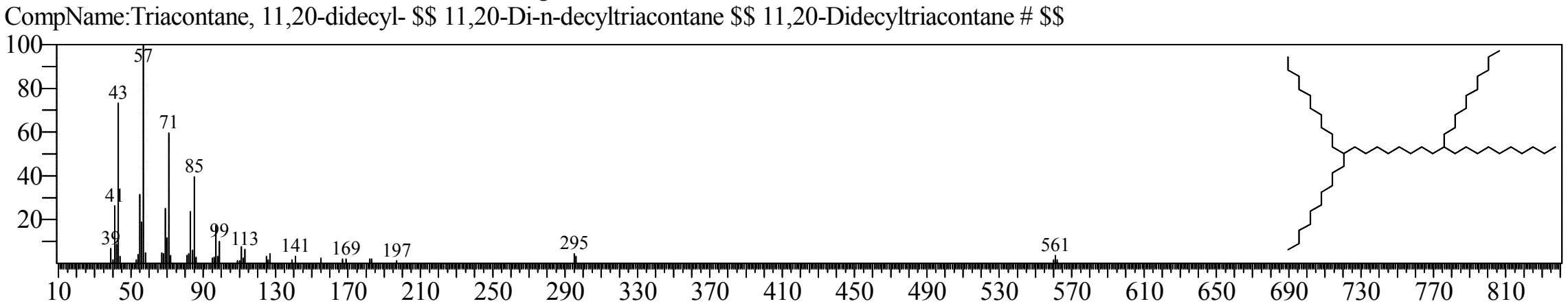

Hit#:18 Entry:27513 Library:NIST05s.LIB
SI:79 Formula:C44H90 CAS:7098-22-8 MolWeight:618 RetIndex:4395
CompName:Tetratetracontane $$ n-Tetratetracontane $$

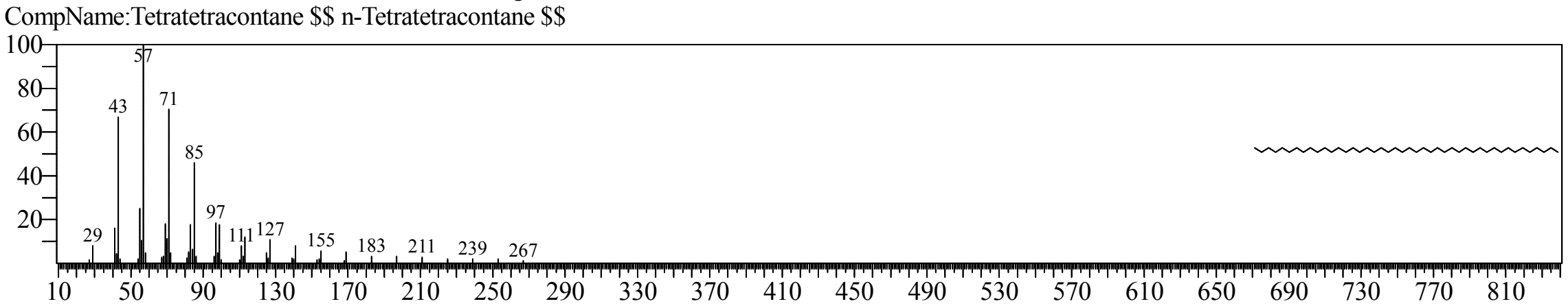

Hit#:19 Entry:145292 Library:NIST05s.LIB
SI:79 Formula:C24H50S2 CAS:27458-90-8 MolWeight:402 RetIndex:2739
CompName:Disulfide, di-tert-dodecyl $$ Di-tert-dodecyl disulfide $$

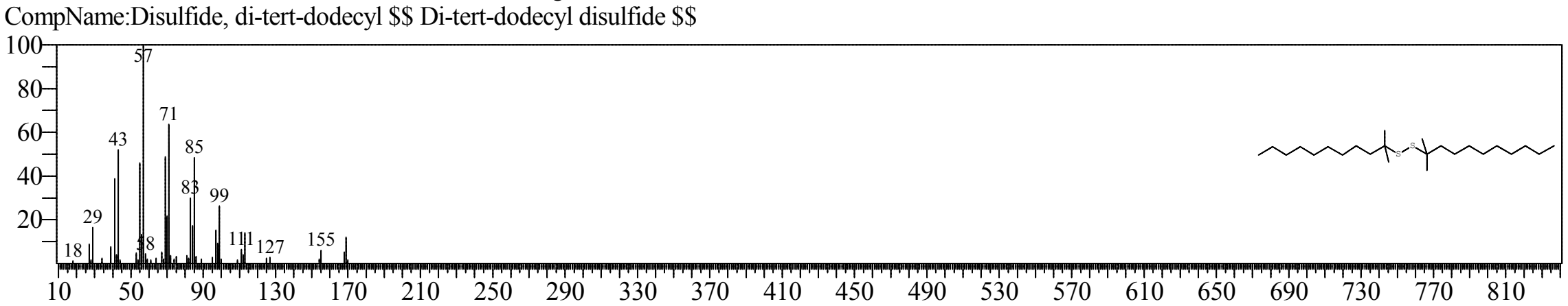

Hit#:20 Entry:27217 Library:NIST05s.LIB
SI:78 Formula:C34H70 CAS:14167-59-0 MolWeight:478 RetIndex:3401
CompName:Tetratriacontane $$ n-Tetratriacontane $$

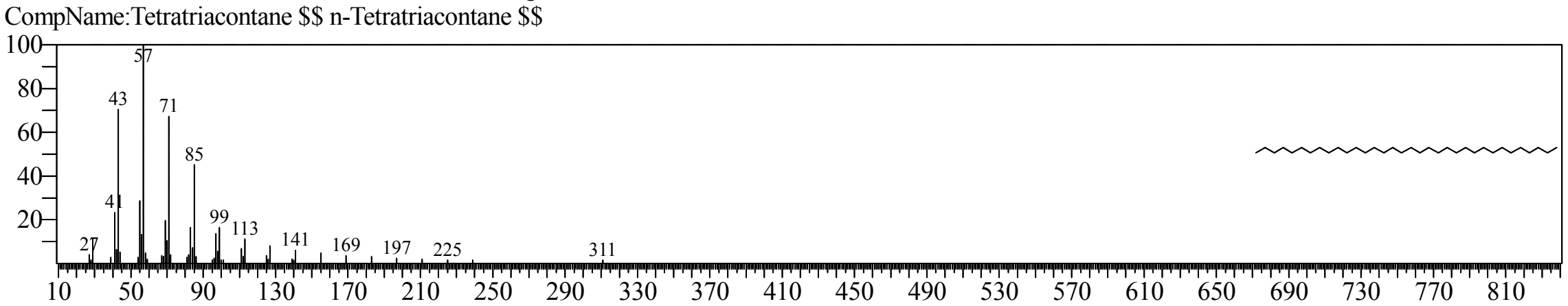

<< Target >>
Line#:14 R.Time:14.658(Scan#:1160) MassPeaks:48
RawMode:Averaged 14.650-14.667(1159-1161) BasePeak:85.15(1206)
BG Mode:Calc. from Peak Group 1 - Event 1 Scan

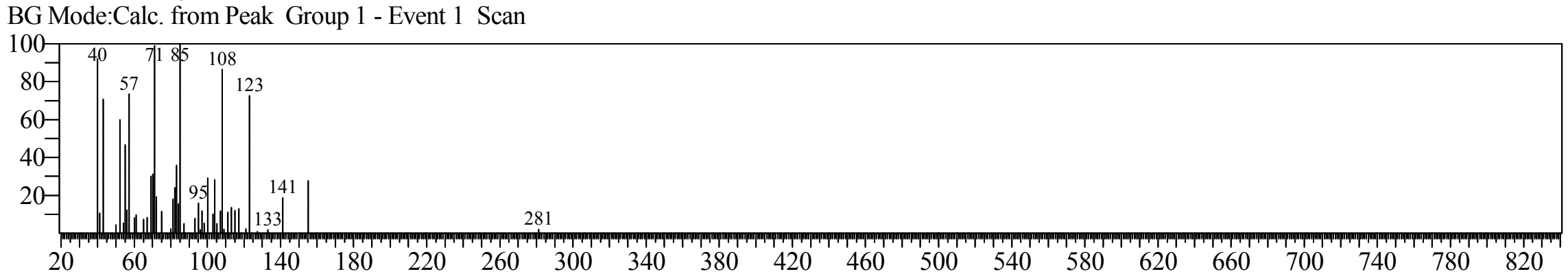

Hit#:1 Entry:337461 Library:WILEY7.LIB
SI:67 Formula:C54 H108 BR2 CAS:0-00-0 MolWeight:915 RetIndex:0
CompName:TETRAPENTACONTAN, 1,54-DIBROMO- $$

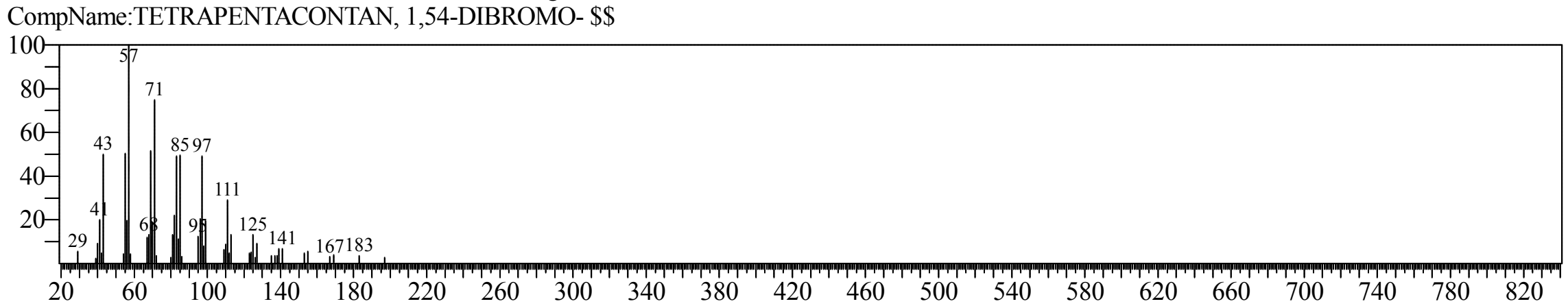

Hit#:2 Entry:163080 Library:NIST05.LIB
SI:67 Formula:C54H108Br2 CAS:0-00-0 MolWeight:914 RetIndex:5981
CompName:Tetrapentacontane, 1,54-dibromo-

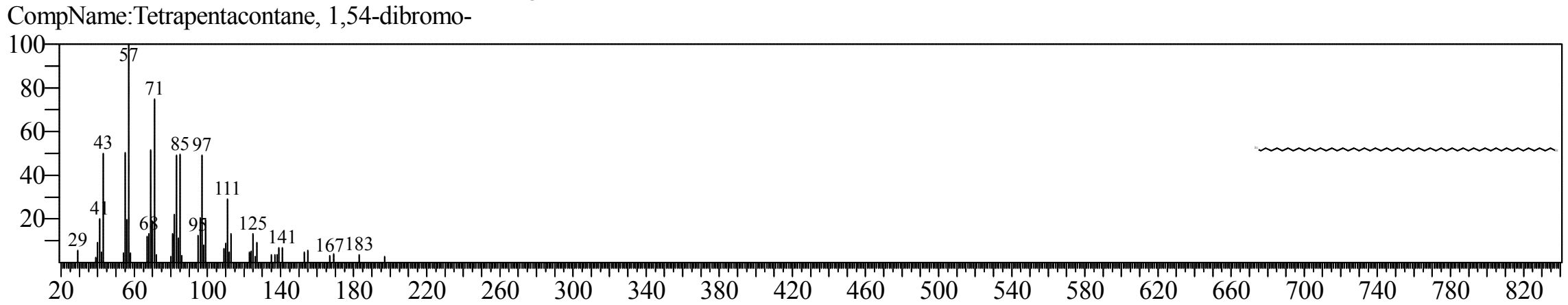

Hit#:3 Entry:212800 Library:NIST11.lib
SI:67 Formula:C54H108Br2 CAS:0-00-0 MolWeight:914 RetIndex:5981
CompName:Tetrapentacontane, 1,54-dibromo- $$ 1,54-Dibromotetrapentacontane # $$

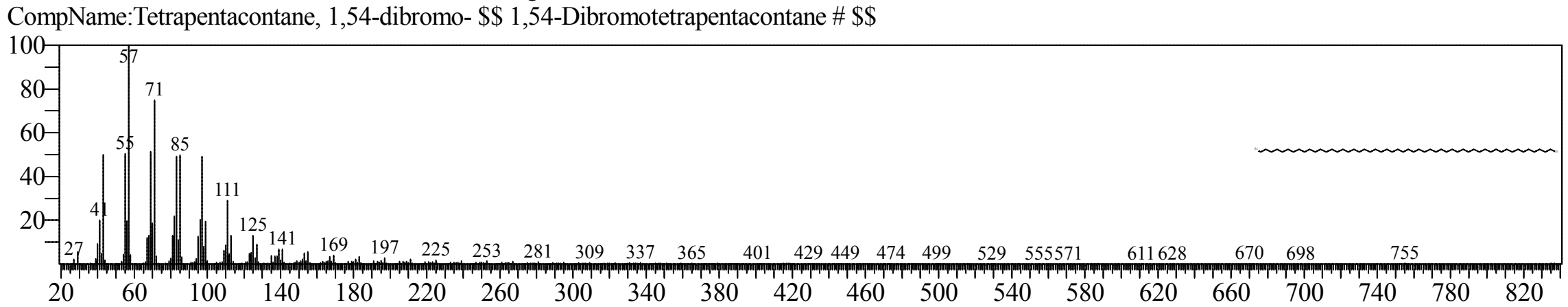

Hit#:4 Entry:157091 Library:NIST11.lib
SI:66 Formula:C25H52 CAS:0-00-0 MolWeight:352 RetIndex:2442
CompName:2-methyltetracosane

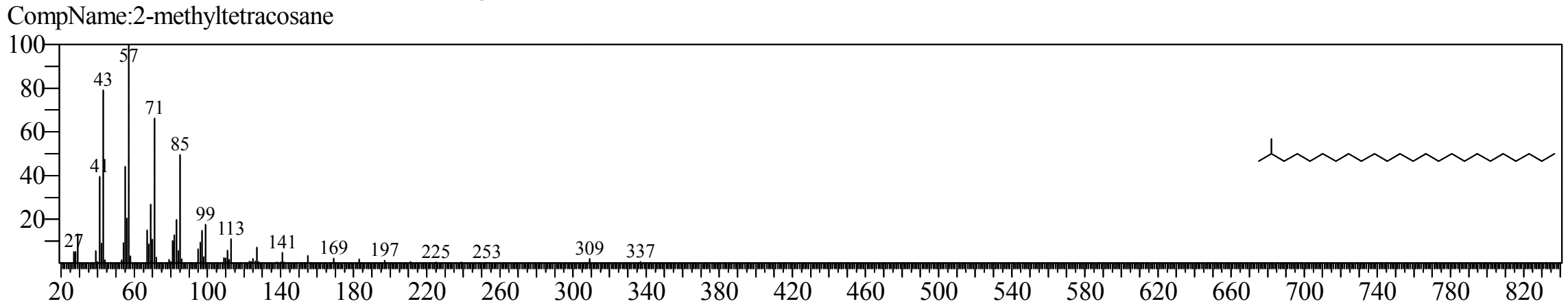

Hit#:5 Entry:17627 Library:NIST05s.LIB
SI:66 Formula:C13H24O2 CAS:57856-81-2 MolWeight:212 RetIndex:1471
CompName:Decanoic acid, 2-propenyl ester $$ Allyl caprate $$ Allyl n-decanoate $$ Allyl decanoate # $$

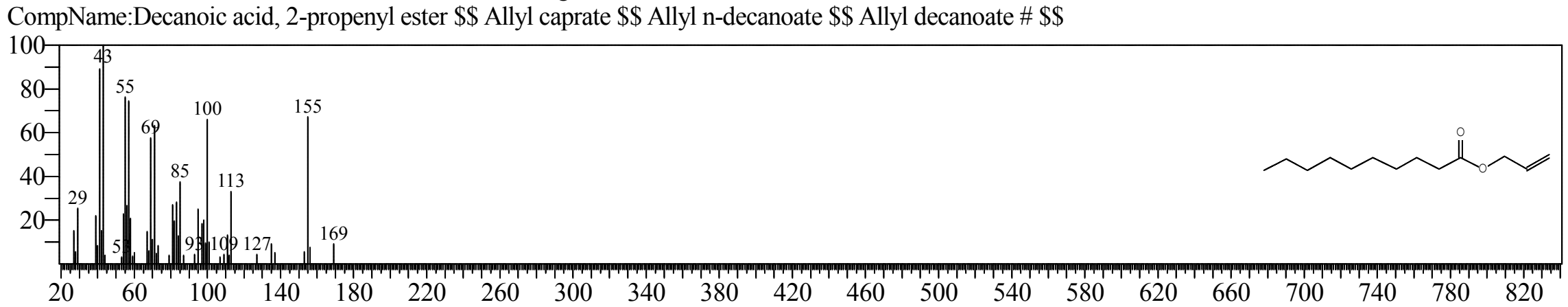

<< Target >>
Line#:14 R.Time:14.658(Scan#:1160) MassPeaks:48
RawMode:Averaged 14.650-14.667(1159-1161) BasePeak:85.15(1206)
BG Mode:Calc. from Peak Group 1 - Event 1 Scan

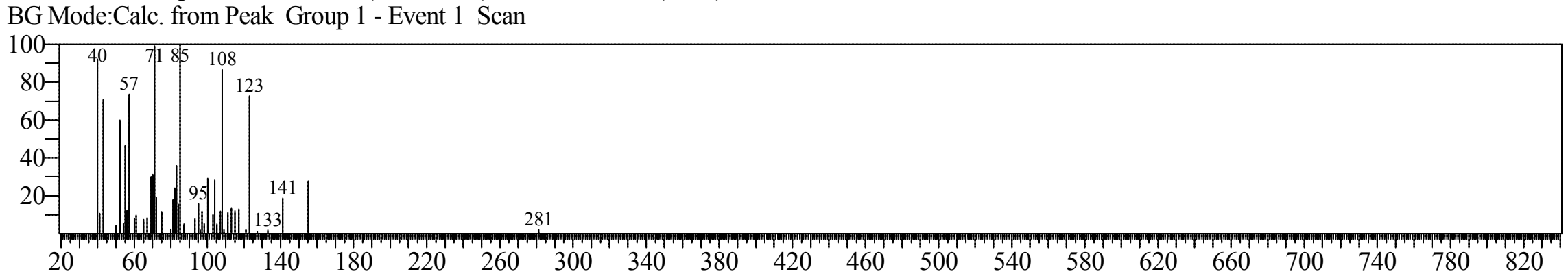

Hit#:6 Entry:167841 Library:NIST11.lib
SI:66 Formula:C22H42O4 CAS:0-00-0 MolWeight:370 RetIndex:2286
CompName:Oxalic acid, bis(6-ethyloct-3-yl) ester

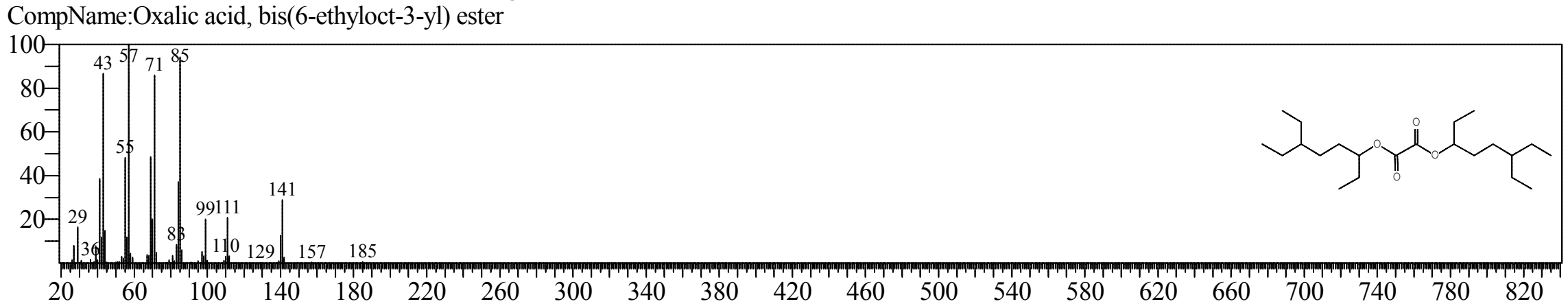

Hit#:7 Entry:135453 Library:NIST05.LIB
SI:66 Formula:C22H42O4 CAS:0-00-0 MolWeight:370 RetIndex:2286
CompName:Oxalic acid, bis(6-ethyloct-3-yl) ester

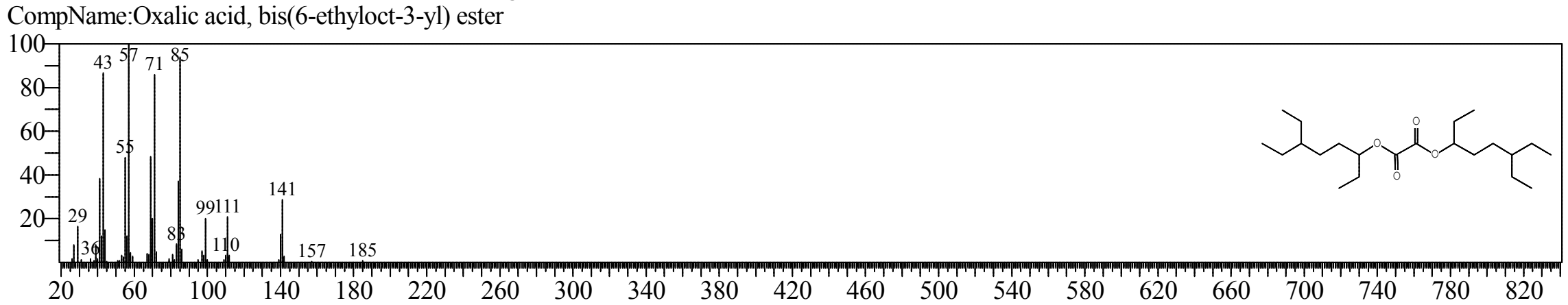

Hit#:8 Entry:129414 Library:NIST11.lib
SI:65 Formula:C18H34O4 CAS:0-00-0 MolWeight:314 RetIndex:2017
CompName:Oxalic acid, 6-ethyloct-3-yl hexyl ester

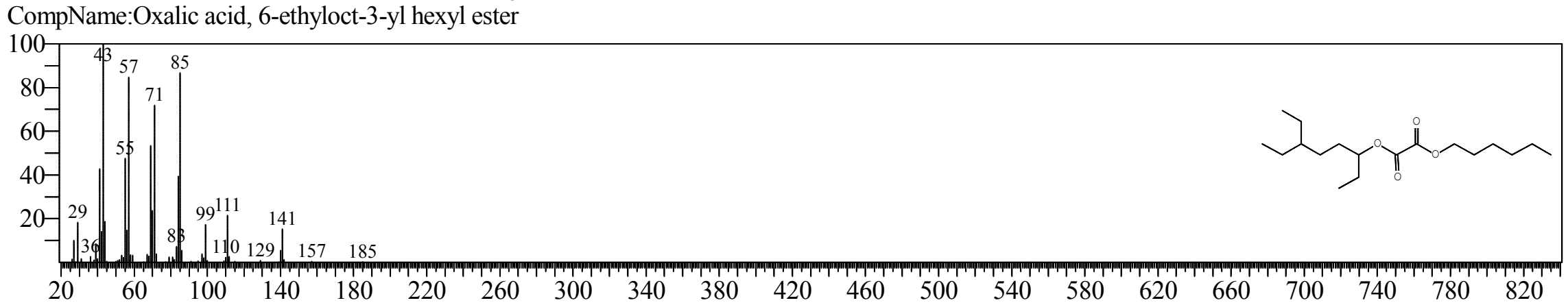

Hit#:9 Entry:109005 Library:NIST05.LIB
SI:65 Formula:C18H34O4 CAS:0-00-0 MolWeight:314 RetIndex:2017
CompName:Oxalic acid, 6-ethyloct-3-yl hexyl ester

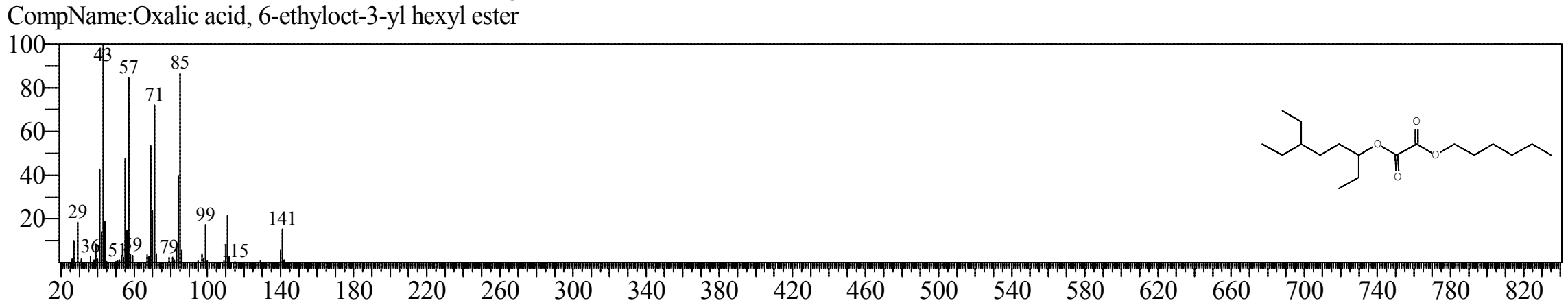

Hit#:10 Entry:25944 Library:NIST11s.lib
SI:65 Formula:C20H40O CAS:150-86-7 MolWeight:296 RetIndex:2045
CompName:Phytol $$ 2-Hexadecen-1-ol, 3,7,11,15-tetramethyl-, [R-[R*,R*-(E)]]- $$ trans-Phytol $$ 3,7,11,15-Tetramethyl-2-hexadecen-1-ol-, (2E,7R,11R

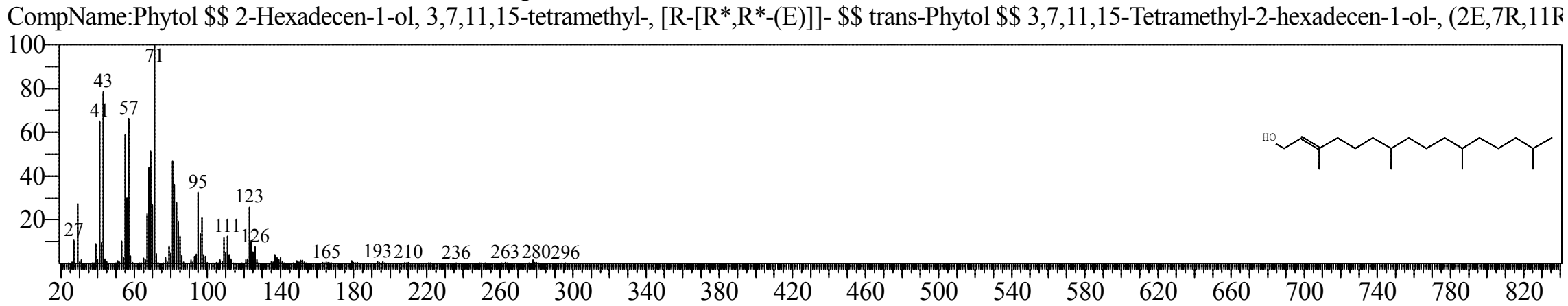

<< Target >>
Line#:14   R.Time:14.658(Scan#:1160) MassPeaks:48
RawMode:Averaged 14.650-14.667(1159-1161) BasePeak:85.15(1206)
BG Mode:Calc. from Peak Group 1 - Event 1 Scan

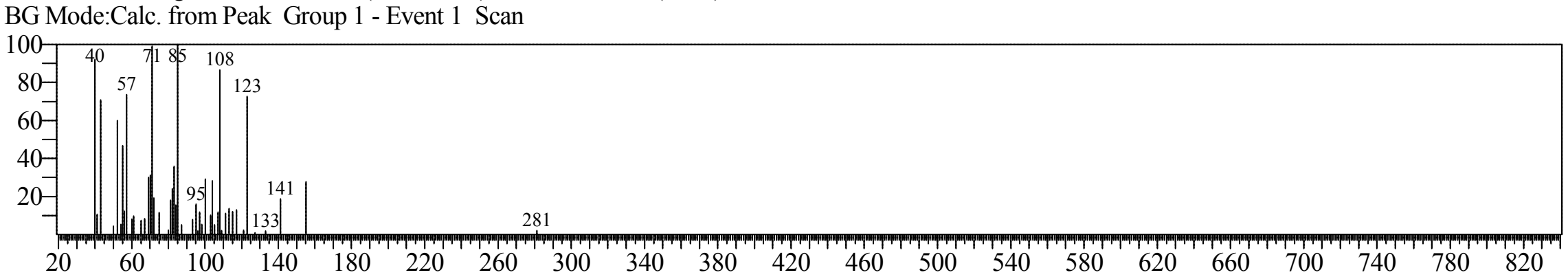

Hit#:11 Entry:26013 Library:NIST05.LIB
SI:65 Formula:C11H24O CAS:91337-07-4 MolWeight:172 RetIndex:1165
CompName:2-Isopropyl-5-methyl-1-heptanol

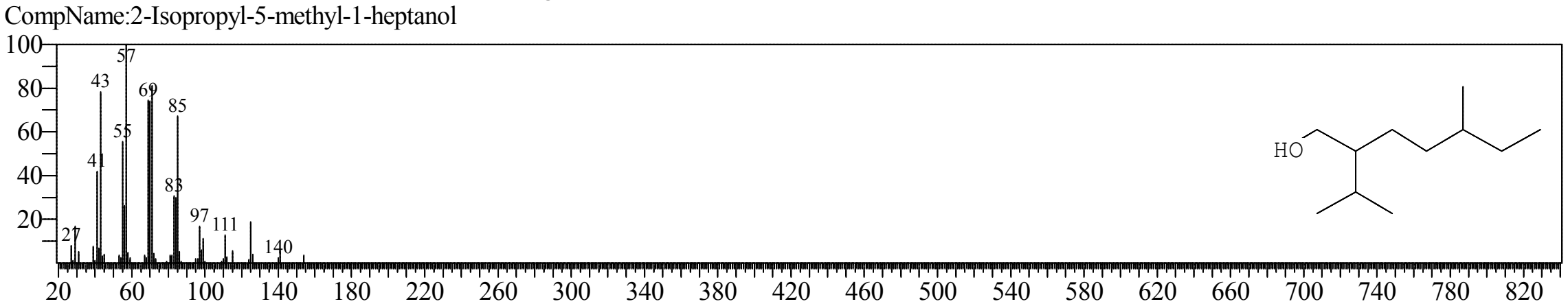

Hit#:12 Entry:145947 Library:WILEY7.LIB
SI:65 Formula:C17 H36 CAS:14905-56-7 MolWeight:240 RetIndex:0
CompName:Tetradecane, 2,6,10-trimethyl- (CAS) 2,6,10-Trimethyltetradecane $$

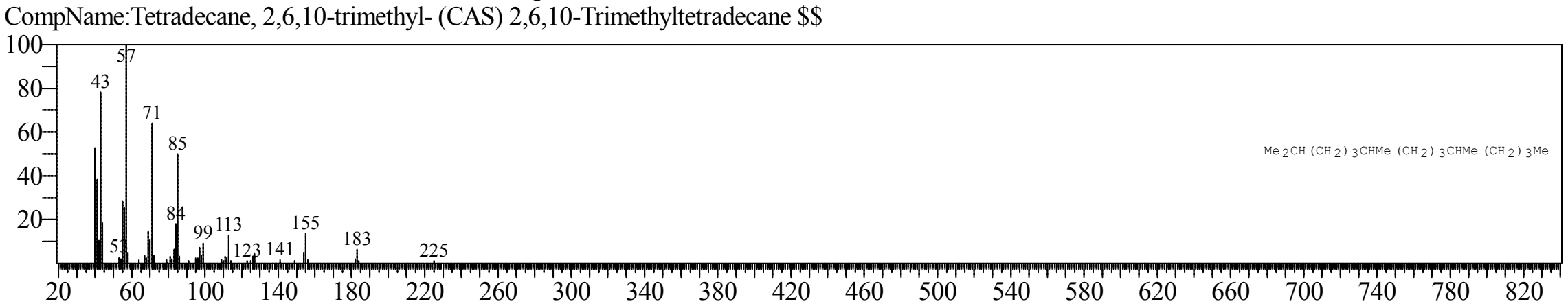

Hit#:13 Entry:109004 Library:NIST05.LIB
SI:65 Formula:C18H34O4 CAS:0-00-0 MolWeight:314 RetIndex:1953
CompName:Oxalic acid, 6-ethyloct-3-yl isohexyl ester

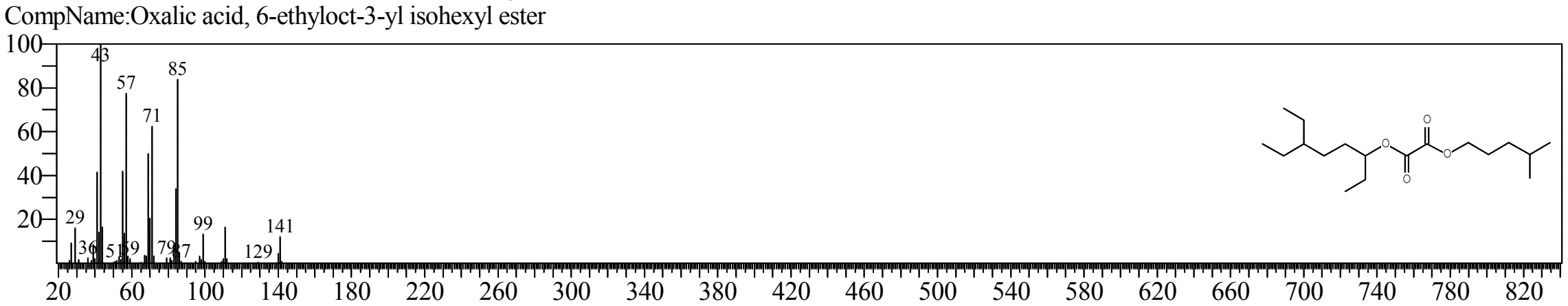

Hit#:14 Entry:100142 Library:NIST05.LIB
SI:65 Formula:C20H42O CAS:2456-28-2 MolWeight:298 RetIndex:2085
CompName:Decane, 1,1'-oxybis- $$ Decyl ether $$ Capric ether $$ Didecyl ether $$ di-n-Decyl ether $$ 1-(Decyloxy)decane # $$

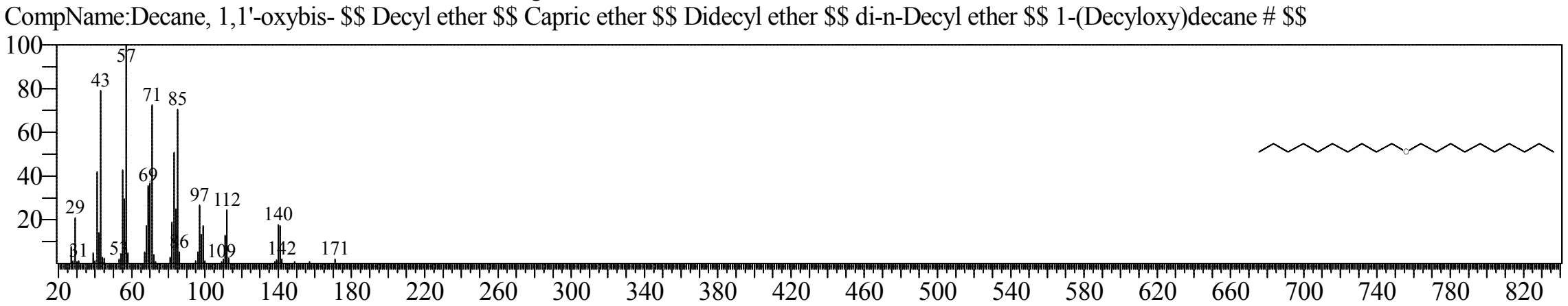

Hit#:15 Entry:129413 Library:NIST11.lib
SI:65 Formula:C18H34O4 CAS:0-00-0 MolWeight:314 RetIndex:1953
CompName:Oxalic acid, 6-ethyloct-3-yl isohexyl ester

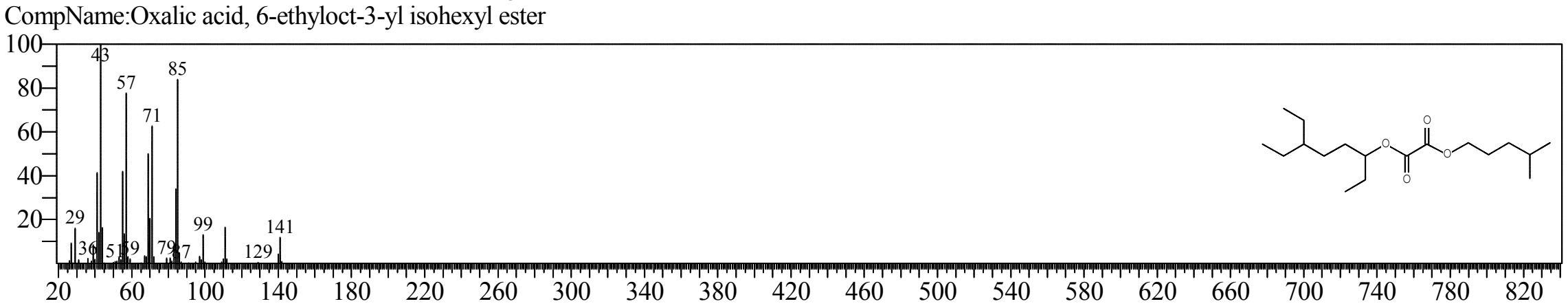

<< Target >>
Line#:14   R.Time:14.658(Scan#:1160) MassPeaks:48
RawMode:Averaged 14.650-14.667(1159-1161) BasePeak:85.15(1206)
BG Mode:Calc. from Peak Group 1 - Event 1 Scan

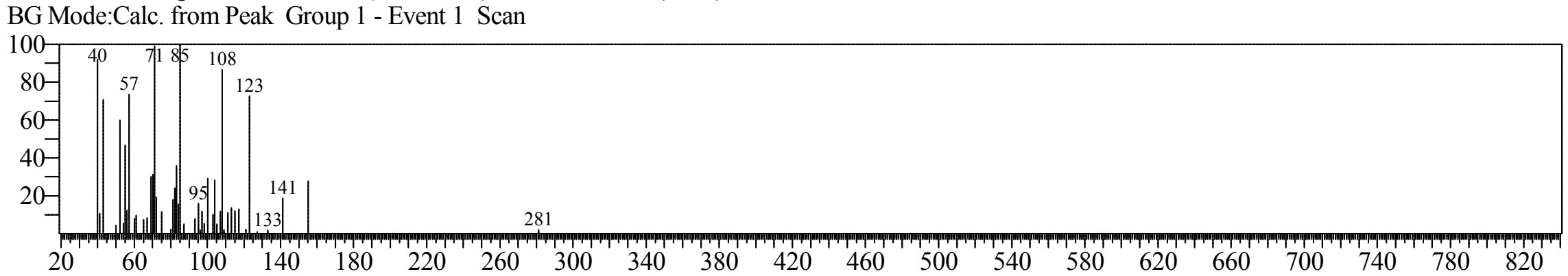

Hit#:16 Entry:115315 Library:NIST05.LIB
SI:65 Formula:C18H30O5 CAS:108533-24-0 MolWeight:326 RetIndex:2021
CompName:2-Butyloxycarbonyloxy-1,1,10-trimethyl-6,9-epidioxydecalin

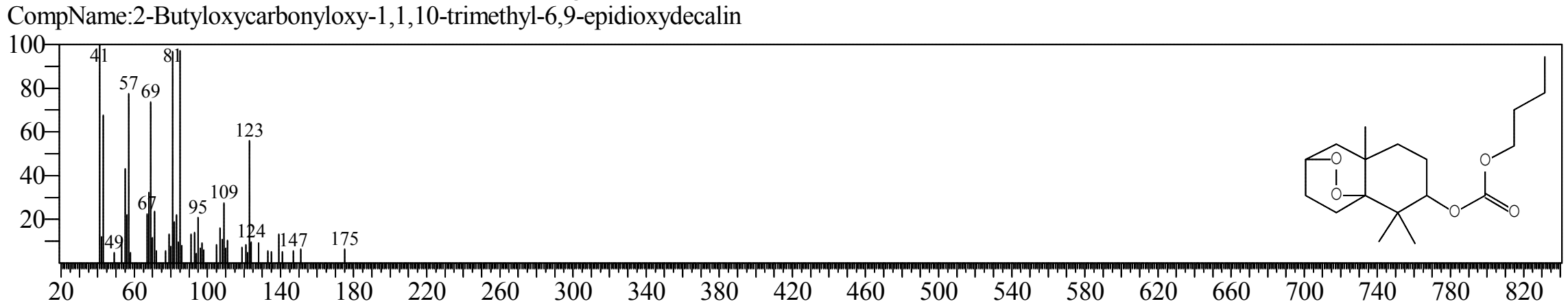

Hit#:17 Entry:96493 Library:NIST11.lib
SI:65 Formula:C15H28O4 CAS:0-00-0 MolWeight:272 RetIndex:1719
CompName:Oxalic acid, 6-ethyloct-3-yl propyl ester

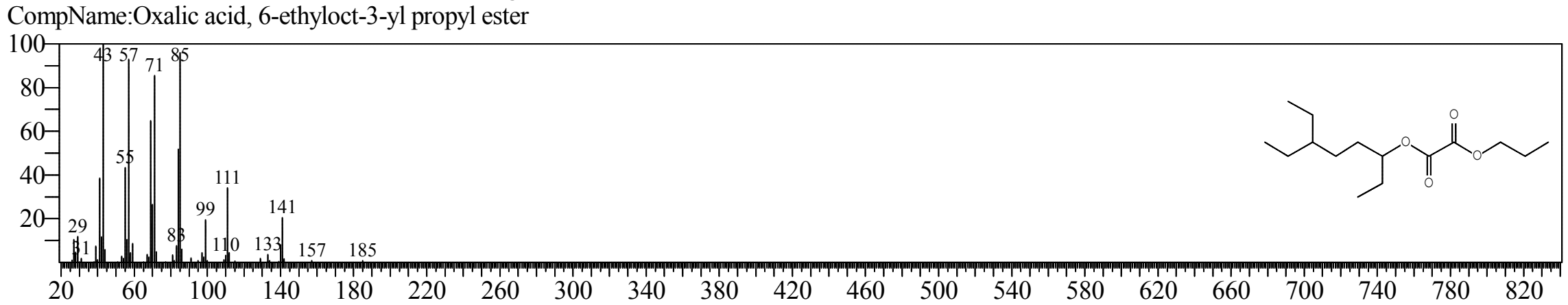

Hit#:18 Entry:84513 Library:NIST05.LIB
SI:65 Formula:C15H28O4 CAS:0-00-0 MolWeight:272 RetIndex:1719
CompName:Oxalic acid, 6-ethyloct-3-yl propyl ester

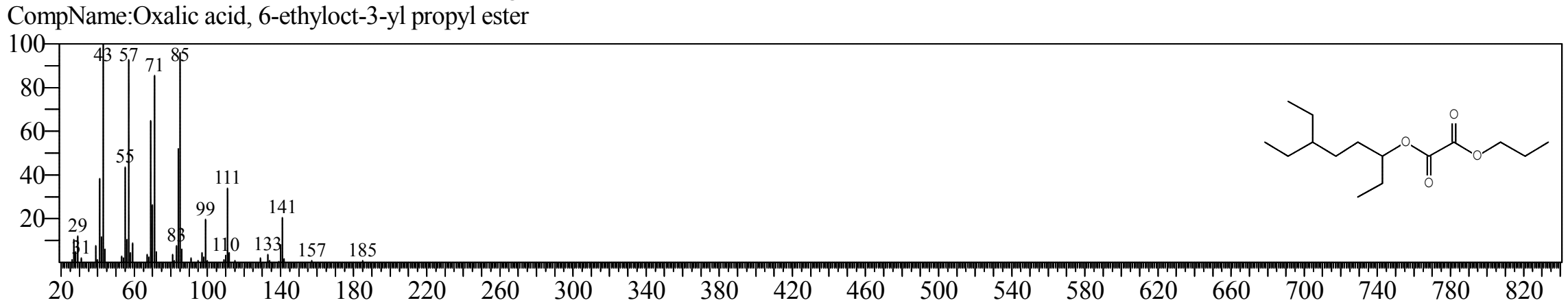

Hit#:19 Entry:15972 Library:NIST05s.LIB
SI:65 Formula:C12H22O2 CAS:7493-72-3 MolWeight:198 RetIndex:1371
CompName:Allyl nonanoate $$ Allyl pelargonate $$ Nonanoic acid, 2-propenyl ester $$ Nonanoic acid, allyl ester $$ Allyl n-nonanoate $$

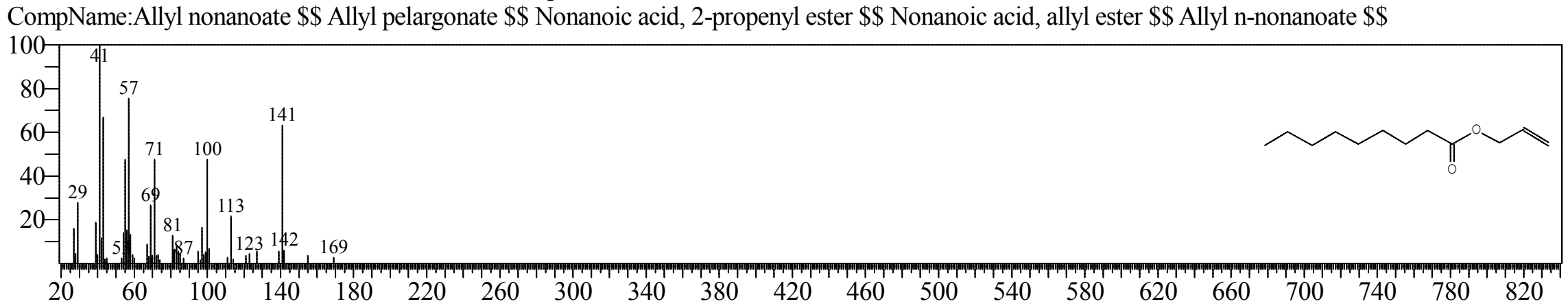

Hit#:20 Entry:84404 Library:NIST11.lib
SI:65 Formula:C16H32O2 CAS:0-00-0 MolWeight:256 RetIndex:1715
CompName:Isovaleric acid, undecyl ester

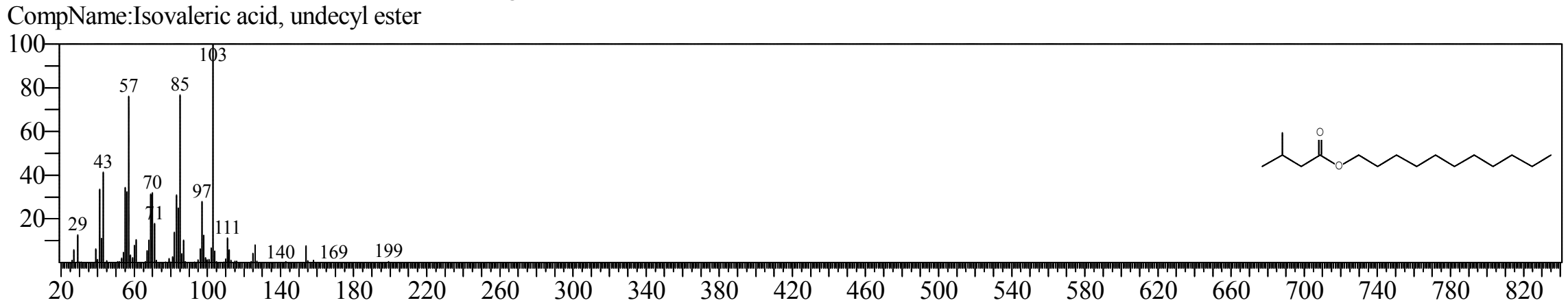

<< Target >>
Line#:15   R.Time:14.800(Scan#:1177) MassPeaks:44
RawMode:Averaged 14.792-14.808(1176-1178) BasePeak:108.15(1093)
BG Mode:Calc. from Peak Group 1 - Event 1 Scan

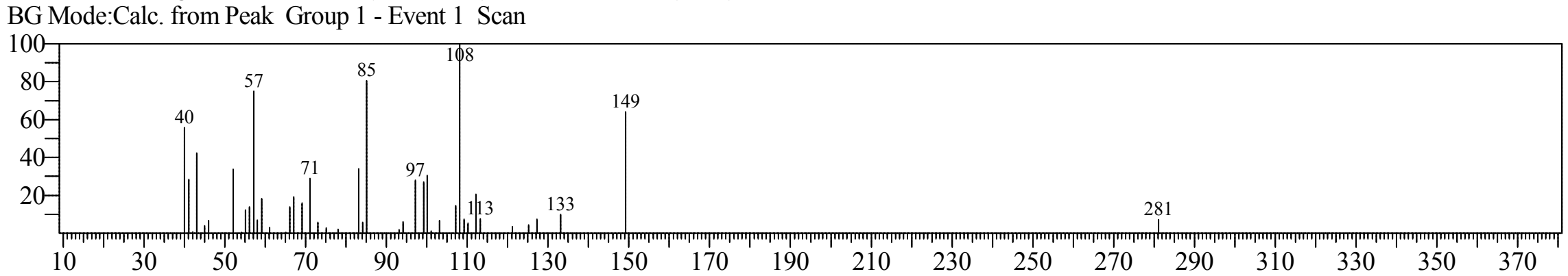

Hit#:1 Entry:61573 Library:NIST05.LIB
SI:65 Formula:C15H22O2 CAS:5457-63-6 MolWeight:234 RetIndex:1770
CompName:Octanoic acid, 2-methylphenyl ester $$ 2-Methylphenyl octanoate # $$

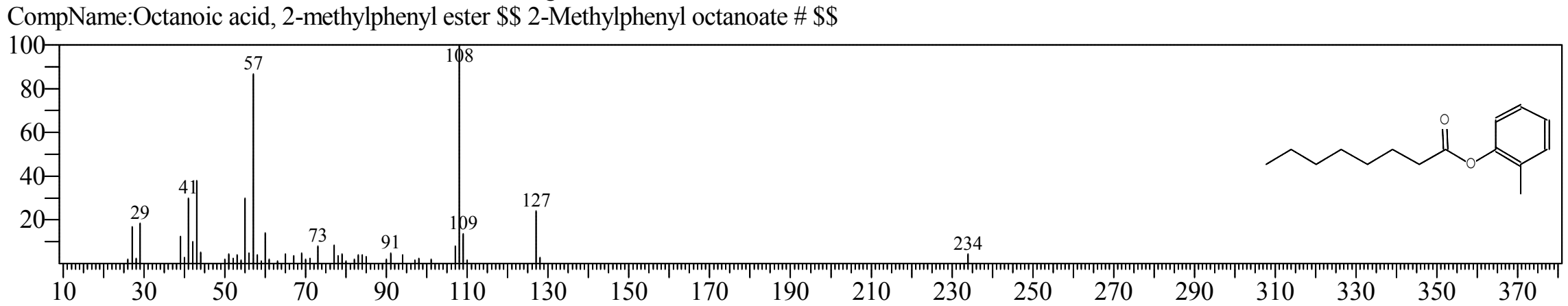

Hit#:2 Entry:143133 Library:NIST11.lib
SI:65 Formula:C20H28O4 CAS:0-00-0 MolWeight:332 RetIndex:2442
CompName:Fumaric acid, nonyl 3-methylphenyl ester

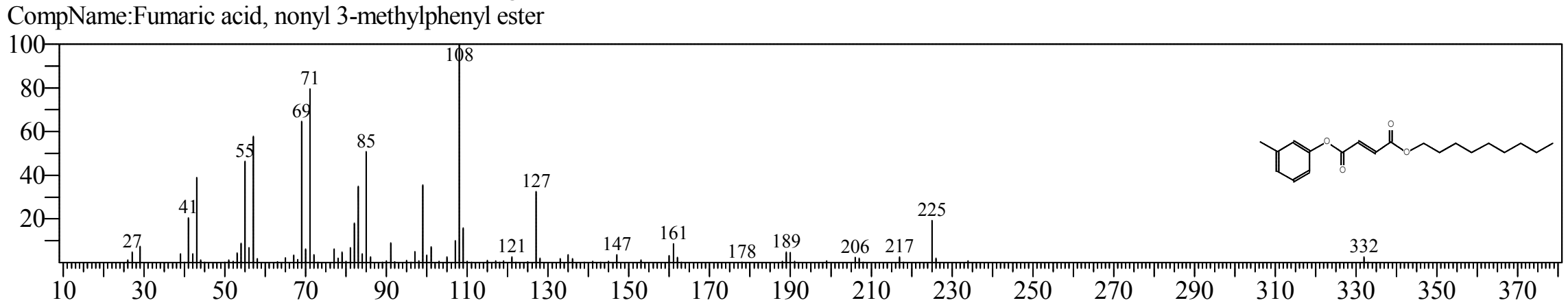

Hit#:3 Entry:170295 Library:NIST11.lib
SI:64 Formula:C23H34O4 CAS:0-00-0 MolWeight:374 RetIndex:2740
CompName:Fumaric acid, dodecyl 3-methylphenyl ester

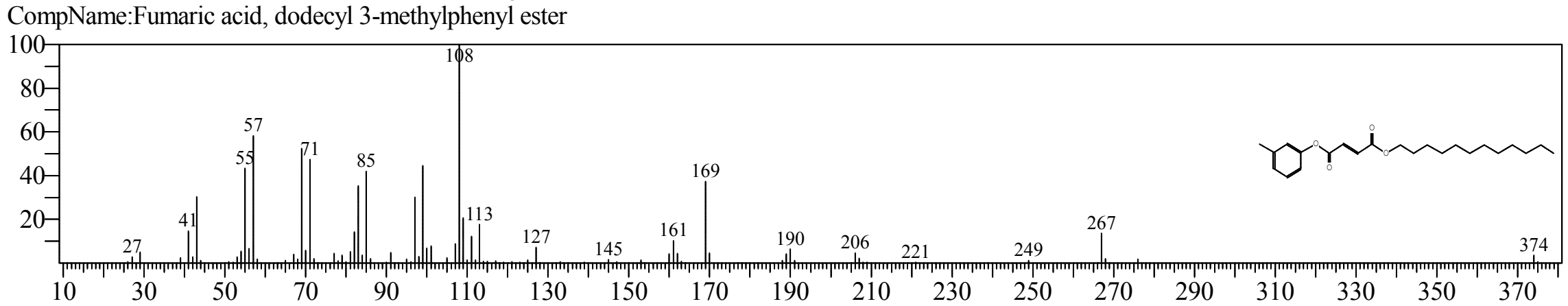

Hit#:4 Entry:99570 Library:NIST11.lib
SI:64 Formula:C14H29Br CAS:74036-95-6 MolWeight:276 RetIndex:1645
CompName:2-Bromotetradecane

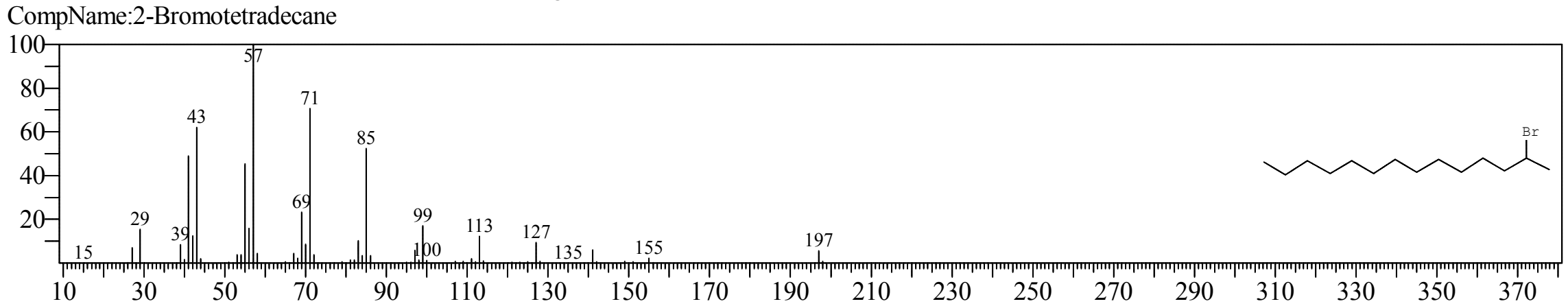

Hit#:5 Entry:161987 Library:NIST11.lib
SI:64 Formula:C22H32O4 CAS:0-00-0 MolWeight:360 RetIndex:2641
CompName:Fumaric acid, 3-methylphenyl undecyl ester

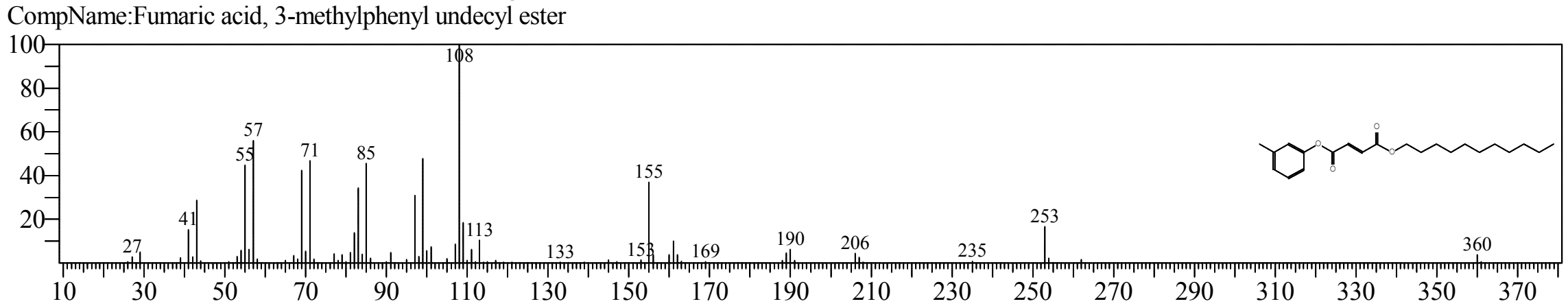

<< Target >>
Line#:15 R.Time:14.800(Scan#:1177) MassPeaks:44
RawMode:Averaged 14.792-14.808(1176-1178) BasePeak:108.15(1093)
BG Mode:Calc. from Peak Group 1 - Event 1 Scan

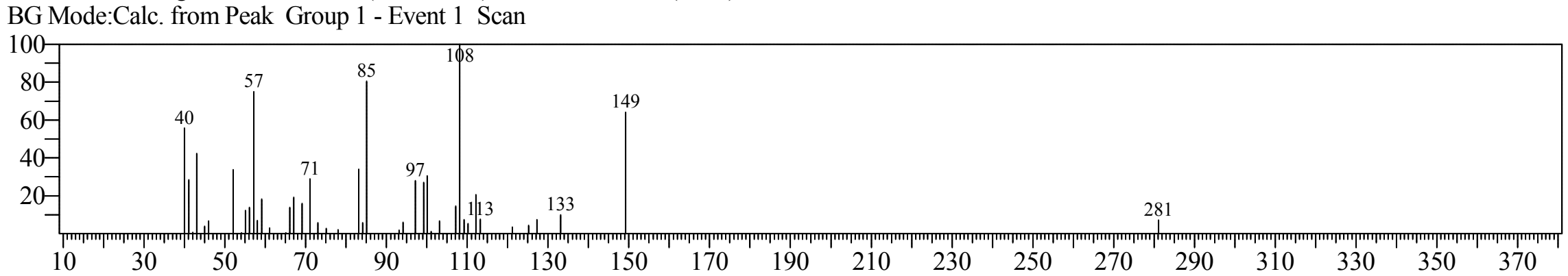

Hit#:6 Entry:21842 Library:NIST11.lib
SI:63 Formula:C5H9BrO CAS:0-00-0 MolWeight:164 RetIndex:867
CompName:2-(2-Bromoethyl)-3-methyl-oxirane

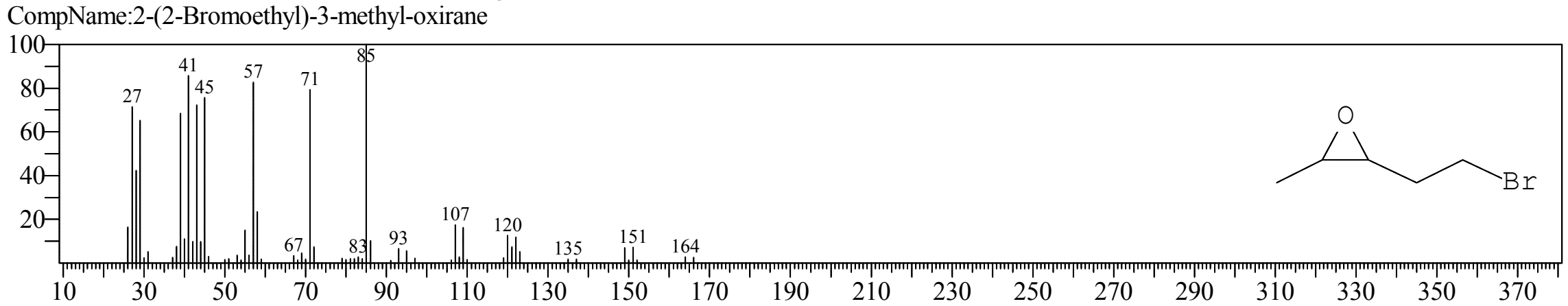

Hit#:7 Entry:20988 Library:NIST05.LIB
SI:63 Formula:C5H9BrO CAS:0-00-0 MolWeight:164 RetIndex:867
CompName:2-(2-Bromoethyl)-3-methyl-oxirane

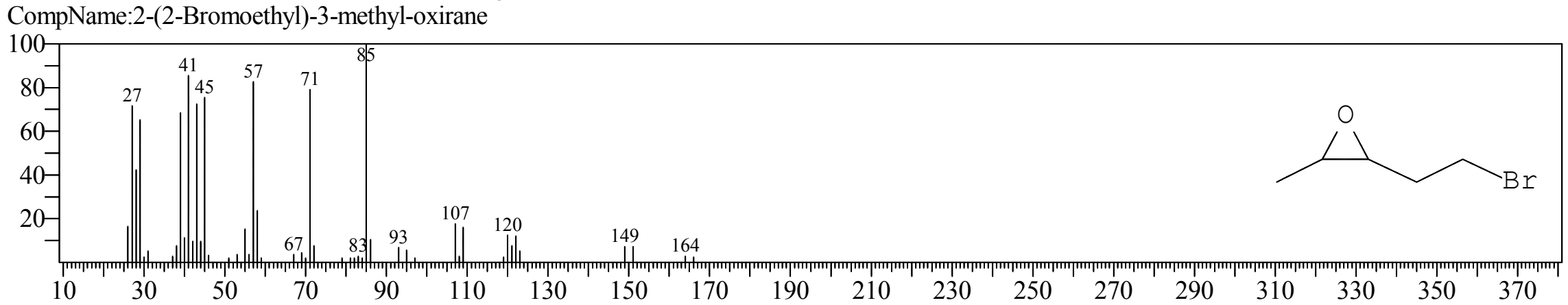

Hit#:8 Entry:153076 Library:NIST11.lib
SI:63 Formula:C21H30O4 CAS:0-00-0 MolWeight:346 RetIndex:2542
CompName:Fumaric acid, decyl 3-methylphenyl ester

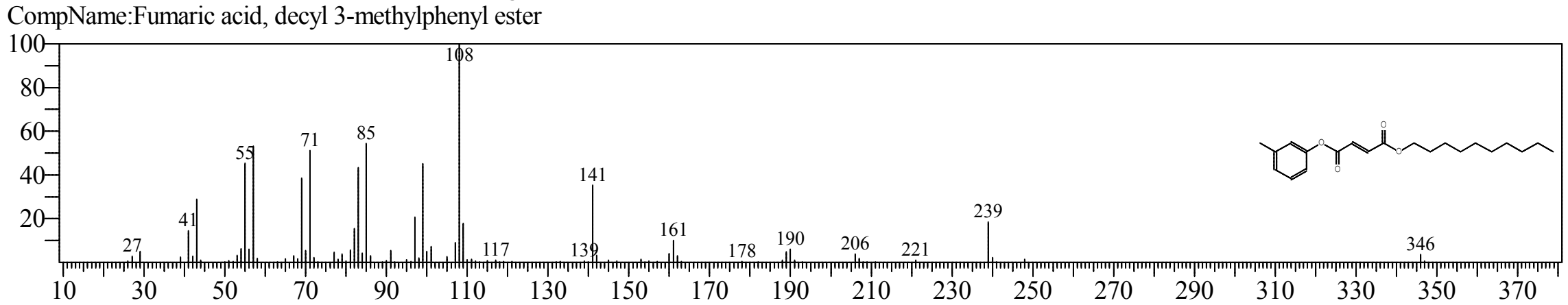

Hit#:9 Entry:52311 Library:WILEY7.LIB
SI:63 Formula:C5 H9 BR O CAS:0-00-0 MolWeight:164 RetIndex:0
CompName:2-(2-BROMO-ETHYL)-3-METHYL-OXIRANE $$

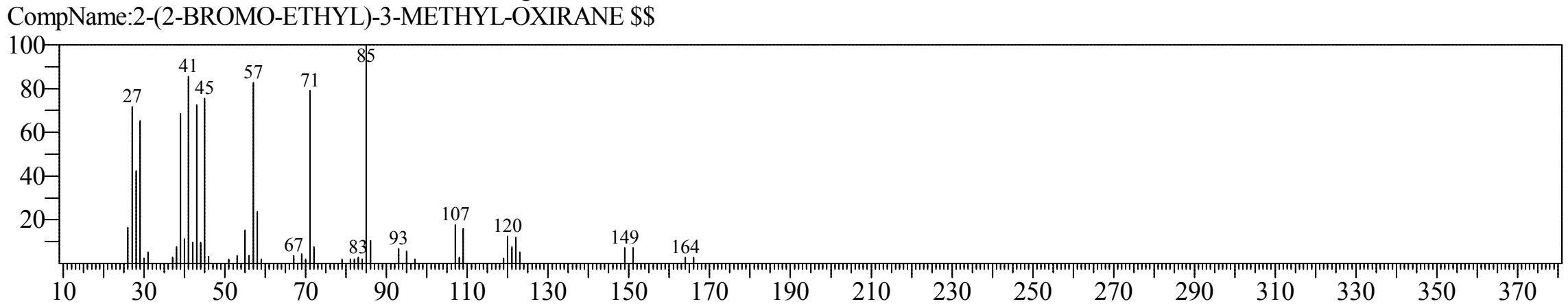

Hit#:10 Entry:79348 Library:NIST05.LIB
SI:63 Formula:C13H28O3S CAS:0-00-0 MolWeight:264 RetIndex:1937
CompName:Sulfurous acid, hexyl heptyl ester

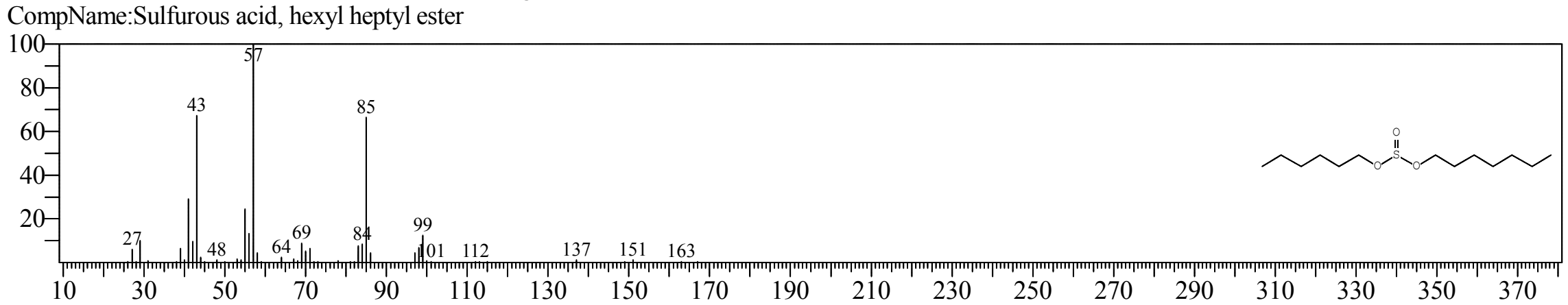

<< Target >>
Line#:15 R.Time:14.800(Scan#:1177) MassPeaks:44
RawMode:Averaged 14.792-14.808(1176-1178) BasePeak:108.15(1093)
BG Mode:Calc. from Peak Group 1 - Event 1 Scan

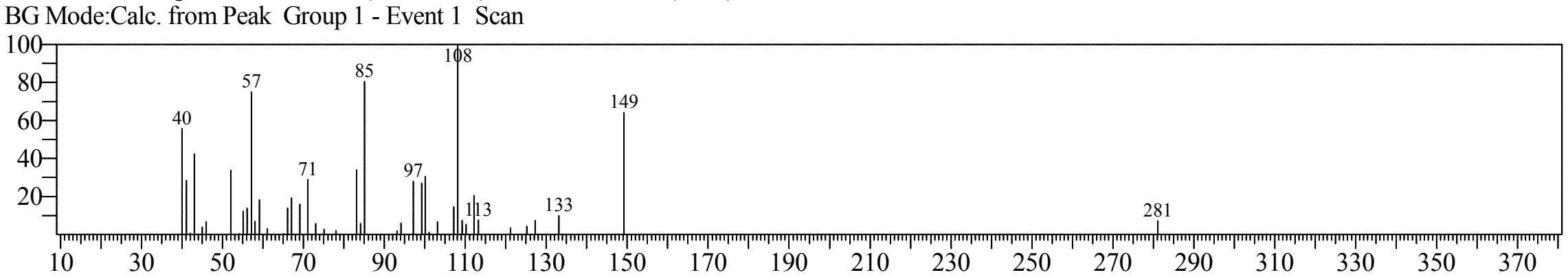

Hit#:11 Entry:69720 Library:NIST05.LIB
SI:63 Formula:C12H25Br CAS:13187-99-0 MolWeight:248 RetIndex:1446
CompName:2-Bromo dodecane $$ Dodecane, 2-bromo- $$

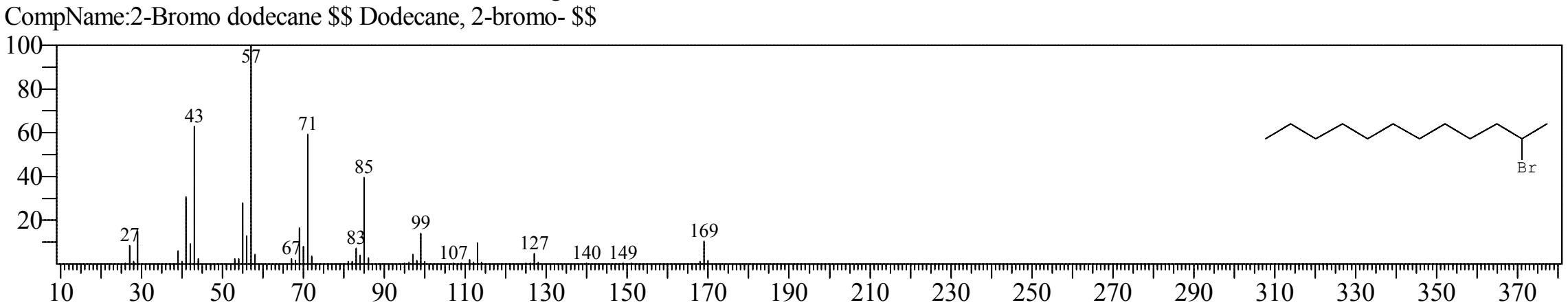

Hit#:12 Entry:89994 Library:NIST11.lib
SI:63 Formula:C13H28O3S CAS:0-00-0 MolWeight:264 RetIndex:1937
CompName:Sulfurous acid, hexyl heptyl ester

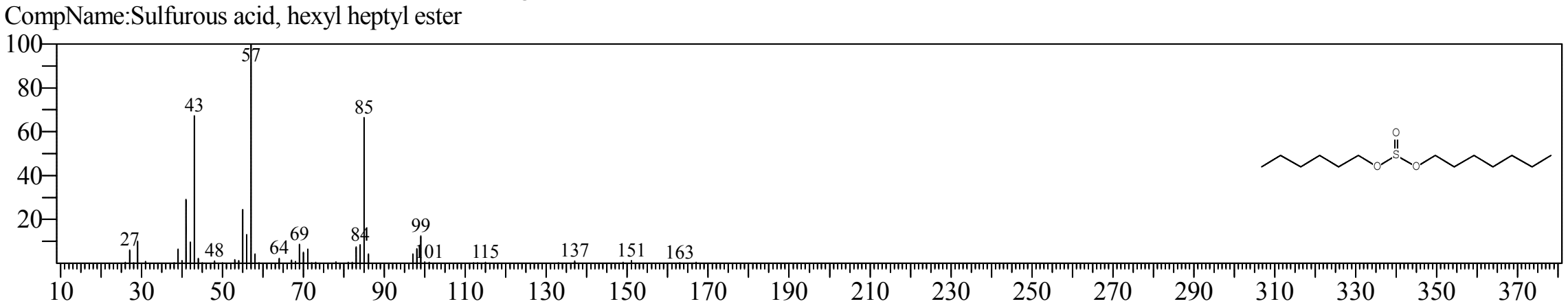

Hit#:13 Entry:87908 Library:NIST05.LIB
SI:63 Formula:C14H30O3S CAS:0-00-0 MolWeight:278 RetIndex:2036
CompName:Sulfurous acid, hexyl octyl ester

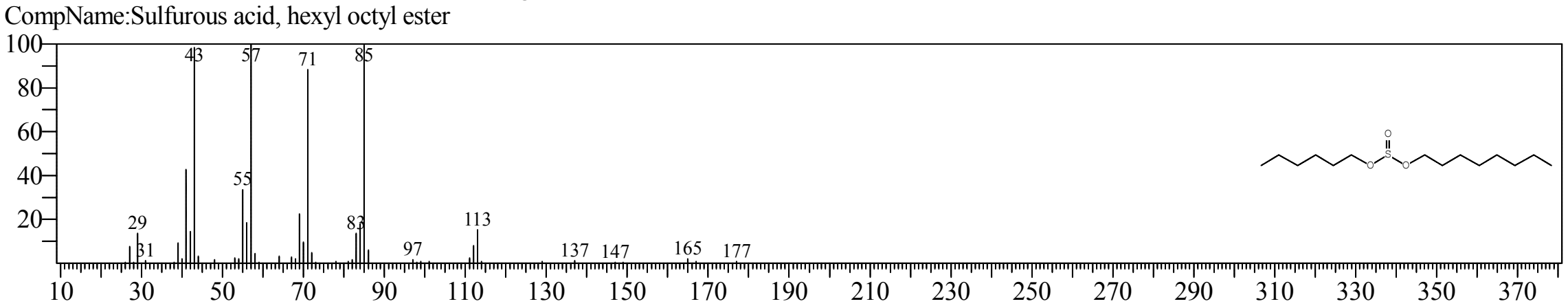

Hit#:14 Entry:77005 Library:WILEY7.LIB
SI:63 Formula:C12 H24 O CAS:123-18-2 MolWeight:184 RetIndex:0
CompName:2,6,8-Trimethyl-4-nonanone $$ 4-Nonanone, 2,6,8-trimethyl- $$ Isobutyl heptyl ketone $$

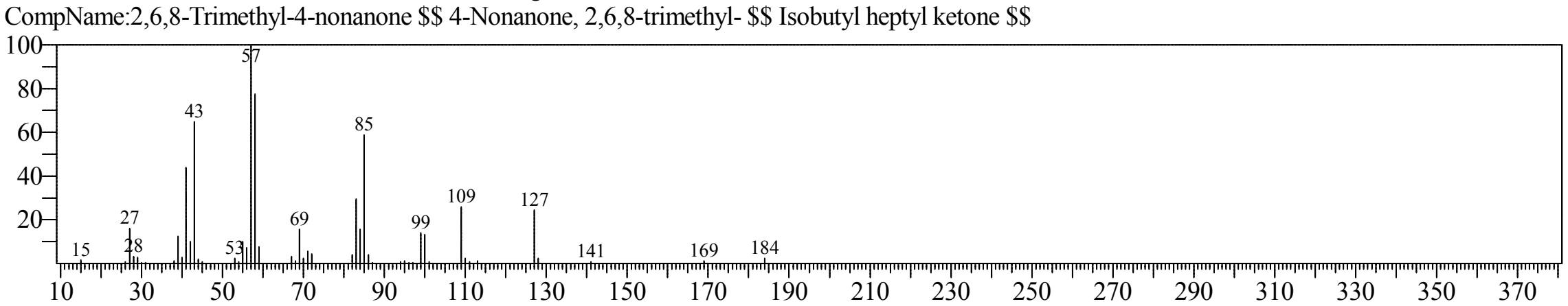

Hit#:15 Entry:100964 Library:NIST11.lib
SI:63 Formula:C14H30O3S CAS:0-00-0 MolWeight:278 RetIndex:2036
CompName:Sulfurous acid, hexyl octyl ester

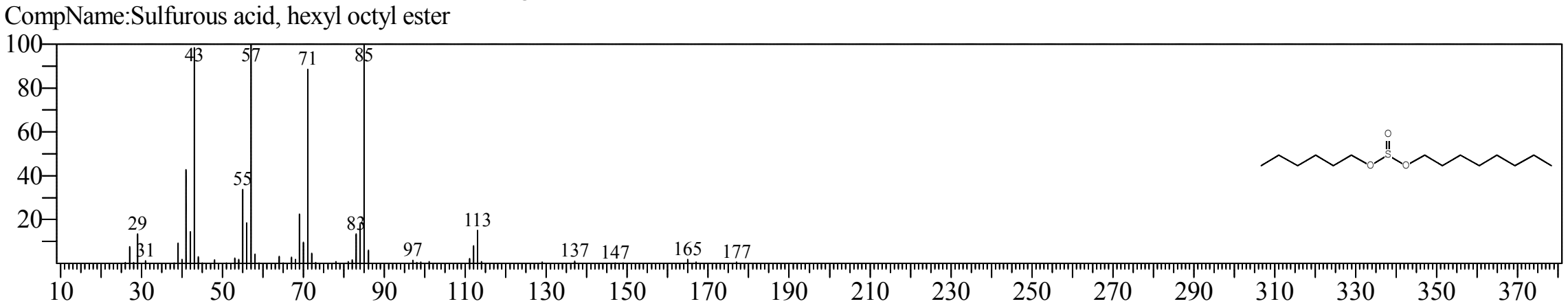

<< Target >>
Line#:15 R.Time:14.800(Scan#:1177) MassPeaks:44
RawMode:Averaged 14.792-14.808(1176-1178) BasePeak:108.15(1093)
BG Mode:Calc. from Peak Group 1 - Event 1 Scan

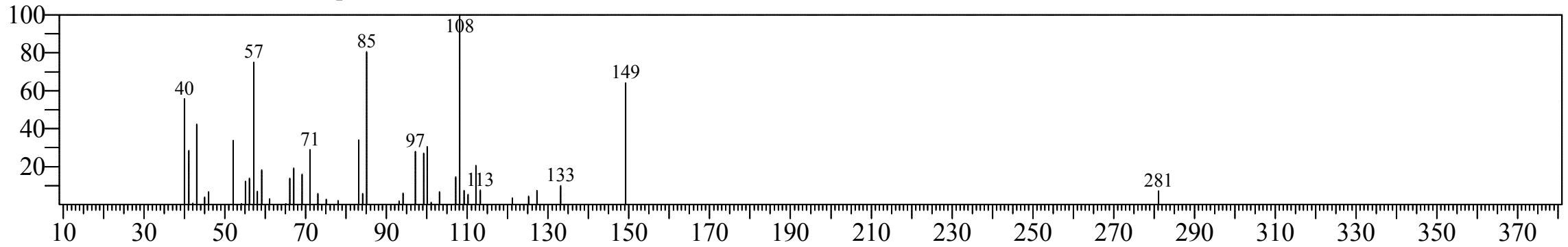

Hit#:16 Entry:145947 Library:WILEY7.LIB
SI:63 Formula:C17 H36 CAS:14905-56-7 MolWeight:240 RetIndex:0
CompName:Tetradecane, 2,6,10-trimethyl- (CAS) 2,6,10-Trimethyltetradecane $$

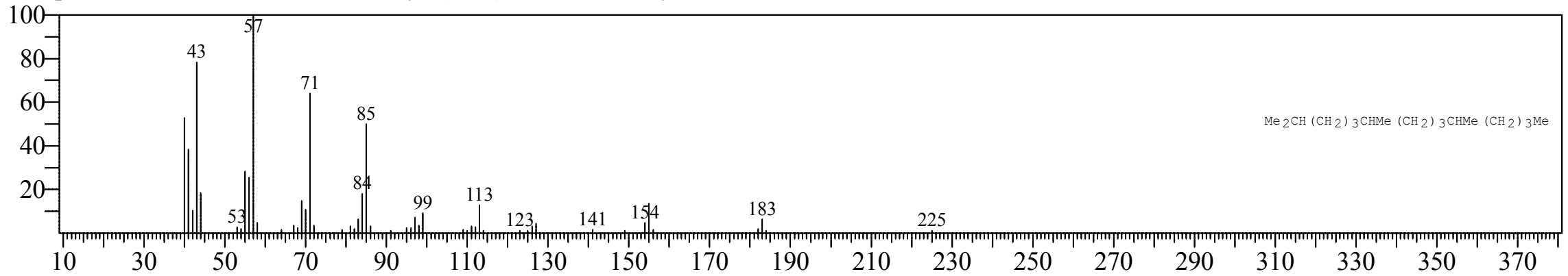

Hit#:17 Entry:184053 Library:NIST11.lib
SI:63 Formula:C25H40O4 CAS:0-00-0 MolWeight:404 RetIndex:2931
CompName:Succinic acid, 3-methylphenyl tetradecyl ester

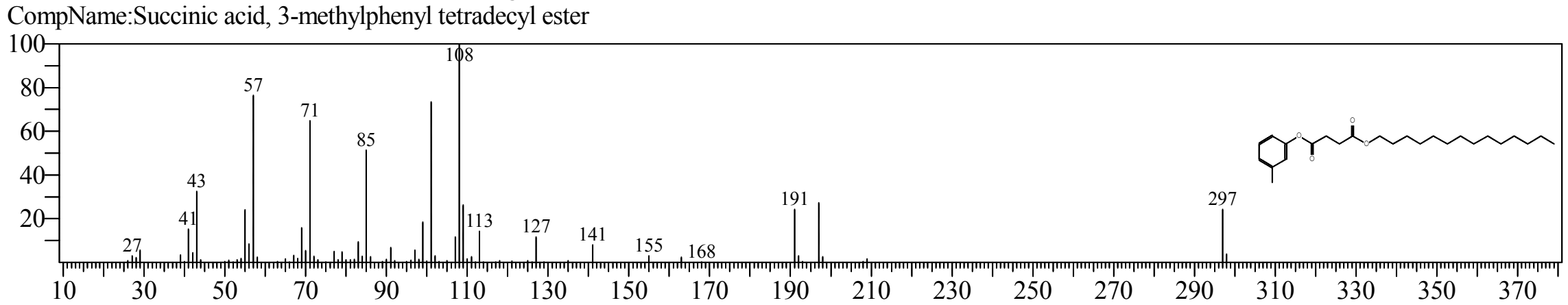

Hit#:18 Entry:100142 Library:NIST05.LIB
SI:63 Formula:C20H42O CAS:2456-28-2 MolWeight:298 RetIndex:2085
CompName:Decane, 1,1'-oxybis- $$ Decyl ether $$ Capric ether $$ Didecyl ether $$ di-n-Decyl ether $$ 1-(Decyloxy)decane # $$

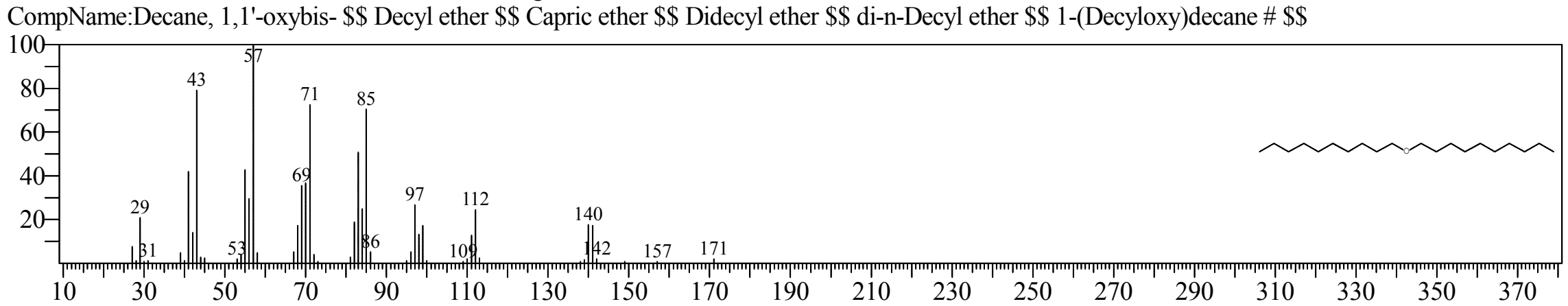

Hit#:19 Entry:229274 Library:WILEY7.LIB
SI:62 Formula:C17 H25 N3 O3 CAS:0-00-0 MolWeight:319 RetIndex:0
CompName:CYANO-(5'-ETHOXY-1,2,3,4,3',4'-HEXAHYDRO-2'H-[2,2']BIPYRROLYL-5-YLIDENE)-ACETIC $$

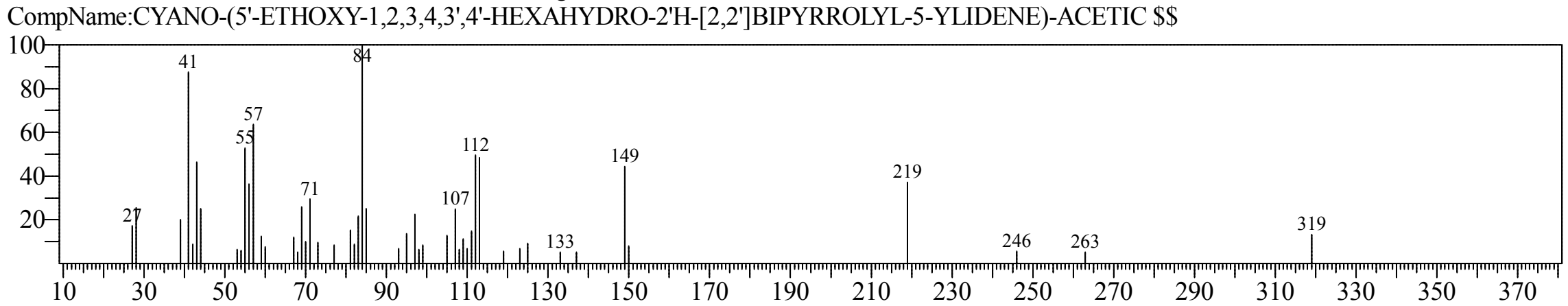

Hit#:20 Entry:188668 Library:NIST11.lib
SI:62 Formula:C26H42O4 CAS:0-00-0 MolWeight:418 RetIndex:3031
CompName:Succinic acid, 3-methylphenyl pentadecyl ester

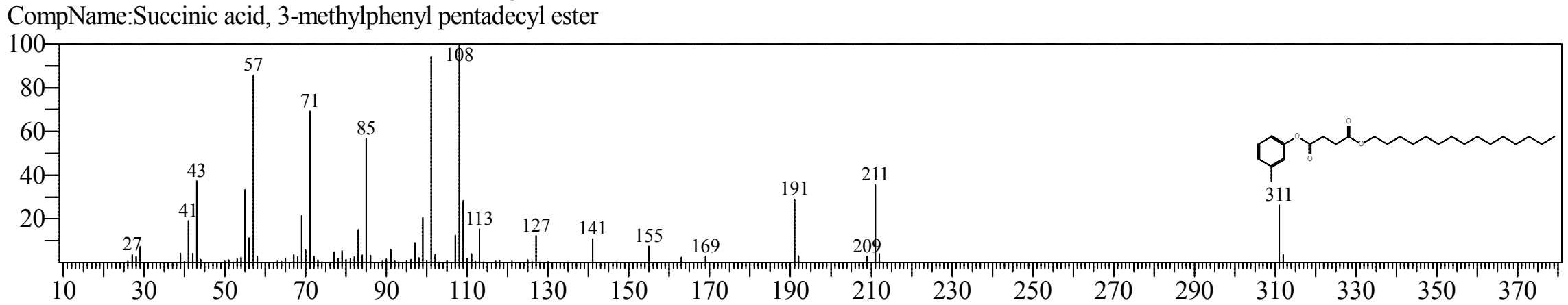

<< Target >>
Line#:16 R.Time:14.833(Scan#:1181) MassPeaks:36
RawMode:Averaged 14.825-14.842(1180-1182) BasePeak:85.15(700)
BG Mode:Calc. from Peak Group 1 - Event 1 Scan

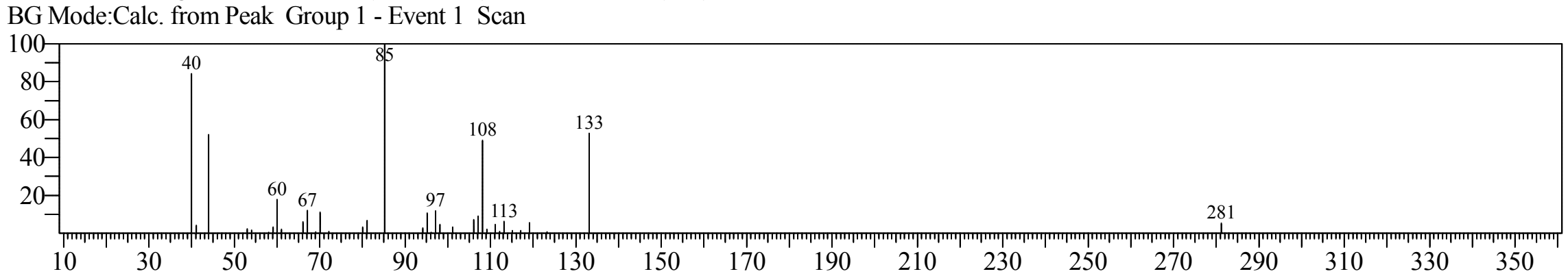

Hit#:1 Entry:11949 Library:NIST05s.LIB
SI:57 Formula:C10H18O2 CAS:706-14-9 MolWeight:170 RetIndex:1383
CompName:2(3H)-Furanone, 5-hexyldihydro- $$ .gamma.-Decalactone $$ .gamma.-Decanolactone $$ .gamma.-Hexyl-.gamma.-butyrolactone $$ .gamma.-H

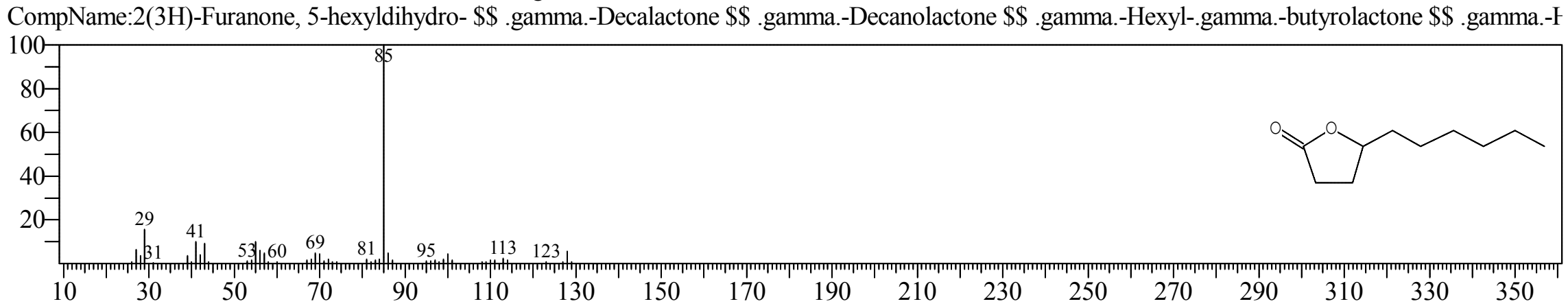

Hit#:2 Entry:42939 Library:WILEY7.LIB
SI:57 Formula:C10 H18 O CAS:57590-19-9 MolWeight:154 RetIndex:0
CompName:ARTEMISIA ALCOHOL $$

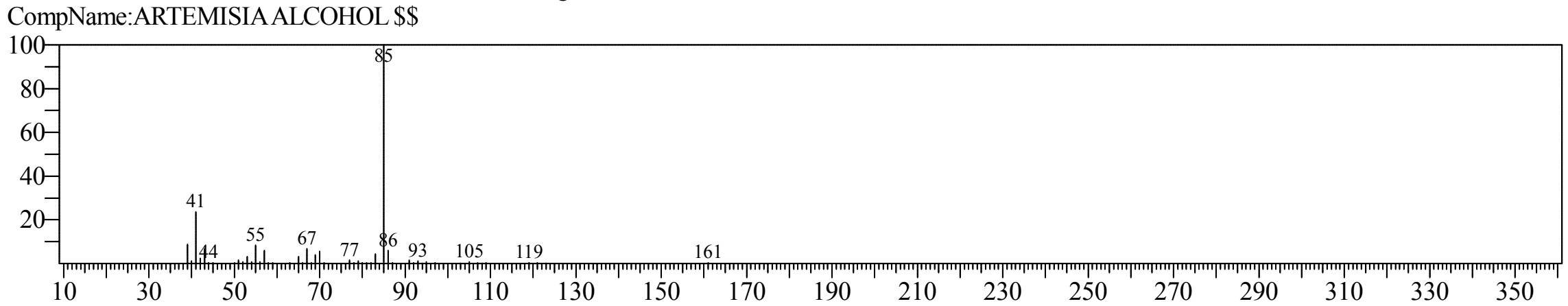

Hit#:3 Entry:143817 Library:WILEY7.LIB
SI:57 Formula:C15 H26 O2 CAS:0-00-0 MolWeight:238 RetIndex:0
CompName:1,7,7-trimethyl-2-endo-(1-oxacyclohex-2-yloxy)bicyclo[2.2.1]heptane $$

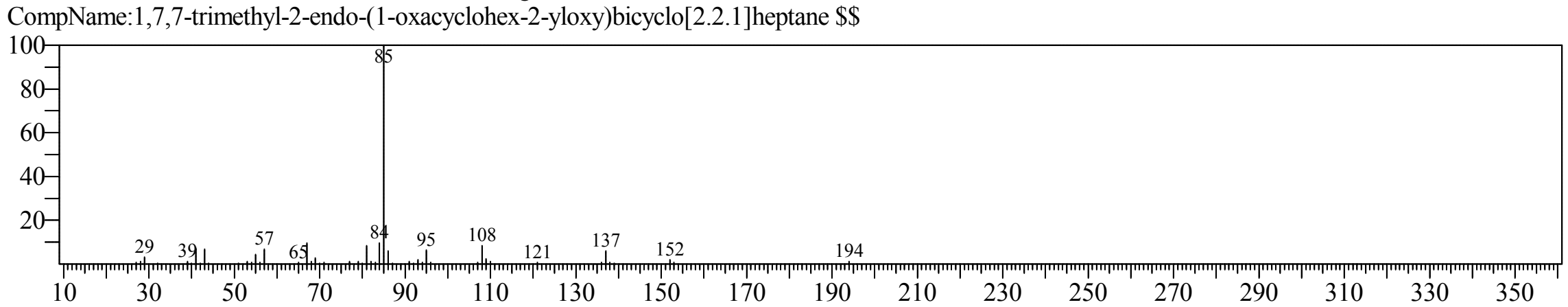

Hit#:4 Entry:6701 Library:NIST05.LIB
SI:57 Formula:C6H14BNO CAS:31748-10-4 MolWeight:127 RetIndex:0
CompName:1,3,2-Oxazaborolane, 2-butyl- $$ Ethanolamine cyclic butaneboronate $$ 2-Butyl-1,3,2-oxazaborolidine # $$

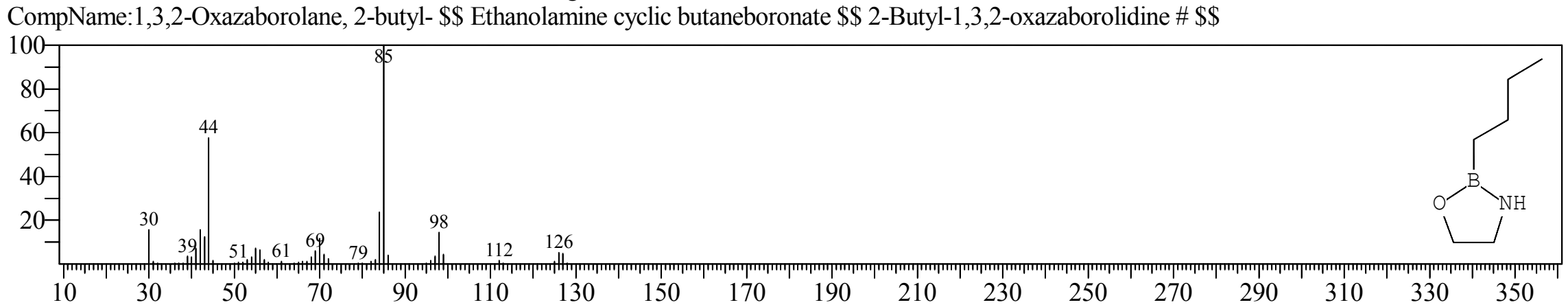

Hit#:5 Entry:81127 Library:NIST11.lib
SI:56 Formula:C15H24O3 CAS:0-00-0 MolWeight:252 RetIndex:1878
CompName:10-(Tetrahydro-pyran-2-yloxy)-tricyclo[4.2.1.1(2,5)]decan-9-ol

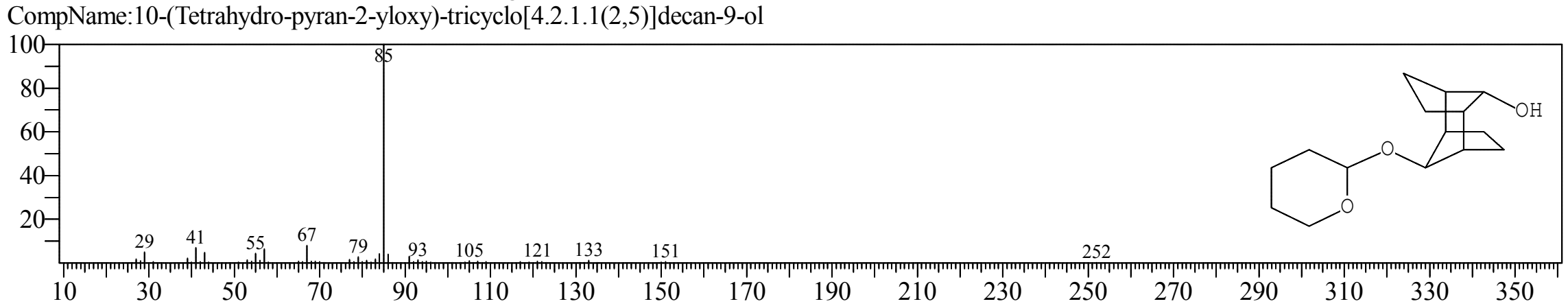

<< Target >>
Line#:16 R.Time:14.833(Scan#:1181) MassPeaks:36
RawMode:Averaged 14.825-14.842(1180-1182) BasePeak:85.15(700)
BG Mode:Calc. from Peak Group 1 - Event 1 Scan

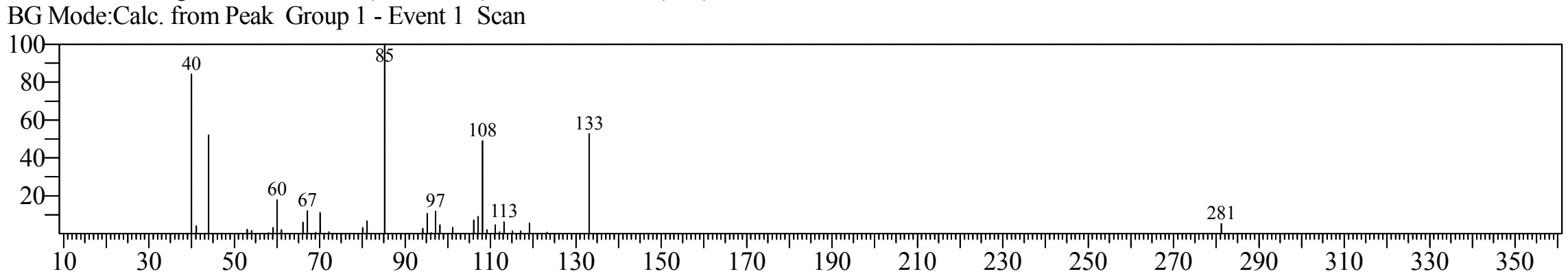

Hit#:6 Entry:72397 Library:NIST05.LIB
SI:56 Formula:C15H24O3 CAS:0-00-0 MolWeight:252 RetIndex:1878
CompName:10-(Tetrahydro-pyran-2-yloxy)-tricyclo[4.2.1.1(2,5)]decan-9-ol

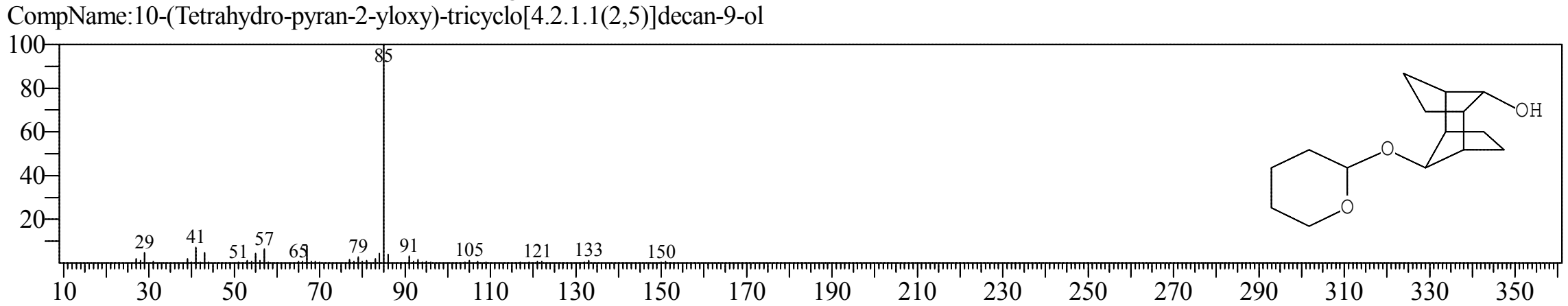

Hit#:7 Entry:19543 Library:WILEY7.LIB
SI:56 Formula:C5 H8 N2 O2 CAS:41138-17-4 MolWeight:128 RetIndex:0
CompName:2-Butenediamide, 2-methyl-, (Z)- (CAS) Citraconic diamide $$ Citraconamide $$ Maleamide, 2-methyl- $$

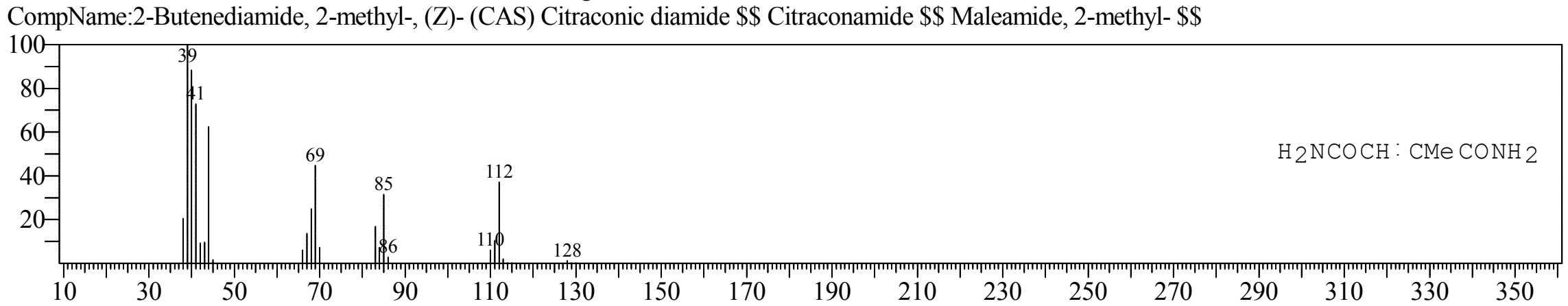

Hit#:8 Entry:160080 Library:WILEY7.LIB
SI:56 Formula:C15 H24 O3 CAS:0-00-0 MolWeight:252 RetIndex:0
CompName:10-(TETRAHYDRO-PYRAN-2-YLOXY)-TRICYCLO[4.2.1.1 2,5]DECAN-9-OL $$

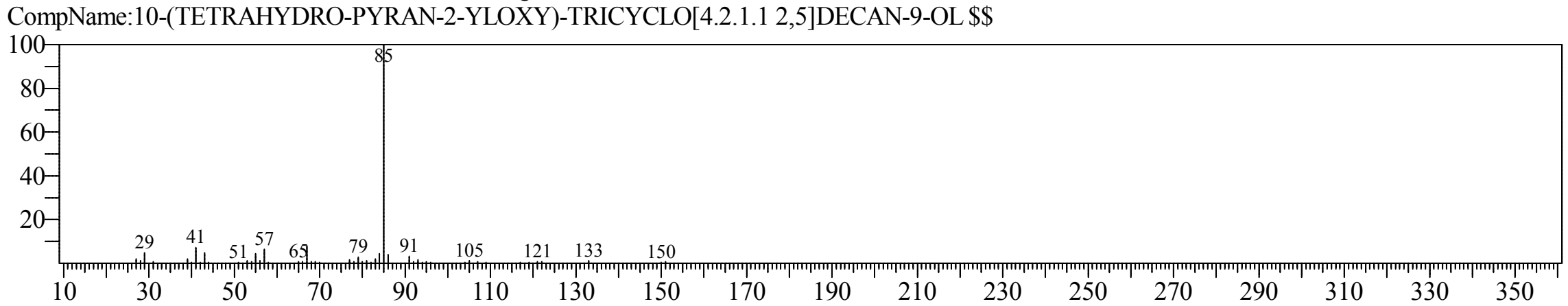

Hit#:9 Entry:60441 Library:WILEY7.LIB
SI:56 Formula:C10 H18 O2 CAS:0-00-0 MolWeight:170 RetIndex:0
CompName:4-HEXYLBUTAN-4-OLIDE $$

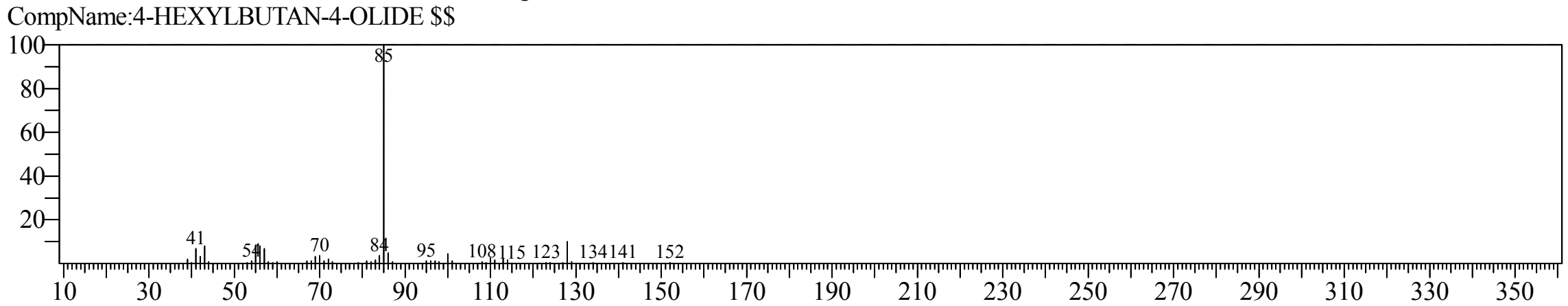

Hit#:10 Entry:8451 Library:NIST05s.LIB
SI:56 Formula:C9H14N2 CAS:13925-06-9 MolWeight:150 RetIndex:1129
CompName:2-Isobutyl-3-methylpyrazine $$ Pyrazine, 2-methyl-3-(2-methylpropyl)- $$ 2-Methyl-3-(2-methylpropyl)pyrazine $$

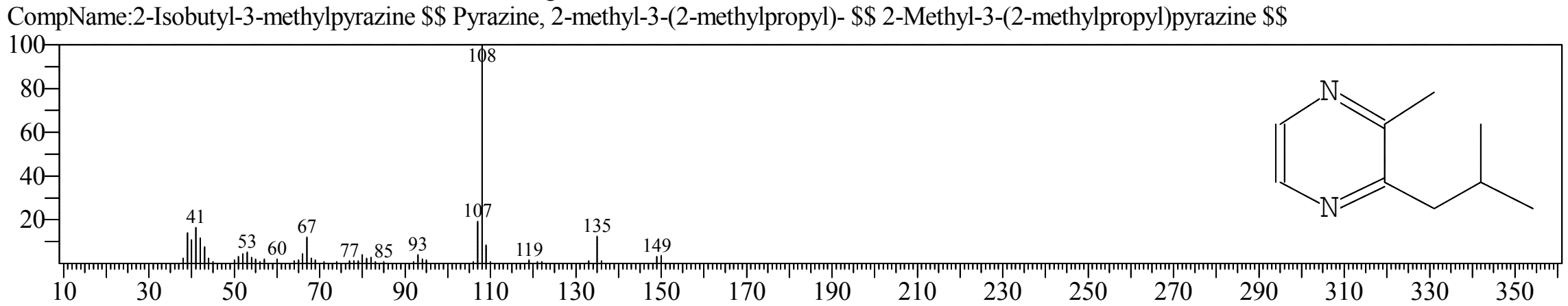

<< Target >>
Line#:16 R.Time:14.833(Scan#:1181) MassPeaks:36
RawMode:Averaged 14.825-14.842(1180-1182) BasePeak:85.15(700)
BG Mode:Calc. from Peak Group 1 - Event 1 Scan

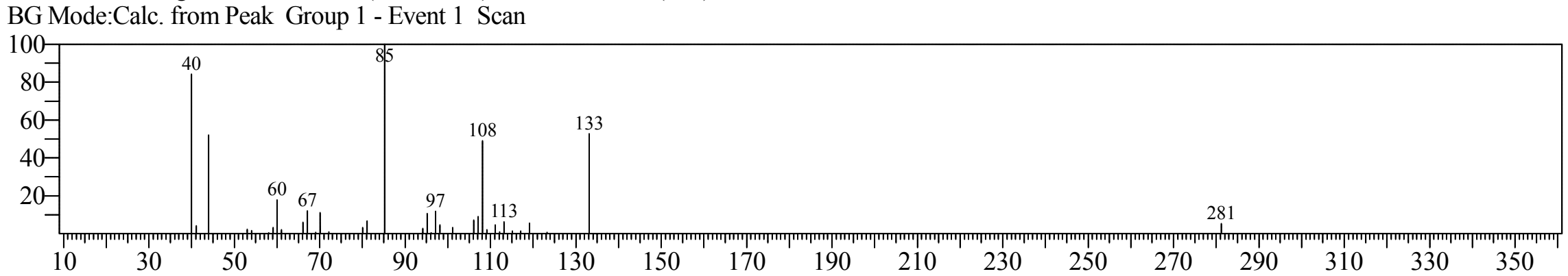

Hit#:11 Entry:789 Library:NIST05s.LIB
SI:56 Formula:C4H7NO CAS:79-39-0 MolWeight:85 RetIndex:795
CompName:Methacrylamide $$ 2-Propenamide, 2-methyl- $$ .alpha.-Methyl acrylic amide $$ Methacryamide $$ Methacrylic acid amide $$ Methacrylic an

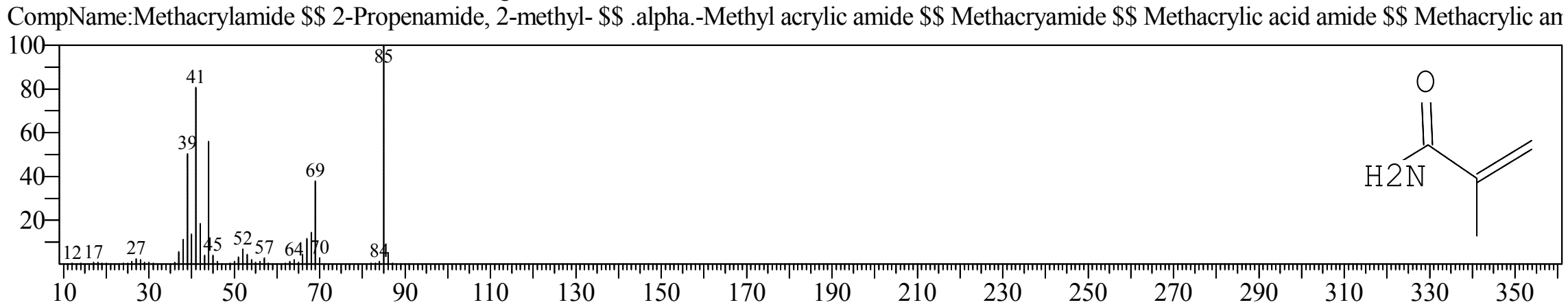

Hit#:12 Entry:58406 Library:WILEY7.LIB
SI:56 Formula:C10 H15 D O2 CAS:617-86-7 MolWeight:168 RetIndex:0
CompName:Silane, triethyl- (CAS) 1,1-DIETHYL-1-SILA-PROPANE $$ Triethylsilane $$ (C2H5)3SiH $$ Triethylsilicon hydride $$

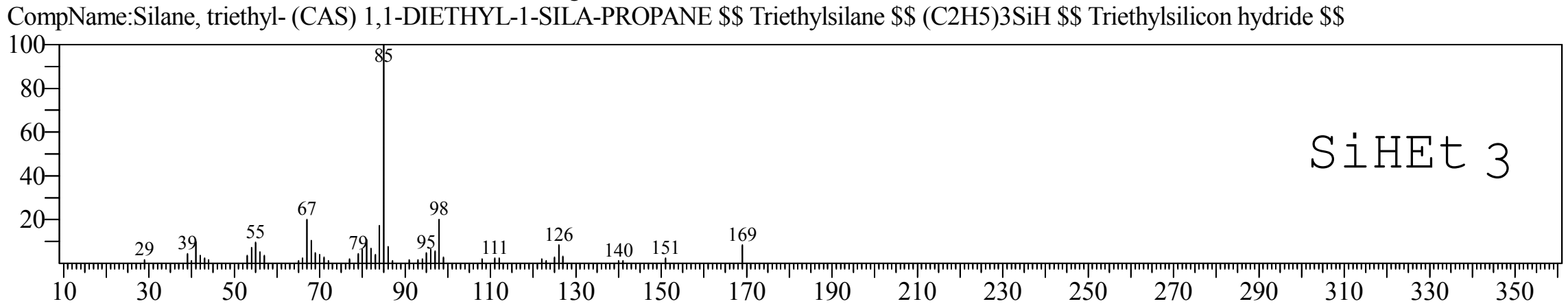

Hit#:13 Entry:108863 Library:WILEY7.LIB
SI:56 Formula:C13 H22 O2 CAS:0-00-0 MolWeight:210 RetIndex:0
CompName:2-exo-methyl-2-endo-(1-oxacyclohex-2-yloxy)bicyclo[2.2.1]heptane $$

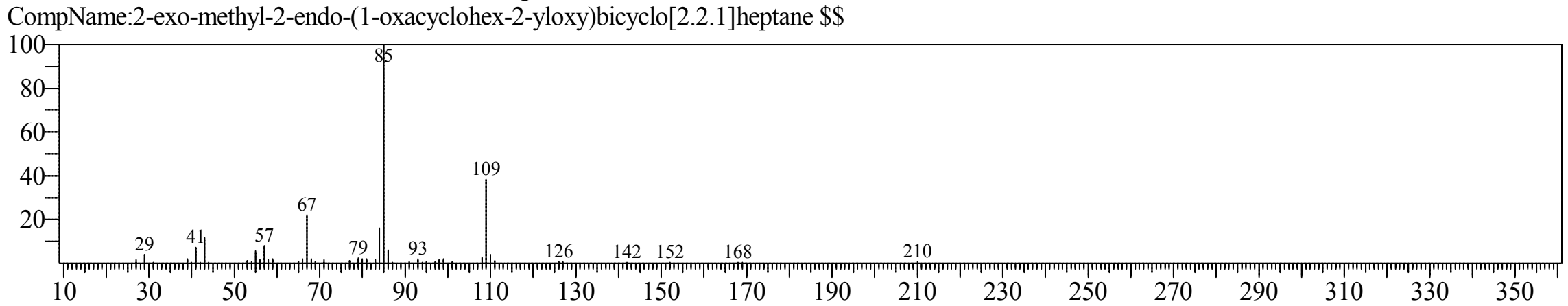

Hit#:14 Entry:17015 Library:NIST05.LIB
SI:55 Formula:C10H18O CAS:27644-04-8 MolWeight:154 RetIndex:1068
CompName:1,5-Heptadien-4-ol, 3,3,6-trimethyl- $$ 3,3,6-Trimethyl-1,5-heptadien-4-ol # $$

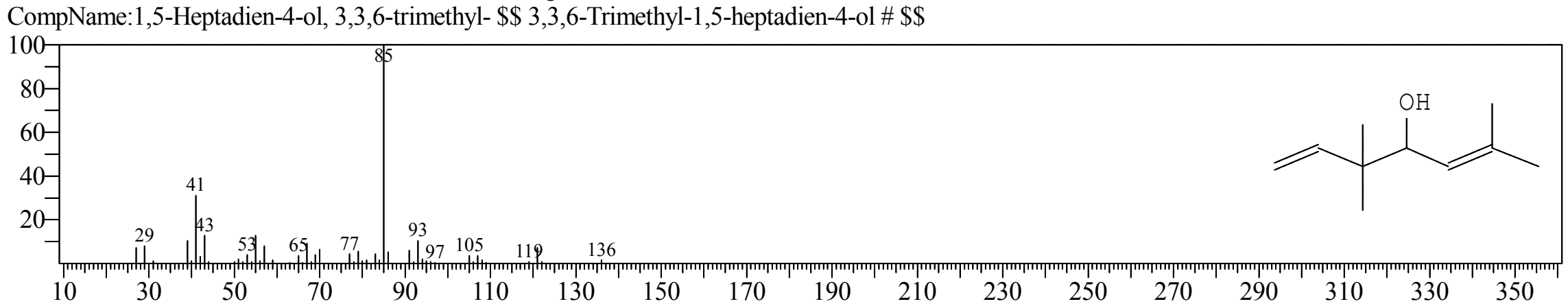

Hit#:15 Entry:108864 Library:WILEY7.LIB
SI:55 Formula:C13 H22 O2 CAS:0-00-0 MolWeight:210 RetIndex:0
CompName:2-endo-methyl-2-exo-(1-oxacyclohex-2-yloxy)bicyclo[2.2.1]heptane $$

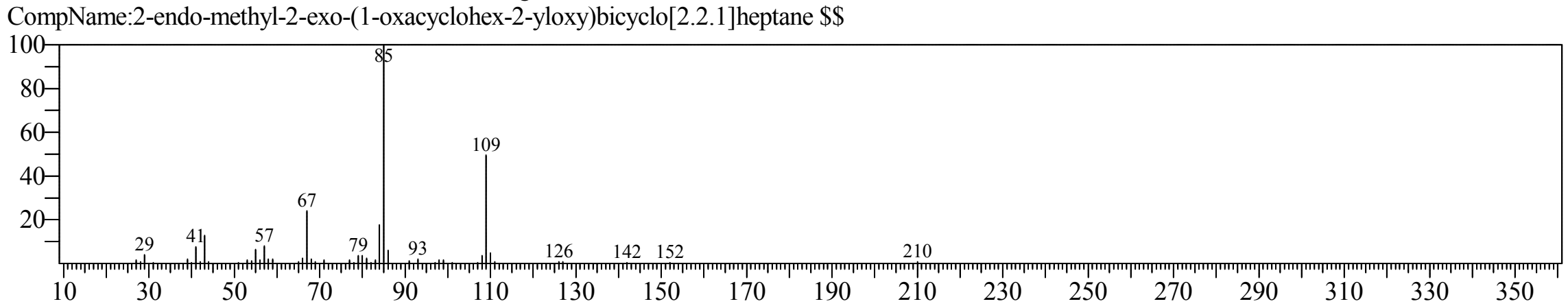

<< Target >>
Line#:16 R.Time:14.833(Scan#:1181) MassPeaks:36
RawMode:Averaged 14.825-14.842(1180-1182) BasePeak:85.15(700)
BG Mode:Calc. from Peak Group 1 - Event 1 Scan

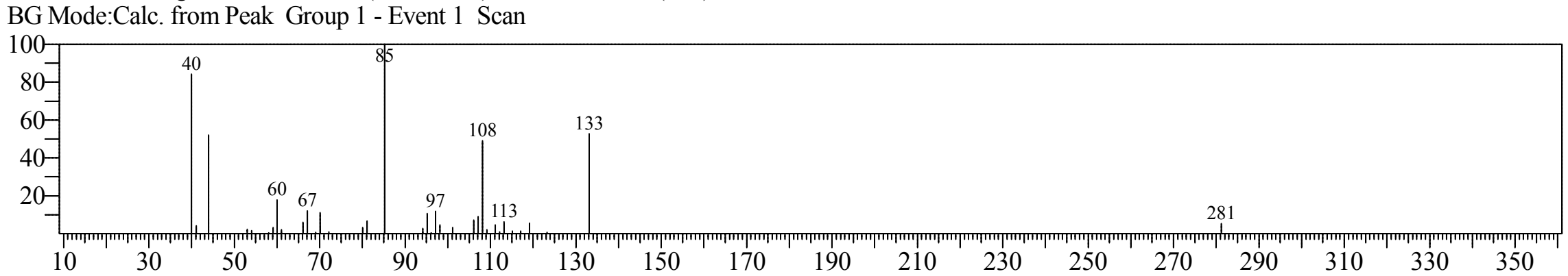

Hit#:16 Entry:131058 Library:NIST05.LIB
SI:55 Formula:C24H41NO CAS:55400-12-9 MolWeight:359 RetIndex:2599
CompName:Pregnan-18-ol, 20-[(1-methylethylidene)amino]- $$ 20-[(1-Methylethylidene)amino]pregnan-18-ol # $$

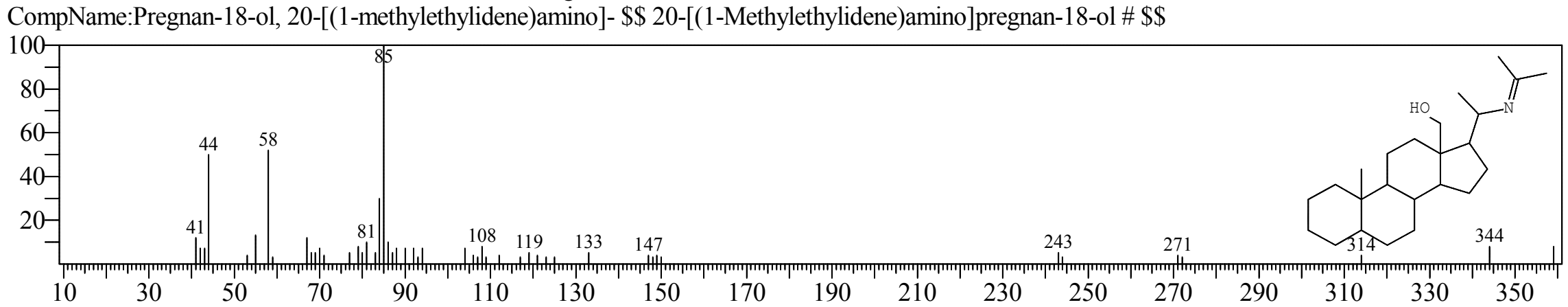

Hit#:17 Entry:73565 Library:NIST05.LIB
SI:55 Formula:C15H26O3 CAS:38290-53-8 MolWeight:254 RetIndex:1953
CompName:2,6-Dimethyl-8-(tetrahydropyran-2-yloxy)-octa-2,6-dien-1-ol $$ (2E,6E)-2,6-Dimethyl-8-(tetrahydro-2H-pyran-2-yloxy)-2,6-octadien-1-ol # $$

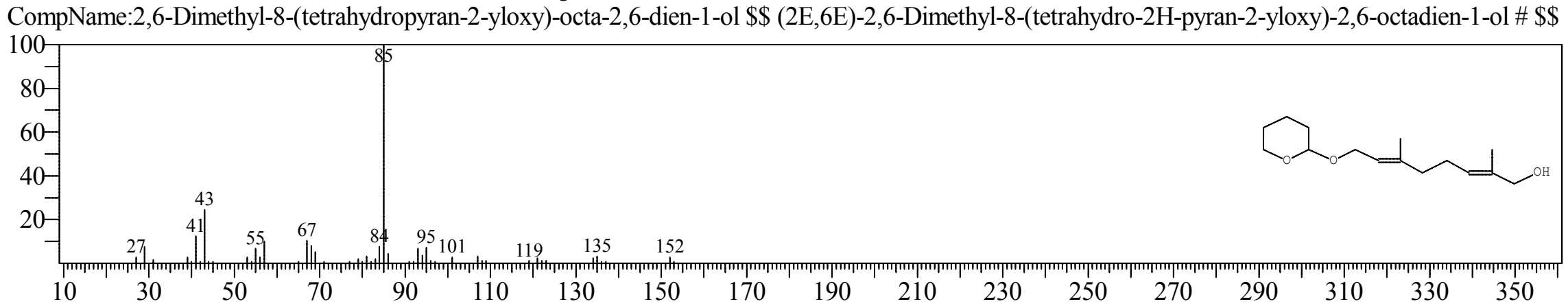

Hit#:18 Entry:3006 Library:WILEY7.LIB
SI:55 Formula:C4 H7 N O CAS:0-00-0 MolWeight:85 RetIndex:0
CompName:3-Methyl-2-propenamide $$

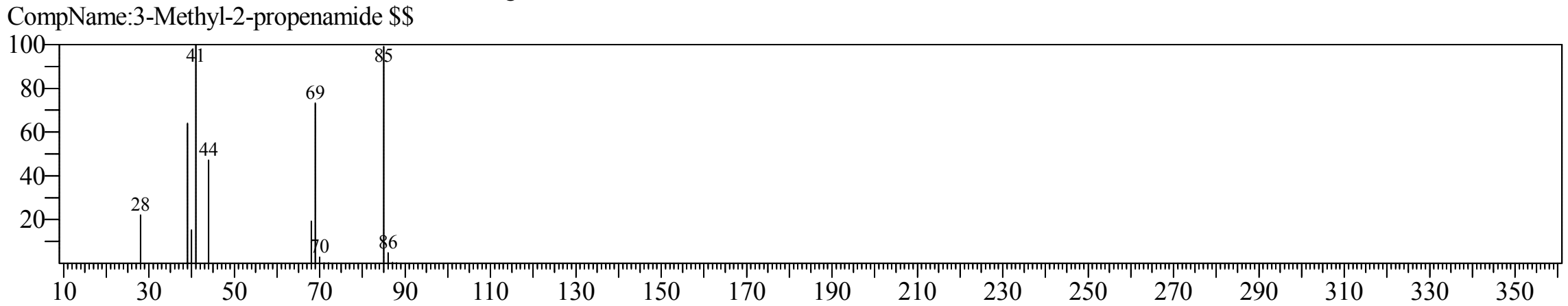

Hit#:19 Entry:91109 Library:WILEY7.LIB
SI:54 Formula:C12 H20 O2 CAS:0-00-0 MolWeight:196 RetIndex:0
CompName:2-exo-(1-oxacyclohex-1-yloxy)bicyclo[2.2.1]heptane $$

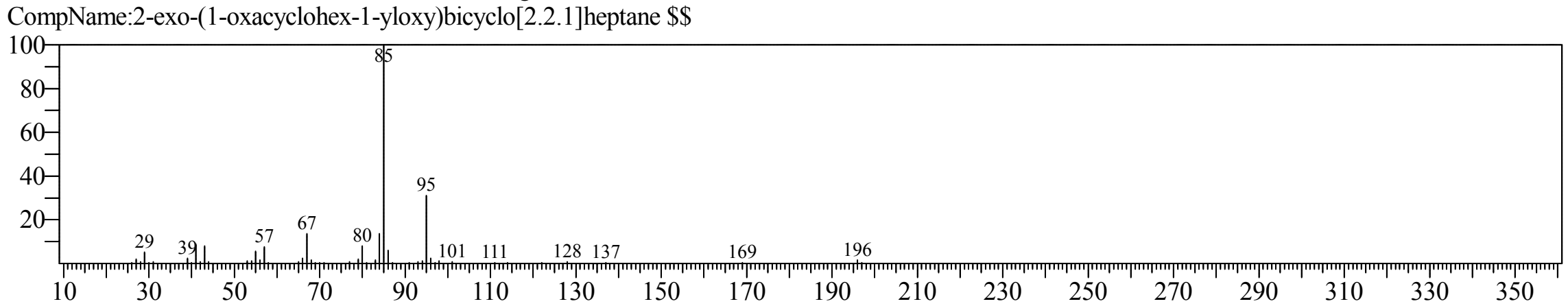

Hit#:20 Entry:16823 Library:WILEY7.LIB
SI:54 Formula:C6 H9 N3 CAS:5621-02-3 MolWeight:123 RetIndex:0
CompName:2-Pyrimidinamine, N,N-dimethyl- $$ Pyrimidine, 2-(dimethylamino)- $$ 2-Dimethylaminopyrimidine $$ 2-Dimethylaminopyrmiidine $$ 2-N,N-

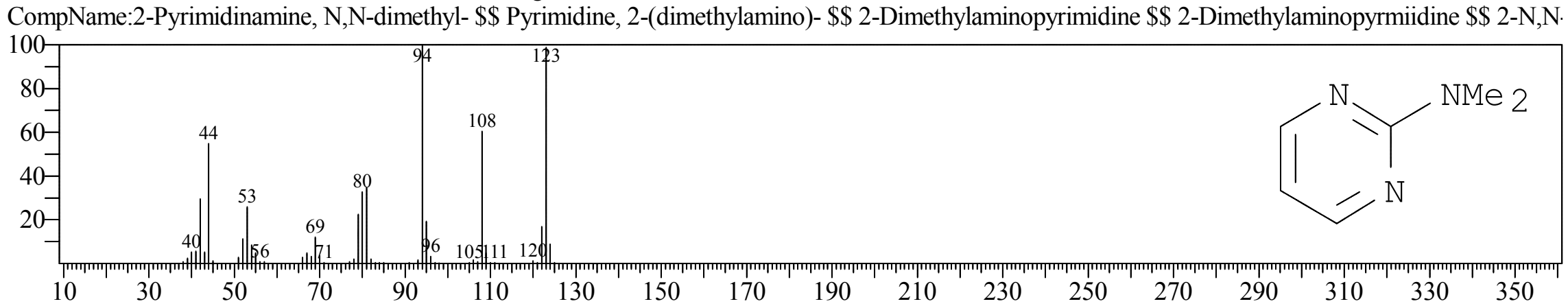

<< Target >>
Line#:17 R.Time:14.867(Scan#:1185) MassPeaks:48
RawMode:Averaged 14.858-14.875(1184-1186) BasePeak:52.00(1051)
BG Mode:Calc. from Peak Group 1 - Event 1 Scan

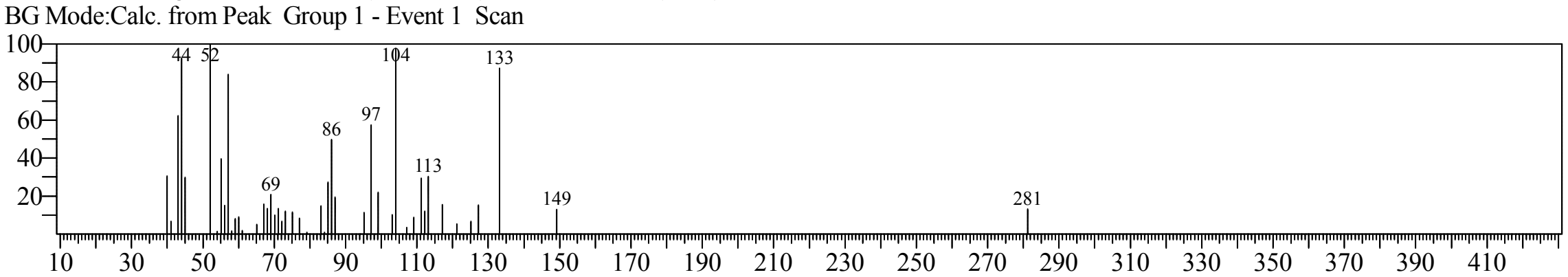

Hit#:1 Entry:129851 Library:NIST05.LIB
SI:65 Formula:C22H44O3 CAS:26158-80-5 MolWeight:356 RetIndex:2618
CompName:Eicosanoic acid, 2-hydroxyethyl ester $$ 2-Hydroxyethyl icosanoate # $$

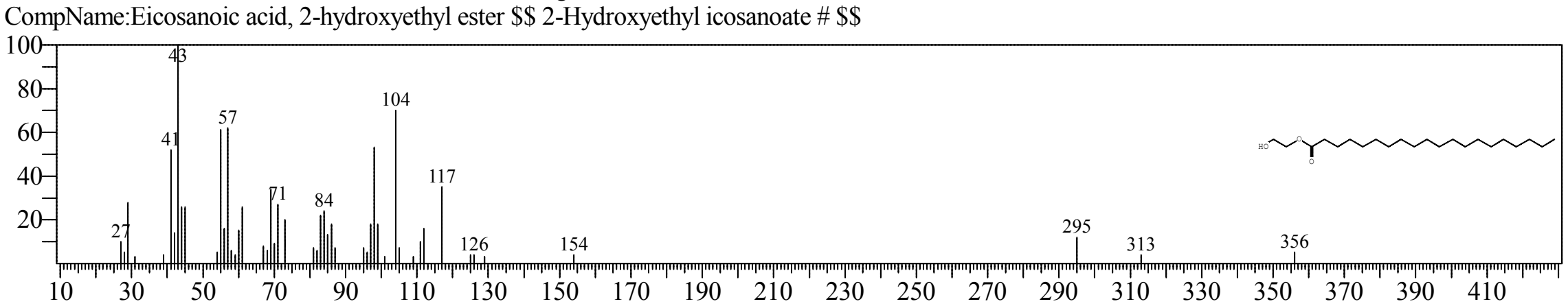

Hit#:2 Entry:116532 Library:NIST05.LIB
SI:64 Formula:C20H40O3 CAS:111-60-4 MolWeight:328 RetIndex:2419
CompName:Octadecanoic acid, 2-hydroxyethyl ester $$ Stearic acid, 2-hydroxyethyl ester $$ Cerasynt M $$ Cerasynt MN $$ Emerest 2350 $$ Empilan 28

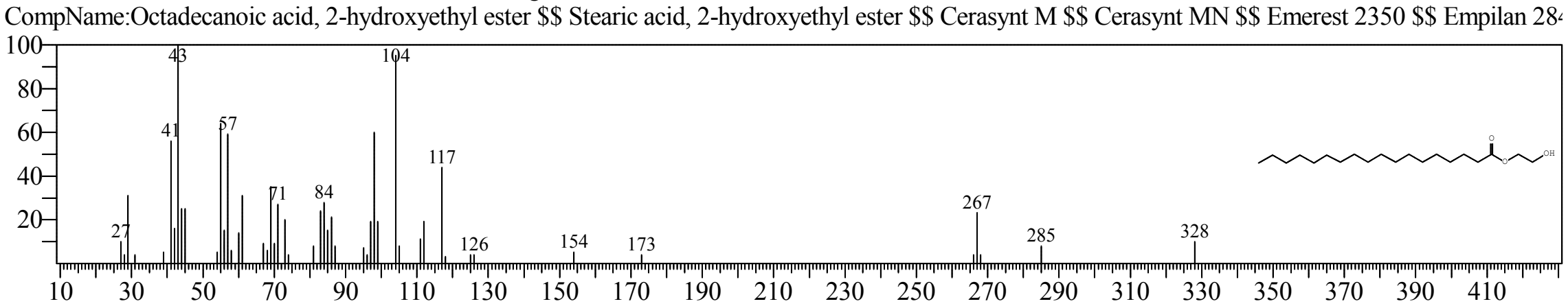

Hit#:3 Entry:84646 Library:NIST05.LIB
SI:64 Formula:C16H32O3 CAS:22122-18-5 MolWeight:272 RetIndex:2022
CompName:Tetradecanoic acid, 2-hydroxyethyl ester $$ Myristic acid, 2-hydroxyethyl ester $$ Ethylene glycol monomyristate $$ Glycol myristate $$ 2-Hy

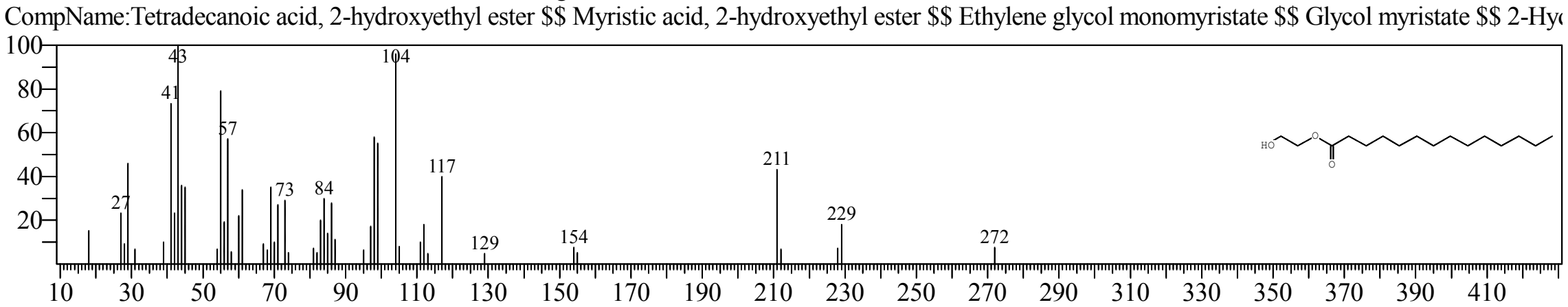

Hit#:4 Entry:135892 Library:NIST05.LIB
SI:64 Formula:C10H11BrF2N2O6 CAS:102302-61-4 MolWeight:372 RetIndex:2485
CompName:1-(.beta.-d-Ribofuranosyl)-4-difluoromethyl-5-bromouracil

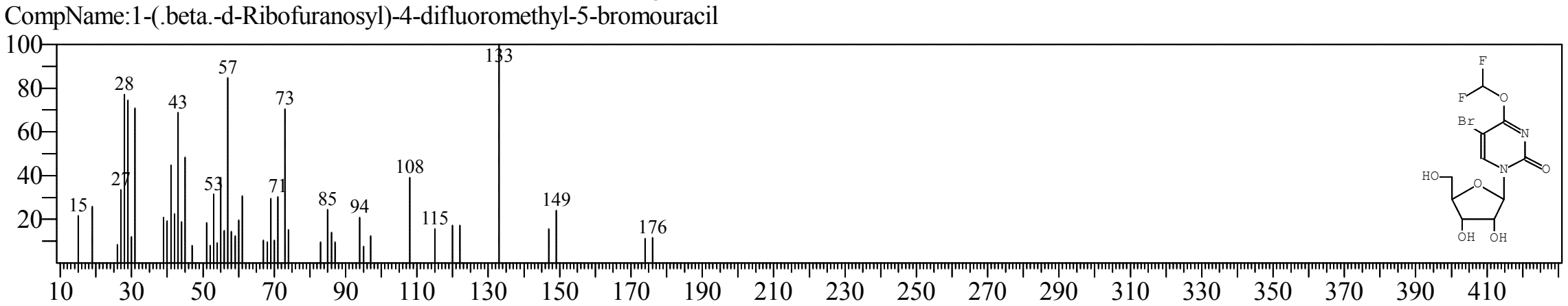

Hit#:5 Entry:211456 Library:WILEY7.LIB
SI:63 Formula:C18 H36 O3 CAS:4219-49-2 MolWeight:300 RetIndex:0
CompName:Hexadecanoic acid, 2-hydroxyethyl ester (CAS) 1-O-HEXADECANOYL-1,2-ETHANEDIOL $$ Palmitoylglycol $$ Glycol palmitate $$ Ethyl

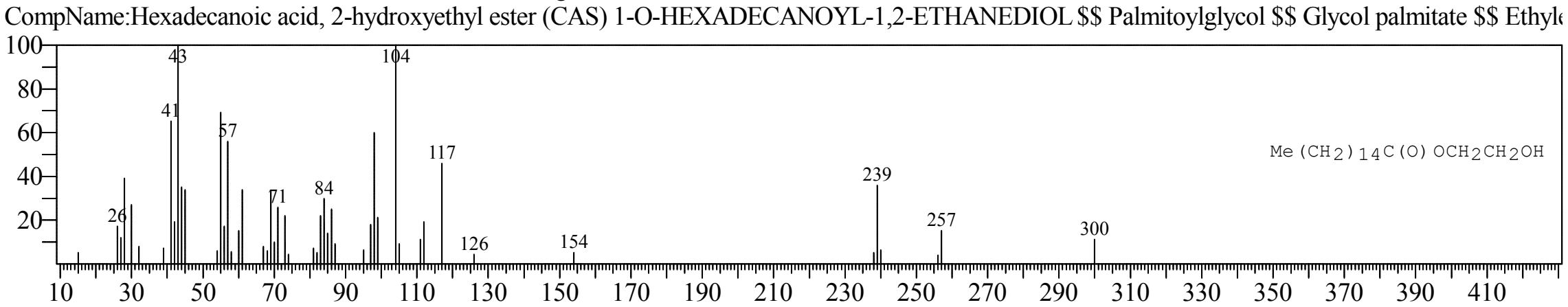

<< Target >>
Line#:17 R.Time:14.867(Scan#:1185) MassPeaks:48
RawMode:Averaged 14.858-14.875(1184-1186) BasePeak:52.00(1051)
BG Mode:Calc. from Peak Group 1 - Event 1 Scan

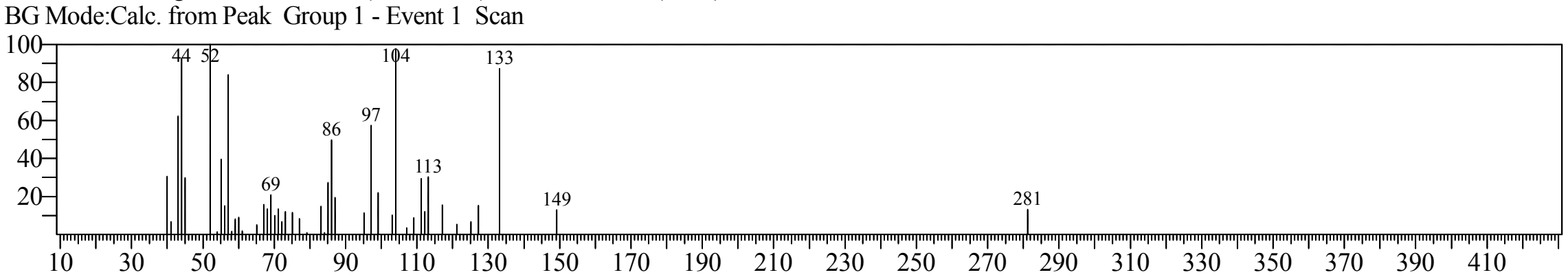

Hit#:6 Entry:122704 Library:NIST05.LIB
SI:63 Formula:C22H44O2 CAS:100508-44-9 MolWeight:340 RetIndex:2119
CompName:6,10,13-Trimethyltetradecyl isovalerate $$ 6,10,13-Trimethyltetradecyl 3-methylbutanoate # $$

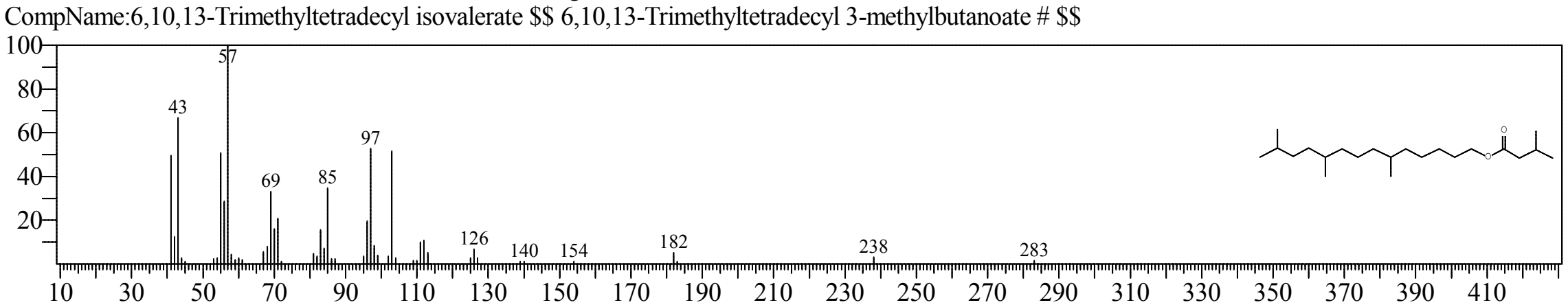

Hit#:7 Entry:294221 Library:WILEY7.LIB
SI:63 Formula:C20 H38 O9 CAS:0-00-0 MolWeight:422 RetIndex:0
CompName:BETA-D-LYXOFURANOSID, 5-O-(BETA-D-LYXOFURANOSYL)-DECANYL- $$

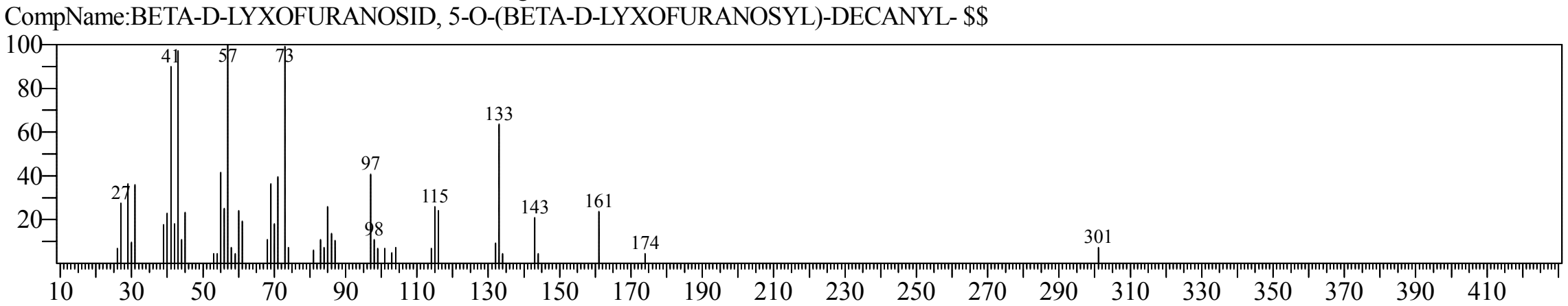

Hit#:8 Entry:7654 Library:NIST05.LIB
SI:63 Formula:C6H10O3 CAS:14059-74-6 MolWeight:130 RetIndex:1028
CompName:1,6-Anhydro-2,4-dideoxy-.beta.-D-ribo-hexopyranose

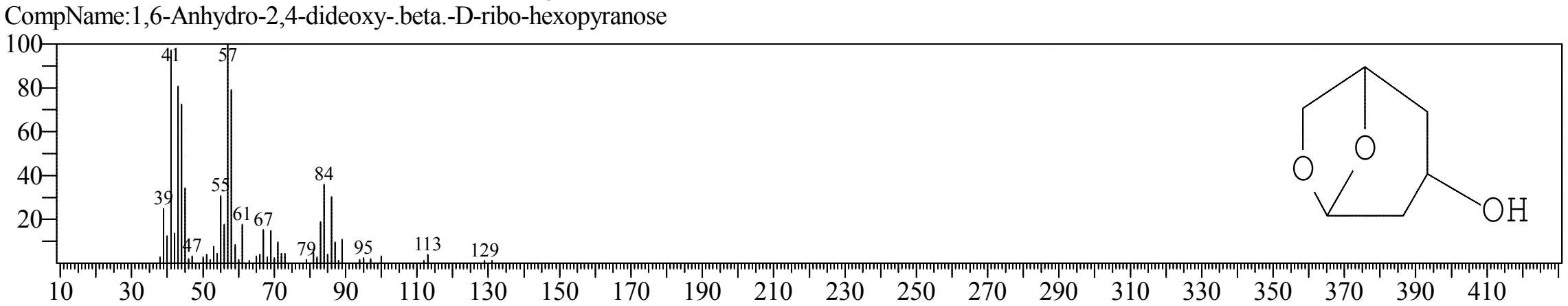

Hit#:9 Entry:148969 Library:NIST05.LIB
SI:63 Formula:C20H38O9 CAS:0-00-0 MolWeight:422 RetIndex:3342
CompName:.beta.-D-Lyxofuranoside, 5-O-(.beta.-D-lyxofuranosyl)-decyl-

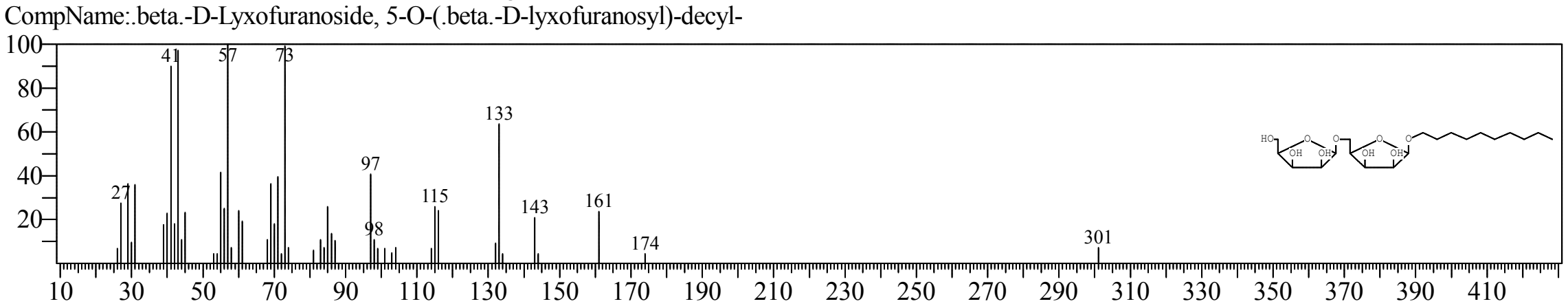

Hit#:10 Entry:189612 Library:NIST11.lib
SI:63 Formula:C20H38O9 CAS:0-00-0 MolWeight:422 RetIndex:3342
CompName:.beta.-D-Lyxofuranoside, 5-O-(.beta.-D-lyxofuranosyl)-decyl- $$ Decyl 5-O-pentofuranosylpentofuranoside # $$

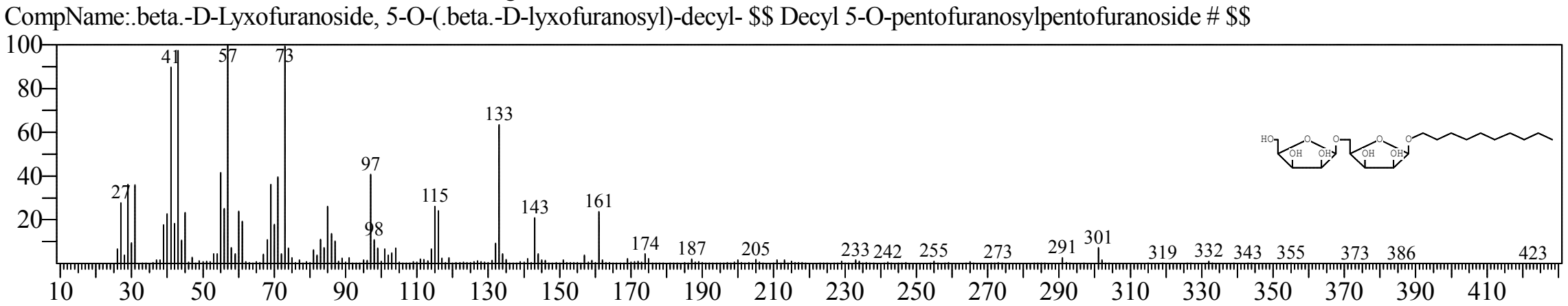

<< Target >>
Line#:17 R.Time:14.867(Scan#:1185) MassPeaks:48
RawMode:Averaged 14.858-14.875(1184-1186) BasePeak:52.00(1051)
BG Mode:Calc. from Peak Group 1 - Event 1 Scan

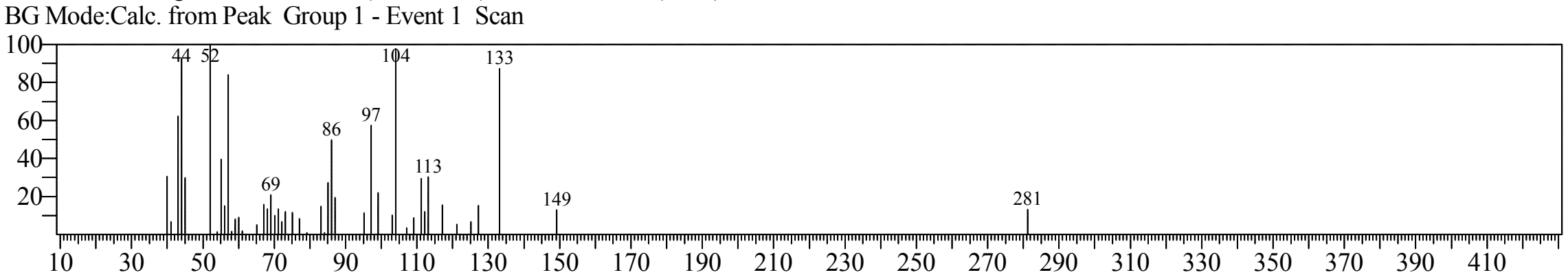

Hit#:11 Entry:21067 Library:NIST05.LIB
SI:63 Formula:C6H12O5 CAS:27299-12-3 MolWeight:164 RetIndex:1530
CompName:D-Glucitol, 1,4-anhydro- $$ 1,4-Anhydrohexitol # $$

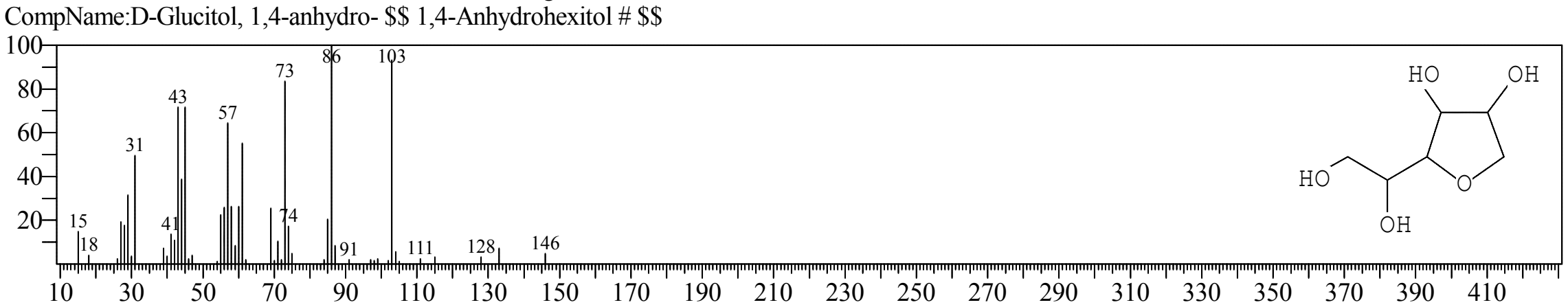

Hit#:12 Entry:11937 Library:NIST05.LIB
SI:63 Formula:C9H18O CAS:56740-09-1 MolWeight:142 RetIndex:968
CompName:trans-4,5-Epoxynonane $$ 2-Butyl-3-propyloxirane # $$

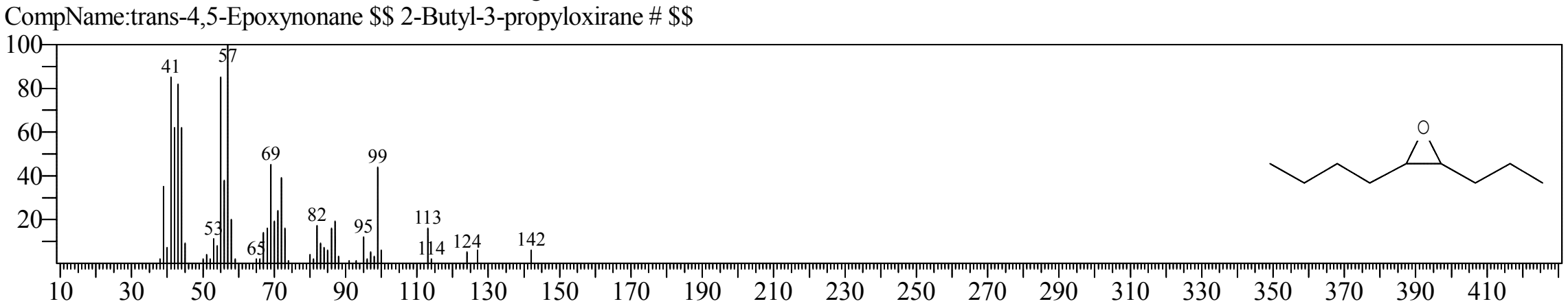

Hit#:13 Entry:21066 Library:NIST05.LIB
SI:62 Formula:C6H12O5 CAS:7726-97-8 MolWeight:164 RetIndex:1530
CompName:d-Mannitol, 1,4-anhydro- $$ 1,4-Anhydro-d-mannitol $$ 1,4-Anhydrohexitol # $$

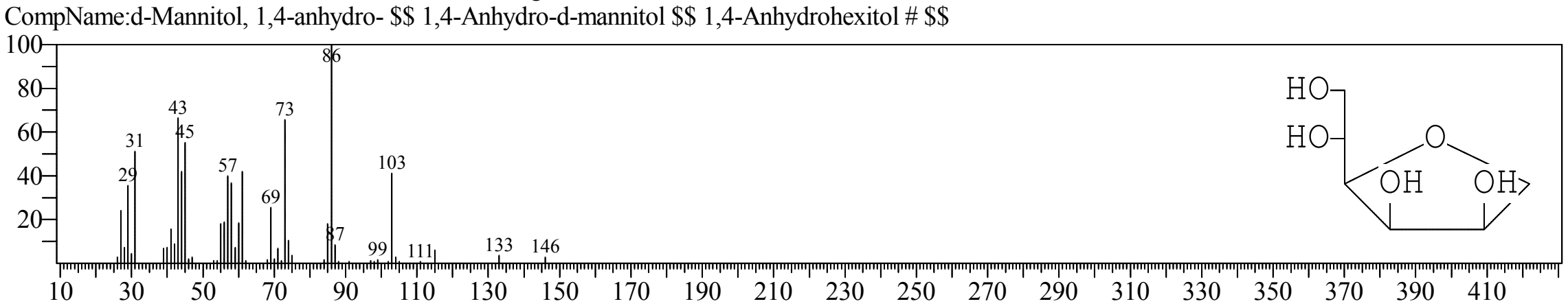

Hit#:14 Entry:19313 Library:NIST05.LIB
SI:62 Formula:C7H12O4 CAS:16503-06-3 MolWeight:160 RetIndex:1221
CompName:Pentanoic acid, 2-(methoxymethyl)-4-oxo- $$ 2-(Methoxymethyl)-4-oxopentanoic acid # $$

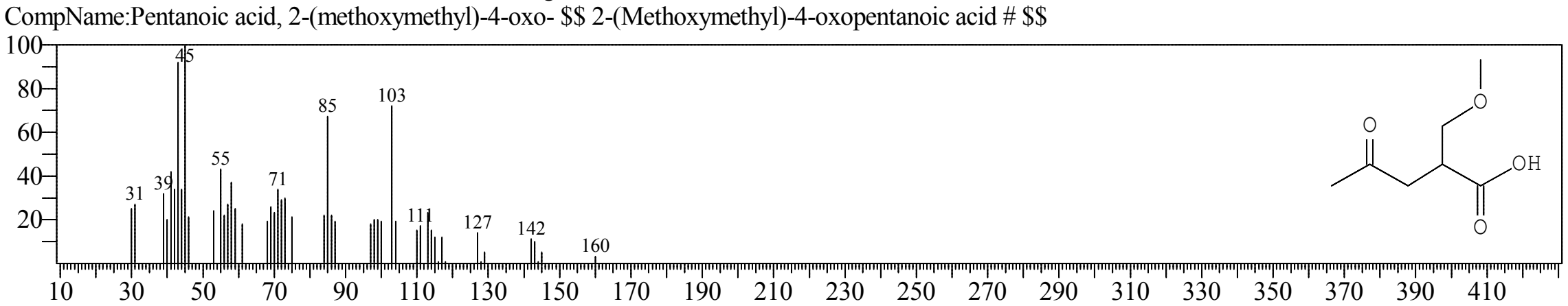

Hit#:15 Entry:64698 Library:NIST05.LIB
SI:62 Formula:C8H16O8 CAS:0-00-0 MolWeight:240 RetIndex:2221
CompName:l-Gala-l-ido-octose

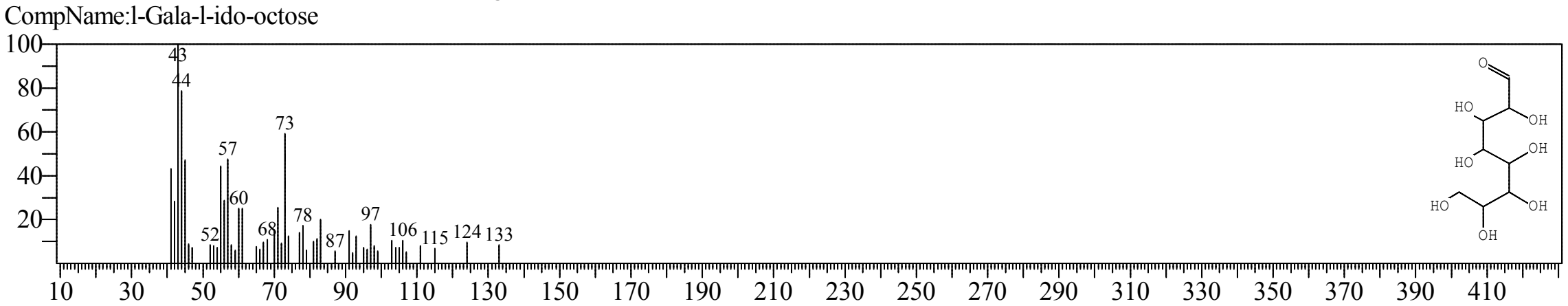

<< Target >>
Line#:17 R.Time:14.867(Scan#:1185) MassPeaks:48
RawMode:Averaged 14.858-14.875(1184-1186) BasePeak:52.00(1051)
BG Mode:Calc. from Peak Group 1 - Event 1 Scan

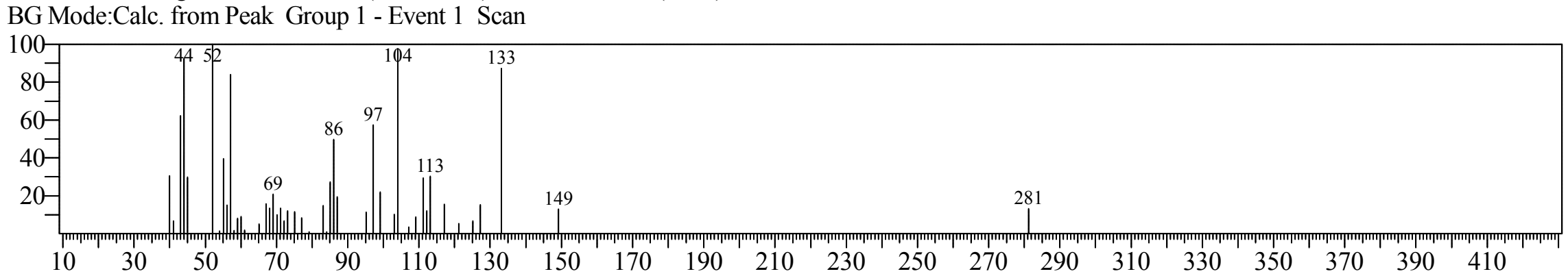

Hit#:16 Entry:71579 Library:NIST11.lib
SI:62 Formula:C8H16O8 CAS:0-00-0 MolWeight:240 RetIndex:2221
CompName:l-Gala-l-ido-octose $$ Octose # $$

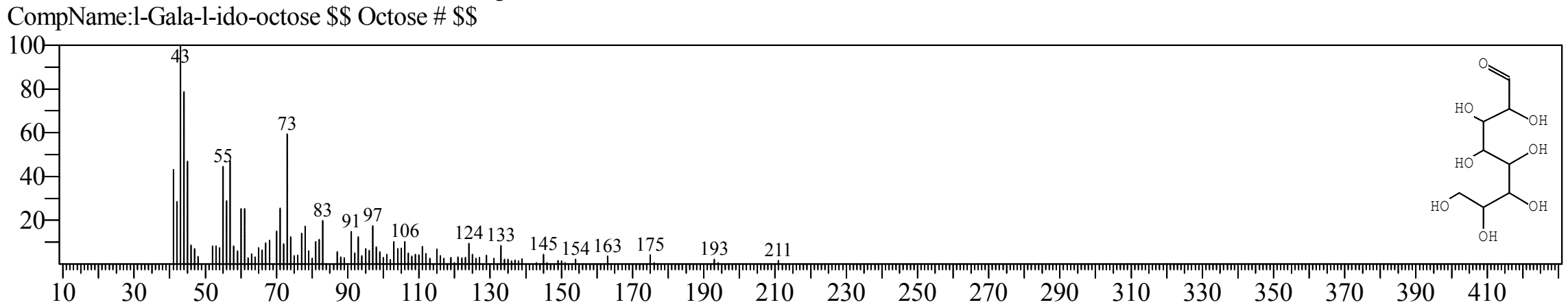

Hit#:17 Entry:21204 Library:WILEY7.LIB
SI:62 Formula:C6 H10 O3 CAS:0-00-0 MolWeight:130 RetIndex:0
CompName:6,8-DIOXABICYCLO(3.2.1)OCTAN-3L-OL $$

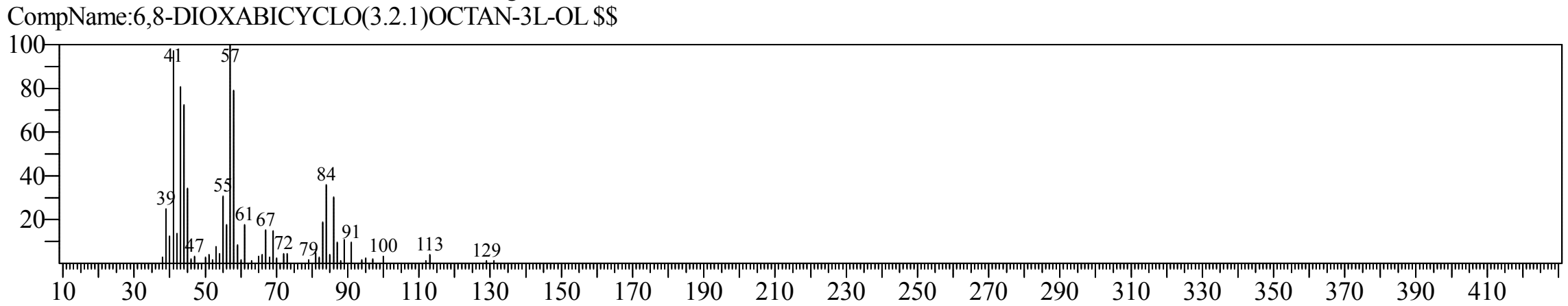

Hit#:18 Entry:161641 Library:NIST05.LIB
SI:62 Formula:C32H34N2O12 CAS:88254-07-3 MolWeight:638 RetIndex:5667
CompName:5,12-Naphthacenedione, 10-((3-(3-cyano-4-morpholinyl)-2,3,6-trideoxy-.alpha.-L-lyxo-hexopyranosyl)oxy)-7,8,9,10-tetrahydro-6,8,11-trihydro:

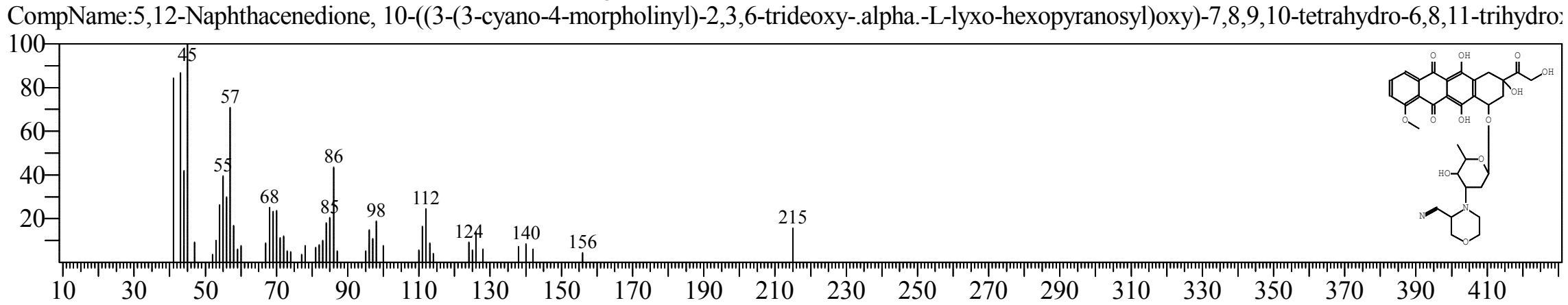

Hit#:19 Entry:17988 Library:NIST05.LIB
SI:62 Formula:C10H20O CAS:2165-61-9 MolWeight:156 RetIndex:1067
CompName:trans-5,6-Epoxydecane $$ 2,3-Dibutyloxirane # $$

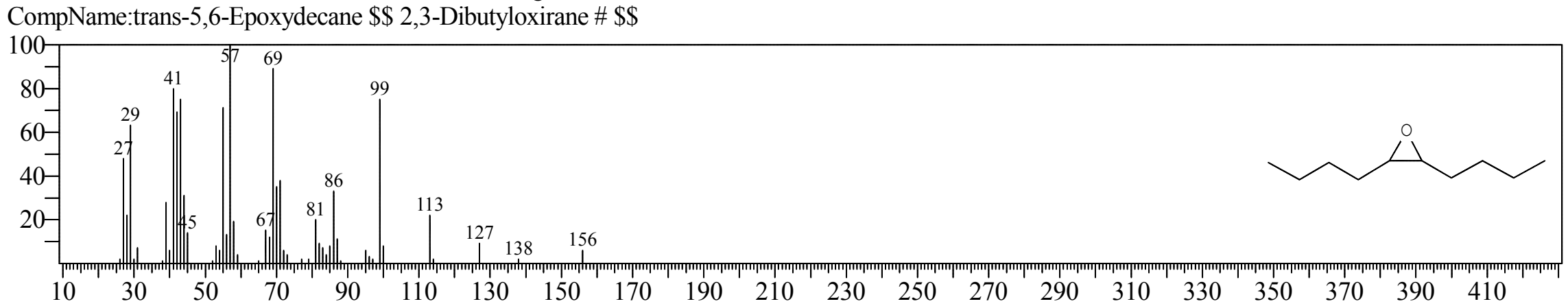

Hit#:20 Entry:21041 Library:NIST05.LIB
SI:62 Formula:C6H12O5 CAS:0-00-0 MolWeight:164 RetIndex:1577
CompName:3-Deoxyglucose

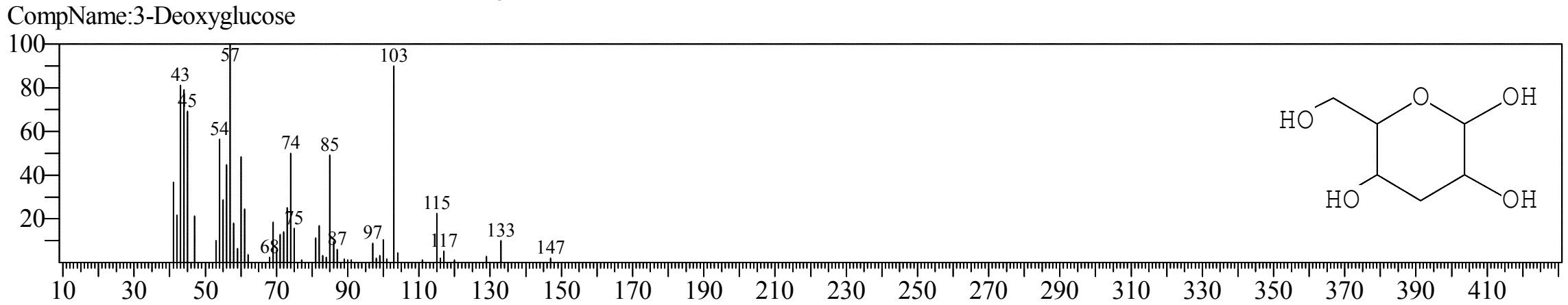

<< Target >>
Line#:18   R.Time:15.025(Scan#:1204) MassPeaks:52
RawMode:Averaged 15.017-15.033(1203-1205) BasePeak:44.00(1533)
BG Mode:Calc. from Peak Group 1 - Event 1 Scan

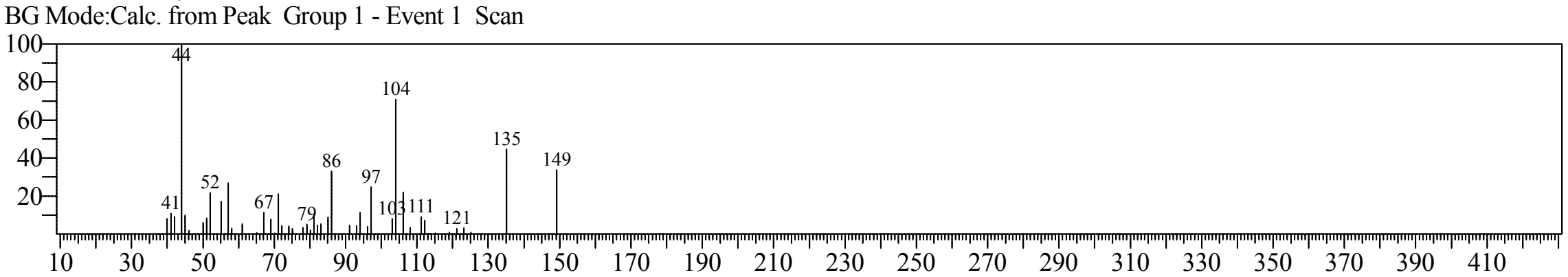

Hit#:1 Entry:21170 Library:NIST05.LIB
SI:64 Formula:C8H8N2O2 CAS:74231-55-3 MolWeight:164 RetIndex:1369
CompName:3-Pyridinecarboxaldehyde, O-acetyloxime, (E)- $$ Nicotinaldehyde o-acetyloxime # $$

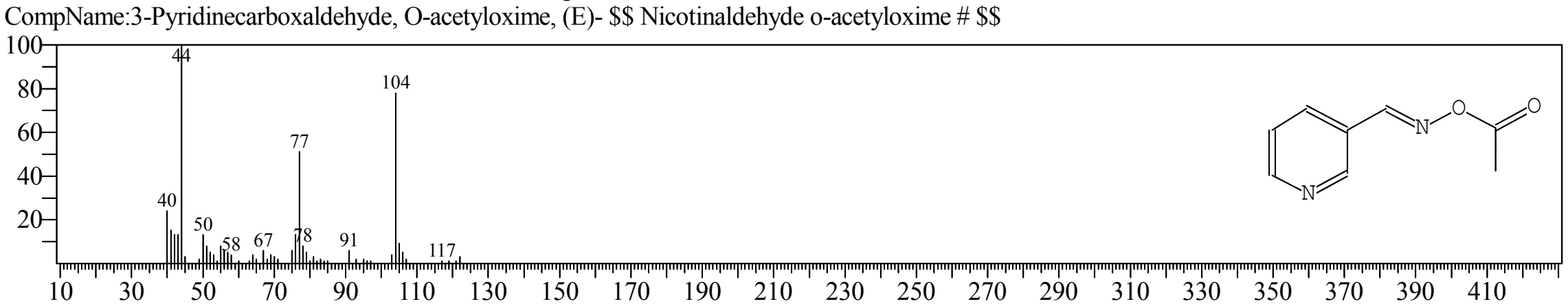

Hit#:2 Entry:27736 Library:NIST11.lib
SI:62 Formula:C7H14N2O3 CAS:116250-49-8 MolWeight:174 RetIndex:1426
CompName:1-Methyl-4-[nitromethyl]-4-piperidinol

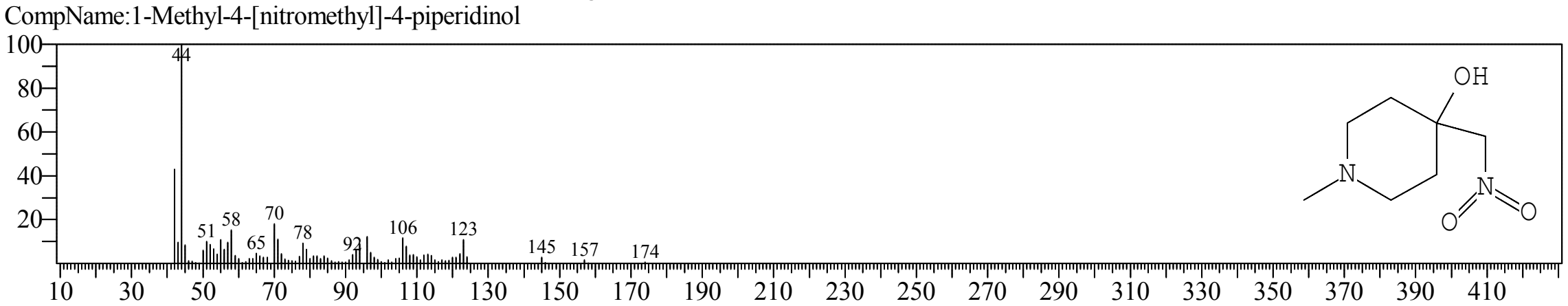

Hit#:3 Entry:37648 Library:NIST05.LIB
SI:62 Formula:C12H18O2 CAS:21898-91-9 MolWeight:194 RetIndex:1553
CompName:Tricyclo[4.3.1.1(3,8)]undecane-3-carboxylic acid $$ 3-Homoadamantanecarboxylic acid $$

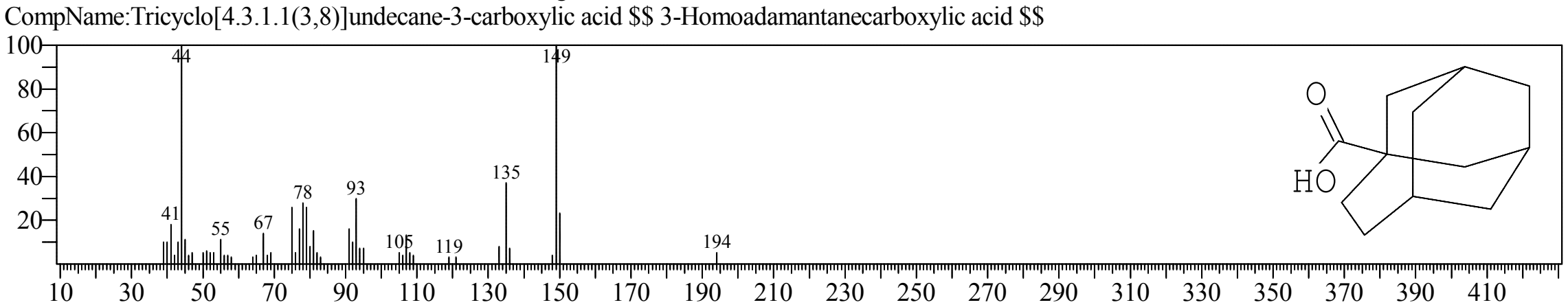

Hit#:4 Entry:6630 Library:WILEY7.LIB
SI:60 Formula:C6 H8 D4 O CAS:53778-63-5 MolWeight:100 RetIndex:0
CompName:.ALPHA.-D4-HEXAMETHYLENE OXIDE $$

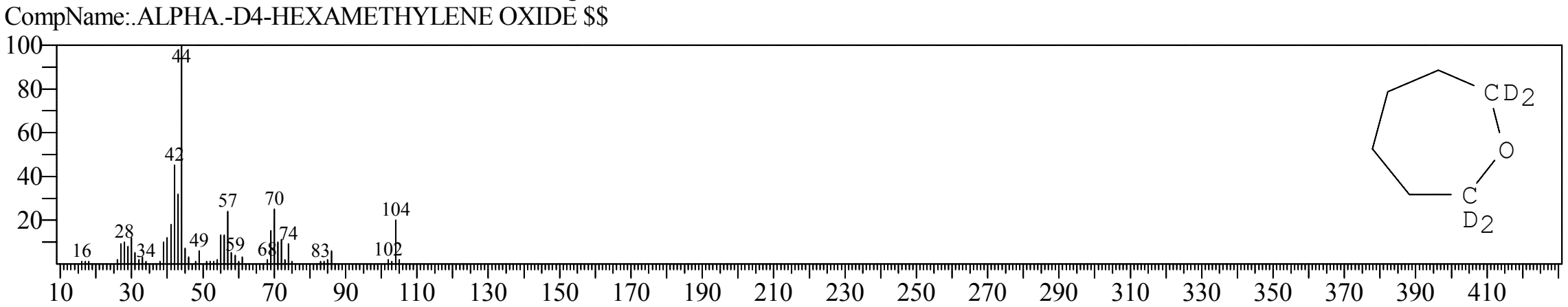

Hit#:5 Entry:200315 Library:WILEY7.LIB
SI:60 Formula:C16 H19 N O4 CAS:519-09-5 MolWeight:289 RetIndex:0
CompName:8-Azabicyclo[3.2.1]octane-2-carboxylic acid, 3-(benzoyloxy)-8-methyl-, [1R-(exo,exo)]- (CAS) Benzoylecgonine $$ Ecgonine benzoate $$ O-B

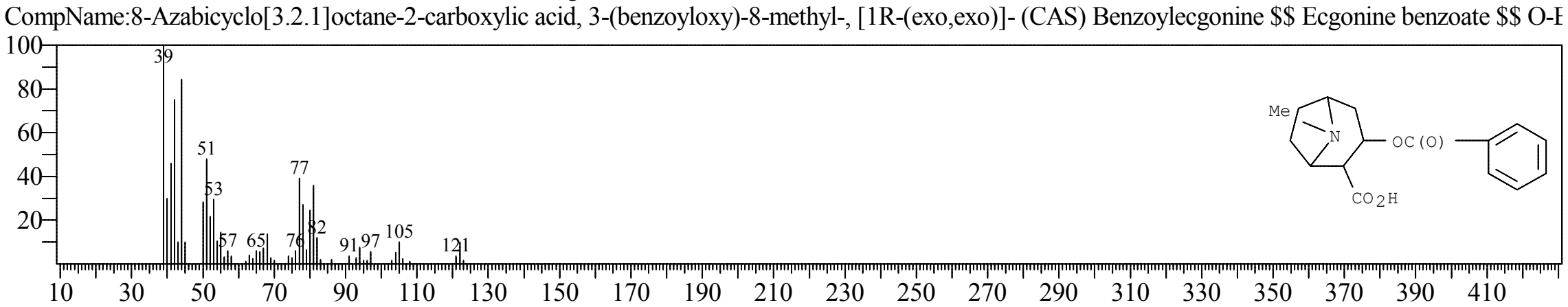

<< Target >>
Line#:18 R.Time:15.025(Scan#:1204) MassPeaks:52
RawMode:Averaged 15.017-15.033(1203-1205) BasePeak:44.00(1533)
BG Mode:Calc. from Peak Group 1 - Event 1 Scan

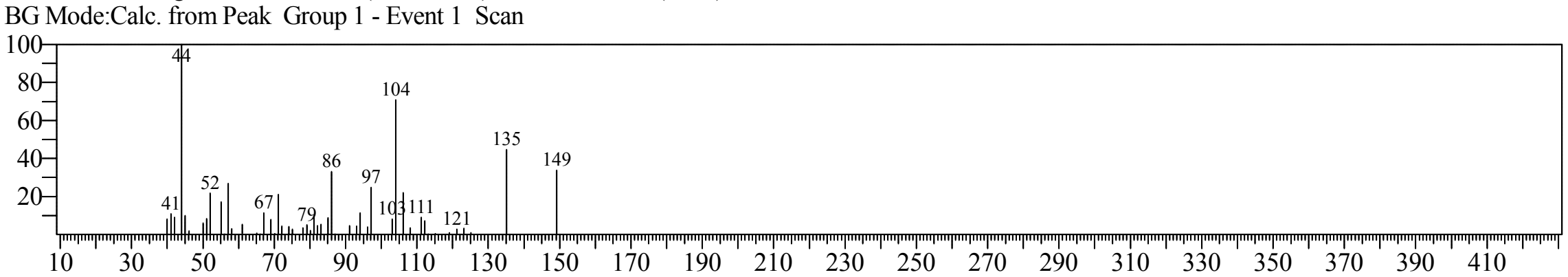

Hit#:6 Entry:42455 Library:NIST05.LIB
SI:60 Formula:C10H9N3O2 CAS:13230-13-2 MolWeight:203 RetIndex:1760
CompName:1-Benzyl-4-nitroimidazole $$ 1-Benzyl-4-nitro-1H-imidazole # $$

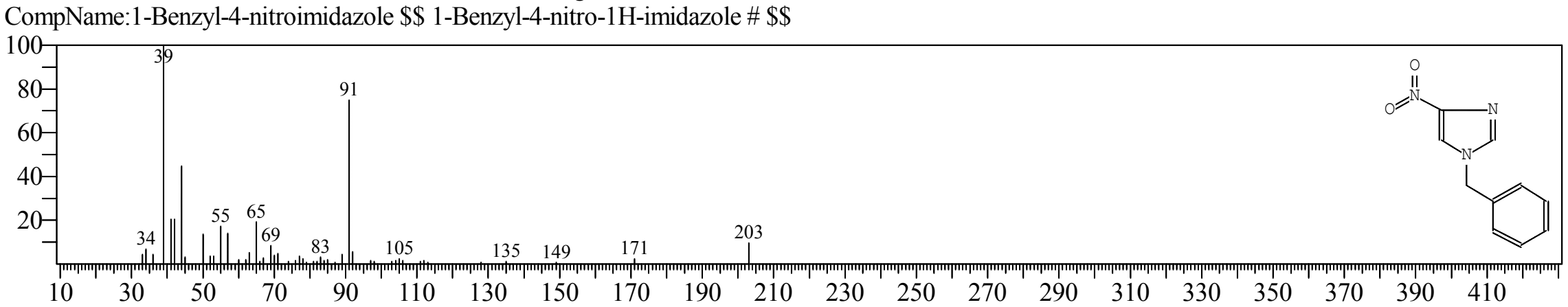

Hit#:7 Entry:48171 Library:NIST11.lib
SI:60 Formula:C7H5N5O3 CAS:948-60-7 MolWeight:207 RetIndex:2253
CompName:Pterin-6-carboxylic acid $$ Pterine-6-carboxylic acid $$ 2-Amino-4-hydroxypteridine-6-carboxylic acid $$ 6-Pteridinecarboxylic acid, 2-amino-

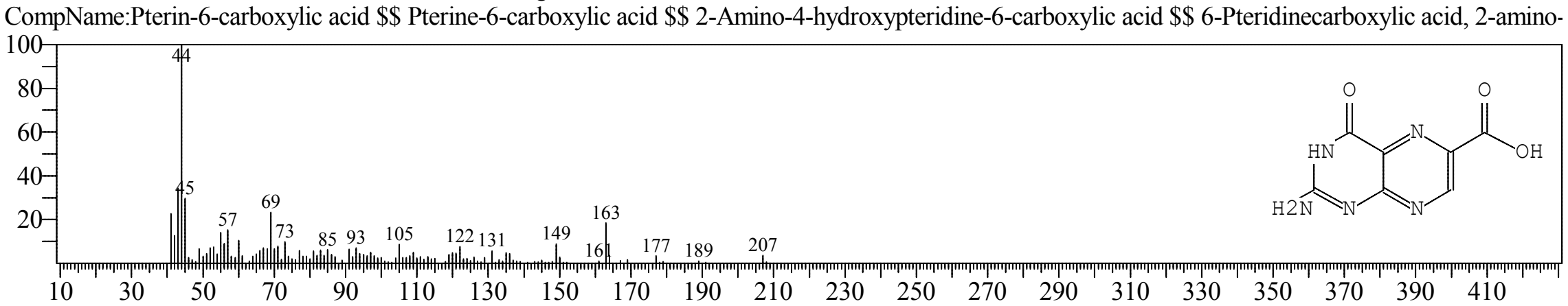

Hit#:8 Entry:37647 Library:NIST05.LIB
SI:59 Formula:C12H18O2 CAS:31061-65-1 MolWeight:194 RetIndex:1553
CompName:Tricyclo[4.3.1.1(3,8)]undecane-1-carboxylic acid $$ 1-Homoadamantanecarboxylic acid $$

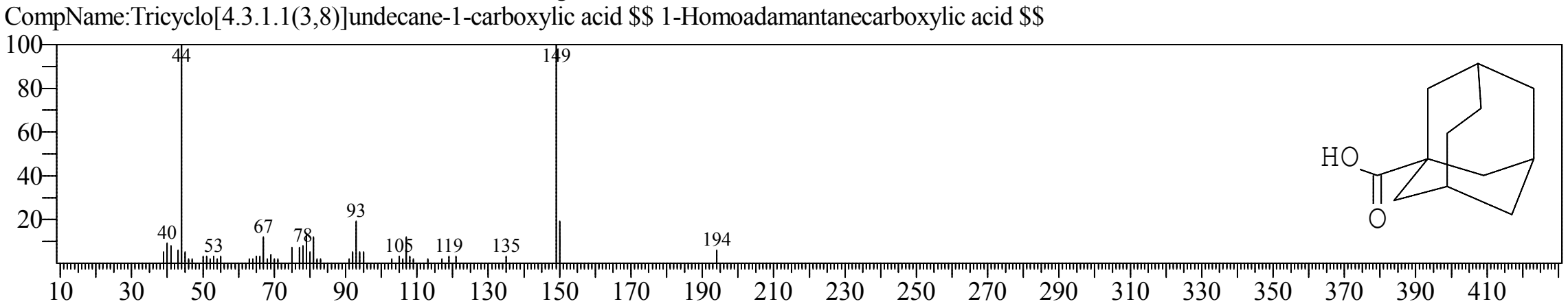

Hit#:9 Entry:150129 Library:NIST05.LIB
SI:58 Formula:C17H21F6N3O3 CAS:0-00-0 MolWeight:429 RetIndex:2129
CompName:4-Aminobutyramide, N-methyl-N-[4-(1-pyrrolidinyl)-2-butynyl]-N',N'-bis(trifluoroacetyl)-

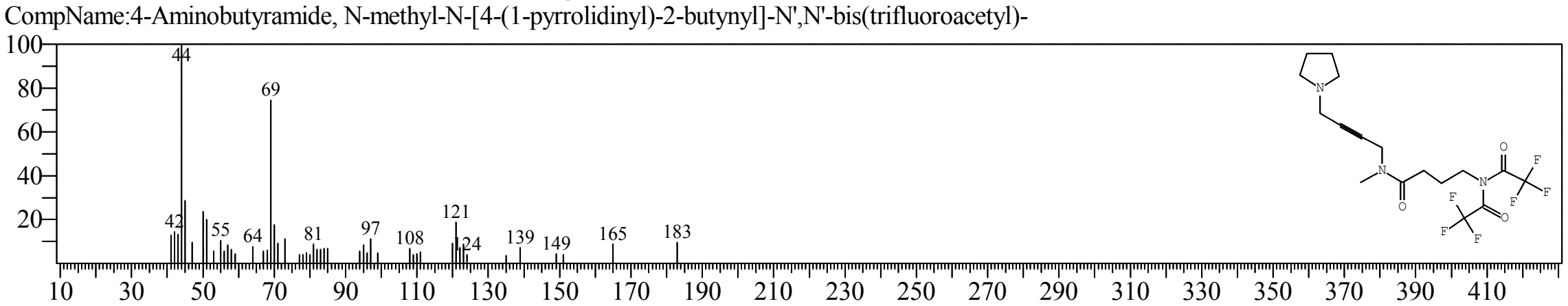

Hit#:10 Entry:81091 Library:WILEY7.LIB
SI:58 Formula:C9 H16 O2 S CAS:55103-51-0 MolWeight:188 RetIndex:0
CompName:1,4-Dioxaspiro[4.5]decane, 8-(methylthio)- (CAS)

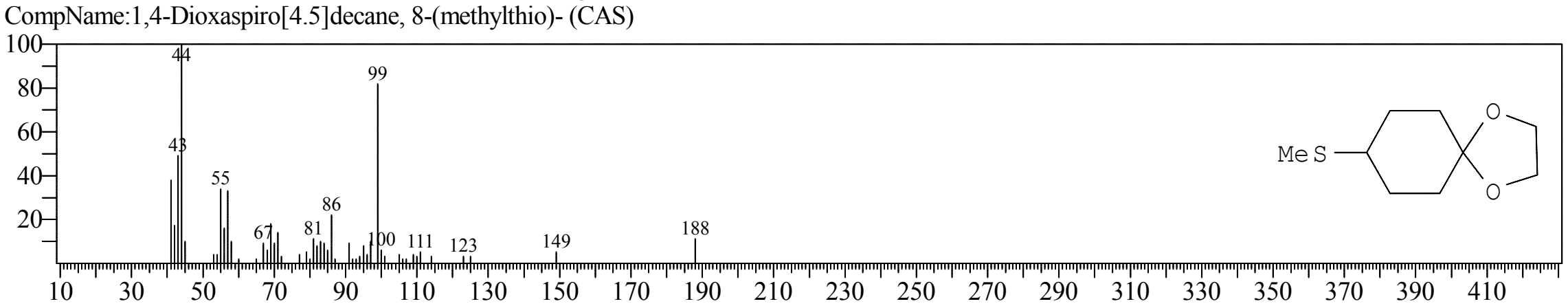

<< Target >>
Line#:18 R.Time:15.025(Scan#:1204) MassPeaks:52
RawMode:Averaged 15.017-15.033(1203-1205) BasePeak:44.00(1533)
BG Mode:Calc. from Peak Group 1 - Event 1 Scan

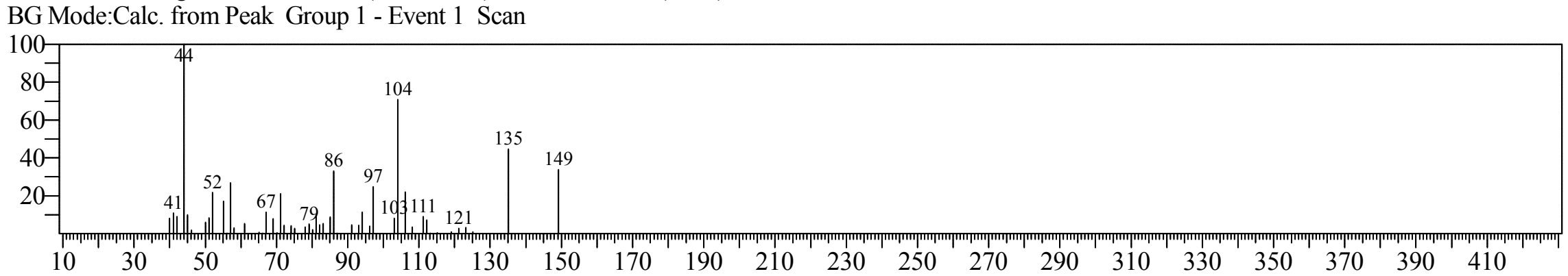

Hit#:11 Entry:191754 Library:NIST11.lib
SI:58 Formula:C17H21F6N3O3 CAS:0-00-0 MolWeight:429 RetIndex:2129
CompName:4-Aminobutyramide, N-methyl-N-[4-(1-pyrrolidinyl)-2-butynyl]-N',N'-bis(trifluoroacetyl)- $$ 4-[Bis(trifluoroacetyl)amino]-N-methyl-N-[4-(1-p

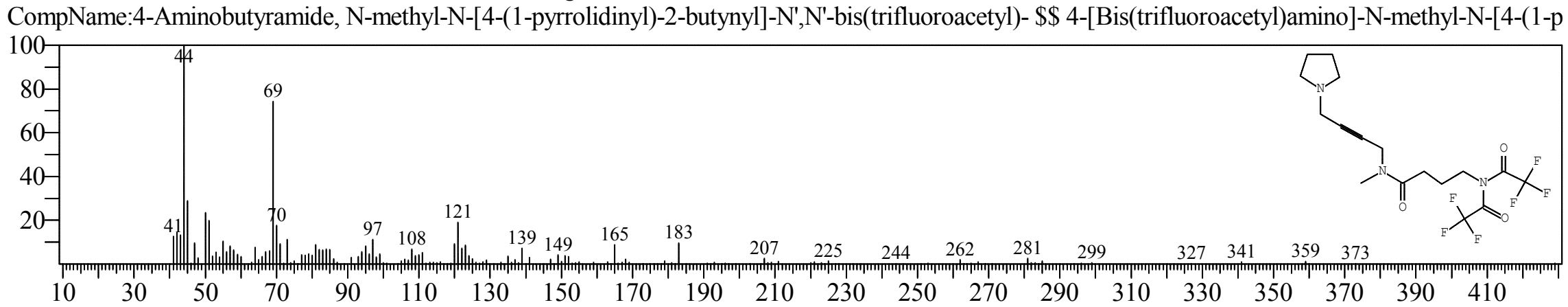

Hit#:12 Entry:9061 Library:NIST05.LIB
SI:58 Formula:C9H13N CAS:0-00-0 MolWeight:135 RetIndex:1171
CompName:4,4-Dimethyl-cyclohex-1-enecarbonitrile

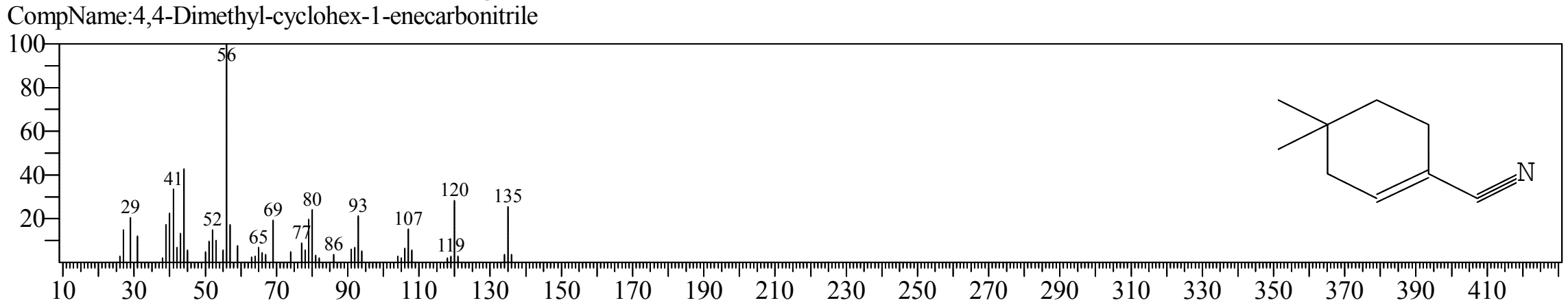

Hit#:13 Entry:24683 Library:WILEY7.LIB
SI:58 Formula:C9 H13 N CAS:0-00-0 MolWeight:135 RetIndex:0
CompName:4,4-DIMETHYL-CYCLOHEX-1-ENECARBONITRILE $$

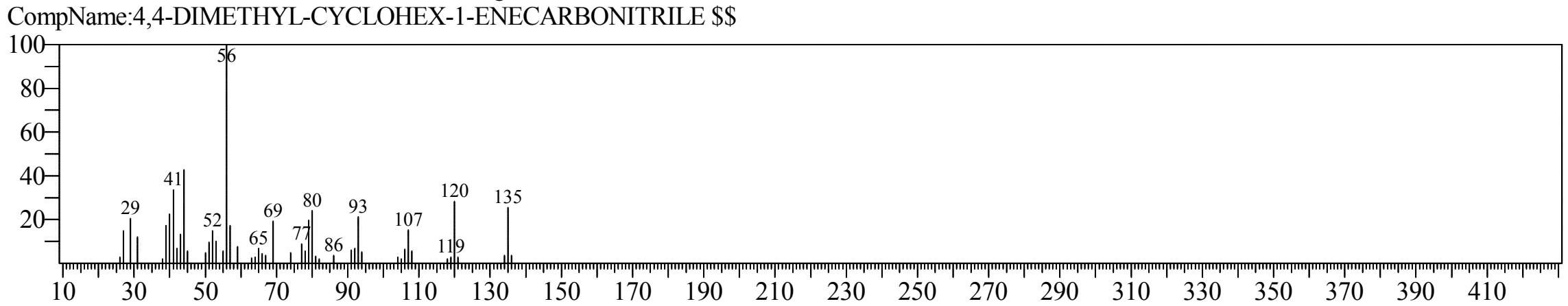

Hit#:14 Entry:98443 Library:WILEY7.LIB
SI:58 Formula:C10 H9 N3 O2 CAS:0-00-0 MolWeight:203 RetIndex:0
CompName:1-BENZYL-4-NITROIMIDAZOLE $$

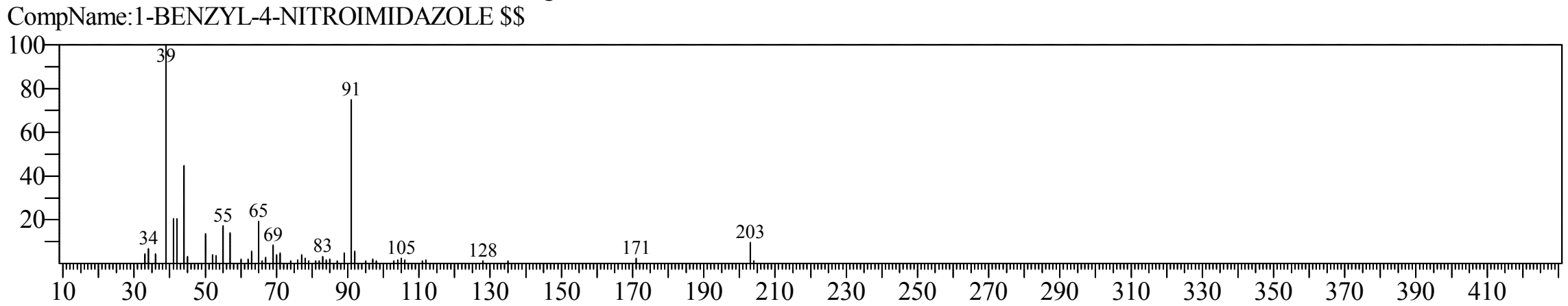

Hit#:15 Entry:9260 Library:NIST11.lib
SI:58 Formula:C9H13N CAS:0-00-0 MolWeight:135 RetIndex:1171
CompName:4,4-Dimethyl-cyclohex-1-enecarbonitrile $$ 4,4-Dimethyl-1-cyclohexene-1-carbonitrile # $$

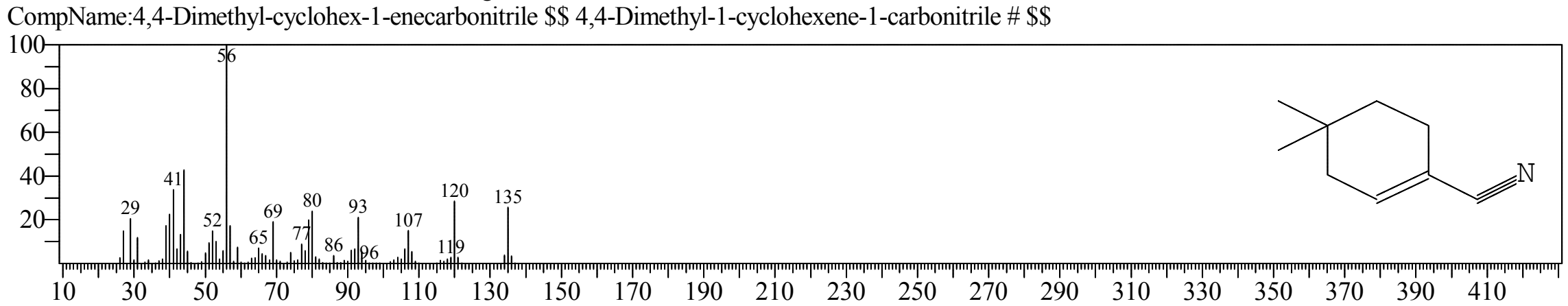

<< Target >>
Line#:18 R.Time:15.025(Scan#:1204) MassPeaks:52
RawMode:Averaged 15.017-15.033(1203-1205) BasePeak:44.00(1533)
BG Mode:Calc. from Peak Group 1 - Event 1 Scan

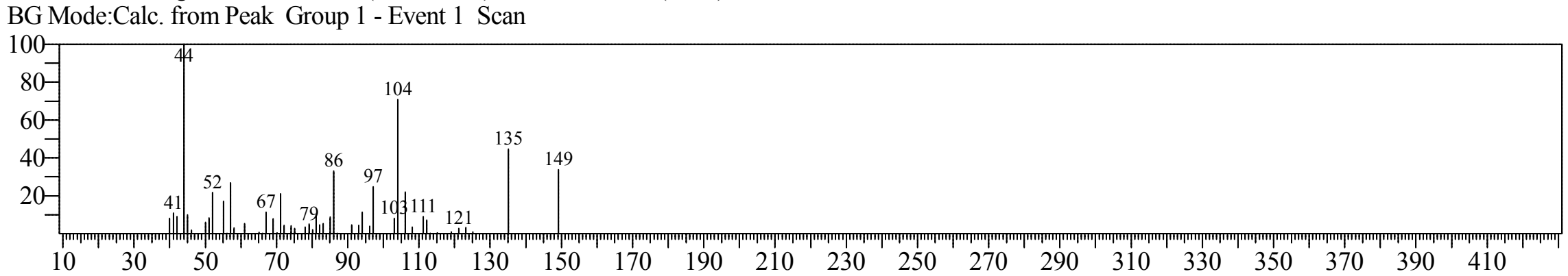

Hit#:16 Entry:150695 Library:NIST11.lib
SI:58 Formula:C14H17NO9 CAS:0-00-0 MolWeight:343 RetIndex:2271
CompName:Tetraacetyl-d-xylonic nitrile

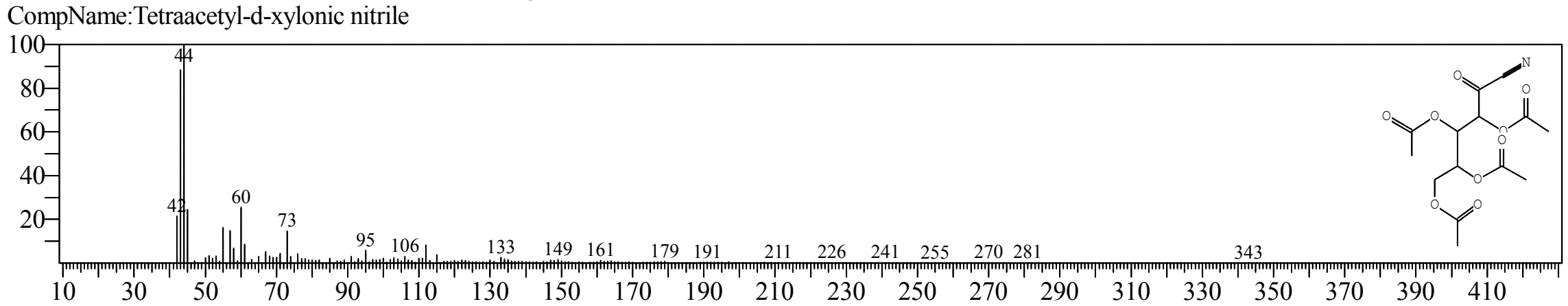

Hit#:17 Entry:123743 Library:NIST05.LIB
SI:58 Formula:C14H17NO9 CAS:0-00-0 MolWeight:343 RetIndex:2271
CompName:Tetraacetyl-d-xylonic nitrile

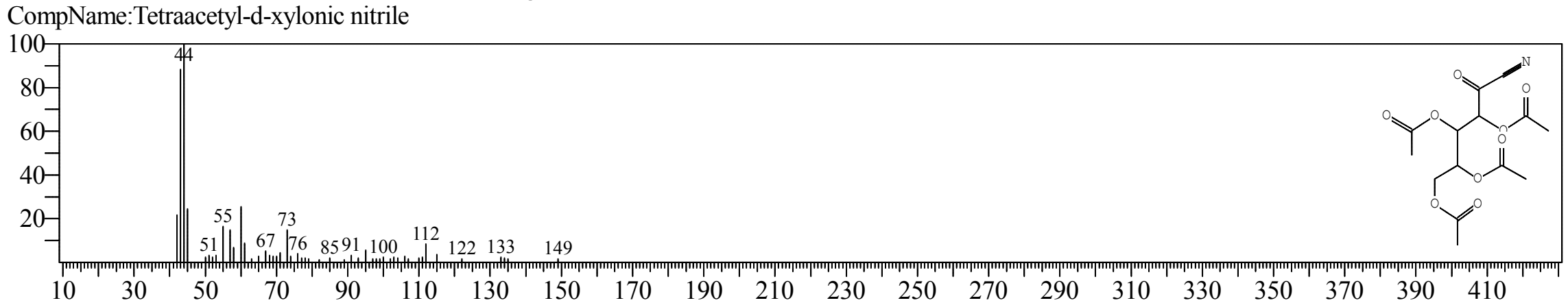

Hit#:18 Entry:16080 Library:NIST05.LIB
SI:58 Formula:C6H7N3O2 CAS:0-00-0 MolWeight:153 RetIndex:1693
CompName:Imidazole, 2-amino-5-[(2-carboxy)vinyl]-

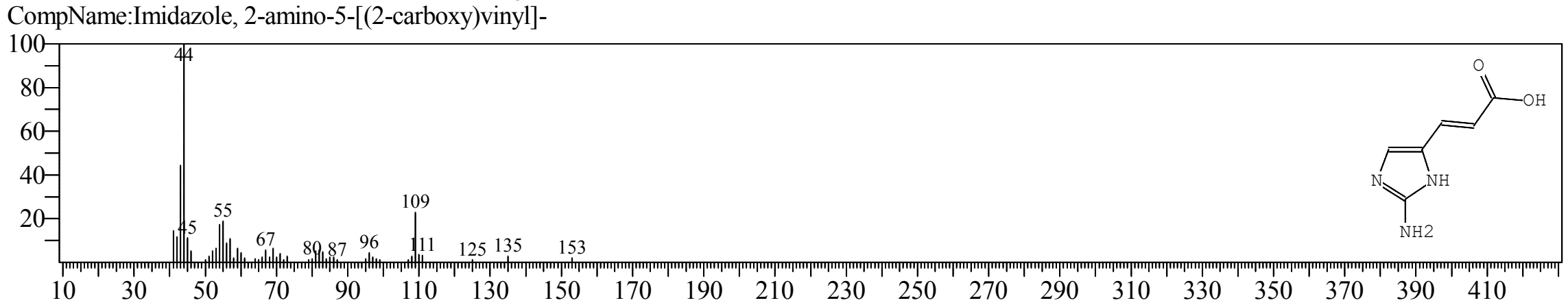

Hit#:19 Entry:16619 Library:NIST11.lib
SI:58 Formula:C6H7N3O2 CAS:0-00-0 MolWeight:153 RetIndex:1693
CompName:Imidazole, 2-amino-5-[(2-carboxy)vinyl]- $$ (2E)-3-(2-Amino-1H-imidazol-5-yl)-2-propenoic acid # $$

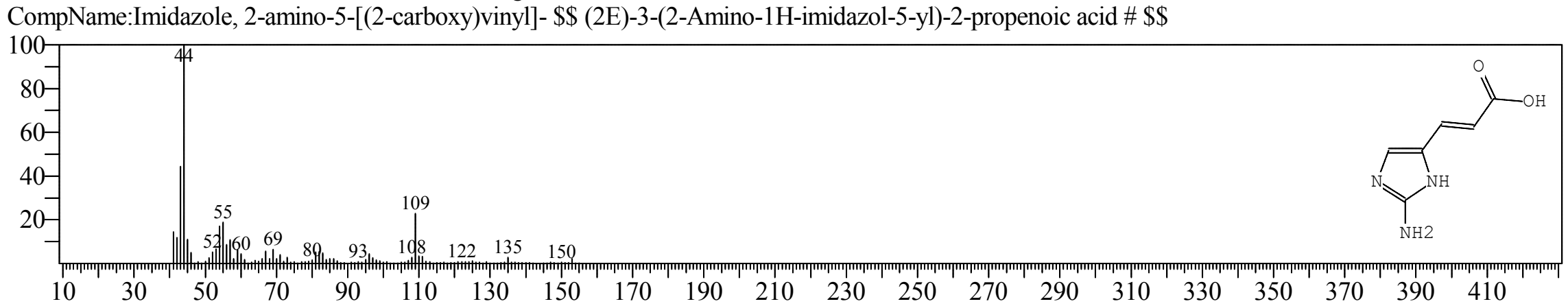

Hit#:20 Entry:180752 Library:WILEY7.LIB
SI:58 Formula:C14 H13 N3 O3 CAS:0-00-0 MolWeight:271 RetIndex:0
CompName:4,6-DIMETHYL(1H)PYRIDONE-2, -3-(3-NITROPHENYLMETHYLENAMINO) $$

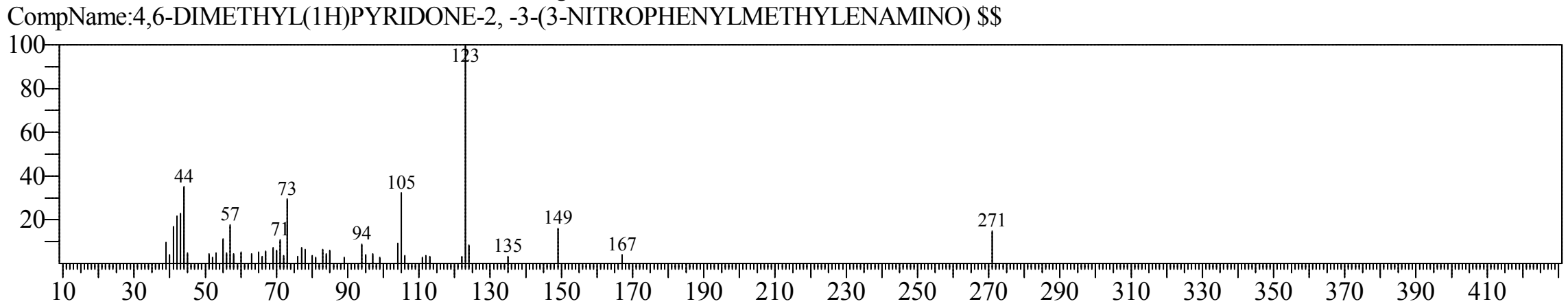

<< Target >>
Line#:19 R.Time:15.175(Scan#:1222) MassPeaks:48
RawMode:Averaged 15.167-15.183(1221-1223) BasePeak:73.05(9453)
BG Mode:Calc. from Peak Group 1 - Event 1 Scan

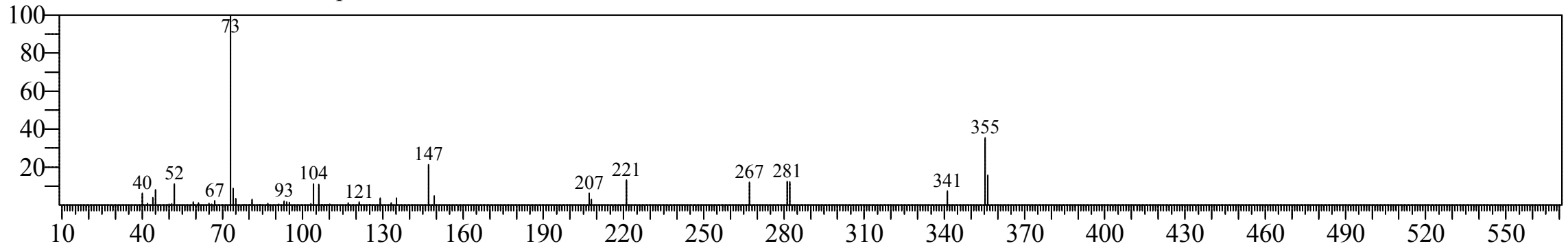

Hit#:1 Entry:25961 Library:NIST05s.LIB
SI:72 Formula:C16H30O4Si3 CAS:10586-16-0 MolWeight:370 RetIndex:1765
CompName:Benzoic acid, 2,4-bis[(trimethylsilyl)oxy]-, trimethylsilyl ester $$ .beta.-Resorcylic acid (tms) $$ 2,4-Bis(trimethylsiloxy)trimethylsilylbenzoate

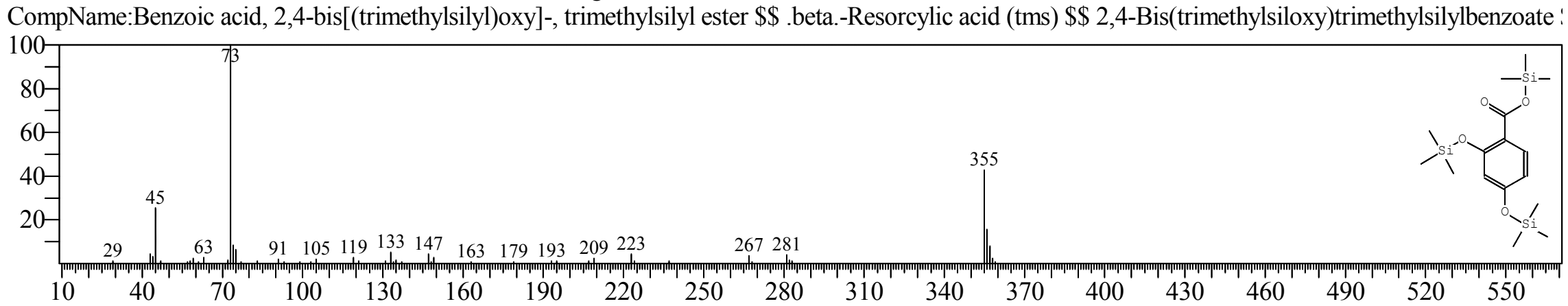

Hit#:2 Entry:267981 Library:WILEY7.LIB
SI:72 Formula:C16 H30 O4 SI3 CAS:3782-85-2 MolWeight:370 RetIndex:0
CompName:2,6-DIHYDROXYBENZOIC ACID 3TMS $$ trimethylsilyl [2,6-di(trimethylsiloxy)phenyl]-methanoate $$ Benzoic acid, 2,6-bis[(trimethylsily

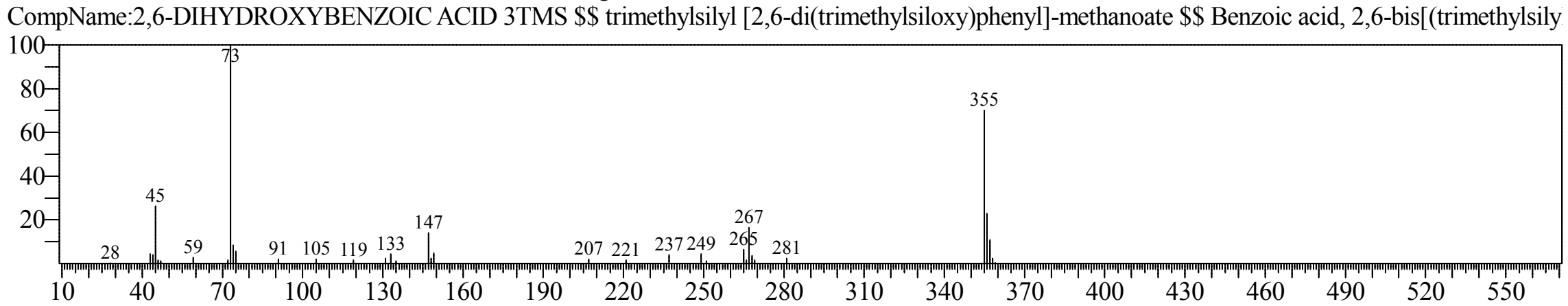

Hit#:3 Entry:310786 Library:WILEY7.LIB
SI:71 Formula:C20 H40 O5 SI4 CAS:37148-65-5 MolWeight:472 RetIndex:0
CompName:Benzeneacetic acid, .alpha.,3,4-tris[(trimethylsilyl)oxy]-, trimethylsilyl ester (CAS) TETRAKISTRIMETHYLSILYL 3,4-DIHYDROXYMAND

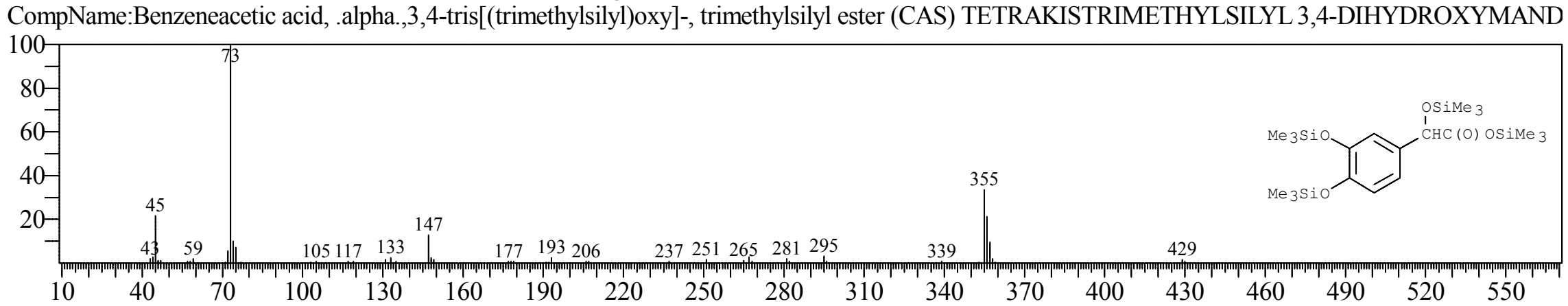

Hit#:4 Entry:208545 Library:NIST11.lib
SI:71 Formula:C22H42F3NO4Si4 CAS:0-00-0 MolWeight:553 RetIndex:2151
CompName:N-(Trifluoroacetyl)-N,O,O',O''-tetrakis(trimethylsilyl)norepinephrine $$ N-(2-(3,4-Bis[(trimethylsilyl)oxy]phenyl)-2-[(trimethylsilyl)oxy]ethyl)-

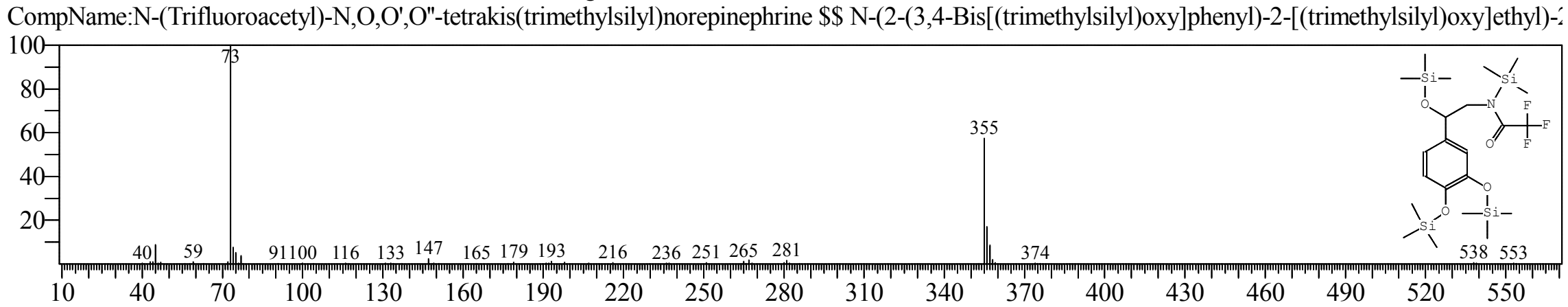

Hit#:5 Entry:159651 Library:NIST05.LIB
SI:71 Formula:C22H42F3NO4Si4 CAS:0-00-0 MolWeight:553 RetIndex:2151
CompName:N-(Trifluoroacetyl)-N,O,O',O''-tetrakis(trimethylsilyl)norepinephrine

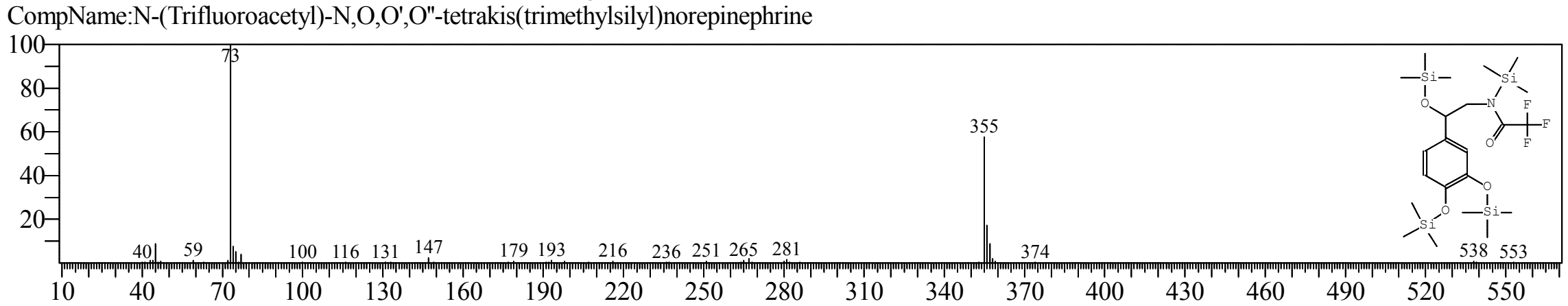

<< Target >>
Line#:19 R.Time:15.175(Scan#:1222) MassPeaks:48
RawMode:Averaged 15.167-15.183(1221-1223) BasePeak:73.05(9453)
BG Mode:Calc. from Peak Group 1 - Event 1 Scan

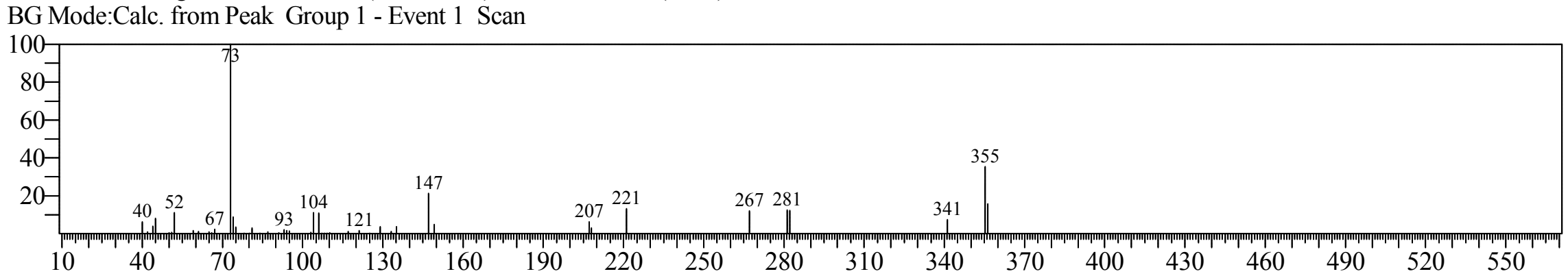

Hit#:6 Entry:27443 Library:NIST05s.LIB
SI:71 Formula:C24H34F5NO3Si3 CAS:55429-13-5 MolWeight:563 RetIndex:2572
CompName:Benzeneethanamine, N-[(pentafluorophenyl)methylene]-.beta.,3,4-tris[(trimethylsilyl)oxy]- $$ N-(Pentafluorobenzylidene)-.beta.,3,4-tris(trimetl

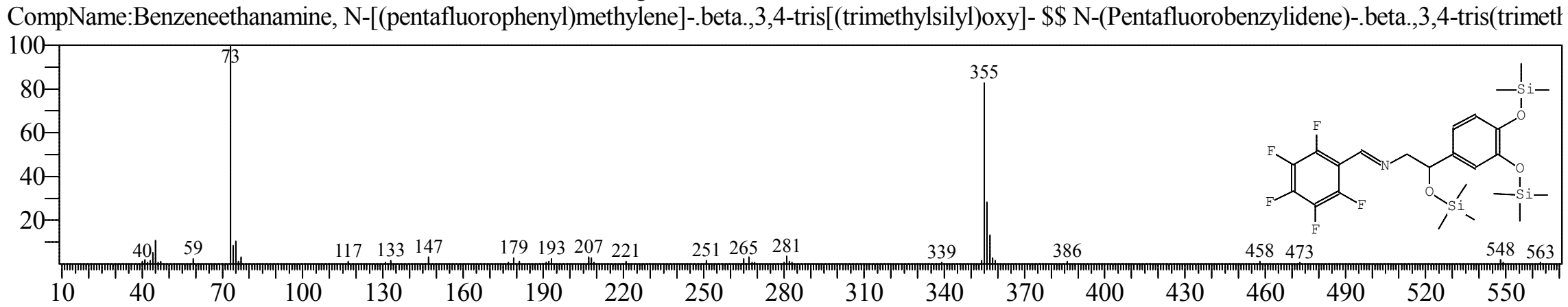

Hit#:7 Entry:317266 Library:WILEY7.LIB
SI:70 Formula:C14 H44 O6 SI7 CAS:0-00-0 MolWeight:504 RetIndex:0
CompName:1,1,3,3,5,5,7,7,9,9,11,11,13,13-TETRADECAMETHYL-HEPTASILOXANE $$

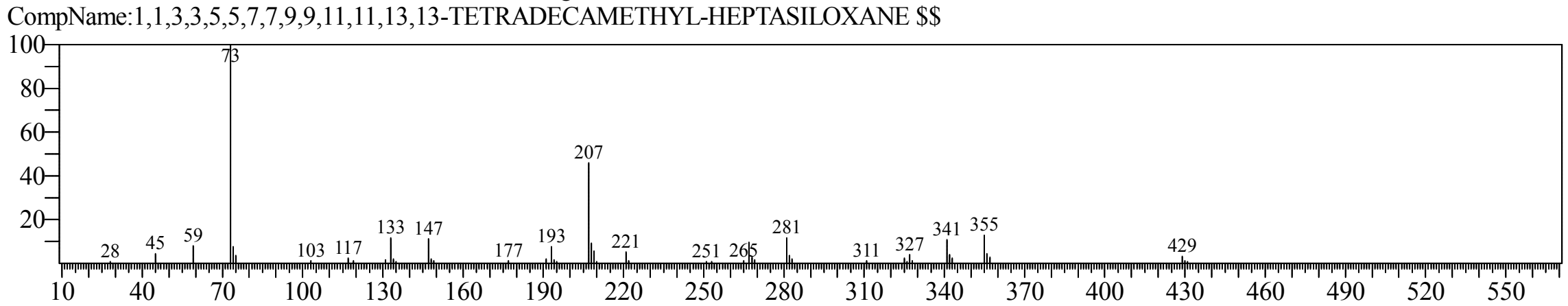

Hit#:8 Entry:157431 Library:NIST05.LIB
SI:70 Formula:C14H44O6Si7 CAS:19095-23-9 MolWeight:504 RetIndex:1526
CompName:Heptasiloxane, 1,1,3,3,5,5,7,7,9,9,11,11,13,13-tetradecamethyl- $$ 1,1,3,3,5,5,7,7,9,9,11,11,13,13-Tetradecamethylheptasiloxane # $$

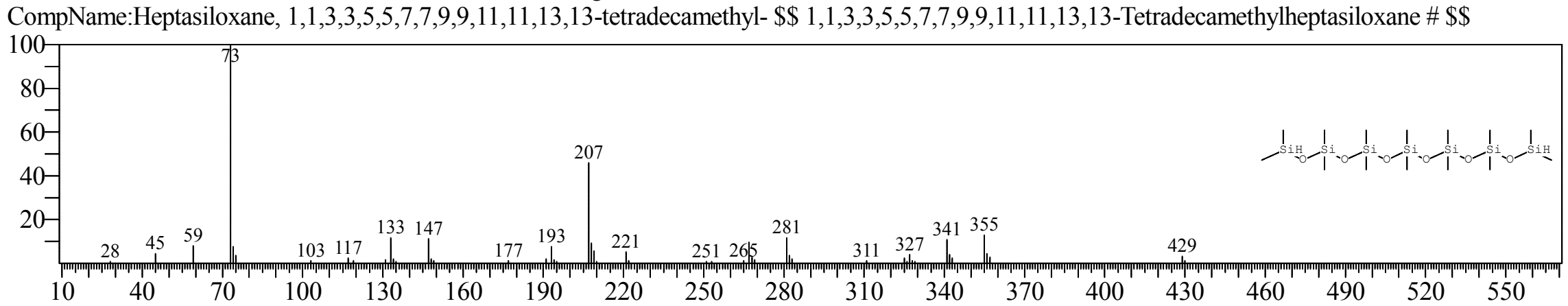

Hit#:9 Entry:310441 Library:WILEY7.LIB
SI:70 Formula:C20 H40 O5 SI4 CAS:0-00-0 MolWeight:472 RetIndex:0
CompName:3,4-DIHYDROXYMANDELIC ACID-TETRATMS $$

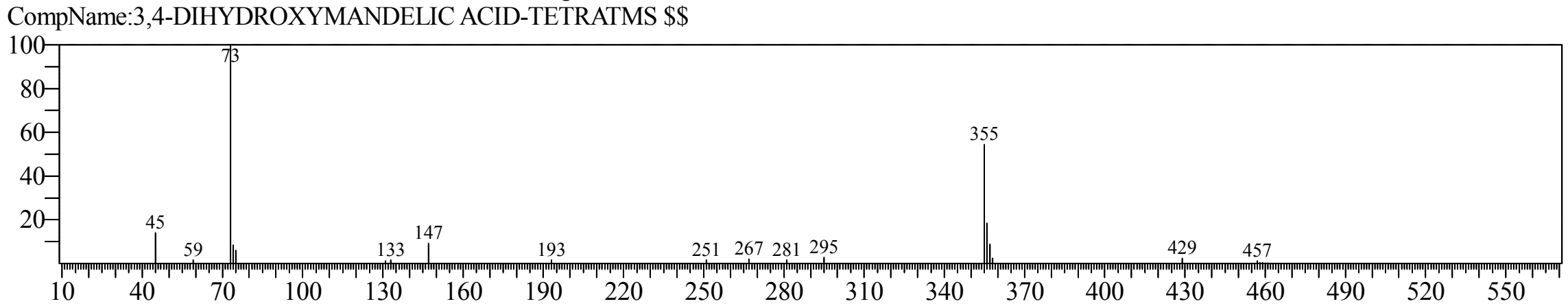

Hit#:10 Entry:156882 Library:NIST05.LIB
SI:70 Formula:C20H36F3NO4Si3 CAS:54135-51-2 MolWeight:495 RetIndex:2042
CompName:N-(Trifluoracetyl)-O,O',O''-tris(trimethylsilyl)epinephrine $$ N-(Trifluoracetyl)-O,O',O''-tris(trimethylsilyl)epinephrine $$ N-(2-(3,4-Bis[(trimetl

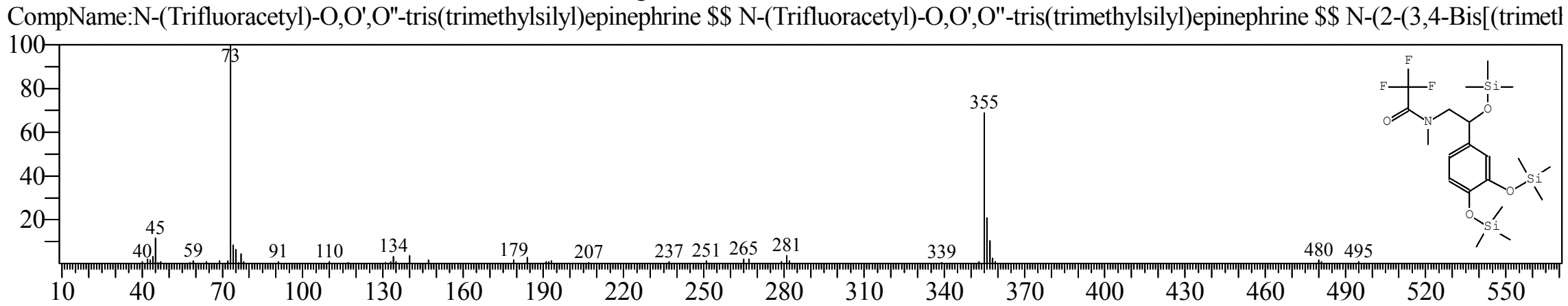

<< Target >>
Line#:19 R.Time:15.175(Scan#:1222) MassPeaks:48
RawMode:Averaged 15.167-15.183(1221-1223) BasePeak:73.05(9453)
BG Mode:Calc. from Peak Group 1 - Event 1 Scan

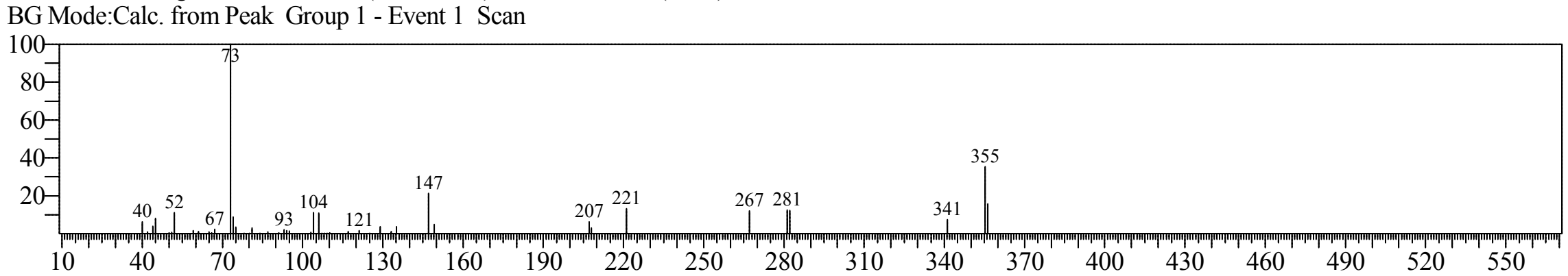

Hit#:11 Entry:153706 Library:NIST05.LIB
SI:70 Formula:C14H42O5Si6 CAS:2003-92-1 MolWeight:458 RetIndex:1252
CompName:1,1,1,3,5,7,7,7-Octamethyl-3,5-bis(trimethylsiloxy)tetrasiloxane $$ 1,1,1,3,5,7,7,7-Octamethyl-3,5-bis[(trimethylsilyl)oxy]tetrasiloxane # $$

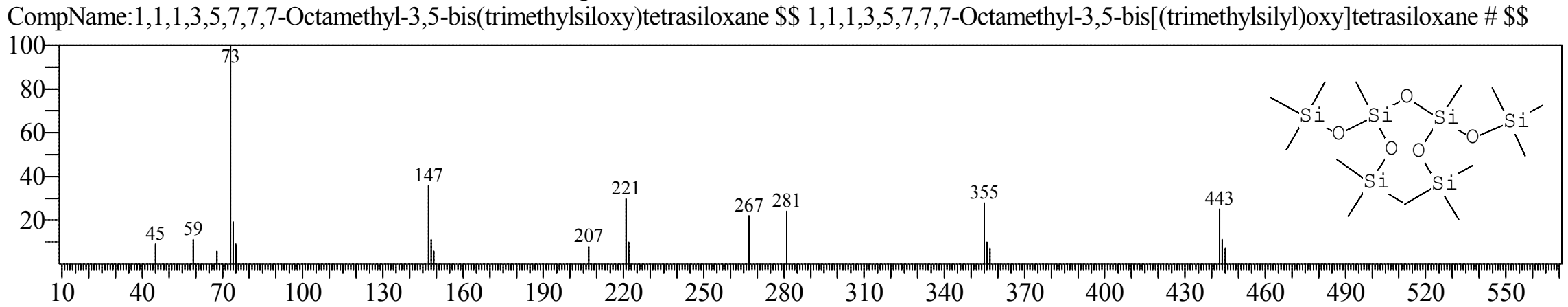

Hit#:12 Entry:160407 Library:NIST05.LIB
SI:69 Formula:C16H50O7Si8 CAS:19095-24-0 MolWeight:578 RetIndex:1710
CompName:Octasiloxane, 1,1,3,3,5,5,7,7,9,9,11,11,13,13,15,15-hexadecamethyl- $$ 1,1,3,3,5,5,7,7,9,9,11,11,13,13,15,15-Hexadecamethyloctasiloxane # $

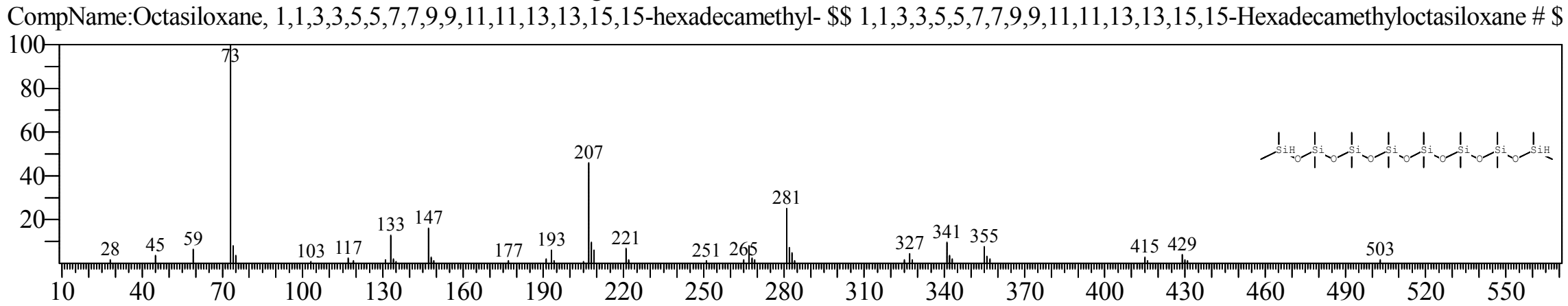

Hit#:13 Entry:25962 Library:NIST05s.LIB
SI:69 Formula:C16H30O4Si3 CAS:3618-20-0 MolWeight:370 RetIndex:1765
CompName:Benzoic acid, 2,5-bis(trimethylsiloxy)-, trimethylsilyl ester $$ Gentisic acid (tms) $$ Trimethylsilyl 2,5-bis[(trimethylsilyl)oxy]benzoate # $$

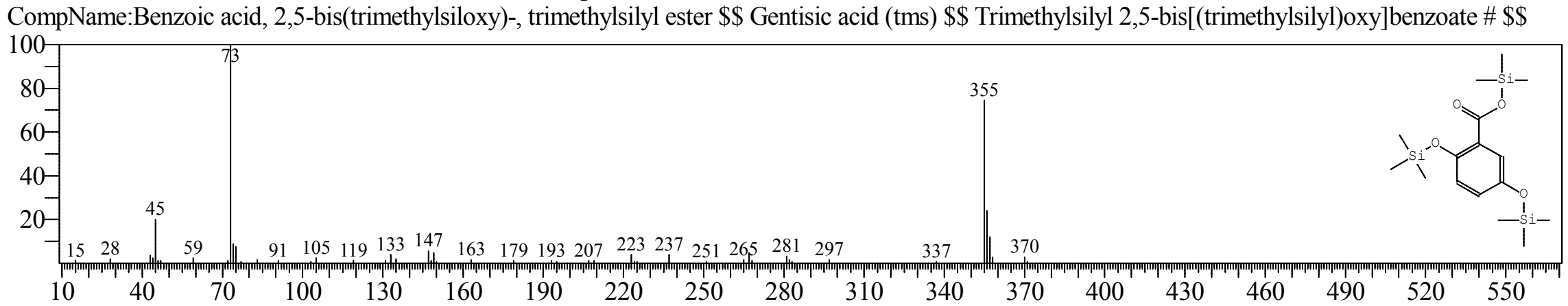

Hit#:14 Entry:26522 Library:NIST05s.LIB
SI:69 Formula:C18H37NO3Si3 CAS:10538-85-9 MolWeight:399 RetIndex:2007
CompName:Phenethylamine, N-methyl-.beta.,3,4-tris(trimethylsiloxy)- $$ 2-(3,4-Bis[(trimethylsilyl)oxy]phenyl)-N-methyl-2-[(trimethylsilyl)oxy]ethanamin

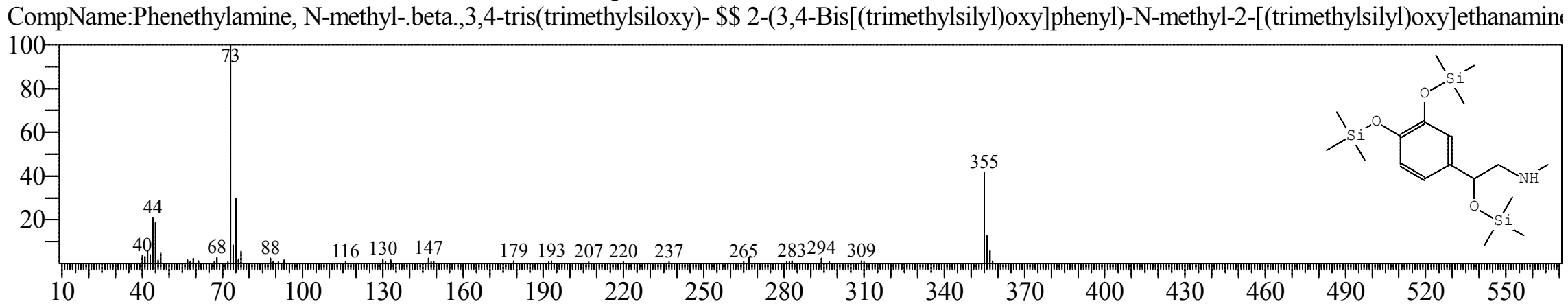

Hit#:15 Entry:153730 Library:NIST05.LIB
SI:68 Formula:C20H42O4Si4 CAS:56114-62-6 MolWeight:458 RetIndex:1993
CompName:Silane, [[4-[1,2-bis[(trimethylsilyl)oxy]ethyl]-1,2-phenylene]bis(oxy)]bis[trimethyl- $$ Silane, [[4-[1,2-bis[(trimethylsilyl)oxy]ethyl]-1,2-phenyl

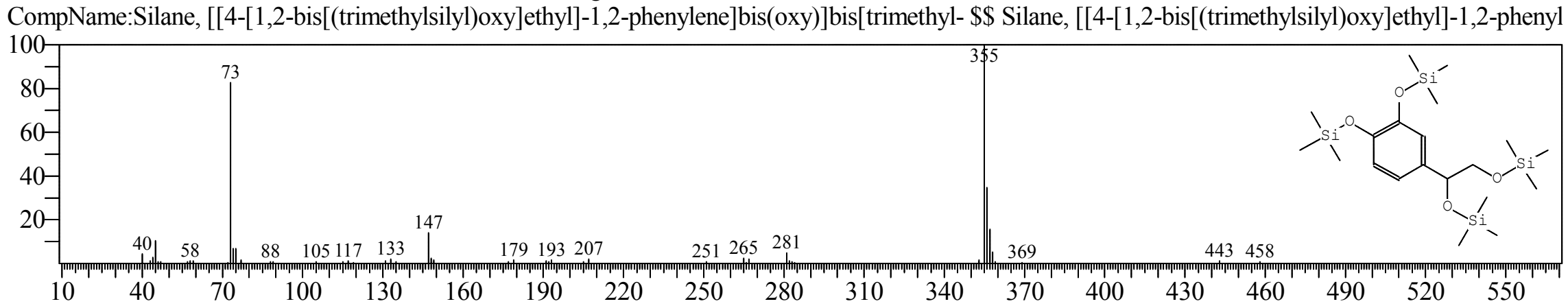

<< Target >>
Line#:19 R.Time:15.175(Scan#:1222) MassPeaks:48
RawMode:Averaged 15.167-15.183(1221-1223) BasePeak:73.05(9453)
BG Mode:Calc. from Peak Group 1 - Event 1 Scan

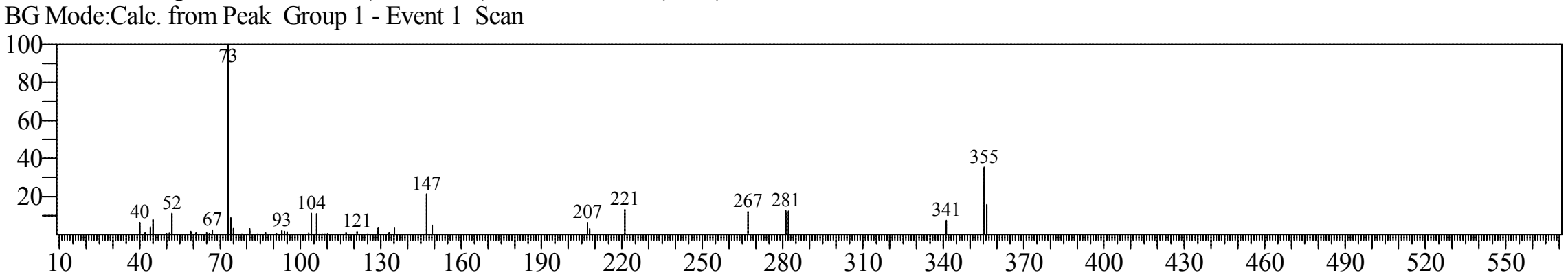

Hit#:16 Entry:202203 Library:NIST11.lib
SI:68 Formula:C19H34F3NO4Si3 CAS:0-00-0 MolWeight:481 RetIndex:2142
CompName:N-(Trifluoroacetyl)-O,O',O''-tris(trimethylsilyl)norepinephrine $$ N-(2-(3,4-Bis[(trimethylsilyl)oxy]phenyl)-2-[(trimethylsilyl)oxy]ethyl)-2,2,2-tr

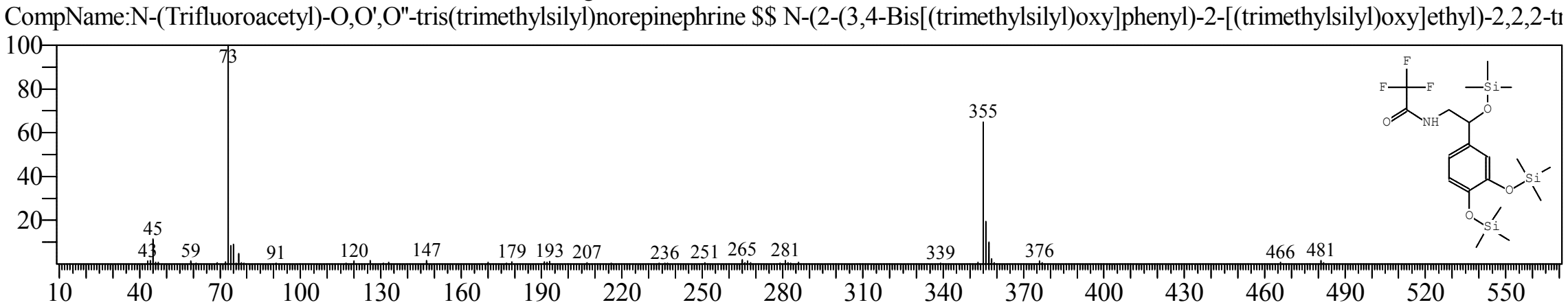

Hit#:17 Entry:155815 Library:NIST05.LIB
SI:68 Formula:C19H34F3NO4Si3 CAS:0-00-0 MolWeight:481 RetIndex:2142
CompName:N-(Trifluoroacetyl)-O,O',O''-tris(trimethylsilyl)norepinephrine

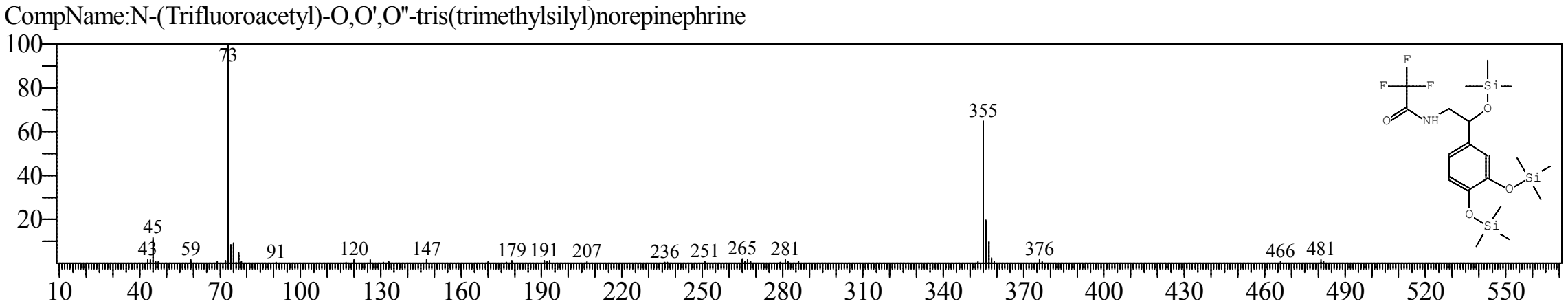

Hit#:18 Entry:150228 Library:NIST05.LIB
SI:68 Formula:C12H38O5Si6 CAS:995-82-4 MolWeight:430 RetIndex:1341
CompName:Hexasiloxane, 1,1,3,3,5,5,7,7,9,9,11,11-dodecamethyl- $$ 1,1,3,3,5,5,7,7,9,9,11,11-Dodecamethylhexasiloxane # $$

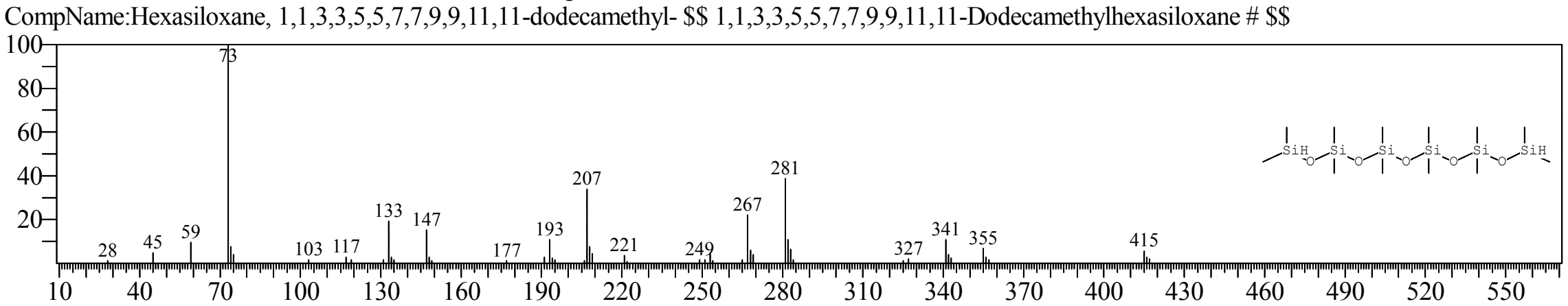

Hit#:19 Entry:297348 Library:WILEY7.LIB
SI:68 Formula:C12 H38 O5 SI6 CAS:0-00-0 MolWeight:430 RetIndex:0
CompName:1,1,3,3,5,5,7,7,9,9,11,11-DODECAMETHYL-HEXASILOXANE $$

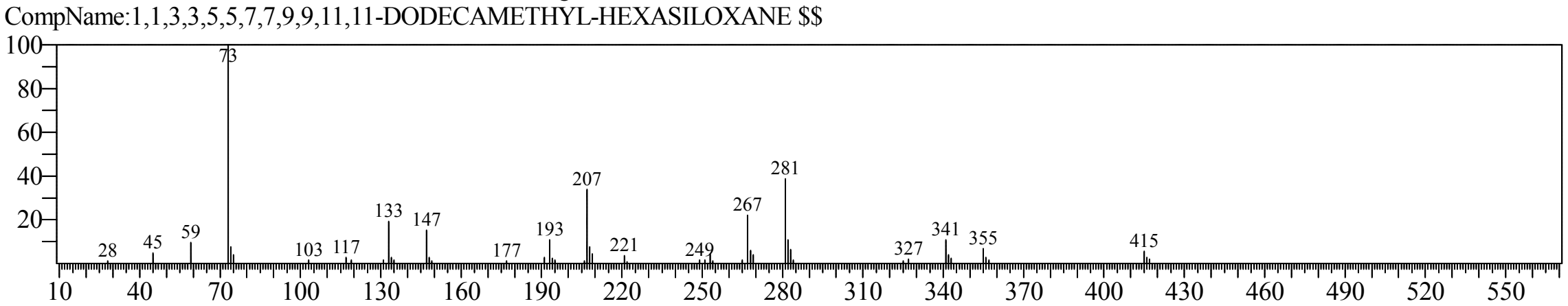

Hit#:20 Entry:326547 Library:WILEY7.LIB
SI:68 Formula:C24 H51 N O5 SI5 CAS:0-00-0 MolWeight:573 RetIndex:0
CompName:1,3-BIS(TRIMETHYLSILYLOXY)-2-TRIMETHYLSILYLAMINO-3-[3',4'-BIS(TRIMETHYLSILYLOXY)PHENYL]-PROPANONE $$

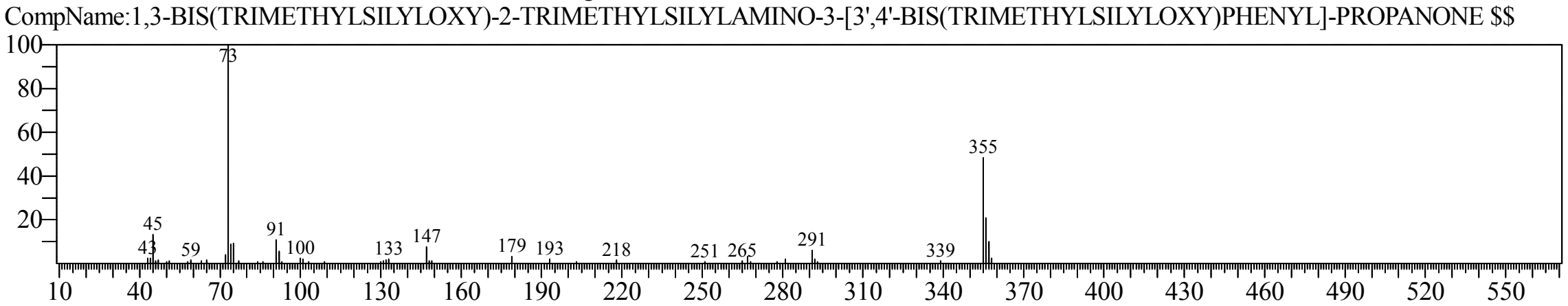

<< Target >>
Line#:20   R.Time:15.350(Scan#:1243) MassPeaks:76
RawMode:Averaged 15.342-15.358(1242-1244) BasePeak:71.10(48404)
BG Mode:Calc. from Peak Group 1 - Event 1 Scan

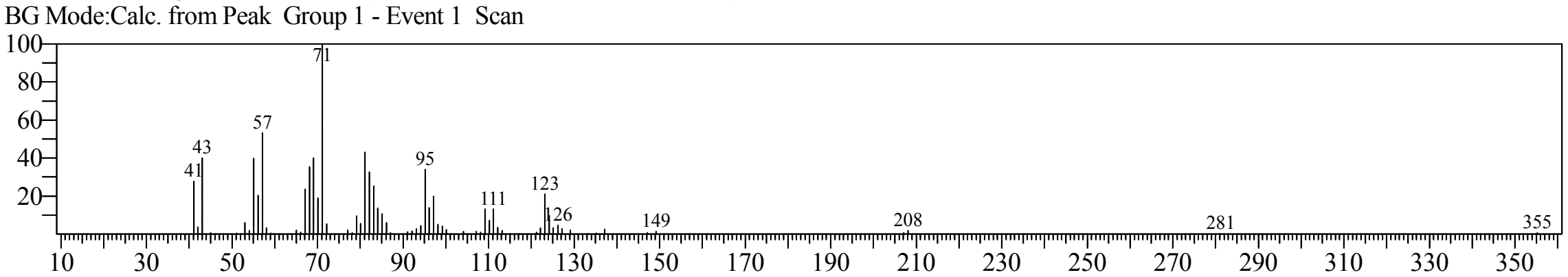

Hit#:1 Entry:25944 Library:NIST11s.lib
SI:93 Formula:C20H40O CAS:150-86-7 MolWeight:296 RetIndex:2045
CompName:Phytol $$ 2-Hexadecen-1-ol, 3,7,11,15-tetramethyl-, [R-[R*,R*-(E)]]- $$ trans-Phytol $$ 3,7,11,15-Tetramethyl-2-hexadecen-1-ol-, (2E,7R,11R

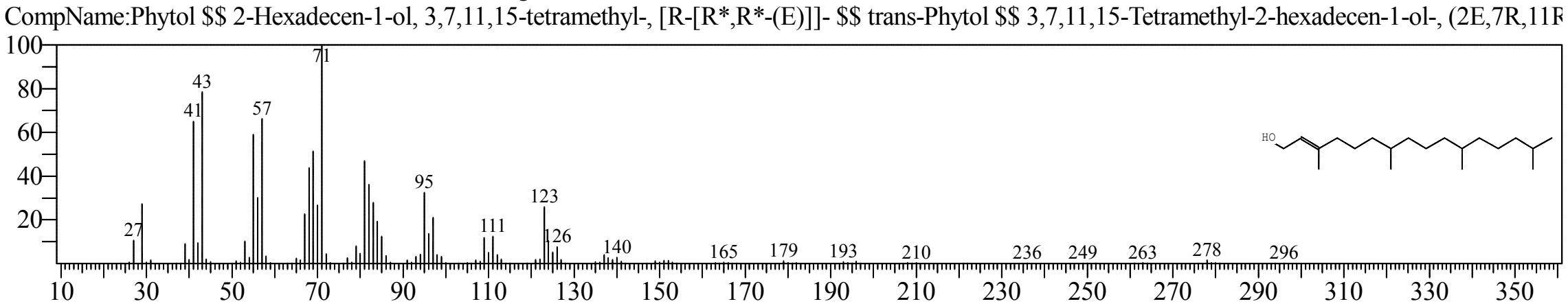

Hit#:2 Entry:207638 Library:WILEY7.LIB
SI:91 Formula:C20 H40 O CAS:0-00-0 MolWeight:296 RetIndex:0
CompName:PHYTOL ISOMER $$

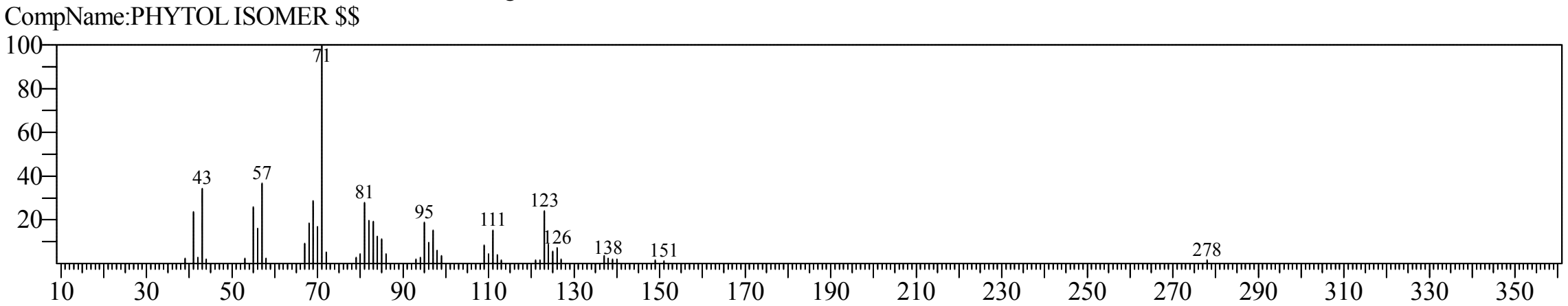

Hit#:3 Entry:9771 Library:NIST05s.LIB
SI:88 Formula:C10H20O CAS:491-02-1 MolWeight:156 RetIndex:1164
CompName:Cyclohexanol, 5-methyl-2-(1-methylethyl)-, (1.alpha.,2.alpha.,5.alpha.)- $$ Menthol, cis-1,3,cis-1,4- $$ Neoisomenthol $$ 2-Isopropyl-5-methyl

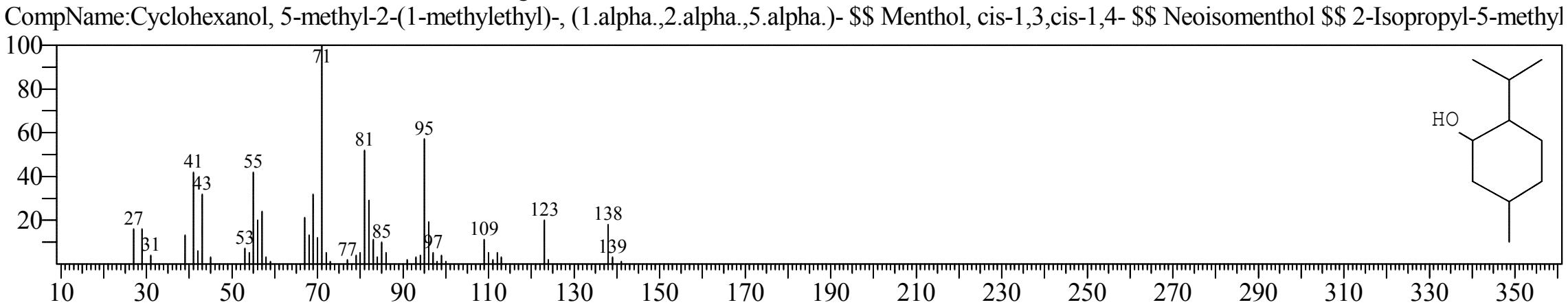

Hit#:4 Entry:18670 Library:NIST11.lib
SI:88 Formula:C10H20O CAS:23283-97-8 MolWeight:156 RetIndex:1164
CompName:Cyclohexanol, 5-methyl-2-(1-methylethyl)-, [1S-(1.alpha.,2.beta.,5.beta.)]- $$ (1S,2R,5R)-(+)-Isomenthol $$ 2-Isopropyl-5-methylcyclohexanol

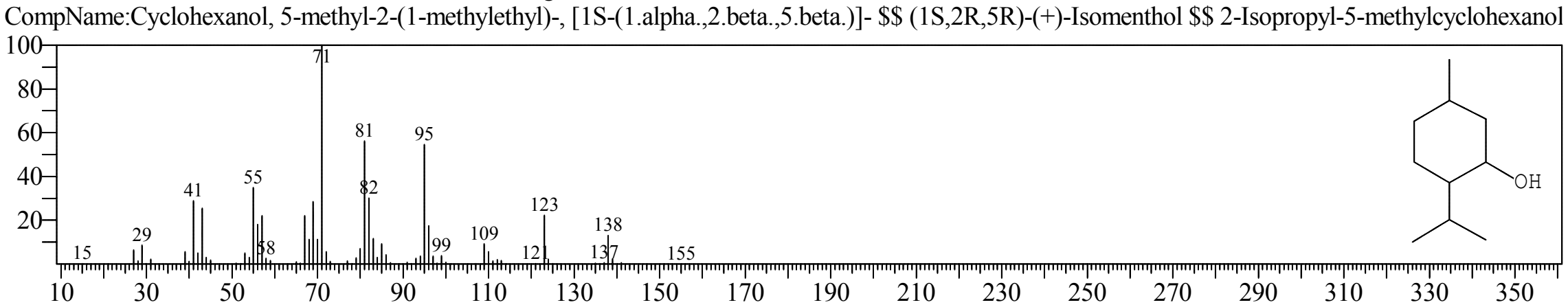

Hit#:5 Entry:9769 Library:NIST05s.LIB
SI:88 Formula:C10H20O CAS:490-99-3 MolWeight:156 RetIndex:1164
CompName:Cyclohexanol, 5-methyl-2-(1-methylethyl)-, (1.alpha.,2.beta.,5.beta.)- $$ Menthol, trans-1,3,cis-1,4- $$ Isomenthol $$ 2-Isopropyl-5-methylcycl

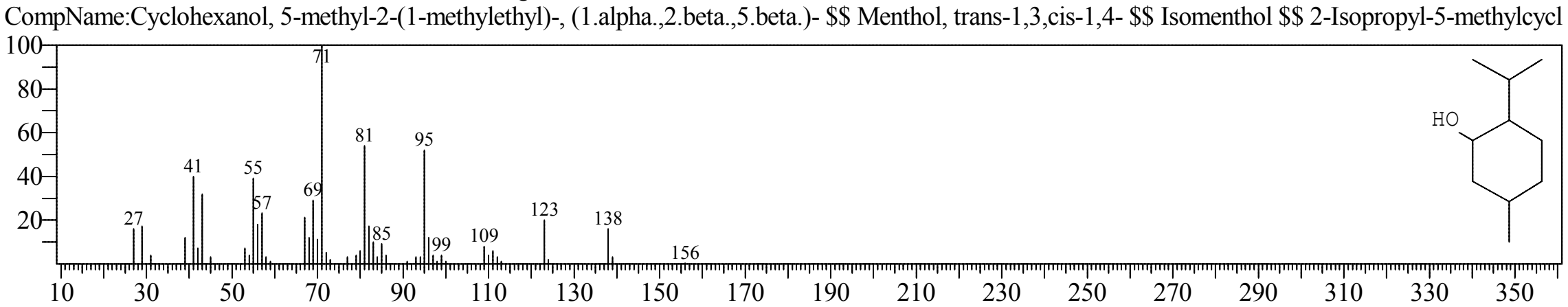

<< Target >>
Line#:20 R.Time:15.350(Scan#:1243) MassPeaks:76
RawMode:Averaged 15.342-15.358(1242-1244) BasePeak:71.10(48404)
BG Mode:Calc. from Peak Group 1 - Event 1 Scan

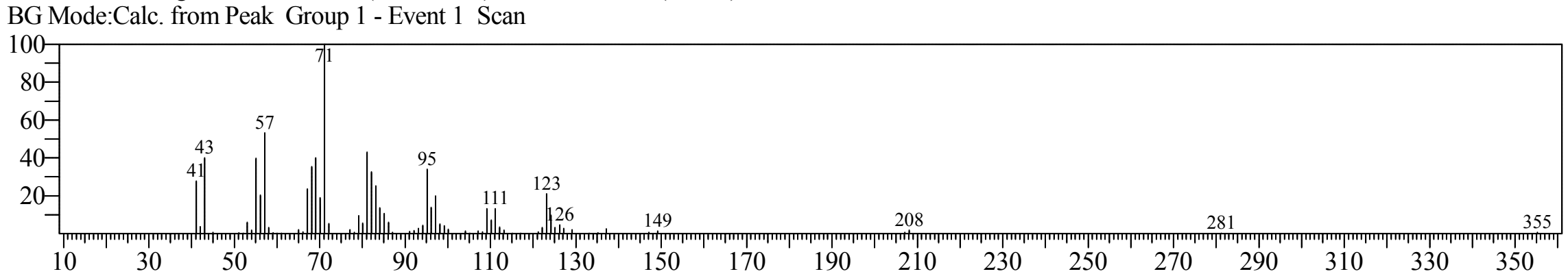

Hit#:6 Entry:147508 Library:NIST11.lib
SI:88 Formula:C22H42O2 CAS:0-00-0 MolWeight:338 RetIndex:2089
CompName:Isophytol, acetate

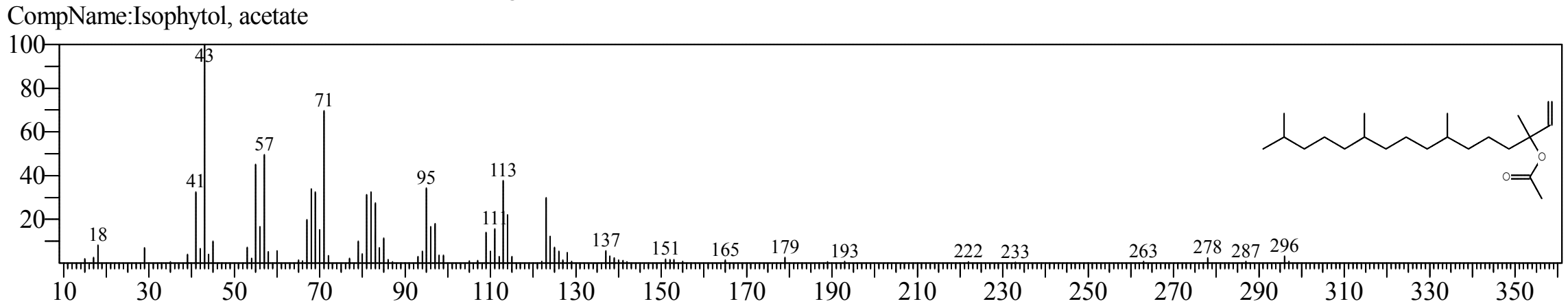

Hit#:7 Entry:45558 Library:WILEY7.LIB
SI:88 Formula:C10 H20 O CAS:0-00-0 MolWeight:156 RetIndex:0
CompName:D-NEOISOMENTHOL $$

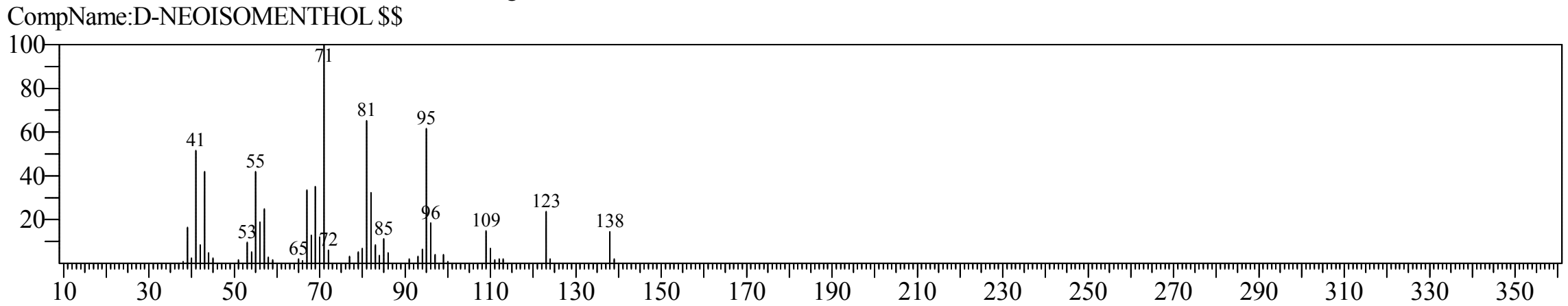

Hit#:8 Entry:45606 Library:WILEY7.LIB
SI:87 Formula:C10 H19 D O CAS:5113-62-2 MolWeight:156 RetIndex:0
CompName:TRANS-2-(1-METHYLETHYL-1-D)-CIS-5-METHYL-1-CYCLOHEXANOL $$ Menthol-8-d (CAS)

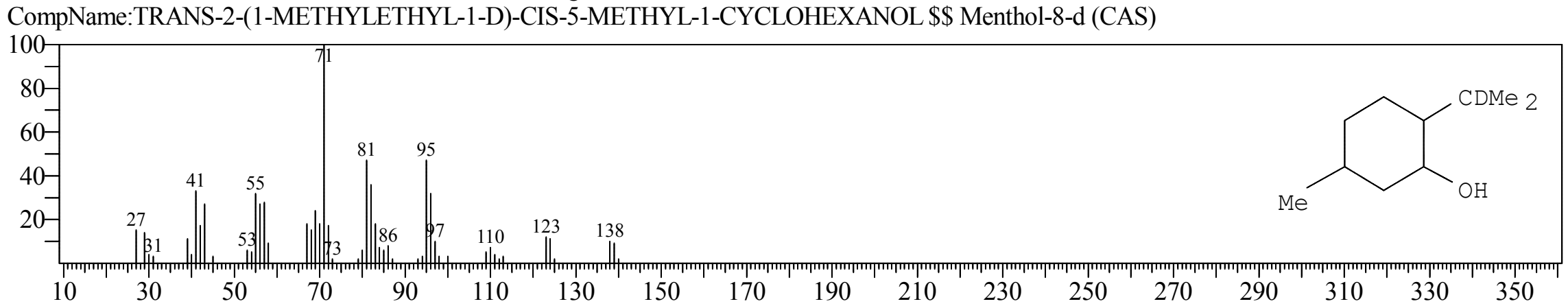

Hit#:9 Entry:46173 Library:WILEY7.LIB
SI:87 Formula:C10 H20 O CAS:1490-04-6 MolWeight:156 RetIndex:0
CompName:Cyclohexanol, 5-methyl-2-(1-methylethyl)- (CAS) 3-p-Menthanol $$ Menthol $$ p-Menthan-3-ol $$ Menthyl alcohol $$ 3-Hydroxy-p-menthan

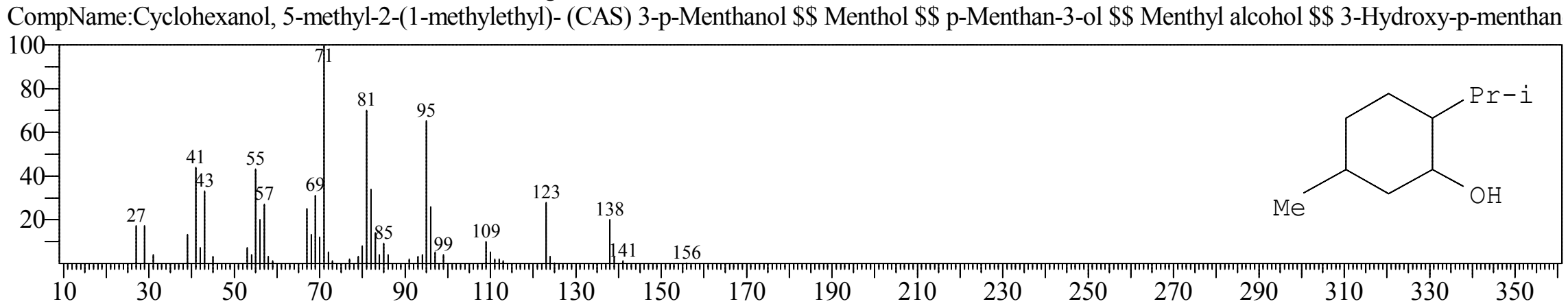

Hit#:10 Entry:46165 Library:WILEY7.LIB
SI:87 Formula:C10 H20 O CAS:89-78-1 MolWeight:156 RetIndex:0
CompName:Menthol $$ Cyclohexanol, 5-methyl-2-(1-methylethyl)-, (1.alpha.,2.beta.,5.alpha.)- (CAS) TRANS-2-ISOPROPYL-CIS-5-METHYL-1-CYCLC

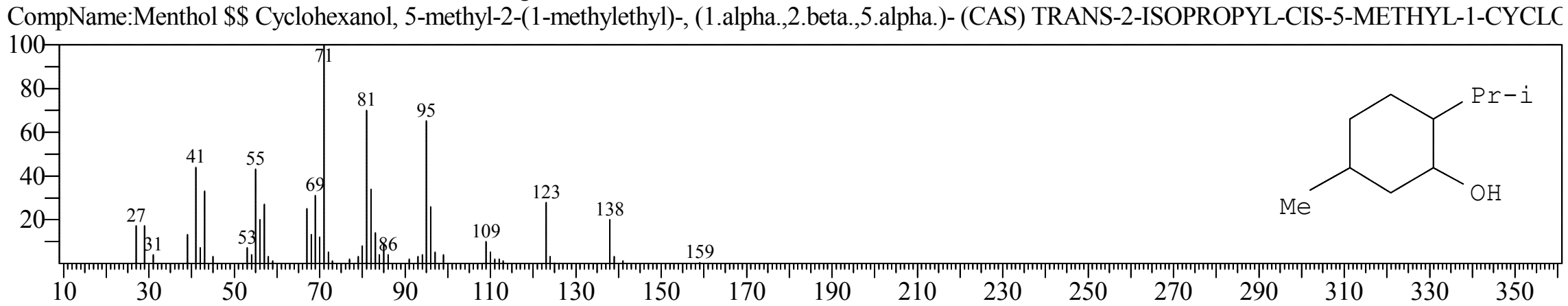

<< Target >>
Line#:20 R.Time:15.350(Scan#:1243) MassPeaks:76
RawMode:Averaged 15.342-15.358(1242-1244) BasePeak:71.10(48404)
BG Mode:Calc. from Peak Group 1 - Event 1 Scan

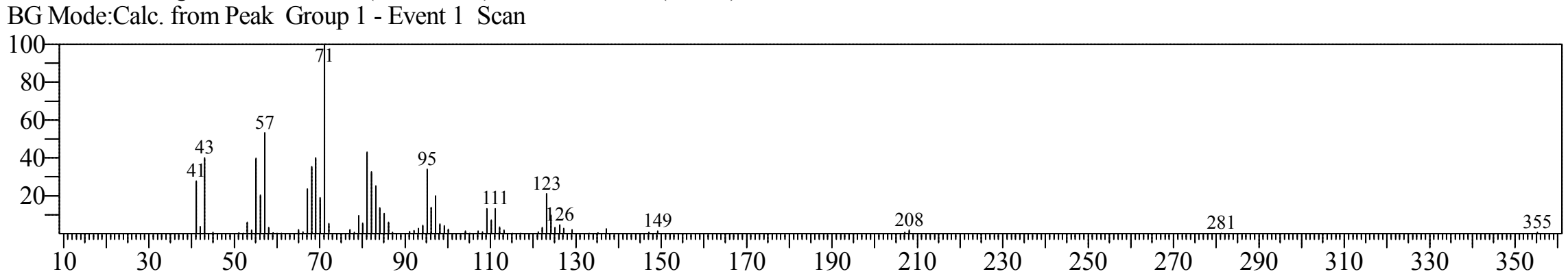

Hit#:11 Entry:45557 Library:WILEY7.LIB
SI:87 Formula:C10 H20 O CAS:0-00-0 MolWeight:156 RetIndex:0
CompName:(+)-NEOISOMENTHOL $$

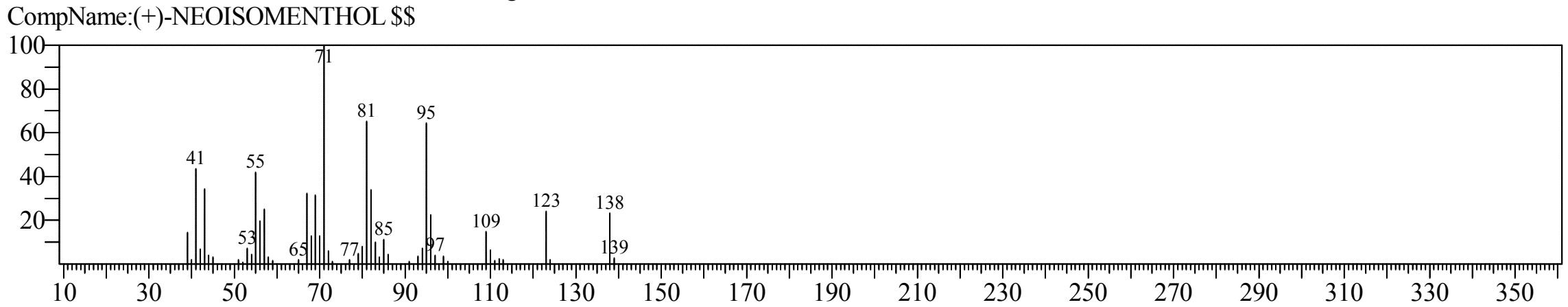

Hit#:12 Entry:18034 Library:NIST05.LIB
SI:87 Formula:C10H20O CAS:0-00-0 MolWeight:156 RetIndex:1164
CompName:(+)-Isomenthol

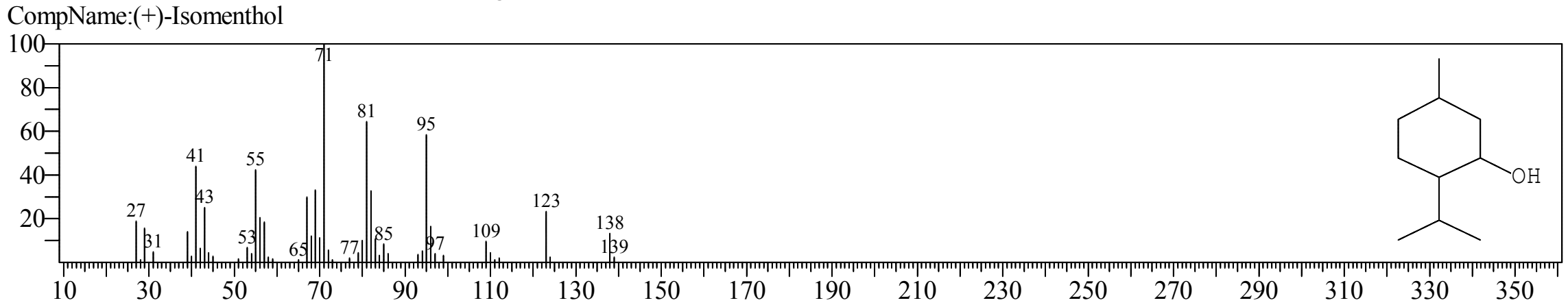

Hit#:13 Entry:18669 Library:NIST11.lib
SI:87 Formula:C10H20O CAS:0-00-0 MolWeight:156 RetIndex:1164
CompName:(+)-Isomenthol $$ 2-Isopropyl-5-methylcyclohexanol # $$

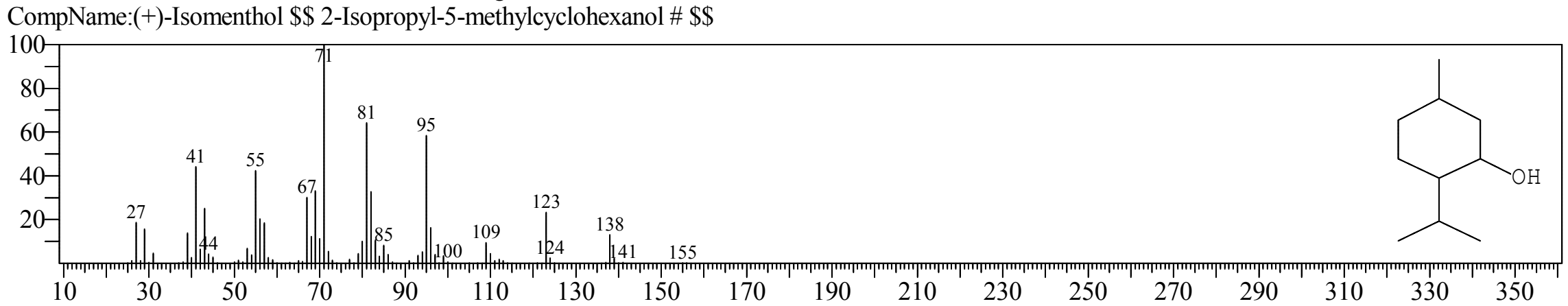

Hit#:14 Entry:18038 Library:NIST05.LIB
SI:87 Formula:C10H20O CAS:21129-27-1 MolWeight:156 RetIndex:1127
CompName:Cyclohexanol, 1-methyl-4-(1-methylethyl)- $$ 1-Methyl-4-(1-methylethyl)cyclohexanol $$ 4-Isopropyl-1-methylcyclohexanol # $$

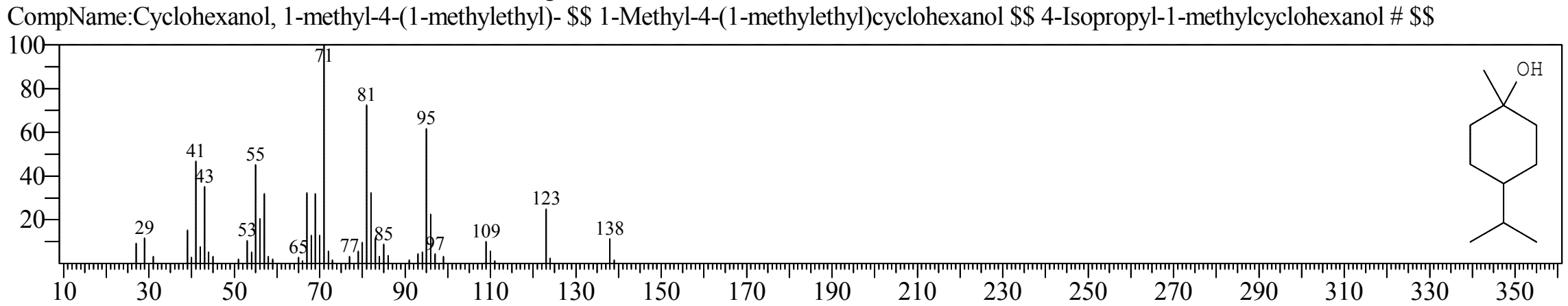

Hit#:15 Entry:46189 Library:WILEY7.LIB
SI:86 Formula:C10 H20 O CAS:491-01-0 MolWeight:156 RetIndex:0
CompName:neo-Menthol $$ Cyclohexanol, 5-methyl-2-(1-methylethyl)-, (1.alpha.,2.alpha.,5.beta.)- (CAS) CIS-2-ISOPROPYL-TRANS-5-METHYL-1-CY

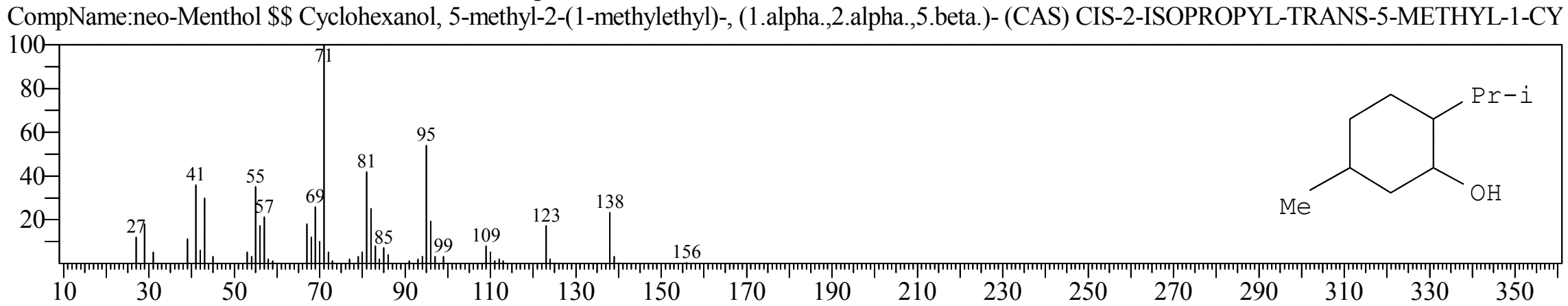

<< Target >>
Line#:20 R.Time:15.350(Scan#:1243) MassPeaks:76
RawMode:Averaged 15.342-15.358(1242-1244) BasePeak:71.10(48404)
BG Mode:Calc. from Peak Group 1 - Event 1 Scan

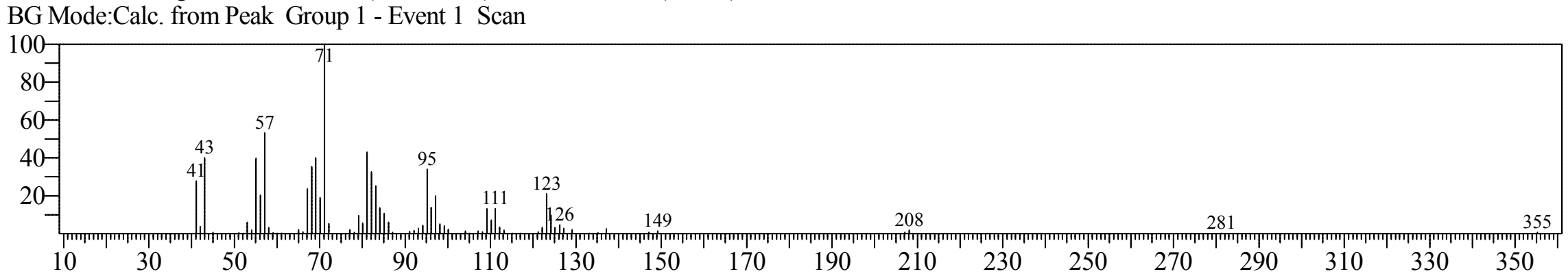

Hit#:16 Entry:46143 Library:WILEY7.LIB
SI:86 Formula:C10 H20 O CAS:26489-01-0 MolWeight:156 RetIndex:0
CompName:dl-Citronellol $$ 6-Octen-1-ol, 3,7-dimethyl-, (.+-.)- (CAS) Dihydrogeraniol $$ (.+-.)-Citronellol $$ 6-Octen-1-ol, 3,7-dimethyl-, (.+/-.)- $$ (.+/-

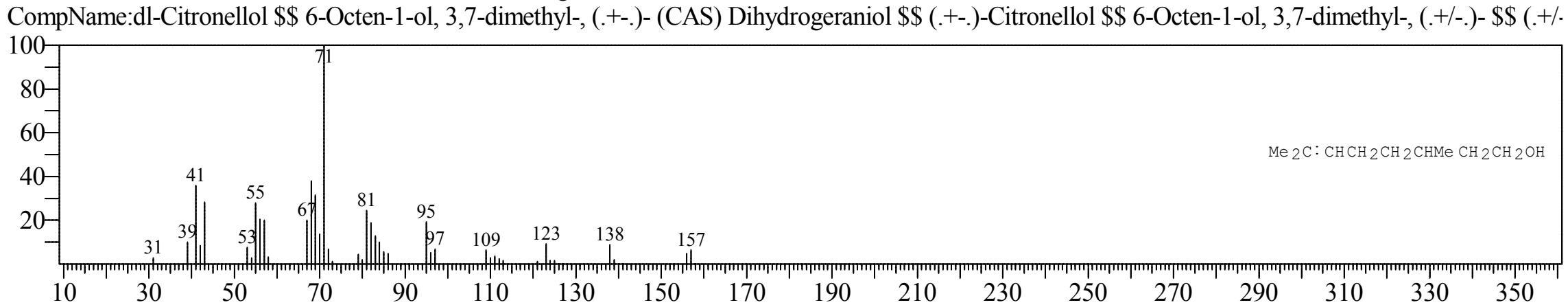

Hit#:17 Entry:45562 Library:WILEY7.LIB
SI:86 Formula:C10 H20 O CAS:2216-52-6 MolWeight:156 RetIndex:0
CompName:Cyclohexanol, 5-methyl-2-(1-methylethyl)-, [1S-(1.alpha.,2.alpha.,5.beta.)]- $$ (1S, 2S, 5R)-(+)-Neomenthol $$ NEOMENTHOL, CYCLOHE

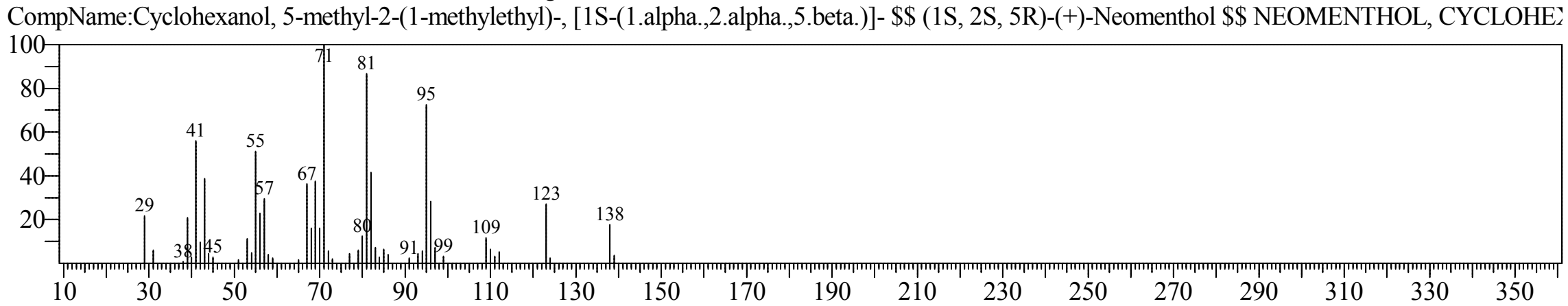

Hit#:18 Entry:10351 Library:NIST11s.lib
SI:85 Formula:C10H20O CAS:106-22-9 MolWeight:156 RetIndex:1179
CompName:Citronellol $$ 6-Octen-1-ol, 3,7-dimethyl- $$ .beta.-Citronellol $$ Cephrol $$ Rodinol $$ 3,7-Dimethyl-6-octen-1-ol $$ Elenol $$ Rhodinol $$

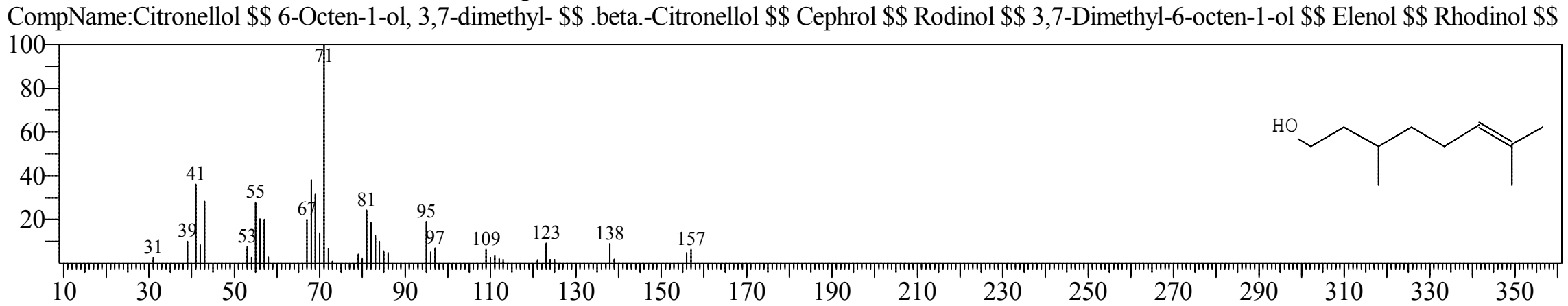

Hit#:19 Entry:18037 Library:NIST05.LIB
SI:85 Formula:C10H20O CAS:15356-70-4 MolWeight:156 RetIndex:1164
CompName:Cyclohexanol, 5-methyl-2-(1-methylethyl)-, (1.alpha.,2.beta.,5.alpha.)-(.+/-.)- $$ Menthol, (.+/-.)- $$ (.+/-.)-Menthol $$ DL-Menthol $$ Mentho

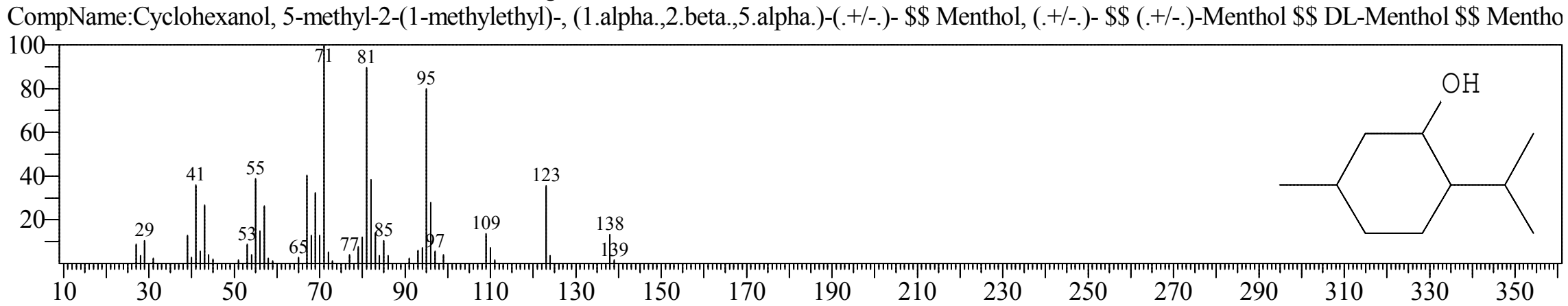

Hit#:20 Entry:14107 Library:NIST05s.LIB
SI:85 Formula:C12H24O CAS:2855-19-8 MolWeight:184 RetIndex:1304
CompName:Oxirane, decyl- $$ Dodecane, 1,2-epoxy- $$ Nedox 1200 $$ 1-Dodecene oxide $$ 1,2-Dodecane oxide $$ 1,2-Dodecene oxide $$ 1,2-Epoxy-n

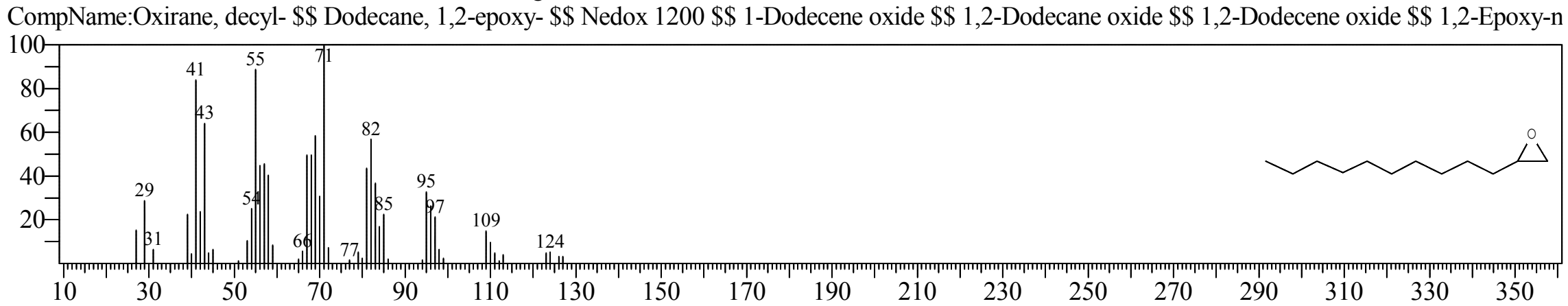

<< Target >>
Line#:21 R.Time:15.708(Scan#:1286) MassPeaks:68
RawMode:Averaged 15.700-15.717(1285-1287) BasePeak:84.10(2793)
BG Mode:Calc. from Peak Group 1 - Event 1 Scan

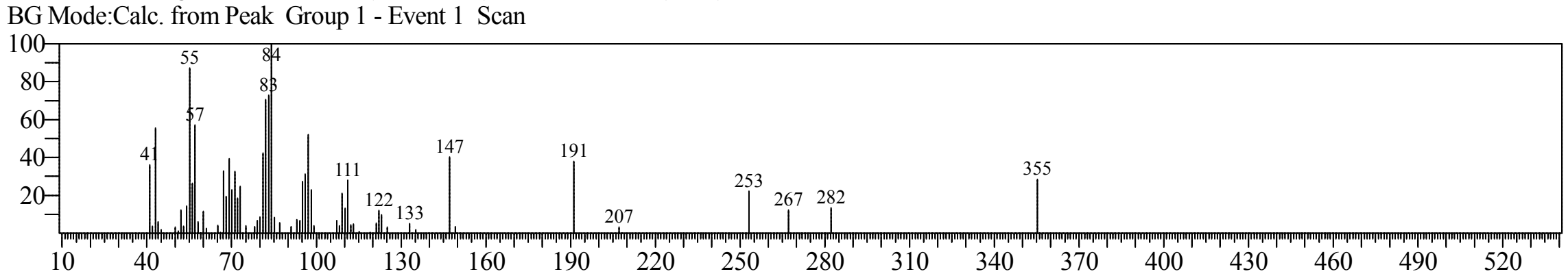

Hit#:1 Entry:80935 Library:NIST05.LIB
SI:78 Formula:C18H34O CAS:2423-10-1 MolWeight:266 RetIndex:2007
CompName:9-Octadecenal, (Z)- $$ Olealdehyde $$ cis-9-Octadecenal $$ Oleylaldehyde $$ Z-9-Octadecenal $$ (9Z)-9-Octadecenal # $$

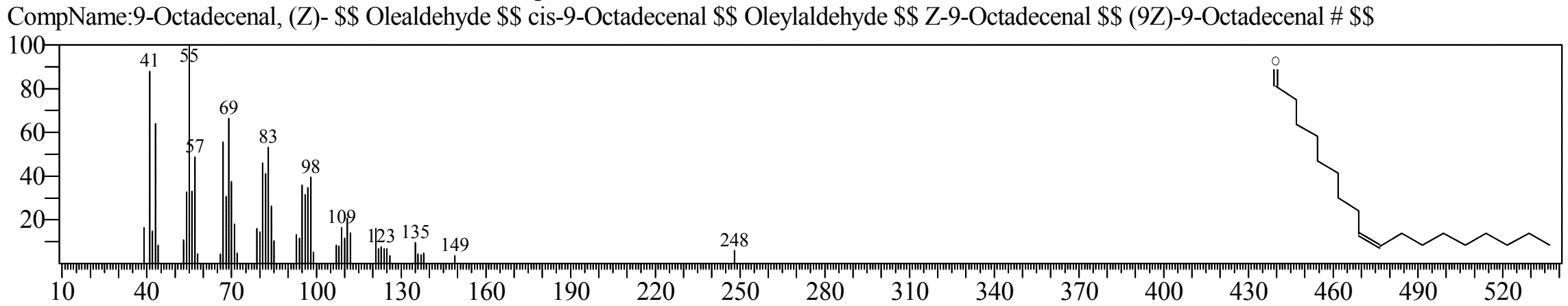

Hit#:2 Entry:72581 Library:NIST05.LIB
SI:77 Formula:C17H32O CAS:60609-53-2 MolWeight:252 RetIndex:1843
CompName:8-Hexadecenal, 14-methyl-, (Z)- $$ 14-Methyl-8-hexadecenal Z; $$ (8Z)-14-Methyl-8-hexadecenal # $$

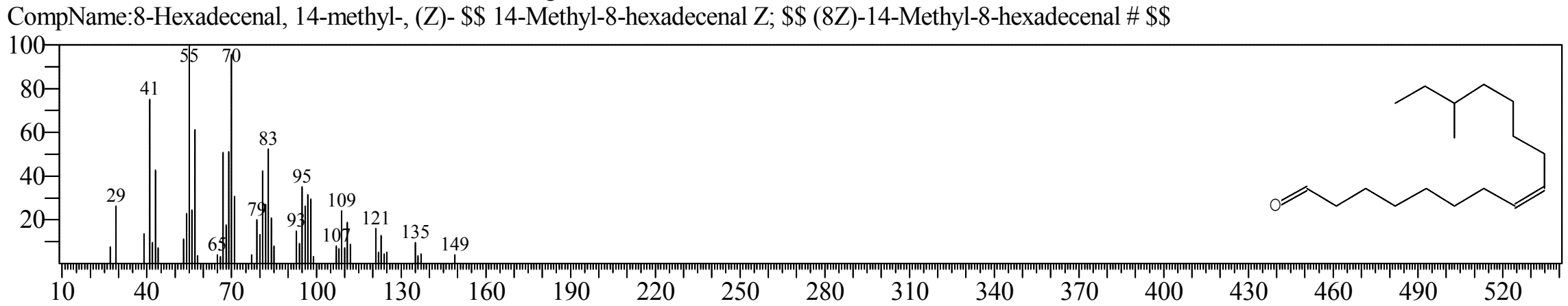

Hit#:3 Entry:164237 Library:NIST11.lib
SI:77 Formula:C20H35F3O2 CAS:0-00-0 MolWeight:364 RetIndex:2019
CompName:Oleyl alcohol, trifluoroacetate

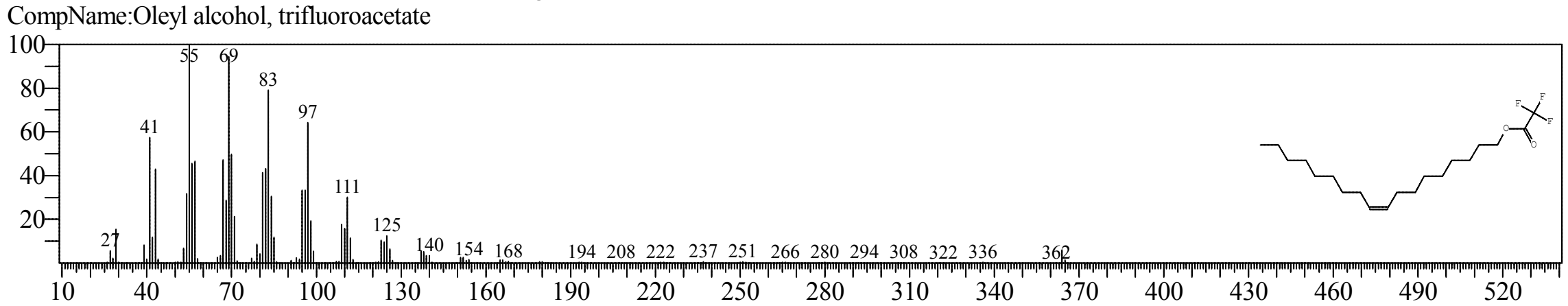

Hit#:4 Entry:133923 Library:NIST05.LIB
SI:77 Formula:C24H46O2 CAS:77899-10-6 MolWeight:366 RetIndex:2679
CompName:(Z)-14-Tricosenyl formate $$ (14Z)-14-Tricosenyl formate # $$

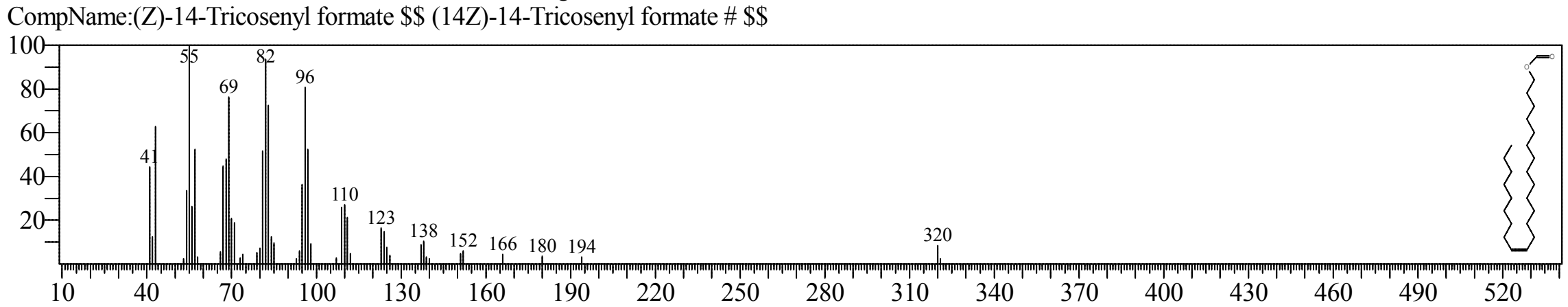

Hit#:5 Entry:321633 Library:WILEY7.LIB
SI:77 Formula:C38 H74 CAS:0-00-0 MolWeight:531 RetIndex:0
CompName:1,37-OCTATRIACONTADIEN $$

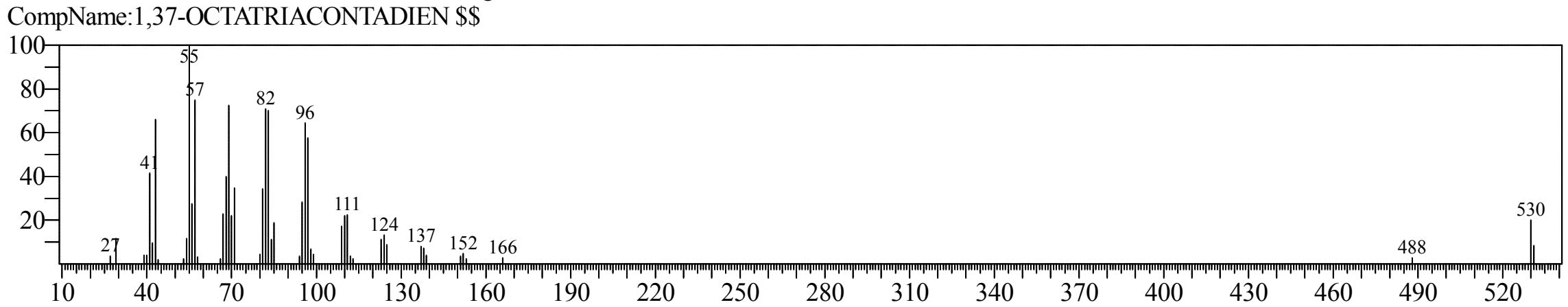

<< Target >>
Line#:21 R.Time:15.708(Scan#:1286) MassPeaks:68
RawMode:Averaged 15.700-15.717(1285-1287) BasePeak:84.10(2793)
BG Mode:Calc. from Peak Group 1 - Event 1 Scan

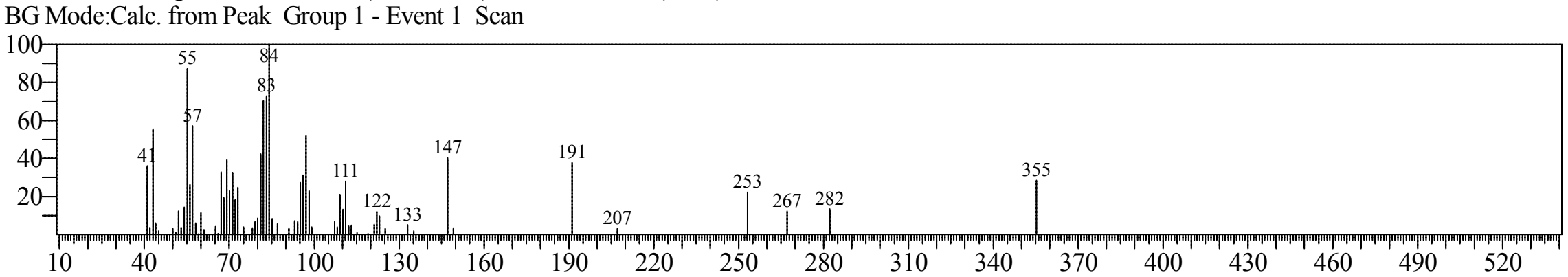

Hit#:6 Entry:207216 Library:NIST11.lib
SI:77 Formula:C38H74 CAS:0-00-0 MolWeight:530 RetIndex:3779
CompName:1,37-Octatriacontadiene

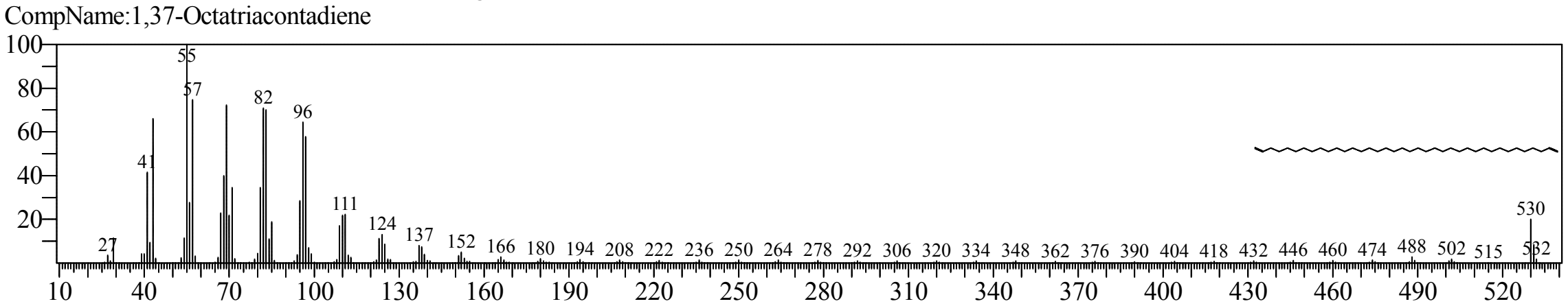

Hit#:7 Entry:158773 Library:NIST05.LIB
SI:77 Formula:C38H74 CAS:0-00-0 MolWeight:530 RetIndex:3779
CompName:1,37-Octatriacontadiene

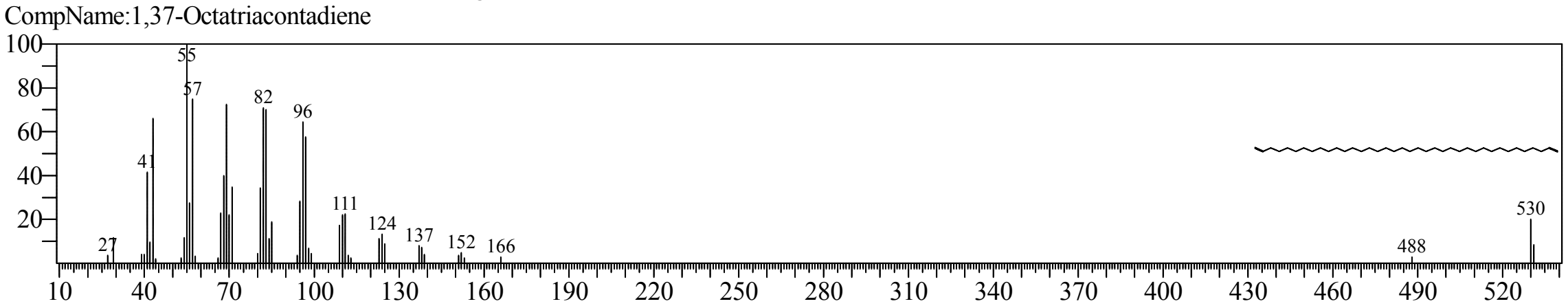

Hit#:8 Entry:73796 Library:NIST05.LIB
SI:77 Formula:C17H34O CAS:30689-78-2 MolWeight:254 RetIndex:1898
CompName:(R)-(-)-(Z)-14-Methyl-8-hexadecen-1-ol $$ (8Z)-14-Methyl-8-hexadecen-1-ol # $$

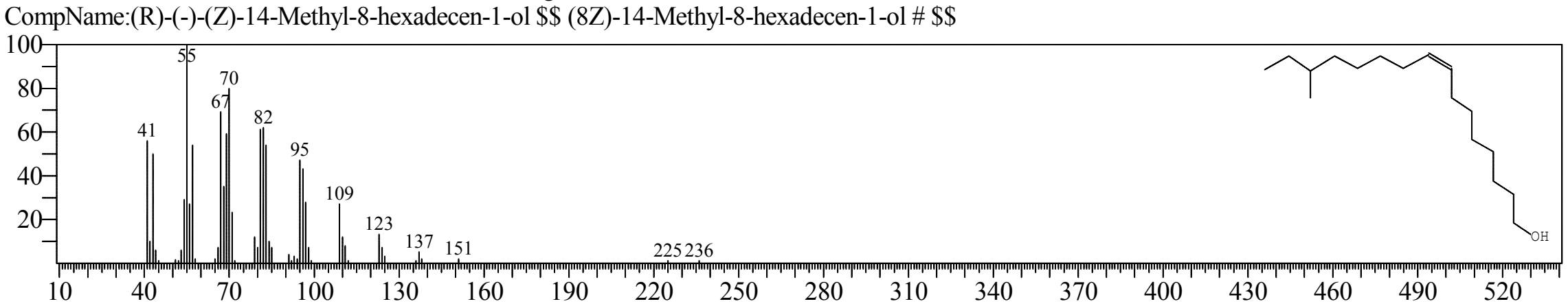

Hit#:9 Entry:153398 Library:NIST05.LIB
SI:77 Formula:C30H62O2 CAS:36645-68-8 MolWeight:454 RetIndex:3489
CompName:1,30-Triacontanediol

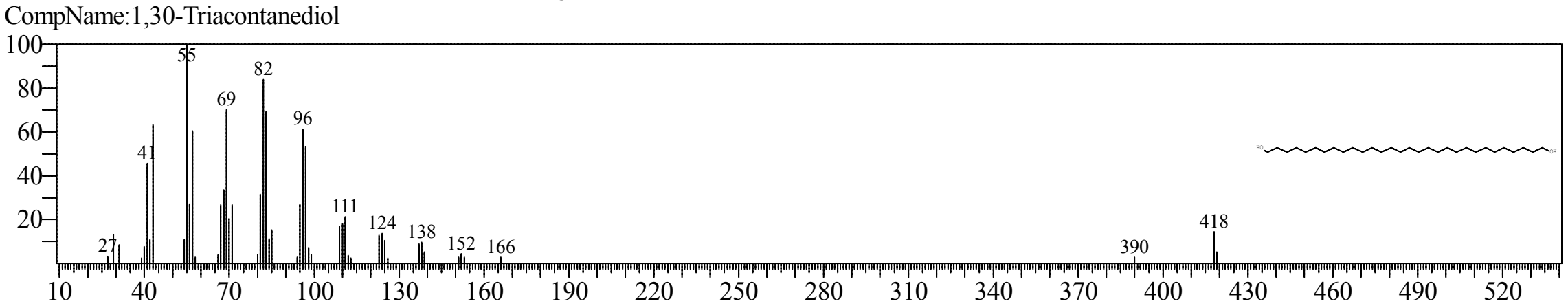

Hit#:10 Entry:22088 Library:NIST05s.LIB
SI:76 Formula:C18H36O CAS:7390-81-0 MolWeight:268 RetIndex:1901
CompName:Oxirane, hexadecyl- $$ 1,2-Epoxyoctadecane $$ 2-Hexadecyloxirane # $$

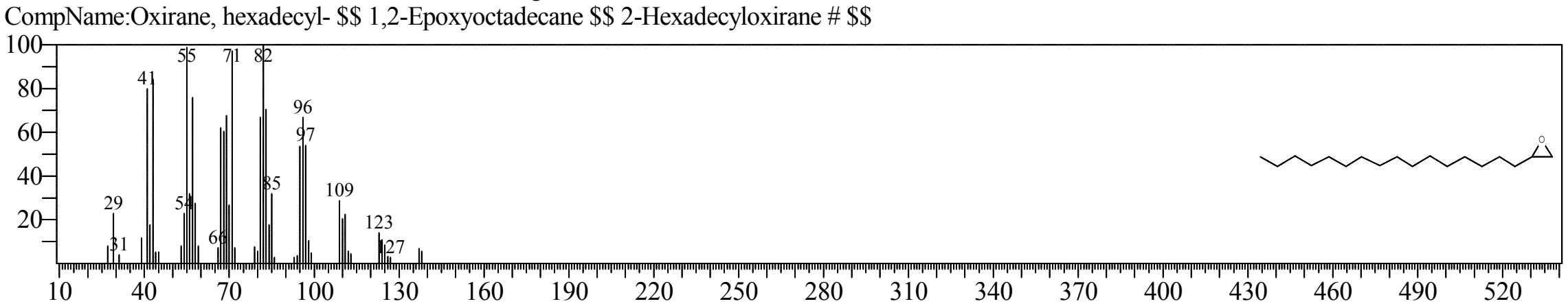

<< Target >>
Line#:21 R.Time:15.708(Scan#:1286) MassPeaks:68
RawMode:Averaged 15.700-15.717(1285-1287) BasePeak:84.10(2793)
BG Mode:Calc. from Peak Group 1 - Event 1 Scan

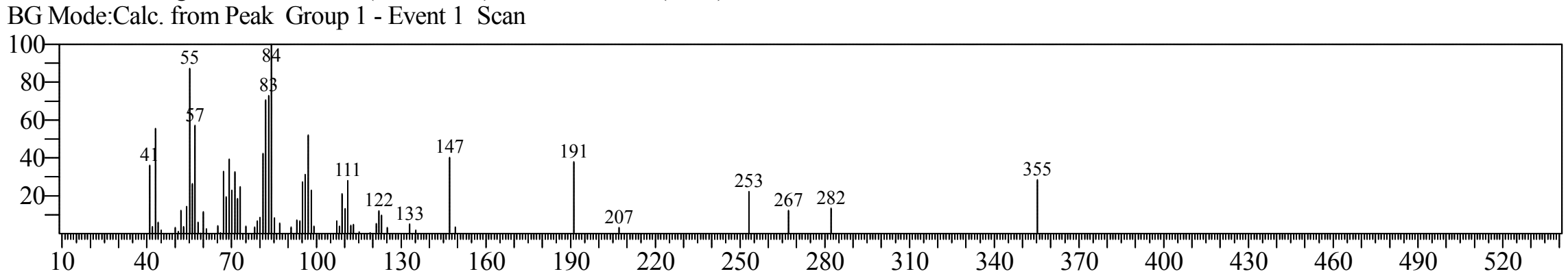

Hit#:11 Entry:175615 Library:WILEY7.LIB
SI:76 Formula:C18 H34 O CAS:56554-95-1 MolWeight:266 RetIndex:0
CompName:11-Octadecenal (spectrum disagrees) (CAS) 11-Octadecenal $$

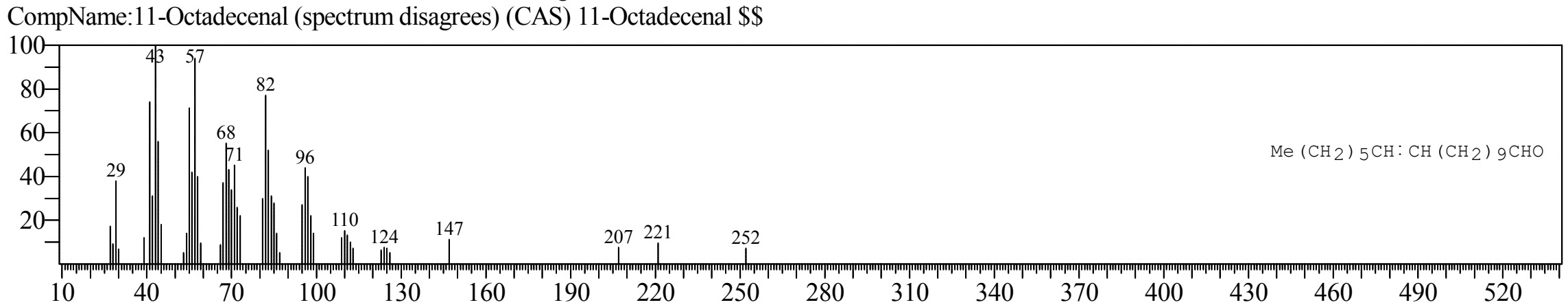

Hit#:12 Entry:41152 Library:NIST05.LIB
SI:76 Formula:C12H24O2 CAS:4422-05-3 MolWeight:200 RetIndex:1815
CompName:cis-1,2-Cyclododecanediol $$ 1,2-Cyclododecanediol # $$

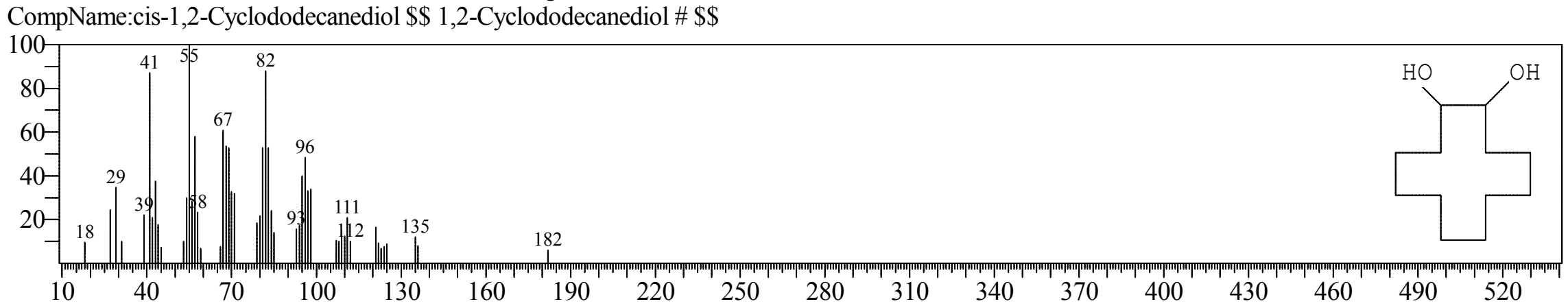

Hit#:13 Entry:90568 Library:NIST05.LIB
SI:76 Formula:C18H34O2 CAS:593-39-5 MolWeight:282 RetIndex:2175
CompName:6-Octadecenoic acid, (Z)- $$ (6Z)-6-Octadecenoic acid # $$

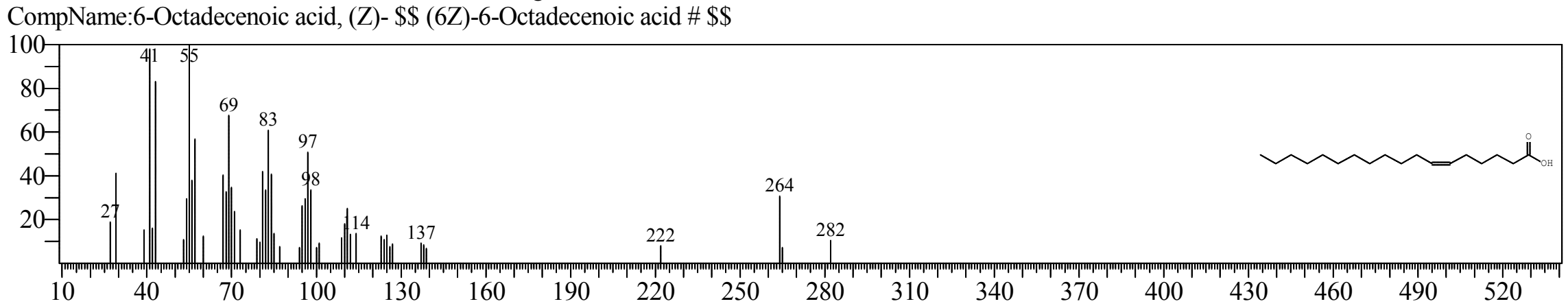

Hit#:14 Entry:47277 Library:NIST05.LIB
SI:76 Formula:C14H26O CAS:85896-31-7 MolWeight:210 RetIndex:1591
CompName:13-Tetradecenal

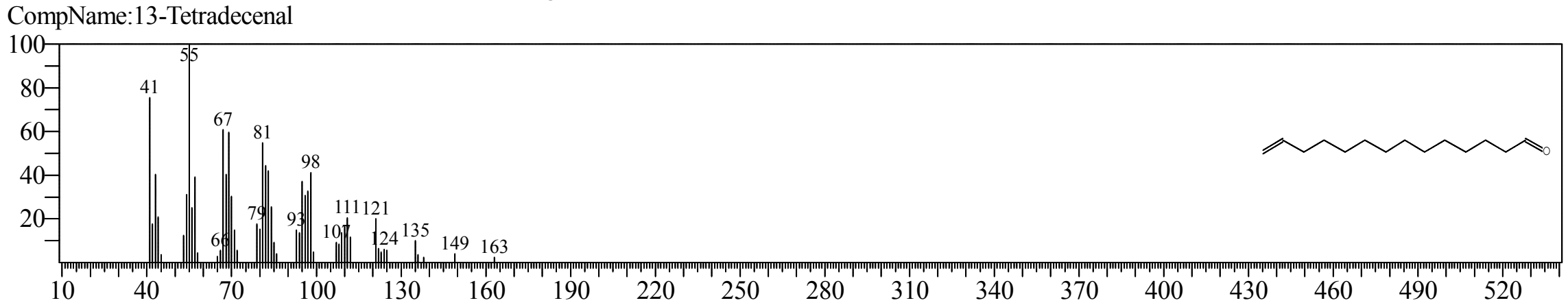

Hit#:15 Entry:21955 Library:NIST05s.LIB
SI:76 Formula:C18H34O CAS:58594-45-9 MolWeight:266 RetIndex:2007
CompName:13-Octadecenal, (Z)- $$ cis-13-Octadecenal $$ Z-13-Octadecenal $$ (13Z)-13-Octadecenal # $$

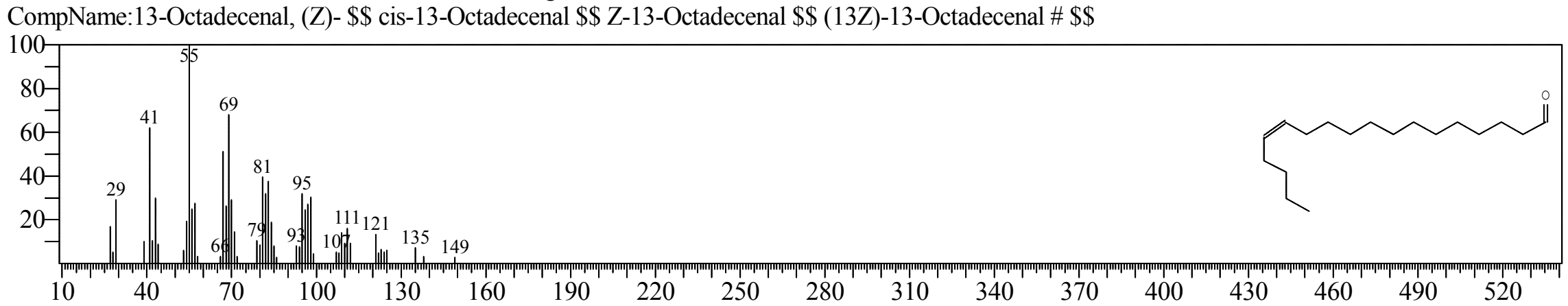

<< Target >>
Line#:21 R.Time:15.708(Scan#:1286) MassPeaks:68
RawMode:Averaged 15.700-15.717(1285-1287) BasePeak:84.10(2793)
BG Mode:Calc. from Peak Group 1 - Event 1 Scan

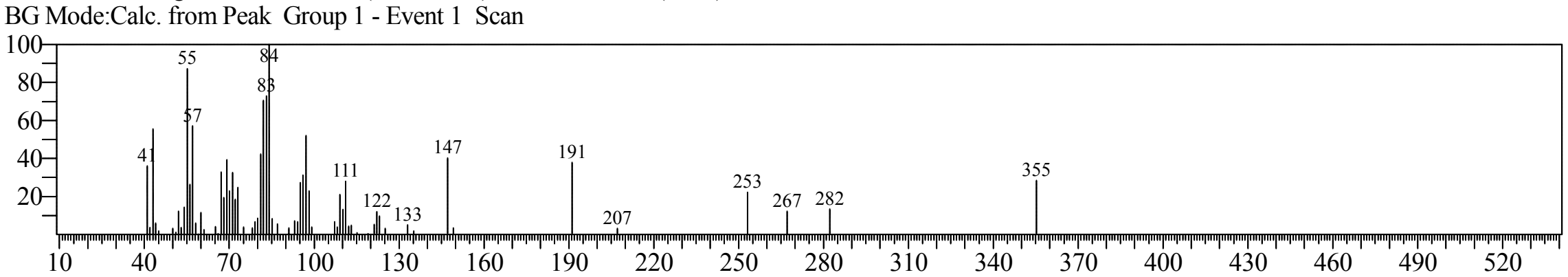

Hit#:16 Entry:20220 Library:NIST05s.LIB
SI:76 Formula:C16H32O CAS:7320-37-8 MolWeight:240 RetIndex:1702
CompName:Oxirane, tetradecyl- $$ Hexadecane, 1,2-epoxy- $$ Hexadecylene oxide $$ 1,2-Epoxyhexadecane $$ 1,2-Hexadecane oxide $$ 1,2-Hexadecene

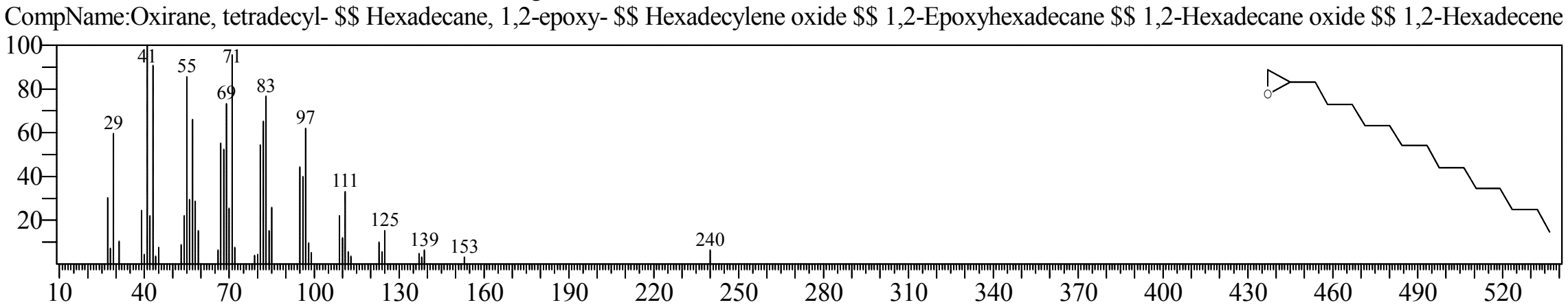

Hit#:17 Entry:80950 Library:NIST05.LIB
SI:76 Formula:C18H34O CAS:56554-90-6 MolWeight:266 RetIndex:2007
CompName:13-Octadecenal $$ (13E)-13-Octadecenal # $$

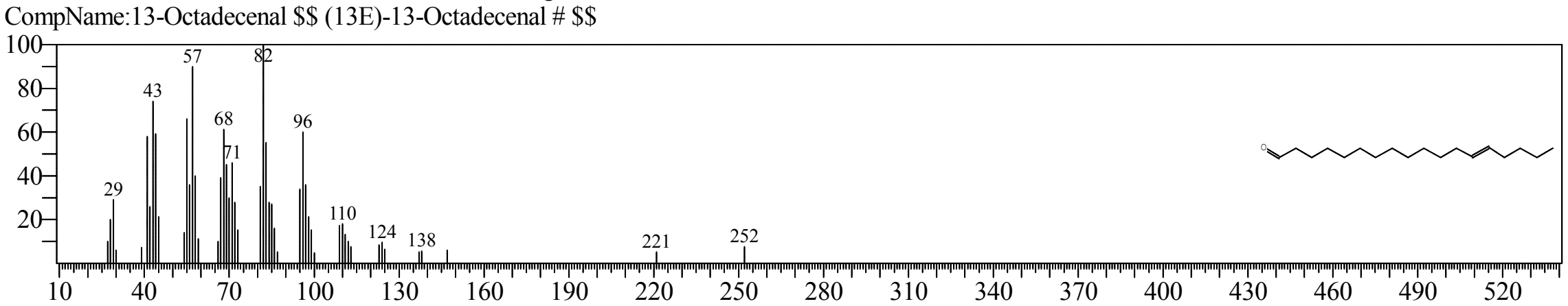

Hit#:18 Entry:57144 Library:NIST05.LIB
SI:76 Formula:C15H30O CAS:2765-11-9 MolWeight:226 RetIndex:1701
CompName:Pentadecanal-

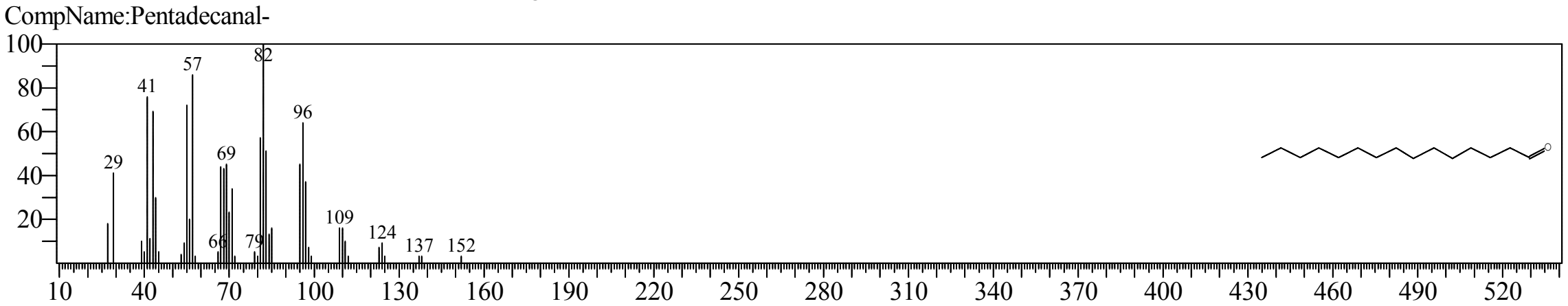

Hit#:19 Entry:80949 Library:NIST05.LIB
SI:76 Formula:C18H34O CAS:56554-89-3 MolWeight:266 RetIndex:2007
CompName:14-Octadecenal $$ (14E)-14-Octadecenal # $$

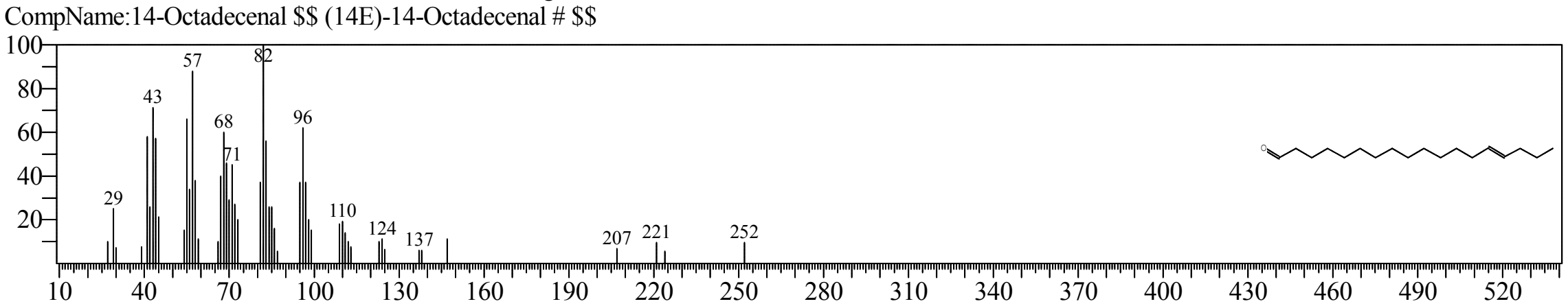

Hit#:20 Entry:330530 Library:WILEY7.LIB
SI:76 Formula:C39 H72 O5 CAS:2465-32-9 MolWeight:621 RetIndex:0
CompName:DI-(9-OCTADECENOYL)-GLYCEROL $$ 9-OCTADECENOIC ACID (Z)-, 2-HYDROXY-1,3-PROPANEDIYL ESTER $$

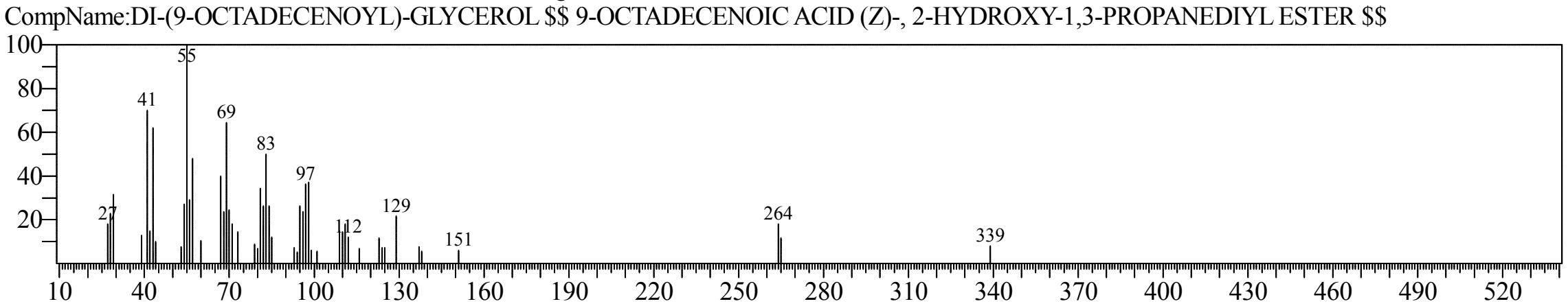

<< Target >>
Line#:22 R.Time:15.942(Scan#:1314) MassPeaks:75
RawMode:Averaged 15.933-15.950(1313-1315) BasePeak:40.00(3888)
BG Mode:Calc. from Peak Group 1 - Event 1 Scan

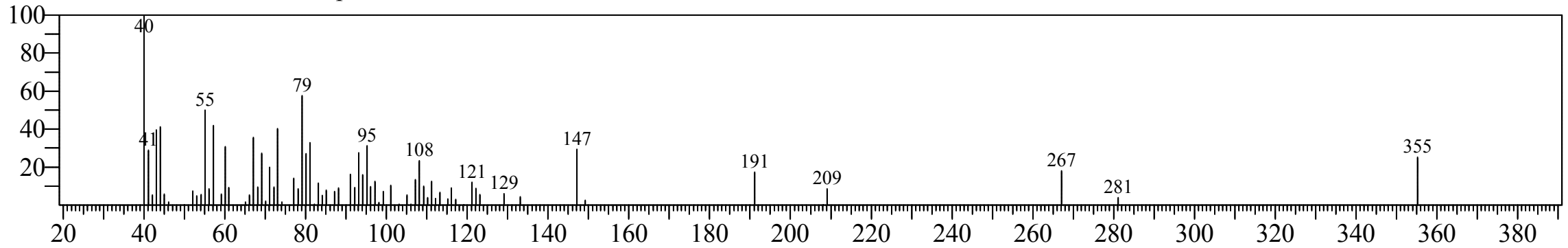

Hit#:1 Entry:78569 Library:NIST05.LIB
SI:68 Formula:C18H30O CAS:26537-71-3 MolWeight:262 RetIndex:2023
CompName:9,12,15-Octadecatrienal $$ (9E,12E,15E)-9,12,15-Octadecatrienal # $$

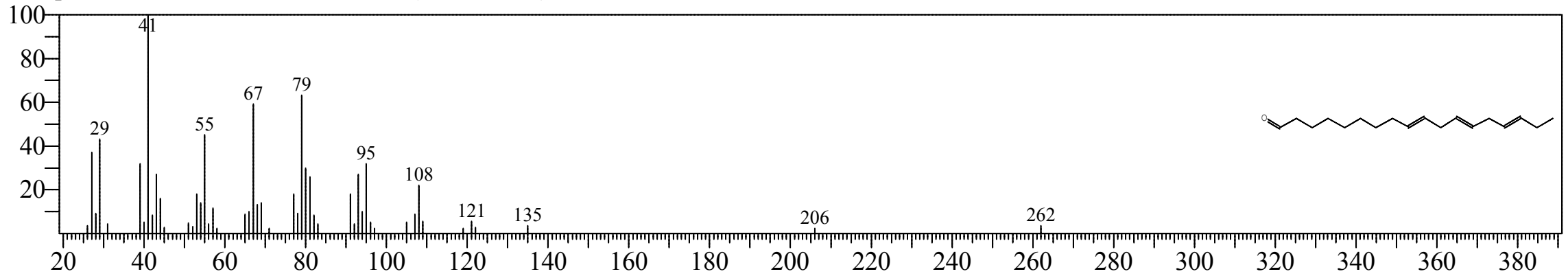

Hit#:2 Entry:141002 Library:NIST05.LIB
SI:68 Formula:C27H46O CAS:6079-19-2 MolWeight:386 RetIndex:2421
CompName:Cholestane, 4,5-epoxy-, (4.alpha.,5.alpha.)- $$ 5.alpha.-Cholestane, 4.alpha.,5-epoxy- $$ 4.alpha.,5.alpha.-Epoxycholestane $$ 4.alpha.,5-Epoxy

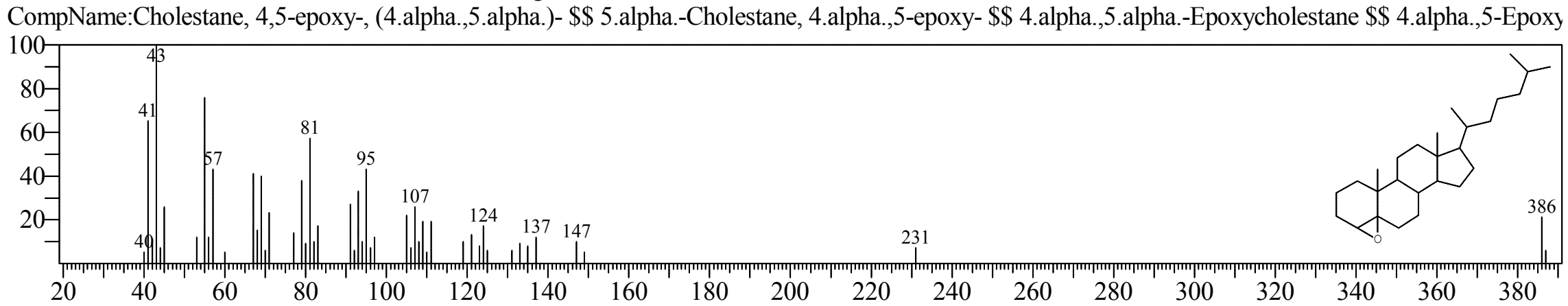

Hit#:3 Entry:88175 Library:NIST05.LIB
SI:68 Formula:C18H30O2 CAS:463-40-1 MolWeight:278 RetIndex:2191
CompName:9,12,15-Octadecatrienoic acid, (Z,Z,Z)- $$ Linolenic acid $$ .alpha.-Linolenic acid $$ All-cis-9,12,15-Octadecatrienoic acid $$ cis,cis,cis-9,12,

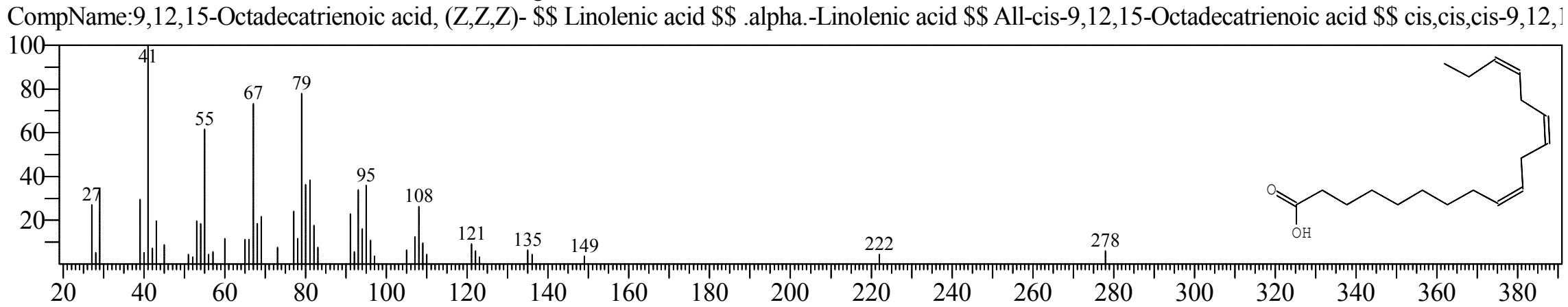

Hit#:4 Entry:123914 Library:WILEY7.LIB
SI:68 Formula:C15 H26 O CAS:142-50-7 MolWeight:222 RetIndex:0
CompName:d-Nerolidol $$ 1,6,10-Dodecatrien-3-ol, 3,7,11-trimethyl-, [S-(Z)]- (CAS) .ALPHA.-3,7,11-TRIMETHYL-1,6,10-DODECATRIEN-3-OL $$ N

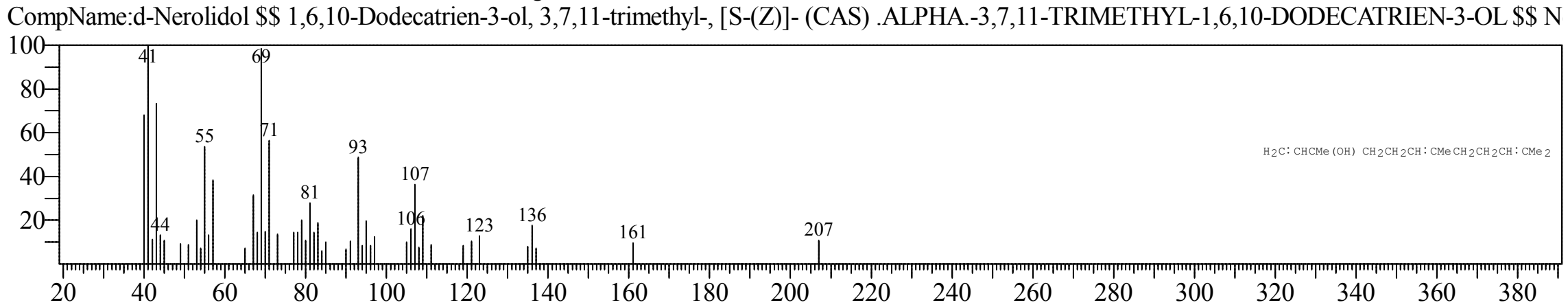

Hit#:5 Entry:9330 Library:NIST05s.LIB
SI:67 Formula:C10H18O CAS:38049-26-2 MolWeight:154 RetIndex:1196
CompName:Cyclohexanol, 2-methyl-5-(1-methylethenyl)-, (1.alpha.,2.beta.,5.alpha.)- $$ Carveol, dihydro- $$ Carveol, dihydro-, cis- $$ Dihydrocarveol $$

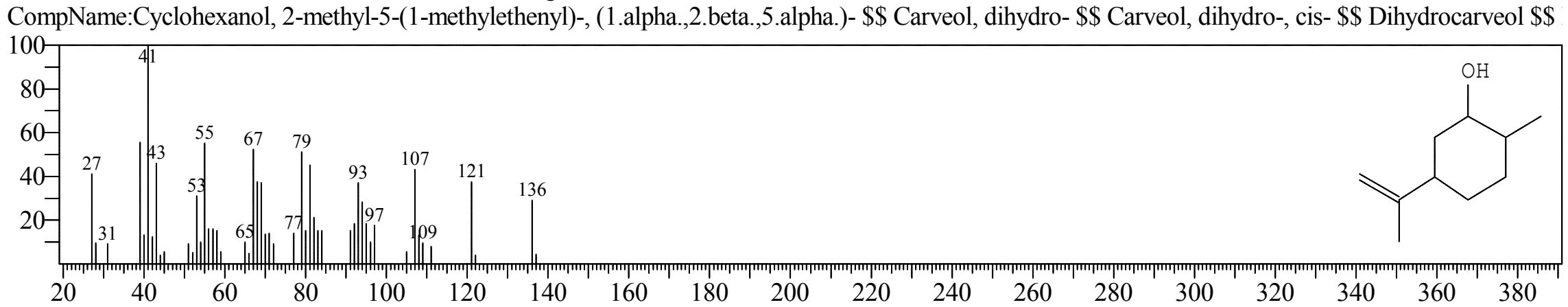

<< Target >>
Line#:22 R.Time:15.942(Scan#:1314) MassPeaks:75
RawMode:Averaged 15.933-15.950(1313-1315) BasePeak:40.00(3888)
BG Mode:Calc. from Peak Group 1 - Event 1 Scan

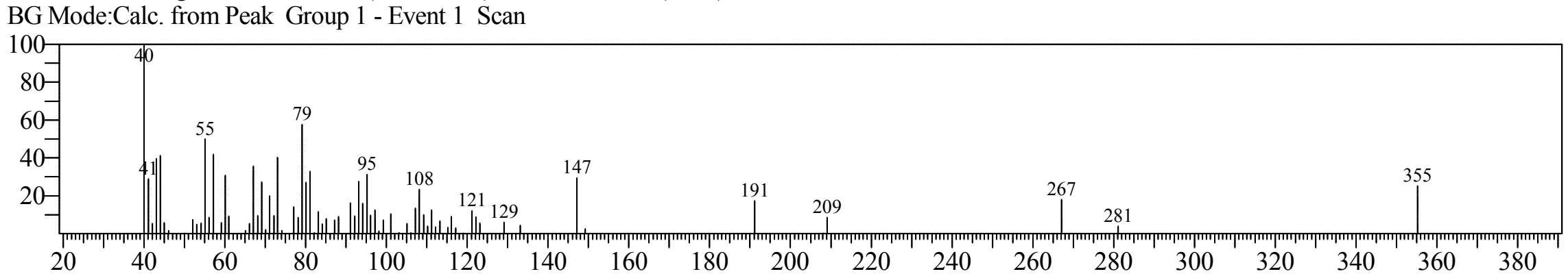

Hit#:6 Entry:9348 Library:NIST05s.LIB
SI:67 Formula:C10H18O CAS:513-23-5 MolWeight:154 RetIndex:1079
CompName:Bicyclo[3.1.0]hexan-3-ol, 4-methyl-1-(1-methylethyl)- $$ Isothujol $$ 1-Isopropyl-4-methylbicyclo[3.1.0]hexan-3-ol # $$

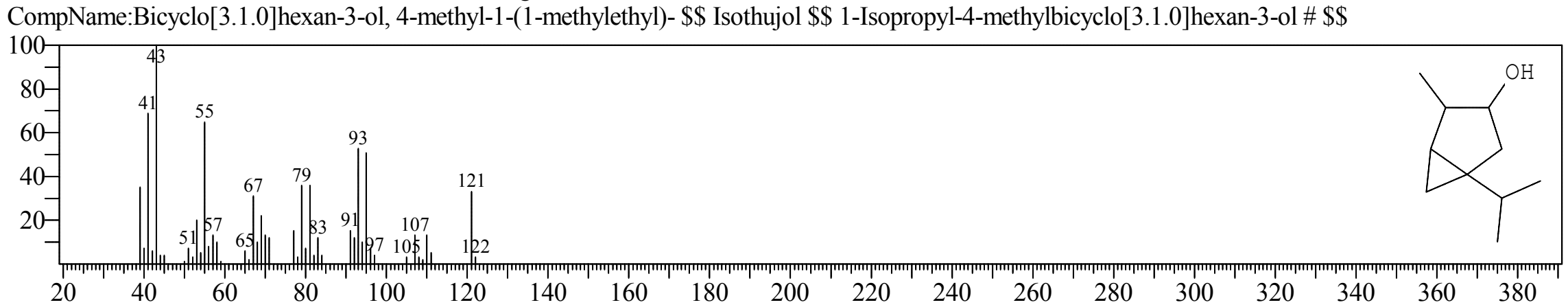

Hit#:7 Entry:112484 Library:NIST05.LIB
SI:67 Formula:C21H36O2 CAS:55682-88-7 MolWeight:320 RetIndex:2300
CompName:11,14,17-Eicosatrienoic acid, methyl ester $$ Methyl (11E,14E,17E)-11,14,17-icosatrienoate # $$

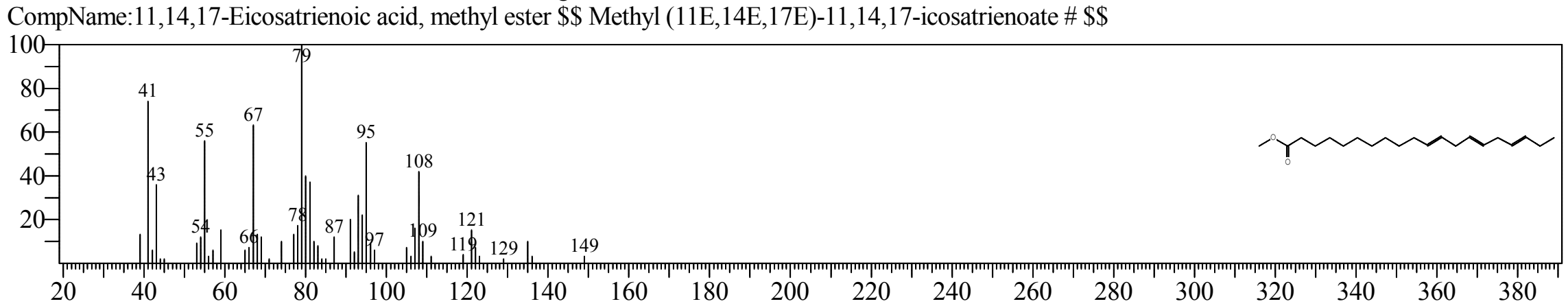

Hit#:8 Entry:61652 Library:NIST05.LIB
SI:66 Formula:C16H26O CAS:56797-43-4 MolWeight:234 RetIndex:1824
CompName:cis,cis,cis-7,10,13-Hexadecatrienal $$ (7Z,10Z,13Z)-7,10,13-Hexadecatrienal # $$

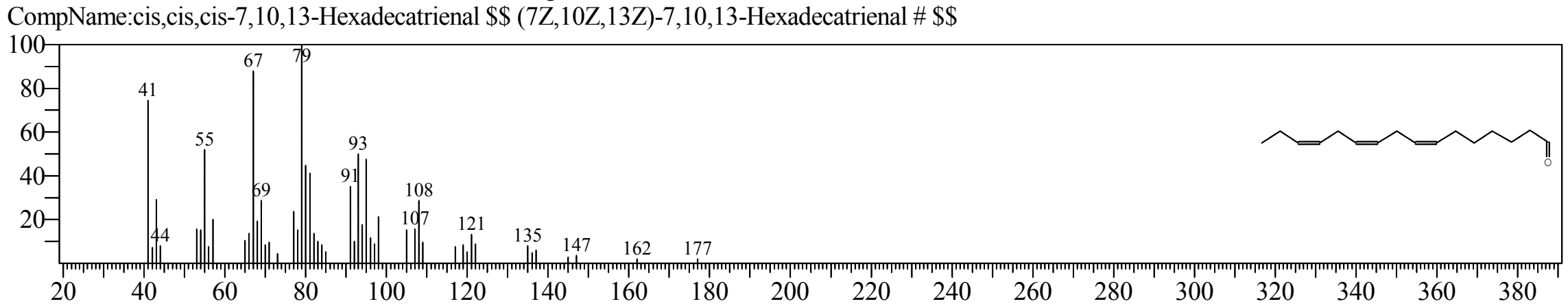

Hit#:9 Entry:203792 Library:WILEY7.LIB
SI:66 Formula:C19 H32 O2 CAS:7361-80-0 MolWeight:292 RetIndex:0
CompName:9,12,15-Octadecatrienoic acid, methyl ester (CAS) Methyl 9,12,15-octadecatrienoate $$

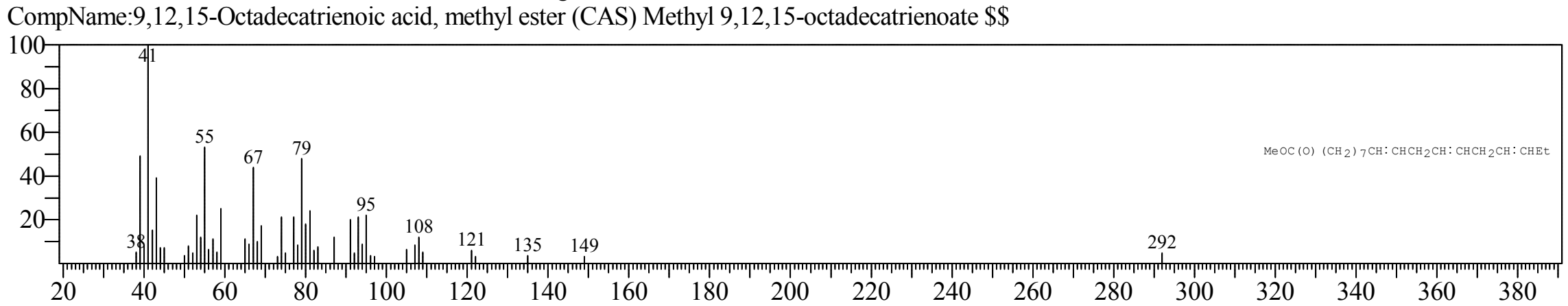

Hit#:10 Entry:119916 Library:NIST05.LIB
SI:66 Formula:C23H42O CAS:0-00-0 MolWeight:334 RetIndex:2539
CompName:1-Heptadec-1-ynyl-cyclohexanol

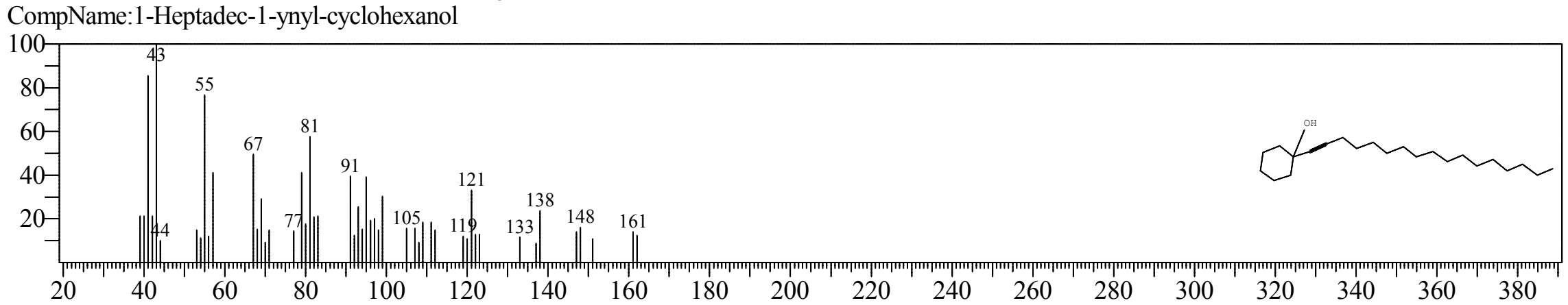

<< Target >>
Line#:22 R.Time:15.942(Scan#:1314) MassPeaks:75
RawMode:Averaged 15.933-15.950(1313-1315) BasePeak:40.00(3888)
BG Mode:Calc. from Peak Group 1 - Event 1 Scan

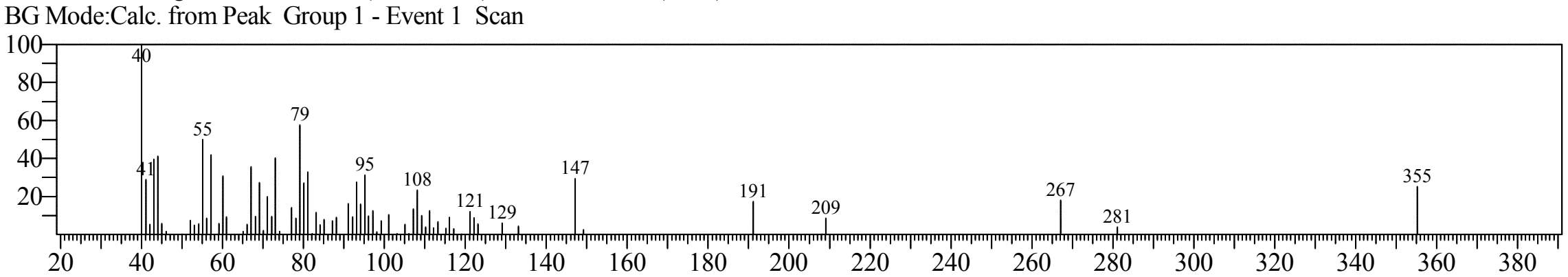

Hit#:11 Entry:9443 Library:NIST05s.LIB
SI:66 Formula:C10H18O CAS:619-01-2 MolWeight:154 RetIndex:1196
CompName:Cyclohexanol, 2-methyl-5-(1-methylethenyl)- $$ p-Menth-8-en-2-ol $$ 1,6-Dihydrocarveol $$ 6-Methyl-3-isopropenylcyclohexanol $$ 8-p-Men

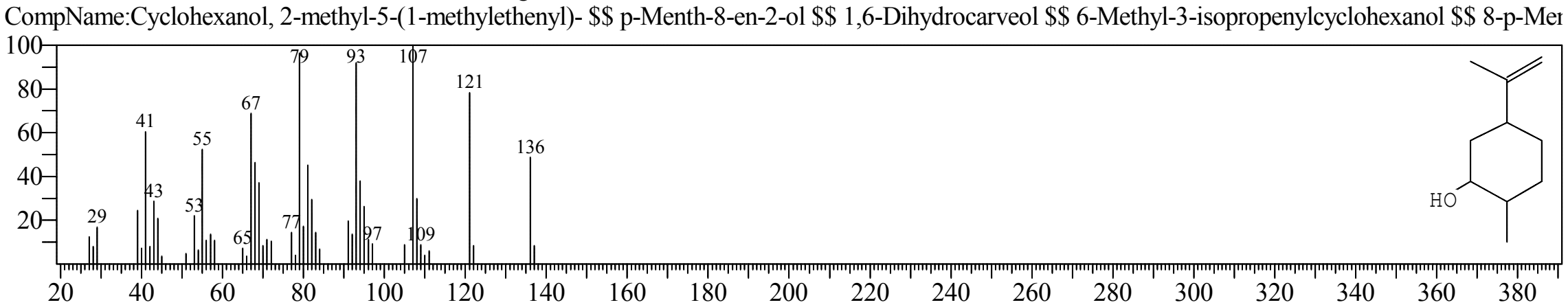

Hit#:12 Entry:144988 Library:NIST11.lib
SI:66 Formula:C23H42O CAS:0-00-0 MolWeight:334 RetIndex:2539
CompName:1-Heptadec-1-ynyl-cyclohexanol $$ 1-(1-Heptadecynyl)cyclohexanol # $$

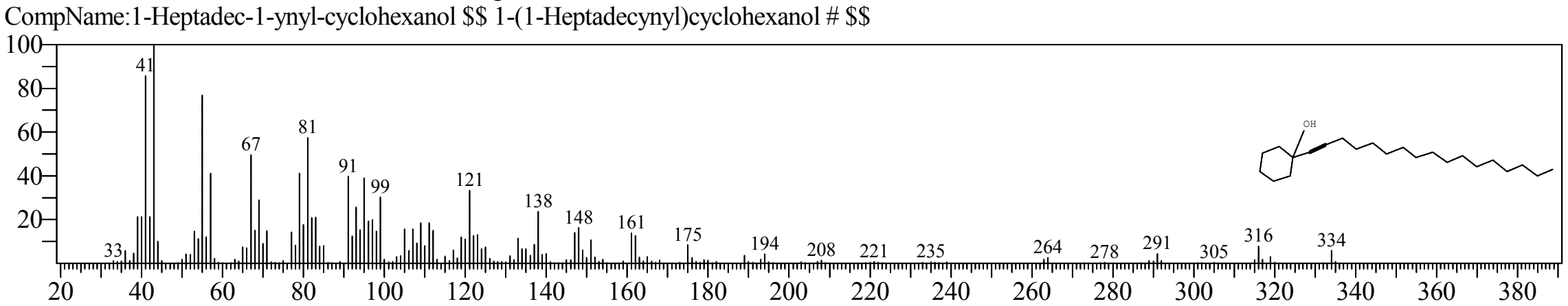

Hit#:13 Entry:112476 Library:NIST05.LIB
SI:66 Formula:C21H36O2 CAS:21061-10-9 MolWeight:320 RetIndex:2300
CompName:8,11,14-Eicosatrienoic acid, methyl ester, (Z,Z,Z)- $$ Methyl dihomo-.gamma.-linolenate $$ Methyl eicosa-8,11,14-trienoate $$ Methyl (8Z,11Z

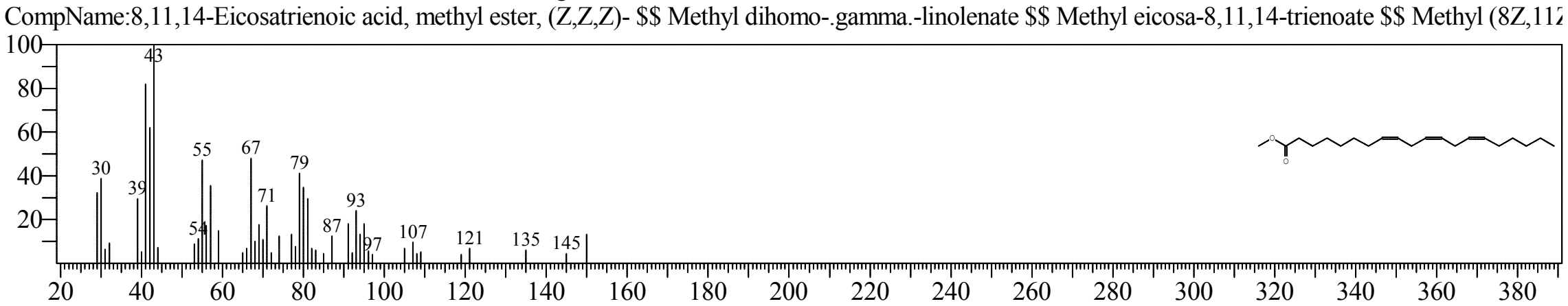

Hit#:14 Entry:230725 Library:WILEY7.LIB
SI:66 Formula:C21 H36 O2 CAS:17364-32-8 MolWeight:320 RetIndex:0
CompName:8,11,14-Eicosatrienoic acid, methyl ester (CAS) METHYL 8,11,14-EICOSATRIENOATE $$

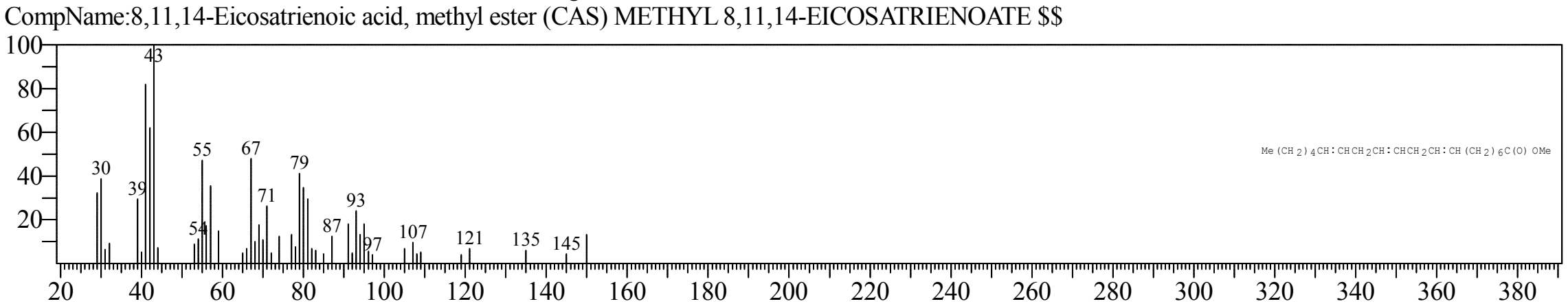

Hit#:15 Entry:36588 Library:NIST05.LIB
SI:66 Formula:C14H24 CAS:74752-91-3 MolWeight:192 RetIndex:1420
CompName:1-Tetradecen-3-yne

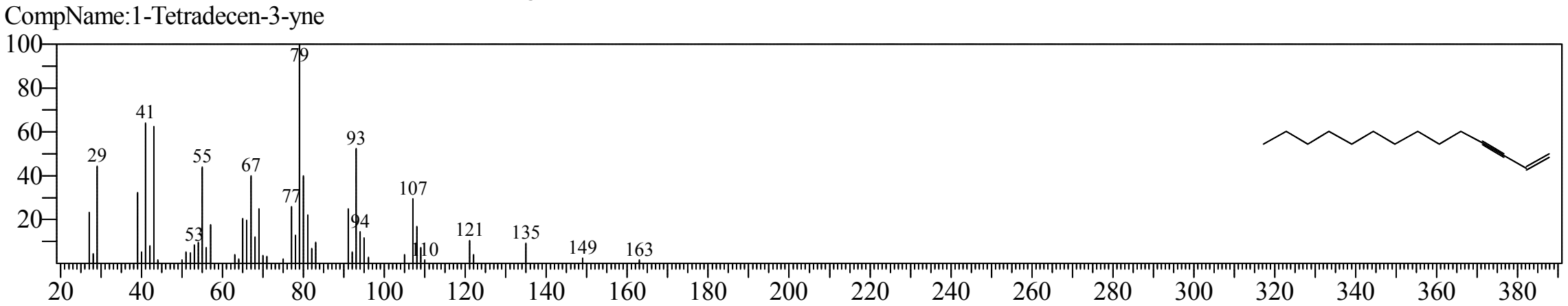

<< Target >>
Line#:22 R.Time:15.942(Scan#:1314) MassPeaks:75
RawMode:Averaged 15.933-15.950(1313-1315) BasePeak:40.00(3888)
BG Mode:Calc. from Peak Group 1 - Event 1 Scan

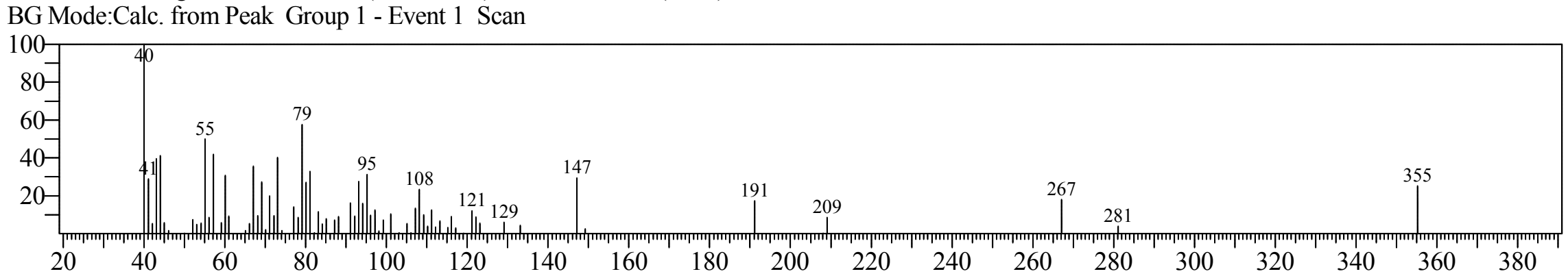

Hit#:16 Entry:23472 Library:NIST05s.LIB
SI:66 Formula:C19H34O2 CAS:56630-74-1 MolWeight:294 RetIndex:2093
CompName:5,8-Octadecadienoic acid, methyl ester $$ Methyl (5E,8E)-5,8-octadecadienoate # $$

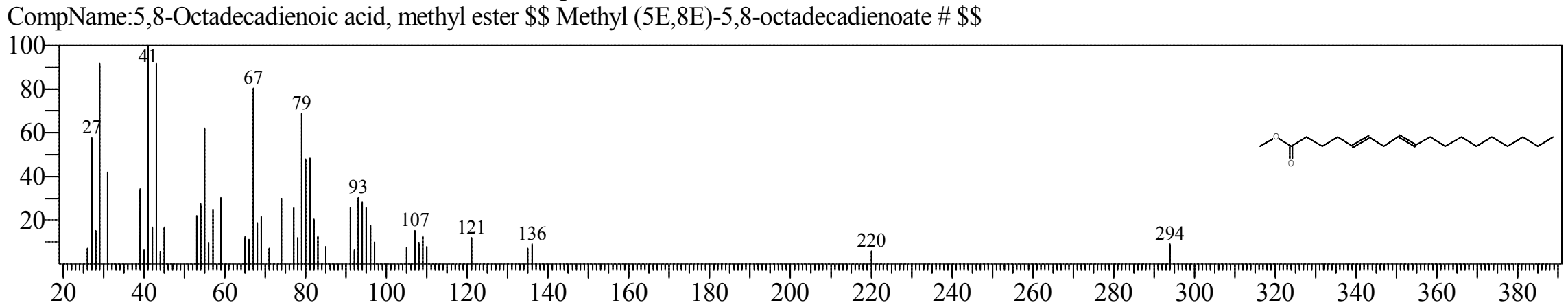

Hit#:17 Entry:21637 Library:NIST05.LIB
SI:66 Formula:C12H20 CAS:0-00-0 MolWeight:164 RetIndex:1227
CompName:1,5-Cyclooctadiene, 1-t-butyl-

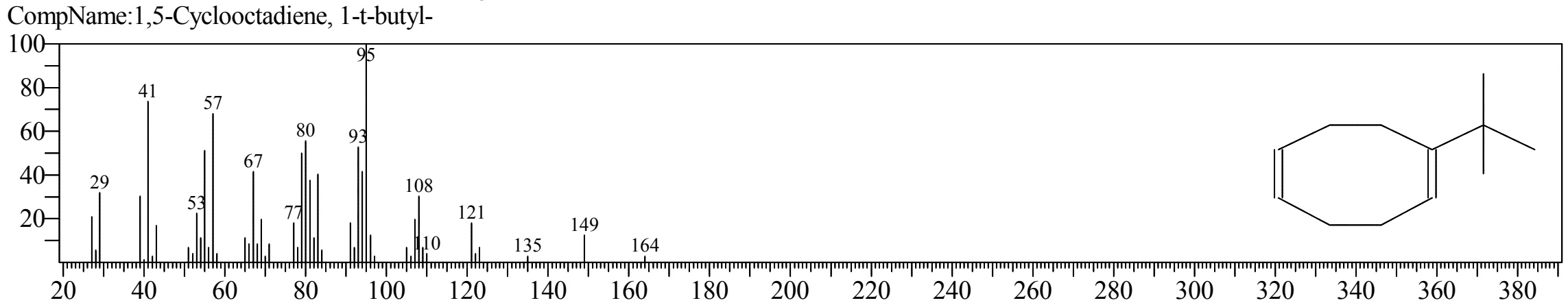

Hit#:18 Entry:31096 Library:NIST05.LIB
SI:66 Formula:C11H18O2 CAS:62555-02-6 MolWeight:182 RetIndex:1204
CompName:Bicyclo[2.2.1]heptan-2-ol, 7,7-dimethyl-, acetate $$ 7,7-Dimethylbicyclo[2.2.1]hept-2-yl acetate # $$

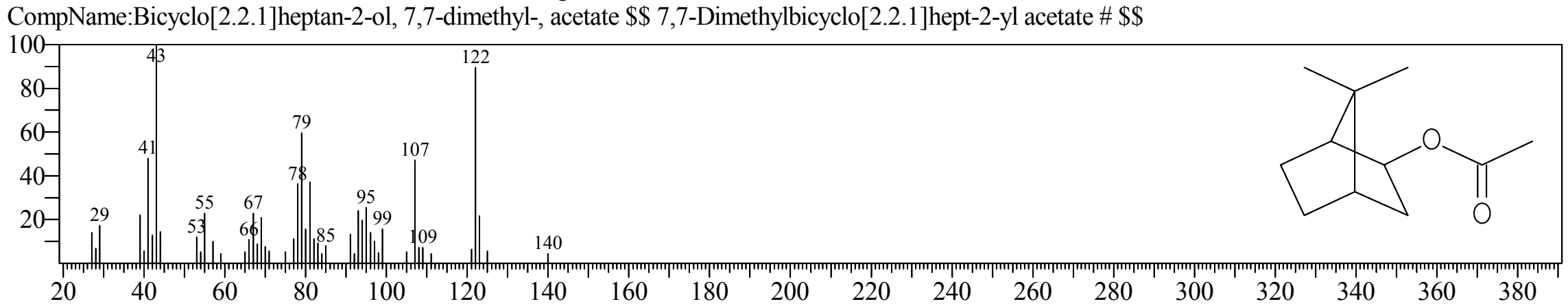

Hit#:19 Entry:29012 Library:NIST05.LIB
SI:66 Formula:C13H22 CAS:74744-41-5 MolWeight:178 RetIndex:1319
CompName:3-Tridecen-1-yne, (E)- $$ (3E)-3-Tridecen-1-yne # $$

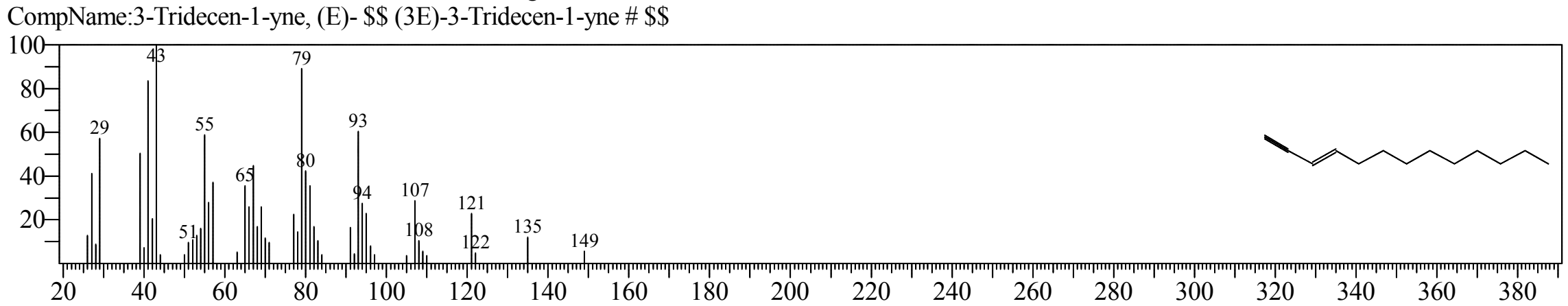

Hit#:20 Entry:22529 Library:NIST11.lib
SI:66 Formula:C12H20 CAS:0-00-0 MolWeight:164 RetIndex:1227
CompName:1,5-Cyclooctadiene, 1-t-butyl- $$ 1-tert-Butyl-1,5-cyclooctadiene # $$

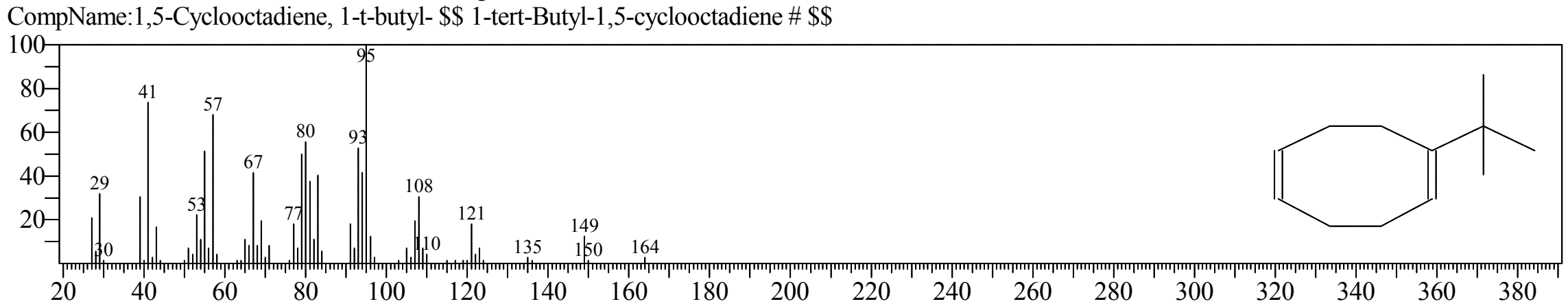

<< Target >>
Line#:23   R.Time:16.000(Scan#:1321) MassPeaks:42
RawMode:Averaged 15.992-16.008(1320-1322) BasePeak:39.95(2006)
BG Mode:Calc. from Peak Group 1 - Event 1 Scan

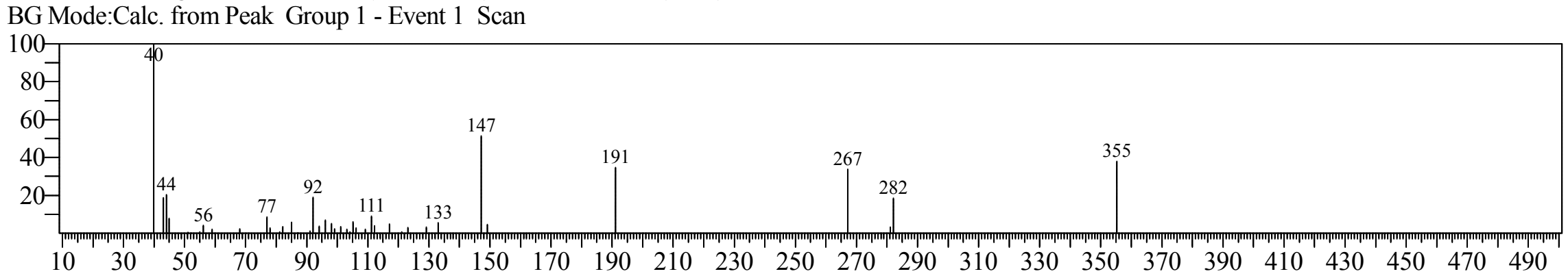

Hit#:1 Entry:83830 Library:WILEY7.LIB
SI:52 Formula:C12 H17 N O CAS:115437-14-4 MolWeight:191 RetIndex:0
CompName:2,2-dimethyl-4-phenylaminobutanone $$ 2-Butanone, 3,3-dimethyl-4-(phenylamino)- (CAS)

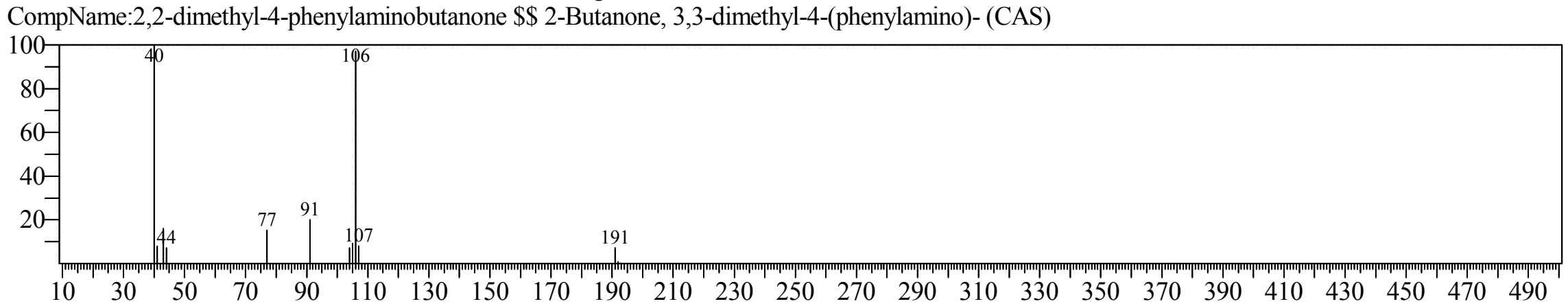

Hit#:2 Entry:257755 Library:WILEY7.LIB
SI:50 Formula:C22 H13 N O4 CAS:0-00-0 MolWeight:355 RetIndex:0
CompName:ETHYL 1-HEXYL-4-HYDROXY-2(1H)-OXO-3-QUINOLINECARBOXYLATE $$ 4-HYDROXY-3-(2-OXO-2H-1-OXA-3-PHENANTHRY

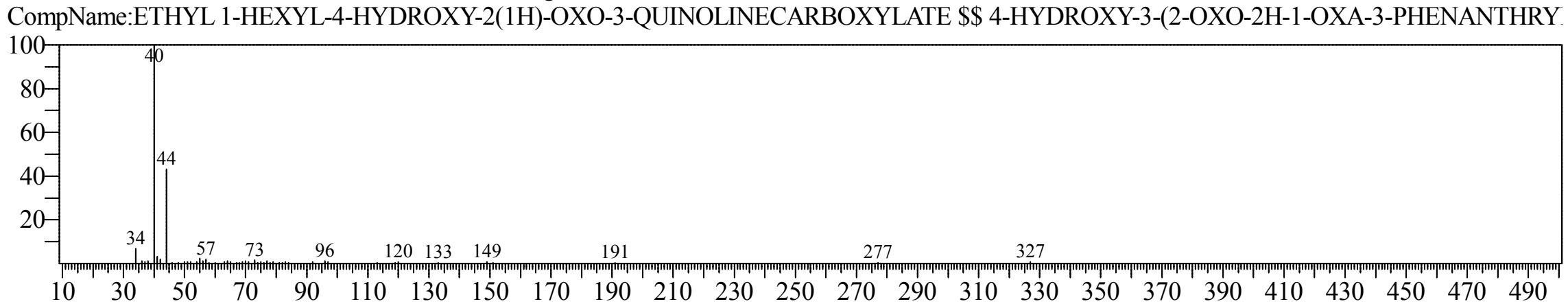

Hit#:3 Entry:98196 Library:WILEY7.LIB
SI:50 Formula:C14 H18 O CAS:33046-68-3 MolWeight:202 RetIndex:0
CompName:3-Octen-2-one, 8-phenyl- (CAS)

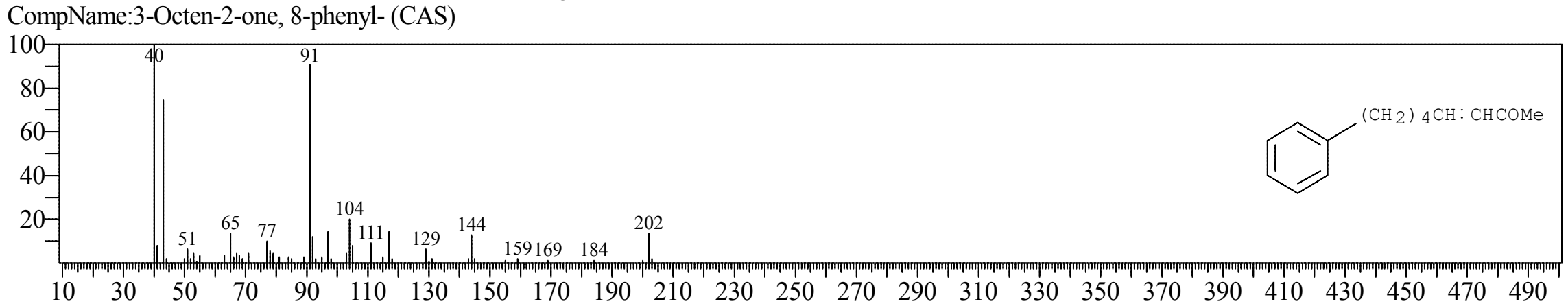

Hit#:4 Entry:160326 Library:WILEY7.LIB
SI:50 Formula:C12 H15 N O5 CAS:108536-10-3 MolWeight:253 RetIndex:0
CompName:pnz-sar $$ Glycine, N-[[(4-methoxyphenyl)methoxy]carbonyl]-N-methyl- (CAS)

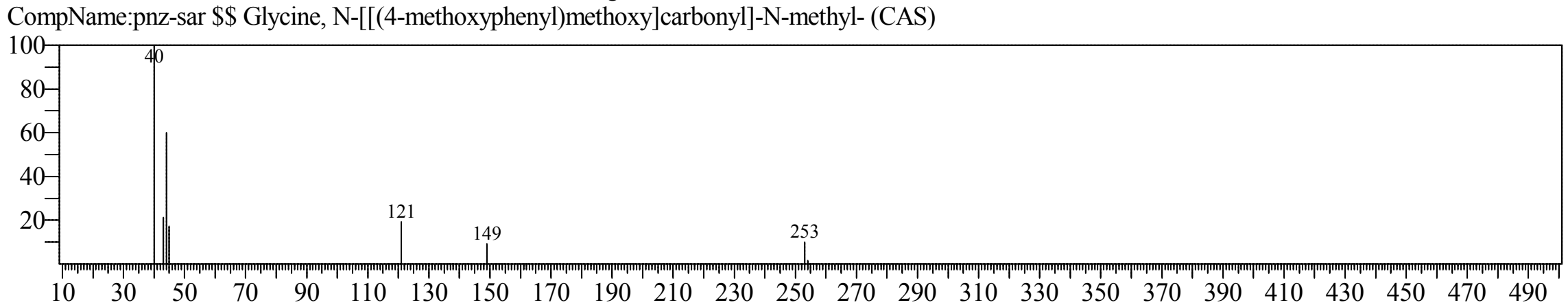

Hit#:5 Entry:37699 Library:NIST11.lib
SI:49 Formula:C8H9N5O CAS:0-00-0 MolWeight:191 RetIndex:2059
CompName:1H-Pyrazole-4-carbonitrile, 5-amino-3-cyanomethyl-1-(2-hydroxyethyl)-

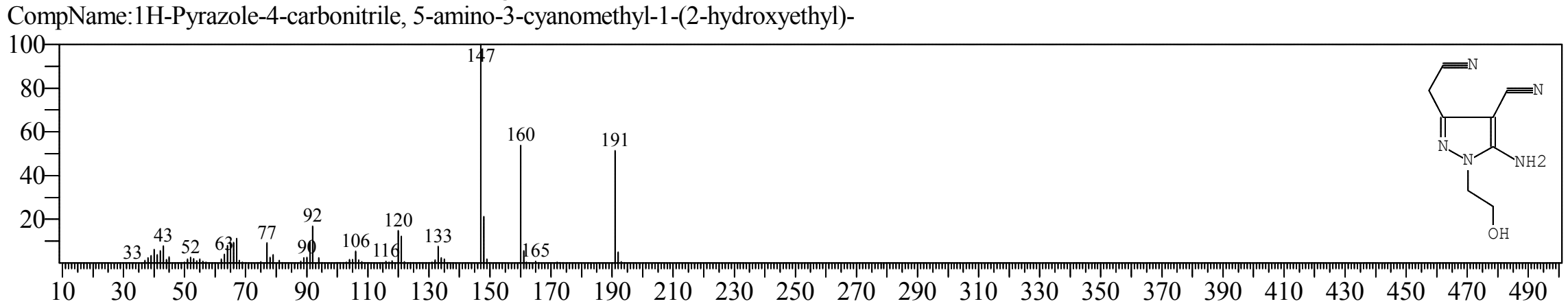

<< Target >>
Line#:23 R.Time:16.000(Scan#:1321) MassPeaks:42
RawMode:Averaged 15.992-16.008(1320-1322) BasePeak:39.95(2006)
BG Mode:Calc. from Peak Group 1 - Event 1 Scan

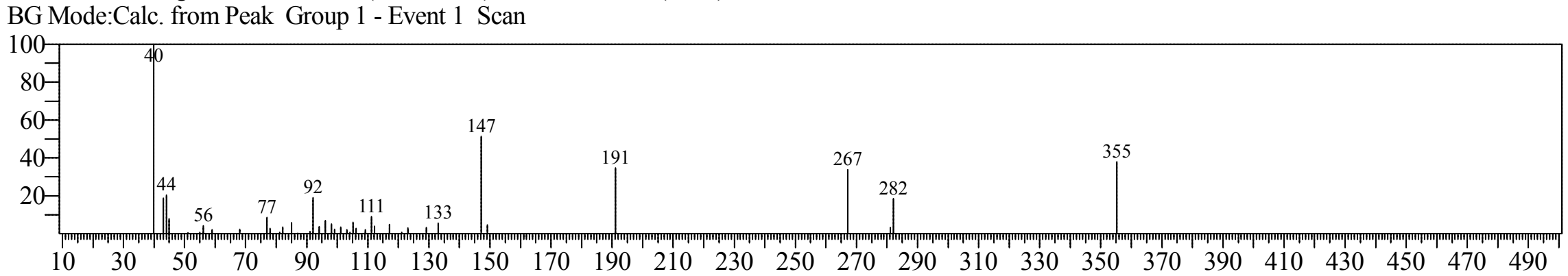

Hit#:6 Entry:35586 Library:NIST05.LIB
SI:49 Formula:C8H9N5O CAS:0-00-0 MolWeight:191 RetIndex:2059
CompName:1H-Pyrazole-4-carbonitrile, 5-amino-3-cyanomethyl-1-(2-hydroxyethyl)-

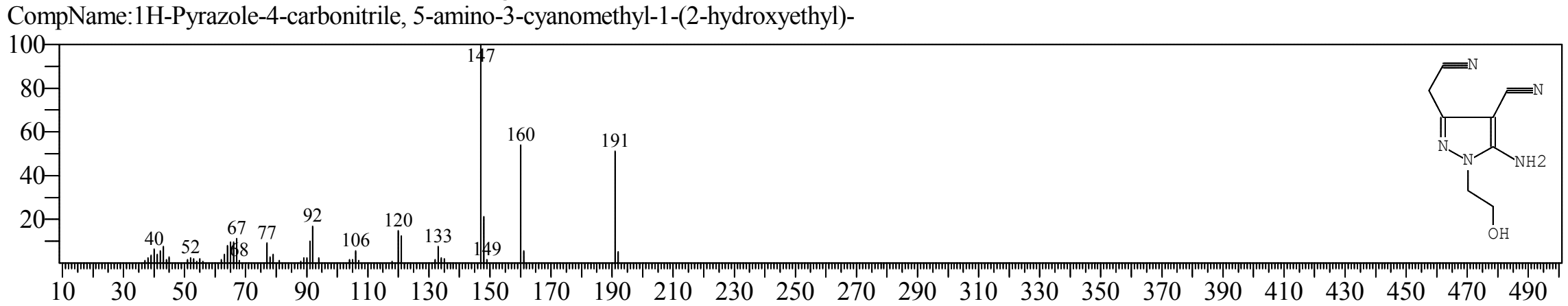

Hit#:7 Entry:250539 Library:WILEY7.LIB
SI:49 Formula:C15 H12 BR N3 O2 CAS:0-00-0 MolWeight:345 RetIndex:0
CompName:N-(P-ANISIDINOMETHYL)-4-METHYLPHTHALIMIDE $$ 4-BROMO-N-[(6-METHYL-2-PYRIDYL)AMINOMETHYL]PHTHALIMIDE

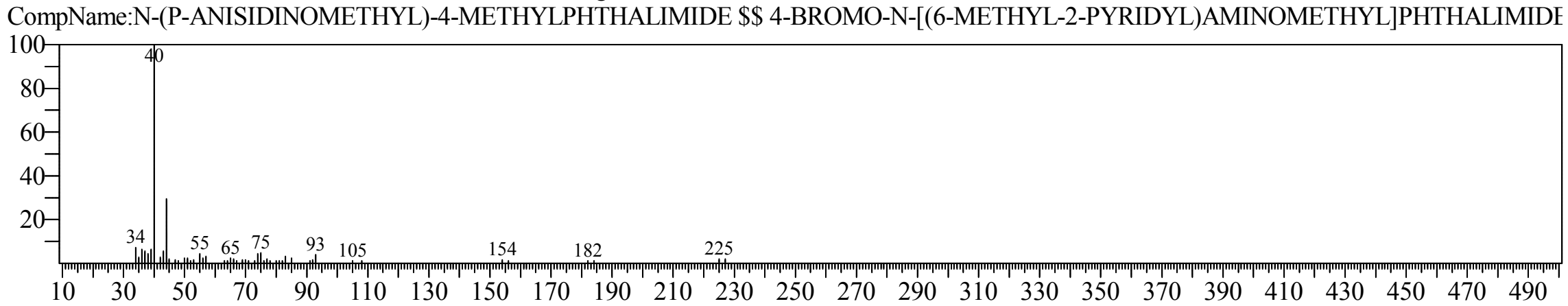

Hit#:8 Entry:321417 Library:WILEY7.LIB
SI:48 Formula:C23 H51 N O3 SI5 CAS:56114-59-1 MolWeight:529 RetIndex:0
CompName:NOREPINEPHRINE-PENTATMS $$ Silanamine, N-[2-[3,4-bis[(trimethylsilyl)oxy]phenyl]-2-[(trimethylsilyl)oxy]ethyl]-1,1,1-trimethyl-N-(tri

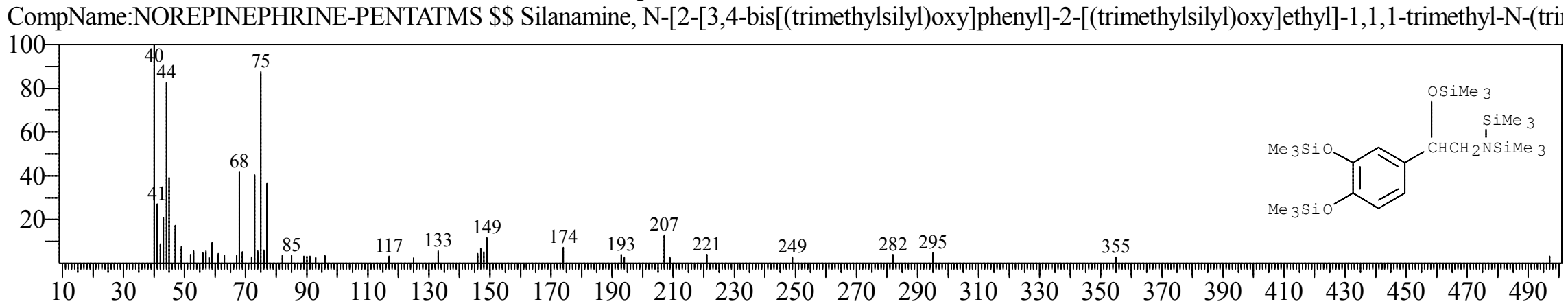

Hit#:9 Entry:264514 Library:WILEY7.LIB
SI:48 Formula:C23 H27 N O3 CAS:0-00-0 MolWeight:365 RetIndex:0
CompName:DIDECYL 1,4-DIHYDRO-2,6-DIMETHYL-3,5-PYRIDINEDICARBOXYLATE $$ 3,4,5,6,9,10-HEXAHYDRO-9-(4-HYDROXYPHENYL)

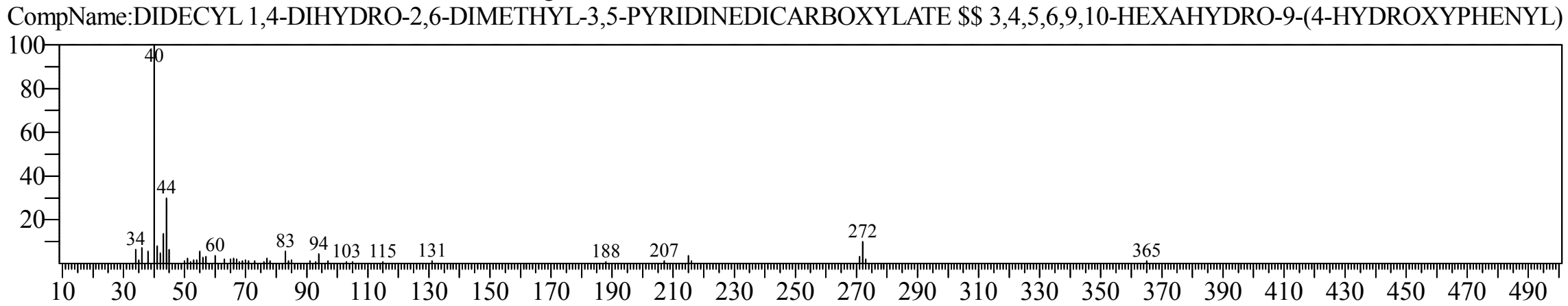

Hit#:10 Entry:35414 Library:NIST05.LIB
SI:48 Formula:C12H18Si CAS:61141-64-8 MolWeight:190 RetIndex:1223
CompName:1H-1-Silaindene, 2,3-dihydro-1-methyl-1-propyl- $$ 1-Methyl-1-propyl-2,3-dihydro-1H-1-benzosilole # $$

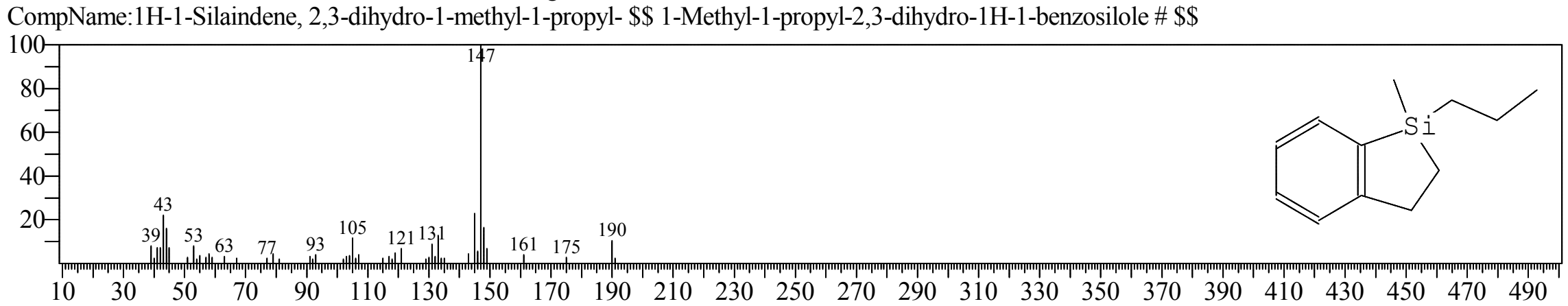

<< Target >>
Line#:23 R.Time:16.000(Scan#:1321) MassPeaks:42
RawMode:Averaged 15.992-16.008(1320-1322) BasePeak:39.95(2006)
BG Mode:Calc. from Peak Group 1 - Event 1 Scan

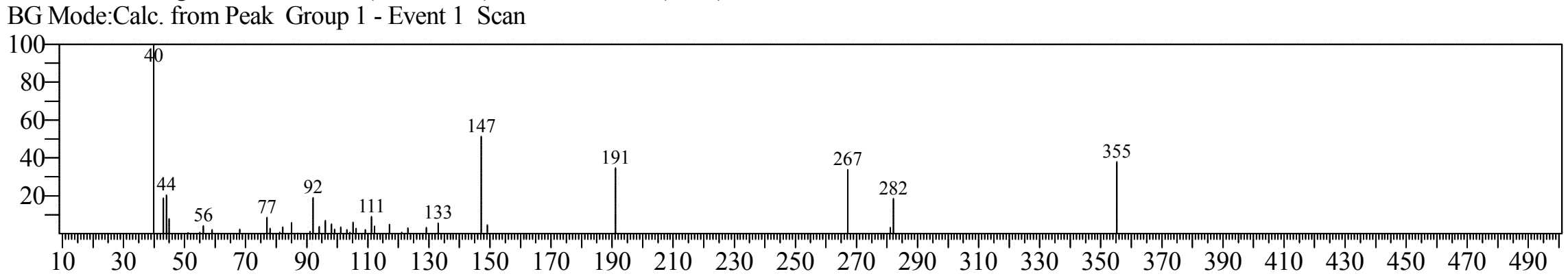

Hit#:11 Entry:10711 Library:WILEY7.LIB
SI:48 Formula:C6 H8 O2 CAS:0-00-0 MolWeight:112 RetIndex:0
CompName:2(5H)-Furanone, 3,5-dimethyl- $$

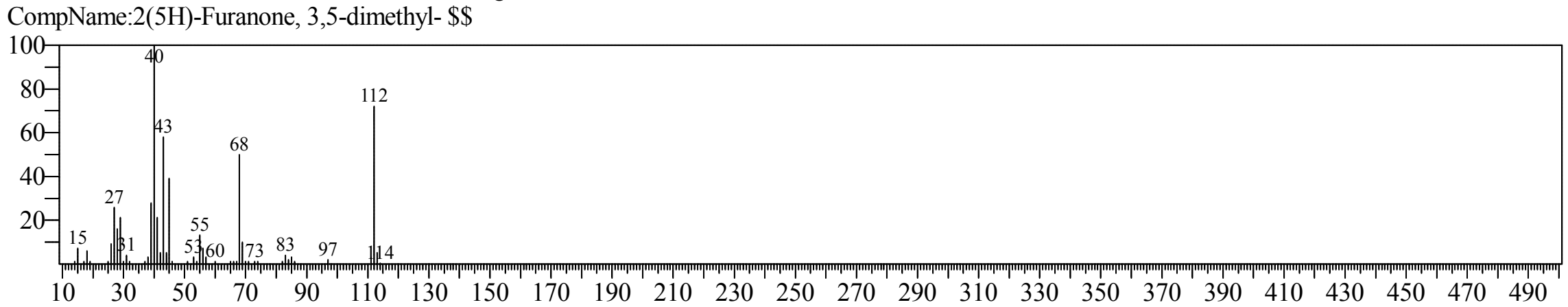

Hit#:12 Entry:106976 Library:NIST05.LIB
SI:48 Formula:C21H26O2 CAS:39000-49-2 MolWeight:310 RetIndex:2288
CompName:2,4-Di-tert-butylphenyl benzoate

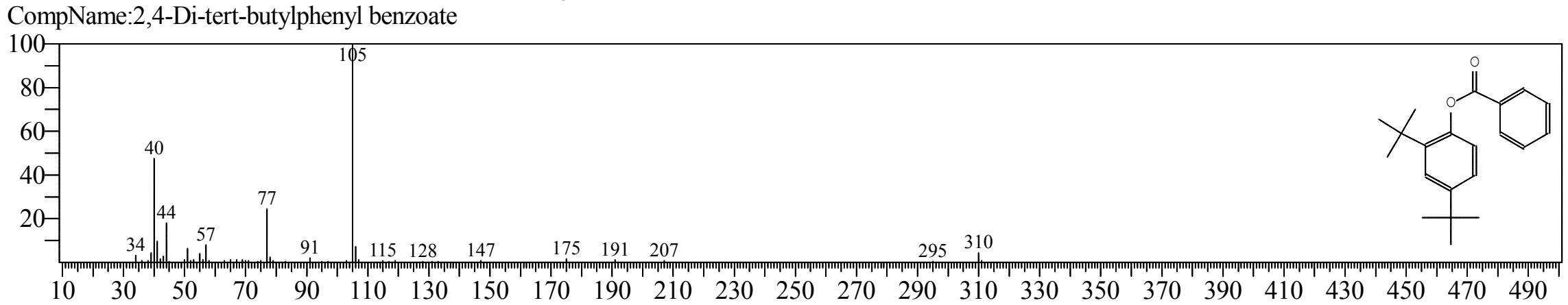

Hit#:13 Entry:221138 Library:WILEY7.LIB
SI:48 Formula:C21 H26 O2 CAS:0-00-0 MolWeight:310 RetIndex:0
CompName:2,4-DI-TERT-BUTYLPHENYL BENZOATE $$

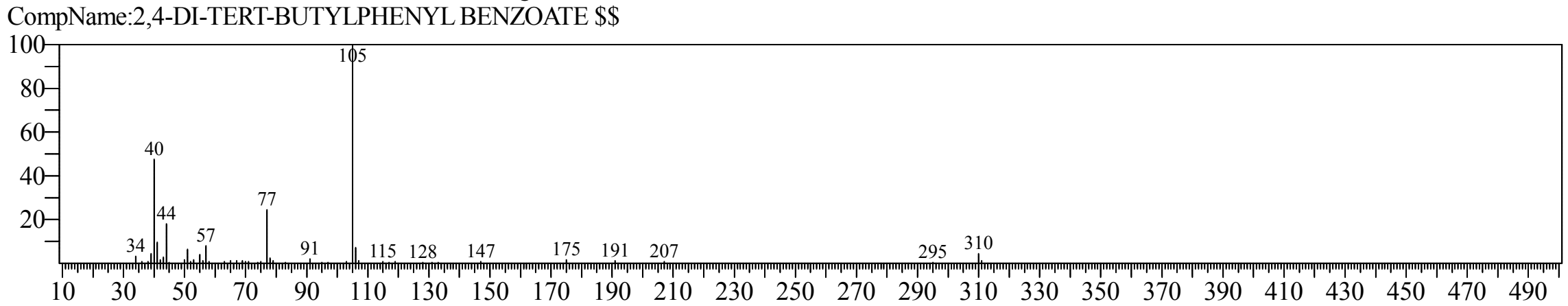

Hit#:14 Entry:235267 Library:WILEY7.LIB
SI:48 Formula:C19 H34 O4 CAS:133695-45-1 MolWeight:326 RetIndex:0
CompName:(3S,4S,5S)-4-Carboxy-3-methyl-2-oxo-5-tridecyltetrahydrofuran $$

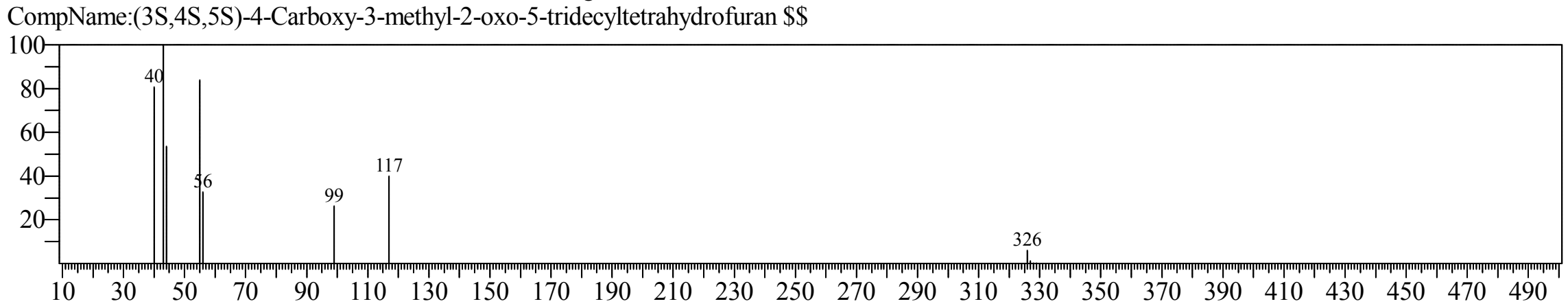

Hit#:15 Entry:108782 Library:NIST05.LIB
SI:48 Formula:C15H23O5P CAS:35085-32-6 MolWeight:314 RetIndex:0
CompName:Benzenepropanoic acid, .alpha.-(diethoxyphosphinyl)-, ethyl ester $$ Propanoic acid, 2-diethylphosphonato-3-phenyl-, ethyl ester $$ Ethyl 2-(di

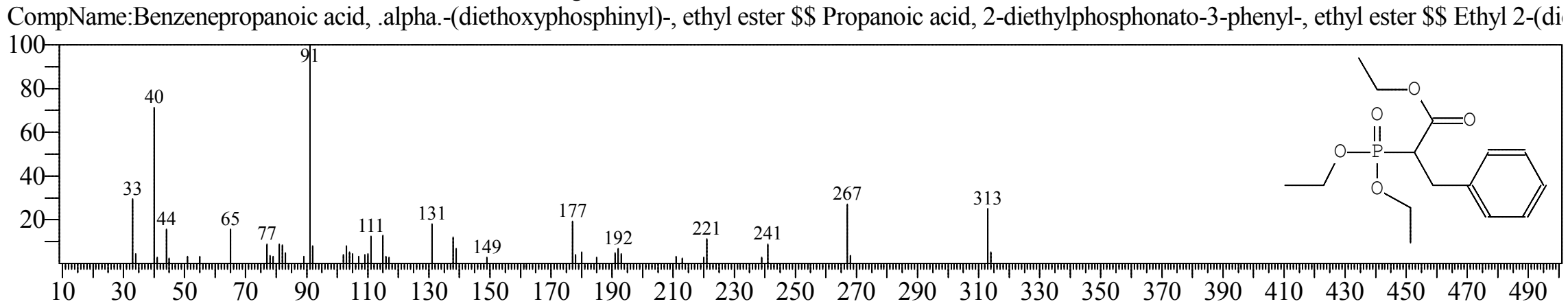

<< Target >>
Line#:23 R.Time:16.000(Scan#:1321) MassPeaks:42
RawMode:Averaged 15.992-16.008(1320-1322) BasePeak:39.95(2006)
BG Mode:Calc. from Peak Group 1 - Event 1 Scan

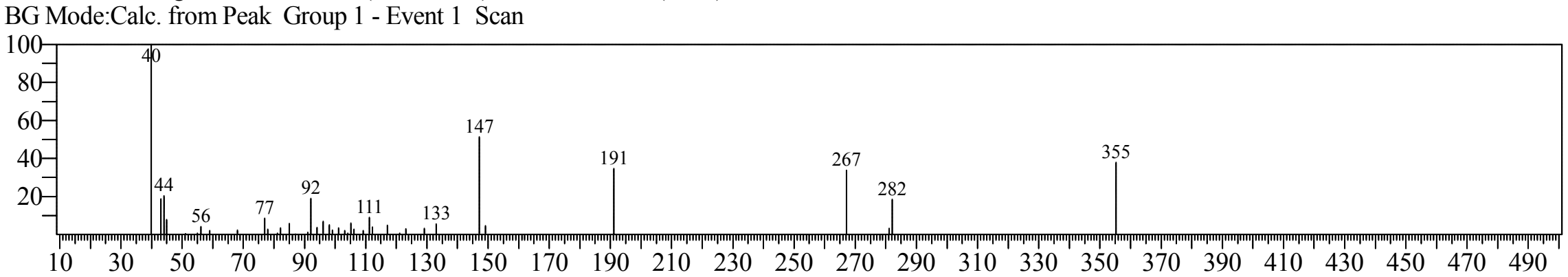

Hit#:16 Entry:212684 Library:WILEY7.LIB
SI:47 Formula:C12 H12 F6 O2 CAS:0-00-0 MolWeight:302 RetIndex:0
CompName:8,9,9,10,10,11-HEXAFLUORO-4,4-DIMETHYL-3,5-DIOXATETRACYCLO[5.4.1.0(2, $$

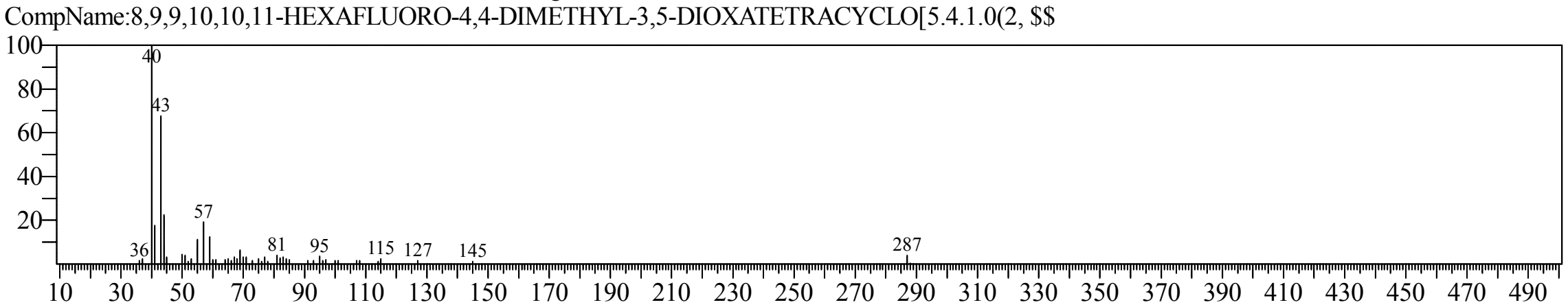

Hit#:17 Entry:17515 Library:NIST05.LIB
SI:47 Formula:C6H4O5 CAS:6318-55-4 MolWeight:156 RetIndex:1524
CompName:cis-Aconitic anhydride $$ (Z)-Aconitic anhydride $$ cis-Aconitic acid anhydride $$ 3-Furanacetic acid, 2,5-dihydro-2,5-dioxo- $$ Aconitic anh

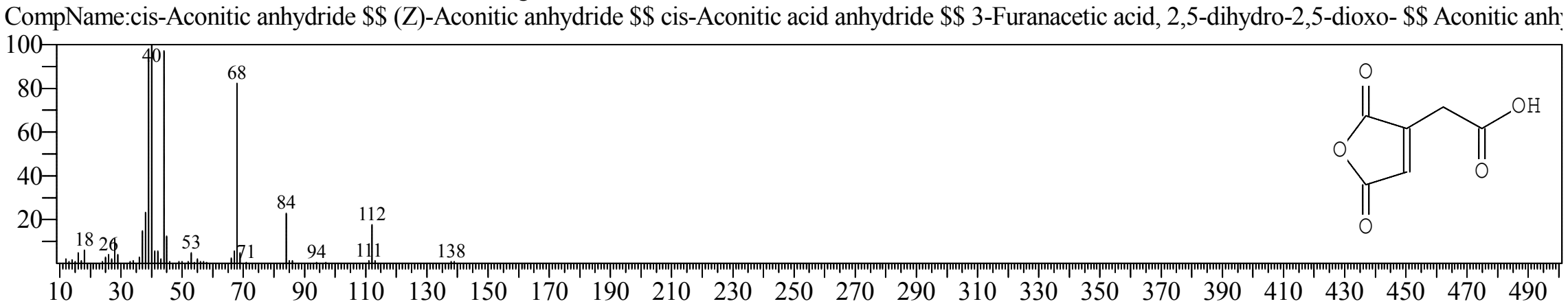

Hit#:18 Entry:106823 Library:NIST11.lib
SI:47 Formula:C18H20ClN CAS:0-00-0 MolWeight:285 RetIndex:2297
CompName:Benzylamine, N-(3-chloro-2,2-dimethyl-1-phenylpropylidene)- $$ N-[(E)-3-Chloro-2,2-dimethyl-1-phenylpropylidene](phenyl)methanamine # $

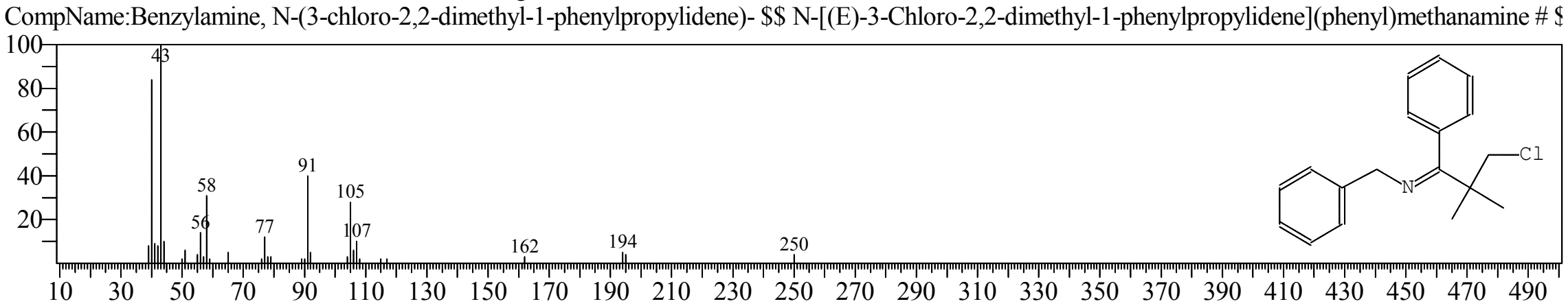

Hit#:19 Entry:92403 Library:NIST05.LIB
SI:47 Formula:C18H20ClN CAS:0-00-0 MolWeight:285 RetIndex:2297
CompName:Benzylamine, N-(3-chloro-2,2-dimethyl-1-phenylpropylidene)-

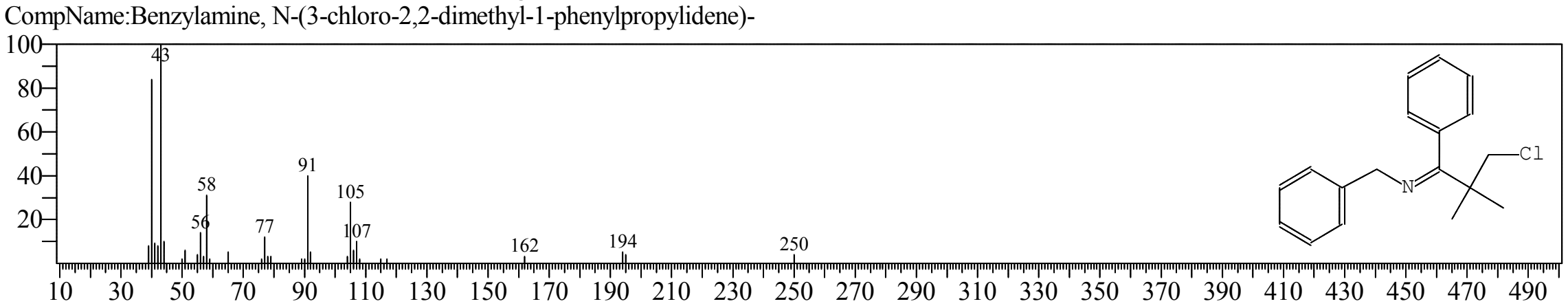

Hit#:20 Entry:196116 Library:WILEY7.LIB
SI:47 Formula:C18 H20 CL N CAS:99315-24-9 MolWeight:285 RetIndex:0
CompName:N-benzyl-3-chloro-2,2-dimethyl-propylimine $$ Benzenemethanamine, N-(3-chloro-2,2-dimethyl-1-phenylpropylidene)- (CAS)

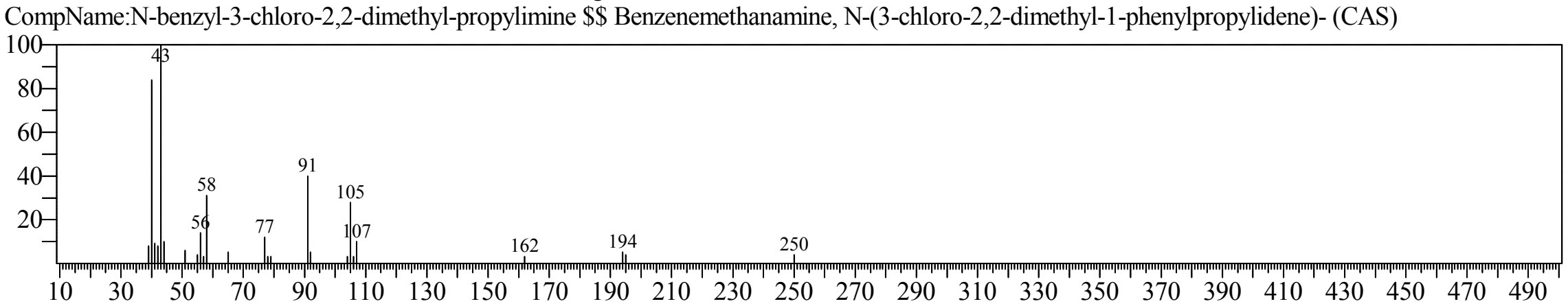

<< Target >>
Line#:24 R.Time:16.767(Scan#:1413) MassPeaks:54
RawMode:Averaged 16.758-16.775(1412-1414) BasePeak:73.10(8635)
BG Mode:Calc. from Peak Group 1 - Event 1 Scan

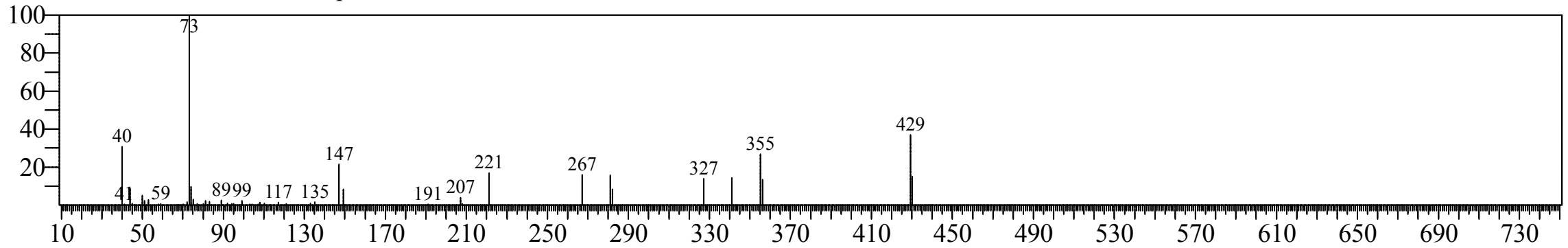

Hit#:1 Entry:152159 Library:NIST05.LIB
SI:72 Formula:C13H40O5Si6 CAS:38147-00-1 MolWeight:444 RetIndex:1297
CompName:1,1,1,5,7,7,7-Heptamethyl-3,3-bis(trimethylsiloxy)tetrasiloxane $$ 1,3,3,3-Tetramethyldisiloxanyl tris(trimethylsilyl) orthosilicate # $$

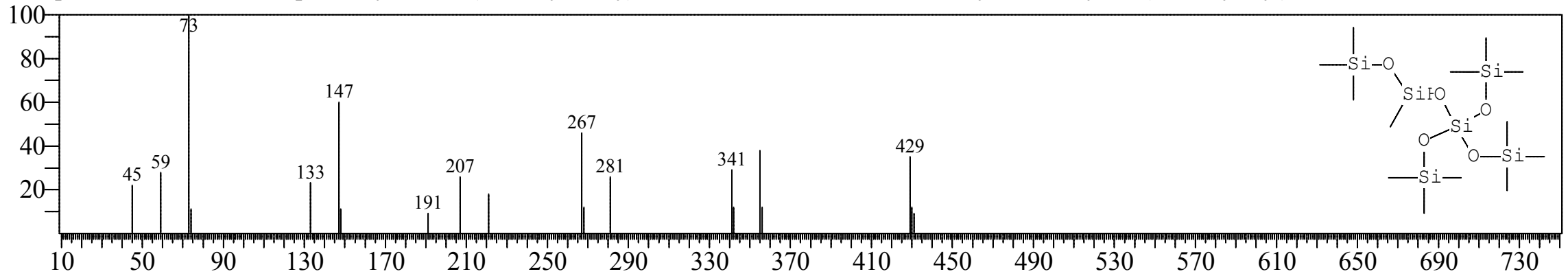

Hit#:2 Entry:160407 Library:NIST05.LIB
SI:69 Formula:C16H50O7Si8 CAS:19095-24-0 MolWeight:578 RetIndex:1710
CompName:Octasiloxane, 1,1,3,3,5,5,7,7,9,9,11,11,13,13,15,15-hexadecamethyl- $$ 1,1,3,3,5,5,7,7,9,9,11,11,13,13,15,15-Hexadecamethyloctasiloxane # $

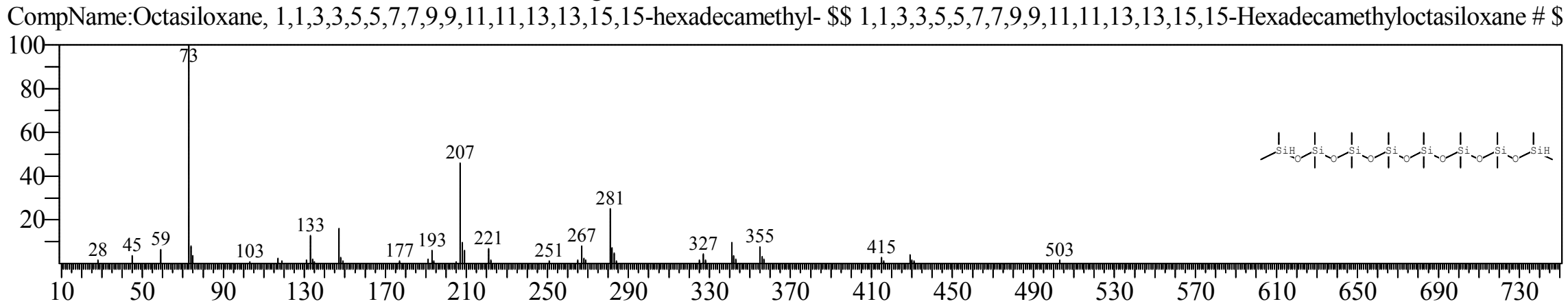

Hit#:3 Entry:157431 Library:NIST05.LIB
SI:69 Formula:C14H44O6Si7 CAS:19095-23-9 MolWeight:504 RetIndex:1526
CompName:Heptasiloxane, 1,1,3,3,5,5,7,7,9,9,11,11,13,13-tetradecamethyl- $$ 1,1,3,3,5,5,7,7,9,9,11,11,13,13-Tetradecamethylheptasiloxane # $$

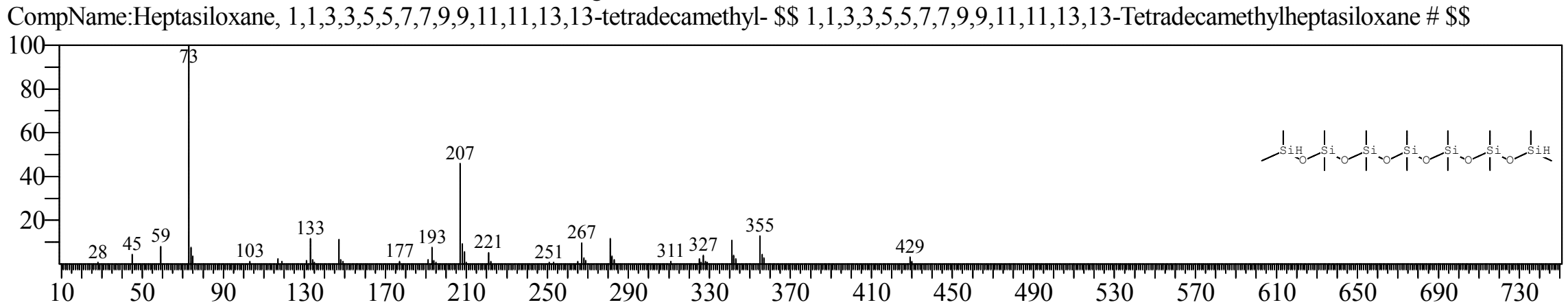

Hit#:4 Entry:317266 Library:WILEY7.LIB
SI:69 Formula:C14 H44 O6 SI7 CAS:0-00-0 MolWeight:504 RetIndex:0
CompName:1,1,3,3,5,5,7,7,9,9,11,11,13,13-TETRADECAMETHYL-HEPTASILOXANE $$

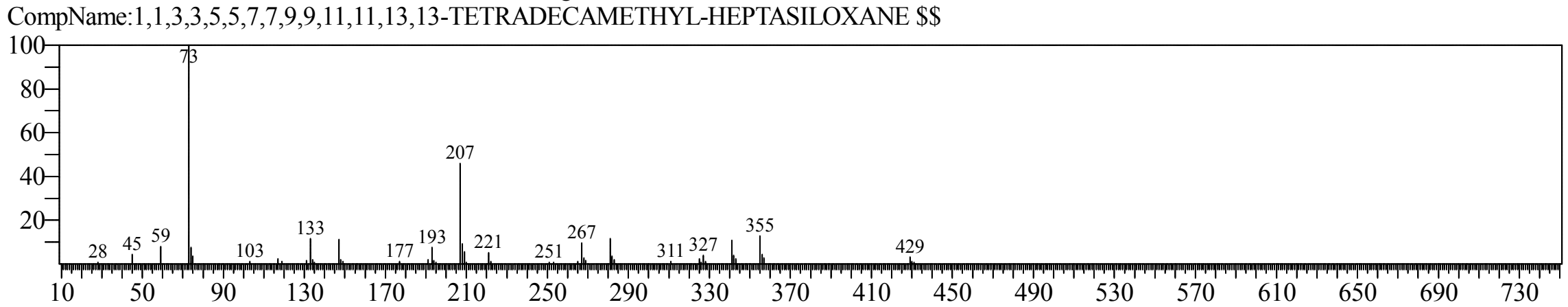

Hit#:5 Entry:335377 Library:WILEY7.LIB
SI:67 Formula:C20 H60 O10 SI10 CAS:18772-36-6 MolWeight:740 RetIndex:0
CompName:EICOSAMETHYLCYCLODECASILOXANE $$ CYCLODECASILOXANE, EICOSAMETHYL- $$

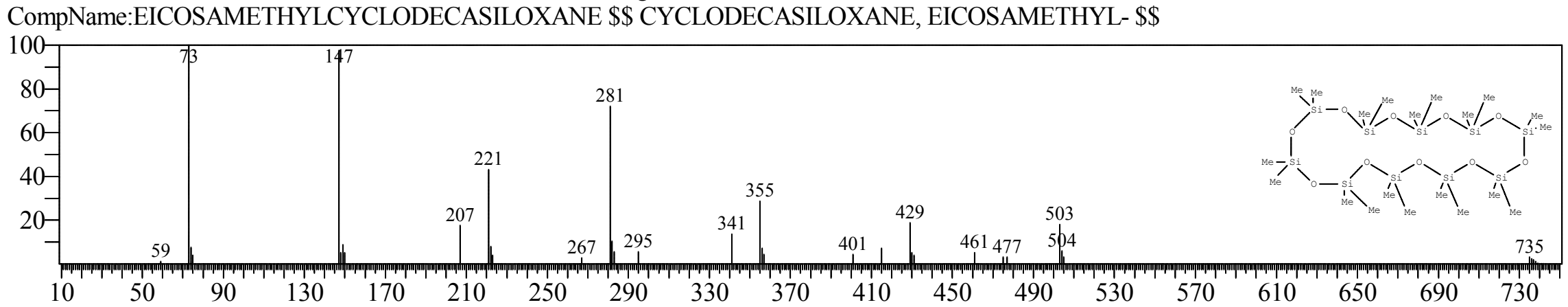

<< Target >>
Line#:24 R.Time:16.767(Scan#:1413) MassPeaks:54
RawMode:Averaged 16.758-16.775(1412-1414) BasePeak:73.10(8635)
BG Mode:Calc. from Peak Group 1 - Event 1 Scan

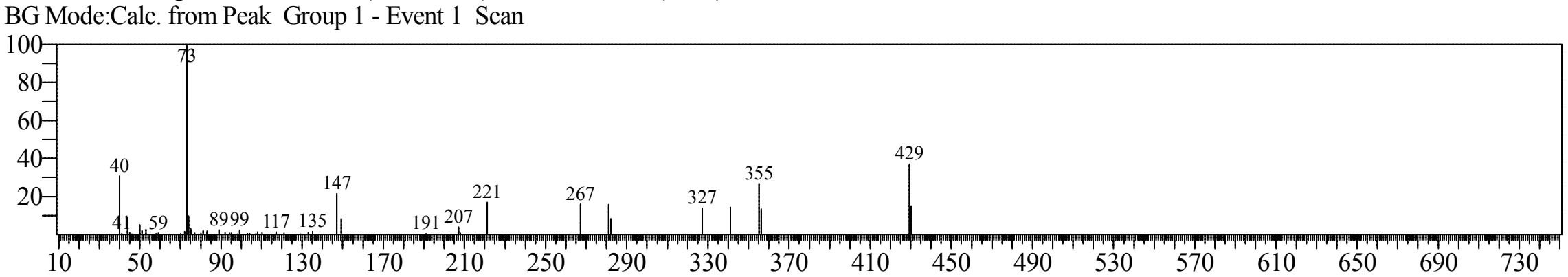

Hit#:6 Entry:161854 Library:NIST05.LIB
SI:67 Formula:C16H48O10Si9 CAS:145344-72-5 MolWeight:652 RetIndex:1716
CompName:2-(2',4',4',6',6',8',8'-Heptamethyltetrasiloxan-2'-yloxy)-2,4,4,6,6,8,8,10,10-nonamethylcyclopentasiloxane $$ 2-[(2,4,4,6,6,8,8-Heptamethyl-1,3,5

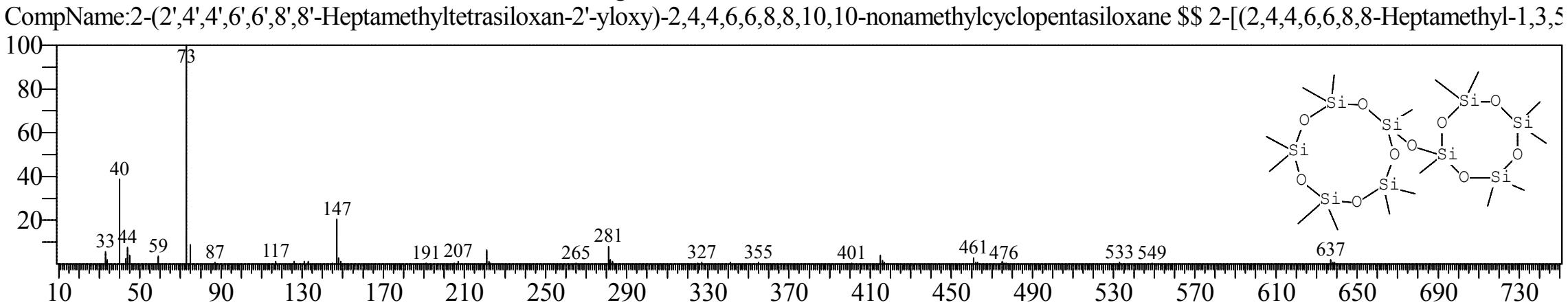

Hit#:7 Entry:310786 Library:WILEY7.LIB
SI:67 Formula:C20 H40 O5 SI4 CAS:37148-65-5 MolWeight:472 RetIndex:0
CompName:Benzeneacetic acid, .alpha.,3,4-tris[(trimethylsilyl)oxy]-, trimethylsilyl ester (CAS) TETRAKISTRIMETHYLSILYL 3,4-DIHYDROXYMAND

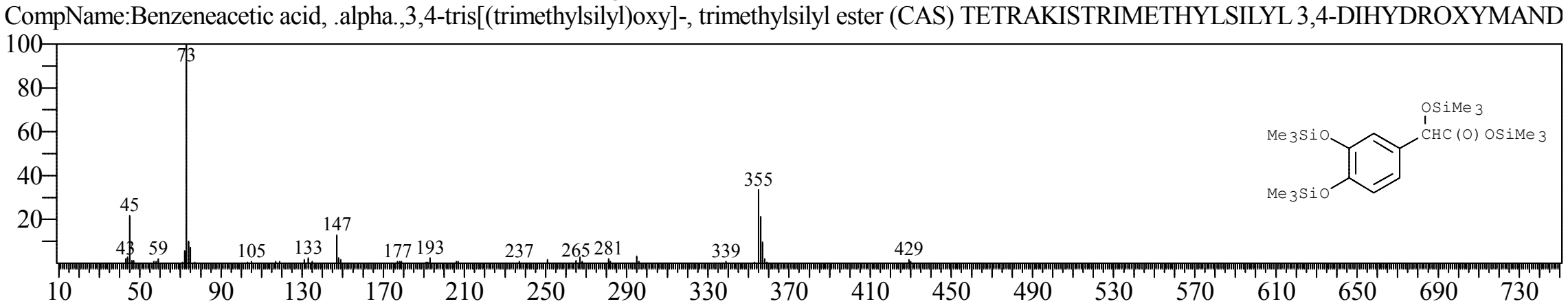

Hit#:8 Entry:152156 Library:NIST05.LIB
SI:67 Formula:C12H36O6Si6 CAS:540-97-6 MolWeight:444 RetIndex:1240
CompName:Cyclohexasiloxane, dodecamethyl- $$ Dodecamethylcyclohexasiloxane $$ 2,2,4,4,6,6,8,8,10,10,12,12-Dodecamethylcyclohexasiloxane # $$

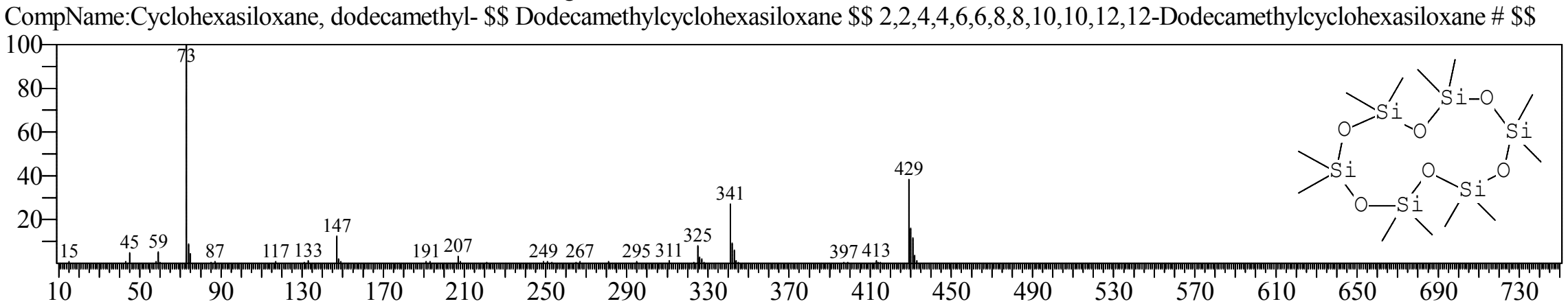

Hit#:9 Entry:27563 Library:NIST05s.LIB
SI:65 Formula:C18H54O9Si9 CAS:556-71-8 MolWeight:666 RetIndex:1860
CompName:Cyclononasiloxane, octadecamethyl- $$ Octadeamethyl-cyclononasiloxane $$ 2,2,4,4,6,6,8,8,10,10,12,12,14,14,16,16,18,18-Octadecamethylcy

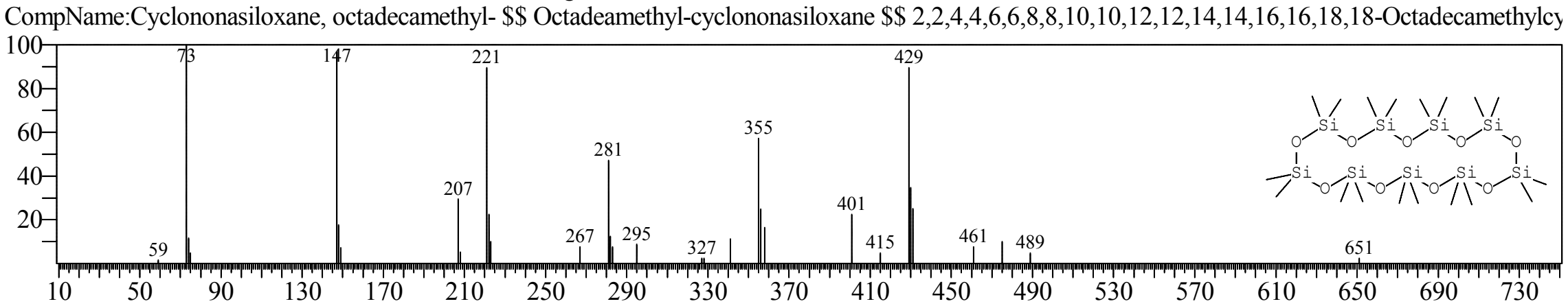

Hit#:10 Entry:306567 Library:WILEY7.LIB
SI:65 Formula:C14 H42 O5 SI6 CAS:107-52-8 MolWeight:458 RetIndex:0
CompName:Hexasiloxane, tetradecamethyl- (CAS) Tetradecamethylhexasiloxane $$

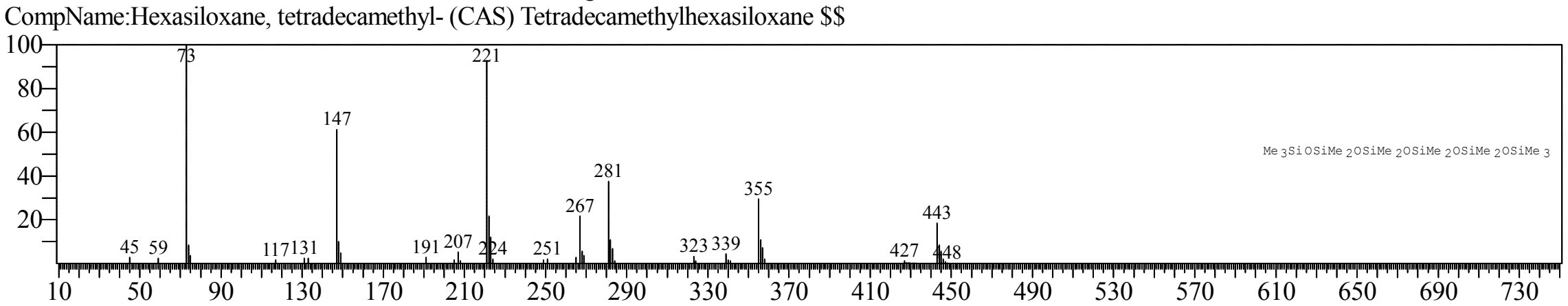

<< Target >>
Line#:24 R.Time:16.767(Scan#:1413) MassPeaks:54
RawMode:Averaged 16.758-16.775(1412-1414) BasePeak:73.10(8635)
BG Mode:Calc. from Peak Group 1 - Event 1 Scan

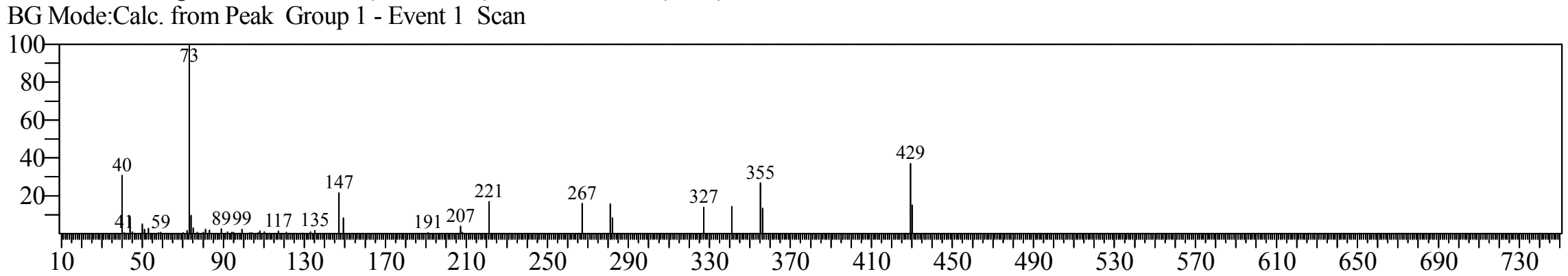

Hit#:11 Entry:338296 Library:WILEY7.LIB
SI:64 Formula: CAS:0-00-0 MolWeight:9999 RetIndex:0
CompName:SILIKONFETT SE30 (GREVELS) $$ Silicone oil $$

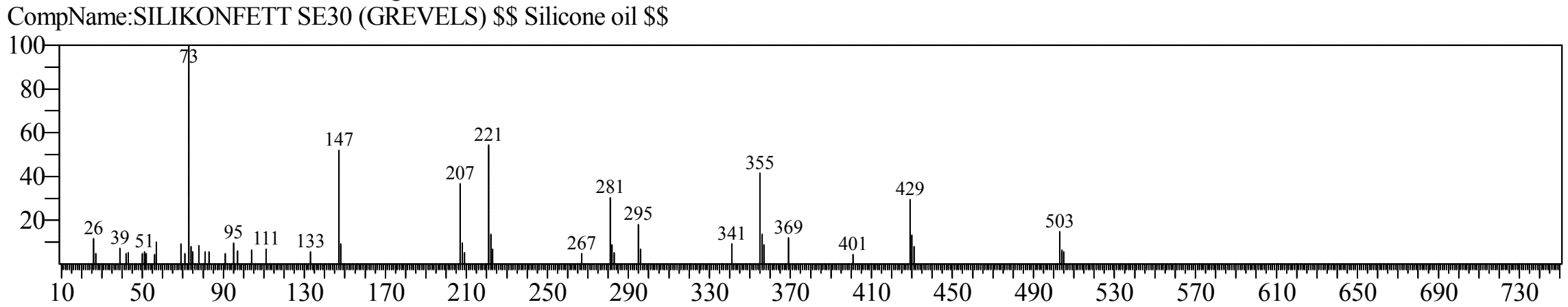

Hit#:12 Entry:300653 Library:WILEY7.LIB
SI:64 Formula:C21 H22 FE N2 O5 CAS:109007-87-6 MolWeight:438 RetIndex:0
CompName:Iron, monocarbonyl-(1,3-butadiene-1,4-dicarbonic acid, diethyl ester) a,a'-dipyridyl $$ IRON, (2,2'-BIPYRIDINE-N,N')CARBONYL[(2,3,4,5-.

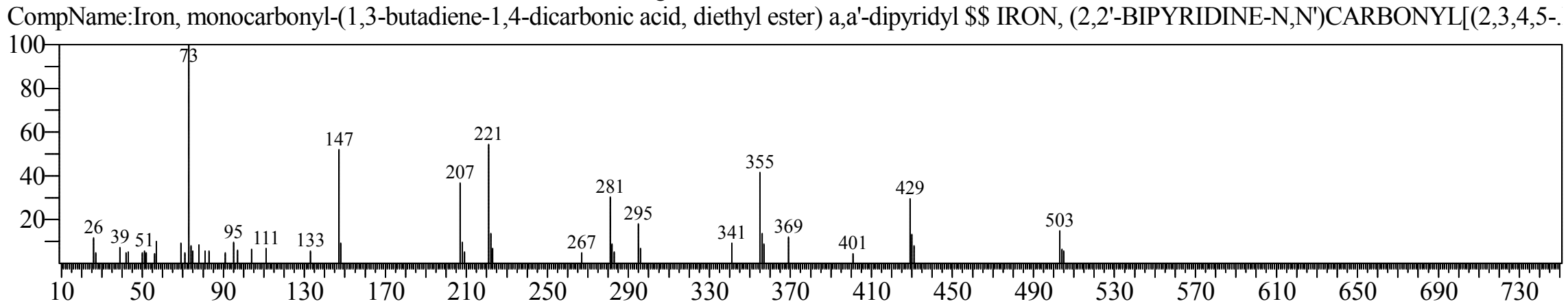

Hit#:13 Entry:158804 Library:NIST05.LIB
SI:64 Formula:C16H48O6Si7 CAS:541-01-5 MolWeight:532 RetIndex:1437
CompName:Heptasiloxane, hexadecamethyl- $$ Hexadecamethylheptasiloxane $$ 1,1,1,3,3,5,5,7,7,9,9,11,11,13,13,13-Hexadecamethylheptasiloxane # $$

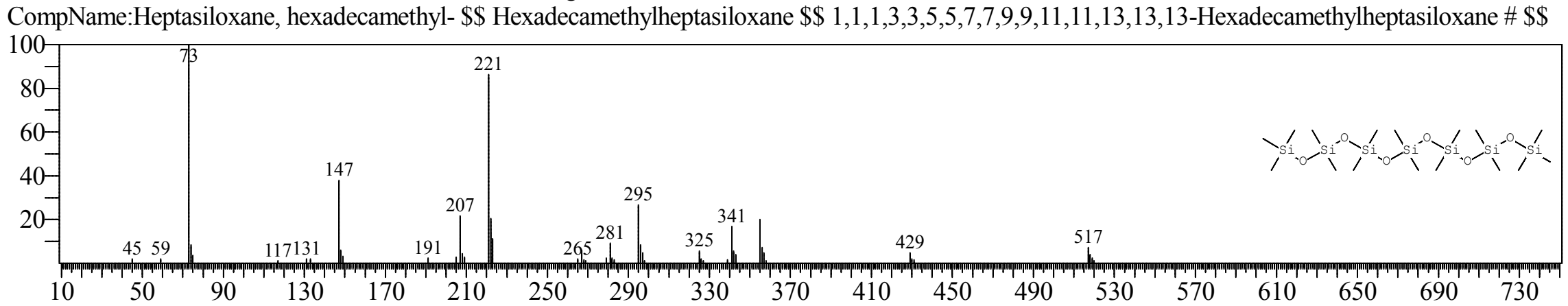

Hit#:14 Entry:153706 Library:NIST05.LIB
SI:64 Formula:C14H42O5Si6 CAS:2003-92-1 MolWeight:458 RetIndex:1252
CompName:1,1,1,3,5,7,7,7-Octamethyl-3,5-bis(trimethylsiloxy)tetrasiloxane $$ 1,1,1,3,5,7,7,7-Octamethyl-3,5-bis[(trimethylsilyl)oxy]tetrasiloxane # $$

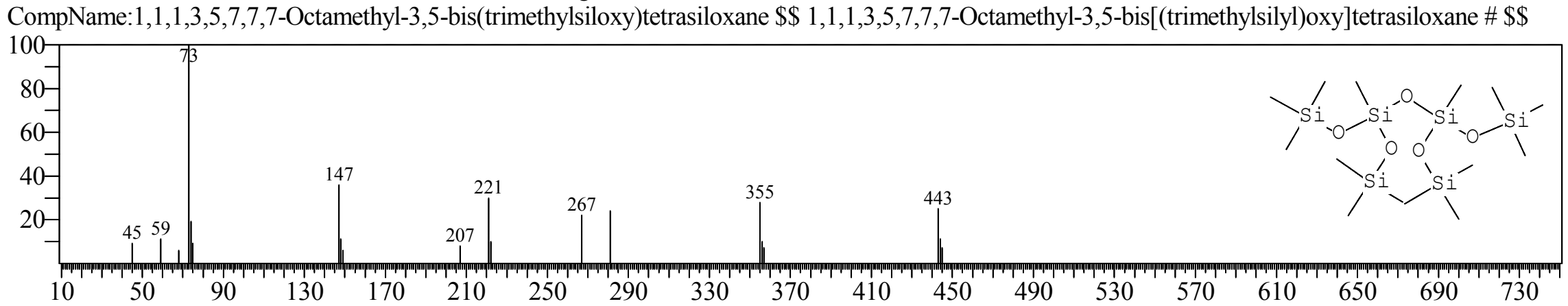

Hit#:15 Entry:306966 Library:WILEY7.LIB
SI:64 Formula:C20 H42 O4 SI4 CAS:56114-62-6 MolWeight:458 RetIndex:0
CompName:Silane, [[4-[1,2-bis[(trimethylsilyl)oxy]ethyl]-1,2-phenylene]bis(oxy)]bis[trimethyl- (CAS) 3,4-DIHYDROXYPHENYLGLYCOL-TETRATMS

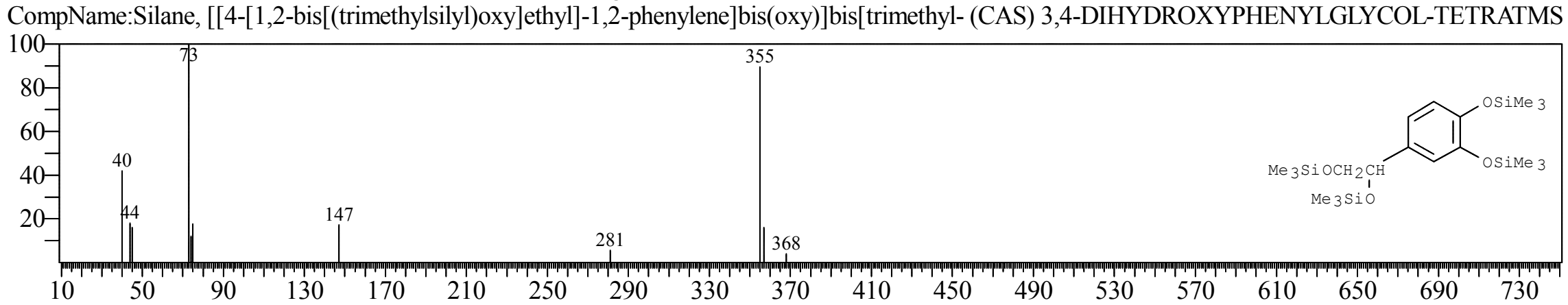

<< Target >>
Line#:24 R.Time:16.767(Scan#:1413) MassPeaks:54
RawMode:Averaged 16.758-16.775(1412-1414) BasePeak:73.10(8635)
BG Mode:Calc. from Peak Group 1 - Event 1 Scan

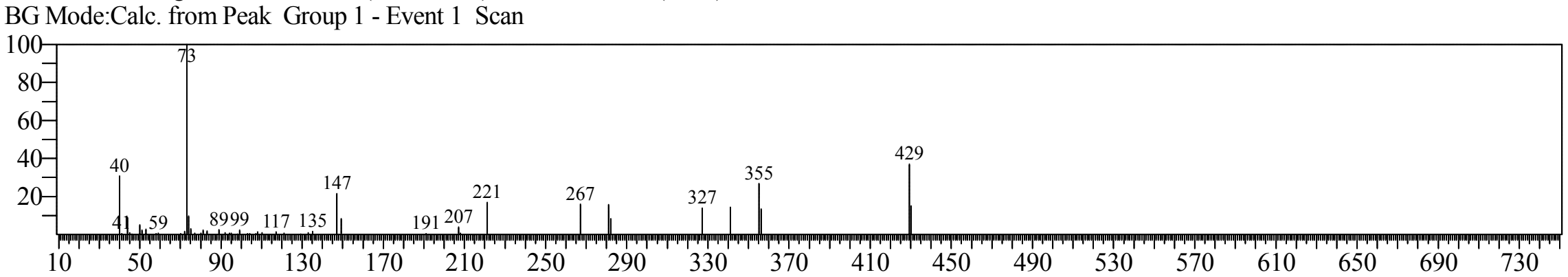

Hit#:16 Entry:159651 Library:NIST05.LIB
SI:64 Formula:C22H42F3NO4Si4 CAS:0-00-0 MolWeight:553 RetIndex:2151
CompName:N-(Trifluoroacetyl)-N,O,O',O''-tetrakis(trimethylsilyl)norepinephrine

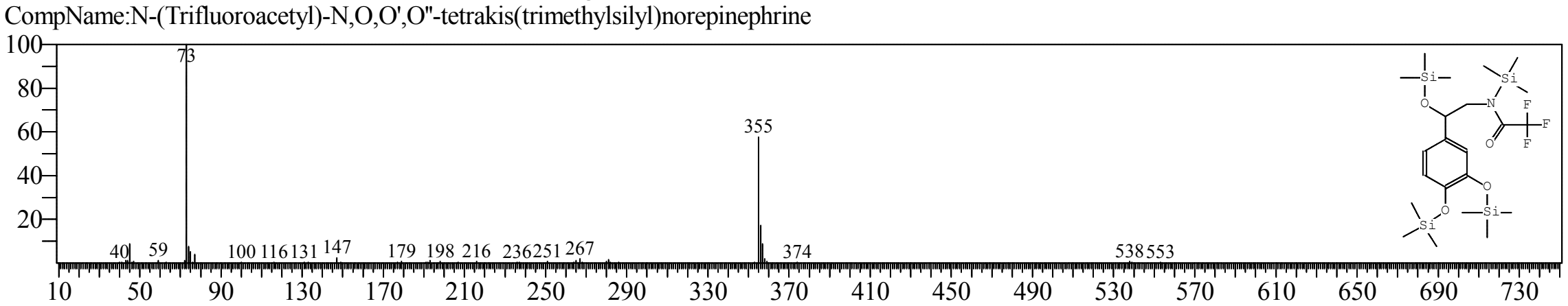

Hit#:17 Entry:208545 Library:NIST11.lib
SI:64 Formula:C22H42F3NO4Si4 CAS:0-00-0 MolWeight:553 RetIndex:2151
CompName:N-(Trifluoroacetyl)-N,O,O',O''-tetrakis(trimethylsilyl)norepinephrine $$ N-(2-(3,4-Bis[(trimethylsilyl)oxy]phenyl)-2-[(trimethylsilyl)oxy]ethyl)-2

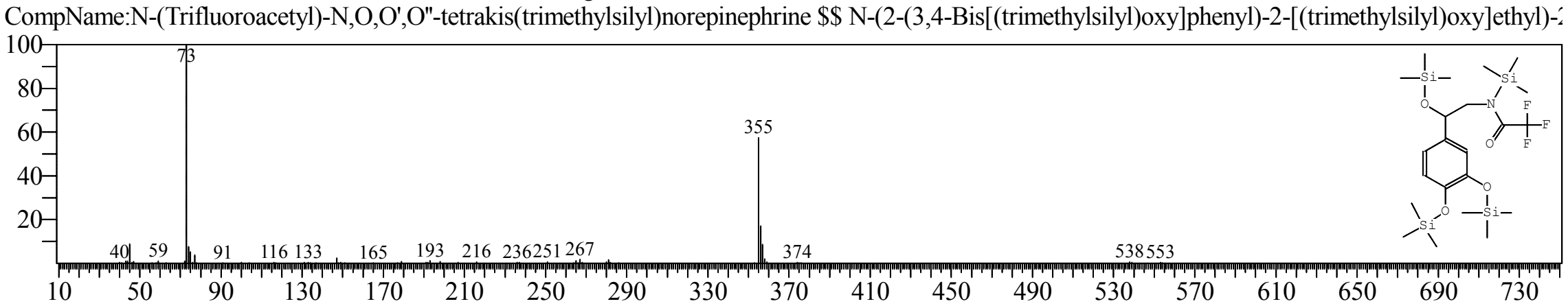

Hit#:18 Entry:156530 Library:NIST05.LIB
SI:63 Formula:C13H42O6Si7 CAS:50694-26-3 MolWeight:490 RetIndex:1570
CompName:1,1,1,3,5,7,9,11,11,11-Decamethyl-5-(trimethylsiloxy)hexasiloxane $$ 1,1,1,3,5,7,9,11,11,11-Decamethyl-5-[(trimethylsilyl)oxy]hexasiloxane #

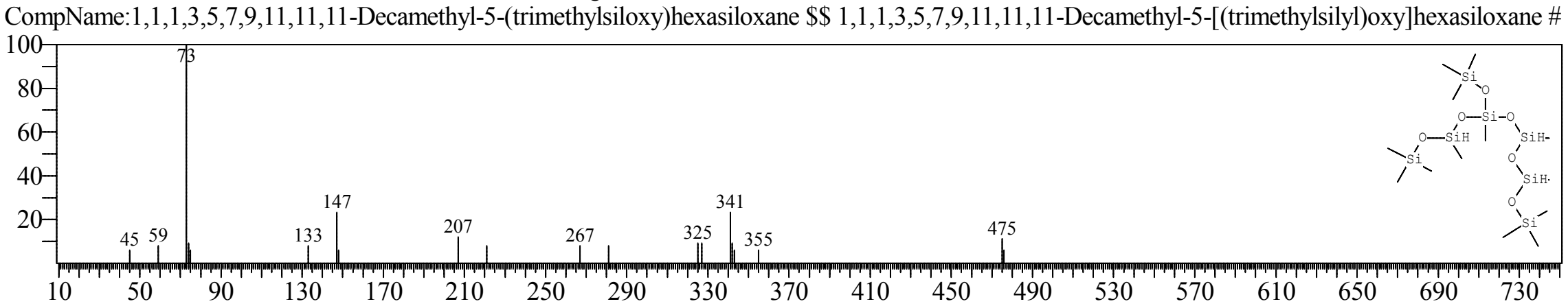

Hit#:19 Entry:267981 Library:WILEY7.LIB
SI:63 Formula:C16 H30 O4 SI3 CAS:3782-85-2 MolWeight:370 RetIndex:0
CompName:2,6-DIHYDROXYBENZOIC ACID 3TMS $$ trimethylsilyl [2,6-di(trimethylsiloxy)phenyl]-methanoate $$ Benzoic acid, 2,6-bis[(trimethylsily

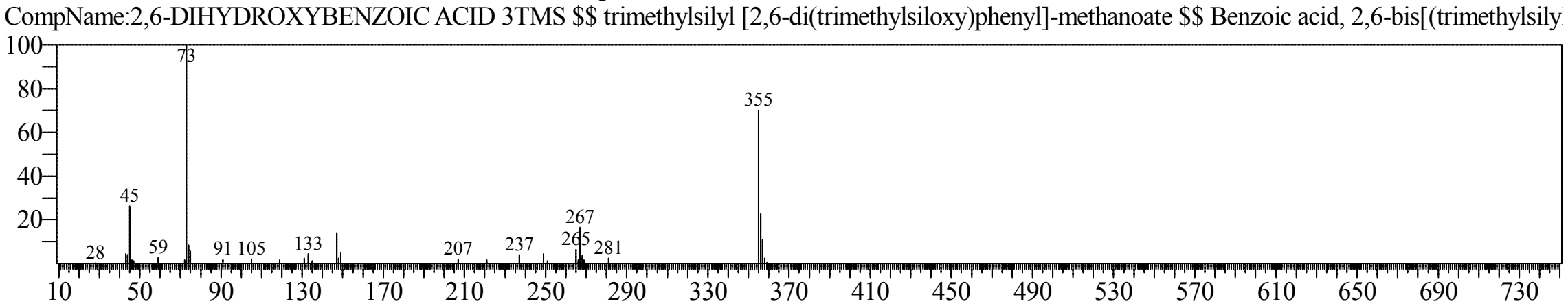

Hit#:20 Entry:27443 Library:NIST05s.LIB
SI:63 Formula:C24H34F5NO3Si3 CAS:55429-13-5 MolWeight:563 RetIndex:2572
CompName:Benzeneethanamine, N-[(pentafluorophenyl)methylene]-.beta.,3,4-tris[(trimethylsilyl)oxy]- $$ N-(Pentafluorobenzylidene)-.beta.,3,4-tris(trimeth

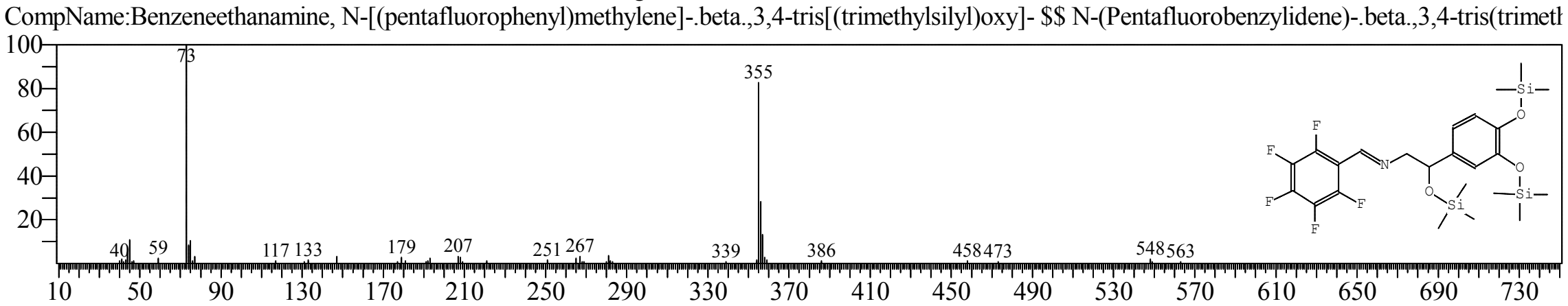

<< Target >>
Line#:25 R.Time:18.792(Scan#:1656) MassPeaks:59
RawMode:Averaged 18.783-18.800(1655-1657) BasePeak:73.05(6700)
BG Mode:Calc. from Peak Group 1 - Event 1 Scan

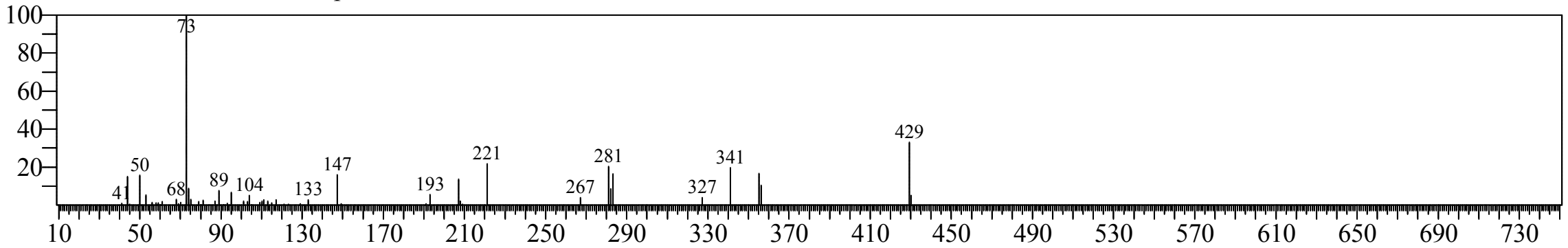

Hit#:1 Entry:160407 Library:NIST05.LIB
SI:72 Formula:C16H50O7Si8 CAS:19095-24-0 MolWeight:578 RetIndex:1710
CompName:Octasiloxane, 1,1,3,3,5,5,7,7,9,9,11,11,13,13,15,15-hexadecamethyl- $$ 1,1,3,3,5,5,7,7,9,9,11,11,13,13,15,15-Hexadecamethyloctasiloxane # $

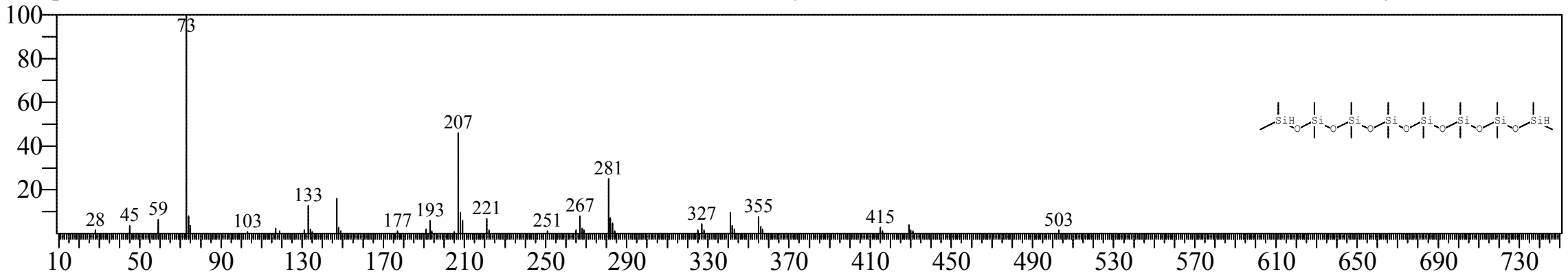

Hit#:2 Entry:317266 Library:WILEY7.LIB
SI:71 Formula:C14 H44 O6 SI7 CAS:0-00-0 MolWeight:504 RetIndex:0
CompName:1,1,3,3,5,5,7,7,9,9,11,11,13,13-TETRADECAMETHYL-HEPTASILOXANE $$

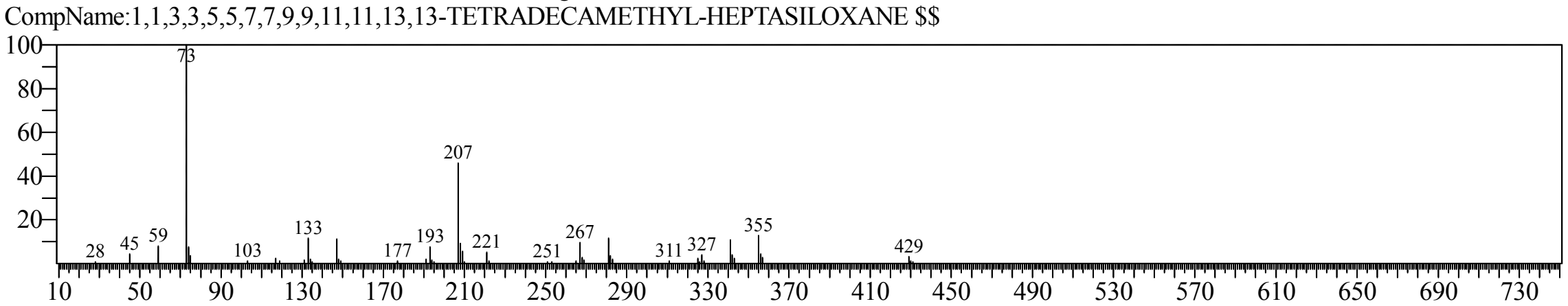

Hit#:3 Entry:157431 Library:NIST05.LIB
SI:71 Formula:C14H44O6Si7 CAS:19095-23-9 MolWeight:504 RetIndex:1526
CompName:Heptasiloxane, 1,1,3,3,5,5,7,7,9,9,11,11,13,13-tetradecamethyl- $$ 1,1,3,3,5,5,7,7,9,9,11,11,13,13-Tetradecamethylheptasiloxane # $$

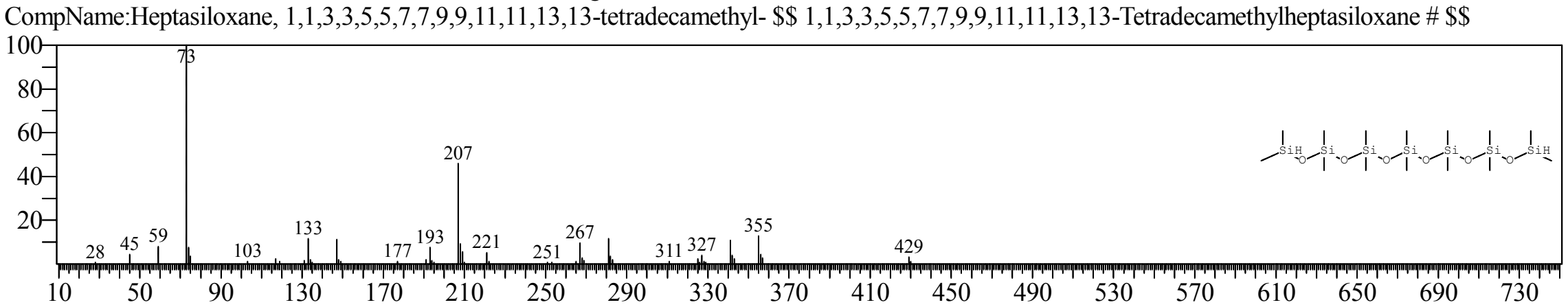

Hit#:4 Entry:152159 Library:NIST05.LIB
SI:69 Formula:C13H40O5Si6 CAS:38147-00-1 MolWeight:444 RetIndex:1297
CompName:1,1,1,5,7,7,7-Heptamethyl-3,3-bis(trimethylsiloxy)tetrasiloxane $$ 1,3,3,3-Tetramethyldisiloxanyl tris(trimethylsilyl) orthosilicate # $$

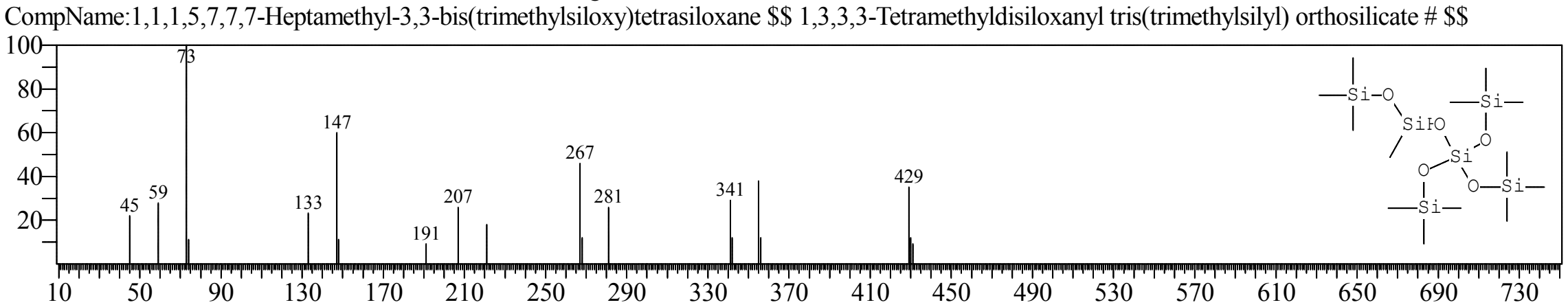

Hit#:5 Entry:335377 Library:WILEY7.LIB
SI:67 Formula:C20 H60 O10 SI10 CAS:18772-36-6 MolWeight:740 RetIndex:0
CompName:EICOSAMETHYLCYCLODECASILOXANE $$ CYCLODECASILOXANE, EICOSAMETHYL- $$

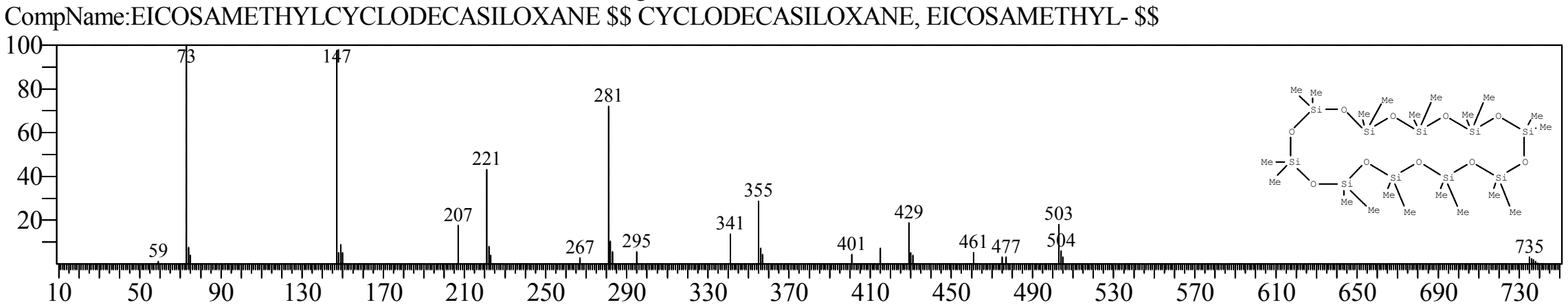

<< Target >>
Line#:25 R.Time:18.792(Scan#:1656) MassPeaks:59
RawMode:Averaged 18.783-18.800(1655-1657) BasePeak:73.05(6700)
BG Mode:Calc. from Peak Group 1 - Event 1 Scan

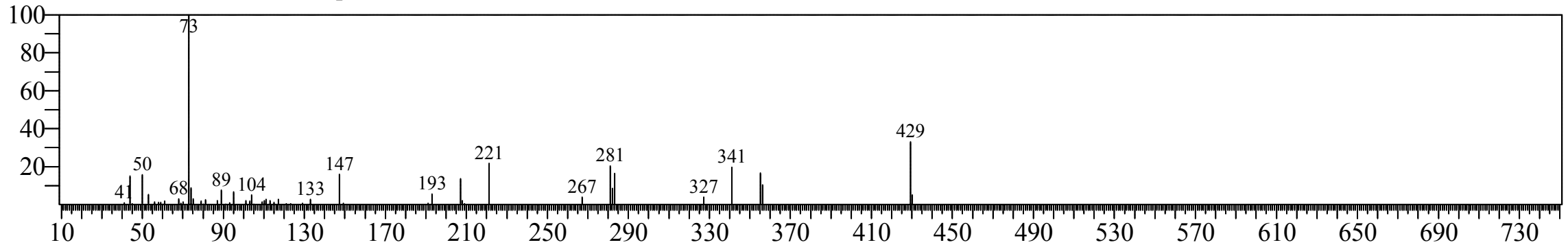

Hit#:6 Entry:338296 Library:WILEY7.LIB
SI:67 Formula: CAS:0-00-0 MolWeight:9999 RetIndex:0
CompName:SILIKONFETT SE30 (GREVELS) $$ Silicone oil $$

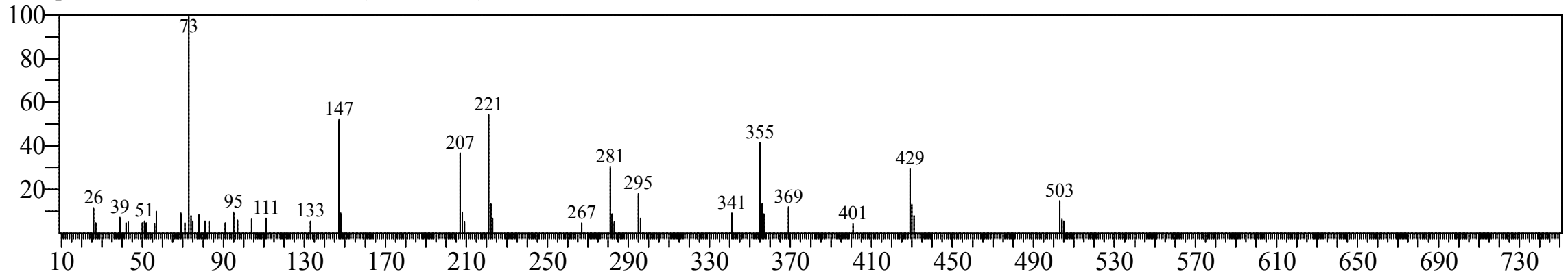

Hit#:7 Entry:300653 Library:WILEY7.LIB
SI:67 Formula:C21 H22 FE N2 O5 CAS:109007-87-6 MolWeight:438 RetIndex:0
CompName:Iron, monocarbonyl-(1,3-butadiene-1,4-dicarbonic acid, diethyl ester) a,a'-dipyridyl $$ IRON, (2,2'-BIPYRIDINE-N,N')CARBONYL[(2,3,4,5-.

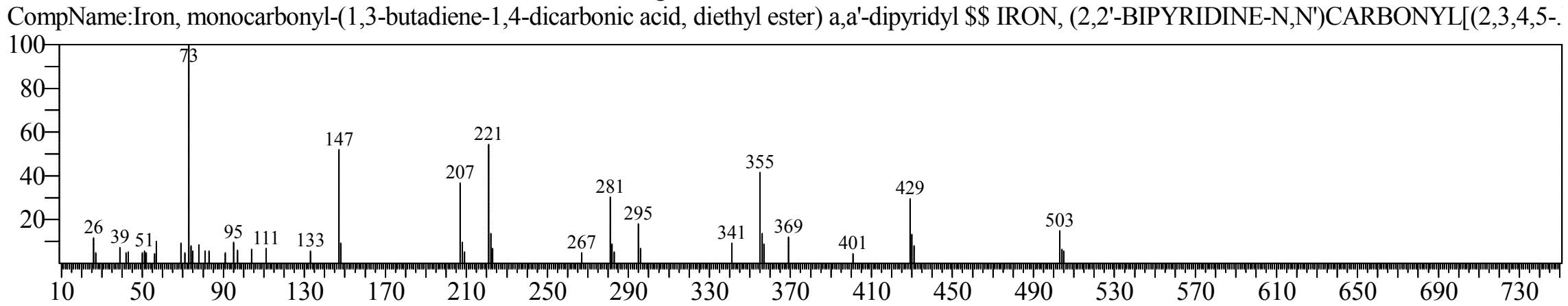

Hit#:8 Entry:150228 Library:NIST05.LIB
SI:66 Formula:C12H38O5Si6 CAS:995-82-4 MolWeight:430 RetIndex:1341
CompName:Hexasiloxane, 1,1,3,3,5,5,7,7,9,9,11,11-dodecamethyl- $$ 1,1,3,3,5,5,7,7,9,9,11,11-Dodecamethylhexasiloxane # $$

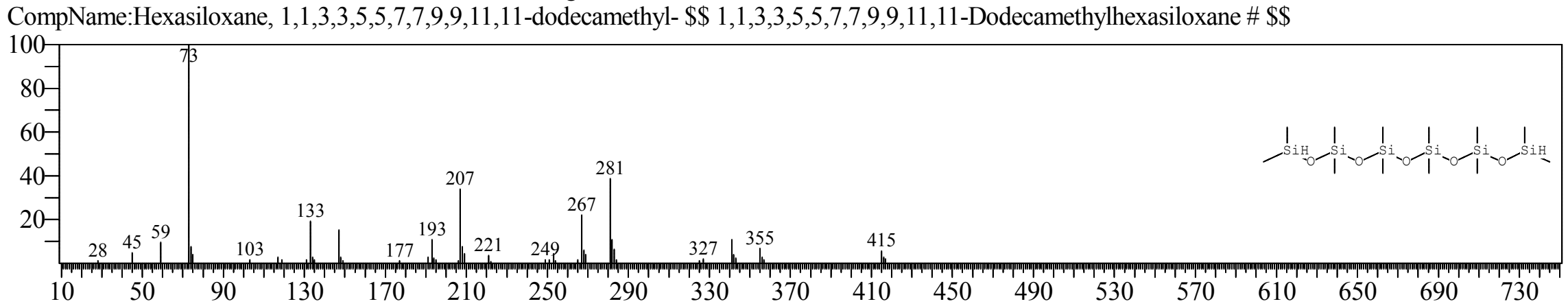

Hit#:9 Entry:297348 Library:WILEY7.LIB
SI:66 Formula:C12 H38 O5 SI6 CAS:0-00-0 MolWeight:430 RetIndex:0
CompName:1,1,3,3,5,5,7,7,9,9,11,11-DODECAMETHYL-HEXASILOXANE $$

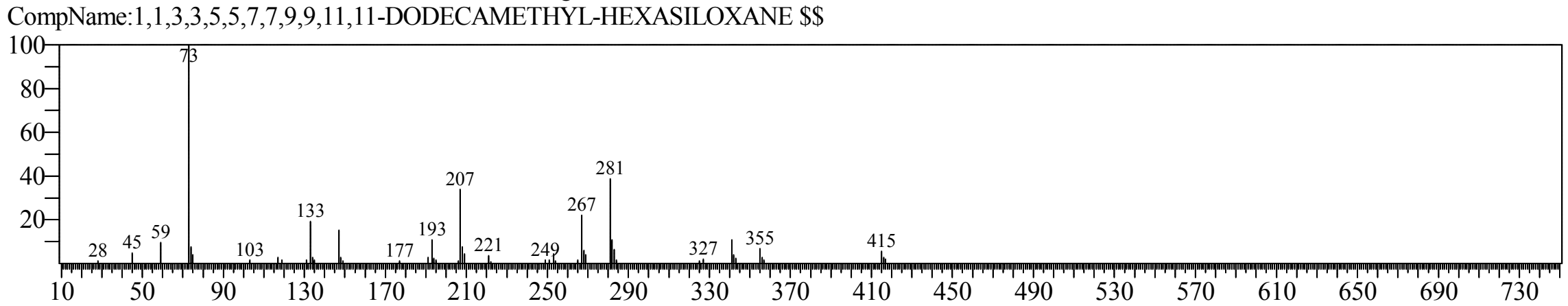

Hit#:10 Entry:152156 Library:NIST05.LIB
SI:65 Formula:C12H36O6Si6 CAS:540-97-6 MolWeight:444 RetIndex:1240
CompName:Cyclohexasiloxane, dodecamethyl- $$ Dodecamethylcyclohexasiloxane $$ 2,2,4,4,6,6,8,8,10,10,12,12-Dodecamethylcyclohexasiloxane # $$

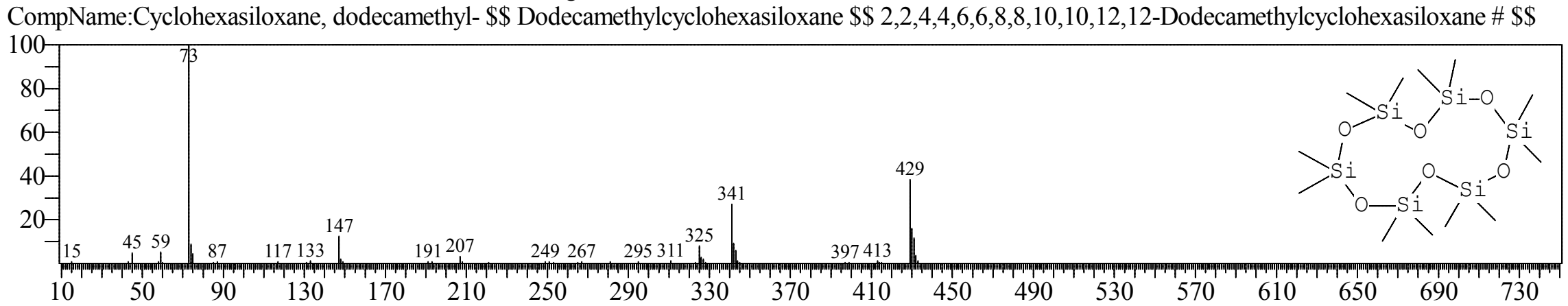

<< Target >>
Line#:25 R.Time:18.792(Scan#:1656) MassPeaks:59
RawMode:Averaged 18.783-18.800(1655-1657) BasePeak:73.05(6700)
BG Mode:Calc. from Peak Group 1 - Event 1 Scan

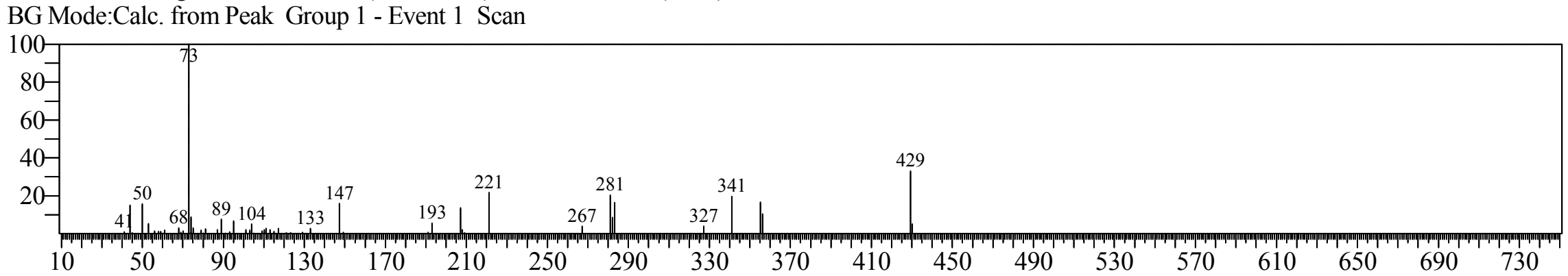

Hit#:11 Entry:158804 Library:NIST05.LIB
SI:64 Formula:C16H48O6Si7 CAS:541-01-5 MolWeight:532 RetIndex:1437
CompName:Heptasiloxane, hexadecamethyl- $$ Hexadecamethylheptasiloxane $$ 1,1,1,3,3,5,5,7,7,9,9,11,11,13,13,13-Hexadecamethylheptasiloxane # $$

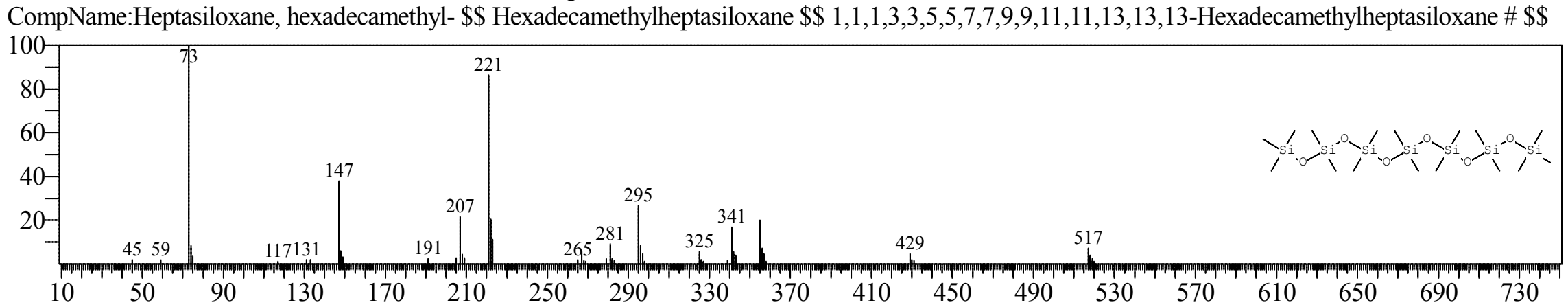

Hit#:12 Entry:162013 Library:NIST05.LIB
SI:63 Formula:C18H54O9Si9 CAS:556-71-8 MolWeight:666 RetIndex:1860
CompName:Cyclononasiloxane, octadecamethyl- $$ Octadeamethyl-cyclononasiloxane $$ 2,2,4,4,6,6,8,8,10,10,12,12,14,14,16,16,18,18-Octadecamethylcy

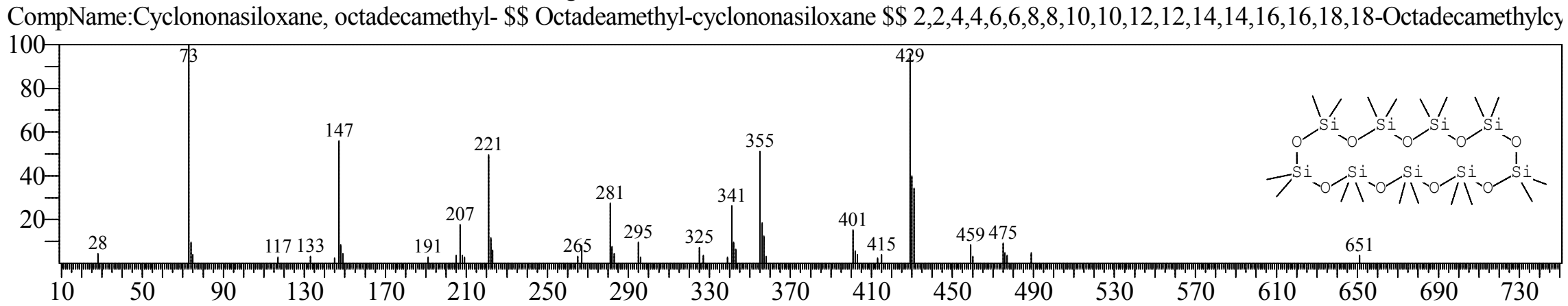

Hit#:13 Entry:306567 Library:WILEY7.LIB
SI:63 Formula:C14 H42 O5 SI6 CAS:107-52-8 MolWeight:458 RetIndex:0
CompName:Hexasiloxane, tetradecamethyl- (CAS) Tetradecamethylhexasiloxane $$

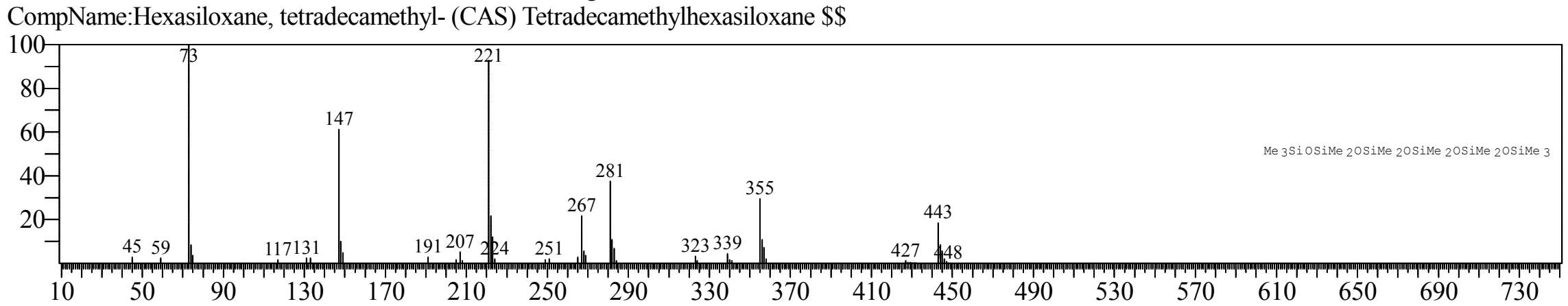

Hit#:14 Entry:160345 Library:NIST05.LIB
SI:63 Formula:C18H52O7Si7 CAS:71579-69-6 MolWeight:576 RetIndex:1648
CompName:3-Isopropoxy-1,1,1,7,7,7-hexamethyl-3,5,5-tris(trimethylsiloxy)tetrasiloxane $$ 1-Isopropoxy-3,3,3-trimethyl-1-[(trimethylsilyl)oxy]disiloxanyl

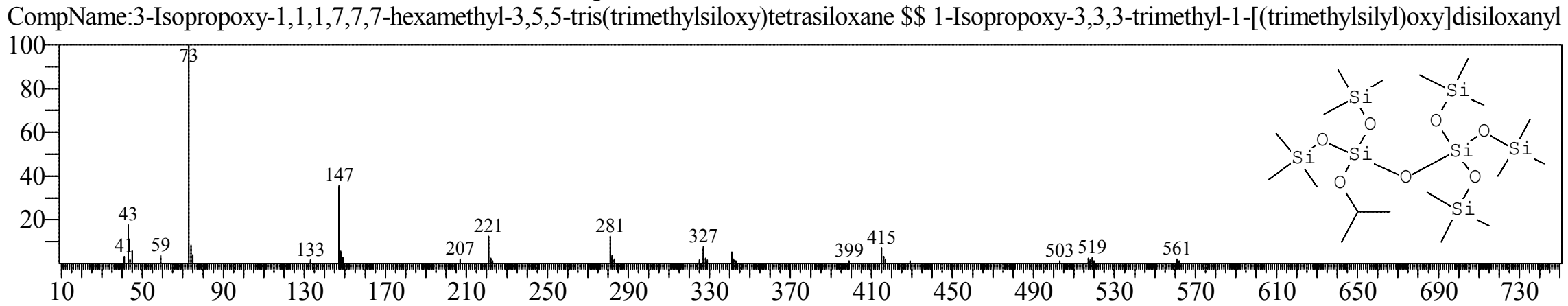

Hit#:15 Entry:310786 Library:WILEY7.LIB
SI:63 Formula:C20 H40 O5 SI4 CAS:37148-65-5 MolWeight:472 RetIndex:0
CompName:Benzeneacetic acid, .alpha.,3,4-tris[(trimethylsilyl)oxy]-, trimethylsilyl ester (CAS) TETRAKISTRIMETHYLSILYL 3,4-DIHYDROXYMAND

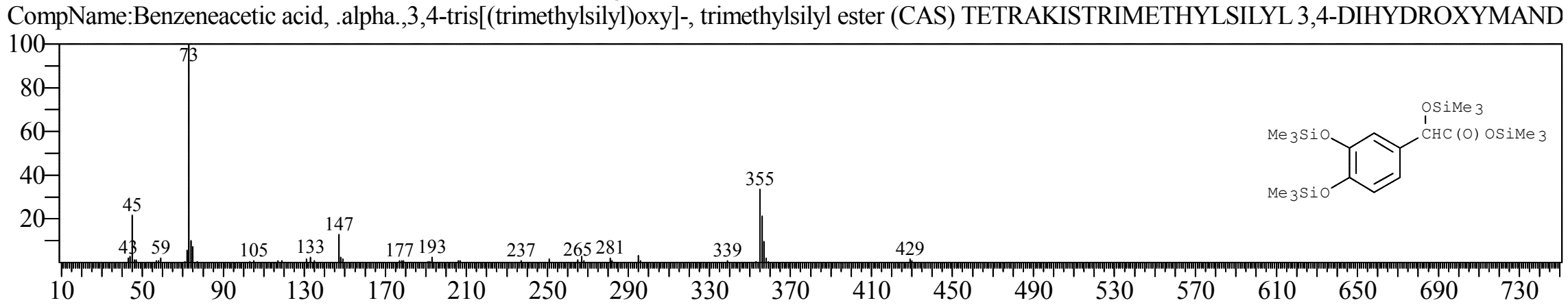

<< Target >>
Line#:25 R.Time:18.792(Scan#:1656) MassPeaks:59
RawMode:Averaged 18.783-18.800(1655-1657) BasePeak:73.05(6700)
BG Mode:Calc. from Peak Group 1 - Event 1 Scan

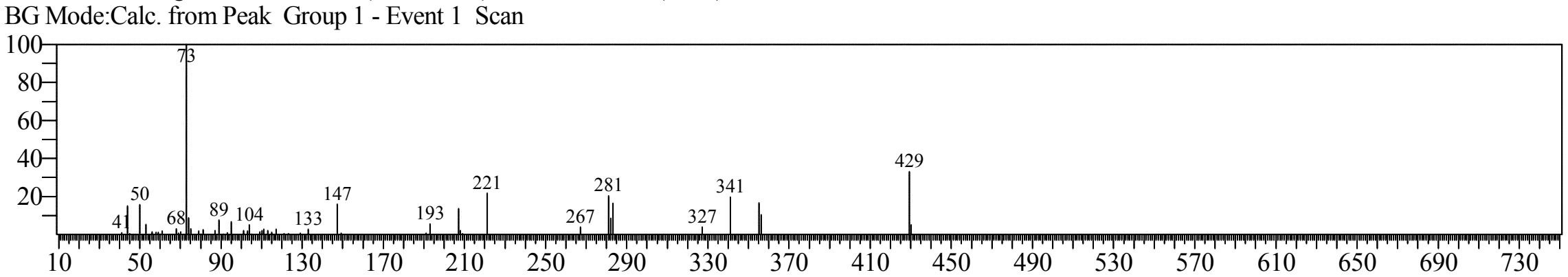

Hit#:16 Entry:156530 Library:NIST05.LIB
SI:62 Formula:C13H42O6Si7 CAS:50694-26-3 MolWeight:490 RetIndex:1570
CompName:1,1,1,3,5,7,9,11,11,11-Decamethyl-5-(trimethylsiloxy)hexasiloxane $$ 1,1,1,3,5,7,9,11,11,11-Decamethyl-5-[(trimethylsilyl)oxy]hexasiloxane #

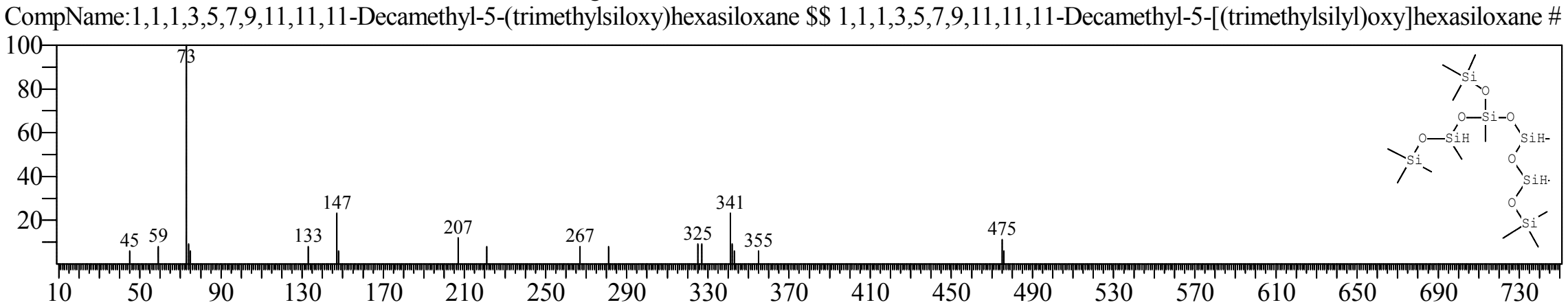

Hit#:17 Entry:338301 Library:WILEY7.LIB
SI:62 Formula: CAS:0-00-0 MolWeight:9999 RetIndex:0
CompName:Silicone grease, Siliconfett $$

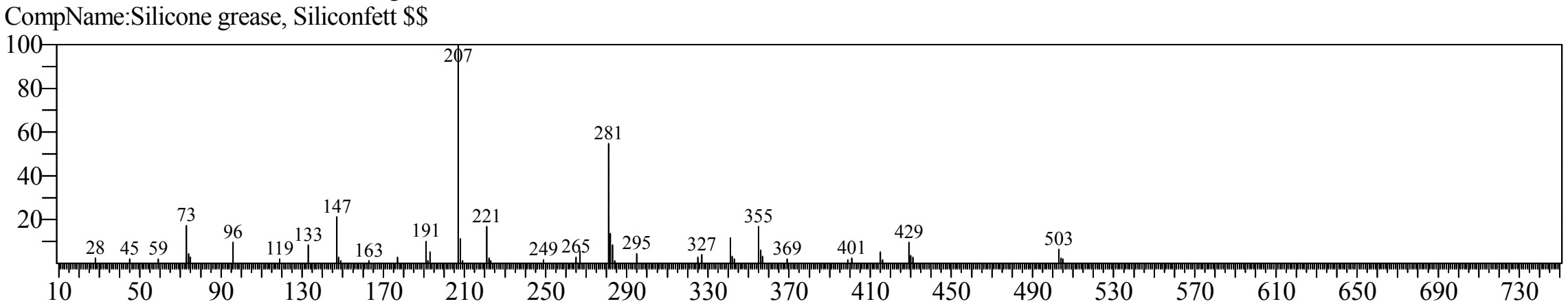

Hit#:18 Entry:121111 Library:WILEY7.LIB
SI:62 Formula:C16 H28 CAS:61177-15-9 MolWeight:220 RetIndex:0
CompName:Cyclobuta[1,2:3,4]dicyclooctene, hexadecahydro-, (6a.alpha.,6b.alpha.,12a.alpha.,12b.alpha.)- (CAS) TRICYCLO[8.6.0.0(2,9)]HEXADECAN

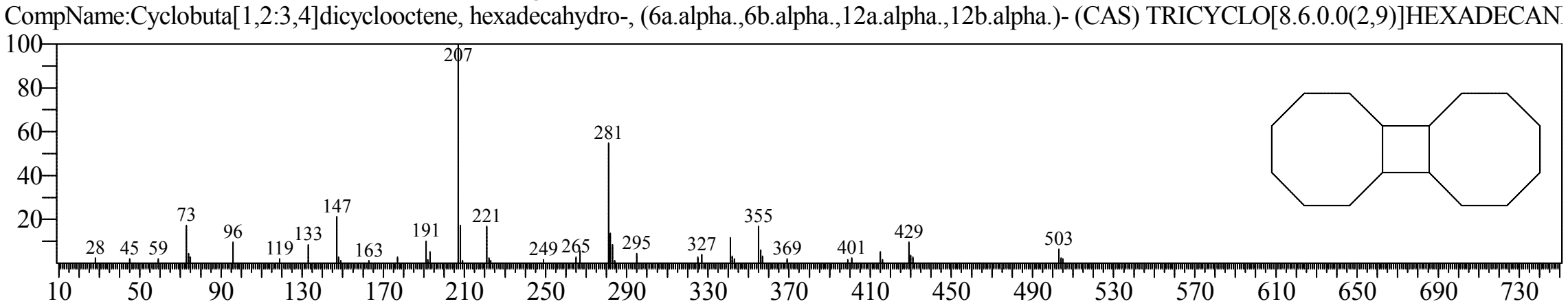

Hit#:19 Entry:161854 Library:NIST05.LIB
SI:62 Formula:C16H48O10Si9 CAS:145344-72-5 MolWeight:652 RetIndex:1716
CompName:2-(2',4',4',6',6',8',8'-Heptamethyltetrasiloxan-2'-yloxy)-2,4,4,6,6,8,8,10,10-nonamethylcyclopentasiloxane $$ 2-[(2,4,4,6,6,8,8-Heptamethyl-1,3,5

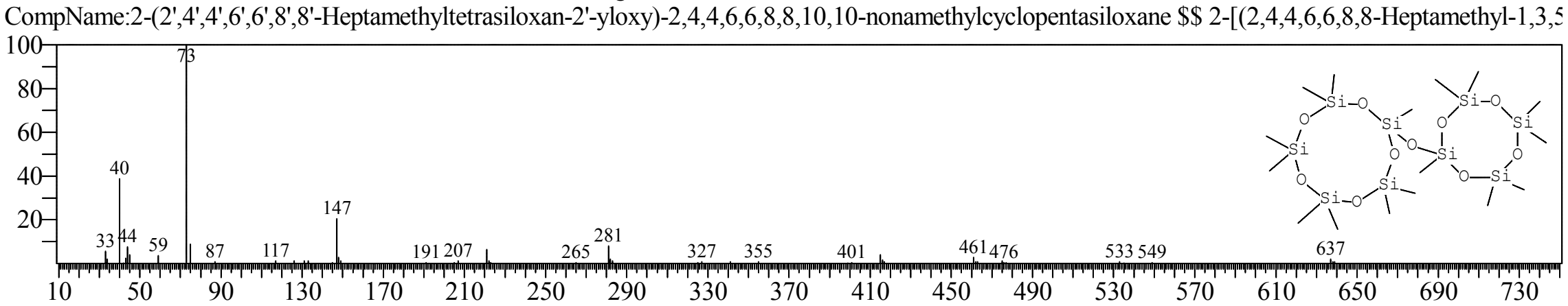

Hit#:20 Entry:158803 Library:NIST05.LIB
SI:61 Formula:C16H48O6Si7 CAS:34915-06-5 MolWeight:532 RetIndex:1437
CompName:1,1,1,5,7,7,7-Heptamethyl-3,3,5-tris(trimethylsiloxy)tetrasiloxane $$ 1,3,3,3-Tetramethyl-1-[(trimethylsilyl)oxy]disiloxanyl tris(trimethylsilyl) o

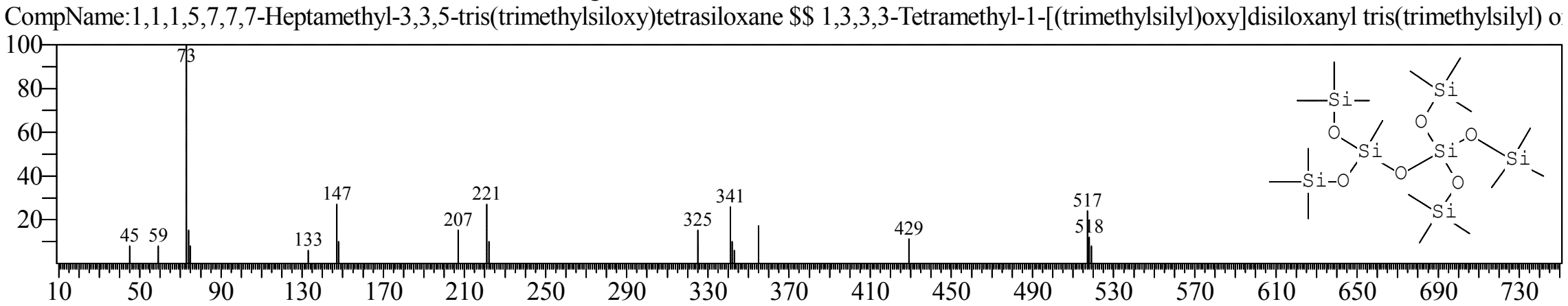

<< Target >>
Line#:26 R.Time:21.317(Scan#:1959) MassPeaks:46
RawMode:Averaged 21.308-21.325(1958-1960) BasePeak:44.00(1473)
BG Mode:Calc. from Peak Group 1 - Event 1 Scan

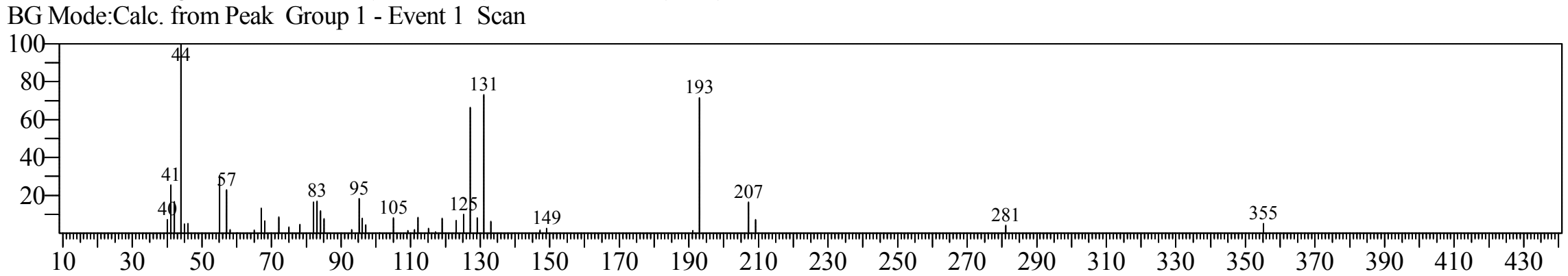

Hit#:1 Entry:141906 Library:WILEY7.LIB
SI:59 Formula:C8 H16 N O5 P CAS:141-66-2 MolWeight:237 RetIndex:0
CompName:Bidrin $$ Phosphoric acid, 3-(dimethylamino)-1-methyl-3-oxo-1-propenyl dimethyl ester, (E)- (CAS) SD 3562 $$ ENT 24,482 $$ C 709 $$ Ekt

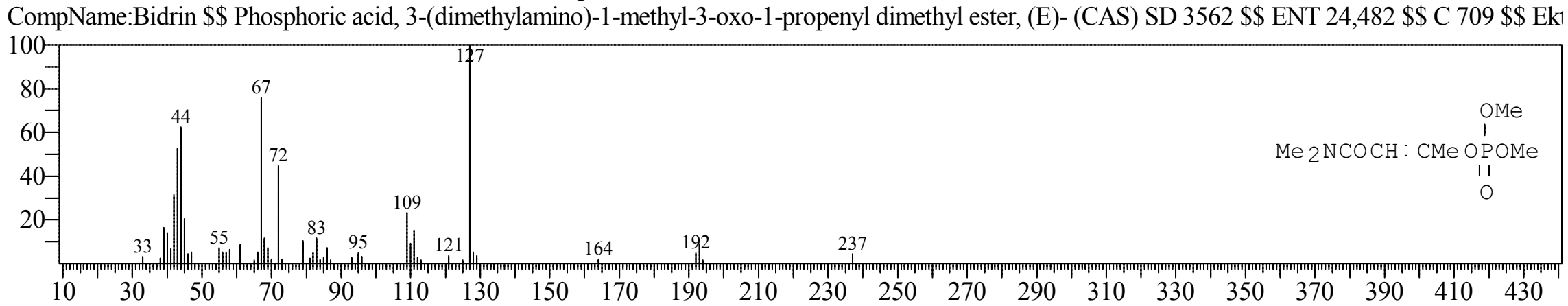

Hit#:2 Entry:6717 Library:NIST05.LIB
SI:58 Formula:C7H13NO CAS:1985-48-4 MolWeight:127 RetIndex:1064
CompName:2H-Azepin-2-one, hexahydro-7-methyl- $$ .epsilon.-Methylcaprolactam $$ 6-Methyl-6-caprolactam $$ 7-Methylcaprolactam $$ Hexahydro-7-r

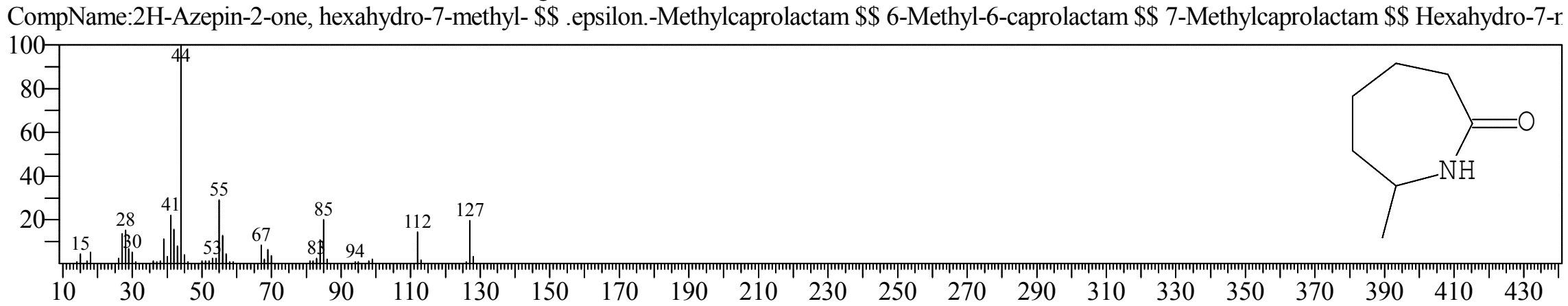

Hit#:3 Entry:4748 Library:NIST05s.LIB
SI:58 Formula:C7H13NO CAS:2556-73-2 MolWeight:127 RetIndex:1160
CompName:2H-Azepin-2-one, hexahydro-1-methyl- $$ N-Methyl-.epsilon.-caprolactam $$ N-Methylcaprolactam $$ 1-Methylcaprolactam $$ Hexahydro-1-

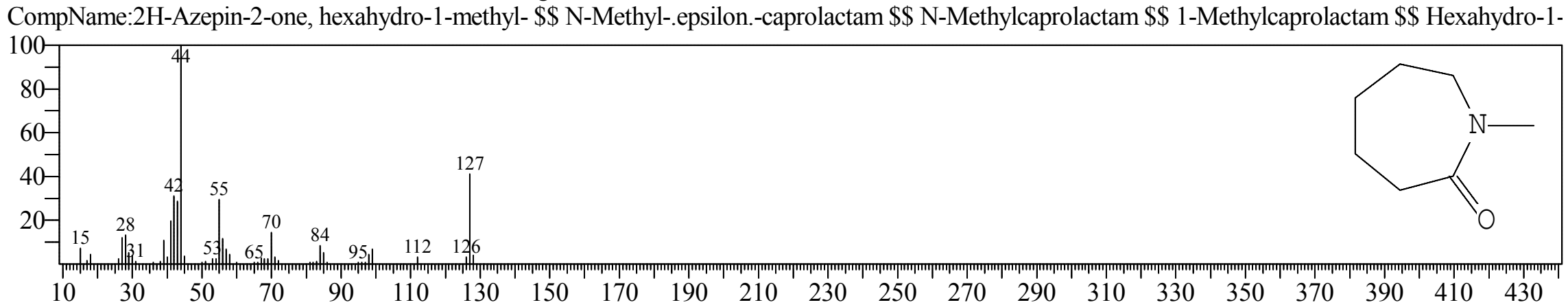

Hit#:4 Entry:11673 Library:NIST05.LIB
SI:58 Formula:C7H14N2O CAS:35216-55-8 MolWeight:142 RetIndex:1157
CompName:Aziridine-2-carboxamide, 1-(1,1-dimethylethyl)- $$ Aziridine-2-carboxamide, 1-tert-butyl- $$ 1-tert-Butyl-2-aziridinecarboxamide # $$

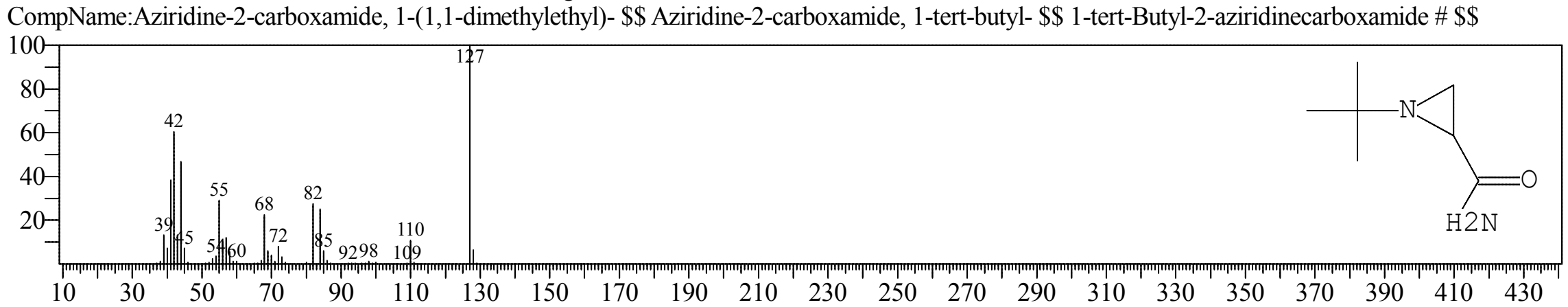

Hit#:5 Entry:6765 Library:NIST05.LIB
SI:56 Formula:C8H17N CAS:13603-18-4 MolWeight:127 RetIndex:1061
CompName:Piperidine, 3-isopropyl- $$ 3-Isopropylpiperidine # $$

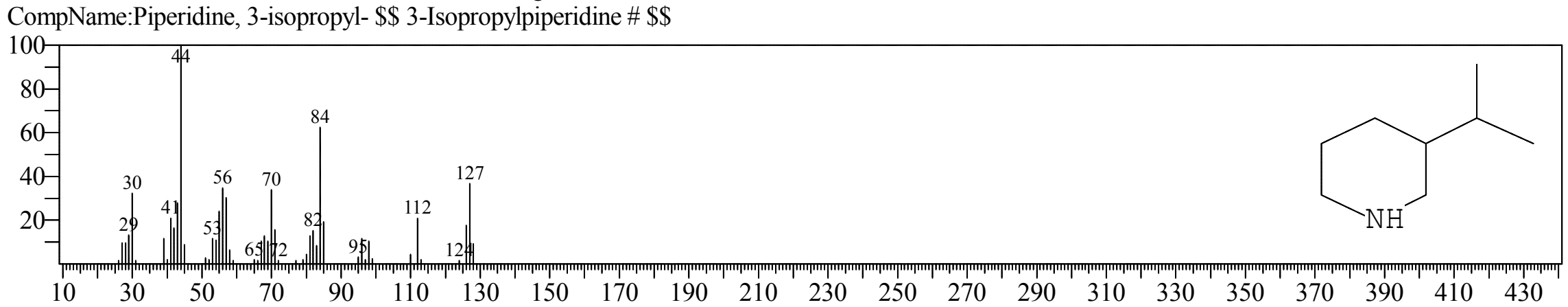

<< Target >>
Line#:26 R.Time:21.317(Scan#:1959) MassPeaks:46
RawMode:Averaged 21.308-21.325(1958-1960) BasePeak:44.00(1473)
BG Mode:Calc. from Peak Group 1 - Event 1 Scan

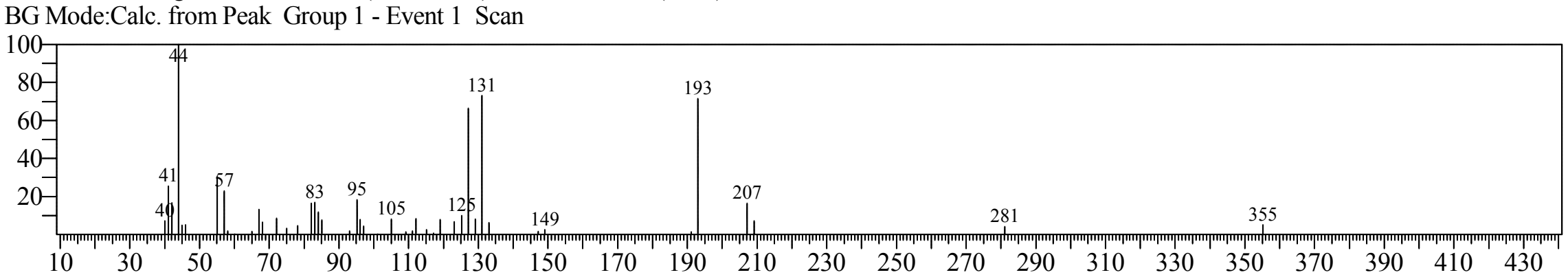

Hit#:6 Entry:19299 Library:WILEY7.LIB
SI:56 Formula:C8 H17 N CAS:0-00-0 MolWeight:127 RetIndex:0
CompName:

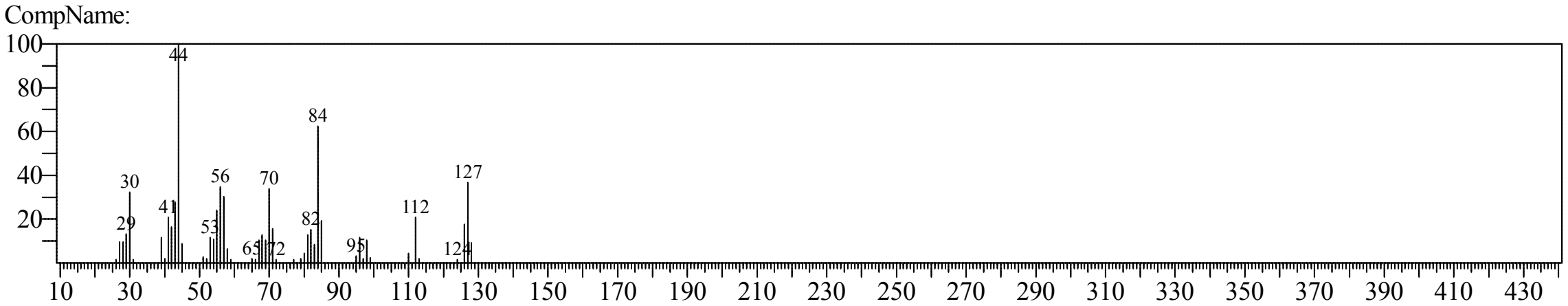

Hit#:7 Entry:26801 Library:NIST11.lib
SI:55 Formula:C8H16N2O2 CAS:0-00-0 MolWeight:172 RetIndex:1310
CompName:L-Valine, N-dimethylaminomethylene-

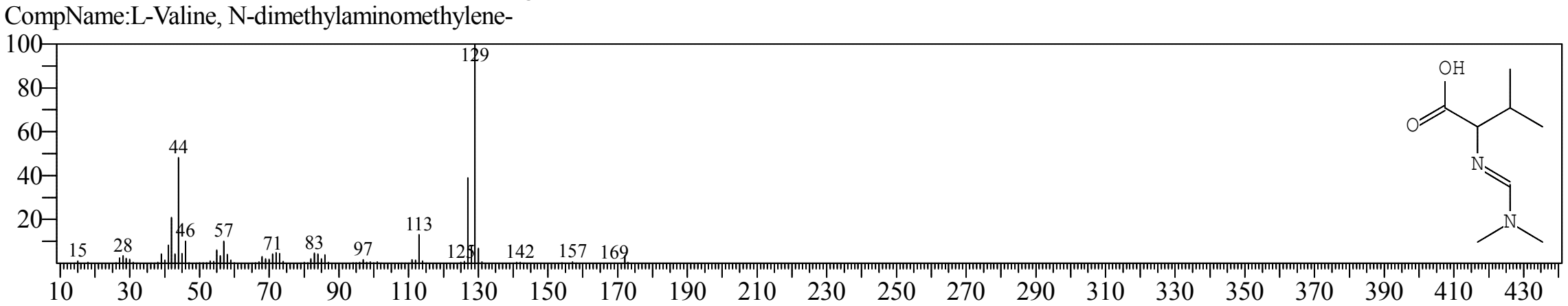

Hit#:8 Entry:300680 Library:WILEY7.LIB
SI:55 Formula:C29 H58 O2 CAS:55320-07-5 MolWeight:438 RetIndex:0
CompName:Heptanoic acid, docosyl ester (CAS) N-DOCOSYL N-HEPTANOATE $$

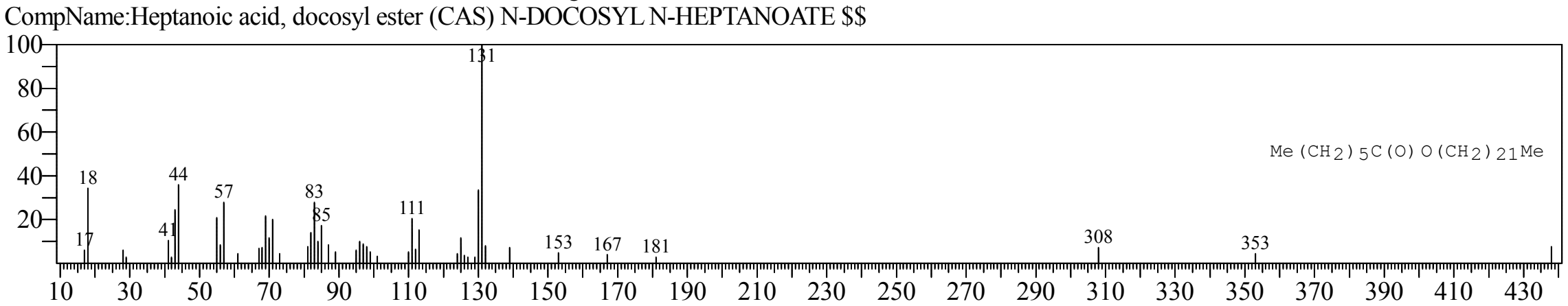

Hit#:9 Entry:26264 Library:NIST11.lib
SI:55 Formula:C7H13N3O2 CAS:0-00-0 MolWeight:171 RetIndex:1712
CompName:1,3(2H)-Pyridinedicarboxamide, tetrahydro-

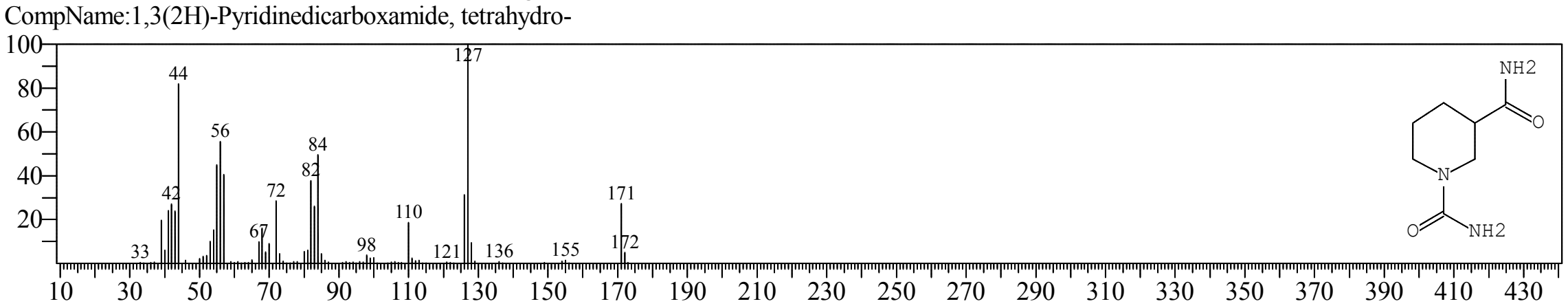

Hit#:10 Entry:19445 Library:WILEY7.LIB
SI:55 Formula:C7 H13 N O CAS:3433-62-3 MolWeight:127 RetIndex:0
CompName:3-Penten-2-one, 4-(dimethylamino)- (CAS) 4-DIMETHYLAMINO-3-PENTENE-2-ONE $$

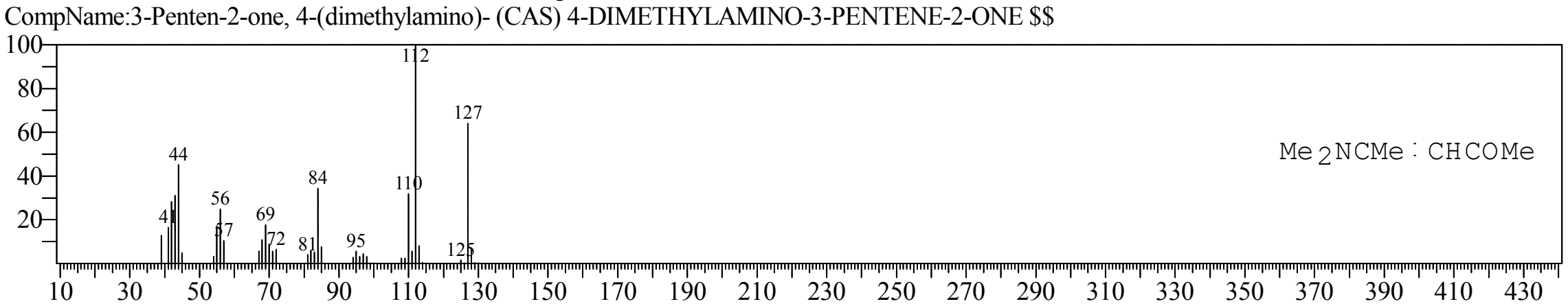

<< Target >>
Line#:26 R.Time:21.317(Scan#:1959) MassPeaks:46
RawMode:Averaged 21.308-21.325(1958-1960) BasePeak:44.00(1473)
BG Mode:Calc. from Peak Group 1 - Event 1 Scan

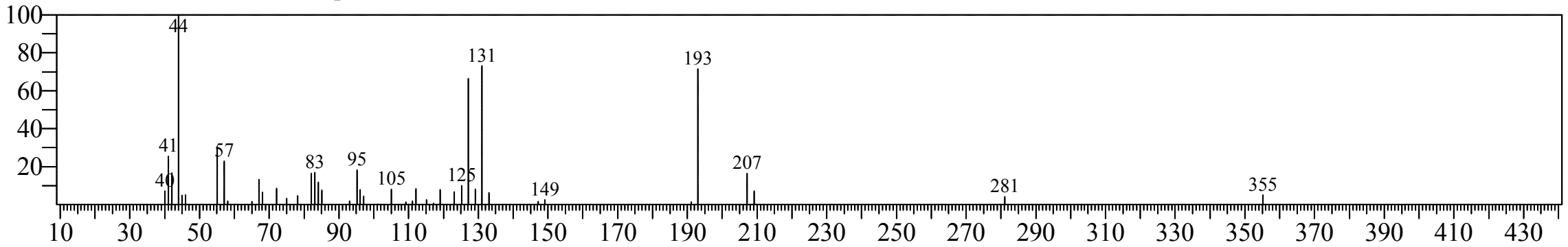

Hit#:11 Entry:7465 Library:NIST11.lib
SI:55 Formula:C3H3N3O3 CAS:0-00-0 MolWeight:129 RetIndex:1420
CompName:1H-1,2,4-Triazole-3-carboxylic acid, 5-hydroxy-

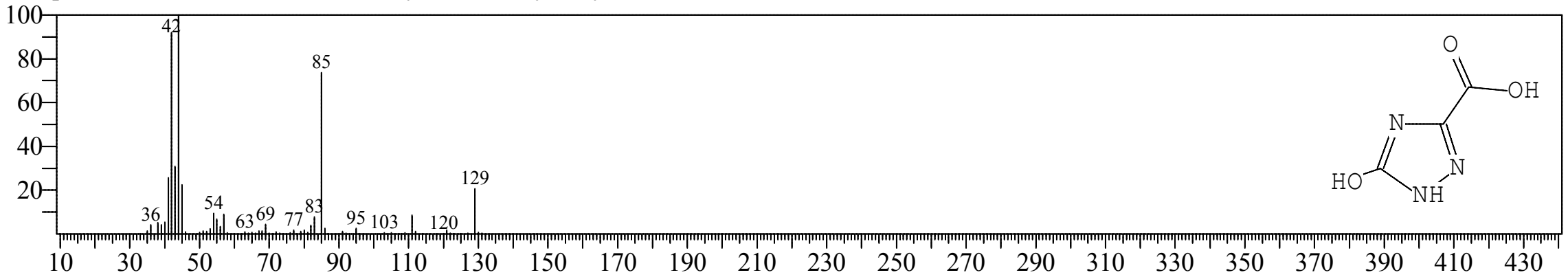

Hit#:12 Entry:32965 Library:NIST05.LIB
SI:54 Formula:C8H14N2O3 CAS:0-00-0 MolWeight:186 RetIndex:1424
CompName:Propanoic acid, 2-(2-aminocarbonyl-1-aziridinyl)-2-methyl-, methyl ester

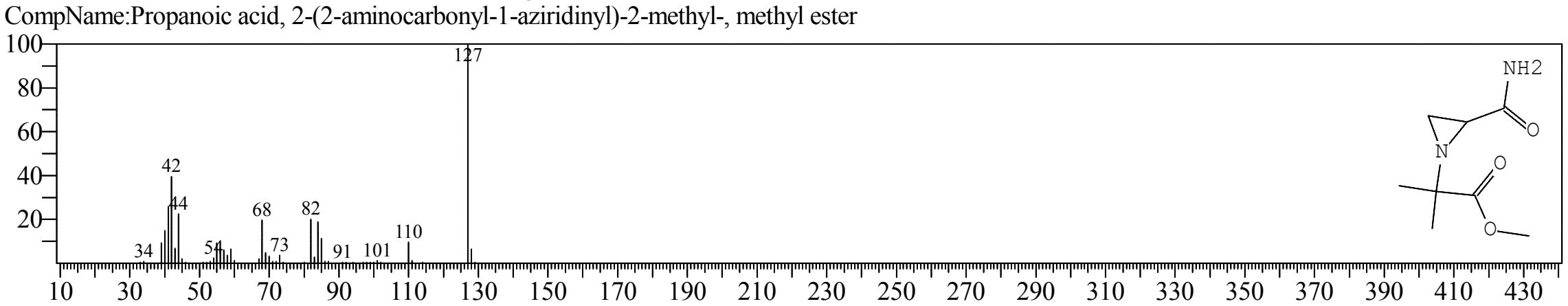

Hit#:13 Entry:6634 Library:NIST05.LIB
SI:54 Formula:C4H5N3O2 CAS:0-00-0 MolWeight:127 RetIndex:1241
CompName:4-Hydroxy-2-hydroxyaminopyrimidine

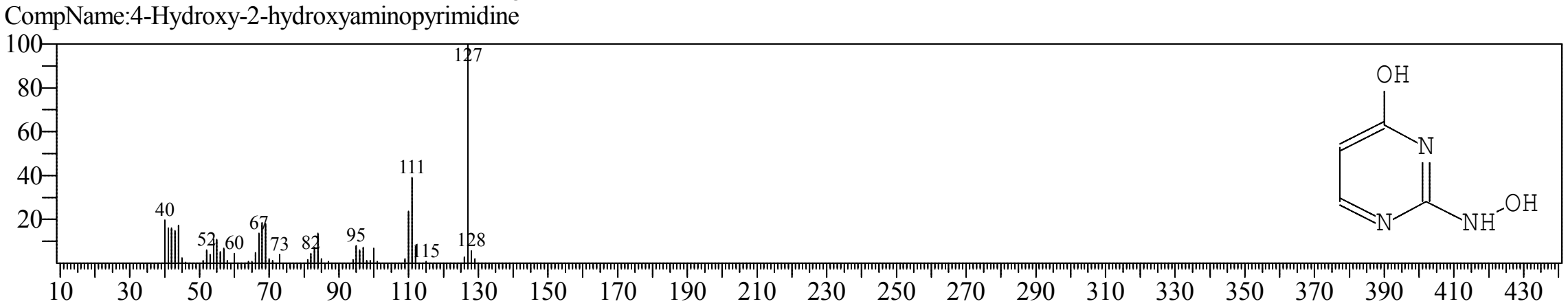

Hit#:14 Entry:6770 Library:NIST11.lib
SI:54 Formula:C4H5N3O2 CAS:0-00-0 MolWeight:127 RetIndex:1241
CompName:4-Hydroxy-2-hydroxyaminopyrimidine $$ 2-(Hydroxyamino)-4-pyrimidinol # $$

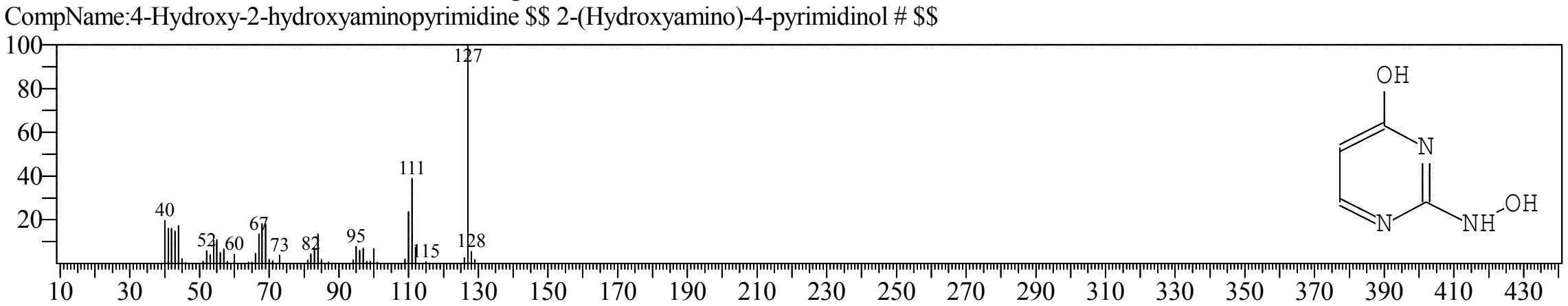

Hit#:15 Entry:34771 Library:NIST11.lib
SI:54 Formula:C8H14N2O3 CAS:0-00-0 MolWeight:186 RetIndex:1424
CompName:Propanoic acid, 2-(2-aminocarbonyl-1-aziridinyl)-2-methyl-, methyl ester $$ Methyl 2-[2-(aminocarbonyl)-1-aziridinyl]-2-methylpropanoate # $

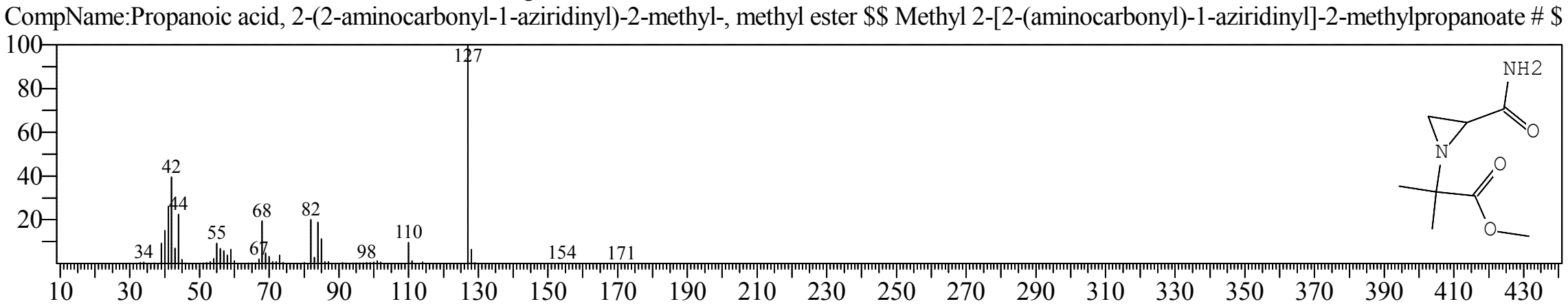

<< Target >>
Line#:26 R.Time:21.317(Scan#:1959) MassPeaks:46
RawMode:Averaged 21.308-21.325(1958-1960) BasePeak:44.00(1473)
BG Mode:Calc. from Peak Group 1 - Event 1 Scan

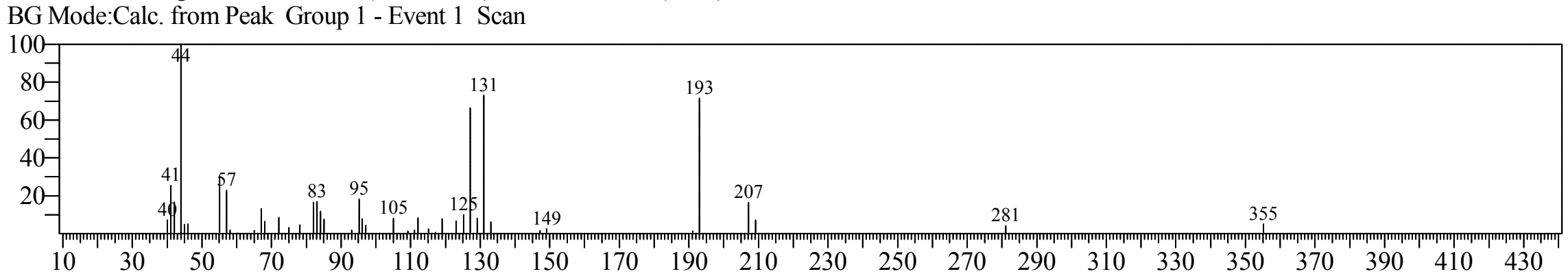

Hit#:16 Entry:48546 Library:NIST05.LIB
SI:54 Formula:C14H28O CAS:40565-00-2 MolWeight:212 RetIndex:1562
CompName:Cycloheptanol, 6-(3,3-dimethylbutyl)-2-methyl- $$ 6-(3,3-Dimethylbutyl)-2-methylcycloheptanol # $$

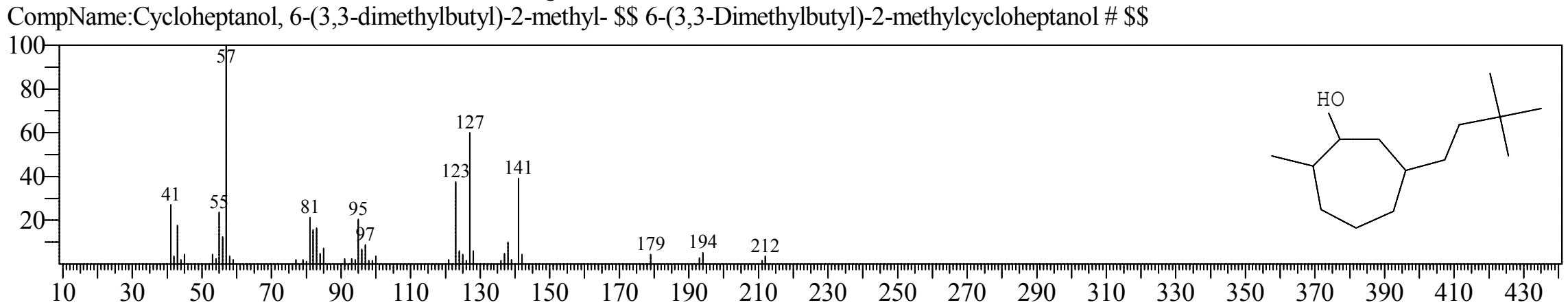

Hit#:17 Entry:29381 Library:NIST11.lib
SI:54 Formula:C5H11N3O4 CAS:0-00-0 MolWeight:177 RetIndex:1396
CompName:Butanamine, 2,2-dinitro-N-methyl-

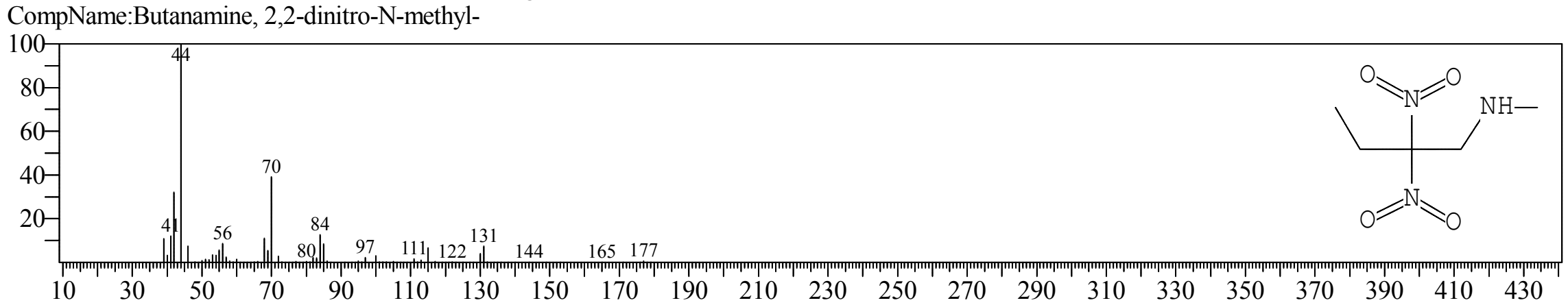

Hit#:18 Entry:28000 Library:NIST05.LIB
SI:54 Formula:C5H11N3O4 CAS:0-00-0 MolWeight:177 RetIndex:1396
CompName:Butanamine, 2,2-dinitro-N-methyl-

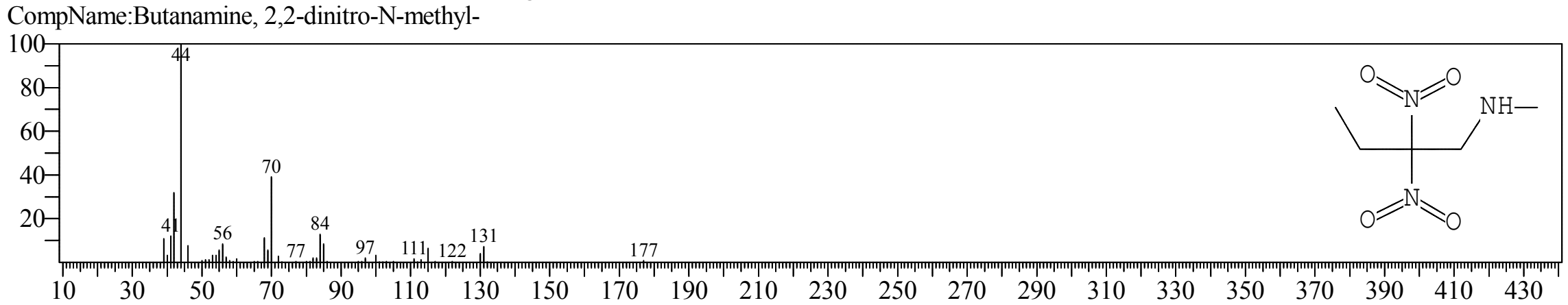

Hit#:19 Entry:92563 Library:NIST05.LIB
SI:53 Formula:C12H15FN2O3S CAS:309279-85-4 MolWeight:286 RetIndex:2358
CompName:Piperidine-4-carboxamide, 1-(4-fluorophenylsulfonyl)- $$ 1-[(4-Fluorophenyl)sulfonyl]-4-piperidinecarboxamide # $$

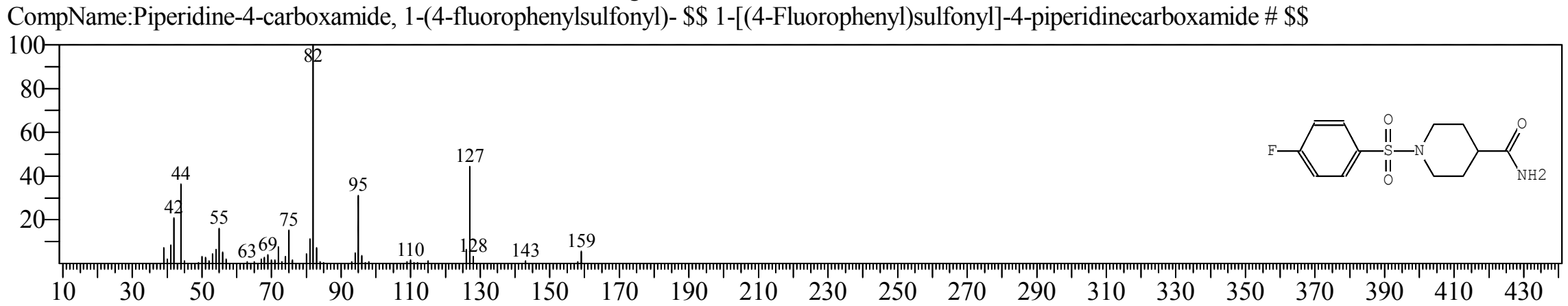

Hit#:20 Entry:19616 Library:WILEY7.LIB
SI:53 Formula:C6 H8 O3 CAS:50767-52-7 MolWeight:128 RetIndex:0
CompName:1,6:3,4-Dianhydro-2-deoxy-.beta.-D-ribo-hexopyranose $$ .beta.-D-ribo-Hexopyranose, 1,6:3,4-dianhydro-2-deoxy- (CAS) 3,7,9-Trioxatricyclc

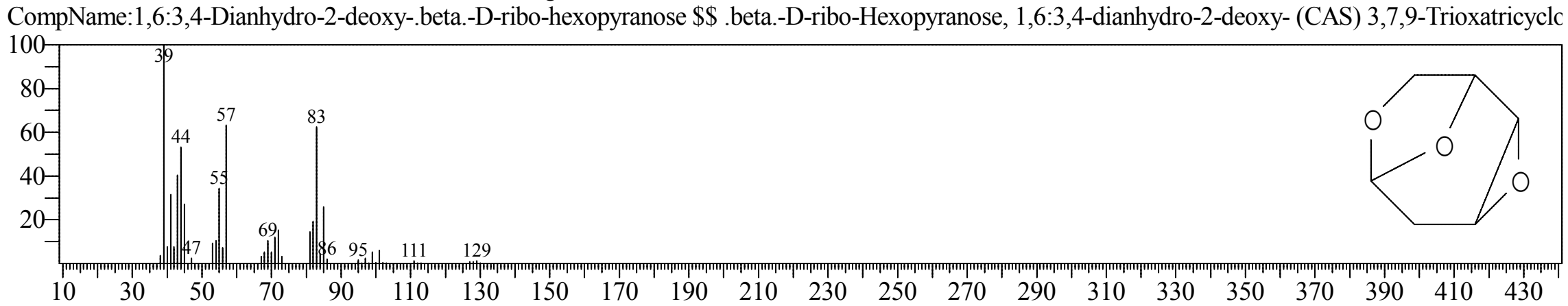

<< Target >>
Line#:27   R.Time:21.550(Scan#:1987) MassPeaks:72
RawMode:Averaged 21.542-21.558(1986-1988) BasePeak:40.00(2236)
BG Mode:Calc. from Peak Group 1 - Event 1 Scan

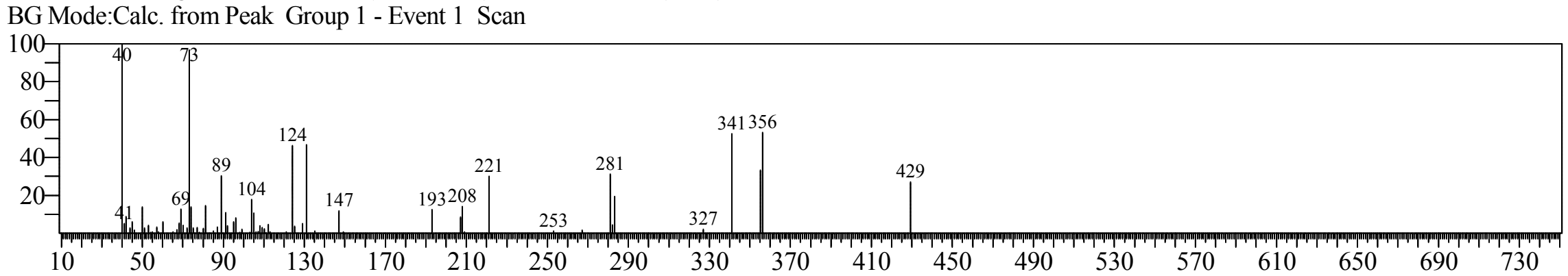

Hit#:1 Entry:338296 Library:WILEY7.LIB
SI:55 Formula: CAS:0-00-0 MolWeight:9999 RetIndex:0
CompName:SILIKONFETT SE30 (GREVELS) $$ Silicone oil $$

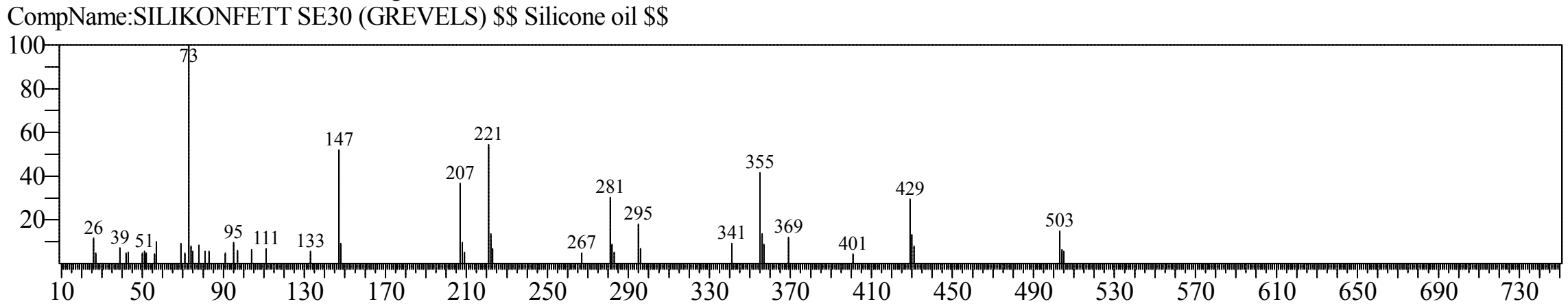

Hit#:2 Entry:300653 Library:WILEY7.LIB
SI:55 Formula:C21 H22 FE N2 O5 CAS:109007-87-6 MolWeight:438 RetIndex:0
CompName:Iron, monocarbonyl-(1,3-butadiene-1,4-dicarbonic acid, diethyl ester) a,a'-dipyridyl $$ IRON, (2,2'-BIPYRIDINE-N,N')CARBONYL[(2,3,4,5-.

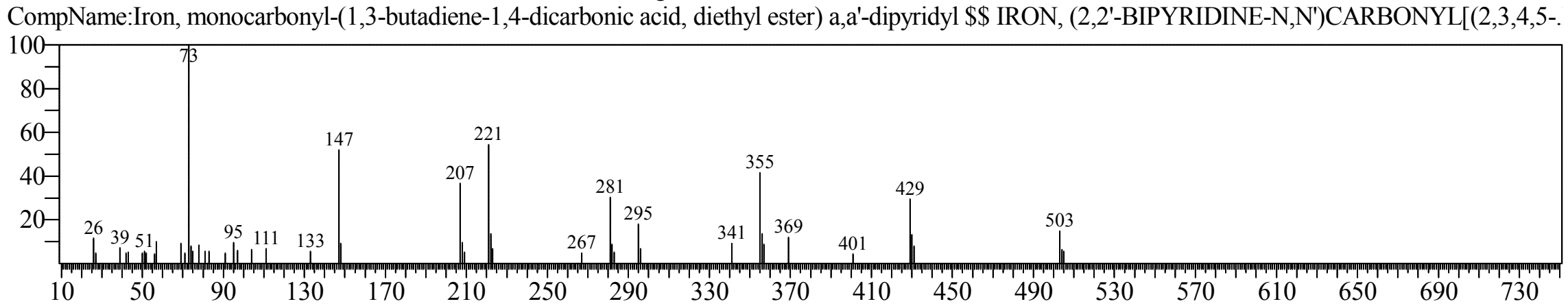

Hit#:3 Entry:27443 Library:NIST05s.LIB
SI:52 Formula:C24H34F5NO3Si3 CAS:55429-13-5 MolWeight:563 RetIndex:2572
CompName:Benzeneethanamine, N-[(pentafluorophenyl)methylene]-.beta.,3,4-tris[(trimethylsilyl)oxy]- $$ N-(Pentafluorobenzylidene)-.beta.,3,4-tris(trimeth

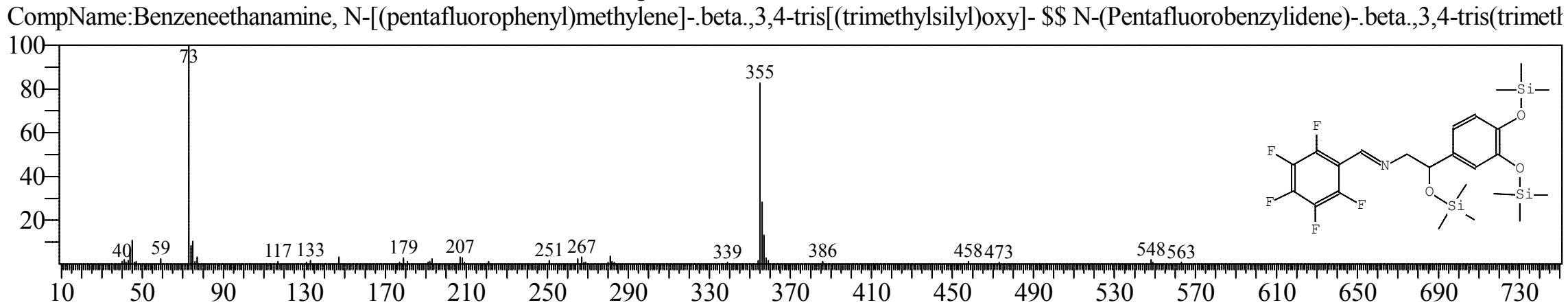

Hit#:4 Entry:26522 Library:NIST05s.LIB
SI:52 Formula:C18H37NO3Si3 CAS:10538-85-9 MolWeight:399 RetIndex:2007
CompName:Phenethylamine, N-methyl-.beta.,3,4-tris(trimethylsiloxy)- $$ 2-(3,4-Bis[(trimethylsilyl)oxy]phenyl)-N-methyl-2-[(trimethylsilyl)oxy]ethanamine

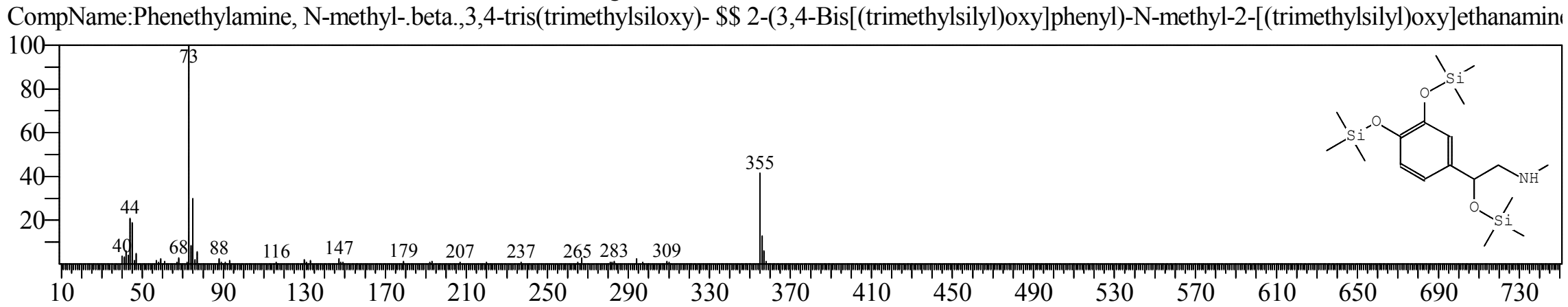

Hit#:5 Entry:27179 Library:NIST05s.LIB
SI:51 Formula:C20H40O5Si4 CAS:37148-65-5 MolWeight:472 RetIndex:2084
CompName:Benzeneacetic acid, .alpha.,3,4-tris[(trimethylsilyl)oxy]-, trimethylsilyl ester $$ 3,4-Dihydroxymadelic acid (tms) $$ Trimethylsilyl 3,4-dihydrox

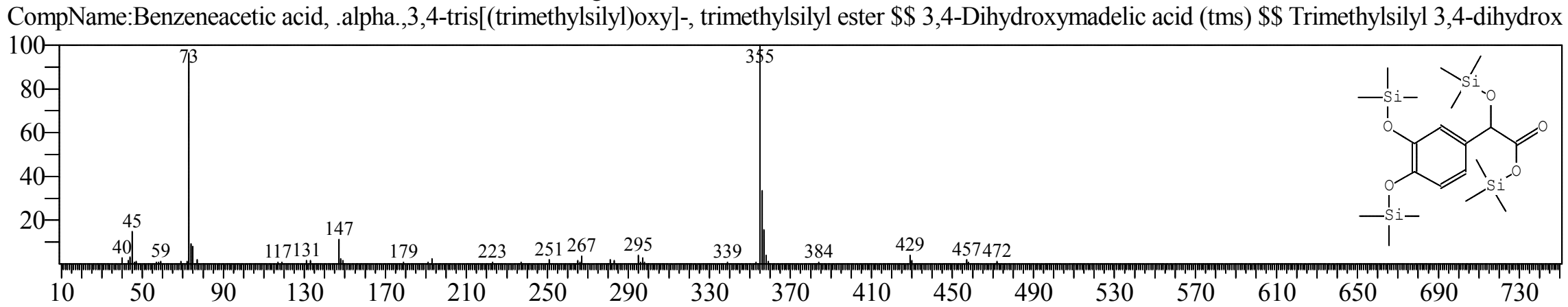

<< Target >>
Line#:27 R.Time:21.550(Scan#:1987) MassPeaks:72
RawMode:Averaged 21.542-21.558(1986-1988) BasePeak:40.00(2236)
BG Mode:Calc. from Peak Group 1 - Event 1 Scan

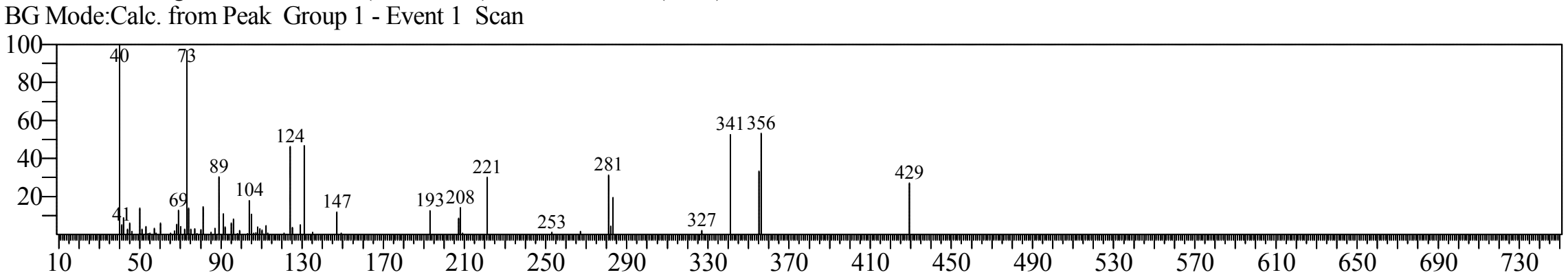

Hit#:6 Entry:153730 Library:NIST05.LIB
SI:51 Formula:C20H42O4Si4 CAS:56114-62-6 MolWeight:458 RetIndex:1993
CompName:Silane, [[4-[1,2-bis[(trimethylsilyl)oxy]ethyl]-1,2-phenylene]bis(oxy)]bis[trimethyl- $$ Silane, [[4-[1,2-bis[(trimethylsilyl)oxy]ethyl]-1,2-phenyl

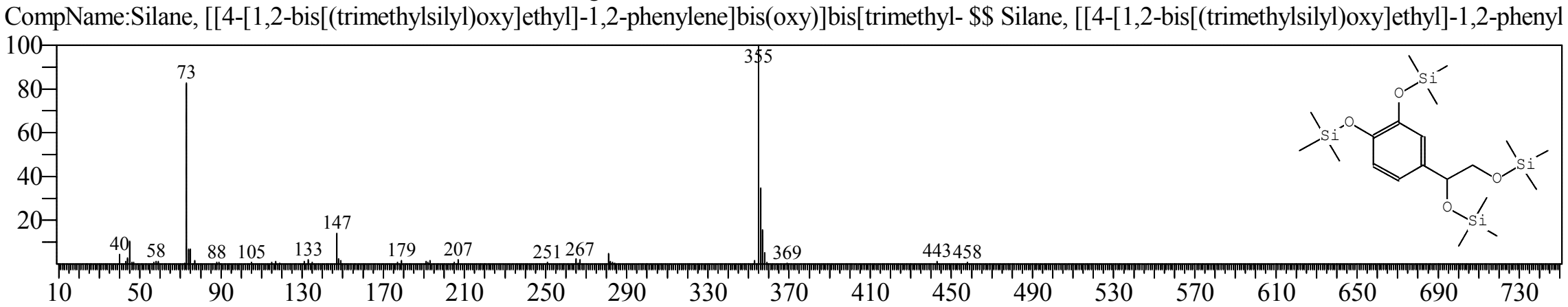

Hit#:7 Entry:148639 Library:WILEY7.LIB
SI:51 Formula:C12 H10 F N5 CAS:74421-44-6 MolWeight:243 RetIndex:0
CompName:1H-Purin-6-amine, [(2-fluorophenyl)methyl]- (CAS)

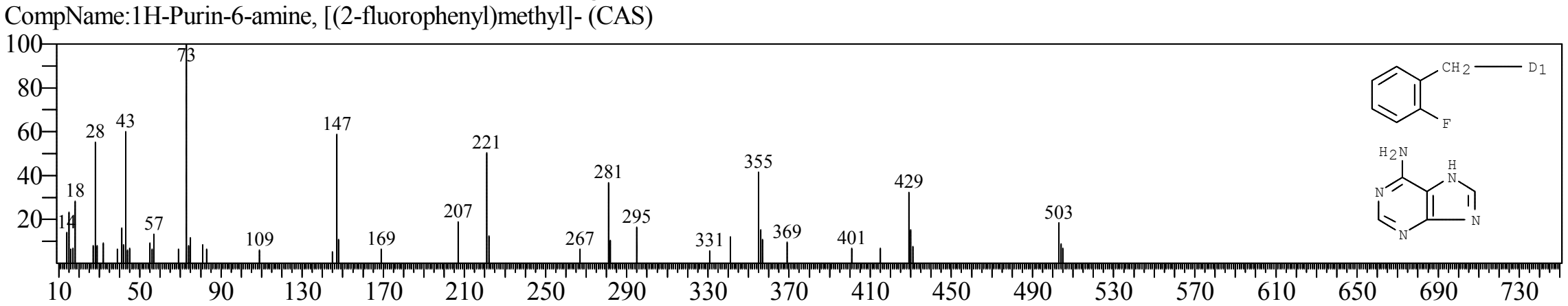

Hit#:8 Entry:160407 Library:NIST05.LIB
SI:50 Formula:C16H50O7Si8 CAS:19095-24-0 MolWeight:578 RetIndex:1710
CompName:Octasiloxane, 1,1,3,3,5,5,7,7,9,9,11,11,13,13,15,15-hexadecamethyl- $$ 1,1,3,3,5,5,7,7,9,9,11,11,13,13,15,15-Hexadecamethyloctasiloxane # $

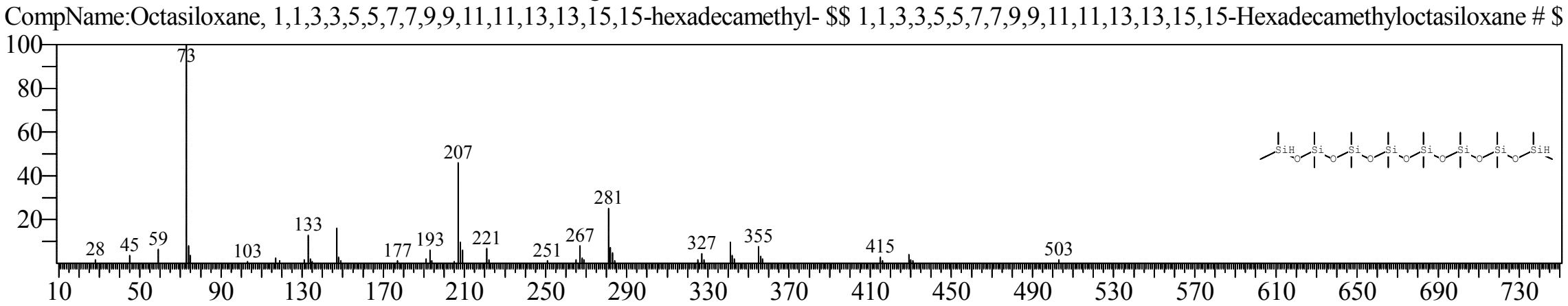

Hit#:9 Entry:27563 Library:NIST05s.LIB
SI:50 Formula:C18H54O9Si9 CAS:556-71-8 MolWeight:666 RetIndex:1860
CompName:Cyclononasiloxane, octadecamethyl- $$ Octadeamethyl-cyclononasiloxane $$ 2,2,4,4,6,6,8,8,10,10,12,12,14,14,16,16,18,18-Octadecamethylcy

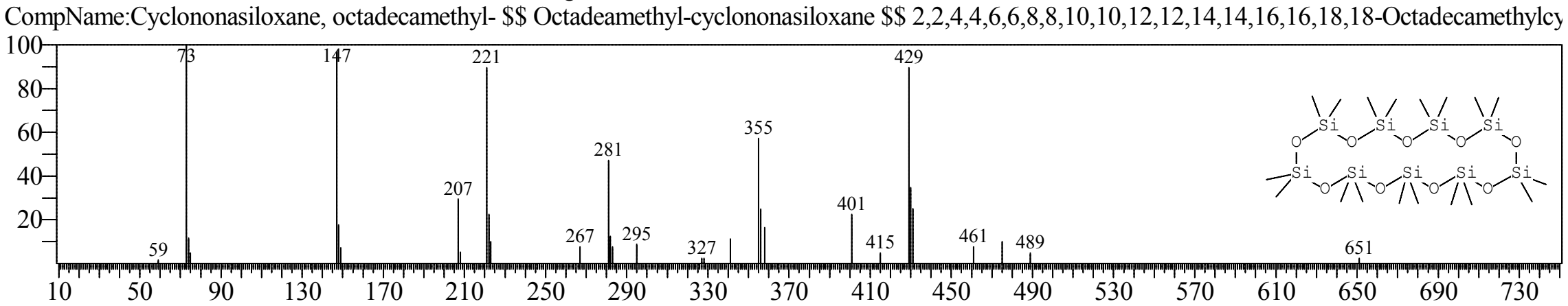

Hit#:10 Entry:191402 Library:NIST11.lib
SI:50 Formula:C19H36O5Si3 CAS:0-00-0 MolWeight:428 RetIndex:2075
CompName:3,4-Dihydroxymandelic acid, ethyl ester, tri-TMS $$ Ethyl (3,4-bis[(trimethylsilyl)oxy]phenyl)[(trimethylsilyl)oxy]acetate # $$

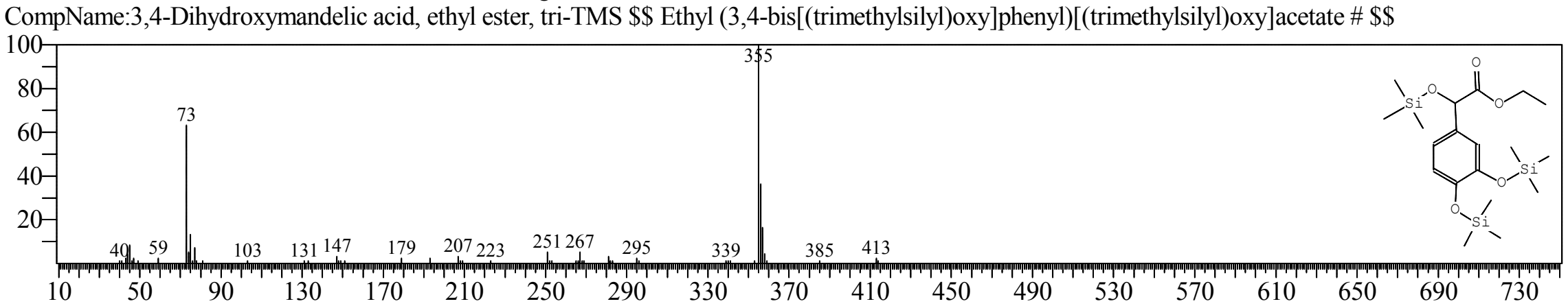

<< Target >>
Line#:27 R.Time:21.550(Scan#:1987) MassPeaks:72
RawMode:Averaged 21.542-21.558(1986-1988) BasePeak:40.00(2236)
BG Mode:Calc. from Peak Group 1 - Event 1 Scan

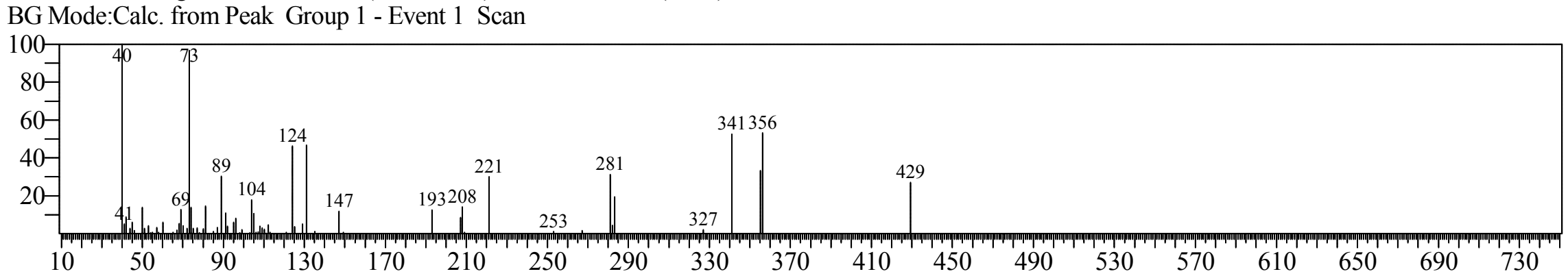

Hit#:11 Entry:149910 Library:NIST05.LIB
SI:50 Formula:C19H36O5Si3 CAS:0-00-0 MolWeight:428 RetIndex:2075
CompName:3,4-Dihydroxymandelic acid, ethyl ester, tri-TMS

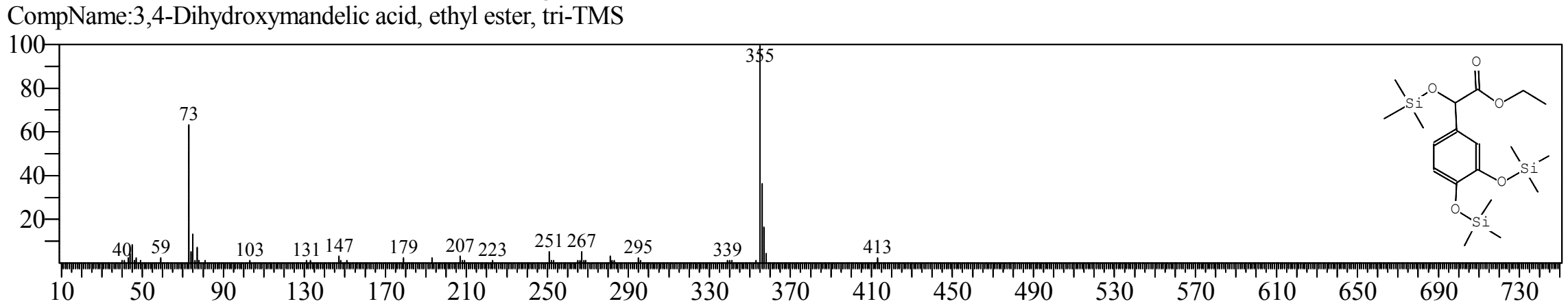

Hit#:12 Entry:335377 Library:WILEY7.LIB
SI:50 Formula:C20 H60 O10 SI10 CAS:18772-36-6 MolWeight:740 RetIndex:0
CompName:EICOSAMETHYLCYCLODECASILOXANE $$ CYCLODECASILOXANE, EICOSAMETHYL- $$

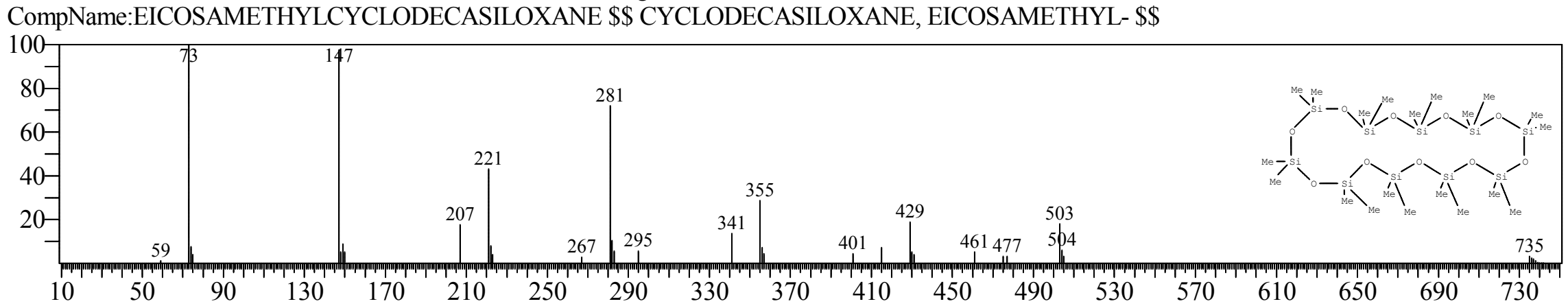

Hit#:13 Entry:337317 Library:WILEY7.LIB
SI:49 Formula:C24 H72 O12 SI12 CAS:18919-94-3 MolWeight:888 RetIndex:0
CompName:TETRACOSAMETHYLCYCLODODECASILOXANE $$ Cyclododecasiloxane, tetracosamethyl- (CAS)

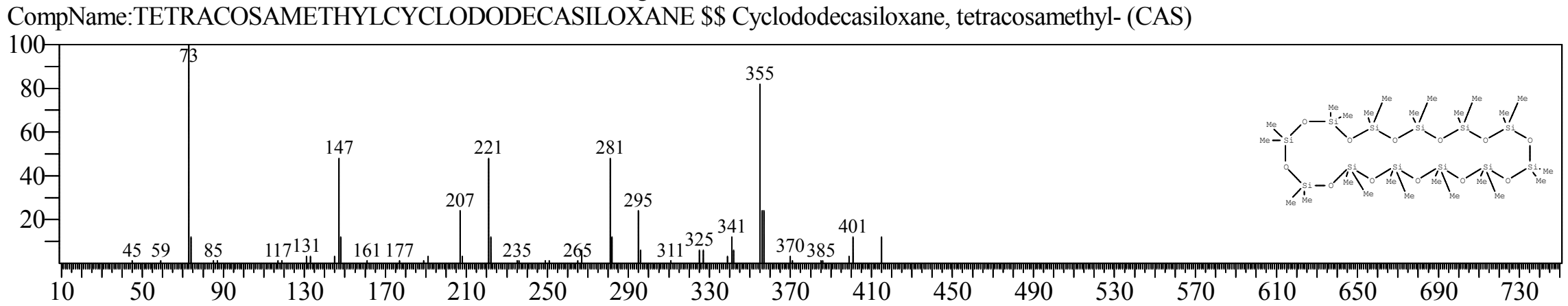

Hit#:14 Entry:317266 Library:WILEY7.LIB
SI:49 Formula:C14 H44 O6 SI7 CAS:0-00-0 MolWeight:504 RetIndex:0
CompName:1,1,3,3,5,5,7,7,9,9,11,11,13,13-TETRADECAMETHYL-HEPTASILOXANE $$

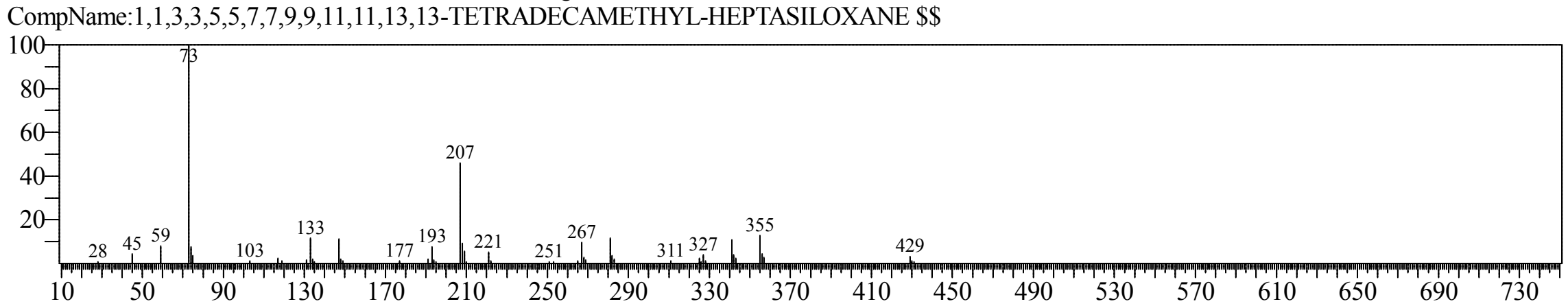

Hit#:15 Entry:157431 Library:NIST05.LIB
SI:49 Formula:C14H44O6Si7 CAS:19095-23-9 MolWeight:504 RetIndex:1526
CompName:Heptasiloxane, 1,1,3,3,5,5,7,7,9,9,11,11,13,13-tetradecamethyl- $$ 1,1,3,3,5,5,7,7,9,9,11,11,13,13-Tetradecamethylheptasiloxane # $$

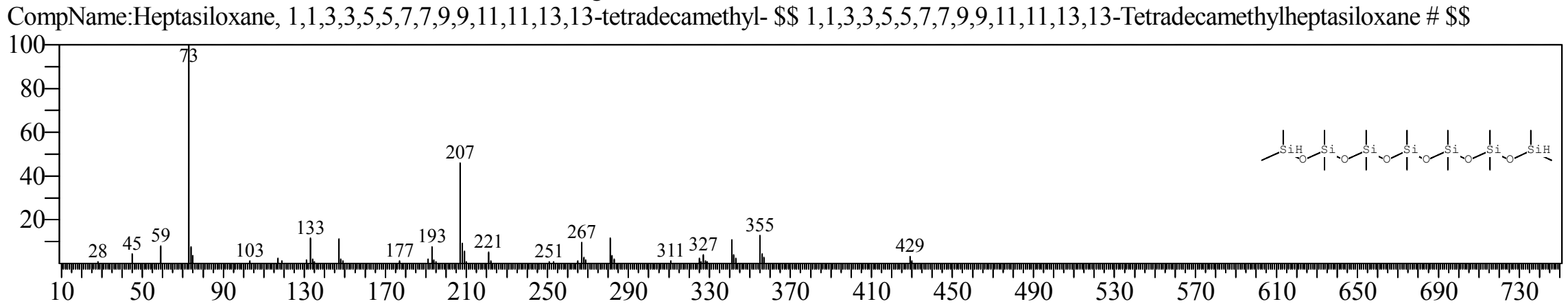

<< Target >>
Line#:27 R.Time:21.550(Scan#:1987) MassPeaks:72
RawMode:Averaged 21.542-21.558(1986-1988) BasePeak:40.00(2236)
BG Mode:Calc. from Peak Group 1 - Event 1 Scan

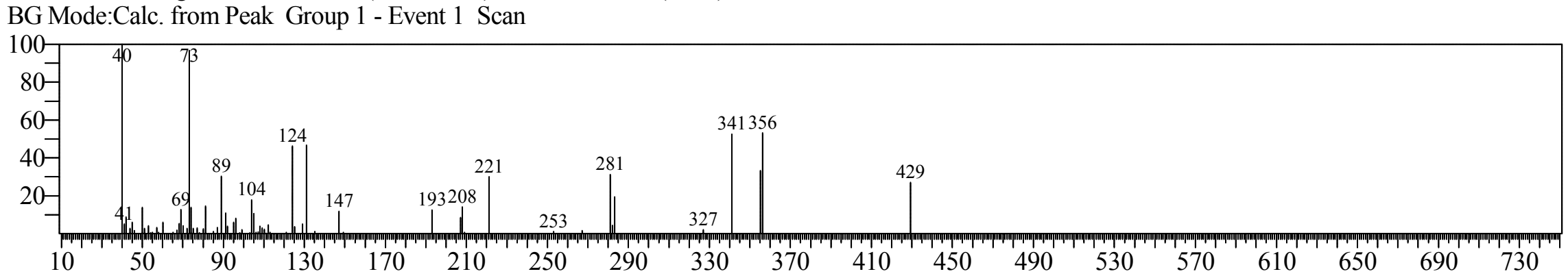

Hit#:16 Entry:156882 Library:NIST05.LIB
SI:49 Formula:C20H36F3NO4Si3 CAS:54135-51-2 MolWeight:495 RetIndex:2042
CompName:N-(Trifluoracetyl)-O,O',O''-tris(trimethylsilyl)epinephrine $$ N-(Trifluoracetyl)-O,O',O''-tris(trimethylsilyl)epinephrine $$ N-(2-(3,4-Bis[(trimetl

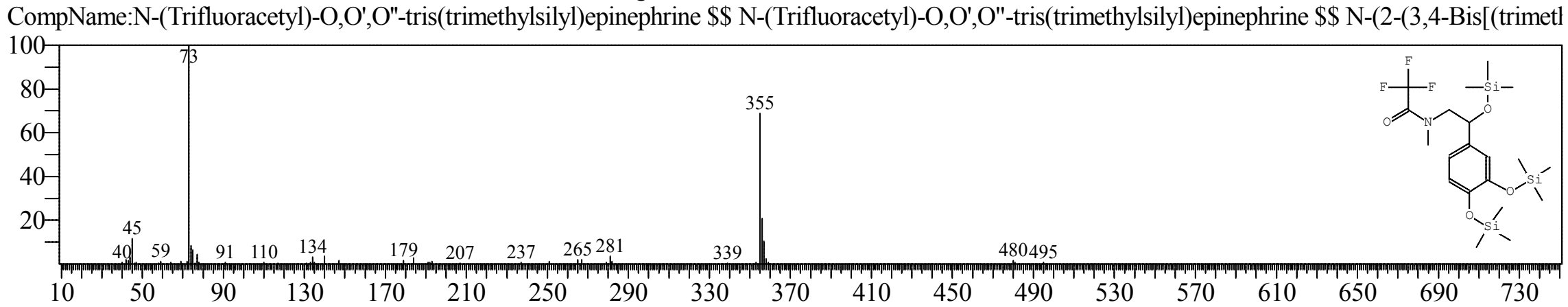

Hit#:17 Entry:306567 Library:WILEY7.LIB
SI:49 Formula:C14 H42 O5 SI6 CAS:107-52-8 MolWeight:458 RetIndex:0
CompName:Hexasiloxane, tetradecamethyl- (CAS) Tetradecamethylhexasiloxane $$

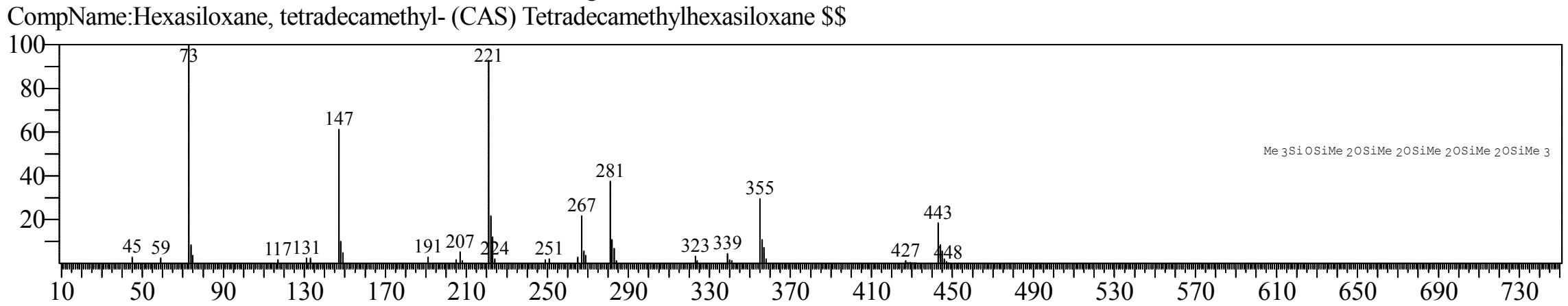

Hit#:18 Entry:161854 Library:NIST05.LIB
SI:48 Formula:C16H48O10Si9 CAS:145344-72-5 MolWeight:652 RetIndex:1716
CompName:2-(2',4',4',6',6',8',8'-Heptamethyltetrasiloxan-2'-yloxy)-2,4,4,6,6,8,8,10,10-nonamethylcyclopentasiloxane $$ 2-[(2,4,4,6,6,8,8-Heptamethyl-1,3,5

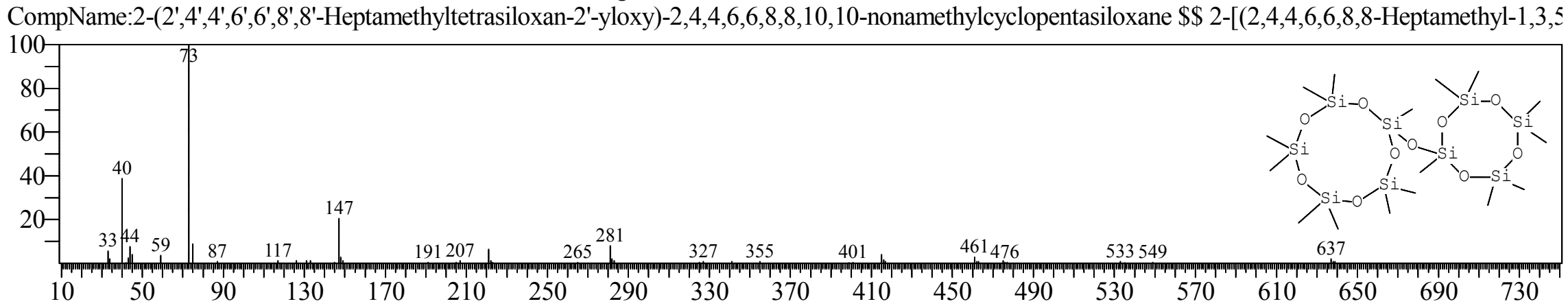

Hit#:19 Entry:152159 Library:NIST05.LIB
SI:48 Formula:C13H40O5Si6 CAS:38147-00-1 MolWeight:444 RetIndex:1297
CompName:1,1,1,5,7,7,7-Heptamethyl-3,3-bis(trimethylsiloxy)tetrasiloxane $$ 1,3,3,3-Tetramethyldisiloxanyl tris(trimethylsilyl) orthosilicate # $$

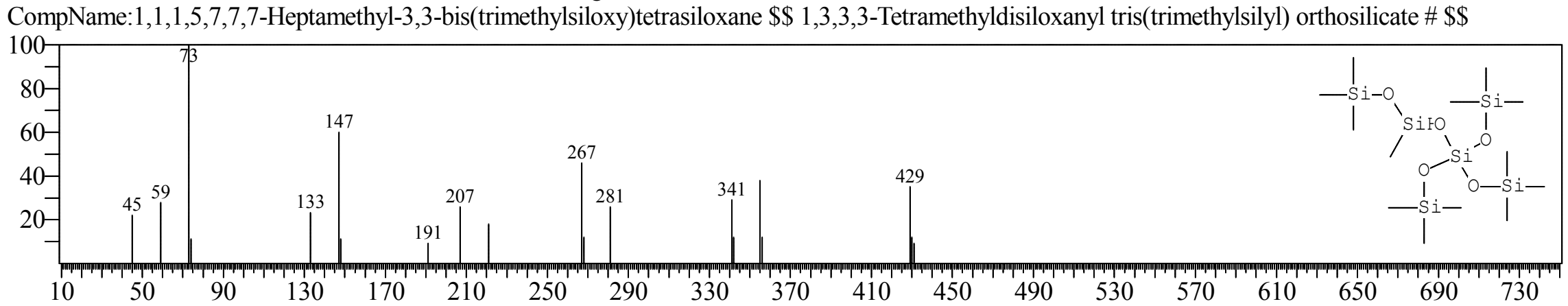

Hit#:20 Entry:27366 Library:NIST05s.LIB
SI:48 Formula:C16H48O6Si7 CAS:541-01-5 MolWeight:532 RetIndex:1437
CompName:Heptasiloxane, hexadecamethyl- $$ Hexadecamethylheptasiloxane $$ 1,1,1,3,3,5,5,7,7,9,9,11,11,13,13,13-Hexadecamethylheptasiloxane # $$

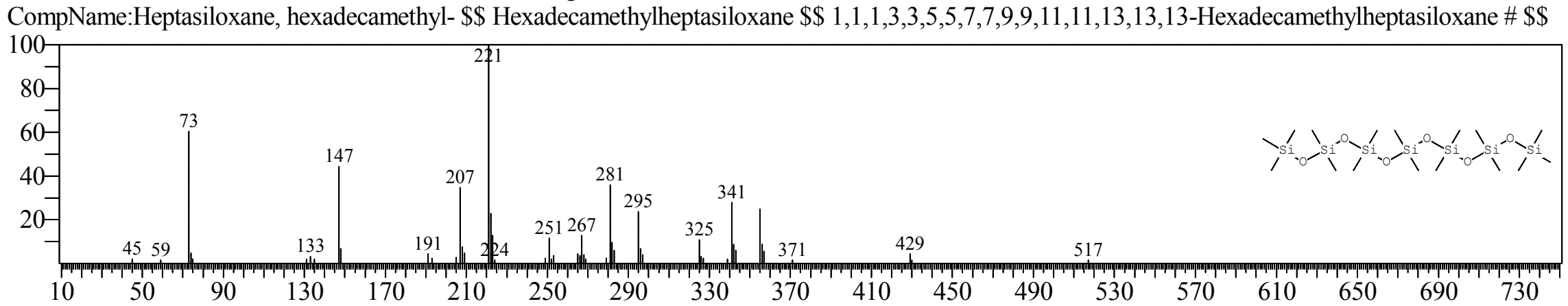

<< Target >>
Line#:28   R.Time:22.375(Scan#:2086) MassPeaks:73
RawMode:Averaged 22.367-22.383(2085-2087) BasePeak:149.10(10897)
BG Mode:Calc. from Peak Group 1 - Event 1 Scan

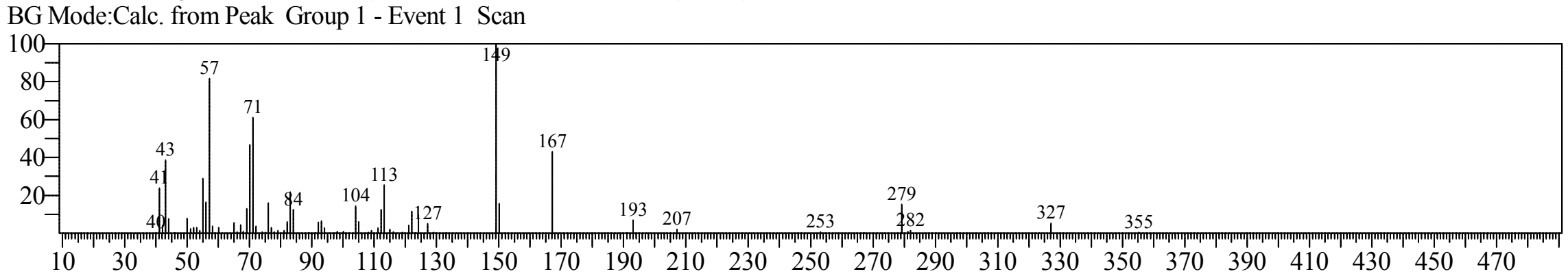

Hit#:1 Entry:26379 Library:NIST05s.LIB
SI:85 Formula:C24H38O4 CAS:117-84-0 MolWeight:390 RetIndex:2832
CompName:Di-n-octyl phthalate $$ 1,2-Benzenedicarboxylic acid, dioctyl ester $$ Phthalic acid, dioctyl ester $$ n-Octyl phthalate $$ Dinopol NOP $$ Dioc

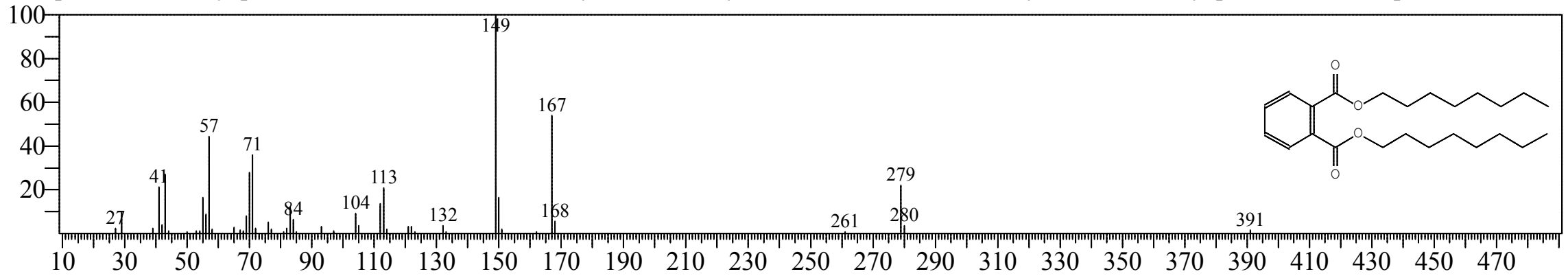

Hit#:2 Entry:279558 Library:WILEY7.LIB
SI:83 Formula:C24 H38 O4 CAS:117-81-7 MolWeight:390 RetIndex:0
CompName:1,2-Benzenedicarboxylic acid, bis(2-ethylhexyl) ester (CAS) Bis(2-ethylhexyl) phthalate $$ DOP $$ DEHP $$ DOF $$ DNOP $$ Octoil $$ Fle:

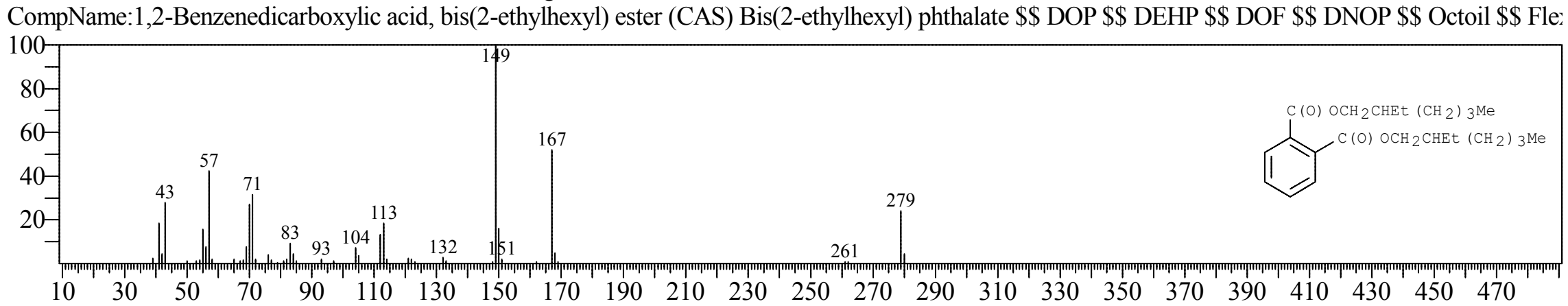

Hit#:3 Entry:279573 Library:WILEY7.LIB
SI:83 Formula:C24 H38 O4 CAS:27554-26-3 MolWeight:390 RetIndex:0
CompName:1,2-Benzenedicarboxylic acid, diisooctyl ester (CAS) Isooctyl phthalate $$ Hexaplas M/O $$ Diisooctyl phthalate $$ Di-isooctyl phthalate $$ IS

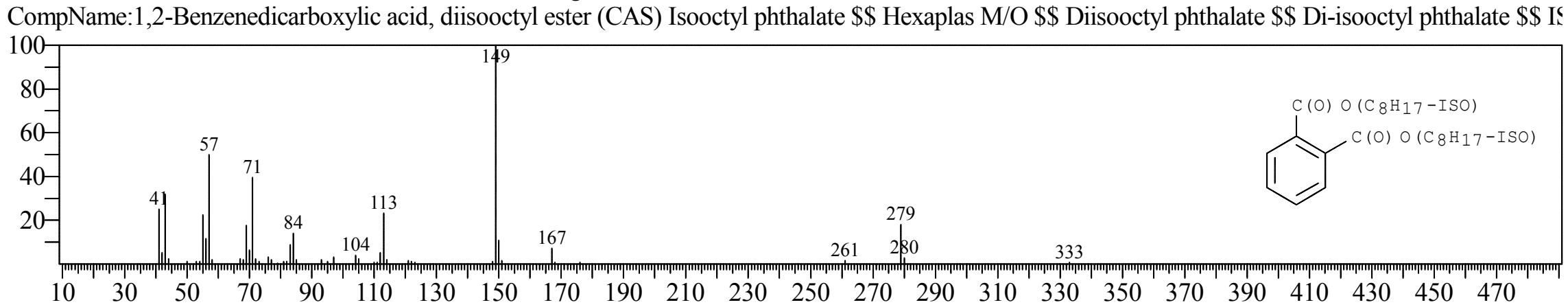

Hit#:4 Entry:29517 Library:NIST11s.lib
SI:83 Formula:C24H38O4 CAS:131-20-4 MolWeight:390 RetIndex:2704
CompName:Diisooctyl phthalate $$ Bis(6-methylheptyl) phthalate $$ 1,2-Benzenedicarboxylic acid, bis(6-methylheptyl) ester $$ Phthalic acid, bis(6-methyl

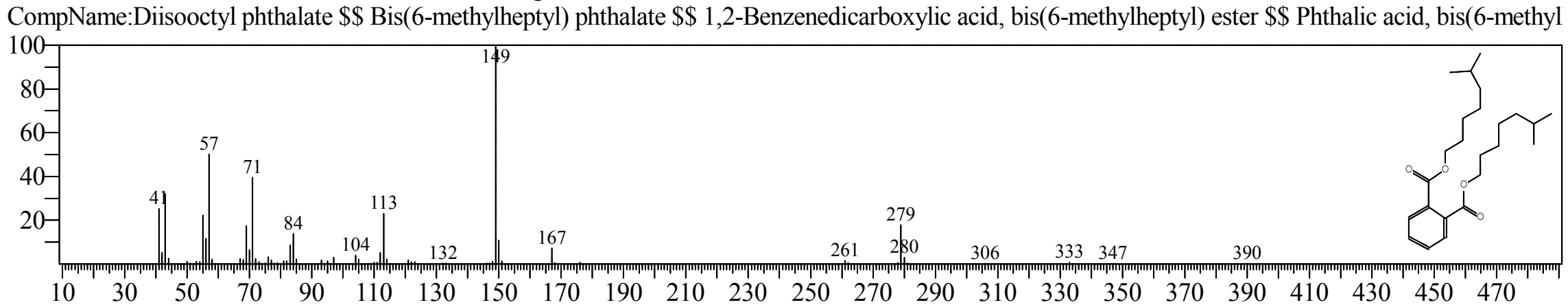

Hit#:5 Entry:293133 Library:WILEY7.LIB
SI:80 Formula:C26 H42 O4 CAS:84-76-4 MolWeight:418 RetIndex:0
CompName:1,2-Benzenedicarboxylic acid, dinonyl ester (CAS) Unimoll DN $$ PHTHALIC ACID BIS-(3,5,5-TRIMETHYL-HEXYL)-ESTER $$ Bisoflex

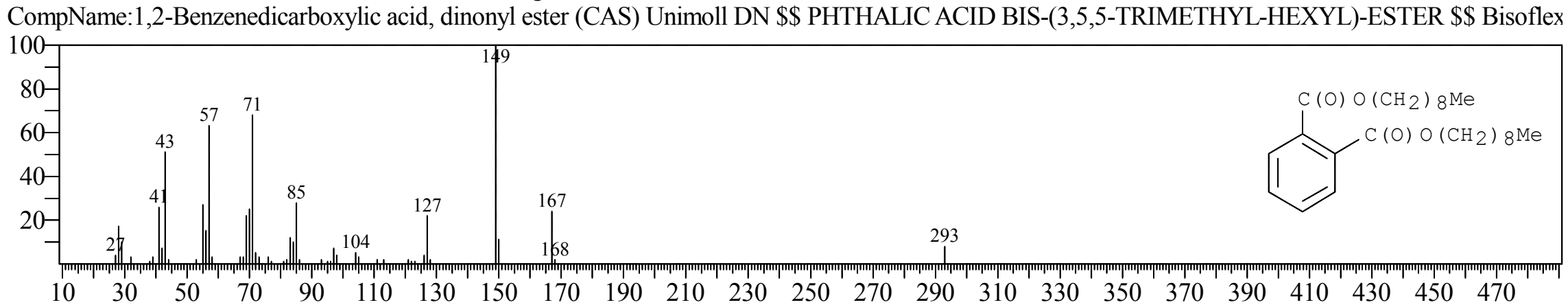

<< Target >>
Line#:28 R.Time:22.375(Scan#:2086) MassPeaks:73
RawMode:Averaged 22.367-22.383(2085-2087) BasePeak:149.10(10897)
BG Mode:Calc. from Peak Group 1 - Event 1 Scan

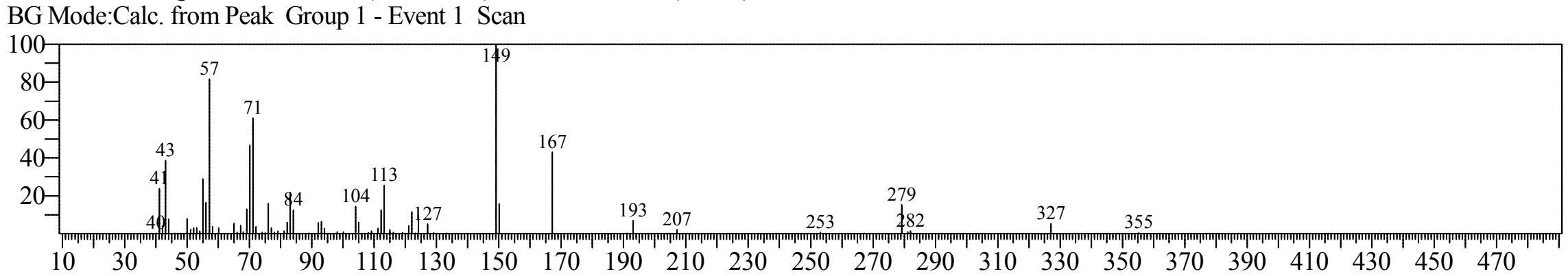

Hit#:6 Entry:106895 Library:NIST05.LIB
SI:80 Formula:C20H26N2O CAS:2656-44-2 MolWeight:310 RetIndex:2510
CompName:Aspidofractinine-3-methanol, (2.alpha.,3.beta.,5.alpha.)- $$ Kopsinyl alcohol $$ Aspidofractinin-3-ylmethanol # $$

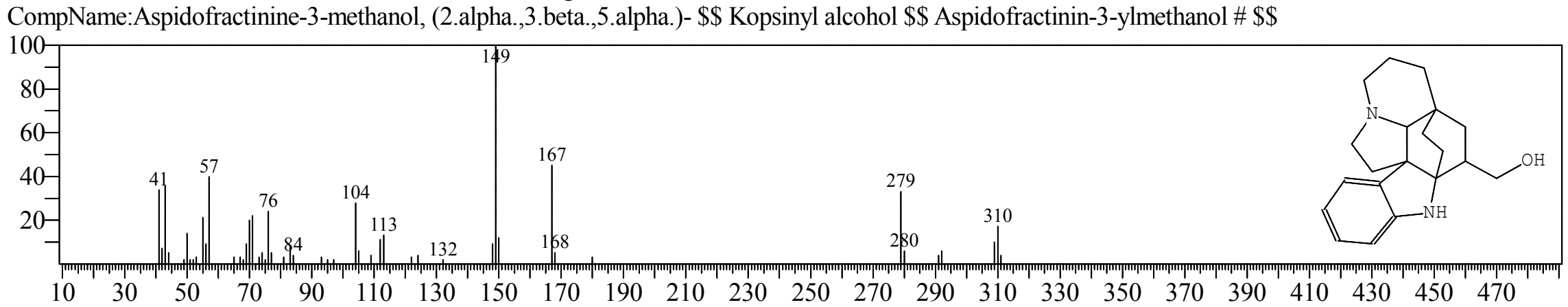

Hit#:7 Entry:148641 Library:WILEY7.LIB
SI:80 Formula:C12 H10 F N5 CAS:74421-46-8 MolWeight:243 RetIndex:0
CompName:1H-Purin-6-amine, [(4-fluorophenyl)methyl]- (CAS)

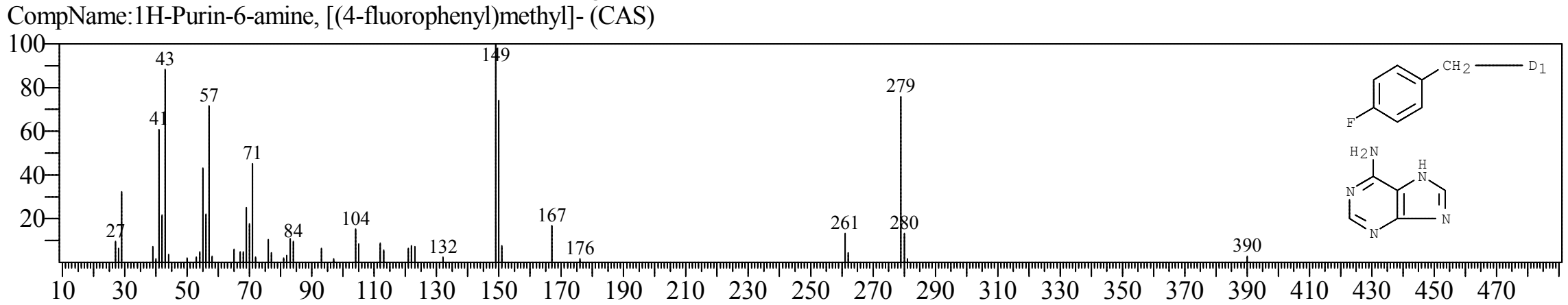

Hit#:8 Entry:158768 Library:NIST05.LIB
SI:78 Formula:C34H58O4 CAS:119-06-2 MolWeight:530 RetIndex:3826
CompName:1,2-Benzenedicarboxylic acid, ditridecyl ester $$ Phthalic acid, ditridecyl ester $$ Bis(tridecyl) phthalate $$ Ditridecyl phthalate $$ Polycizer 9(

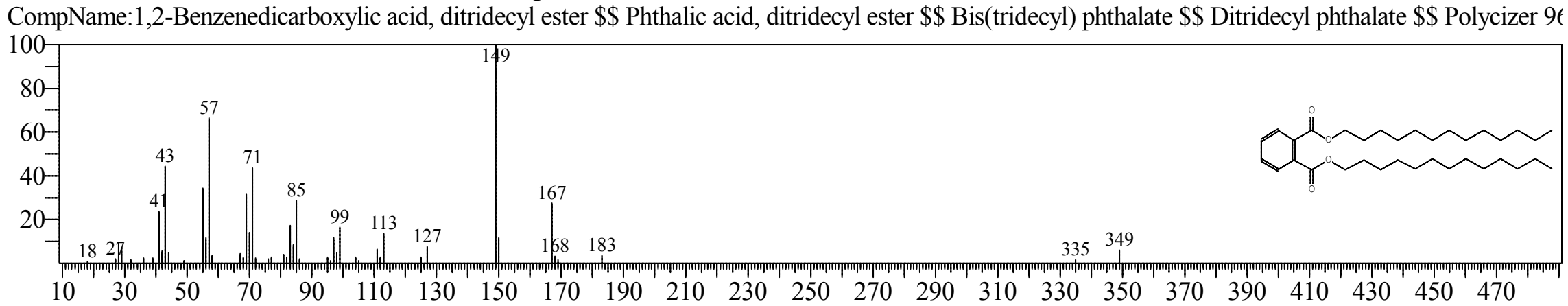

Hit#:9 Entry:29514 Library:NIST11s.lib
SI:78 Formula:C24H38O4 CAS:137-89-3 MolWeight:390 RetIndex:2704
CompName:1,3-Benzenedicarboxylic acid, bis(2-ethylhexyl) ester $$ Bis(2-ethylhexyl) isophthalate $$ Di-2-ethylhexyl isophthalate $$ Dioctyl isophthalate :

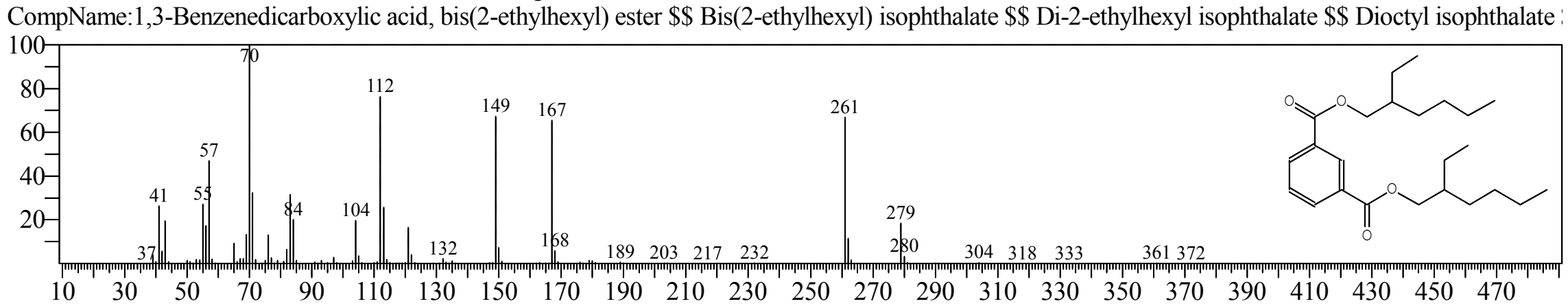

Hit#:10 Entry:88004 Library:NIST05.LIB
SI:78 Formula:C16H22O4 CAS:4376-20-9 MolWeight:278 RetIndex:2162
CompName:1,2-Benzenedicarboxylic acid, mono(2-ethylhexyl) ester $$ Mono(2-ethylhexyl) phthalate $$ Phthalic acid, mono-(2-ethylhexyl) ester $$ Mehp

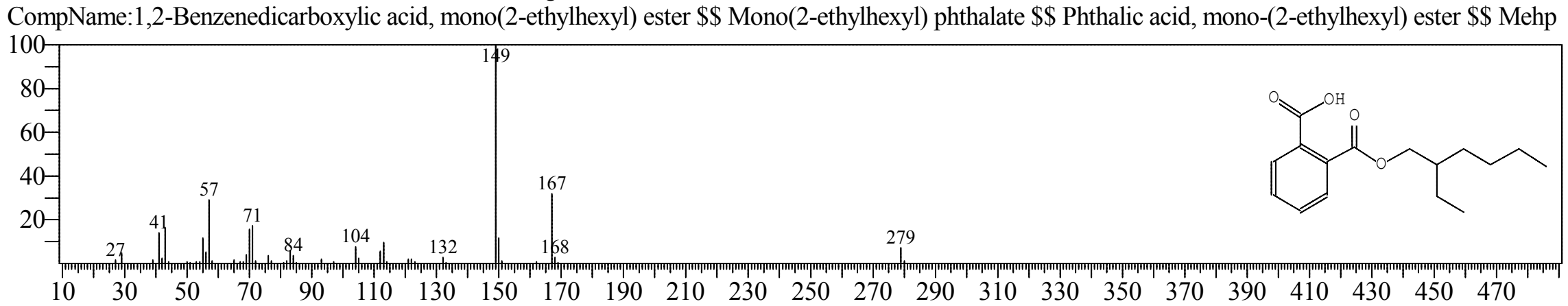

<< Target >>
Line#:28 R.Time:22.375(Scan#:2086) MassPeaks:73
RawMode:Averaged 22.367-22.383(2085-2087) BasePeak:149.10(10897)
BG Mode:Calc. from Peak Group 1 - Event 1 Scan

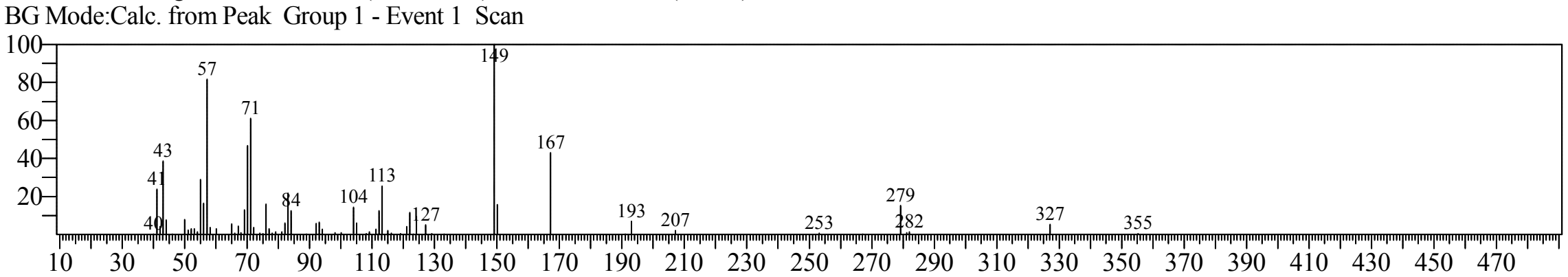

Hit#:11 Entry:178177 Library:NIST11.lib
SI:76 Formula:C24H38O4 CAS:0-00-0 MolWeight:390 RetIndex:2575
CompName:Phthalic acid, di(4-methylhept-3-yl) ester

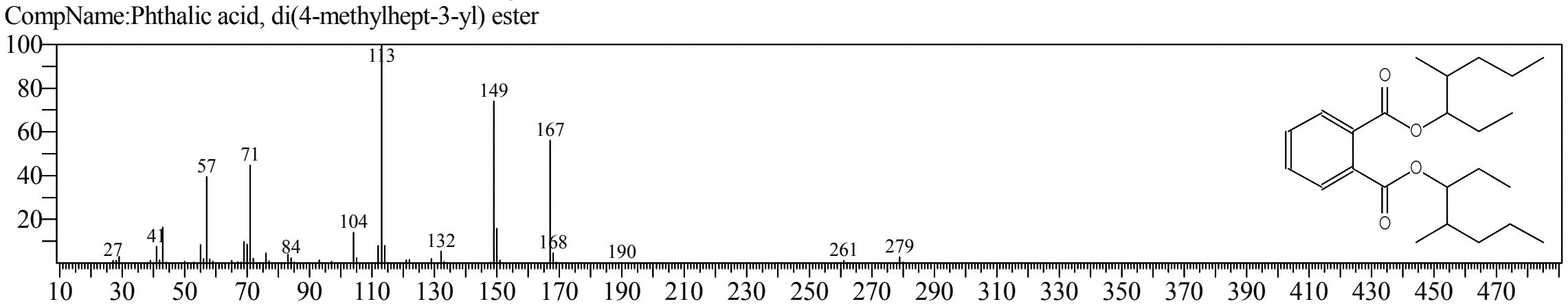

Hit#:12 Entry:26786 Library:NIST05s.LIB
SI:75 Formula:C26H42O4 CAS:20548-62-3 MolWeight:418 RetIndex:2902
CompName:Phthalic acid, bis(7-methyloctyl) ester $$ 1,2-Benzenedicarboxylic acid, bis(7-methyloctyl) ester $$ Bis(7-methyloctyl) phthalate # $$

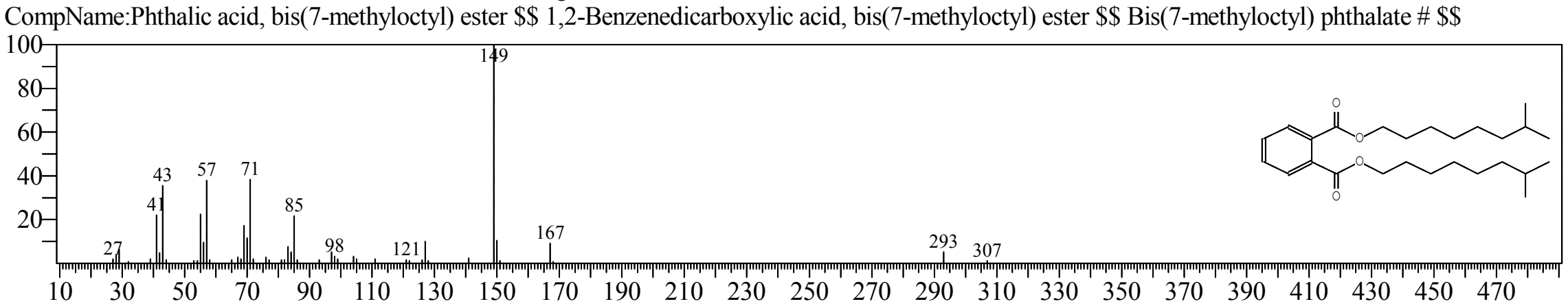

Hit#:13 Entry:292961 Library:WILEY7.LIB
SI:75 Formula:C26 H42 O4 CAS:28553-12-0 MolWeight:418 RetIndex:0
CompName:1,2-Benzenedicarboxylic acid, diisononyl ester $$ Phthalic acid, diisononyl ester $$ Diisononyl phthalate $$ ENJ 2065 $$ Palatinol DN $$ Vest

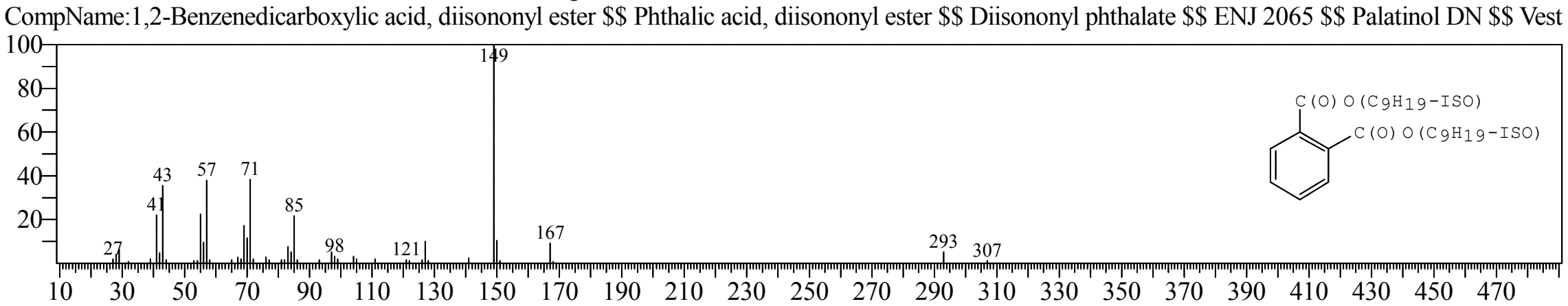

Hit#:14 Entry:109005 Library:WILEY7.LIB
SI:75 Formula:C8 H5 N O6 CAS:603-11-2 MolWeight:211 RetIndex:0
CompName:1,2-Benzenedicarboxylic acid, 3-nitro- (CAS) 3-Nitrophthalic acid $$ Phthalic acid, 3-nitro- $$ m-Nitrophthalic acid $$

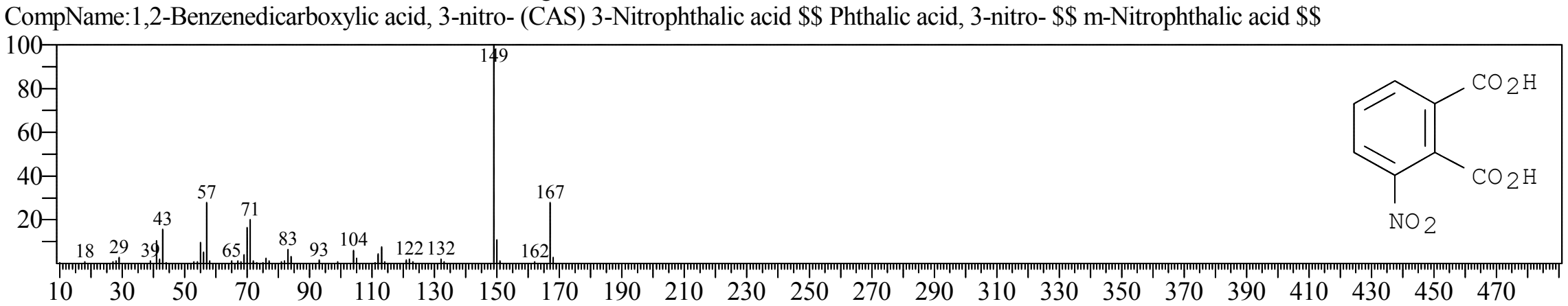

Hit#:15 Entry:148408 Library:NIST05.LIB
SI:75 Formula:C26H42O4 CAS:14103-61-8 MolWeight:418 RetIndex:2733
CompName:Bis-(3,5,5-trimethylhexyl) phthalate $$ 1,2-Benzenedicarboxylic acid, bis(3,5,5-trimethylhexyl) ester $$

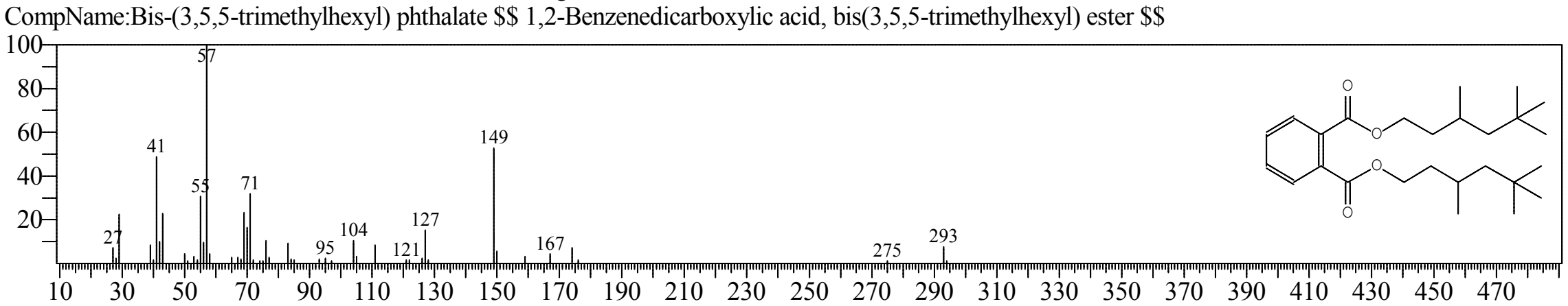

<< Target >>
Line#:28 R.Time:22.375(Scan#:2086) MassPeaks:73
RawMode:Averaged 22.367-22.383(2085-2087) BasePeak:149.10(10897)
BG Mode:Calc. from Peak Group 1 - Event 1 Scan

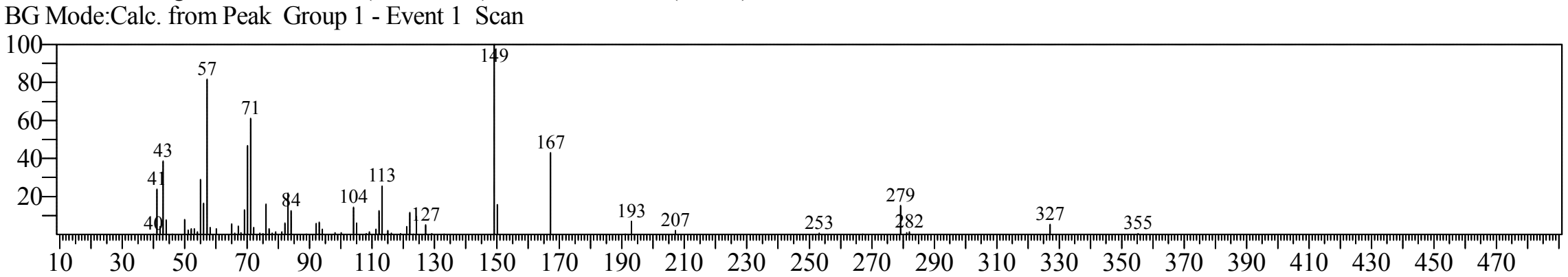

Hit#:16 Entry:303414 Library:WILEY7.LIB
SI:74 Formula:C28 H46 O4 CAS:26761-40-0 MolWeight:446 RetIndex:0
CompName:1,2-Benzenedicarboxylic acid, diisodecyl ester (CAS) PX 120 $$ Didp $$ Sicol 184 $$ Vestinol DZ $$ Palatinol Z $$ DIDP (plasticizer) $$ Dii

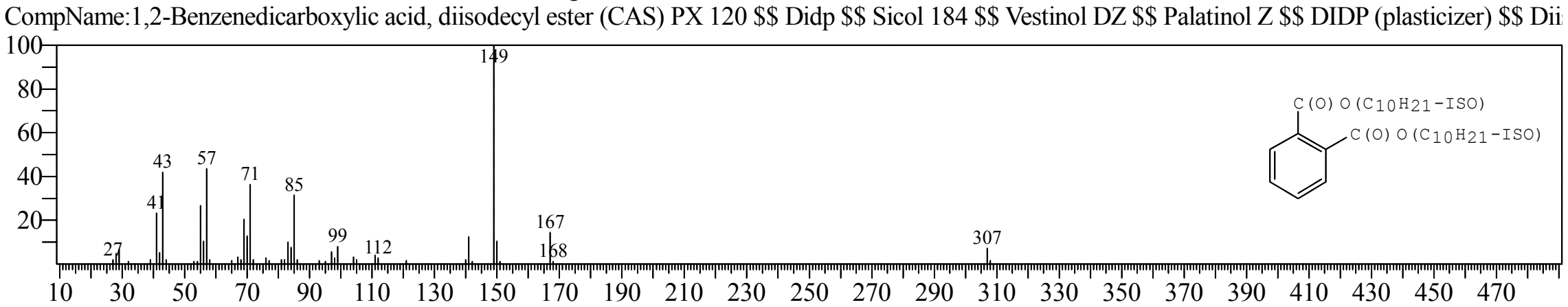

Hit#:17 Entry:156351 Library:NIST05.LIB
SI:74 Formula:C28H25NO7 CAS:127611-84-1 MolWeight:487 RetIndex:4187
CompName:3',8,8'-Trimethoxy-3-piperidyl-2,2'-binaphthalene-1,1',4,4'-tetrone $$ 3',8,8'-Trimethoxy-3-piperidin-1-yl-2,2'-binaphthyl-1,1',4,4'-tetrone $$

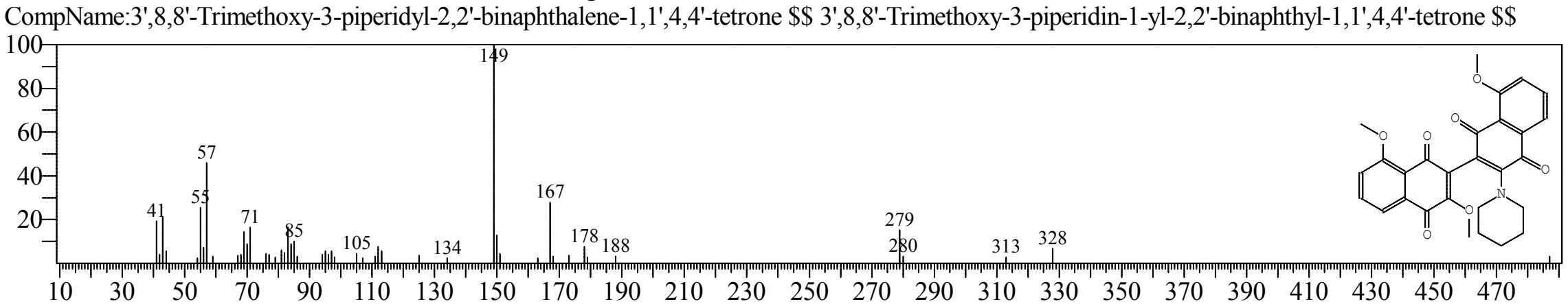

Hit#:18 Entry:27198 Library:NIST05s.LIB
SI:74 Formula:C30H50O4 CAS:3648-20-2 MolWeight:474 RetIndex:3428
CompName:1,2-Benzenedicarboxylic acid, diundecyl ester $$ Phthalic acid, diundecyl ester $$ Diundecyl phthalate $$ DUP $$ Santicizer 711 $$ Diundecyl

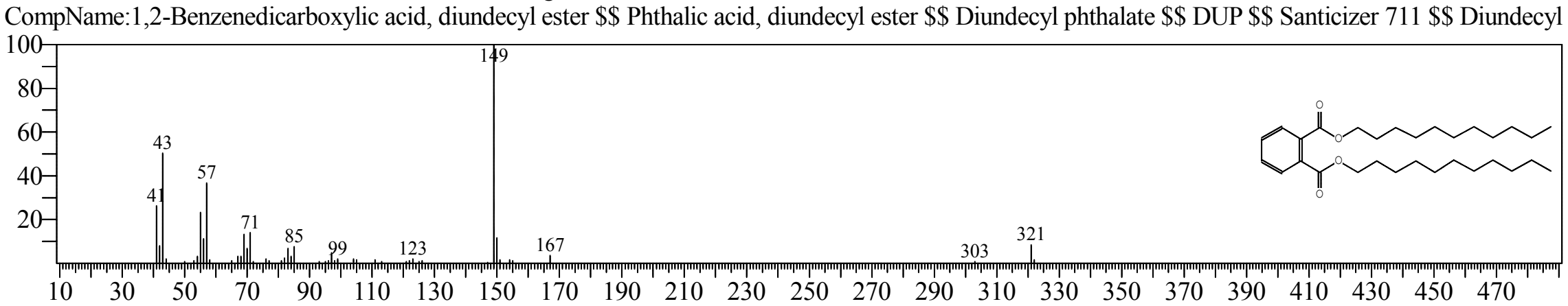

Hit#:19 Entry:303409 Library:WILEY7.LIB
SI:74 Formula:C28 H46 O4 CAS:84-77-5 MolWeight:446 RetIndex:0
CompName:Phthalic acid, didecyl ester $$ 1,2-Benzenedicarboxylic acid, didecyl ester (CAS) DIDECYL ESTER PHTHALIC ACID $$ Didecyl phthalate $

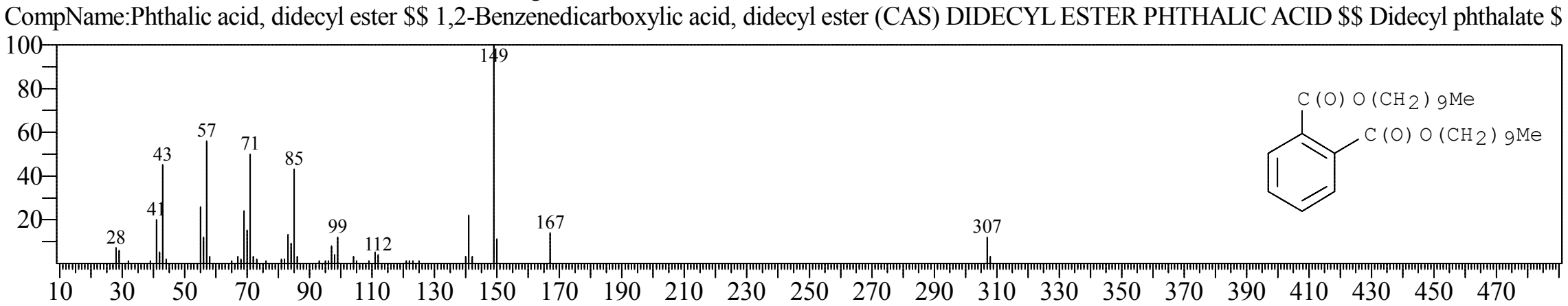

Hit#:20 Entry:141568 Library:NIST05.LIB
SI:74 Formula:C24H36O4 CAS:0-00-0 MolWeight:388 RetIndex:2831
CompName:Phthalic acid, 2-cyclohexylethyl 2-ethylhexyl ester

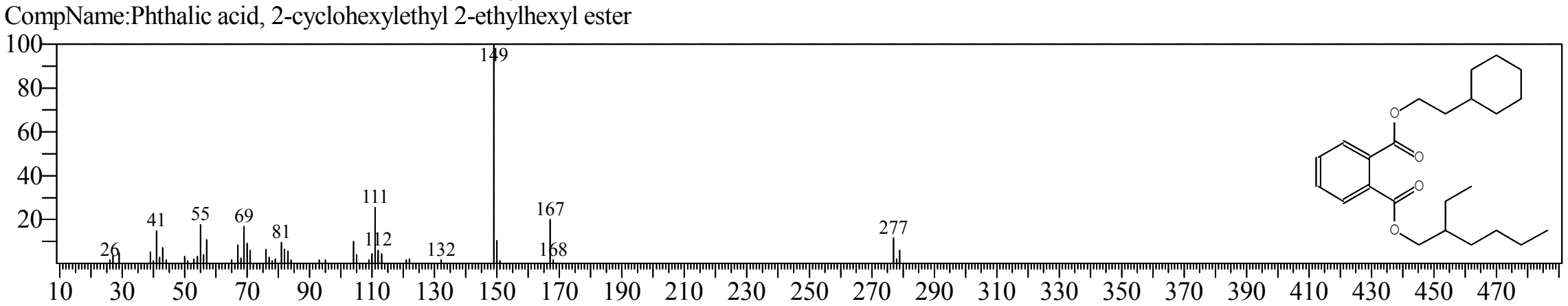

<< Target >>
Line#:29   R.Time:22.592(Scan#:2112) MassPeaks:49
RawMode:Averaged 22.583-22.600(2111-2113) BasePeak:39.95(1024)
BG Mode:Calc. from Peak Group 1 - Event 1 Scan

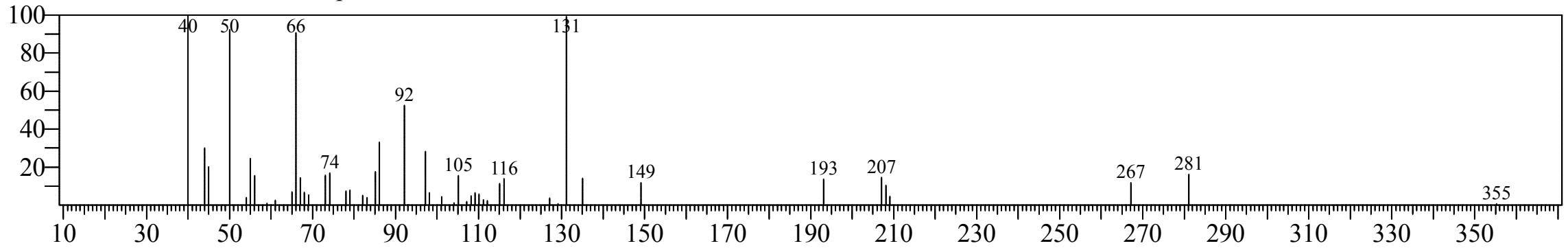

Hit#:1 Entry:13175 Library:NIST05.LIB
SI:52 Formula:C6H10S2 CAS:66612-70-2 MolWeight:146 RetIndex:1094
CompName:1,3-Butadiene, 1,1-bis(methylthio)- $$ 1,1-Bis(methylsulfanyl)-1,3-butadiene # $$

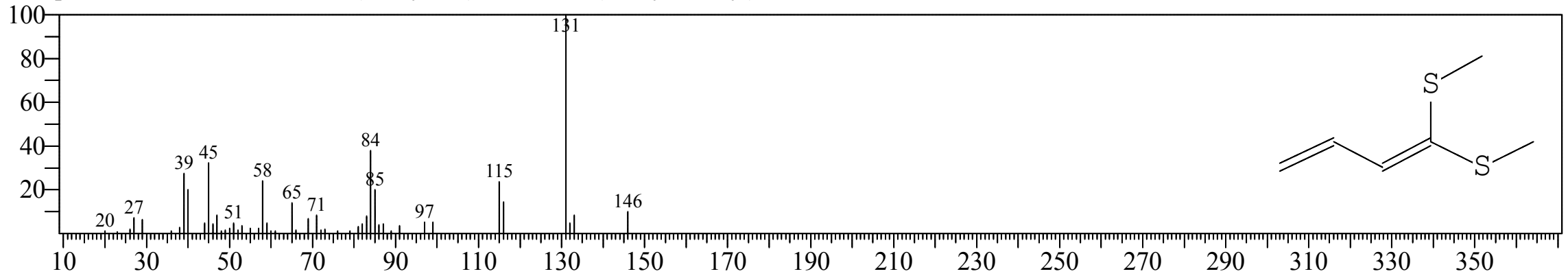

Hit#:2 Entry:80921 Library:WILEY7.LIB
SI:51 Formula:C13 H16 O CAS:0-00-0 MolWeight:188 RetIndex:0
CompName:Allyloxydicyclopentadiene $$

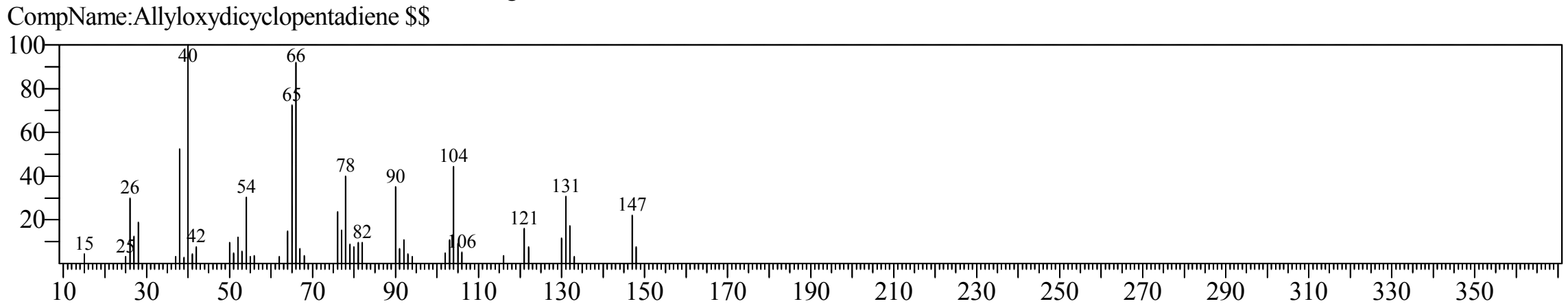

Hit#:3 Entry:34166 Library:WILEY7.LIB
SI:51 Formula:C7 H11 CL O CAS:56816-12-7 MolWeight:146 RetIndex:0
CompName:Bicyclo[2.2.1]heptan-2-ol, 3-chloro- (CAS)

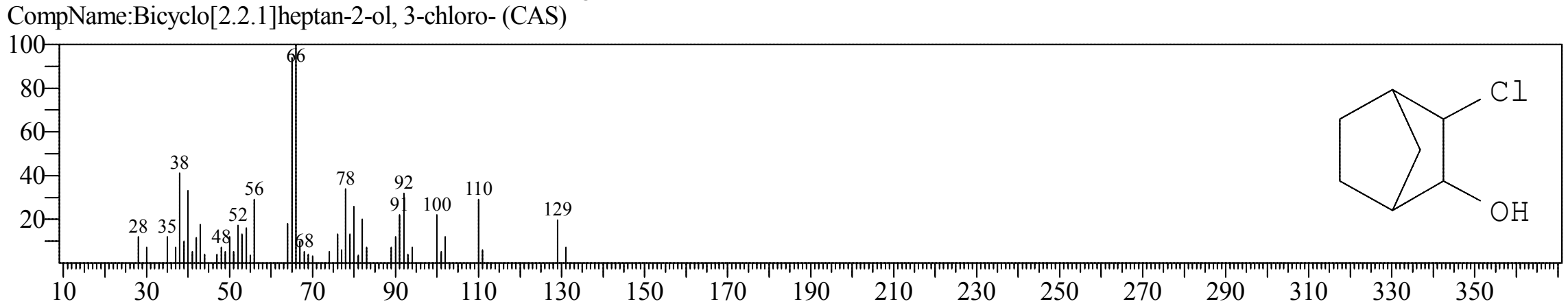

Hit#:4 Entry:264514 Library:WILEY7.LIB
SI:50 Formula:C23 H27 N O3 CAS:0-00-0 MolWeight:365 RetIndex:0
CompName:DIDECYL 1,4-DIHYDRO-2,6-DIMETHYL-3,5-PYRIDINEDICARBOXYLATE $$ 3,4,5,6,9,10-HEXAHYDRO-9-(4-HYDROXYPHENYL)

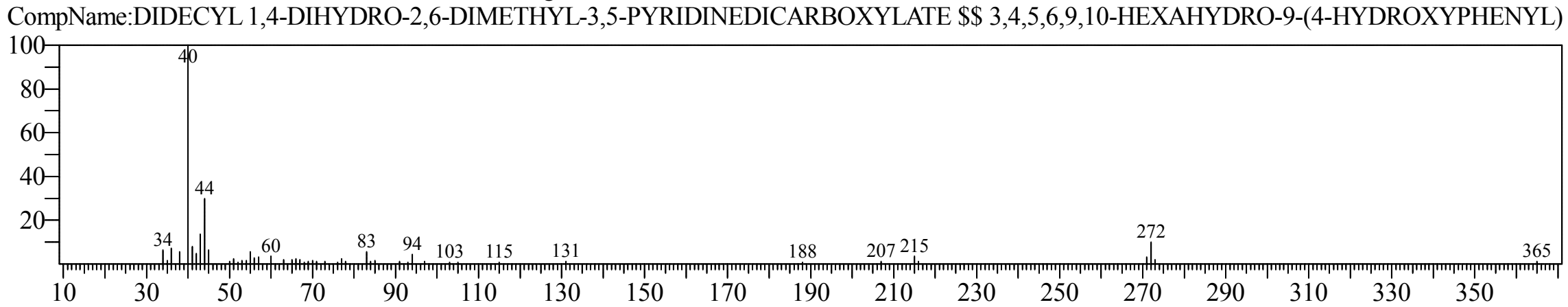

Hit#:5 Entry:46298 Library:NIST11.lib
SI:49 Formula:C11H12N2S CAS:0-00-0 MolWeight:204 RetIndex:1759
CompName:3-Pyridinemethanamine, N-(3-thienylmethyl)-

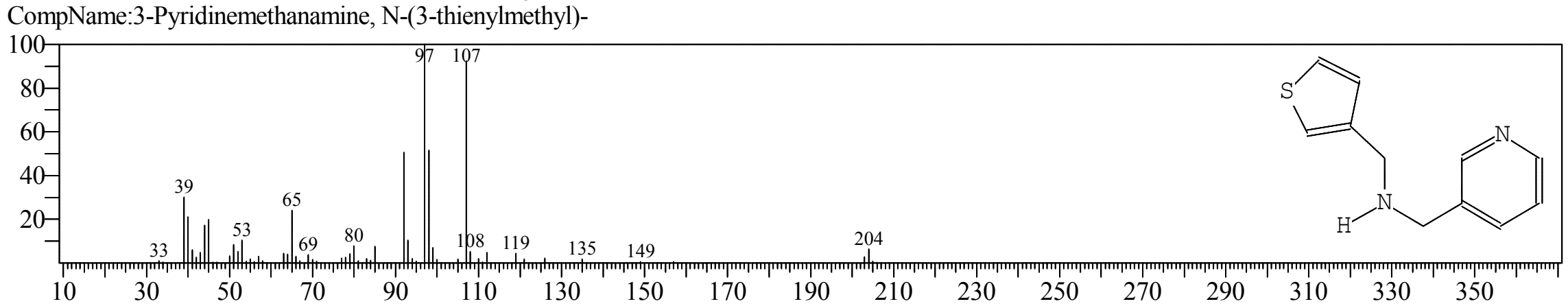

<< Target >>
Line#:29 R.Time:22.592(Scan#:2112) MassPeaks:49
RawMode:Averaged 22.583-22.600(2111-2113) BasePeak:39.95(1024)
BG Mode:Calc. from Peak Group 1 - Event 1 Scan

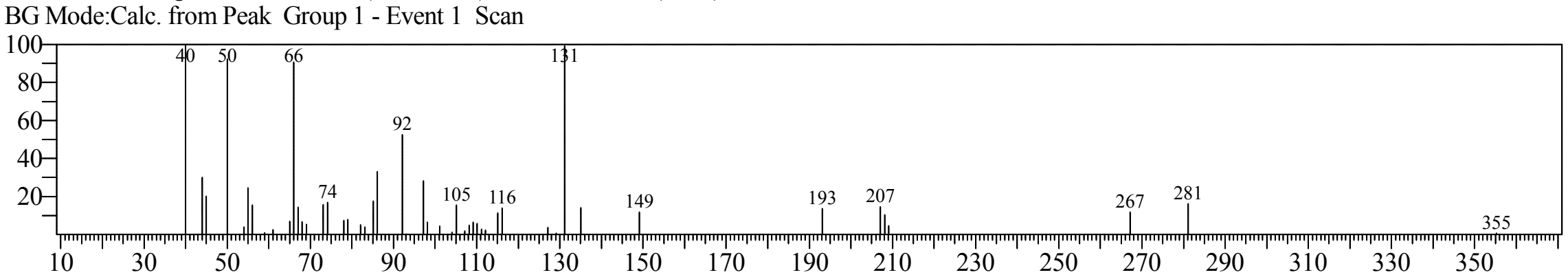

Hit#:6 Entry:286212 Library:WILEY7.LIB
SI:49 Formula:C22 H16 N2 O6 CAS:0-00-0 MolWeight:404 RetIndex:0
CompName:N,N'-ISOPHTHALOYLBIS(3-AMINOBENZOIC ACID) $$

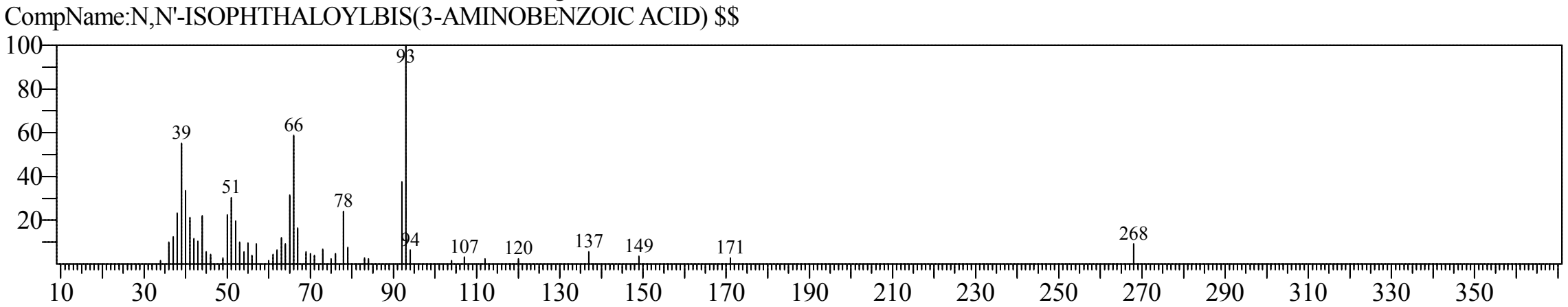

Hit#:7 Entry:45991 Library:NIST11.lib
SI:49 Formula:C9H8N4O2 CAS:0-00-0 MolWeight:204 RetIndex:0
CompName:Benzeneacetic acid, 4-(1H-1,2,3,4-tetrazol-1-yl)-

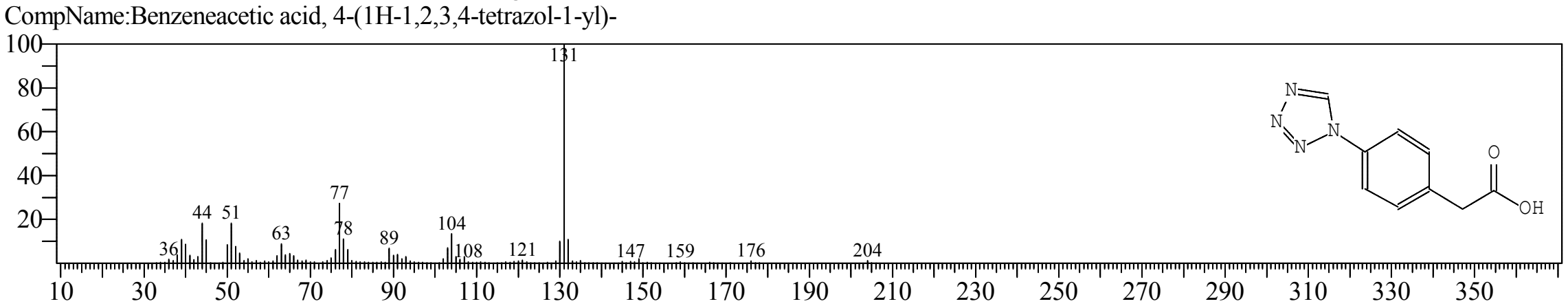

Hit#:8 Entry:145656 Library:NIST05.LIB
SI:49 Formula:C22H16N2O6 CAS:94413-83-9 MolWeight:404 RetIndex:3992
CompName:N,N'-Isophthaloylbis(3-aminobenzoic acid) $$ 3-((3-[(3-Carboxyanilino)carbonyl]benzoyl)amino)benzoic acid # $$

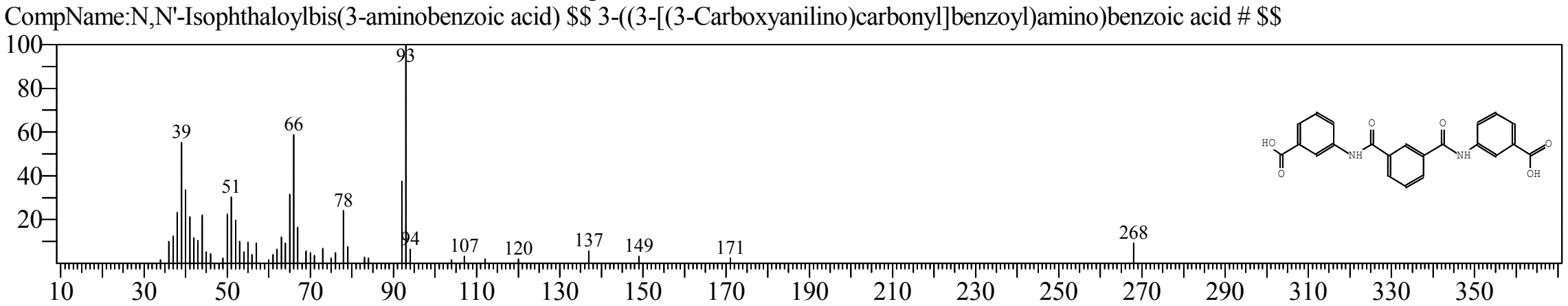

Hit#:9 Entry:52159 Library:NIST11.lib
SI:49 Formula:C12H24OSi CAS:0-00-0 MolWeight:212 RetIndex:1153
CompName:3-Trimethylsilyloxy-5-nonyne $$ [(1-Ethyl-3-heptynyl)oxy](trimethyl)silane # $$

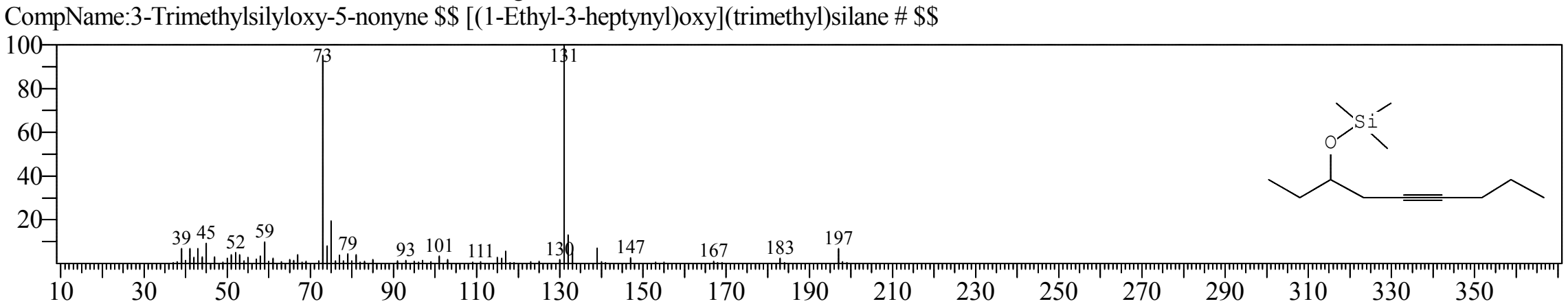

Hit#:10 Entry:151247 Library:WILEY7.LIB
SI:49 Formula:C4 H CL2 F5 O2 CAS:375-07-5 MolWeight:246 RetIndex:0
CompName:Butanoic acid, 3,4-dichloro-2,2,3,4,4-pentafluoro- (CAS) Butyric acid, 3,4-dichloro-2,2,3,4,4-pentafluoro- $$ 3,4-DICHLOROPENTAFLUOR(

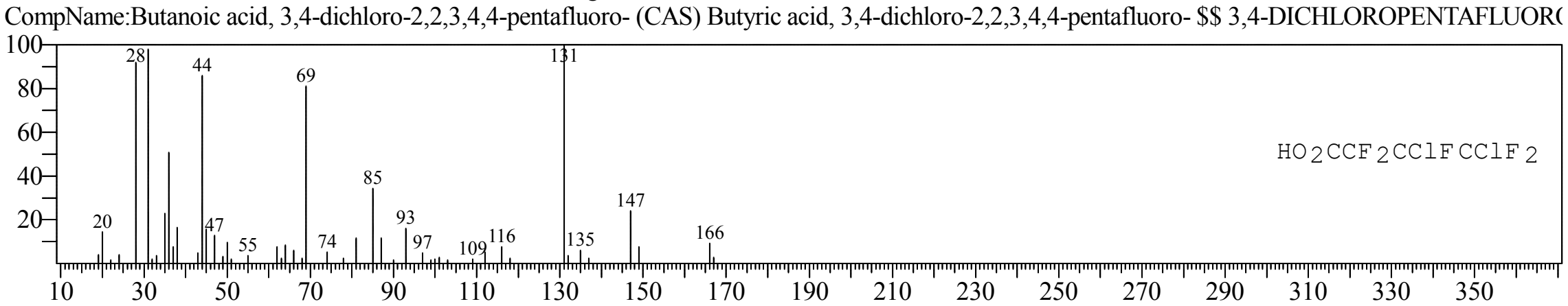

<< Target >>
Line#:29 R.Time:22.592(Scan#:2112) MassPeaks:49
RawMode:Averaged 22.583-22.600(2111-2113) BasePeak:39.95(1024)
BG Mode:Calc. from Peak Group 1 - Event 1 Scan

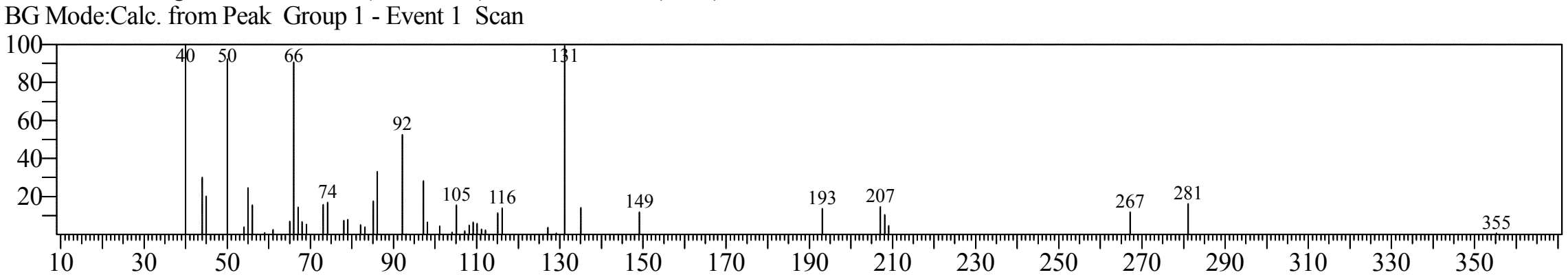

Hit#:11 Entry:48299 Library:NIST05.LIB
SI:49 Formula:C12H24OSi CAS:0-00-0 MolWeight:212 RetIndex:1153
CompName:3-Trimethylsilyloxy-5-nonyne

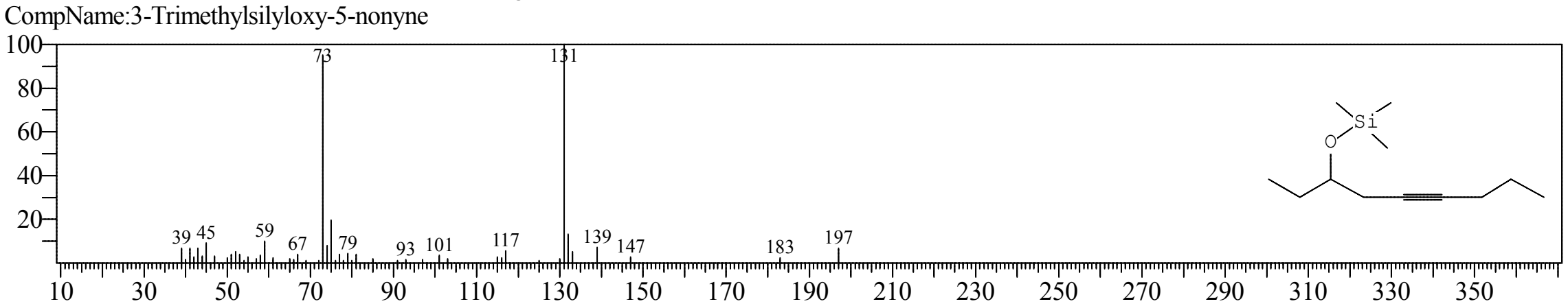

Hit#:12 Entry:36073 Library:WILEY7.LIB
SI:48 Formula:C10 H12 O CAS:0-00-0 MolWeight:148 RetIndex:0
CompName:BICYCLO [2.2.1] HEPT-2-ENE-5-YL,VINYL KETONE $$

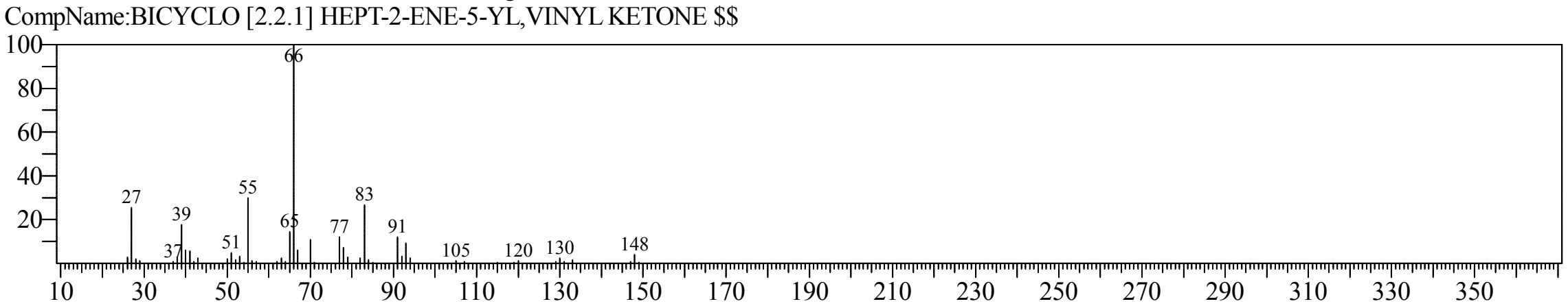

Hit#:13 Entry:2863 Library:WILEY7.LIB
SI:48 Formula:C5 H8 O CAS:5390-04-5 MolWeight:84 RetIndex:0
CompName:4-Pentyn-1-ol $$ 2-(.gamma.-hydroxy)propylacetylene $$ 1-Pentyn-5-ol $$ 1-Hydroxy-4-pentyne $$ 5-Hydroxy-1-pentyne $$

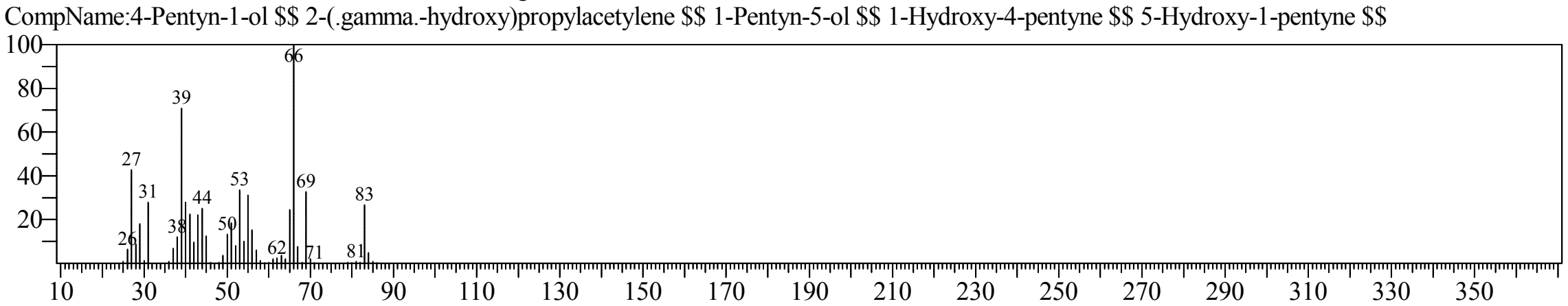

Hit#:14 Entry:8392 Library:NIST05.LIB
SI:48 Formula:C10H12 CAS:6707-86-4 MolWeight:132 RetIndex:685
CompName:1,2,4-Metheno-1H-cyclobuta[cd]pentalene, octahydro- $$ Pentacyclo[5.3.0.0(2,6).0(3,9).0(5,8)]decane $$ 1,3-Bishomocubane $$

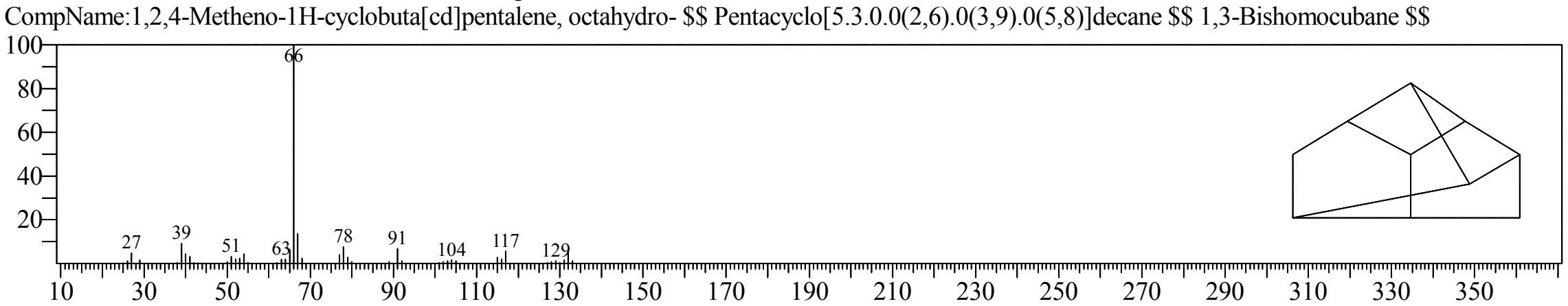

Hit#:15 Entry:5628 Library:NIST05s.LIB
SI:48 Formula:C10H12 CAS:77-73-6 MolWeight:132 RetIndex:845
CompName:4,7-Methano-1H-indene, 3a,4,7,7a-tetrahydro- $$ 4,7-Methanoindene, 3a,4,7,7a-tetrahydro- $$ Bicyclopentadiene $$ Bis[cyclopentadiene] $$ (

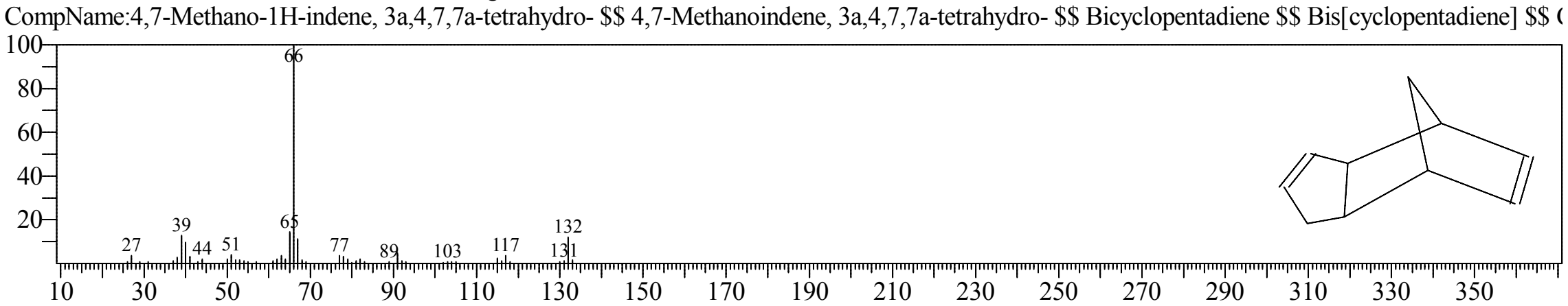

<< Target >>
Line#:29 R.Time:22.592(Scan#:2112) MassPeaks:49
RawMode:Averaged 22.583-22.600(2111-2113) BasePeak:39.95(1024)
BG Mode:Calc. from Peak Group 1 - Event 1 Scan

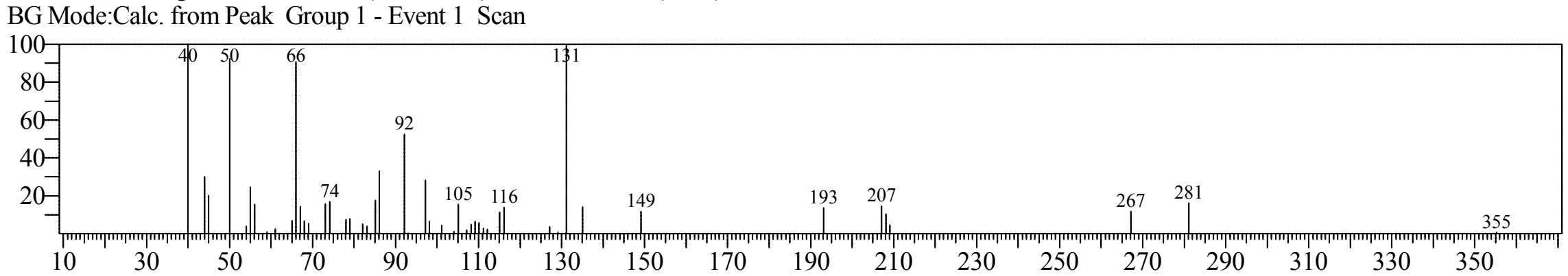

Hit#:16 Entry:27511 Library:NIST11.lib
SI:48 Formula:C11H11NO CAS:0-00-0 MolWeight:173 RetIndex:1340
CompName:4-Oxotricyclo[5.2.1.0(2,6)]dec-8-ene-3-carbonitrile

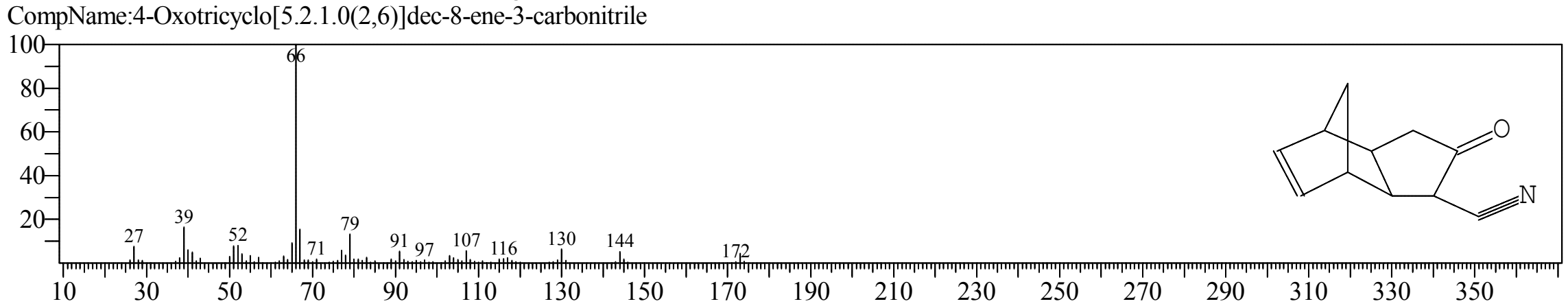

Hit#:17 Entry:26289 Library:NIST05.LIB
SI:48 Formula:C11H11NO CAS:0-00-0 MolWeight:173 RetIndex:1340
CompName:4-Oxotricyclo[5.2.1.0(2,6)]dec-8-ene-3-carbonitrile

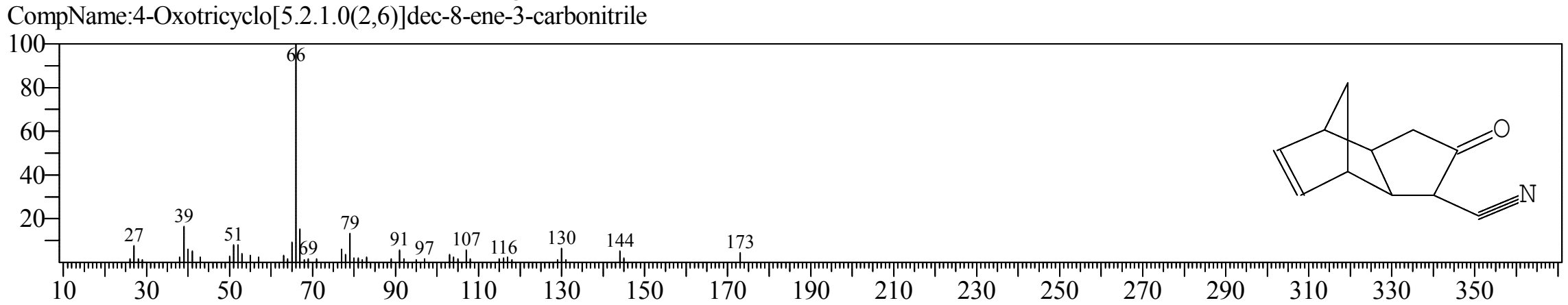

Hit#:18 Entry:63795 Library:WILEY7.LIB
SI:48 Formula:C11 H11 N O CAS:0-00-0 MolWeight:173 RetIndex:0
CompName:4-OXO-TRICYCLO[5.2.1.0 2,6]DEC-8-ENE-3-CARBONITRILE $$

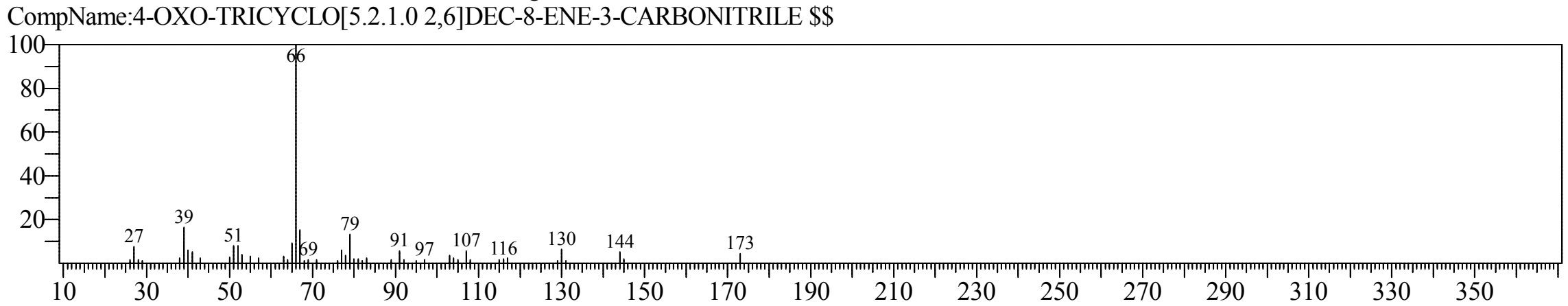

Hit#:19 Entry:82867 Library:WILEY7.LIB
SI:48 Formula:C13 H18 O CAS:55556-82-6 MolWeight:190 RetIndex:0
CompName:1,4-Ethano-5,8-methanonaphthalen-6-ol, 1,4,4a,5,6,7,8,8a-octahydro-, (1.alpha.,4.alpha.,4a.alpha.,5.beta.,6.beta.,8.beta.,8a.alpha.)- (CAS)

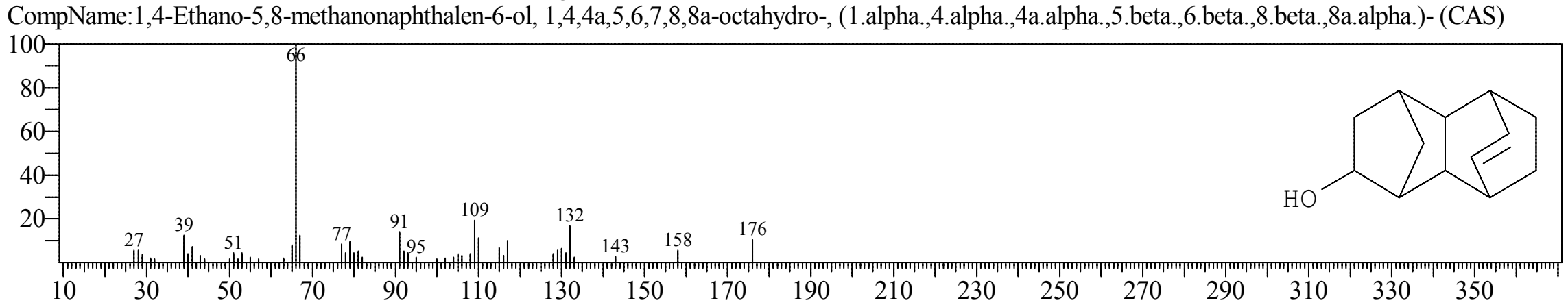

Hit#:20 Entry:66710 Library:WILEY7.LIB
SI:48 Formula:C12 H16 O CAS:33163-93-8 MolWeight:176 RetIndex:0
CompName:ENDO,EXO-1,2,3,4,4A,5,8,8A-OCTAHYDRO-1,4:5,8-DIMETHANONAPHTALENE ANTI-2-OL $$ 1,4:5,8-Dimethanonaphthalen-2-ol, 1,2,

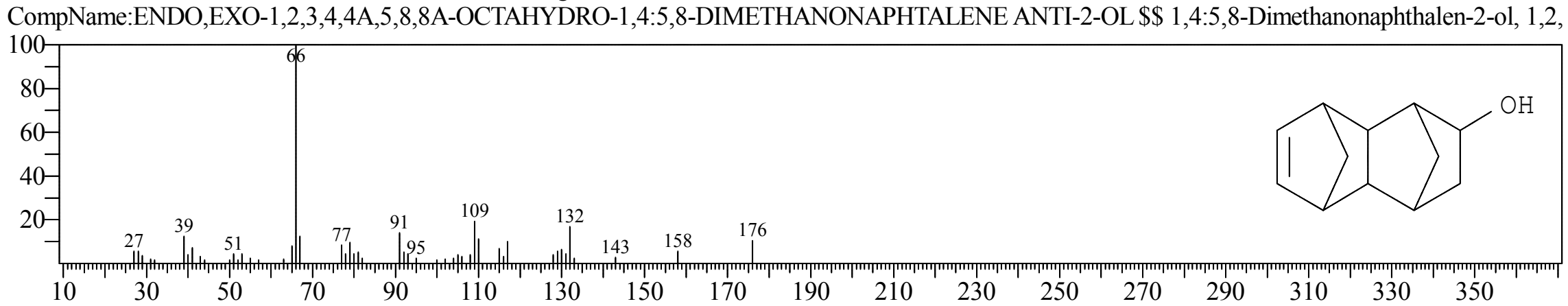

<< Target >>
Line#:30 R.Time:22.700(Scan#:2125) MassPeaks:49
RawMode:Averaged 22.692-22.708(2124-2126) BasePeak:40.00(3789)
BG Mode:Calc. from Peak Group 1 - Event 1 Scan

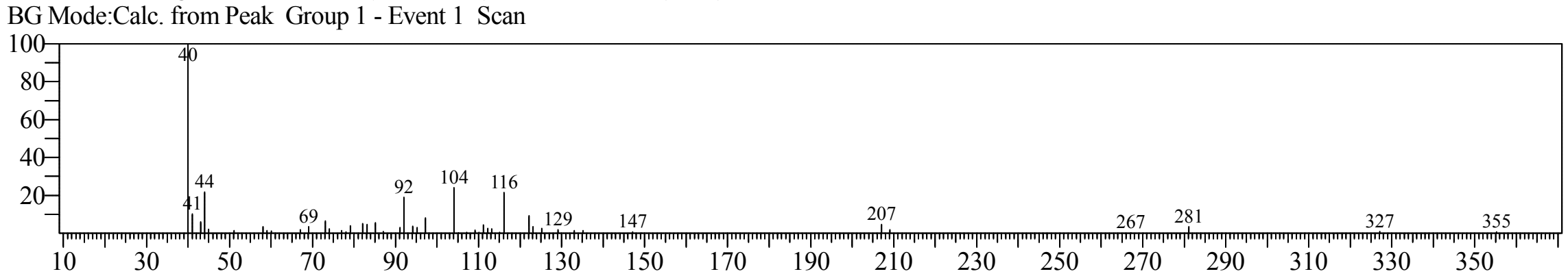

Hit#:1 Entry:257755 Library:WILEY7.LIB
SI:64 Formula:C22 H13 N O4 CAS:0-00-0 MolWeight:355 RetIndex:0
CompName:ETHYL 1-HEXYL-4-HYDROXY-2(1H)-OXO-3-QUINOLINECARBOXYLATE $$ 4-HYDROXY-3-(2-OXO-2H-1-OXA-3-PHENANTHRY

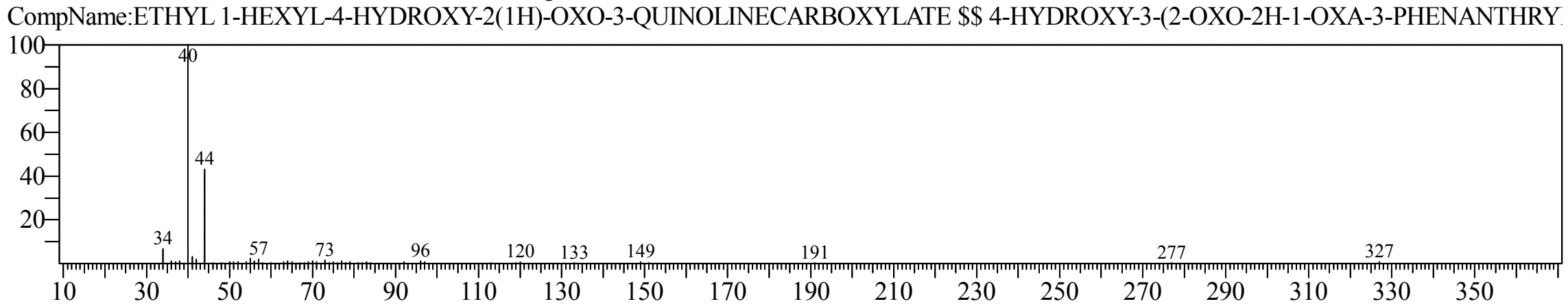

Hit#:2 Entry:264514 Library:WILEY7.LIB
SI:62 Formula:C23 H27 N O3 CAS:0-00-0 MolWeight:365 RetIndex:0
CompName:DIDECYL 1,4-DIHYDRO-2,6-DIMETHYL-3,5-PYRIDINEDICARBOXYLATE $$ 3,4,5,6,9,10-HEXAHYDRO-9-(4-HYDROXYPHENYL)

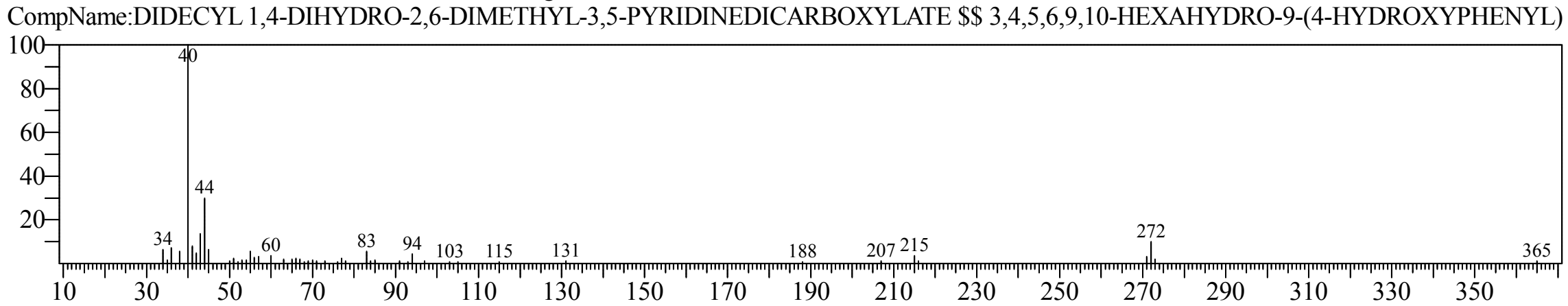

Hit#:3 Entry:250539 Library:WILEY7.LIB
SI:61 Formula:C15 H12 BR N3 O2 CAS:0-00-0 MolWeight:345 RetIndex:0
CompName:N-(P-ANISIDINOMETHYL)-4-METHYLPHTHALIMIDE $$ 4-BROMO-N-[(6-METHYL-2-PYRIDYL)AMINOMETHYL]PHTHALIMIDE

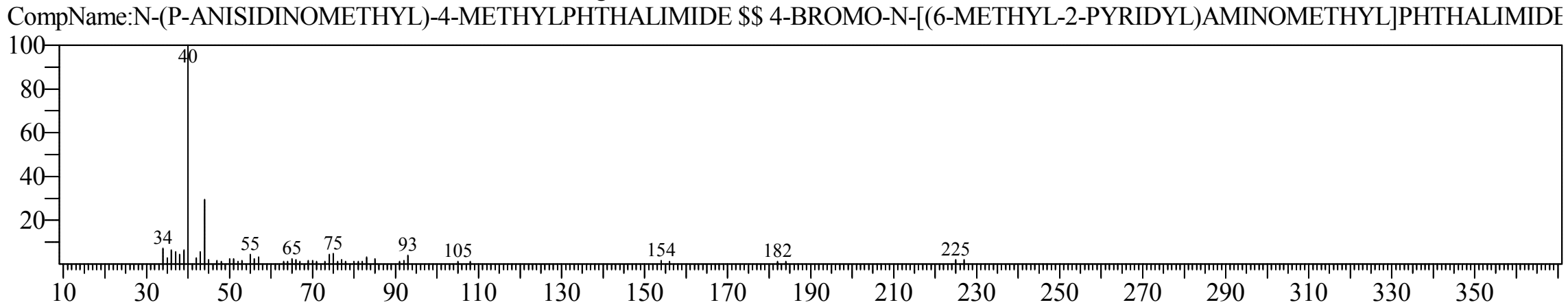

Hit#:4 Entry:212684 Library:WILEY7.LIB
SI:60 Formula:C12 H12 F6 O2 CAS:0-00-0 MolWeight:302 RetIndex:0
CompName:8,9,9,10,10,11-HEXAFLUORO-4,4-DIMETHYL-3,5-DIOXATETRACYCLO[5.4.1.0(2, $$

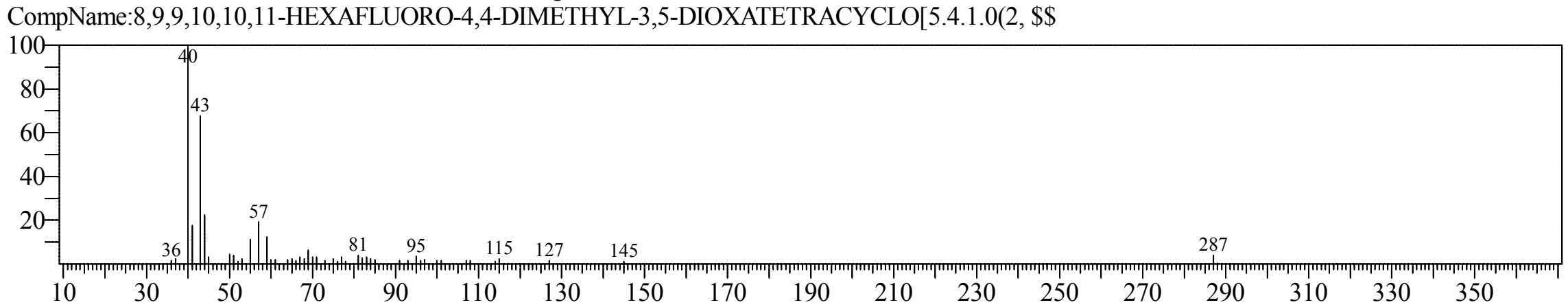

Hit#:5 Entry:98196 Library:WILEY7.LIB
SI:60 Formula:C14 H18 O CAS:33046-68-3 MolWeight:202 RetIndex:0
CompName:3-Octen-2-one, 8-phenyl- (CAS)

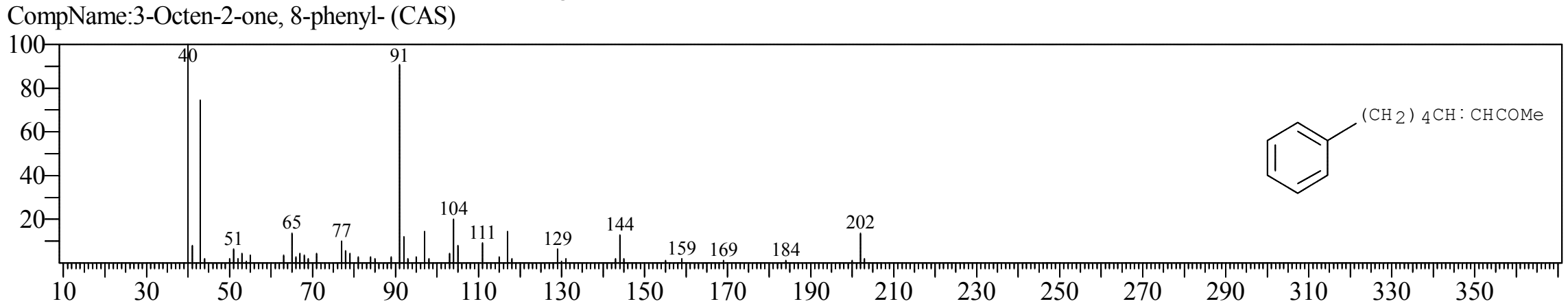

<< Target >>
Line#:30 R.Time:22.700(Scan#:2125) MassPeaks:49
RawMode:Averaged 22.692-22.708(2124-2126) BasePeak:40.00(3789)
BG Mode:Calc. from Peak Group 1 - Event 1 Scan

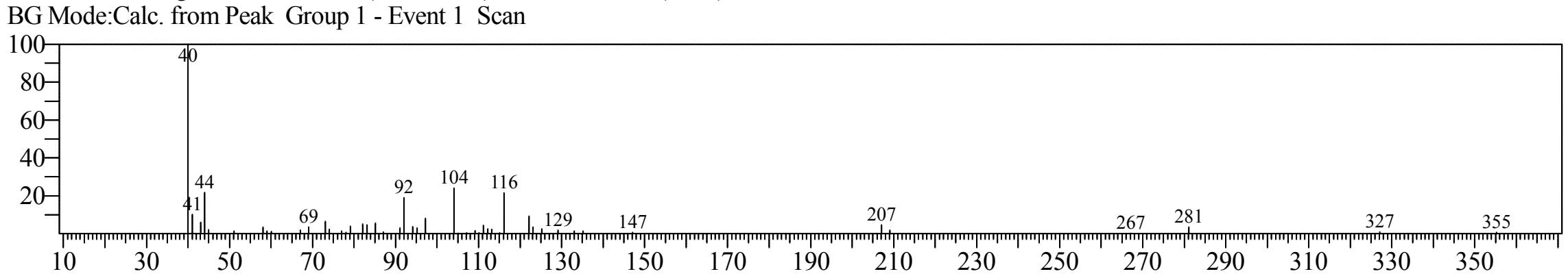

Hit#:6 Entry:12927 Library:WILEY7.LIB
SI:59 Formula:C5 H9 N O2 CAS:52855-96-6 MolWeight:115 RetIndex:0
CompName:5-METHOXY-1-AZA-6-OXABICYCLO(3.1.0)HEXANE $$ 6-Oxa-1-azabicyclo[3.1.0]hexane, 5-methoxy- (CAS)

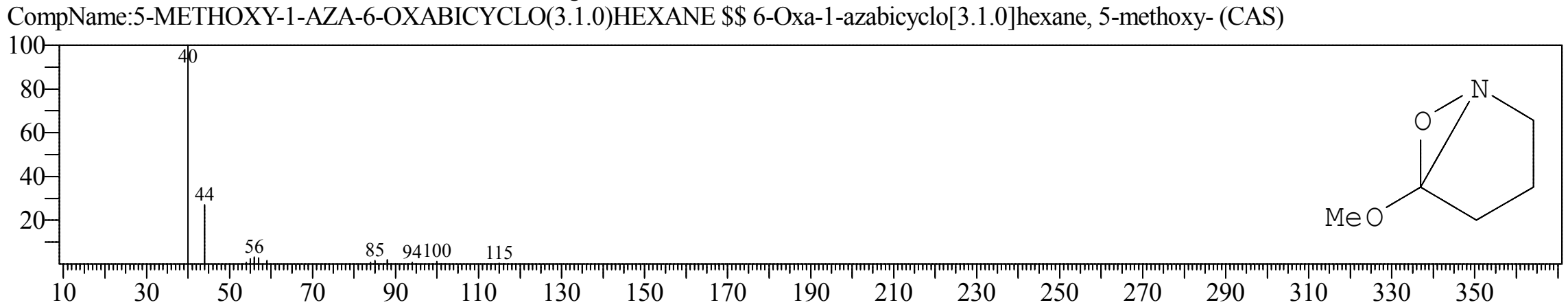

Hit#:7 Entry:6299 Library:WILEY7.LIB
SI:59 Formula:C6 H13 N CAS:0-00-0 MolWeight:99 RetIndex:0
CompName:1,2,2-Trimethylcyclopropylamine $$

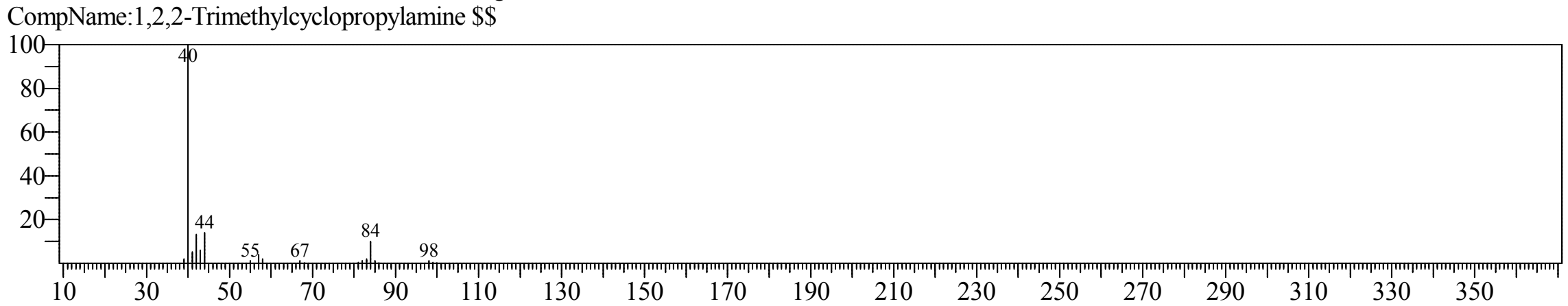

Hit#:8 Entry:181233 Library:WILEY7.LIB
SI:58 Formula:C8 H7 F3 O3 S2 CAS:96088-83-4 MolWeight:272 RetIndex:0
CompName:2-isopropylthio-5-trifluoracetyl-1,3-oxathiolyium-4-olat $$ 1,3-Oxathiol-1-ium, 4-hydroxy-2-[(1-methylethyl)thio]-5-(trifluoroacetyl)-, hydroxid

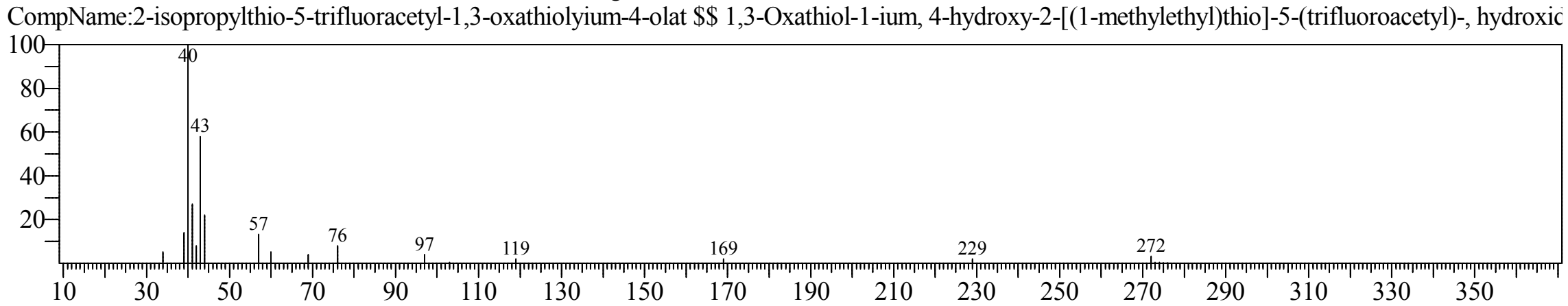

Hit#:9 Entry:11687 Library:WILEY7.LIB
SI:58 Formula:C3 H3 N3 O2 CAS:3034-38-6 MolWeight:113 RetIndex:0
CompName:4(5)-Nitroimidazole $$ 1H-Imidazole, 4-nitro- $$ Imidazole, 4-nitro- $$ 4-Nitroimidazole $$ 5-Nitroimidazole $$ Imidazole, 4(or 5)-nitro- $$

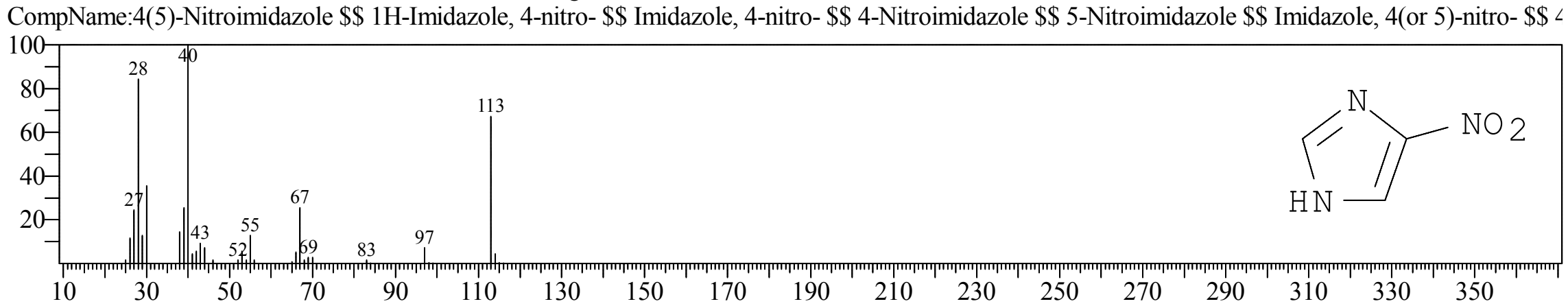

Hit#:10 Entry:19543 Library:WILEY7.LIB
SI:57 Formula:C5 H8 N2 O2 CAS:41138-17-4 MolWeight:128 RetIndex:0
CompName:2-Butenediamide, 2-methyl-, (Z)- (CAS) Citraconic diamide $$ Citraconamide $$ Maleamide, 2-methyl- $$

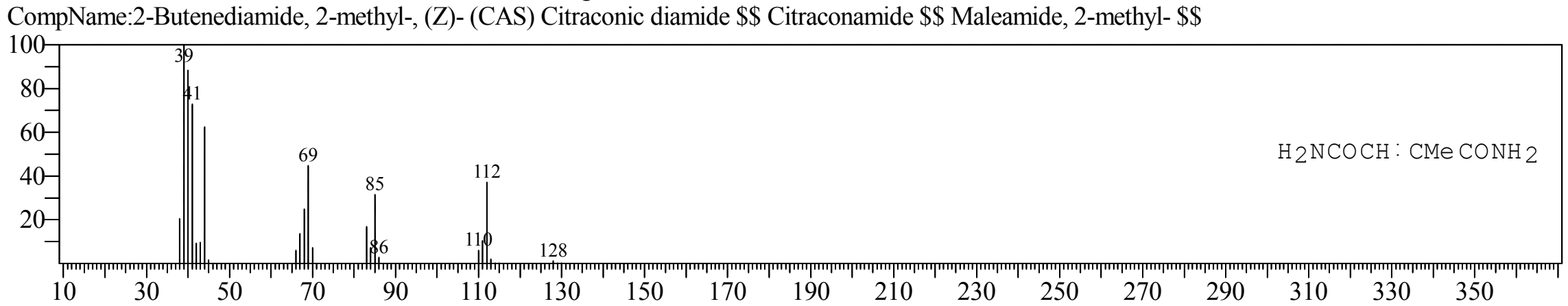

<< Target >>
Line#:30 R.Time:22.700(Scan#:2125) MassPeaks:49
RawMode:Averaged 22.692-22.708(2124-2126) BasePeak:40.00(3789)
BG Mode:Calc. from Peak Group 1 - Event 1 Scan

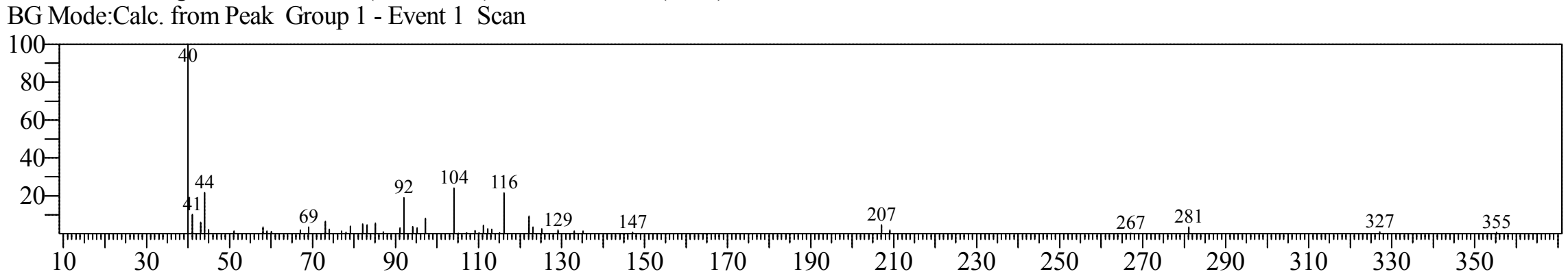

Hit#:11 Entry:11626 Library:WILEY7.LIB
SI:57 Formula:C6 H11 N O CAS:0-00-0 MolWeight:113 RetIndex:0
CompName:4-dimethylaminobut-2-yn-1-ol $$

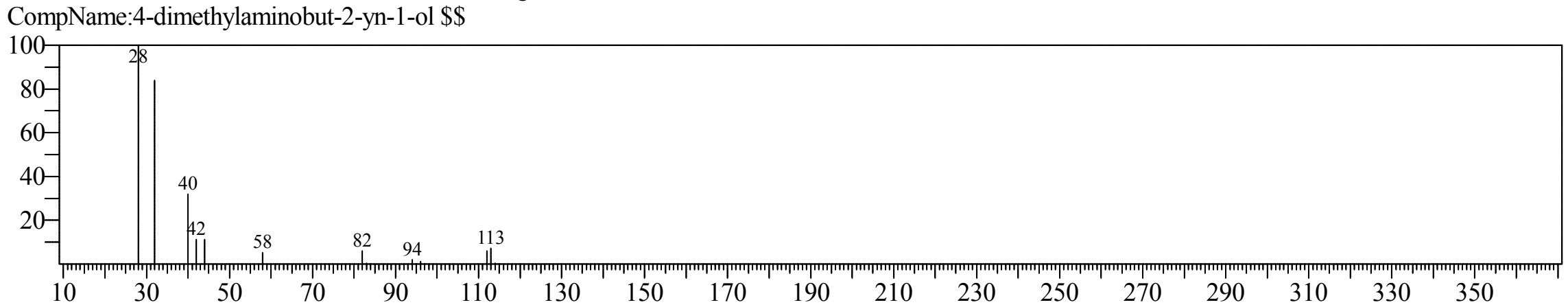

Hit#:12 Entry:202321 Library:WILEY7.LIB
SI:57 Formula:C20 H21 N O CAS:74235-54-4 MolWeight:291 RetIndex:0
CompName:3,3-DIMETHYL-2-PHENYL-2-(1-OXO-1,2,3,4-TETRAHYDRONAPHTHALEN-2-YL)AZIRANE $$ 1(2H)-Naphthalenone, 2-(3,3-dimethyl

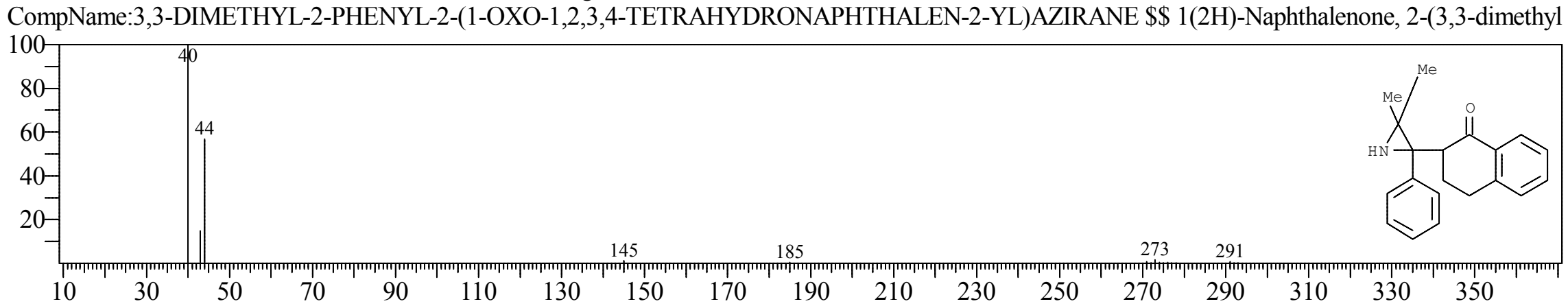

Hit#:13 Entry:178949 Library:WILEY7.LIB
SI:57 Formula:C8 H5 F3 O3 S2 CAS:96088-84-5 MolWeight:270 RetIndex:0
CompName:2-allythio-5-trifluoracetyl-1,3-oxathiolylium-4-olat $$ 1,3-Oxathiol-1-ium, 4-hydroxy-2-(2-propenylthio)-5-(trifluoroacetyl)-, hydroxide, inner sa

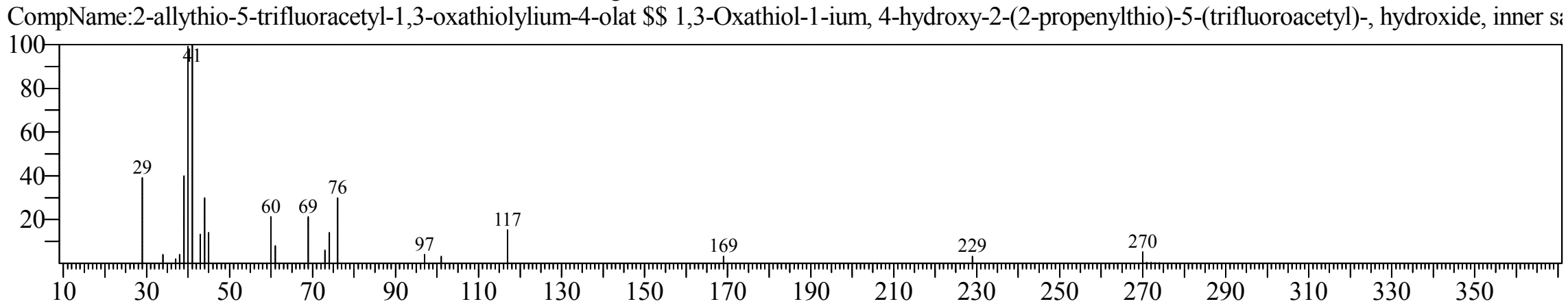

Hit#:14 Entry:196116 Library:WILEY7.LIB
SI:57 Formula:C18 H20 CL N CAS:99315-24-9 MolWeight:285 RetIndex:0
CompName:N-benzyl-3-chloro-2,2-dimethyl-propylimine $$ Benzenemethanamine, N-(3-chloro-2,2-dimethyl-1-phenylpropylidene)- (CAS)

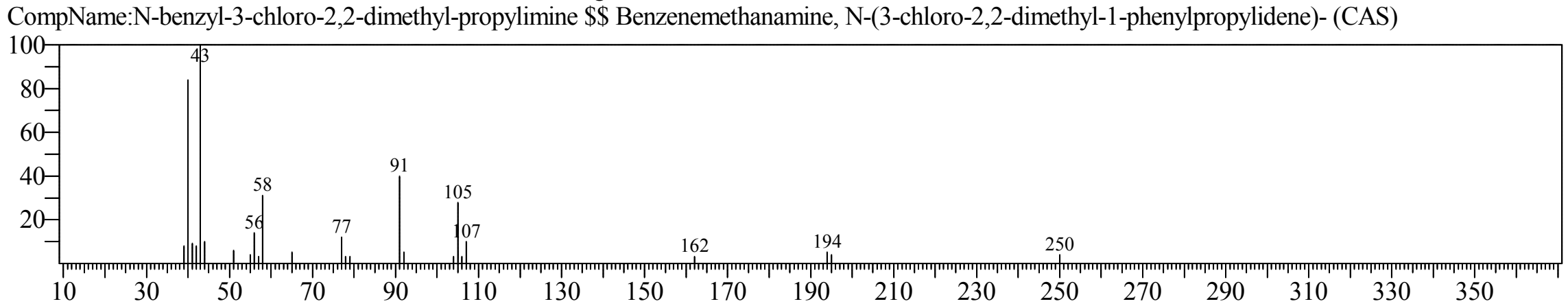

Hit#:15 Entry:97984 Library:WILEY7.LIB
SI:57 Formula:C7 H10 N2 O3 S CAS:1950-68-1 MolWeight:202 RetIndex:0
CompName:Benzenesulfonic acid, 4-methoxy-, hydrazide (CAS) p-Methoxybenzenesulfonylhydrazide $$ p-Methoxyphenylsulfonylhydrazine $$ p-Methoxy

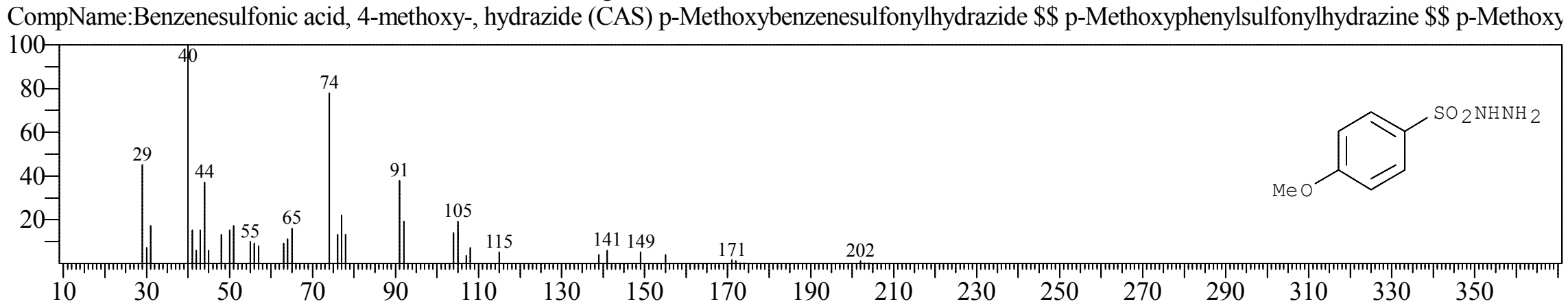

<< Target >>
Line#:30 R.Time:22.700(Scan#:2125) MassPeaks:49
RawMode:Averaged 22.692-22.708(2124-2126) BasePeak:40.00(3789)
BG Mode:Calc. from Peak Group 1 - Event 1 Scan

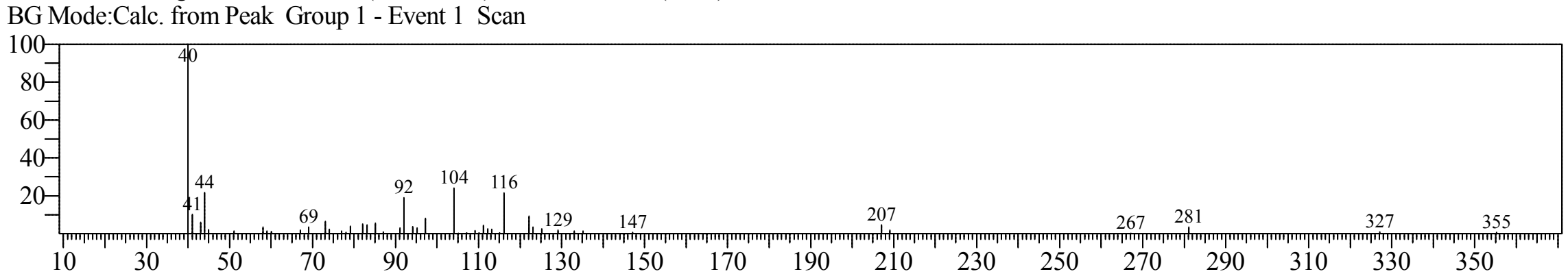

Hit#:16 Entry:247054 Library:WILEY7.LIB
SI:56 Formula:C19 H32 O5 CAS:80009-19-4 MolWeight:340 RetIndex:0
CompName:(Z)-Methyl-5-[(E)-3-(1-ethoxyethoxy)oct-1-en-1-yl]tetrahydrofuran-2-ylideneacetate $$ Acetic acid, [5-[3-(1-ethoxyethoxy)-1-octenyl]dihydro-

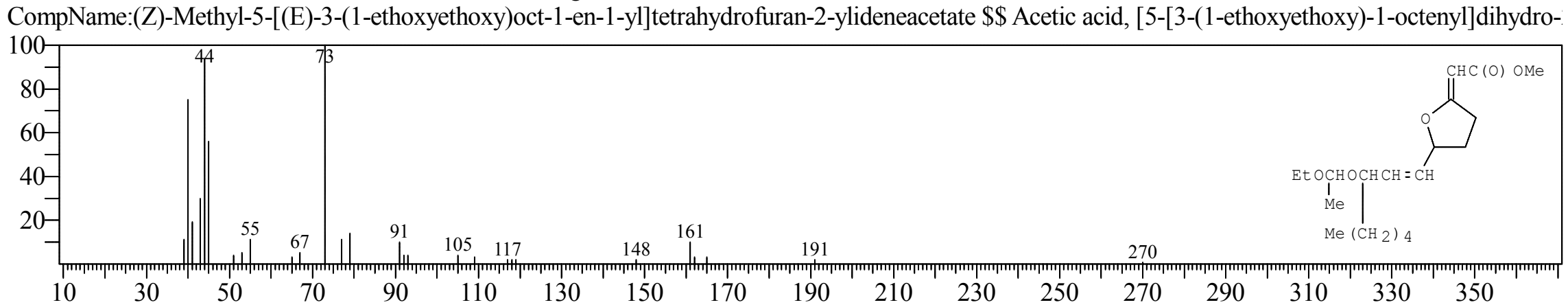

Hit#:17 Entry:10711 Library:WILEY7.LIB
SI:56 Formula:C6 H8 O2 CAS:0-00-0 MolWeight:112 RetIndex:0
CompName:2(5H)-Furanone, 3,5-dimethyl- $$

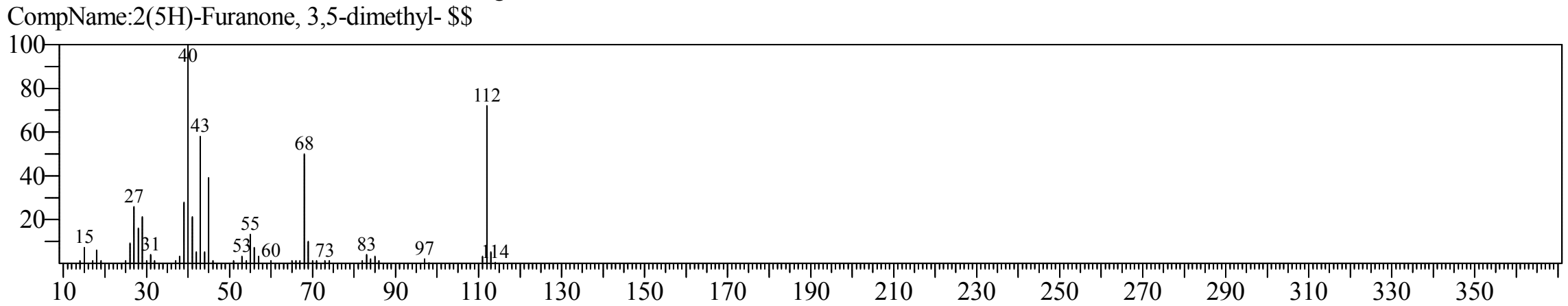

Hit#:18 Entry:106823 Library:NIST11.lib
SI:56 Formula:C18H20ClN CAS:0-00-0 MolWeight:285 RetIndex:2297
CompName:Benzylamine, N-(3-chloro-2,2-dimethyl-1-phenylpropylidene)- $$ N-[(E)-3-Chloro-2,2-dimethyl-1-phenylpropylidene](phenyl)methanamine # $

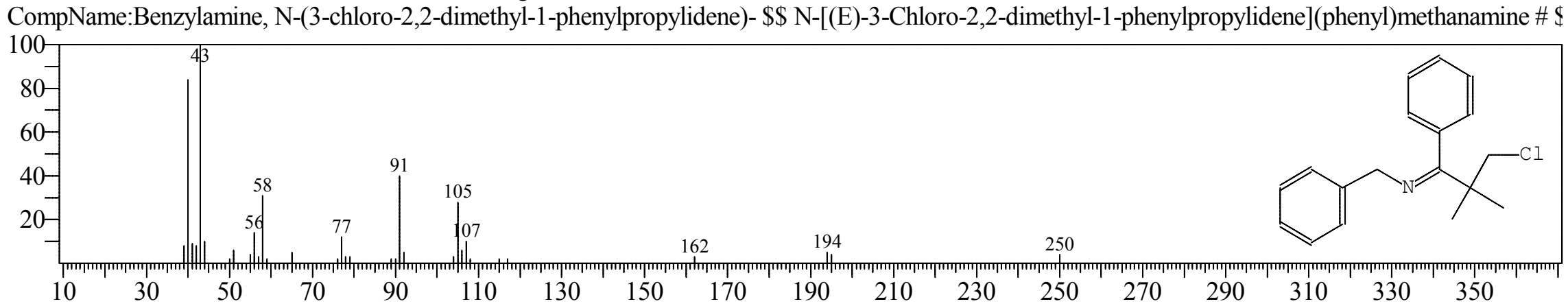

Hit#:19 Entry:92403 Library:NIST05.LIB
SI:56 Formula:C18H20ClN CAS:0-00-0 MolWeight:285 RetIndex:2297
CompName:Benzylamine, N-(3-chloro-2,2-dimethyl-1-phenylpropylidene)-

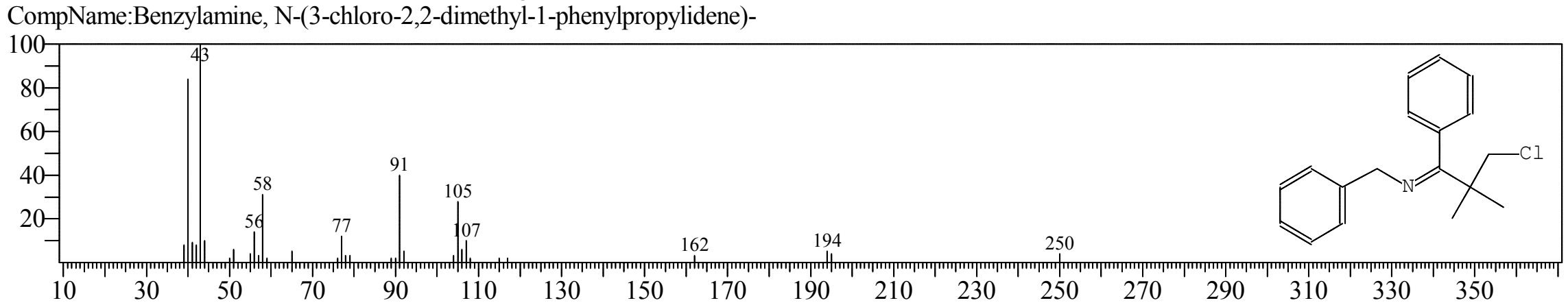

Hit#:20 Entry:19544 Library:WILEY7.LIB
SI:56 Formula:C5 H8 N2 O2 CAS:41138-18-5 MolWeight:128 RetIndex:0
CompName:2-Butenediamide, 2-methyl-, (E)- (CAS) Mesaconic diamide $$

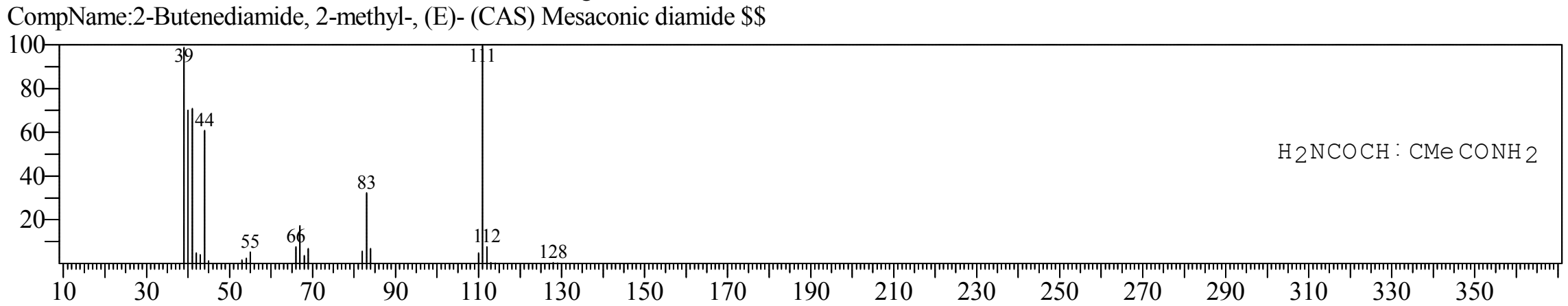

Peak Report

Library
