# Supplementary material for: Antioxidant, anti-inflammatory, cytotoxicity and larvicidal activities of Ziziphus oenopolia leaves: in vitro and in silico studies
Source: PLoS One. 2026 Oct 30;21(10):e0358577. doi: 10.1371/journal.pone.0358577 (PMC13722189; doi:10.1371/journal.pone.0358577)
Supplement: Supplementary file 3 [file pone.0358577.s005.docx]

**Response to Reviewers**

**Additional Editor Comments:**

Reviewer 1 found the manuscript technically sound, well-structured, and linguistically clear. The reviewer suggested only minor improvements, mainly related to figure resolution and refining keywords.
Reviewer 2 generally agreed that the study is suitable for publication but indicated several methodological and presentation-related improvements to strengthen its scientific rigor.

**Reviewer #1:**

In "the title": "Antioxidant, anti-inflammatory, cytotoxicity and larvicidal activities of Ziziphus oenopolia leaves: in vitro and in silico studies"
- It is rich in many scientific expressions and suitable to describe scope and content of the manuscript.

In "Abstract":
- It is written well according to instruction of the Journal. Moreover, it covered all general points discussed inside manuscript completely.

In "Keywords":
- The keywords are informative and suitable to describe contents of the manuscript.
- Do not use abbreviations as keywords. Please, replace the abbreviations by complete words.
- It is better to replace the keyword "Anti-inflammatory" by "Anti-inflammatory Activity".

In "Introduction":
- It is clear, simple and covered all required ideas about the techniques used to be under study.
- It is relatively suitable to other partitions of the manuscript.
- All the references used to illustrate the previous studies during this partition are recent and suitable to be embedded in the manuscript.

In "Materials and Methods":
- It is written well as clear, short and brief referenced paragraphs.
-It is clear and simple showing the methods used for the experimental work.
- It shows recent techniques used for undergoing the experiments and suitable to be published at this respect journal.

In "Results and Discussion":
- The manuscript contains many figures with low resolution. Please, re-formulate the figures with higher resolution.
- Arrangement of the figures and the data obtained are good and attractive.
- All references used to support the data obtained studies during this partition are recent and suitable to support the findings.
- It is relatively suitable to the other partitions of the manuscript.

In "Conclusions":
- It is simple, informative, summarized, and attractive. Moreover, it contains suitable recommendation.

In "References":
- All the references are written as one style following instruction of the journal for authors for illustrating the references. - Most of references are recent and suitable to support the findings.

**Response** : Thank you for your constructive and insightful comments, which have significantly improved the quality and clarity of the manuscript. All suggestions have been carefully considered and incorporated where appropriate. In particular, the resolution of all figures has been enhanced to meet publication standards in the revised version.

**Reviewer #2:**

 1. The botanical name of the plant Ziziphus oenopolia shall be suffixed with the taxonomist / Botanist name at its first occurrence in the Title (Example, Zea Mays L.).

**Response** : Thank you for your valuable comments. We have revised the entire manuscript as per your suggestions for *Ziziphus oenopolia* (L.). Mill, throughout the manuscript.

2. The introduction section is unclear and it needs to address the existing research gap, and how the study fulfils the research gap, hypothesis of the study, narrate the experiments carried out in the study at the last part of introduction, novelty of the study?

**Response** : Thank you for your valuable comment. The authors have revised the introduction section as per your suggestion.

3.The authors need to justify the selection of the plant by at least detailing the key compounds present in the plant through literature evidences, I would suggest to perform LCMS analysis of the plant extract to have a comprehensive profile of the compounds present in the plant extract. The authors can read, [https://doi.org/10.1186/s43094-024-00695-1](https://doi.org/10.1186/s43094-024-00695-1)

**Response** : Thank you for your valuable comment. We have included a detailed study of LCMS in the revised manuscript.

The LC–MS profiling of the *Z. oenopolia* leaves extract revealed the presence of diverse secondary metabolites, such as fatty acids, flavonoids, and phenolic derivatives (Figure 1). A total of eight compounds were tentatively annotated based on their accurate mass (m/z) values, retention behavior, and compared with previously reported literature. The chromatographic separation was performed on a reverse-phase C18 column, which enables the detection of bioactive compounds across a wide range of polarities. Notably, the peaks observed at retention times between 24 and 29 mins were attributed to phenolic acids such as caffeoylquinic acid (m/z 353) and rosmarinic acid (m/z 359), which are widely reported in the plant metabolite studies.

Flavonoid-related compounds such as luteolin C-hexoside (m/z 616), dimethoxy apigenin (m/Z 297) and monomethoxy-tetrahydroxy flavone (m/z 317), were also identified within the expected mass range for flavone derivatives and glycosylated flavonoids. However, structural confirmation of such metabolites typically relies on the fragmentation pathways, like the loss of sugar moieties or the formation of aglycon ions, which is not examined in the present analysis.

Organic acids and fatty acid derivatives, such as tartaric acid (m/z 149) and trihydroxy-octadecadienoic acid (m/z 327), further support the presence of primary and oxidized lipid metabolites. Nevertheless, the retention time of certain highly polar compounds, including tartaric acid, appears relatively delayed under the described chromatographic conditions, which may indicate co-elution effects or the presence of structurally related isomers. It is important that the compounds are annotated in this study by matching observed m/z values with the reported literature. In addition, the ionization mode and possible adduct formation were not explicitly considered, which may influence the observed m/z values for certain compounds.

4. All the chemicals and reagents used for this study need to be detailed with the purity percentage, CAS No., manufacturer details and the country of origin.

**Response** : Thank you for your suggestions. We have included the details of manufacturers, purity, and country of origin of the chemicals and reagents used in the revised manuscript.

5. All the equipment's / instruments used in the study need to be mentioned with the manufacturer, model and country of origin.

**Response** : Thank you for your valuable suggestions. We have included the details of the instruments in the revised manuscript.

6. The authors have performed a comprehensive qualitative phytochemical assays for the plant extract, certain important quantitative assays like total phenolic's, flavonoids, terpenoids, alkaloids etc. need to be performed and incorporated.

**Response** : Thank you for your valuable suggestion. The present qualitative test revealed the highest concentration of three major phytochemicals, such as polyphenol, flavonoids, and terpenoids; for that reason, the quantification was done using standard phytochemicals. As per your suggestions, we have incorporated the information in the revised manuscript.

7. In Table 2, the authors need to indicate the medicinal properties / various other properties of the compounds listed through supporting literature evidences (An additional column can be incorporated).

**Response** : Thank you for your valuable comment. We have included the medicinal properties/ various properties in Table 2, as per your suggestion.

8. All the figures are of poor resolution and it needs to be improved.

**Response** : Thank you for your valuable suggestion. We have improved the resolution of the figures as per your suggestions.

9. In all the biological assays, The standard control values need to be compared with the treatment values at various confidence intervals and the statistical significance need to indicated by (*) symbols with context to the P values. The P value need to be mentioned in the figure description.

**Response** : Thank you for your valuable comment. The authors have used one-way ANOVA with Tukey's HSD post-hoc test in the *Z. oenopolia leaves*' hydroalcoholic extract between the concentrations of 50 and 250 µg/mL. We used a statistical significance symbol (homogeneous subsets) in the cytotoxicity and larvicidal assay.
