# Supplementary material for: Antioxidant, anti-inflammatory, cytotoxicity and larvicidal activities of Ziziphus oenopolia leaves: in vitro and in silico studies
Source: PLoS One. 2026 Oct 30;21(10):e0358577. doi: 10.1371/journal.pone.0358577 (PMC13722189; doi:10.1371/journal.pone.0358577)
Supplement: Supplementary file 4 [file pone.0358577.s003.doc]

**In "the title":**

"Antioxidant, anti-inflammatory, cytotoxicity and larvicidal activities of *Ziziphus oenopolia* leaves: *in vitro* and *in silico* studies"

- It is rich in many scientific expressions and suitable to describe scope and content of the manuscript.

**In "Abstract":**

- It is written well according to instruction of the Journal. Moreover, it covered all general points discussed inside manuscript completely.

**In "Keywords":**

- The keywords are informative and suitable to describe contents of the manuscript.

- Do not use abbreviations as keywords. Please, replace the abbreviations by complete words.

- It is better to replace the keyword "Anti-inflammatory" by "Anti-inflammatory Activity".

**In "Introduction":**

- It is clear, simple and covered all required ideas about the techniques used to be under study.

- It is relatively suitable to other partitions of the manuscript.

- All the references used to illustrate the previous studies during this partition are recent and suitable to be embedded in the manuscript.

**In "Materials and Methods":**

- It is written well as clear, short and brief referenced paragraphs.

-It is clear and simple showing the methods used for the experimental work.

- It shows recent techniques used for undergoing the experiments and suitable to be published at this respect journal.

**In "Results and Discussion":**

- The manuscript contains many figures with low resolution. Please, re-formulate the figures with higher resolution.

- Arrangement of the figures and the data obtained are good and attractive.

- All references used to support the data obtained studies during this partition are recent and suitable to support the findings.

- It is relatively suitable to the other partitions of the manuscript.

**In "Conclusions":**

- It is simple, informative, summarized, and attractive. Moreover, it contains suitable recommendation.

**In "References":**

- All the references are written as one style following instruction of the journal for authors for illustrating the references.

- Most of references are recent and suitable to support the findings.

**Recommendation**

- According to my own point of view, the manuscript is good and shows innovative and promising results belongs to scope of your respect and great journal. I think the scientific idea covered well during the manuscript.

- The manuscript accepted to be published in your journal after making the minor corrections.
