# Supplementary material for: Antioxidant, anti-inflammatory, cytotoxicity and larvicidal activities of Ziziphus oenopolia leaves: in vitro and in silico studies
Source: PLoS One. 2026 Oct 30;21(10):e0358577. doi: 10.1371/journal.pone.0358577 (PMC13722189; doi:10.1371/journal.pone.0358577)
Supplement: Peer review history

# Peer review: Decision Letter 0

**DOI:** 10.1371/journal.pone.0358577.r001

**Authors:** Gayathiri Ekambaram

4 Jan 2026

Dear Dr. Lo,

Thank you for submitting your manuscript to PLOS ONE. After careful consideration, we feel that it has merit but does not fully meet PLOS ONE’s publication criteria as it currently stands. Therefore, we invite you to submit a revised version of the manuscript that addresses the points raised during the review process.

Please submit your revised manuscript by Feb 16 2026 11:59PM. If you will need more time than this to complete your revisions, please reply to this message or contact the journal office at plosone@plos.org. When you're ready to submit your revision, log on to https://www.editorialmanager.com/pone/ and select the 'Submissions Needing Revision' folder to locate your manuscript file.

- A letter that responds to each point raised by the academic editor and reviewer(s). You should upload this letter as a separate file labeled 'Response to Reviewers'.
- A marked-up copy of your manuscript that highlights changes made to the original version. You should upload this as a separate file labeled 'Revised Manuscript with Track Changes'.
- An unmarked version of your revised paper without tracked changes. You should upload this as a separate file labeled 'Manuscript'.

If you would like to make changes to your financial disclosure, please include your updated statement in your cover letter. Guidelines for resubmitting your figure files are available below the reviewer comments at the end of this letter.

If applicable, we recommend that you deposit your laboratory protocols in protocols.io to enhance the reproducibility of your results. Protocols.io assigns your protocol its own identifier (DOI) so that it can be cited independently in the future. For instructions see: https://journals.plos.org/plosone/s/submission-guidelines#loc-laboratory-protocols. Additionally, PLOS ONE offers an option for publishing peer-reviewed Lab Protocol articles, which describe protocols hosted on protocols.io. Read more information on sharing protocols at https://plos.org/protocols?utm_medium=editorial-email&utm_source=authorletters&utm_campaign=protocols.

We look forward to receiving your revised manuscript.

Kind regards,

Gayathiri Ekambaram, Ph.D

Academic Editor

PLOS One

Journal Requirements:

When submitting your revision, we need you to address these additional requirements.

1. Please ensure that your manuscript meets PLOS ONE's style requirements, including those for file naming. The PLOS ONE style templates can be found at

https://journals.plos.org/plosone/s/file?id=wjVg/PLOSOne_formatting_sample_main_body.pdf and

https://journals.plos.org/plosone/s/file?id=ba62/PLOSOne_formatting_sample_title_authors_affiliations.pdf

2. We note that your Data Availability Statement is currently as follows: The conducted research is not related to either human or animal use.

Please confirm at this time whether or not your submission contains all raw data required to replicate the results of your study. Authors must share the “minimal data set” for their submission. PLOS defines the minimal data set to consist of the data required to replicate all study findings reported in the article, as well as related metadata and methods (https://journals.plos.org/plosone/s/data-availability#loc-minimal-data-set-definition).

For example, authors should submit the following data:

- The values behind the means, standard deviations and other measures reported;

- The values used to build graphs;

- The points extracted from images for analysis.

Authors do not need to submit their entire data set if only a portion of the data was used in the reported study.

If your submission does not contain these data, please either upload them as Supporting Information files or deposit them to a stable, public repository and provide us with the relevant URLs, DOIs, or accession numbers. For a list of recommended repositories, please see https://journals.plos.org/plosone/s/recommended-repositories.

If there are ethical or legal restrictions on sharing a de-identified data set, please explain them in detail (e.g., data contain potentially sensitive information, data are owned by a third-party organization, etc.) and who has imposed them (e.g., an ethics committee). Please also provide contact information for a data access committee, ethics committee, or other institutional body to which data requests may be sent. If data are owned by a third party, please indicate how others may request data access.

3. If the reviewer comments include a recommendation to cite specific previously published works, please review and evaluate these publications to determine whether they are relevant and should be cited. There is no requirement to cite these works unless the editor has indicated otherwise.

4. Please review your reference list to ensure that it is complete and correct. If you have cited papers that have been retracted, please include the rationale for doing so in the manuscript text, or remove these references and replace them with relevant current references. Any changes to the reference list should be mentioned in the rebuttal letter that accompanies your revised manuscript. If you need to cite a retracted article, indicate the article’s retracted status in the References list and also include a citation and full reference for the retraction notice.

**Additional Editor Comments:**

Reviewer 1 found the manuscript technically sound, well-structured, and linguistically clear. The reviewer suggested only minor improvements, mainly related to figure resolution and refining keywords.

Reviewer 2 generally agreed that the study is suitable for publication but indicated several methodological and presentation-related improvements to strengthen its scientific rigor.

[Note: HTML markup is below. Please do not edit.]

Reviewers' comments:

Reviewer's Responses to Questions

**Comments to the Author**

1. Is the manuscript technically sound, and do the data support the conclusions?

Reviewer #1: Yes

Reviewer #2: Yes

**********

2. Has the statistical analysis been performed appropriately and rigorously?

Reviewer #1: Yes

Reviewer #2: No

**********

3. Have the authors made all data underlying the findings in their manuscript fully available?

The PLOS Data policy

Reviewer #1: Yes

Reviewer #2: Yes

**********

4. Is the manuscript presented in an intelligible fashion and written in standard English?

Reviewer #1: Yes

Reviewer #2: Yes

**********

Reviewer #1: In "the title":

"Antioxidant, anti-inflammatory, cytotoxicity and larvicidal activities of Ziziphus oenopolia leaves: in vitro and in silico studies"

- It is rich in many scientific expressions and suitable to describe scope and content of the manuscript.

In "Abstract":

- It is written well according to instruction of the Journal. Moreover, it covered all general points discussed inside manuscript completely.

In "Keywords":

- The keywords are informative and suitable to describe contents of the manuscript.

- Do not use abbreviations as keywords. Please, replace the abbreviations by complete words.

- It is better to replace the keyword "Anti-inflammatory" by "Anti-inflammatory Activity".

In "Introduction":

- It is clear, simple and covered all required ideas about the techniques used to be under study.

- It is relatively suitable to other partitions of the manuscript.

- All the references used to illustrate the previous studies during this partition are recent and suitable to be embedded in the manuscript.

In "Materials and Methods":

- It is written well as clear, short and brief referenced paragraphs.

-It is clear and simple showing the methods used for the experimental work.

- It shows recent techniques used for undergoing the experiments and suitable to be published at this respect journal.

In "Results and Discussion":

- The manuscript contains many figures with low resolution. Please, re-formulate the figures with higher resolution.

- Arrangement of the figures and the data obtained are good and attractive.

- All references used to support the data obtained studies during this partition are recent and suitable to support the findings.

- It is relatively suitable to the other partitions of the manuscript.

In "Conclusions":

- It is simple, informative, summarized, and attractive. Moreover, it contains suitable recommendation.

In "References":

- All the references are written as one style following instruction of the journal for authors for illustrating the references.

- Most of references are recent and suitable to support the findings.

Reviewer #2: 1. The botanical name of the plant Ziziphus oenopolia shall be suffixed with the taxonomist / Botanist name at its first occurrence in the Title (Example, Zea Mays L.)

2. The introduction section is unclear and it needs to address the existing research gap, and how the study fulfils the research gap, hypothesis of the study, narrate the experiments carried out in the study at the last part of introduction, novelty of the study?

3.The authors need to justify the selection of the plant by at least detailing the key compounds present in the plant through literature evidences, I would suggest to perform LCMS analysis of the plant extract to have a comprehensive profile of the compounds present in the plant extract. The authors can read, https://doi.org/10.1186/s43094-024-00695-1

4. All the chemicals and reagents used for this study need to be detailed with the purity percentage, CAS No., manufacturer details and the country of origin.

5. All the equipment's / instruments used in the study need to be mentioned with the manufacturer, model and country of origin.

6. The authors have performed a comprehensive qualitative phytochemical assays for the plant extract, certain important quantitative assays like total phenolic's, flavonoids, terpenoids, alkaloids etc. need to be performed and incorporated.

7. In Table 2, the authors need to indicate the medicinal properties / various other properties of the compounds listed through supporting literature evidences (An additional column can be incorporated).

8. All the figures are of poor resolution and it needs to be improved.

9. In all the biological assays, The standard control values need to be compared with the treatment values at various confidence intervals and the statistical significance need to indicated by (*) symbols with context to the P values. The P value need to be mentioned in the figure description

**********

what does this mean?). If published, this will include your full peer review and any attached files.

If you choose “no”, your identity will remain anonymous but your review may still be made public.

**Do you want your identity to be public for this peer review?**  For information about this choice, including consent withdrawal, please see our Privacy Policy

Reviewer #1: **Yes:**  Asst. Prof. Wael Mahmoud Aboulthana

Biochemistry Department

Biotechnology Research Institute

National Research Centre

Reviewer #2: No

**********

[NOTE: If reviewer comments were submitted as an attachment file, they will be attached to this email and accessible via the submission site. Please log into your account, locate the manuscript record, and check for the action link "View Attachments". If this link does not appear, there are no attachment files.]

To ensure your figures meet our technical requirements, please review our figure guidelines: https://journals.plos.org/plosone/s/figures

You may also use PLOS’s free figure tool, NAAS, to help you prepare publication quality figures: https://journals.plos.org/plosone/s/figures#loc-tools-for-figure-preparation.

NAAS will assess whether your figures meet our technical requirements by comparing each figure against our figure specifications.

Attachment Submitted filename: Recommendation.doc

# Author comment: Author response to Decision Letter 1

**DOI:** 10.1371/journal.pone.0358577.r002

27 Apr 2026

Response to Reviewers

Additional Editor Comments:

Reviewer 1 found the manuscript technically sound, well-structured, and linguistically clear. The reviewer suggested only minor improvements, mainly related to figure resolution and refining keywords.

Reviewer 2 generally agreed that the study is suitable for publication but indicated several methodological and presentation-related improvements to strengthen its scientific rigor.

Reviewer #1:

In "the title": "Antioxidant, anti-inflammatory, cytotoxicity and larvicidal activities of Ziziphus oenopolia leaves: in vitro and in silico studies"

- It is rich in many scientific expressions and suitable to describe scope and content of the manuscript.

In "Abstract":

- It is written well according to instruction of the Journal. Moreover, it covered all general points discussed inside manuscript completely.

In "Keywords":

- The keywords are informative and suitable to describe contents of the manuscript.

- Do not use abbreviations as keywords. Please, replace the abbreviations by complete words.

- It is better to replace the keyword "Anti-inflammatory" by "Anti-inflammatory Activity".

In "Introduction":

- It is clear, simple and covered all required ideas about the techniques used to be under study.

- It is relatively suitable to other partitions of the manuscript.

- All the references used to illustrate the previous studies during this partition are recent and suitable to be embedded in the manuscript.

In "Materials and Methods":

- It is written well as clear, short and brief referenced paragraphs.

-It is clear and simple showing the methods used for the experimental work.

- It shows recent techniques used for undergoing the experiments and suitable to be published at this respect journal.

In "Results and Discussion":

- The manuscript contains many figures with low resolution. Please, re-formulate the figures with higher resolution.

- Arrangement of the figures and the data obtained are good and attractive.

- All references used to support the data obtained studies during this partition are recent and suitable to support the findings.

- It is relatively suitable to the other partitions of the manuscript.

In "Conclusions":

- It is simple, informative, summarized, and attractive. Moreover, it contains suitable recommendation.

In "References":

- All the references are written as one style following instruction of the journal for authors for illustrating the references. - Most of references are recent and suitable to support the findings.

Response : Thank you for your constructive and insightful comments, which have significantly improved the quality and clarity of the manuscript. All suggestions have been carefully considered and incorporated where appropriate. In particular, the resolution of all figures has been enhanced to meet publication standards in the revised version.

Reviewer #2:

1. The botanical name of the plant Ziziphus oenopolia shall be suffixed with the taxonomist / Botanist name at its first occurrence in the Title (Example, Zea Mays L.).

Response : Thank you for your valuable comments. We have revised the entire manuscript as per your suggestions for Ziziphus oenopolia (L.). Mill, throughout the manuscript.

2. The introduction section is unclear and it needs to address the existing research gap, and how the study fulfils the research gap, hypothesis of the study, narrate the experiments carried out in the study at the last part of introduction, novelty of the study?

Response : Thank you for your valuable comment. The authors have revised the introduction section as per your suggestion.

3.The authors need to justify the selection of the plant by at least detailing the key compounds present in the plant through literature evidences, I would suggest to perform LCMS analysis of the plant extract to have a comprehensive profile of the compounds present in the plant extract. The authors can read, https://doi.org/10.1186/s43094-024-00695-1

Response : Thank you for your valuable comment. We have included a detailed study of LCMS in the revised manuscript.

The LC–MS profiling of the Z. oenopolia leaves extract revealed the presence of diverse secondary metabolites, such as fatty acids, flavonoids, and phenolic derivatives (Figure 1). A total of eight compounds were tentatively annotated based on their accurate mass (m/z) values, retention behavior, and compared with previously reported literature. The chromatographic separation was performed on a reverse-phase C18 column, which enables the detection of bioactive compounds across a wide range of polarities. Notably, the peaks observed at retention times between 24 and 29 mins were attributed to phenolic acids such as caffeoylquinic acid (m/z 353) and rosmarinic acid (m/z 359), which are widely reported in the plant metabolite studies.

Flavonoid-related compounds such as luteolin C-hexoside (m/z 616), dimethoxy apigenin (m/Z 297) and monomethoxy-tetrahydroxy flavone (m/z 317), were also identified within the expected mass range for flavone derivatives and glycosylated flavonoids. However, structural confirmation of such metabolites typically relies on the fragmentation pathways, like the loss of sugar moieties or the formation of aglycon ions, which is not examined in the present analysis.

Organic acids and fatty acid derivatives, such as tartaric acid (m/z 149) and trihydroxy-octadecadienoic acid (m/z 327), further support the presence of primary and oxidized lipid metabolites. Nevertheless, the retention time of certain highly polar compounds, including tartaric acid, appears relatively delayed under the described chromatographic conditions, which may indicate co-elution effects or the presence of structurally related isomers. It is important that the compounds are annotated in this study by matching observed m/z values with the reported literature. In addition, the ionization mode and possible adduct formation were not explicitly considered, which may influence the observed m/z values for certain compounds.

4. All the chemicals and reagents used for this study need to be detailed with the purity percentage, CAS No., manufacturer details and the country of origin.

Response : Thank you for your suggestions. We have included the details of manufacturers, purity, and country of origin of the chemicals and reagents used in the revised manuscript.

5. All the equipment's / instruments used in the study need to be mentioned with the manufacturer, model and country of origin.

Response : Thank you for your valuable suggestions. We have included the details of the instruments in the revised manuscript.

6. The authors have performed a comprehensive qualitative phytochemical assays for the plant extract, certain important quantitative assays like total phenolic's, flavonoids, terpenoids, alkaloids etc. need to be performed and incorporated.

Response : Thank you for your valuable suggestion. The present qualitative test revealed the highest concentration of three major phytochemicals, such as polyphenol, flavonoids, and terpenoids; for that reason, the quantification was done using standard phytochemicals. As per your suggestions, we have incorporated the information in the revised manuscript.

7. In Table 2, the authors need to indicate the medicinal properties / various other properties of the compounds listed through supporting literature evidences (An additional column can be incorporated).

Response : Thank you for your valuable comment. We have included the medicinal properties/ various properties in Table 2, as per your suggestion.

8. All the figures are of poor resolution and it needs to be improved.

Response : Thank you for your valuable suggestion. We have improved the resolution of the figures as per your suggestions.

9. In all the biological assays, The standard control values need to be compared with the treatment values at various confidence intervals and the statistical significance need to indicated by (*) symbols with context to the P values. The P value need to be mentioned in the figure description.

Response : Thank you for your valuable comment. The authors have used one-way ANOVA with Tukey's HSD post-hoc test in the Z. oenopolia leaves' hydroalcoholic extract between the concentrations of 50 and 250 µg/mL. We used a statistical significance symbol (homogeneous subsets) in the cytotoxicity and larvicidal assay.

Attachment Submitted filename: Response to Reviewers.docx

# Peer review: Decision Letter 1

**DOI:** 10.1371/journal.pone.0358577.r003

**Authors:** Gayathiri Ekambaram

2 Sep 2026

Antioxidant, anti-inflammatory, cytotoxicity and larvicidal activities of Ziziphus oenopolia leaves: in vitro and in silico studies

PONE-D-25-60707R1

Dear Dr. Lo,

We’re pleased to inform you that your manuscript has been judged scientifically suitable for publication and will be formally accepted for publication once it meets all outstanding technical requirements.

Within one week, you’ll receive an e-mail detailing the required amendments. When these have been addressed, you’ll receive a formal acceptance letter and your manuscript will be scheduled for publication.

An invoice will be generated when your article is formally accepted. Please note, if your institution has a publishing partnership with PLOS and your article meets the relevant criteria, all or part of your publication costs will be covered. Please make sure your user information is up-to-date by logging into Editorial Manager at Editorial Manager® and clicking the ‘Update My Information' link at the top of the page. For questions related to billing, please contact billing support.

If your institution or institutions have a press office, please notify them about your upcoming paper to help maximize its impact. If they’ll be preparing press materials, please inform our press team as soon as possible -- no later than 48 hours after receiving the formal acceptance. Your manuscript will remain under strict press embargo until 2 pm Eastern Time on the date of publication. For more information, please contact onepress@plos.org.

Kind regards,

Gayathiri Ekambaram, Ph.D

Academic Editor

PLOS One

Additional Editor Comments (optional):

I am pleased to inform you that your revised manuscript PONE-D-25-60707R1, titled "Antioxidant, anti-inflammatory, cytotoxicity and larvicidal activities of Ziziphus oenopolia leaves: in vitro and in silico studies", has been evaluated following the re-review process. Both reviewers have confirmed that all previous comments and concerns have been comprehensively addressed. They have noted that the manuscript is technically sound, the data and statistical analyses support the conclusions, and the presentation meets the publication standards of PLOS ONE. Therefore, I am delighted to accept your manuscript for publication in PLOS ONE in its present form.

Reviewers' comments:

Reviewer's Responses to Questions

**Comments to the Author**

Reviewer #3: All comments have been addressed

Reviewer #4: All comments have been addressed

**********

2. Is the manuscript technically sound, and do the data support the conclusions?

Reviewer #3: Yes

Reviewer #4: Yes

**********

3. Has the statistical analysis been performed appropriately and rigorously?

Reviewer #3: Yes

Reviewer #4: Yes

**********

4. Have the authors made all data underlying the findings in their manuscript fully available?

The PLOS Data policy

Reviewer #3: Yes

Reviewer #4: Yes

**********

5. Is the manuscript presented in an intelligible fashion and written in standard English?

Reviewer #3: Yes

Reviewer #4: Yes

**********

Reviewer #3: Dear author,

All previous comments have been addressed. The manuscript is technically sound, clearly presented, and suitable for publication in its current form.

Reviewer #4: The authors have addressed the comments raised by the previous reviewers. The revised manuscript has been improved and the responses provided by the authors are satisfactory.

I have no further major comments or concerns. Therefore, I recommend that the manuscript be accepted for publication in its present form.

**********

what does this mean?). If published, this will include your full peer review and any attached files.

If you choose “no”, your identity will remain anonymous but your review may still be made public.

**Do you want your identity to be public for this peer review?**  For information about this choice, including consent withdrawal, please see our Privacy Policy

Reviewer #3: No

Reviewer #4: No

**********

# Editor report: Acceptance letter

**DOI:** 10.1371/journal.pone.0358577.r004

**Authors:** Gayathiri Ekambaram

PONE-D-25-60707R1

PLOS One

Dear Dr. Lo,

I'm pleased to inform you that your manuscript has been deemed suitable for publication in PLOS One. Congratulations! Your manuscript is now being handed over to our production team.

At this stage, our production department will prepare your paper for publication. This includes ensuring the following:

* All references, tables, and figures are properly cited

* All relevant supporting information is included in the manuscript submission,

* There are no issues that prevent the paper from being properly typeset

You will receive further instructions from the production team, including instructions on how to review your proof when it is ready. Please keep in mind that we are working through a large volume of accepted articles, so please give us a few days to review your paper and let you know the next and final steps.

Lastly, if your institution or institutions have a press office, please let them know about your upcoming paper now to help maximize its impact. If they'll be preparing press materials, please inform our press team within the next 48 hours. Your manuscript will remain under strict press embargo until 2 pm Eastern Time on the date of publication. For more information, please contact onepress@plos.org.

You will receive an invoice from PLOS for your publication fee after your manuscript has reached the completed accept phase. If you receive an email requesting payment before acceptance or for any other service, this may be a phishing scheme. Learn how to identify phishing emails and protect your accounts at https://explore.plos.org/phishing.

If we can help with anything else, please email us at customercare@plos.org.

Thank you for submitting your work to PLOS One and supporting open access.

Kind regards,

PLOS One Editorial Office Staff

on behalf of

Dr. Gayathiri Ekambaram

Academic Editor

PLOS One
